# CRISPR-Cas9 genetic screens reveal regulation of TMPRSS2 by the Elongin BC-VHL complex

**DOI:** 10.1038/s41598-025-95644-0

**Published:** 2025-04-07

**Authors:** Ildar Gabaev, Alexandra Rowland, Emilija Jovanovic, Christian M. Gawden-Bone, Thomas W. M. Crozier, Ana Teixeira-Silva, Edward J. D. Greenwood, Pehuén Pereyra Gerber, Niek Wit, James A. Nathan, Nicholas J. Matheson, Paul J. Lehner

**Affiliations:** 1 Department of Medicine, University of Cambridge, Hills Road, Cambridge, CB2 0QQ UK; 2 Cambridge Institute for Therapeutic Immunology and Infectious Disease (CITIID), University of Cambridge, Puddicombe Way, Cambridge, CB2 0AW UK; 3 Wellcome-MRC Cambridge Stem Cell Institute, University of Cambridge, Puddicombe Way, Cambridge, CB2 0AW UK; 4 NHS Blood and Transplant, Cambridge, UK

**Keywords:** Transmembrane serine proteases, Coronavirus entry factors, CRISPR-Cas9 screen, Colon epithelial cells, Hypoxic regulation of surface proteins, Viral infection, Gene expression, SARS-CoV-2, CRISPR-Cas9 genome editing

## Abstract

The TMPRSS2 cell surface protease is used by a broad range of respiratory viruses to facilitate entry into target cells. Together with ACE2, TMPRSS2 represents a key factor for SARS-CoV-2 infection, as TMPRSS2 mediates cleavage of viral spike protein, enabling direct fusion of the viral envelope with the host cell membrane. Since the start of the COVID-19 pandemic, TMPRSS2 has gained attention as a therapeutic target for protease inhibitors which would inhibit SARS-CoV-2 infection, but little is known about TMPRSS2 regulation, particularly in cell types physiologically relevant for SARS-CoV-2 infection. Here, we performed an unbiased genome-wide CRISPR-Cas9 library screen, together with a library targeted at epigenetic modifiers and transcriptional regulators, to identify cellular factors that modulate cell surface expression of TMPRSS2 in human colon epithelial cells. We find that endogenous TMPRSS2 is regulated by the Elongin BC-VHL complex and HIF transcription factors. Depletion of Elongin B or treatment of cells with PHD inhibitors resulted in downregulation of TMPRSS2 and inhibition of SARS-CoV-2 infection. We show that TMPRSS2 is still utilised by SARS-CoV-2 Omicron variants for entry into colonic epithelial cells. Our study enhances our understanding of the regulation of endogenous surface TMPRSS2 in cells physiologically relevant to SARS-CoV-2 infection.

## Introduction

TMPRSS2 is a type II transmembrane serine protease (TTSP) and a member of a large family of 17 proteins, many of which play important roles in the regulation of the immune response as well as the cardiac and gastrointestinal system^1–3^. While the exact biological role of TMPRSS2 remains unclear, multiple studies have demonstrated how TMPRSS2 is appropriated by respiratory viruses to facilitate their entry into host cells^4^. Over the last two decades TMPRSS2 has been shown to proteolytically activate the membrane fusion proteins of influenza viruses^5–7^, parainfluenza and Sendai virus^8^, metapneumovirus^9^, MERS^10^, SARS-CoV^11^ and most recently, SARS-CoV-2^12^. Recently, TMPRSS2 has also been shown to be a functional receptor for human coronavirus HKU1^13–17^.

Together with angiotensin-converting enzyme 2 (ACE2), TMPRSS2 is well-established as a key host factor for SARS-CoV-2 cell entry and pathogenesis^12,18^. While ACE2 determines the permissiveness of target cells for SARS-CoV-2 entry as well as tropism^19–21^, TMPRSS2 dictates the route of virus entry into host cells. SARS-CoV-2 enters TMPRSS2-positive cells following fusion with the plasma membrane, whereas entry into TMPRSS2-negative cells is via the endocytic pathway^22,23^. TMPRSS2-mediated proteolytic cleavage of the SARS-CoV-2 Spike protein within the S2 subunit exposes the fusion peptide which allows the virion to fuse with the plasma membrane^12,24^. Although several reports have indicated that SARS-CoV-2 Omicron variants have an attenuated TMPRSS2 requirement for cell entry in tissue culture cells^25–27^ or utilise TMPRSS2-independent entry into nasal epithelial cells^28^, recent in vivo studies with TMPRSS2 knockout mice demonstrated that TMPRSS2 also plays an important role in the spread of Omicron variants in the respiratory tract^18,29^.

For many coronaviruses, including SARS-CoV-2, utilising this TMPRSS2-dependent route, not only allows direct entry into the cell via membrane fusion, but also helps avoid important endosomal innate immune restriction factors such as members of the IFITM protein family^30–34^. Indeed, endosomal IFITM2 and IFITM3 restrict SARS-CoV-2 infection in TMPRSS2-negative human cells^35,36^. Conversely, overexpression of TMPRSS2 attenuates IFITM3-mediated restriction of SARS-CoV-2^36,37^. Furthermore, in TMPRSS2-positive human lung cells, eg Calu-3 and iPSC-derived alveolar type II cells^22,38^, the endogenous IFITM2 and IFITM3 proteins do not act as restriction factors, but positive regulators of SARS-CoV-2 infection^39–41^.

The expression of TMPRSS2 is organ- and tissue-specific and most abundant in the lung, prostate and colon^42,43^. Immunohistochemistry studies and single cell transcriptomics analyses detected TMPRSS2 in nasal and bronchial epithelial cells as well as kidney, pancreas and stomach^44–47^.

Increasing evidence indicates that, in addition to the respiratory tract, the gastrointestinal tract is also a common site of SARS-CoV-2 infection and pathogenesis, with intestinal epithelium showing high cell surface expression of both ACE2 and TMPRSS2^48^. Consistent with this observation, SARS-CoV-2 infection is detected in human small intestine^49^ with human gut organoids productively infected by SARS-CoV-2^50,51^. Furthermore, intestinal clinical manifestations of SARS-CoV-2 infection are common^52,53^ with symptoms including nausea, vomiting, diarrhoea and abdominal pain^54–56^.

TMPRSS2 is androgen-responsive^57–59^ with previous studies on TMPRSS2 regulation predominantly focusing on (a) prostate cells carrying the TMPRSS2-ERG translocation, a common determinant of prostate cancer^60,61^ and (b) androgen-responsive cell subsets in the lung^62,63^. Data on TMPRSS2 regulation in non-androgen responsive cells is rarer and limited to analysis of TMPRSS2 expression using libraries of chemical compounds^64^. Therefore, despite the central role of TMPRSS2 in respiratory virus infections, how cell surface, non-androgen responsive TMPRSS2 is regulated and its role in SARS-CoV-2 infection remains unclear.

Here we performed CRISPR-Cas9-based genetic screens to identify cellular factors that regulate surface expression of the TMPRSS2 protein. Our unbiased approach identified Elongin B, VHL and the components of the HIF pathway as regulators of TMPRSS2 expression in colonic epithelial cells. We show that stabilisation of the HIF pathway by prolyl hydroxylase (PHD) inhibitors results in downregulation of TMPRSS2 and reduced SARS-CoV-2 infection of target cells.

## Results

### Selection of human cell line with endogenous TMPRSS2 expression for CRISPR screens

**Fig. 1:**
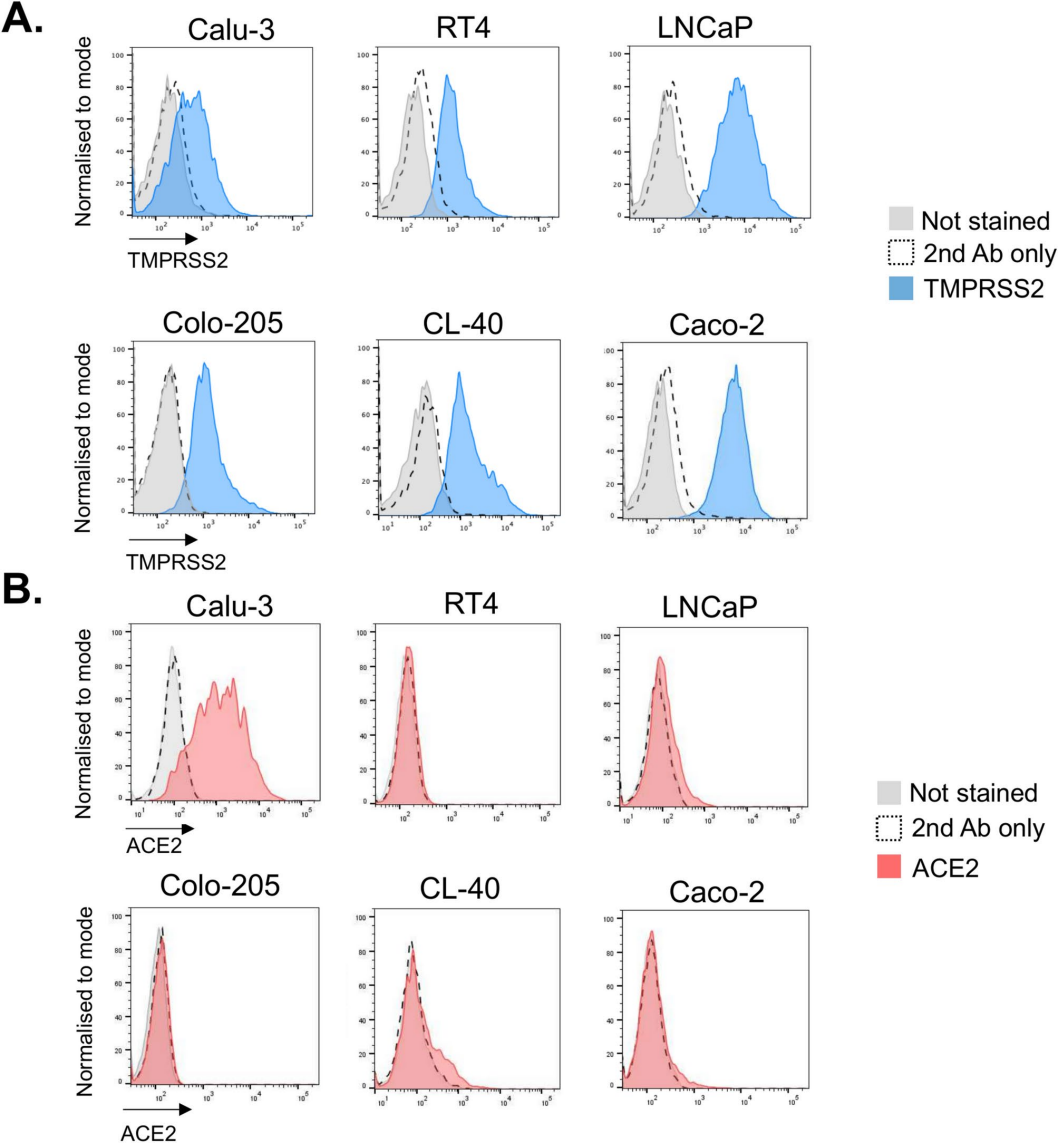
Expression of TMPRSS2 and ACE2 at the surface of human cell lines. (**A**) Calu-3, RT4, LNCaP, Colo-205, CL-40 and Caco-2 cell lines were stained with the TMPRSS2-specific antibody or secondary antibody alone as a control and analysed by flow cytometry. (**B**) Cell lines from the panel 1A were stained with ACE2-specific antibody or secondary antibody alone as a control and analysed by flow cytometry. See also Figure S1.

To select a cell line suitable for CRISPR-Cas9-based genetic screening, we used a TMPRSS2-specific antibody to determine cell surface staining of six human cell lines previously reported to express TMPRSS2 by RT-qPCR or immunoblot analyses^22,42,65,66^. These included lung epithelial cells Calu-3, prostate cancer cell line LNCaP, urinary bladder cell line RT4 and three colon cell lines, Colo-205, CL-40, and Caco-2. Flow cytometry analysis confirmed that all six cell lines express cell surface TMPRSS2, at varying levels (Fig. 1A). Calu-3 showed the least TMPRSS2 expression, RT4, CL-40 and Colo-205 were intermediate, while the highest cell surface expression was seen in LNCaP and Caco-2 cells. To determine the level of ACE2 on the surface of these cells, in addition, we stained them with an ACE2-specific antibody. Flow cytometry analysis showed that RT4 and Colo-205 cells did not express ACE2 on their cell surface, whereas Calu-3 had the highest ACE2 expression (Fig. 1B). LNCaP cells had low levels of surface ACE2, whereas CL-40 and Caco-2 cells showed heterogeneous ACE2 expression with only a small proportion of ACE2-positive cells. Out of the three colon-derived cell lines, Caco-2 cells consistently showed the highest cell surface TMPRSS2 expression—the crucial parameter for effective fluorescence-based genetic screens. Caco-2 cells were also the most tractable for genetic manipulation and we therefore chose to use these cells in the genetic screens. TMPRSS2-specific or control sgRNAs confirmed the specificity of the anti-TMPRSS2 antibody for cell surface staining as flow cytometry of transduced Caco-2 Cas9 cells showed clear loss of cell surface staining in the TMPRSS2 knockout, but not the control b2m KO cells (Supplementary Figure S1A). Complementary immunoblot analysis showed that TMPRSS2-specific sgRNAs abrogated expression of both high and low molecular weight forms of TMPRSS2 in Caco-2 Cas9 cells (Supplementary Figure S1B). Colonic epithelial Caco-2 cells were therefore selected as the most suitable for CRISPR-Cas9-based genetic screens.

### Depletion of TMPRSS2 reduces SARS-CoV-2 entry into Caco-2 cells and Calu-3 cells

**Fig. 2:**
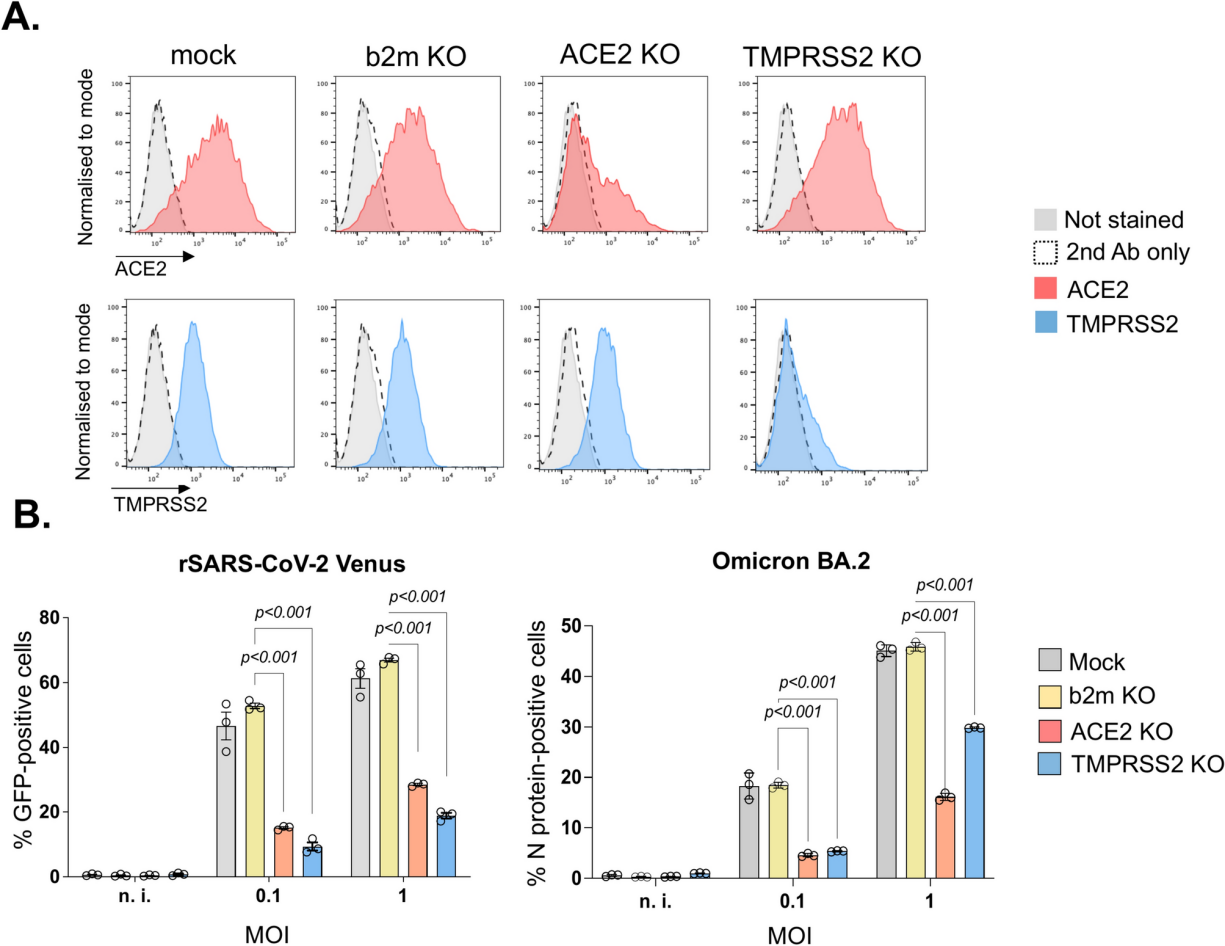
TMPRSS2 KO affects SARS-CoV-2 entry into colon epithelial Caco-2-ACE2 cells. (**A**) Caco-2-ACE2 Cas9 cells stably expressing sgRNAs specific for *b2m*, *ACE2* or *TMPRSS2* were stained with ACE2- and TMPRSS2-specific antibodies or secondary antibody alone and analysed by flow cytometry. (**B**) Caco-2-ACE2 Cas9 cells from the panel 2A were infected with rSARS-CoV-2 Venus or Omicron BA.2 at an MOI of 0.1 or 1, fixed 24 h later and analysed by automated microscopy. Y-axis indicates percentage of GFP (rSARS-CoV-2 Venus) or N-protein (BA.2)-positive cells. Data are presented as mean of *n* = 3 biological replicates ± s.d. The statistical significance was assessed by two-way ANOVA and Bonferroni’s multiple comparison correction. See also Figures S2, S3 and S4.

The colon epithelial cell line Caco-2, along with the lung epithelial cell line Calu-3 have been used extensively for SARS-CoV-2 infection experiments^67,68^. To confirm that TMPRSS2 depletion affects SARS-CoV-2 entry in Caco-2 and Calu-3 cells we infected the respective wildtype and gene-knockout cells. Infectivity was low in Caco-2 cells due to poor ACE2 cell surface expression, as compared with the high endogenous cell surface ACE2 levels in Calu-3 cells^69^ (Supplementary Figure S2 and unpublished observations). Caco-2 cells were therefore transduced with an ACE2-expressing lentiviral vector and this parental cell line (Caco-2-ACE2) together with Calu-3 cells subsequently depleted of either TMPRSS2 or ACE2. Flow cytometry confirmed the reduction in cell surface TMPRSS2 and ACE2 in both cell types (Figs. 2A and S3A) and infection with wild type rSARS-CoV-2-Venus^70,71^ showed reduced viral entry following either ACE2 or TMPRSS2 depletion (Figs. 2B and S3B). We also tested the effect of TMPRSS2 depletion on SARS-CoV-2 Omicron BA.2 cellular entry as this viral variant was reported to show attenuated TMPRSS2 usage^72^. At low viral MOIs, Omicron BA.2 entry into Caco-2-ACE2 was also reduced following TMPRSS2 depletion, though not as effectively at higher viral MOIs, suggesting that Omicron variants also rely on TMPRSS2 for entry into TMPRSS2-positive cells. This effect was confirmed using the protease inhibitor camostat mesilate which completely blocked Caco-2-ACE2 infection with rSARS-CoV-2 Venus and significantly (*p* < 0.001) reduced infection with Omicron variant BA.2 validating the results seen with the genetic depletions (Supplementary Figure S4). Likewise, infection of Caco-2-ACE2 cells with Omicron subvariants XBB.1.1 and XBB.2.3 was also significantly (*p* < 0.001) reduced in the presence of camostat mesilate. Together, our results confirm the central role for TMPRSS2 as an entry factor for wild type SARS-CoV-2 and Omicron variants in infection of transformed colon-derived epithelial Caco-2 cells.

### Genome-wide and targeted CRISPR screens reveal Elongin BC-VHL complex as a regulator of the surface TMPRSS2 expression

**Fig. 3:**
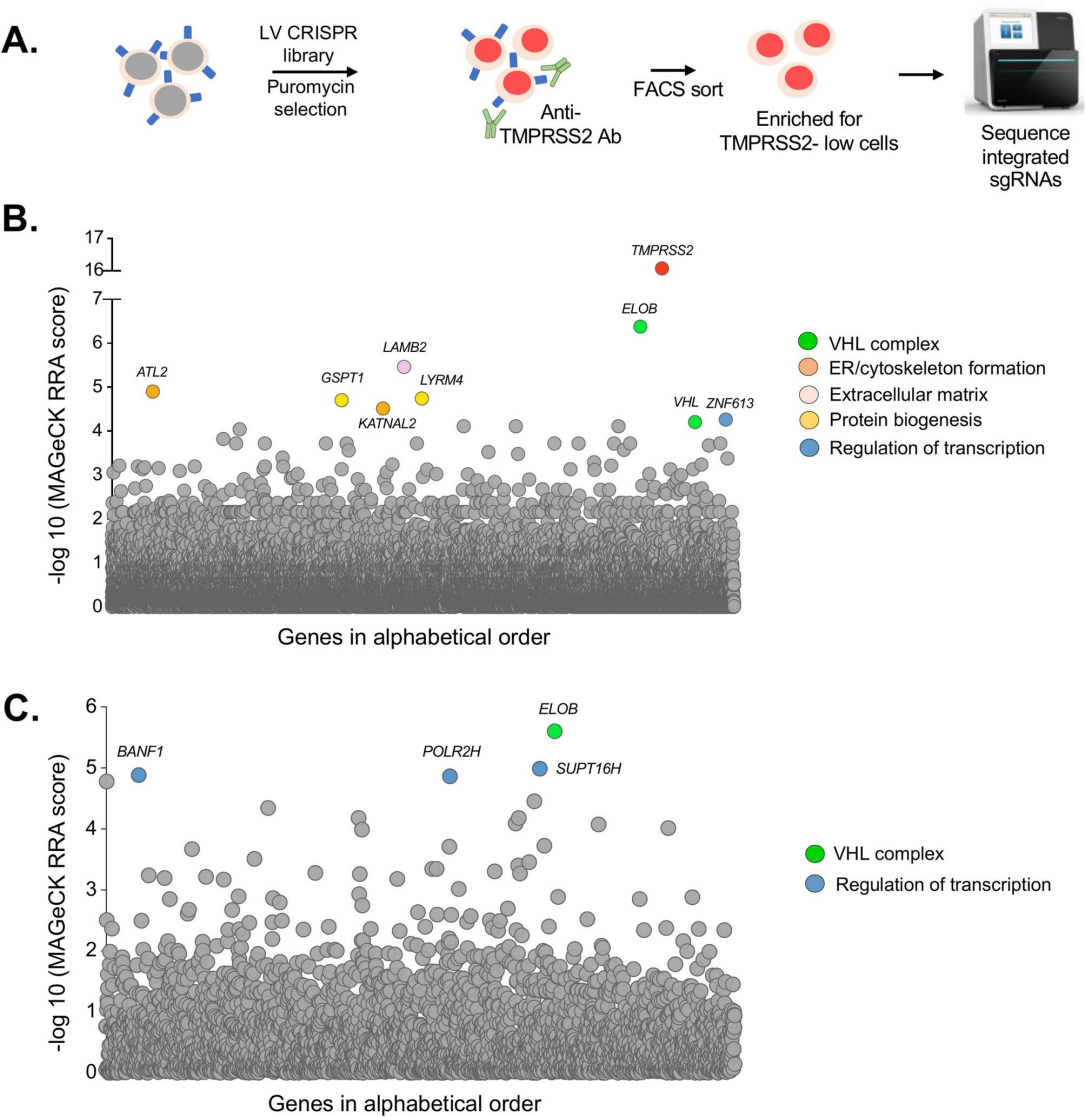
Genome-wide and targeted CRISPR-Cas9 screens reveal *Elongin* B and VHL as regulators of TMPRSS2 expression. (**A**) Schematic workflow of the genome-wide and targeted CRISPR-Cas9 screens. Caco-2 cells were transduced with lentivirus encoding CRISPR library followed by selection with puromycin. The populations of cells with low TMPRSS2 expression were enriched by FACS with the TMPRSS2-specific antibody, subjected to genomic DNA isolation followed by Illumina sequencing of the integrated gRNAs. (**B**, **C**) MAGecK RRA (robust rank aggregation) scores of the enriched sgRNAs from the CRISPR-Cas9 screens performed with the genome-wide sgRNA library (**B**) and sgRNA library targeting Epigenetic Modifiers and Transcriptional Regulators (**C**).

**Fig. 4:**
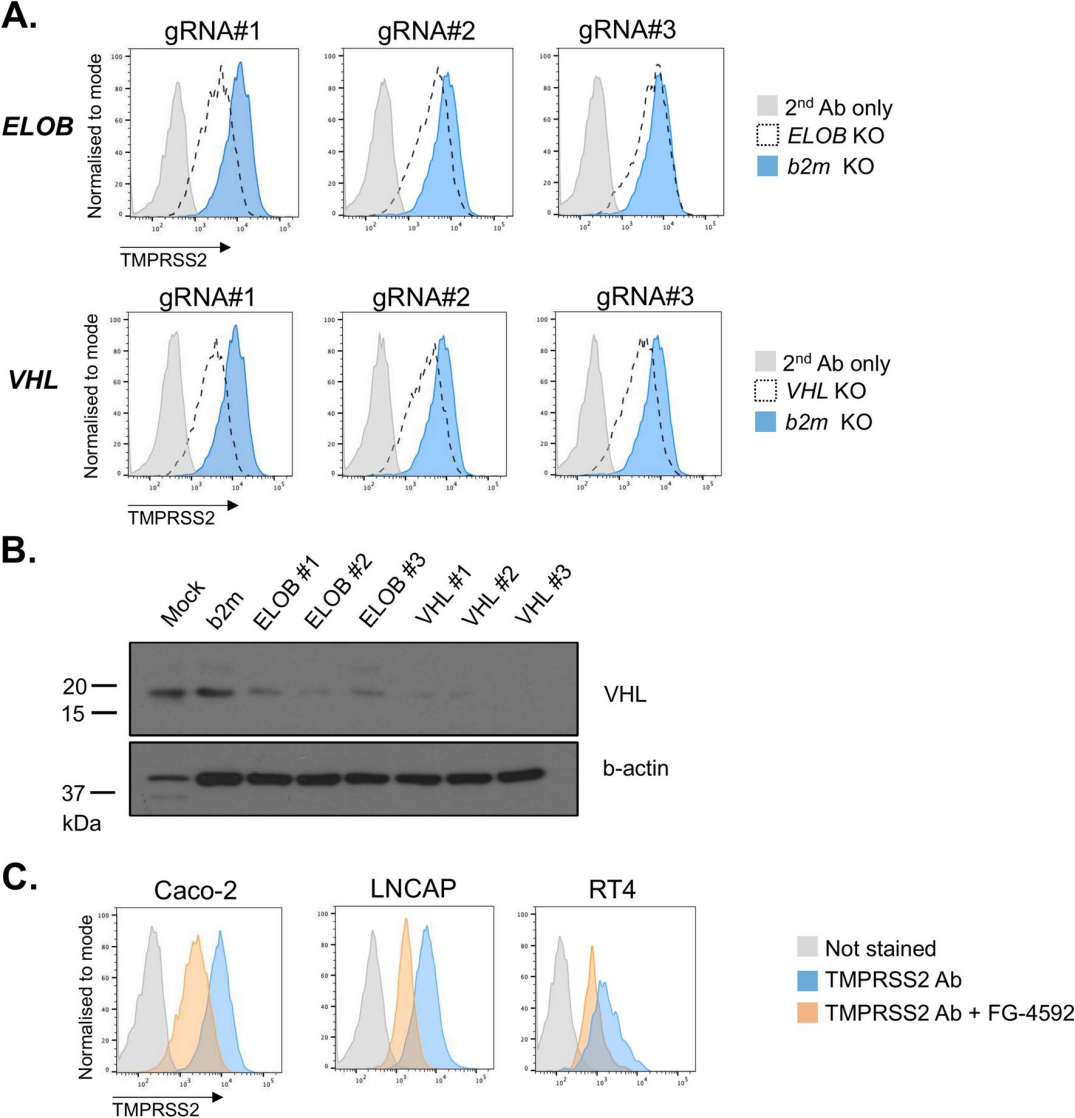
Surface TMPRSS2 is regulated by Elongin B and VHL in Caco-2 cells. (**A**) Caco-2 Cas9 cells stably expressing sgRNAs targeting *ELOB*, *VHL* and *b2m* were stained with the TMPRSS2-specific antibody or secondary antibody alone as a control and analysed by flow cytometry. (**B**) Caco2 Cas9 cells from the panel 4A were lysed and analysed by immunoblot with the antibody specific for VHL and b-actin. (**C**) Caco-2, LNCAP and RT4 cells were treated with FG-4592 (roxadustat), stained with the TMPRSS2-specific antibody and analysed by flow cytometry. See also Figures S5 and S6.

To identify cellular proteins that regulate cell surface TMPRSS2 expression we performed CRISPR-Cas9-based genetic screens in Caco-2 cells. We first performed the screen with the genome-wide CRISPR library^73^. Following lentiviral transduction of the genome-wide CRISPR library, the TMPRSS2-low expressing cells were sorted by FACS and the integrated sgRNAs from this enriched population were decoded by Illumina sequencing (Fig. 3A). The top hit was *TMPRSS2* itself, thus validating the screen (Fig. 3B) with *Elongin B* being the second most enriched gene. *Elongin B* encodes a component of the HIF (hypoxia-induced factors) degradation complex^74–76^, and sgRNAs specific for the von Hippel–Lindau (*VHL*) gene, that encodes the VHL E3 ligase component of this protein degradation complex were also enriched in the screen. Other significant (MAGeCK robust rank aggregation (RRA) score > 4.2) hits identified included regulators of ER and cytoskeleton formation (eg *ATL2),* extracellular matrix constituent *LAMB2*, regulators of protein biogenesis (eg *GSPT1*) and a putative regulator of transcription *ZNF613* (Table 1). A complementary genetic screen was performed with a sub-genomic CRISPR library targeting Epigenetic Modifiers^77^ combined with a sub-genomic library specific for Transcriptional Regulators (EMTR library). Consistent with the results of the initial genome-wide screen, sgRNAs specific for *Elongin B* were most significantly (MAGeCK RRA score > 4.8) enriched (Fig. 3C). Other significant hits included (i) *POLR2H,* that encodes a conserved subunit shared by RNA polymerases I, II and III, (ii) *SUPT16H,* that encodes a subunit of the FACT (FAcilitates Chromatin Transcription) complex, a critical regulator of transcription^78,79^ and (iii) *BANF1*, that encodes a Barrier-to-Autointegration Factor 1 (BAF/BANF1), a multifunctional protein which, along with other functions, regulates gene expression^80^. We next set out to validate the *Elongin B* and *VHL* hits through their CRISPR-Cas9-mediated depletion and subsequent flow cytometry analysis of TMPRSS2 expression in the knockout cells. Three independent sgRNAs for *Elongin B* and *VHL* each showed a marked downregulation of cell surface TMPRSS2 (Fig. 4A) with efficient depletion of *VHL* confirmed by immunoblot analysis (Fig. 4B). As Elongin B stabilises VHL^81^, depletion of Elongin B leads to a marked reduction in VHL expression (Fig. 4B). Together our results show that the Elongin BC-VHL complex regulates expression of surface TMPRSS2 in Caco-2 cells.

**Table 1:** Summary of hits identified in the genetic screens with genome-wide and EMTR CRISPR libraries.

Gene	Protein	MAGeCK RRA score (-log10)
*Genome-wide CRISPR screen*		
*TMPRSS2*	TMPRSS2	16.0
*ELOB*	Elongin B	6.3
*LAMB2*	Laminin subunit beta 2	5.4
*ATL2*	Atlastin GTPase 2	4.9
*LYRM4*	LYR Motif-containing protein 4	4.7
*GSPT1*	Eukaryotic Release Factor 3a	4.7
*KATNAL2*	Katanin Catalytic Subunit A1 Like 2	4.5
*ZNF613*	Zinc Finger protein 613	4.2
*VHL*	Von Hippel-Lindau tumor suppressor	4.2
*EMTR library CRISPR screen*		
*ELOB*	Elongin B	5.6
*BANF1*	Barrier-to-Autointegration Factor 1 (BAF/BANF1)	4.8
*POLR2H*	RNA polymerase I, II and III subunit H	4.8
*SUPT16H*	FACT Complex subunit SPT16	4.9

### TMPRSS2 is regulated by the HIF pathway in Caco-2 cells

As the Elongin BC-VHL complex regulates proteolytic turnover of HIF proteins^74–76^, it was important to determine whether TMPRSS2 is itself HIF-dependent. The prolyl hydroxylase (PHD) inhibitor FG-4592 (roxadustat) stabilises HIF subunits and treatment of Caco-2, LNCaP and RT4 cells with roxadustat led to a marked downregulation of cell surface TMPRSS2 (Fig. 4C). RT-qPCR analysis also showed that *TMPRSS2* gene expression was reduced in Caco-2 cells following 24-h treatment with either the daprodustat or roxadustat PHD inhibitor (Supplementary Figure S5), with HIF stabilisation confirmed by upregulation of the *PGK1* and *VEGFA* positive control genes. These findings were extended to analysis of primary intestinal organoids which were isolated and expanded from biopsy samples of human gut tissue. Following roxadustat treatment, *TMPRSS2* expression was again downregulated while the positive control HIF-responsive gene *PGK1* was upregulated (Supplementary Figure S6). Together these data show that TMPRSS2 is regulated by the HIF pathway in both colon epithelial cell lines and primary human intestinal cells.

### A complementary CRISPR-Cas9 screen confirms the role of HIF subunits as regulators of surface TMPRSS2 expression

**Fig. 5:**
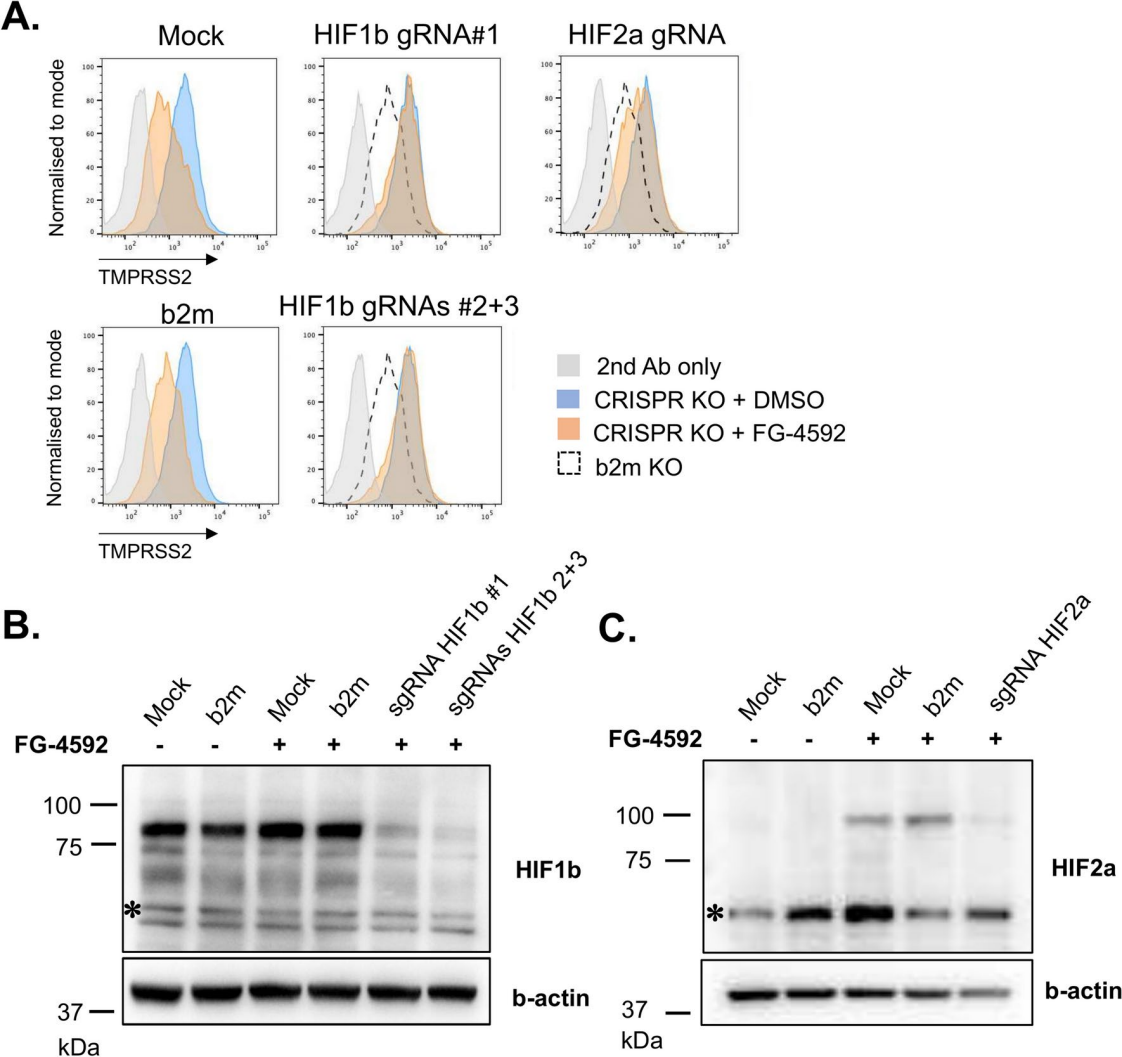
Surface TMPRSS2 is downregulated in a HIF-dependent manner. (**A**) Caco-2 Cas9 cells stably expressing sgRNAs targeting *HIF1b* (*ARNT*), *HIF2a* (*EPAS1*) and *b2m* were treated with FG-4592 (roxadustat) for 7 days, harvested and stained with the TMPRSS2-specific antibody or secondary antibody alone as a control and analysed by flow cytometry. (**B**, **C**) Immunoblot analysis of Caco2 Cas9 cells from the panel 5A. The cells were lysed and analysed by immunoblot with antibodies specific for HIF1b, HIF2a and b-actin. Asterisks denote non-specific bands. See also Figure S7.

Our previous data show that treatment of Caco-2 cells with PHD inhibitors resulted in TMPRSS2 downregulation, but it was unclear whether this effect was mediated by the HIF pathway directly (i.e. through binding of HIFs to the TMPRSS2 promoter) or indirectly as may occur through recruitment of a potential repressor. To try and identify proteins responsible for this HIF-dependent TMPRSS2 downregulation, we performed a complementary genetic screen in Caco-2 cells again using the CRISPR EMTR library (Supplementary Figure S7A). However, in this screen, we enriched for cells unable to downregulate cell surface TMPRSS2 in the presence of roxadustat. Two components of the HIF pathway, HIF1b (*ARNT*) and HIF2a *(EPAS1)*, were both dominant hits from this screen (Supplementary Figure S7B), confirming the central role of HIFs in TMPRSS2 regulation. Other hits included actin-related protein 6 (*ACTR6*), subunits of the integrator complex (eg *INTS6* and *INTS8*), cleavage and polyadenylation factor *CFIm25*, arginine methyltransferase *PRMT7*, all transcriptional regulators; ATP-dependent RNA helicase *DDX51* and components of E3 ubiquitin protein ligase complexes (eg *FBXO10*). CRISPR-Cas9-based knockouts of these hits confirmed that depletion of HIF1b completely rescued roxadustat-induced TMPRSS2 downregulation, whereas depletion of HIF2a, CFIm25, PRMT7, DDX51, FBXO10 and integrator complex subunits also resulted in a partial rescue of TMPRSS2 downregulation (Fig. 5A and Supplementary Figures S7C and S7D). Efficient depletion of HIF1b and HIF2a from roxadustat-treated Caco-2 cells was confirmed by immunoblot (Fig. 5B and C). Although the targeted CRISPR screen failed to identify putative repressor proteins that might be recruited by HIFs for TMPRSS2 downregulation, it clearly substantiated the role of HIF subunits in the observed phenotype. Our data therefore confirm that surface TMPRSS2 is regulated by Elongin BC-VHL complex in Caco-2 cells in a HIF-dependent manner.

### CRISPR Cas9-mediated depletion of Elongin B or PHD inhibitor treatment decrease SARS-CoV-2 infection of Calu-3 cells

**Fig. 6:**
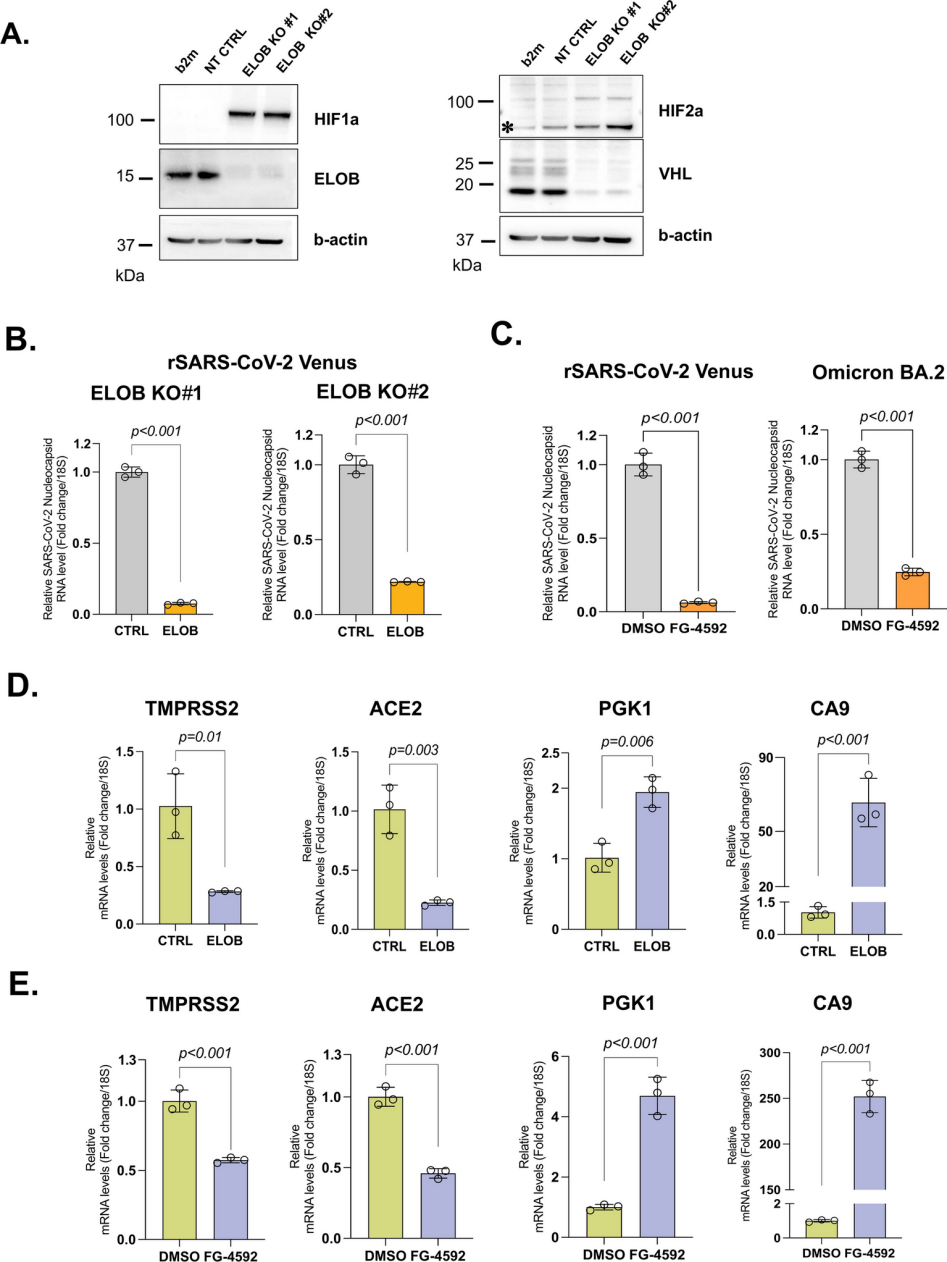
CRISPR Cas9-mediated depletion of Elongin B or PHD inhibitor treatment decrease SARS-CoV-2 infection of Calu-3 cells. (**A**) Calu-3 Cas9 cells stably expressing sgRNAs targeting *b2m*, *Elongin B* (*TCEB2*) or control sgRNAs were lysed and analysed by immunoblot with antibodies specific for HIF1⍺, HIF2⍺, Elongin B, VHL and b-actin. Asterisks denote non-specific bands. (**B**) Calu-3 Cas9 cells stably expressing two independent pairs of sgRNAs targeting *Elongin B* (*TCEB2*) or control sgRNAs were infected with rSARS-CoV-2 Venus at an MOI of 0.1, harvested 24 h later and subjected to RNA extraction followed by RT-qPCR analysis with the primers specific for SARS-CoV-2 nucleocapsid RNA and *18S*. Data are presented as mean of *n* = 3 technical replicates ± s.d. The statistical significance was assessed by unpaired two-tailed *t* test. (**C**) Calu-3 cells were treated with 100 uM FG-4592 (roxadustat) or DMSO as a control for 72 h, infected with rSARS-CoV-2 Venus or Omicron BA.2 at an MOI of 0.1, harvested 24 h later and subjected to RNA extraction followed by RT-qPCR analysis with the primers specific for SARS-CoV-2 nucleocapsid RNA and *18S*. Data are presented as mean of *n* = 3 technical replicates ± s.d. The statistical significance was assessed by unpaired two-tailed *t* test. (**D**) Depletion of Elongin B induces downregulation of *ACE2* and *TMPRSS2* in Calu-3 Cas9 cells. The cells from the panel 6A (right) were subjected to RNA isolation followed by RT-qPCR analysis with primers specific for *TMPRSS2*, *ACE2*, *PGK1*, *CA9* and *18S*. Data are presented as mean of *n* = 3 technical replicates ± s.d. The statistical significance was assessed by unpaired two-tailed *t* test. (**E**) PHD inhibitor treatment induces downregulation of *ACE2* and *TMPRSS2* in Calu-3 cells. The cells were treated with 100 uM FG-4592 (roxadustat) for 72 h and subjected to RT-qPCR analysis as described above (6D).

Depletion of Elongin B and VHL, as well as treatment of Caco-2 cells with PHD inhibitors led to the HIF-dependent downregulation of TMPRSS2. It was therefore important to determine whether stabilisation of HIF proteins influences SARS-CoV-2 infection. To this end, we generated CRISPR Cas9-mediated knockouts of Elongin B in a clonal population of Calu-3 cells expressing endogenous TMPRSS2 and ACE2^69^ (Supplementary Figure S3). Efficient knockout of Elongin B in two independent cell populations with dual sgRNAs was confirmed by immunoblot (Fig. 6A). Depletion of Elongin B also resulted in marked reduction of VHL and stabilisation of HIFa proteins. We then infected the resulting Calu-3 Elongin B knockout (KO) cells with the rSARS-CoV-2 Venus virus and analysed the infection by RT-qPCR. Our results showed that virus infection of Elongin B KO cells was inhibited compared to the control cells (Fig. 6B). To substantiate this result, we treated Calu-3 cells with PHD inhibitor FG-4592 followed by infection with rSARS-CoV-2 Venus or Omicron BA.2 viruses. RT-qPCR analysis showed that FG-4592 treatment reduced the infection of Calu-3 cells with both viruses, compared to the control cells (Fig. 6C). Taken together, these results demonstrate that stabilisation of HIF proteins inhibits SARS-CoV-2 infection of Calu-3 cells. Next, to determine whether depletion of Elongin B affects expression of *TMPRSS2* in cells other than Caco-2 we showed that Calu-3 Elongin B KO cells also downregulated *TMPRSS2* gene expression (Fig. 6D). Similarly, Calu-3 cells downregulated *TMPRSS2* gene expression after treatment with FG-4592 (Fig. 6E). Importantly, in addition to *TMPRSS2, ACE2* was also downregulated in Calu-3 Elongin B KO and FG-4592-treated cells, while control HIF-responsive genes *PGK1* and *CA9* were upregulated. Therefore, in Calu-3 cells, HIF stabilisation induces downregulation of both ACE2 and TMPRSS2, resulting in decreased SARS-CoV-2 infection.

## Discussion

For entry into target cells, SARS-CoV-2 relies on two key cellular proteins: ACE2, which serves as a receptor for virion attachment, and TMPRSS2, a surface protease that mediates cleavage of the viral spike protein thereby enabling fusion of the virion with the plasma membrane. Our study has shown that the TMPRSS2 protease is regulated by the Elongin BC-VHL complex and HIF proteins. Activation of the HIF pathway through either genetic depletion of Elongin B and VHL, or treatment of cells with PHD inhibitors resulted in downregulation of cell surface TMPRSS2. Furthermore, activation of the HIF pathway reduced SARS-CoV-2 infection of lung epithelial cells.

Several recent studies have reported that the Omicron lineage of SARS-CoV-2 has a decreased reliance on TMPRSS2 for cell entry and preferentially enters cells via a TMPRSS2-independent endosomal route^25–27^. In addition, Omicron entry into primary nasal epithelial cells has been reported to occur through a divergent, TMPRSS2-independent, non-endosomal route that relies upon metalloproteinases^28^. Our results show that entry of Omicron BA.2 variant into colon epithelial cells remains sensitive to TMPRSS2. Furthermore, cell entry of the Omicron subvariants XBB.1.1 and XBB.2.3 was also inhibited by the protease inhibitor camostat mesilate. Our data are therefore consistent with recent observations that: (i) SARS-CoV-2 Omicron variants remain TTSP dependent for entry into human primary airway organoids^82^ and colon epithelial cells^83^; (ii) Omicron subvariant BA.2.86 uses TMPRSS2 for entry into lung epithelial cells^84^. Collectively, these data imply that protease inhibitors are likely to remain effective against new SARS-CoV-2 variants. Our data also substantiate the results of the recent in vivo studies that demonstrate an essential role of TMPRSS2 for Omicron infection in mice^18,29^.

CRISPR-Cas9-based screening approaches have been widely used for identification of cellular genes and pathways required for SARS-CoV-2 infection (reviewed in^85,86^). These include loss-of-function and gain-of-function screens for cellular factors that mediate virus entry^87–92^, dropout screens for pro-viral and anti-viral cellular factors^93–95^ and an antibody-based CRISPR screen for regulators of ACE2 expression^96^. With only a few exceptions, those screens relied on engineering of cell lines to express exogenous ACE2 and TMPRSS2. This approach, therefore, will not identify regulators of endogenous gene expression. Furthermore, in many instances the target cell lines for CRISPR screens did not express endogenous TMPRSS2 at the cell surface and were modified with ACE2 alone, therefore skewing the results of the screens towards TMPRSS2-independent endosomal route of SARS-CoV-2 entry. In comparison to previous studies, our CRISPR screens specifically focus on regulation of the SARS-CoV-2 co-receptor, endogenous surface TMPRSS2 protein, in colon epithelial cells, a cell type susceptible to SARS-CoV-2 infection.

**Fig. 7:**
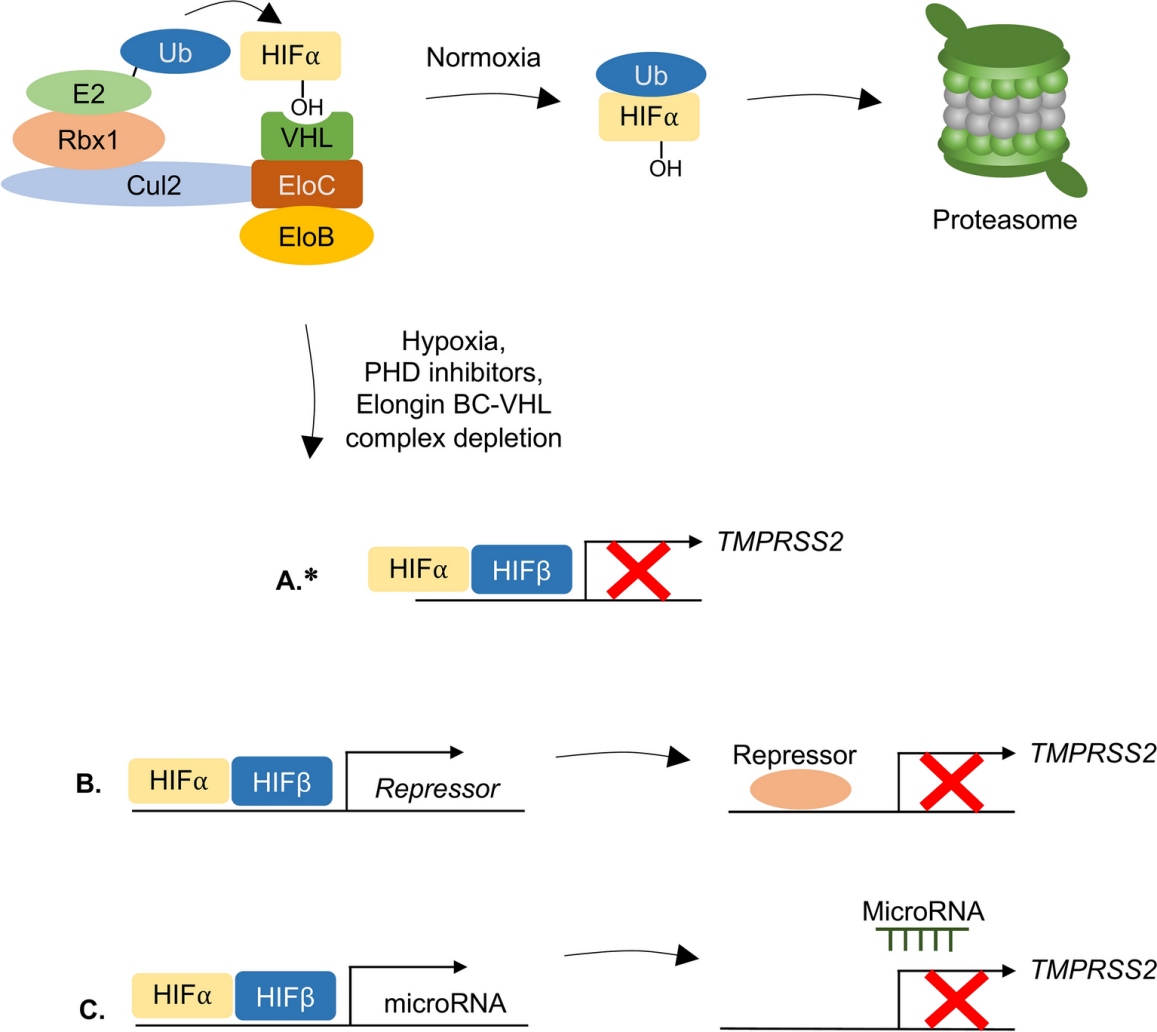
Proposed mechanisms of TMPRSS2 regulation by the Elongin BC-VHL complex. Under normal physiological conditions, constitutively expressed HIF⍺ proteins are hydroxylated by prolyl hydroxylase domain (PHD) enzymes, recognised by the Elongin BC-VHL complex, ubiquitinated and targeted for proteasomal degradation. Under conditions of hypoxia, treatment with PHD inhibitors or depletion of the Elongin BC-VHL complex, the stabilised HIF⍺ protein heterodimerises with HIFβ and inhibits expression of TMPRSS2 either (**A**) through direct binding to the promoter or indirectly through induction of expression of a putative protein repressor (**B**) or microRNA (**C**). *It should be noted that since no hypoxia response elements were identified in the *TMPRSS2* promoter, the regulation of TMPRSS2 expression will likely occur as described in scenarios (**B**) or (**C**).

Two CRISPR-Cas9-based genetic screens revealed Elongin B as a key regulator of TMPRSS2 expression. Together with VHL and Elongin C, Elongin B is part of the complex that mediates HIF degradation under normoxia^74–76^. Finding that surface TMPRSS2 is downregulated by the HIF signaling pathway was therefore intriguing. It implies that HIF activation causes critical gene repression. While the role of HIFs in gene activation is well established, much less is known about HIF-mediated gene repression^97,98^. HIF-mediated gene repression is versatile, and occurs either directly through DNA binding^99^, indirectly through engagement of repressor complexes^100^ or through RNA interference^101^ (Fig. 7). We were unable to identify the short core 5′-(A/G)CGTG-3′ motif in the *TMPRSS2* promoter commonly found in hypoxia response elements^102^, prompting us to perform a complementary CRISPR screen for cellular factors required for HIF-induced TMPRSS2 downregulation. A subsequent analysis of our validated hits (e.g. cleavage and polyadenylation factor CFIm25 and subunits of the Integrator complex) suggested that they likely act upstream of HIFs or regulate transcription independently of the HIF pathway. No hits with a putative repressor function (that could be recruited by HIFs to inhibit TMPRSS2 expression) were identified. Therefore, how HIF mediates TMPRSS2 downregulation remains unclear. Interestingly, hypoxia-mediated inhibition of the main SARS-CoV-2 co-receptor, ACE2 is mediated by specific miRNA- silencing^103^ and it will be interesting to learn whether TMPRSS2 relies on a similar mechanism.

Neither Elongin B nor VHL were among the top-ranking hits (*z*-score > 3, MAGeCK RRA score > 4) in the published genome-wide CRISPR knockout screens for regulators of SARS-CoV-2 infection conducted in cells expressing endogenous TMPRSS2, namely Calu-3 and Caco-2-ACE2^87,88^. The explanation for this discrepancy likely lies in the nature of the screens performed: (i) in CRISPR knockout screens with SARS-CoV-2, enriched cells contain sgRNAs that confer full resistance to viral infection, for example, by completely blocking receptor expression on the cell surface; (ii) in contrast, in phenotypic CRISPR screens, the enriched population of cells contains sgRNAs that are responsible only for changes in receptor expression (either substantial or minor) that may influence viral entry but do not necessarily completely block it.

Depletion of Elongin B or treatment of Calu-3 cells with PHD inhibitors decreased expression of both TMPRSS2 and ACE2 and reduced SARS-CoV-2 infection. These results are consistent with studies showing that HIF activation inhibits SARS-CoV-2 infection of lung epithelial cells^104^, while contrasting with a report showing that PHD inhibition with Molidustat, increases SARS-CoV-2 infection of monocytes^105^. This apparent discrepancy may be explained by the cell type-specificity in HIF-mediated regulation of SARS-CoV-2 entry factors and virus infection. Variable hypoxic regulation of TMPRSS2 and ACE2 has been observed even between two subsets of primary murine alveolar epithelial cells^106^. The duration of hypoxia may also affect expression of SARS-CoV-2 entry factors. For instance, in brain endothelial cells, hypoxia induces dynamic changes in ACE2 and TMPRSS2 expression, with an acute increase and subsequent decrease of mRNA levels^107^. Further studies will be required to clarify this differential regulation of SARS-CoV-2 entry factors.

HIF pathway-dependent regulation of both TMPRSS2 and ACE2 in Calu-3 cells is a confounding factor in this study as we are unable to attribute the Elongin B knockout-mediated reduction in SARS-CoV-2 infection (or the antiviral activity of the PHD inhibitor) to one or the other entry factor. This problem could potentially be addressed by performing infection experiments with ACE2 overexpressed under an exogenous, hypoxia-insensitive promoter. However, the exogenous promoters we have tested to date (pCMV, pSFFV, pEF1a, pEF4a) have all shown sensitivity to PHD inhibitor treatment (data not shown) and therefore cannot be used in such an experiment. Thus, it remains unclear to what extent Elongin BC-VHL complex-mediated regulation of TMPRSS2 affects SARS-CoV-2 infection independently of ACE2.

In summary, we have applied unbiased CRISPR Cas9 genetic screening approaches to identify cellular genes that regulate surface expression of TMPRSS2 protein. Our findings show how surface TMPRSS2 is regulated by the Elongin BC-VHL complex and HIF transcription factors. Stabilisation of HIFs mediated by depletion Elongin B or treatment with PHD inhibitor compound resulted in inhibition of SARS-CoV-2 infection. Our study provides insight into the regulation of TMPRSS2 and underlines its important role in the cellular entry of SARS-CoV-2.

## Materials and methods

### Lead contact

Further information and requests for resources and reagents should be directed to and will be fulfilled by the lead contact, Ildar Gabaev (gabaev.ildar@outlook.com).

### Materials availability

All unique reagents generated in this study are available upon request.

### Cell lines

HEK293T cells (obtained from ATCC) were grown in Iscove’s Modified Dulbecco’s Medium (Sigma), human colon adenocarcinoma Caco-2 cells (obtained from Abcam) were grown DMEM (Sigma), supplemented with 1 mM Sodium pyruvate (Gibco), human lung carcinoma Calu-3 cells (a kind gift of Stefan Pöhlmann, German Primate Centre, Göttingen) were grown in MEM (Sigma) supplemented with sodium pyruvate (1 mM, Gibco) and non-essential aminoacids supplement (1 mM, Gibco). Prostate carcinoma LNCaP cells (a kind gift of Frank McCaughan) were grown in RPMI-1640 (Cytiva) supplemented with 2.5 g/L glucose and non-essential aminoacids (Gibco). Human colon carcinoma Colo-205 and CL-40 cells, as well as human urinary bladder RT4 cells were a kind gift of Jason Carroll and Jill Temple (Cancer Research UK Cambridge Institute). Colo-205 cells were grown in RPMI-1640 (Sigma), CL-40 cells were grown in DMEM (Sigma) and Ham’s F12 media (Sigma) (1:1 mix) supplemented with 20% fetal calf serum (Gibco), RT4 cells were grown in McCoy’s 5A Medium (Lonza). Vero AT2 cells (a kind gift of E. Thomson) were grown in DMEM (Sigma) supplemented with hygromycin (50 ug/ml) and neomycin (50 ug/ml). Unless stated otherwise, all cell lines were grown at 37 °C and 5% CO_2_, all media were supplemented 10% fetal calf serum (Gibco), penicillin (100 units/ml), streptomycin sulphate (100ug/ml), and 2 mM Glutamax (Gibco).

### Plasmid construction

To generate pHRSIN-pEIF4a-hACE2-PGK-Hygro vector, DNA fragments containing hACE2 cDNA^108^, EIF4a promoter sequence and hygromycin resistance cassette driven by a pGK promoter (amplified from the pHRSIN-pEIF4a-GFP and pHRSIN-pSFFV-pGK-Hygro vectors, respectively; both vectors are from the Lehner Lab plasmid collection) were purified and cloned into NheI-treated pHRSIN-cSGW vector backbone using NEBuilder HiFi DNA Assembly master Mix (NEB, Cat. No. E2611L). For sgRNA cloning, sense and antisense oligonucleotides (Merck) were phosphorylated using T4 PNK (NEB) at 37 °C for 30 min, annealed at 95 °C for 5 min and cooled to room temperature. The resulting annealed fragments were treated with BpiI endonuclease (Thermo Fischer Scientific) and cloned into BbsI-treated pKLV-U6gRNA(BbsI)-PGKpuro2ABFP or SapI/BbsI-treated pKLV2.2-h7SKgRNA5(SapI)-hU6gRNA5(BbsI)-PGKpuroBFP-W vectors (Addgene plasmids #50946 and #72666, kindly deposited by K. Yusa) using T7 ligase (NEB). The resulting reactions were transformed in NEB 5-alpha competent *E. coli* (NEB, Cat. No. C2987) and selected on agar plates with ampicillin. Plasmid DNA was isolated using QIAprep Spin Miniprep Kit (Qiagen) and validated by Sanger sequencing.

### Lentivirus production and transduction

HEK293 cells were transfected with a lentivirus expression vector and the packaging vectors pCMVΔR8.91 and pMD.G using TransIT-293 transfection reagent (Mirus, Cat. No. MIR2704) according to manufacturer’s recommendations. Supernatants were harvested 48 h post transfection and passed through 0.45 um filter units. Typically, cells were transduced at an MOI < 1 in 6 well plates by centrifugation at 800 g for 1 h. The following drug concentrations were used for selection of transduced cells: puromycin (4 ug/ml), blasticidin (3 ug/mL) or hygromycin (50 ug/ml).

### CRISPR-Cas9- mediated gene knockouts

For CRISPR-Cas9-mediated gene disruption, cells were first transduced with pHRSIN-pSFFV-Cas9-pPGK-Blasticidin lentivirus vector^109^ followed by blasticidin selection. The efficiency of Cas9 was assessed by analysis of the surface MHC class I expression upon transduction of the cells with lentivirus vector harbouring sgRNAs specific for b2m as described previously^110^. sgRNA sequences were selected from the published sgRNA libraries^73,111–113^. Sequences of sgRNAs specific for HIF1b and HIF2a were described previously^114^. All the sgRNA sequences are listed in the Supplementary table 2. The cells were transduced with lentivirus expressing sgRNA, selected by puromycin 48 h later and analysed for gene disruption by flow cytometry or immunoblotting using gene-specific antibody at least 5 days post transduction.

### Genetic screens with genome-wide and Epigenetic modifiers and transcriptional regulators CRISPR sgRNA libraries

Human Improved Genome-wide Knockout CRISPR Library v1 was a gift from Kosuke Yusa (Addgene #67989)^73^. To generate CRISPR library targeting Epigenetic Modifiers and Transcriptional Regulators (EMTR library/15000 sgRNAs), the CRISPR sub-library (7123 sgRNAs) targeting Epigenetic Modifiers (EM)^77^ was complemented with the CRISPR sub-library (7874 sgRNAs) targeting Transcriptional Regulators (TR) as well as 340 non-targeting sgRNAs^110^. To design the complementary “TR” CRISPR sub-library, we first assembled a candidate gene list by integrating (a) transcription factors with known or postulated DNA binding activity from the Human Transcription Factor database^115^ and (b) known transcriptional regulators from multiple source lists^116–118^. Candidates appearing on multiple source lists were immediately accepted. Further candidate genes were manually reviewed for inclusion or omission (if already present in the ‘EM” CRISPR sub-library) resulting in a total number of 1575 genes in the sub-library. For each gene in the candidate gene list, where possible, we selected five unique sgRNAs from the previously reported high-performance CRISPR libraries^119–121^ using custom scripts. The comprehensive list of sgRNA sequences from the EMTR library is provided in the Supplementary Table 1. Cloning of the “TR” CRISPR sub-library was performed as described previously^77^. Single cell clones of Caco-2 Cas9 cell population were obtained by FACS based on the surface staining with TMPRSS2-specific antibody. Lentiviruses encoding CRSIPR libraries were titrated by assessment of the number of BFP + cells 72 h after transduction. For genome-wide CRISPR-Cas9 screen, a total of 1 × 10^8^ Caco-2 Cas9 cells were transduced with a library containing 90700 sgRNAs^73^ at an MOI of 0.3 (~ 330-fold coverage) followed by puromycin selection 48 h post transduction. The cells were stained with TMPRSS2-specific antibody at day 9 post transduction and the top 1% (~ 2.6 × 10*5 cells) of TMPRSS2-low cell population was selected by FACS. The sorted TMPRSS2-low cell population was expanded, stained with TMPRSS2 antibody and enriched by additional sort 28 days post transduction. For targeted CRISPR-Cas9 screens, a total of 2.4 × 10^7^ Caco-2 Cas9 cells were transduced with lentivirus harbouring EMTR library at an MOI of 0.3 (~ 480-fold coverage) and selected on puromycin 48 h later. For the targeted CRISPR-Cas9 screen on cellular factors that rescue PHD inhibitor-induced TMPRSS2 downregulation, the cells were additionally treated with 100 uM roxadustat for 120 h starting at day 7 post transduction. The cells were then cell surface stained with the TMPRSS2-specific antibody and sorted by FACS. The top 2% (~ 1 × 10*5 cells) of TMPRSS2-low (Fig. 3C) or TMPRSS2-high (Figures S7A and S7B) cell populations were selected for subsequent DNA isolation. DNA from the parental (unsorted) Caco-2 Cas9 cells harbouring stably integrated CRISPR libraries was extracted using Puregene Core kit A (Qiagen, Cat. No. 158489). DNA from the sorted TMPRSS2-low or TMPRSS2-high cell populations was extracted using Quick-gDNA MicroPrep Kit (Zymo research, Cat. No. D3020). Integrated sgRNA sequences were amplified by two sequential rounds of amplification with introducing Illumina adaptors in the second round of PCR, followed by sequencing using the Illumina MiniSeq and NovaSeq platforms. Sequence analysis was performed using a Snakemake workflow v7.32.4^122^ with the following step: the first 19 base pairs of each read were kept using CUTADAPT v3.7^123^. The resulting sequences were aligned against the relevant sgRNA library using HISAT2 (version 2.2.1) while allowing no mismatches. Mapping rates were > 85% for each sample. Statistical analysis of the enriched sgRNAs in the sorted vs unsorted cell populations was performed using MAGeCK algorithm^124^. The Snakemake workflow is publicly available (DOI 10.5281/zenodo.10286661).

### Flow cytometry analysis

For cell surface staining, cells were dissociated with Accutase Cell Detachment Solution (Biolegend, Cat. No. 423201), washed twice with blocking buffer (4% fetal calf serum in PBS) and incubated with primary antibody for 30 min. The cells were then washed twice with blocking buffer followed by incubation with secondary antibody for 30 min. Cell viability was assessed using DAPI (Cell Signaling, Cat. No. 4083S) or Zombie green Fixable Viability kit (Biolegend, Cat. No. 423112). Measurements were performed on LSR II Fortessa flow cytometer (Becton Dickinson) and analysed using FlowJo (TreeStar) software. Ten thousand events were counted for each sample. Sorts were performed on an Influx cell sorter (Becton Dickinson). All procedures were performed at 4 °C.

### Immunoblot analysis

The cells were dissociated with Accutase Cell Detachment Solution, washed with PBS twice, lysed in RIPA buffer (150 mM NaCl, 1% Triton X-100, 0.5% Sodium deoxycholate, 0.1% SDS, 50 mM Tris pH 8.0, 1 mM EDTA) and treated with ultrasound (10 cycles, 15 s each). Total protein amount in the samples was measured by BCA assay using Pierce BCA Protein Assay Kit (Thermo Fischer Scientific, Cat. No. 23227) and the amount of cell lysate equivalent to 20 ug of protein per lane was resolved on SDS-page gel. The immunoblot analysis was performed with primary antibody dissolved in PBS with 0.1% Tween-20. Membranes were washed three times in PBS with 0.1% Tween-20 and probed with secondary HRP-linked antibody. Antibody specific for beta-actin was used as loading control. Signals were visualised by chemiluminescence using ECL and ECL Dura Western blotting detection reagents and iBright imaging system (Thermo Fischer Scientific) or SRX-101a medical film processor (Konica Minolta).

### RT-qPCR analysis

Total mRNA from the cells was isolated using RNeasy kit (Qiagen) or Monarch Total RNA Miniprep kit (NEB, cat. No. T2010S) according to manufacturer specifications. 1 ug of purified RNA was taken for cDNA synthesis using Random Hexamer primers (Thermo Fischer Scientific, Cat. No. N8080127) and SuperScript III Reverse Transcriptase (Thermo Fisher Scientific) in 50 ul reaction. The sequences of the primers specific for *ACE2*, *VEGFA and CA9* genes were obtained from Harvard PrimerBank (https://pga.mgh.harvard.edu/primerbank/). Primers specific for *TMPRSS2,* SARS-CoV-2 Nucleocapsid transcript and *18S* ribosomal RNA gene were described previously^125–127^. All the primer sequences are listed in the Supplementary table 3. Transcript levels were determined by quantitative real-time PCR (RT-qPCR) using SYBR Green dye (Applied Biosystems, Cat. No. 4309155) incorporation and 8 ng of cDNA per reaction. The reaction was carried out using MicroAmp Optical 384-Well Reaction Plate (Applied Biosystems, Cat. No. 4309849) and QuantStudio 7 Flex Real time PCR system (Thermo Fisher Scientific). The comparative threshold cycle method was used to determine the change in gene expression between the samples, using 18S for normalisation.

### SARS-CoV-2 infections

All the infections were performed at the CITIID CL3 facility. The SARS-CoV-2 viruses used in this study were rSARS-CoV-2 Venus^70,71^ and clinical isolates of SARS-CoV-2: variants Omicron BA.2, Omicron XBB.1.1 and XBB.2.3. Virus stocks were grown in Vero AT2 cells. The viral titers were determined using plaque assay. For analysis of SARS-CoV-2 infection, the cells were seeded in 96-well PhenoPlate (Perkin Elmer, Cat No. 6055300) at the density of 4 × 10*4 cells per well and infected with SARS-CoV-2 at an MOI of 1 and 0.1. Twenty hours post infection, the cells were fixed with 2% formaldehyde (Sigma-Aldrich, Cat. No F8775), washed with PBS twice, permeabilised with Intracellular Staining Permeabilization Wash Buffer (Biolegend, cat. No. 421002), stained with sheep anti-SARS-CoV-2 nucleoprotein antibody at concentration of 0.78 ug/ml (University of Dundee, Cat. No DA114), washed three times with the same buffer, followed by staining with secondary Alexa-647-conjugated antibody and DAPI. The cells in the wells were then covered with Anti-Fade Fluorescence Mounting Medium (Abcam, Cat. No. 104135) and analysed using ArrayScan XTI automated microscope (Thermo Fisher Scientific). For RT-qPCR analysis of SARS-CoV-2 infection the cells were seeded in 48 well plates, infected with virus at an MOI of 0.1, incubated at 37 °C for 2 h and washed twice with PBS. The cells were further incubated in fresh media for 22 h, harvested and subjected to RNA extraction using RNAeasy Mini kit (Qiagen).

### Human intestinal organoids

Human primary intestinal organoids were derived from terminal ileum biopsies. Primary tissue was obtained from patients undergoing endoscopy following the acquisition of the written informed consent for use in research and ethical approval (Research Ethics Committee (REC) 20/NI/0109) within Addenbrooke’s Hospital on 18.08.2020. All experiments were performed in accordance with relevant guidelines and regulations. The organoids were derived using an adapted version of previously published protocols^126,128–130^. Human intestinal tissue was dissociated into small epithelial fragments, embedded in Matrigel, and cultured in IntestiCult Organoid Growth Medium (StemCell Technologies) supplemented with penicillin–streptomycin and Rho kinase inhibitor (Stratech Scientific). The medium was replaced every 2–3 days and once organoids were established, they were passaged every 7–10 days by mechanical disruption and re-plating in fresh Matrigel.

### Compounds

The following chemical compounds were used for treatment of the cells: Camostat mesilate (APExBIO, Cat. No. B2082), roxadustat (FG-4592, Selleckchem, Cat. No. S1007), GSK1278863 (daprodustat, AOBIOUS, Cat. No. AOB0500).

### Antibodies

Antibodies specific for the following proteins were used for flow cytometry and immunoblotting: rabbit anti-TMPRSS2 (Abcam, Cat. No. EPR24407-87, flow cytometry) rabbit anti-TMPRSS2 (Abcam, Cat. No. EPR3862, immunobloting), rabbit anti-ACE2 (Abcam, Cat. No. EPR24705-45), rabbit-anti-HIF1b/ARNT (Cell Signaling Cat. No. 5537 T), rabbit-anti-HIF2a (Cell Signaling Cat. No. 7096S), rabbit anti-VHL (Cell Signaling Cat. No. 68547S), rabbit anti-TCEB2/Elongin-B (Abcam, Cat. No. EPR10440(B)), Alexa Fluor 647 conjugated goat anti-rabbit (Thermo Fisher Scientific, Cat. No. A21245) and donkey anti-sheep antibody (Thermo Fisher Scientific, Cat. No. A21448), mouse anti-beta Actin (Sigma-Aldrich, Cat. No. A5316), Horseradish Peroxidase (HRP)-conjugated goat-anti-rabbit (Jackson Immunoresearch, Cat. No. 111-035-144), HRP-conjugated goat anti-mouse antibody (Cell signaling, Cat. No. 7076).

### Statistical analysis

Statistical analyses were performed using Prism version 10.1.0 (GraphPad Software). Data were evaluated for normal distribution using Shapiro–Wilk test. A two-tailed unpaired *t* test was used to compare data of two groups. Comparison of more than two groups was done by one-way or two-way ANOVA and Bonferroni’s multiple comparison correction. Statistical parameters are specified in the figure legends. *P* values of less than 0.05 were considered statistically significant. Only significant differences are highlighted in the figures.

## Supplementary Information

Supplementary Information.

Supplementary Information.

Supplementary Information.

## Data Availability

Sequencing data from CRISPR/Cas9 screens presented in this study have been deposited at the Sequence Read Archive (SRA)/PRJNA1101180.

## References

[1] Bugge, T. H., Antalis, T. M. & Wu, Q. Type II transmembrane serine proteases. *J. Biol. Chem.* **284**, 23177–23181 (2009). 19487698 10.1074/jbc.R109.021006 PMC2749090

[2] Forni, D., Sironi, M. & Cagliani, R. Evolutionary history of type II transmembrane serine proteases involved in viral priming. *Hum. Genet.* **141**, 1705–1722 (2022). 35122525 10.1007/s00439-022-02435-y PMC8817155

[3] Paoloni-Giacobino, A., Chen, H., Peitsch, M. C., Rossier, C. & Antonarakis, S. E. Cloning of the TMPRSS2 gene, which encodes a novel serine protease with transmembrane, LDLRA, and SRCR domains and maps to 21q22.3. *Genomics* **44**, 309–320 (1997). 9325052 10.1006/geno.1997.4845

[4] Wettstein, L., Kirchhoff, F. & Münch, J. The transmembrane protease TMPRSS2 as a therapeutic target for COVID-19 treatment. *Int. J. Mol. Sci.* **23**, 1351 (2022). 35163273 10.3390/ijms23031351 PMC8836196

[5] Böttcher, E. et al. Proteolytic activation of influenza viruses by serine proteases TMPRSS2 and HAT from human airway epithelium. *J. Virol.* **80**, 9896–9898 (2006). 16973594 10.1128/JVI.01118-06 PMC1617224

[6] Limburg, H. et al. TMPRSS2 is the major activating protease of influenza A virus in primary human airway cells and influenza B virus in human type II pneumocytes. *J. Virol.* **93**, 10–1128 (2019). 10.1128/JVI.00649-19 PMC6803253 31391268

[7] Chaipan, C. et al. Proteolytic activation of the 1918 influenza virus hemagglutinin. *J. Virol.* **83**, 3200–3211 (2009). 19158246 10.1128/JVI.02205-08 PMC2655587

[8] Abe, M. et al. TMPRSS2 is an activating protease for respiratory parainfluenza viruses. *J. Virol.* **87**, 11930 (2013). 23966399 10.1128/JVI.01490-13 PMC3807344

[9] Shirogane, Y. et al. Efficient multiplication of human metapneumovirus in Vero cells expressing the transmembrane serine protease TMPRSS2. *J. Virol.* **82**, 8942–8946 (2008). 18562527 10.1128/JVI.00676-08 PMC2519639

[10] Shirato, K., Kawase, M. & Matsuyama, S. Middle East respiratory syndrome coronavirus infection mediated by the transmembrane serine protease TMPRSS2. *J. Virol.* **87**, 12552 (2013). 24027332 10.1128/JVI.01890-13 PMC3838146

[11] Glowacka, I. et al. Evidence that TMPRSS2 activates the severe acute respiratory syndrome coronavirus spike protein for membrane fusion and reduces viral control by the humoral immune response. *J. Virol.* **85**, 4122–4134 (2011). 21325420 10.1128/JVI.02232-10 PMC3126222

[12] Hoffmann, M. et al. SARS-CoV-2 cell entry depends on ACE2 and TMPRSS2 and is blocked by a clinically proven protease inhibitor. *Cell* **181**, 271 (2020). 32142651 10.1016/j.cell.2020.02.052 PMC7102627

[13] Saunders, N. et al. TMPRSS2 is a functional receptor for human coronavirus HKU1. *Nature* **624**, 207–214 (2023). 37879362 10.1038/s41586-023-06761-7 PMC11331971

[14] Xia, L., Zhang, Y. & Zhou, Q. Structural basis for the recognition of HCoV-HKU1 by human TMPRSS2. *Cell Res.* **2024**, 1–4. 10.1038/s41422-024-00958-9 (2024). 10.1038/s41422-024-00958-9 PMC11217304 38641728

[15] McCallum, M. et al. Human coronavirus HKU1 recognition of the TMPRSS2 host receptor. *bioRxiv Prepr. Serv. Biol.*10.1101/2024.01.09.574565 (2024). 10.1016/j.cell.2024.06.006 PMC12854727 38964328

[16] Wang, H. et al. TMPRSS2 and glycan receptors synergistically facilitate coronavirus entry. *Cell* **187**(16), 4261–4271 (2024). 38964329 10.1016/j.cell.2024.06.016

[17] Fernández, I. et al. Structural basis of TMPRSS2 zymogen activation and recognition by the HKU1 seasonal coronavirus. *Cell* **187**(16), 4246–4260 (2024). 38964326 10.1016/j.cell.2024.06.007

[18] Iwata-Yoshikawa, N. et al. Essential role of TMPRSS2 in SARS-CoV-2 infection in murine airways. *Nat. Commun.* **13**, 1–11 (2022). 36243815 10.1038/s41467-022-33911-8 PMC9568946

[19] Liu, J. et al. SARS-CoV-2 cell tropism and multiorgan infection. *Cell Discov.* **7**, 1–4 (2021). 33758165 10.1038/s41421-021-00249-2 PMC7987126

[20] Chu, H. et al. Comparative tropism, replication kinetics, and cell damage profiling of SARS-CoV-2 and SARS-CoV with implications for clinical manifestations, transmissibility, and laboratory studies of COVID-19: An observational study. *Lancet Microbe* **1**, e14–e23 (2020). 32835326 10.1016/S2666-5247(20)30004-5 PMC7173822

[21] Conceicao, C. et al. The SARS-CoV-2 spike protein has a broad tropism for mammalian ACE2 proteins. *PLOS Biol.* **18**, e3001016 (2020). 33347434 10.1371/journal.pbio.3001016 PMC7751883

[22] Koch, J. et al. TMPRSS2 expression dictates the entry route used by SARS-CoV-2 to infect host cells. *EMBO J.* **40**, e107821 (2021). 34159616 10.15252/embj.2021107821 PMC8365257

[23] Jackson, C. B., Farzan, M., Chen, B. & Choe, H. Mechanisms of SARS-CoV-2 entry into cells. *Nat. Rev. Mol. Cell Biol.* **23**, 3–20 (2021). 34611326 10.1038/s41580-021-00418-x PMC8491763

[24] Takeda, M. Proteolytic activation of SARS-CoV-2 spike protein. *Microbiol. Immunol.* **66**, 15–23 (2022). 34561887 10.1111/1348-0421.12945 PMC8652499

[25] Willett, B. J. et al. SARS-CoV-2 Omicron is an immune escape variant with an altered cell entry pathway. *Nat. Microbiol.* **7**, 1161–1179 (2022). 35798890 10.1038/s41564-022-01143-7 PMC9352574

[26] Meng, B. et al. Altered TMPRSS2 usage by SARS-CoV-2 Omicron impacts infectivity and fusogenicity. *Nature* **603**, 706–714 (2022). 35104837 10.1038/s41586-022-04474-x PMC8942856

[27] Hu, B. et al. Spike mutations contributing to the altered entry preference of SARS-CoV-2 omicron BA.1 and BA.2. *Emerg. Microbes Infect.* **11**, 2275–2287 (2022). 36039901 10.1080/22221751.2022.2117098 PMC9542985

[28] Shi, G. et al. Omicron Spike confers enhanced infectivity and interferon resistance to SARS-CoV-2 in human nasal tissue. *Nat. Commun.* **15**, 889 (2024). 38291024 10.1038/s41467-024-45075-8 PMC10828397

[29] Metzdorf, K. et al. TMPRSS2 is essential for SARS-CoV-2 beta and Omicron infection. *Viruses* **15**, 271 (2023). 36851486 10.3390/v15020271 PMC9961888

[30] Huang, I. C. et al. Distinct patterns of IFITM-mediated restriction of filoviruses, SARS coronavirus, and influenza A virus. *PLOS Pathog.* **7**, e1001258 (2011). 21253575 10.1371/journal.ppat.1001258 PMC3017121

[31] Bertram, S. et al. TMPRSS2 activates the human coronavirus 229E for cathepsin-independent host cell entry and is expressed in viral target cells in the respiratory epithelium. *J. Virol.* **87**, 6150–6160 (2013). 23536651 10.1128/JVI.03372-12 PMC3648130

[32] Zheng, M. et al. Bat SARS-Like WIV1 coronavirus uses the ACE2 of multiple animal species as receptor and evades IFITM3 restriction via TMPRSS2 activation of membrane fusion. *Emerg. Microbes Infect.* **9**, 1567 (2020). 32602823 10.1080/22221751.2020.1787797 PMC7473123

[33] Peacock, T. P. et al. The furin cleavage site in the SARS-CoV-2 spike protein is required for transmission in ferrets. *Nat. Microbiol.* **6**, 899–909 (2021). 33907312 10.1038/s41564-021-00908-w PMC7619196

[34] Wrensch, F., Winkler, M. & Pöhlmann, S. IFITM proteins inhibit entry driven by the MERS-coronavirus spike protein: Evidence for cholesterol-independent mechanisms. *Viruses* **6**, 3683–3698 (2014). 25256397 10.3390/v6093683 PMC4189045

[35] Winstone, H. et al. The polybasic cleavage site in SARS-CoV-2 spike modulates viral sensitivity to type I interferon and IFITM2. *J. Virol.* **95**, 10–1128 (2021). 10.1128/JVI.02422-20 PMC8104117 33563656

[36] Xu, F. et al. IFITM3 inhibits SARS-CoV-2 infection and is associated with COVID-19 susceptibility. *Viruses* **14**, 2553 (2022). 36423162 10.3390/v14112553 PMC9692367

[37] Shi, G. et al. Opposing activities of IFITM proteins in SARS-CoV-2 infection. *EMBO J.* **40**, e106501 (2021). 33270927 10.15252/embj.2020106501 PMC7744865

[38] Huang, J. et al. SARS-CoV-2 infection of pluripotent stem cell-derived human lung alveolar type 2 cells elicits a rapid epithelial-intrinsic inflammatory response. *Cell Stem Cell* **27**, 962-973.e7 (2020). 32979316 10.1016/j.stem.2020.09.013 PMC7500949

[39] Xie, Q. et al. Endogenous IFITMs boost SARS-coronavirus 1 and 2 replication whereas overexpression inhibits infection by relocalizing ACE2. *iScience* **26**, 106395 (2023). 36968088 10.1016/j.isci.2023.106395 PMC10009997

[40] Prelli Bozzo, C. et al. IFITM proteins promote SARS-CoV-2 infection and are targets for virus inhibition in vitro. *Nat. Commun.* **12**, 1–13 (2021). 34321474 10.1038/s41467-021-24817-y PMC8319209

[41] Nchioua, R. et al. SARS-CoV-2 variants of concern hijack IFITM2 for efficient replication in human lung cells. *J. Virol.* **96**, e00594 (2022). 35543509 10.1128/jvi.00594-22 PMC9175628

[42] Afar, D. E. H. et al. Catalytic cleavage of the androgen-regulated TMPRSS2 protease results in its secretion by prostate and prostate cancer epithelia. *Cancer Res.* **61**, 1686–1692 (2001). 11245484

[43] Vaarala, M. H. et al. Expression of transmembrane serine protease TMPRSS2 in mouse and human tissues. *J. Pathol.*10.1002/1096-9896(2000)9999:9999 (2000). 10.1002/1096-9896(2000)9999:9999<::AID-PATH743>3.0.CO;2-T 11169526

[44] Dong, M. et al. ACE2, TMPRSS2 distribution and extrapulmonary organ injury in patients with COVID-19. *Biomed. Pharmacother.* **131**, 110678 (2020). 32861070 10.1016/j.biopha.2020.110678 PMC7444942

[45] Singh, M., Bansal, V. & Feschotte, C. A single-cell RNA expression map of human coronavirus entry factors. *Cell Rep.* **32**, 108175 (2020). 32946807 10.1016/j.celrep.2020.108175 PMC7470764

[46] Muus, C. et al. Single-cell meta-analysis of SARS-CoV-2 entry genes across tissues and demographics. *Nat. Med.* **27**, 546–559 (2021). 33654293 10.1038/s41591-020-01227-z PMC9469728

[47] Sungnak, W. et al. SARS-CoV-2 entry factors are highly expressed in nasal epithelial cells together with innate immune genes. *Nat. Med.*10.1038/s41591-020-0868-6 (2020). 32327758 10.1038/s41591-020-0868-6 PMC8637938

[48] Burgueno, J. F. et al. Expression of SARS-CoV-2 entry molecules ACE2 and TMPRSS2 in the gut of patients with IBD. *Inflamm. Bowel Dis.* **26**, 797 (2020). 32333601 10.1093/ibd/izaa085 PMC7188157

[49] Lehmann, M. et al. Human small intestinal infection by SARS-CoV-2 is characterized by a mucosal infiltration with activated CD8+ T cells. *Mucosal Immunol.* **14**, 1381–1392 (2021). 34420043 10.1038/s41385-021-00437-z PMC8379580

[50] Lamers, M. M. et al. SARS-CoV-2 productively infects human gut enterocytes. *Science* **369**, 50–54 (2020). 32358202 10.1126/science.abc1669 PMC7199907

[51] Zhou, J. et al. Infection of bat and human intestinal organoids by SARS-CoV-2. *Nat. Med.* **26**, 1077–1083 (2020). 32405028 10.1038/s41591-020-0912-6

[52] Wu, X. et al. Intestinal damage in COVID-19: SARS-CoV-2 infection and intestinal thrombosis. *Front. Microbiol.* **13**, 860931 (2022). 35391725 10.3389/fmicb.2022.860931 PMC8981312

[53] Guimarães Sousa, S. et al. SARS-CoV-2 infection causes intestinal cell damage: Role of interferon’s imbalance. *Cytokine* **152**, 155826 (2022). 35158258 10.1016/j.cyto.2022.155826 PMC8828414

[54] Cheung, K. S. et al. Gastrointestinal manifestations of SARS-CoV-2 infection and virus load in Fecal samples from a Hong Kong cohort: Systematic review and meta-analysis. *Gastroenterology* **159**, 81–95 (2020). 32251668 10.1053/j.gastro.2020.03.065 PMC7194936

[55] Mao, R. et al. Manifestations and prognosis of gastrointestinal and liver involvement in patients with COVID-19: A systematic review and meta-analysis. *Lancet Gastroenterol. Hepatol.* **5**, 667–678 (2020). 32405603 10.1016/S2468-1253(20)30126-6 PMC7217643

[56] Lin, L. et al. Gastrointestinal symptoms of 95 cases with SARS-CoV-2 infection. *Gut* **69**, 997–1001 (2020). 32241899 10.1136/gutjnl-2020-321013

[57] Lin, B. et al. Prostate-localized and androgen-regulated expression of the membrane-bound serine protease TMPRSS2. *Cancer Res.* **59**, 4180–4184 (1999). 10485450

[58] Lucas, J. M. et al. The androgen-regulated protease TMPRSS2 activates a proteolytic cascade involving components of the tumor microenvironment and promotes prostate cancer metastasis. *Cancer Discov.* **4**, 1310 (2014). 25122198 10.1158/2159-8290.CD-13-1010 PMC4409786

[59] Jacquinet, E. et al. Cloning and characterization of the cDNA and gene for human epitheliasin. *Eur. J. Biochem.* **268**, 2687–2699 (2001). 11322890 10.1046/j.1432-1327.2001.02165.x

[60] Tomlins, S. A. et al. Recurrent fusion of TMPRSS2 and ETS transcription factor genes in prostate cancer. *Science* **310**, 644–648 (2005). 16254181 10.1126/science.1117679

[61] Wang, J., Cai, Y., Ren, C. & Ittmann, M. Expression of variant TMPRSS2/ERG fusion messenger RNAs is associated with aggressive prostate cancer. *Cancer Res.* **66**, 8347–8351 (2006). 16951141 10.1158/0008-5472.CAN-06-1966

[62] Qiao, Y. et al. Targeting transcriptional regulation of SARS-CoV-2 entry factors ACE2 and TMPRSS2. *Proc. Natl. Acad. Sci. USA* **118**, e2021450118 (2020). 33310900 10.1073/pnas.2021450118 PMC7817128

[63] Deng, Q., ur Rasool, R., Russell, R. M., Natesan, R. & Asangani, I. A. Targeting androgen regulation of TMPRSS2 and ACE2 as a therapeutic strategy to combat COVID-19. *iScience* **24**(3), 102254. 10.1016/j.isci.2021.102254 (2021). 33681723 10.1016/j.isci.2021.102254 PMC7919514

[64] Chen, Y. et al. A high-throughput screen for TMPRSS2 expression identifies FDA-approved compounds that can limit SARS-CoV-2 entry. *Nat. Commun.* **12**, 1–15 (2021). 34162861 10.1038/s41467-021-24156-y PMC8222394

[65] Pommerenke, C. et al. Identification of cell lines CL-14, CL-40 and CAL-51 as suitable models for SARS-CoV-2 infection studies. *PLoS ONE* **16**, e0255622 (2021). 34339474 10.1371/journal.pone.0255622 PMC8328321

[66] Partridge, L. J. et al. Ace2-independent interaction of sars-cov-2 spike protein with human epithelial cells is inhibited by unfractionated heparin. *Cells* **10**, 1419 (2021). 34200372 10.3390/cells10061419 PMC8229176

[67] Pandamooz, S. et al. Experimental models of SARS-CoV-2 infection: Possible platforms to study COVID-19 pathogenesis and potential treatments. *Ann. Rev. Pharmacol. Toxicol.* **62**, 25–53 (2022). 33606962 10.1146/annurev-pharmtox-121120-012309

[68] Pires De Souza, G. A. et al. Choosing a cellular model to study SARS-CoV-2. *Front. Cell. Infect. Microbiol.* **12**, 1003608 (2022). 36339347 10.3389/fcimb.2022.1003608 PMC9634005

[69] Crozier, T. W. M. et al. Quantitative proteomic analysis of SARS-CoV-2 infection of primary human airway ciliated cells and lung epithelial cells demonstrates the effectiveness of SARS-CoV-2 innate immune evasion. *Wellcome Open Res.* **7**, 224 (2022). 36483314 10.12688/wellcomeopenres.17946.1 PMC9706147

[70] Ye, C. et al. Analysis of SARS-CoV-2 infection dynamic in vivo using reporter-expressing viruses. *Proc. Natl. Acad. Sci. USA* **118**, e2111593118 (2021). 34561300 10.1073/pnas.2111593118 PMC8521683

[71] Chiem, K. et al. Generation and characterization of recombinant SARS-CoV-2 expressing reporter genes. *J. Virol.*10.1128/JVI.02209-20 (2021). 33431557 10.1128/JVI.02209-20 PMC8092710

[72] Chan, J. F. W. et al. Virological features and pathogenicity of SARS-CoV-2 Omicron BA.2. *Cell Rep. Med.* **3**, 100743 (2022). 36084644 10.1016/j.xcrm.2022.100743 PMC9420712

[73] Tzelepis, K. et al. A CRISPR dropout screen identifies genetic vulnerabilities and therapeutic targets in acute myeloid leukemia. *Cell Rep.* **17**, 1193–1205 (2016). 27760321 10.1016/j.celrep.2016.09.079 PMC5081405

[74] Maxwell, P. H. et al. The tumour suppressor protein VHL targets hypoxia-inducible factors for oxygen-dependent proteolysis. *Nature* **399**, 271–275 (1999). 10353251 10.1038/20459

[75] Ohh, M. et al. Ubiquitination of hypoxia-inducible factor requires direct binding to the beta-domain of the von Hippel-Lindau protein. *Nat. Cell Biol.* **2**, 423–427 (2000). 10878807 10.1038/35017054

[76] Tanimoto, K. Mechanism of regulation of the hypoxia-inducible factor-1alpha by the von Hippel-Lindau tumor suppressor protein. *EMBO J.* **19**, 4298–4309 (2000). 10944113 10.1093/emboj/19.16.4298 PMC302039

[77] Champion, A. et al. MORC3 represses the HCMV major immediate early promoter in myeloid cells in the absence of PML nuclear bodies. *J. Med. Virol.* **95**, e29227 (2023). 38009611 10.1002/jmv.29227 PMC10952291

[78] Jeronimo, C. & Robert, F. The histone chaperone FACT: A guardian of chromatin structure integrity. *Transcription* **13**, 16–38 (2022). 35485711 10.1080/21541264.2022.2069995 PMC9467567

[79] Mason, P. B. & Struhl, K. The FACT complex travels with elongating RNA polymerase II and is important for the fidelity of transcriptional initiation in vivo. *Mol. Cell. Biol.* **23**, 8323 (2003). 14585989 10.1128/MCB.23.22.8323-8333.2003 PMC262413

[80] Jamin, A. & Wiebe, M. S. Barrier to autointegration factor (BANF1): Interwoven roles in nuclear structure, genome integrity, innate immunity, stress responses and progeria. *Curr. Opin. Cell Biol.* **34**, 61 (2015). 26072104 10.1016/j.ceb.2015.05.006 PMC4522355

[81] Schoenfeld, A. R., Davidowitz, E. J. & Burk, R. D. Elongin BC complex prevents degradation of von Hippel-Lindau tumor suppressor gene products. *Proc. Natl. Acad. Sci. USA* **97**, 8507–8512 (2000). 10900011 10.1073/pnas.97.15.8507 PMC26978

[82] Mykytyn, A. Z. et al. SARS-CoV-2 Omicron entry is type II transmembrane serine protease-mediated in human airway and intestinal organoid models. *J. Virol.* **97**, e00851 (2023). 37555660 10.1128/jvi.00851-23 PMC10506477

[83] Pastorio, C. et al. Impact of mutations defining SARS-CoV-2 Omicron subvariants BA.2.12.1 and BA.4/5 on Spike function and neutralization. *iScience* **26**, 108299 (2023). 38026181 10.1016/j.isci.2023.108299 PMC10661123

[84] Zhang, L. et al. SARS-CoV-2 BA.2.86 enters lung cells and evades neutralizing antibodies with high efficiency. *Cell* **187**, 596–608 (2024). 38194966 10.1016/j.cell.2023.12.025 PMC11317634

[85] Tang, W. F., Tran, A. T., Wang, L. Y. & Horng, J. T. SARS-CoV-2 pandemics: An update of CRISPR in diagnosis and host–virus interaction studies. *Biomed. J.* **46**, 100587 (2023). 36849044 10.1016/j.bj.2023.02.007 PMC9957976

[86] Bailey, A. L. & Diamond, M. S. A Crisp(r) new perspective on SARS-CoV-2 biology. *Cell* **184**, 15–17 (2021). 33338422 10.1016/j.cell.2020.12.003 PMC7746090

[87] Biering, S. B. et al. Genome-wide bidirectional CRISPR screens identify mucins as host factors modulating SARS-CoV-2 infection. *Nat. Genet.* **54**, 1078–1089 (2022). 35879412 10.1038/s41588-022-01131-x PMC9355872

[88] Rebendenne, A. et al. Bidirectional genome-wide CRISPR screens reveal host factors regulating SARS-CoV-2, MERS-CoV and seasonal HCoVs. *Nat. Genet.* **54**, 1090–1102 (2022). 35879413 10.1038/s41588-022-01110-2 PMC11627114

[89] Daniloski, Z. et al. Identification of required host factors for SARS-CoV-2 infection in human cells. *Cell* **184**, 92-105.e16 (2021). 33147445 10.1016/j.cell.2020.10.030 PMC7584921

[90] Wang, R. et al. Genetic screens identify host factors for SARS-CoV-2 and common cold coronaviruses. *Cell* **184**, 106-119.e14 (2021). 33333024 10.1016/j.cell.2020.12.004 PMC7723770

[91] Zhu, Y. et al. A genome-wide CRISPR screen identifies host factors that regulate SARS-CoV-2 entry. *Nat. Commun.* **12**, 1–11 (2021). 33574281 10.1038/s41467-021-21213-4 PMC7878750

[92] Wei, J. et al. Genome-wide CRISPR screens reveal host factors critical for SARS-CoV-2 infection. *Cell* **184**, 76-91.e13 (2021). 33147444 10.1016/j.cell.2020.10.028 PMC7574718

[93] Chan, K. et al. Survival-based CRISPR genetic screens across a panel of permissive cell lines identify common and cell-specific SARS-CoV-2 host factors. *Heliyon* **9**, e12744 (2023). 36597481 10.1016/j.heliyon.2022.e12744 PMC9800021

[94] Hou, J. et al. Integrated multi-omics analyses identify anti-viral host factors and pathways controlling SARS-CoV-2 infection. *Nat. Commun.* **15**, 1–14 (2024). 38168026 10.1038/s41467-023-44175-1 PMC10761986

[95] Israeli, M. et al. Genome-wide CRISPR screens identify GATA6 as a proviral host factor for SARS-CoV-2 via modulation of ACE2. *Nat. Commun.* **13**, 1–16 (2022). 35469023 10.1038/s41467-022-29896-z PMC9039069

[96] Sherman, E. J. et al. Identification of cell type specific ACE2 modifiers by CRISPR screening. *PLOS Pathog.* **18**, e1010377 (2022). 35231079 10.1371/journal.ppat.1010377 PMC8929698

[97] Cavadas, M. A. S., Cheong, A. & Taylor, C. T. The regulation of transcriptional repression in hypoxia. *Exp. Cell Res.* **356**, 173–181 (2017). 28219680 10.1016/j.yexcr.2017.02.024

[98] Choudhry, H. & Harris, A. L. Advances in hypoxia-inducible factor biology. *Cell Metab.* **27**, 281–298 (2018). 29129785 10.1016/j.cmet.2017.10.005

[99] Batie, M., del Peso, L. & Rocha, S. Hypoxia and chromatin: A focus on transcriptional repression mechanisms. *Biomedicines* **6**, 47 (2018). 29690561 10.3390/biomedicines6020047 PMC6027312

[100] Cavadas, M. A. S. et al. REST is a hypoxia-responsive transcriptional repressor. *Sci. Rep.* **6**, 1–13 (2016). 27531581 10.1038/srep31355 PMC4987654

[101] Kulshreshtha, R. et al. A microRNA signature of hypoxia. *Mol. Cell. Biol.* **27**, 1859–1867 (2007). 17194750 10.1128/MCB.01395-06 PMC1820461

[102] Dengler, V. L., Galbraith, M. D. & Espinosa, J. M. Transcriptional regulation by hypoxia inducible factors. *Crit. Rev. Biochem. Mol. Biol.* **49**, 1 (2014). 24099156 10.3109/10409238.2013.838205 PMC4342852

[103] Zhang, R. et al. MiRNA let-7b promotes the development of hypoxic pulmonary hypertension by targeting ACE2. *Am. J. Physiol. Lung Cell. Mol. Physiol.* **316**, L547–L557 (2019). 30628484 10.1152/ajplung.00387.2018

[104] Wing, P. A. C. et al. Hypoxic and pharmacological activation of HIF inhibits SARS-CoV-2 infection of lung epithelial cells. *Cell Rep.* **35**, 109020 (2021). 33852916 10.1016/j.celrep.2021.109020 PMC8020087

[105] Codo, A. C. et al. Elevated glucose levels favor SARS-CoV-2 infection and monocyte response through a HIF-1α/glycolysis-dependent axis. *Cell Metab.* **32**, 437-446.e5 (2020). 32697943 10.1016/j.cmet.2020.07.007 PMC7367032

[106] Sturrock, A., Zimmerman, E., Helms, M., Liou, T. G. & Paine, R. Hypoxia induces expression of angiotensin-converting enzyme II in alveolar epithelial cells: Implications for the pathogenesis of acute lung injury in COVID-19. *Physiol. Rep.* **9**, e14854 (2021). 33991451 10.14814/phy2.14854 PMC8123561

[107] Imperio, G. E. et al. Hypoxia alters the expression of ACE2 and TMPRSS2 SARS-CoV-2 cell entry mediators in hCMEC/D3 brain endothelial cells. *Microvasc. Res.* **138**, 104232 (2021). 34416267 10.1016/j.mvr.2021.104232 PMC8372440

[108] Shilts, J. et al. LRRC15 mediates an accessory interaction with the SARS-CoV-2 spike protein. *PLOS Biol.* **21**, e3001959 (2023). 36735681 10.1371/journal.pbio.3001959 PMC9897555

[109] Burr, M. L. et al. CMTM6 maintains the expression of PD-L1 and regulates anti-Tumour immunity. *Nature* **549**, 101–105 (2017). 28813417 10.1038/nature23643 PMC5706633

[110] Menzies, S. A. et al. The sterol-responsive RNF145 E3 ubiquitin ligase mediates the degradation of HMG-CoA reductase together with gp78 and hrd1. *Elife* **7**, e40009 (2018). 30543180 10.7554/eLife.40009 PMC6292692

[111] Morgens, D. W. et al. Genome-scale measurement of off-target activity using Cas9 toxicity in high-throughput screens. *Nat. Commun.* **8**, 15178 (2017). 28474669 10.1038/ncomms15178 PMC5424143

[112] Wang, T., Wei, J. J., Sabatini, D. M. & Lander, E. S. Genetic screens in human cells using the CRISPR-Cas9 system. *Science* **343**, 80–84 (2014). 24336569 10.1126/science.1246981 PMC3972032

[113] Michlits, G. et al. Multilayered VBC score predicts sgRNAs that efficiently generate loss-of-function alleles. *Nat. Methods* **17**, 708–716 (2020). 32514112 10.1038/s41592-020-0850-8

[114] Ortmann, B. M. et al. The HIF complex recruits the histone methyltransferase SET1B to activate specific hypoxia-inducible genes. *Nat. Genet.* **53**, 1022–1035 (2021). 34155378 10.1038/s41588-021-00887-y PMC7611696

[115] Lambert, S. A. et al. The human transcription factors. *Cell* **172**, 650–665 (2018). 29425488 10.1016/j.cell.2018.01.029 PMC12908702

[116] Medvedeva, Y. A. et al. EpiFactors: A comprehensive database of human epigenetic factors and complexes. *Database*10.1093/database/bav067 (2015). 26153137 10.1093/database/bav067 PMC4494013

[117] Williams, R. T. et al. ZBTB1 regulates asparagine synthesis and leukemia cell response to L-asparaginase. *Cell Metab.* **31**, 852-861.e6 (2020). 32268116 10.1016/j.cmet.2020.03.008 PMC7219601

[118] Gu, Z. et al. Silencing of LINE-1 retrotransposons is a selective dependency of myeloid leukemia. *Nat. Genet.* **53**, 672–682 (2021). 33833453 10.1038/s41588-021-00829-8 PMC8270111

[119] Hart, T. et al. Evaluation and design of genome-wide CRISPR/SpCas9 knockout screens. *G3* **7**, 2719–2727 (2017). 28655737 10.1534/g3.117.041277 PMC5555476

[120] Doench, J. G. et al. Optimized sgRNA design to maximize activity and minimize off-target effects of CRISPR-Cas9. *Nat. Biotechnol.* **34**, 184–191 (2016). 26780180 10.1038/nbt.3437 PMC4744125

[121] DeWeirdt, P. C. et al. Genetic screens in isogenic mammalian cell lines without single cell cloning. *Nat. Commun.* **11**, 1–15 (2020). 32029722 10.1038/s41467-020-14620-6 PMC7005275

[122] Mölder, F. et al. Sustainable data analysis with Snakemake. *F1000Research* **10**, 33 (2021). 34035898 10.12688/f1000research.29032.1 PMC8114187

[123] Martin, M. Cutadapt removes adapter sequences from high-throughput sequencing reads. *EMBnet J.* **17**, 10–12 (2011).

[124] Li, W. et al. MAGeCK enables robust identification of essential genes from genome-scale CRISPR/Cas9 knockout screens. *Genome Biol.* **15**, 554 (2014). 25476604 10.1186/s13059-014-0554-4 PMC4290824

[125] Hartenian, E., Gilbertson, S., Federspiel, J. D., Cristea, I. M. & Glaunsinger, B. A. RNA decay during gammaherpesvirus infection reduces RNA polymerase II occupancy of host promoters but spares viral promoters. *PLOS Pathog.* **16**, e1008269 (2020). 32032393 10.1371/journal.ppat.1008269 PMC7032723

[126] Brevini, T. et al. FXR inhibition may protect from SARS-CoV-2 infection by reducing ACE2. *Nat.* **615**, 134–142 (2022). 10.1038/s41586-022-05594-0 PMC9977684 36470304

[127] Grodzki, M. et al. Genome-scale CRISPR screens identify host factors that promote human coronavirus infection. *Genome Med.* **14**, 1–18 (2022). 35086559 10.1186/s13073-022-01013-1 PMC8792531

[128] Pleguezuelos-Manzano, C. et al. Establishment and culture of human intestinal organoids derived from adult stem cells. *Curr. Protoc. Immunol.* **130**, e106 (2020). 32940424 10.1002/cpim.106 PMC9285512

[129] Tysoe, O. C. et al. Isolation and propagation of primary human cholangiocyte organoids for the generation of bioengineered biliary tissue. *Nat. Protoc.* **14**, 1884–1925 (2019). 31110298 10.1038/s41596-019-0168-0

[130] Sampaziotis, F. et al. Cholangiocyte organoids can repair bile ducts after transplantation in the human liver. *Science* **371**, 839–846 (2021). 33602855 10.1126/science.aaz6964 PMC7610478

