# Supplementary material for: CRISPR-Cas9 genetic screens reveal regulation of TMPRSS2 by the Elongin BC-VHL complex
Source: Sci Rep. 2025 Apr 7;15:11907. doi: 10.1038/s41598-025-95644-0 (PMC11976923; doi:10.1038/s41598-025-95644-0)

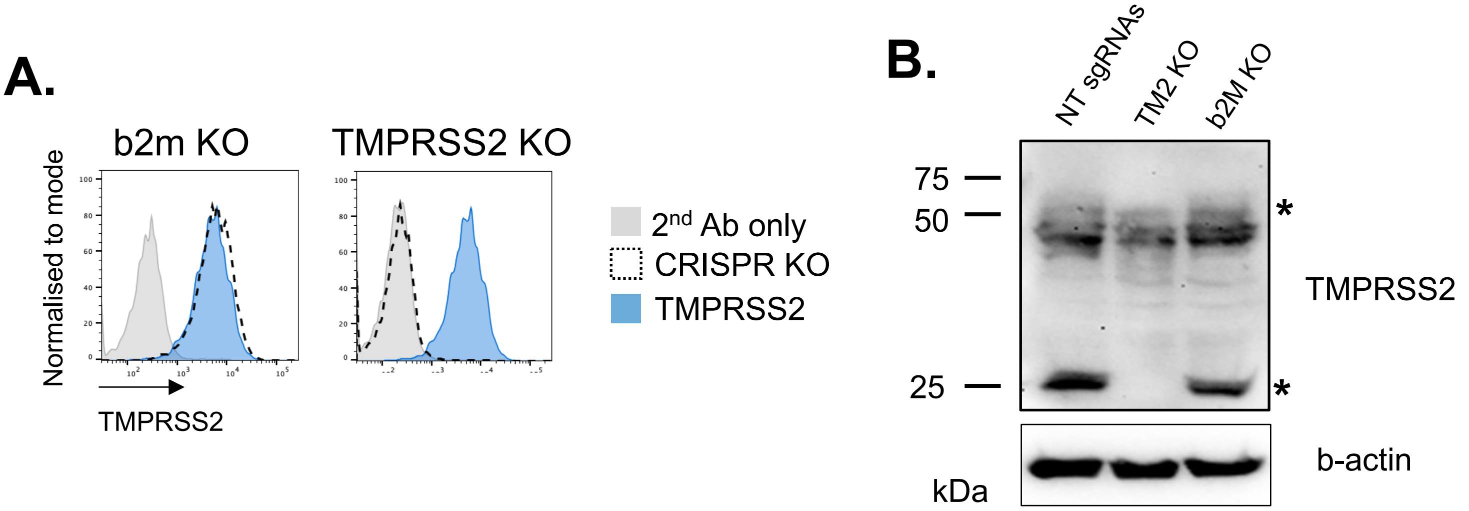

Supplementary figure S1. Specificity of anti-TMPRSS2 antibody for cell surface staining. A. Caco-2 Cas9 cells stably expressing sgRNAs specific for *TMPRSS2* or *b2m* were stained with the TMPRSS2-specific antibody and analysed by flow cytometry. **B.** Caco-2 Cas9 cells stably expressing sgRNAs specific for *TMPRSS2*, *b2m* or control non targeting (NT) sgRNAs were lysed and analysed by immunoblot with the antibody specific for TMPRSS2 or b-actin. Asterisks denote low and high molecular weight forms of TMPRSS2.

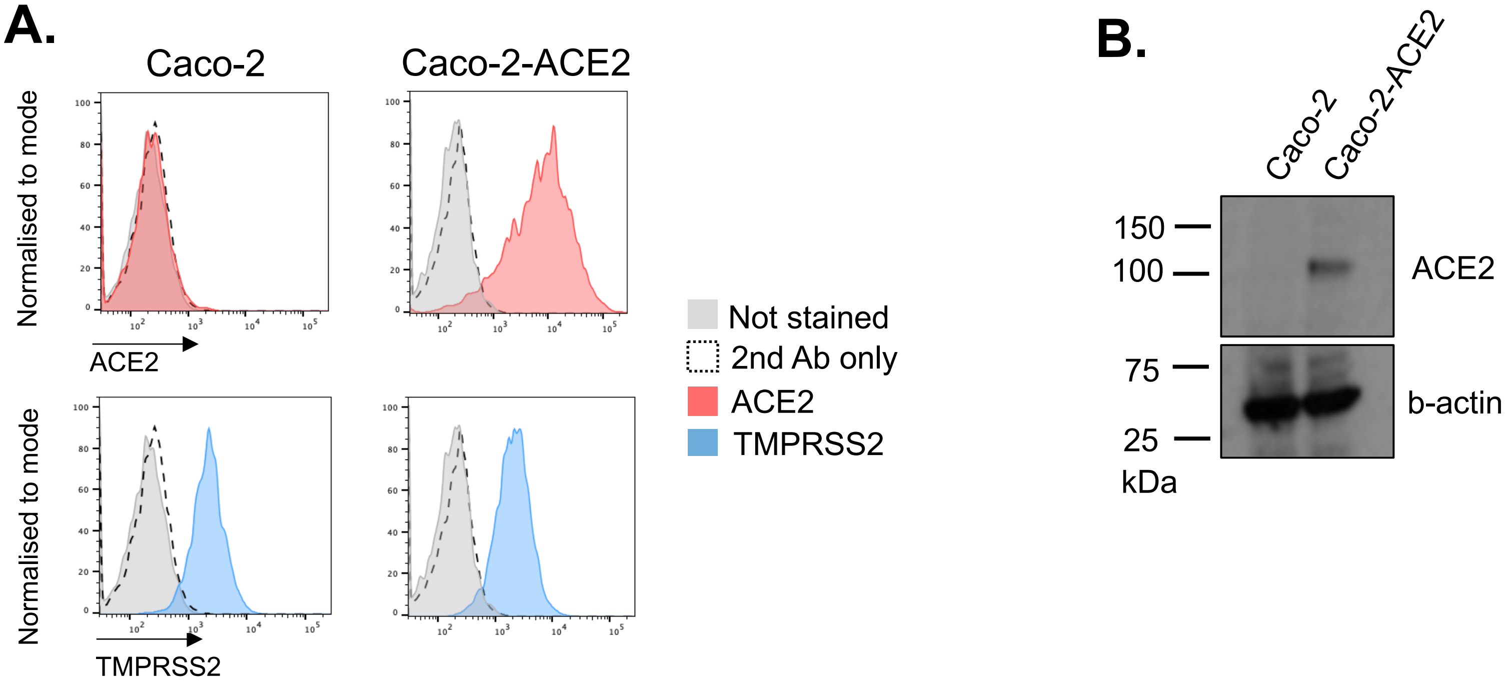

Supplementary figure S2. Caco-2 cells expressing exogenous cell surface ACE2. A. Caco-2 Cas9 cells and Caco-2-ACE2 Cas9 cells were stained with the antibody specific for ACE2 and TMPRSS2 or secondary antibody alone as a control and analysed by flow cytometry. **B.** Caco-2 Cas9 and Caco-2-ACE2 Cas9 cells from the panel S2A were lysed and analysed by immunoblot with the antibody specific ACE2 or b-actin.

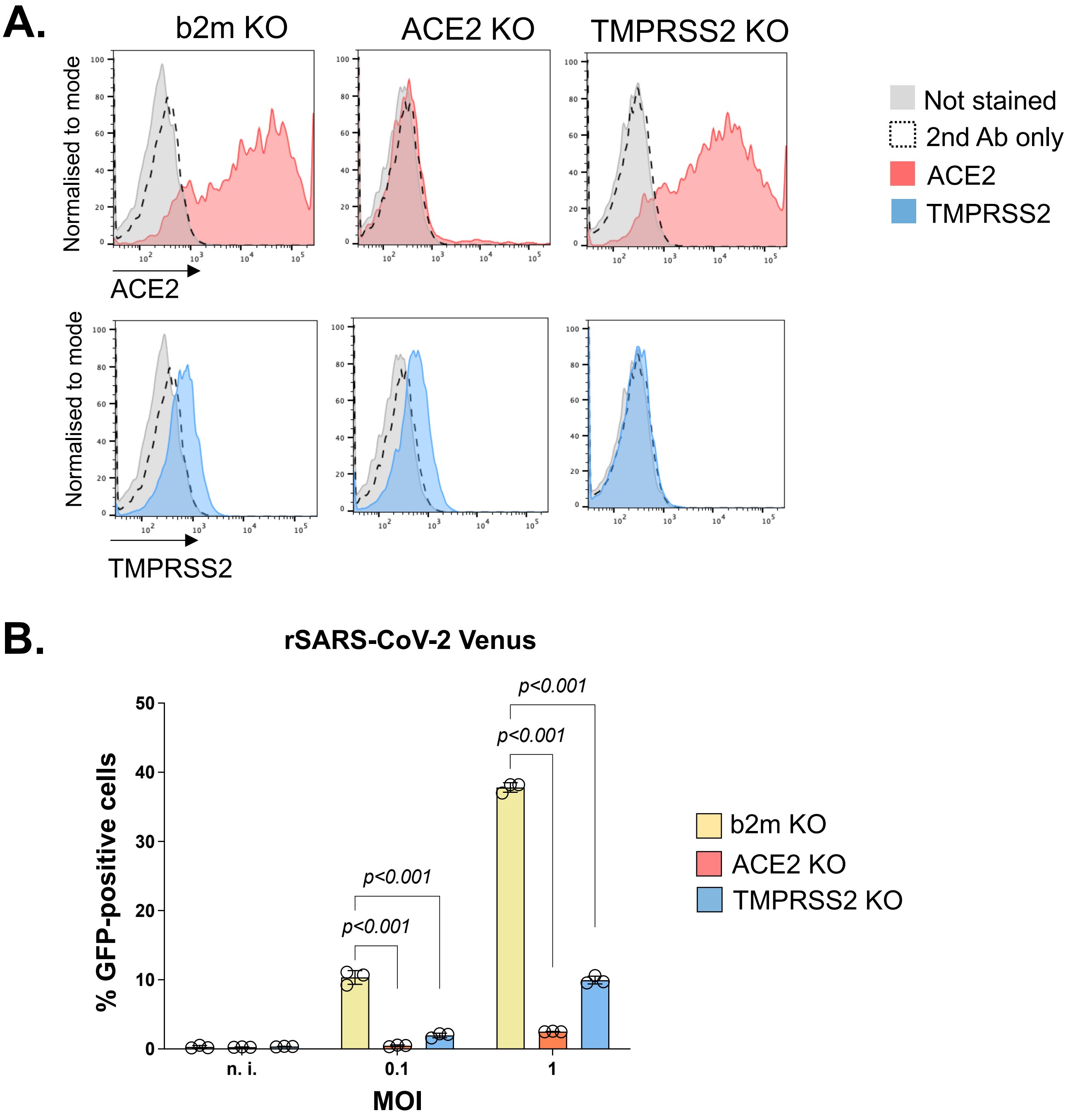

Supplementary figure S3. TMPRSS2 KO affects SARS-CoV-2 entry into lung epithelial Calu-3 cells. **A.** Calu-3 Cas9 cells stably expressing sgRNAs specific for for *b2m*, *ACE2* or *TMPRSS2* were stained with ACE2- or TMPRSS2-specific antibody or secondary antibody alone as a control and analysed by flow cytometry. **B.** Calu3 Cas9 cells from the panel S3A were infected with SARS-CoV-2 Venus at an MOI of 0.1 or 1 and analysed by automated microscopy. Y-axis indicates percentage of GFP-positive cells. Data are presented as mean of n=3 biological replicates ± s.d. The statistical significance was assessed by two-way ANOVA and Bonferroni's multiple comparison correction.

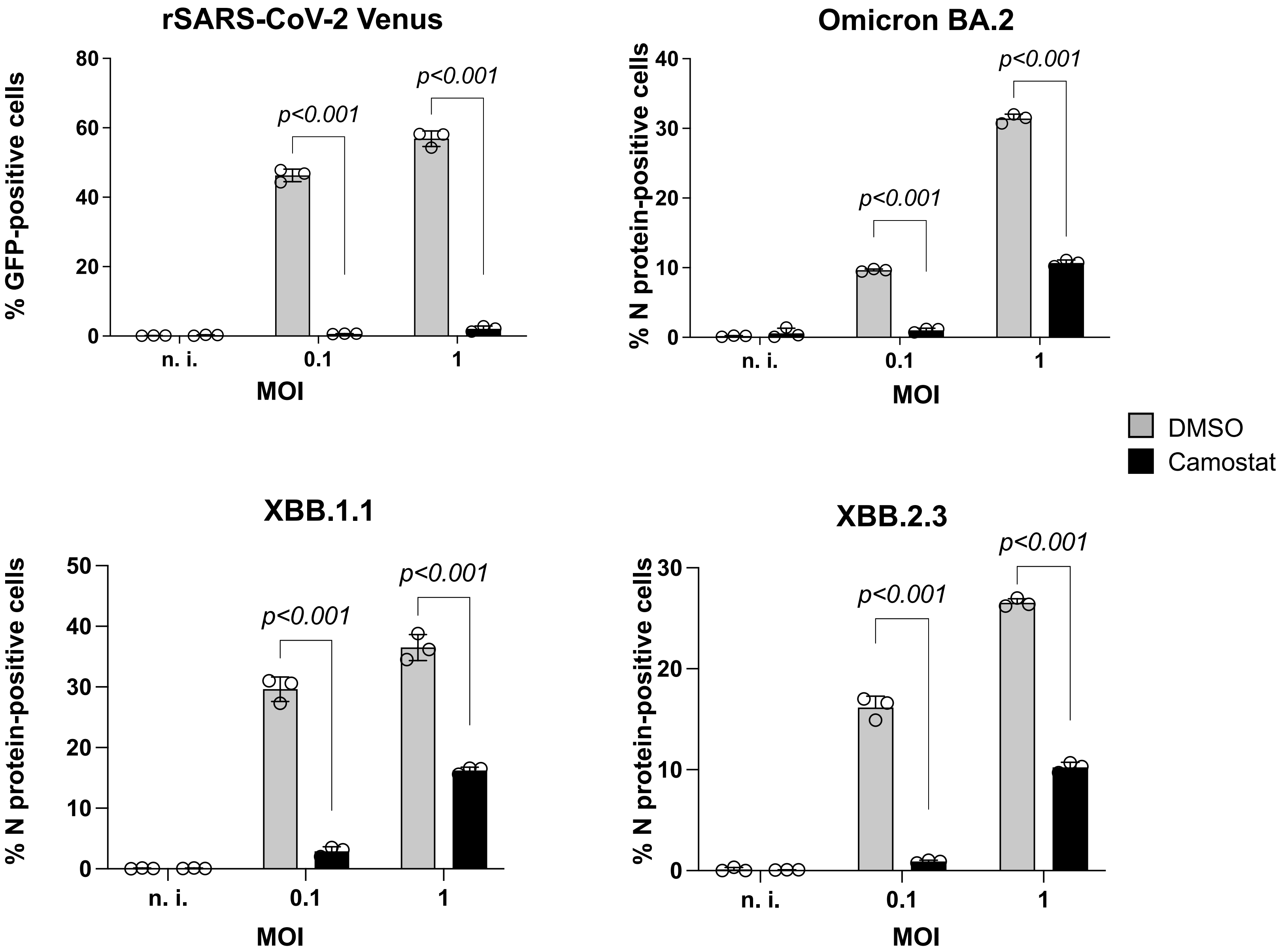

Supplementary figure S4. SARS-CoV-2 entry into Caco-2-ACE2 cells is reduced by Camostat mesilate. Caco-2-ACE2 Cas9 were infected with rSARS-CoV-2 Venus, Omicron BA.2, Omicron XBB.1.1 and XBB.2.3 subvariants at an MOI of 0.1 or 1 in the presence of 200 uM Camostat mesilate or DMSO control, fixed 20 hpi and analysed by automated microscopy. Y-axis indicates percentage of GFP (rSARS-CoV-2 Venus) or N-protein (BA.2, XBB.1.1 and XBB.2.3)-positive cells. Data are presented as mean of *n*=3 biological replicates ± s.d. The statistical significance was assessed by two-way ANOVA and Bonferroni's multiple comparison correction.

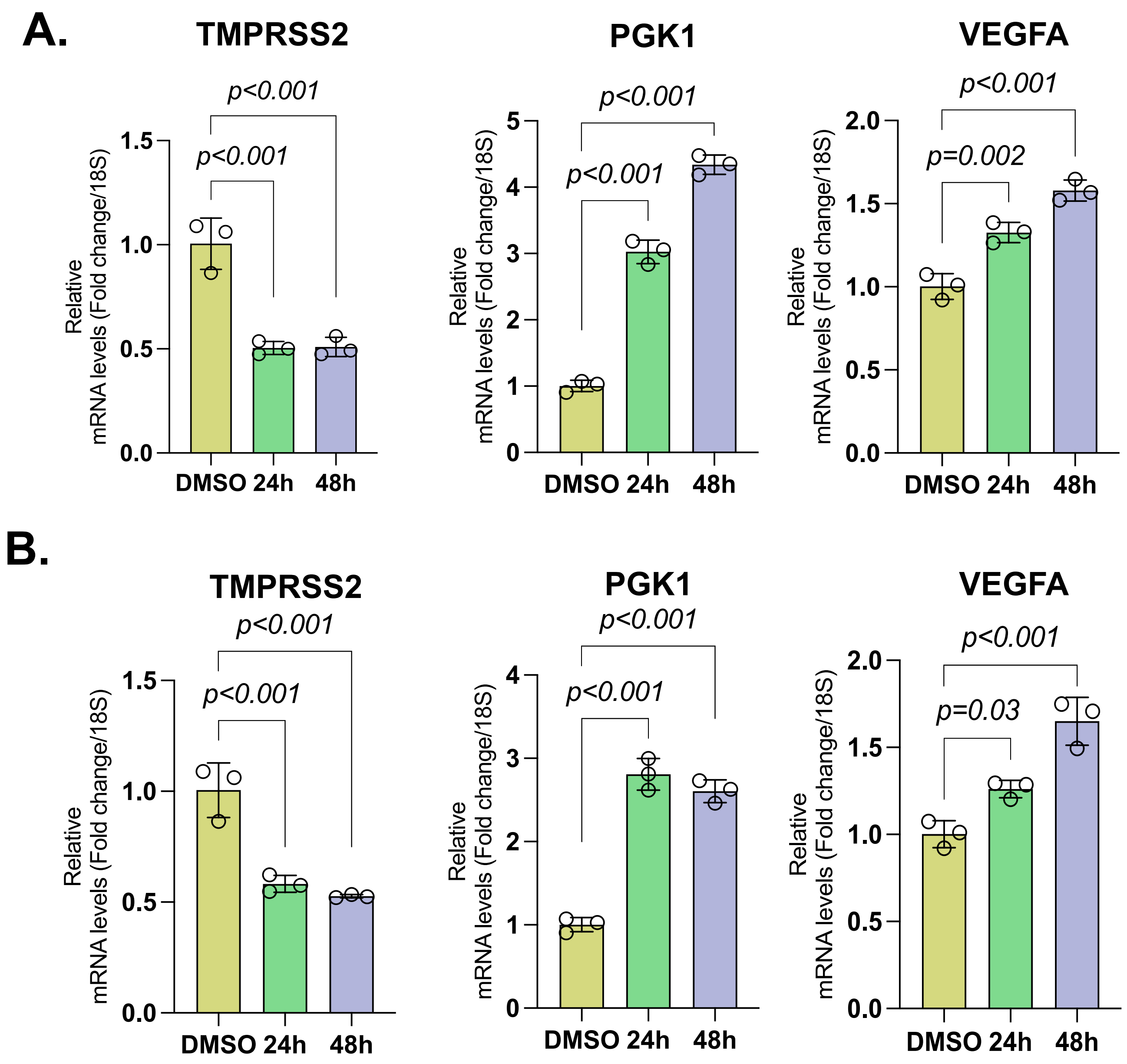

Supplementary figure S5. PHD inhibitor treatment reduces *TMPRSS2* expression in Caco-2 Cas9 cells. A, B. The cells were treated with 100 uM FG-4592 (roxadustat) (A), 50 uM daprodustat (B) or DMSO as a control for 24h or 48h, subjected to RNA extraction followed by RT-qPCR analysis with the primers specific for *TMPRSS2*, *PGK1*, *VEGFA* and *18S*. Data are presented as mean of *n*=3 technical replicates ± s.d. The statistical significance was assessed by one-way ANOVA and Bonferroni's multiple comparison correction.

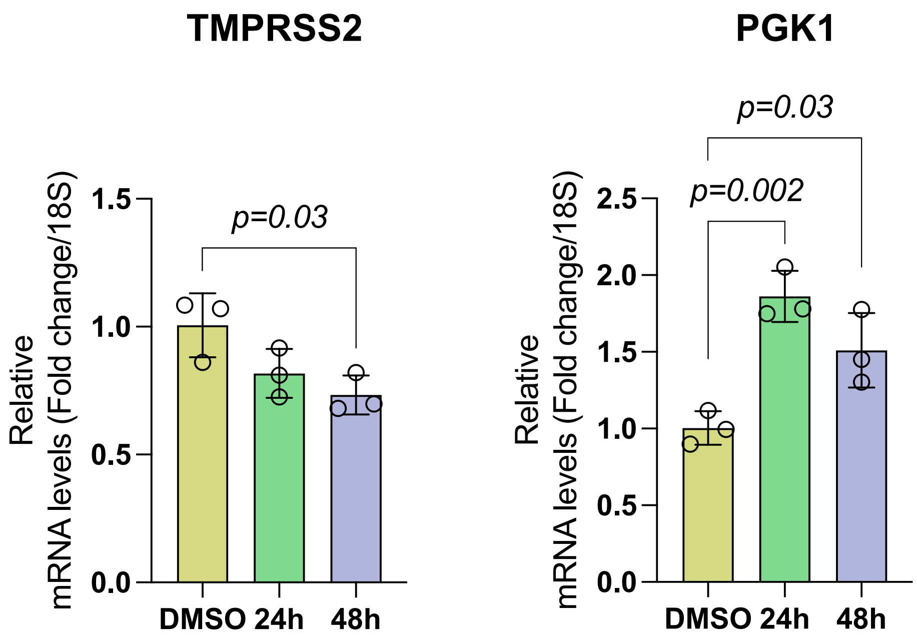

Supplementary figure S6. Roxadustat treatment reduces *TMPRSS2* expression in primary human intestinal organoids. The cells were treated with 100 uM FG-4592 (roxadustat) or DMSO as a control for 24h or 48h, subjected to RNA extraction followed by RT-qPCR analysis with the primers specific for *TMPRSS2*, *PGK1* and *18S*. Data are presented as mean of *n*=3 technical replicates ± s.d. The statistical significance was assessed by one-way ANOVA and Bonferroni's multiple comparison correction.

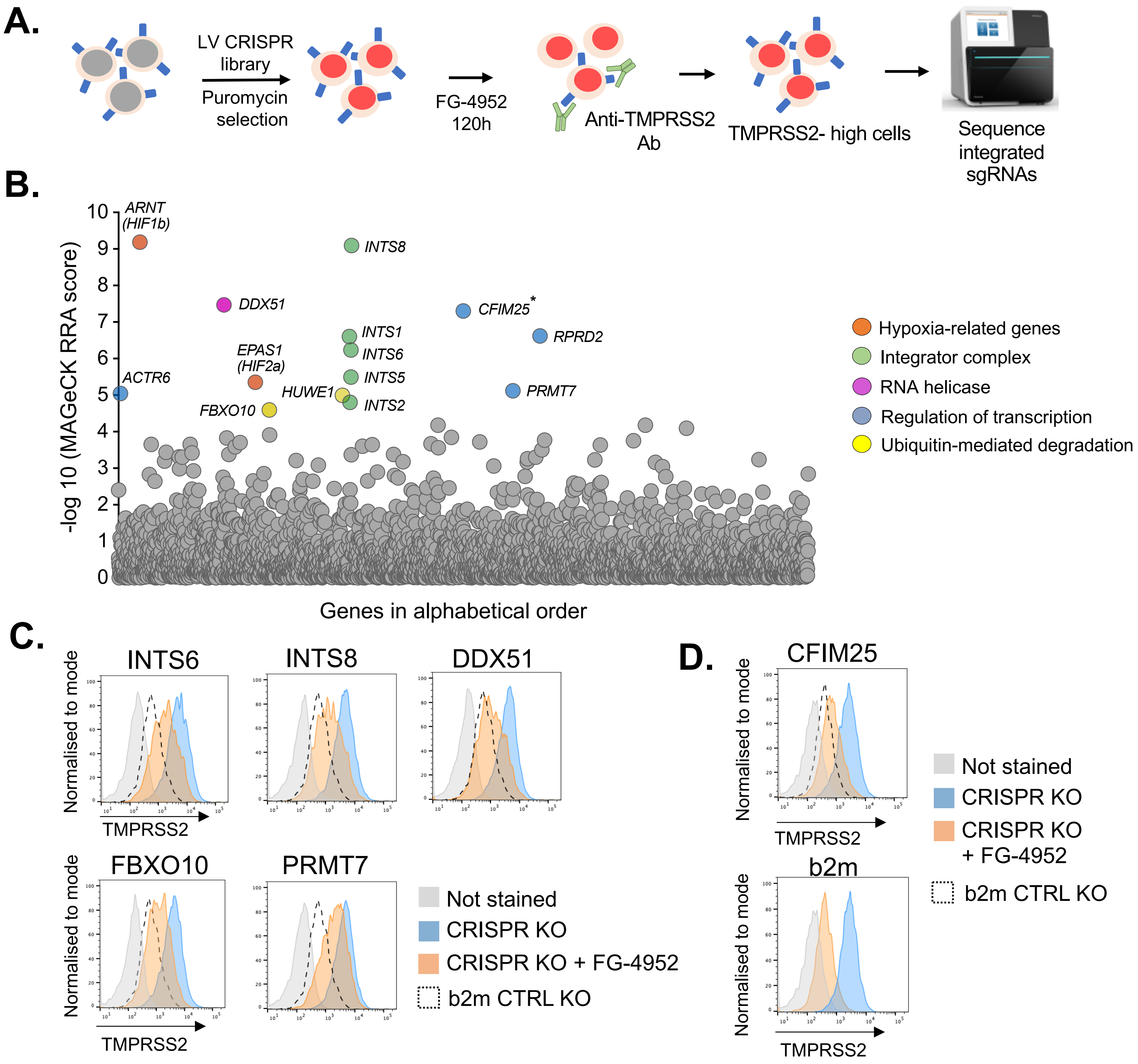

Supplementary figure S7. Complementary CRISPR-Cas9 screen identifies HIFs as regulators of TMPRSS2 expression. **A.** Schematic workflow of the targeted CRISPR-Cas9 screen. Caco-2 cells were transduced with lentiviruses encoding CRISPR library followed by selection with puromycin. Cells were treated with 100 uM Roxadustat (FG-4592) for 120 hours and the TMPRSS2 high-expressing population was enriched using FACS with the specific antibody, subjected to genomic DNA isolation followed by Illumina sequencing of the integrated gRNAs. **B.** MAGecK RRA scores of the sgRNAs enriched in the EMTR CRISPR library screen. *RRA score for the *CFIM25* hit was calculated based on four sgRNAs, as two overlapping sgRNAs were identified for *CFIM25* in the library (differ in a single nucleotide only) and one sgRNA was removed from the analysis. **C, D.** Validation of the hits obtained in the CRISPR-Cas9 screen. Caco2-ACE2 Cas9 cells expressing combinations of the specific gRNAs were stained with anti-TMPRSS2 antibody and analysed by flow cytometry.

Full-length blot images

Figure 4B

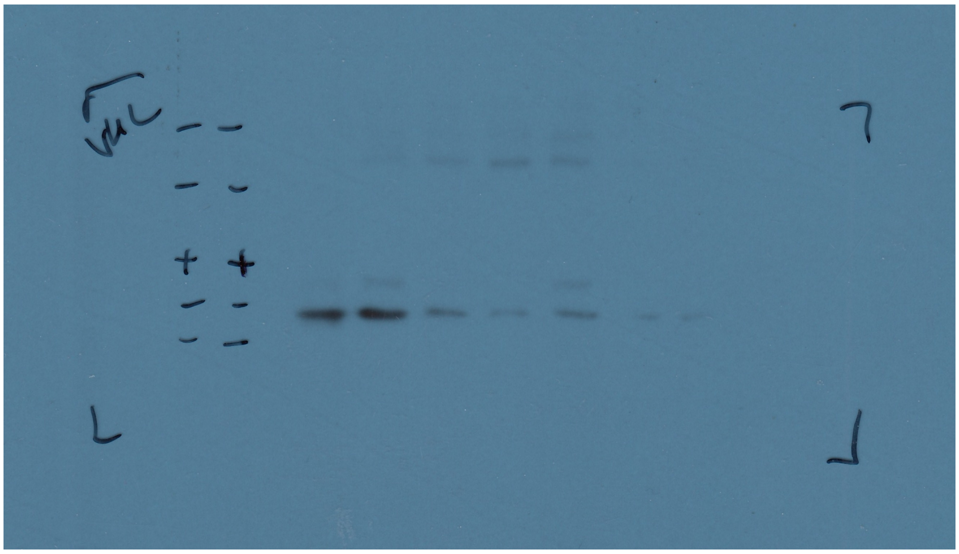

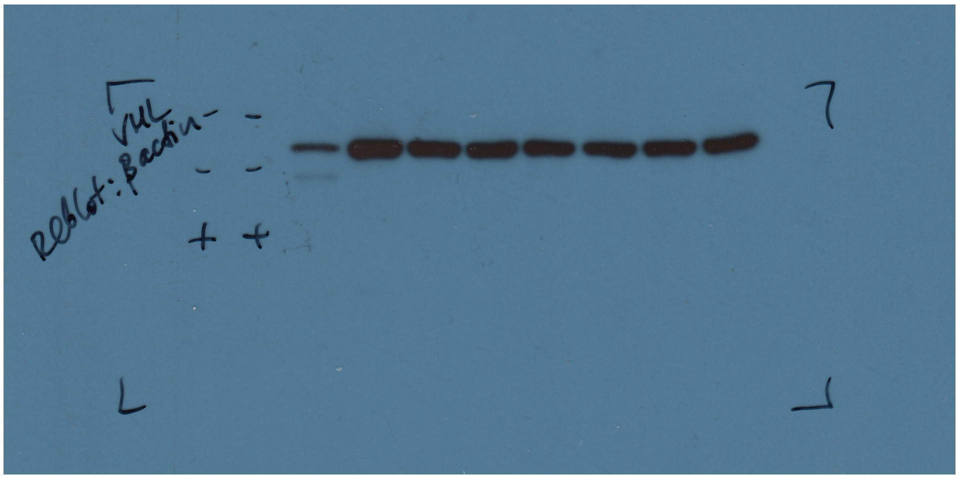

Figure 5B

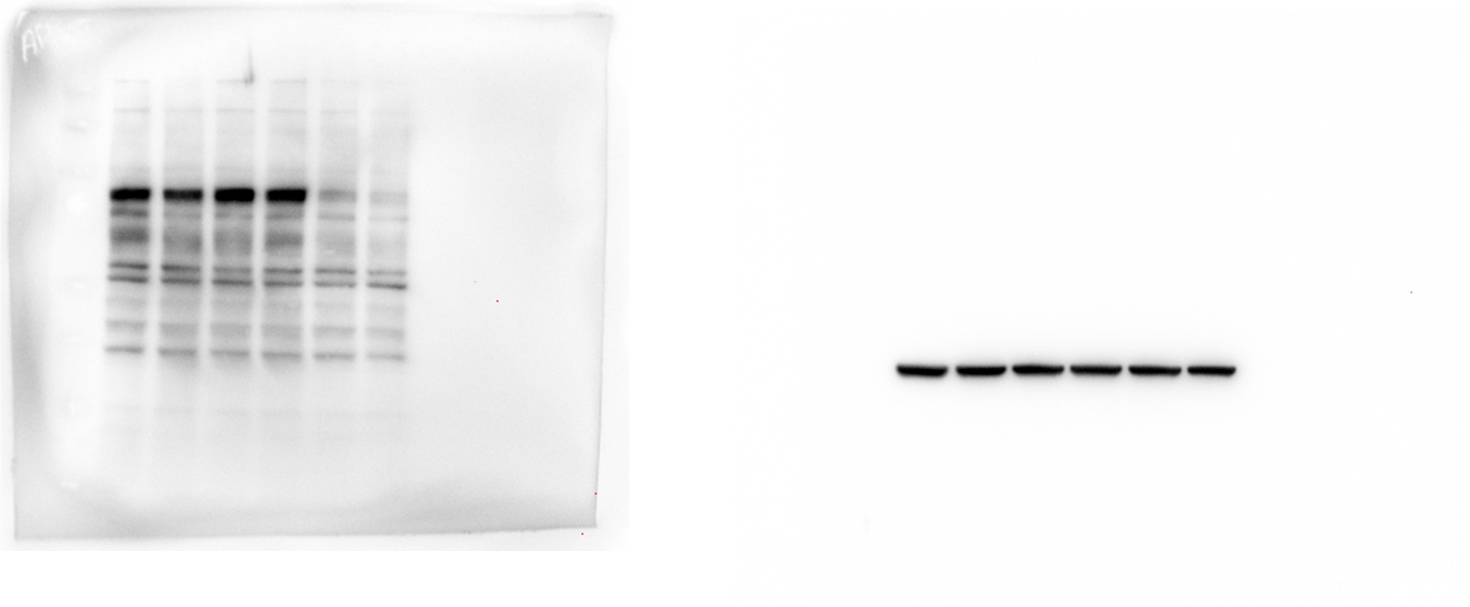

Figure 5B (overlay with ladder)

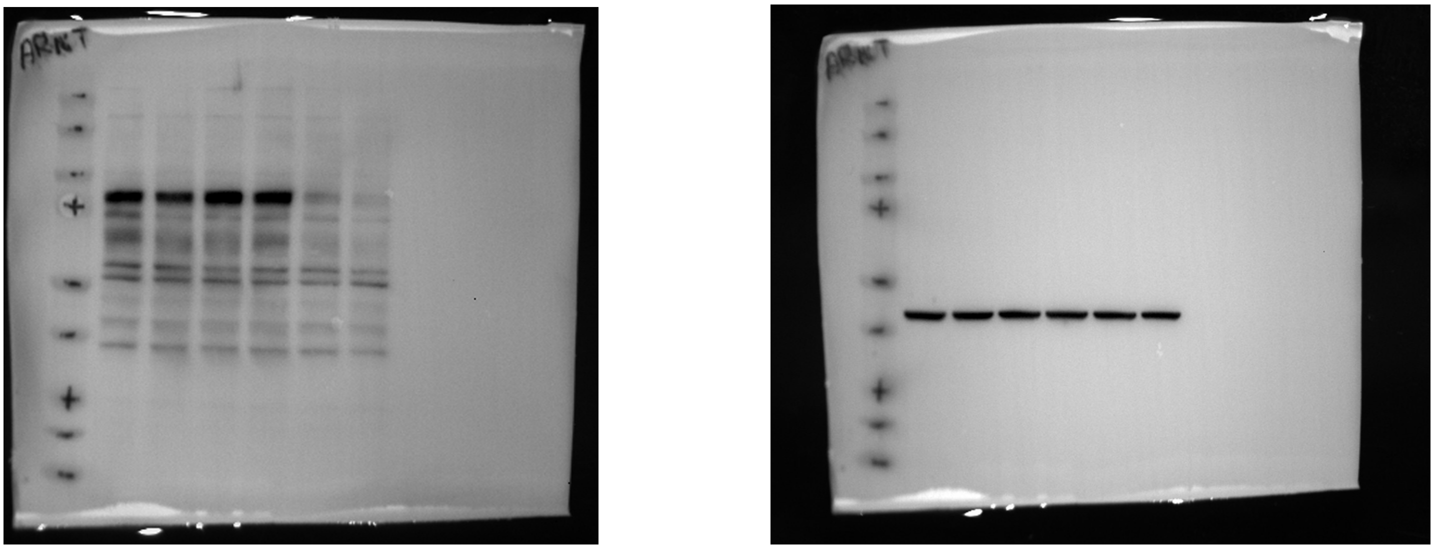

Figure 5C

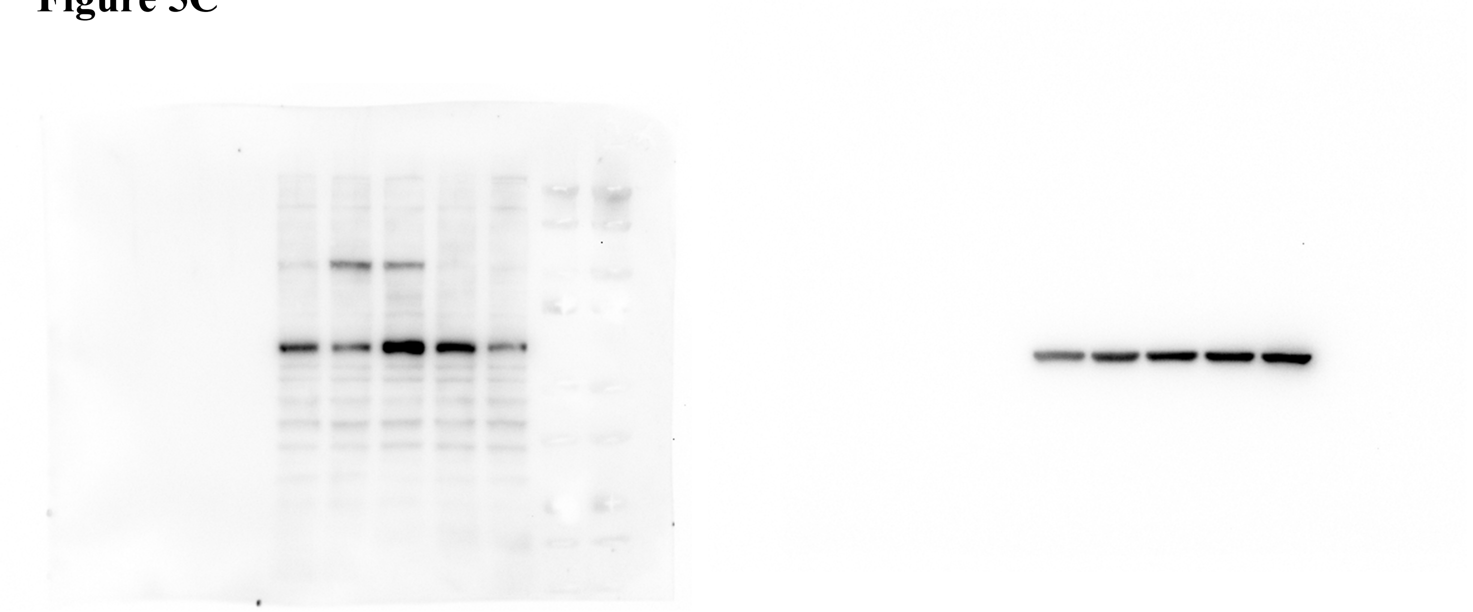

Figure 5C (overlay with ladder)

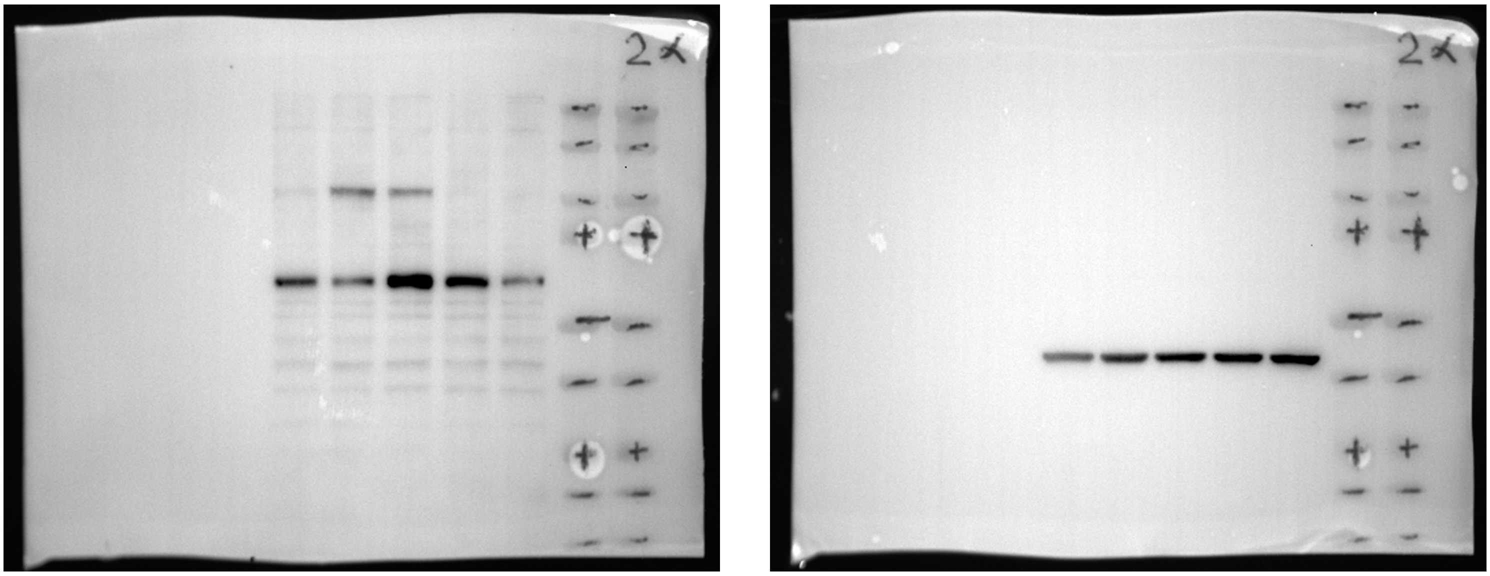

Figure S1B

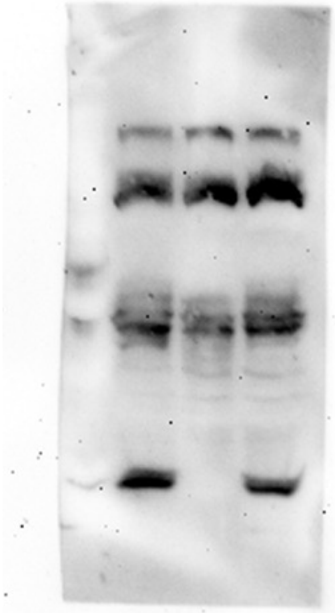

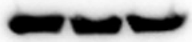

Figure S1B (overlay with ladder)

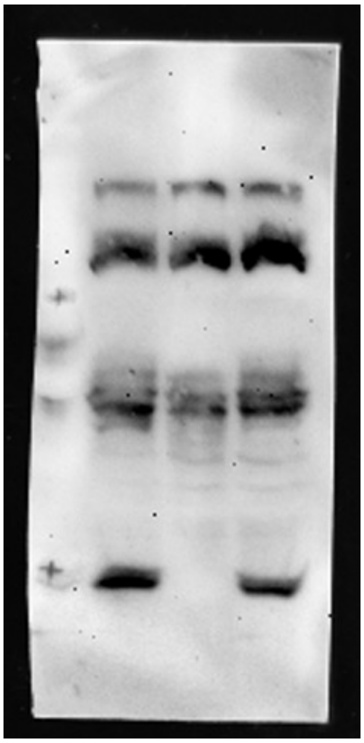

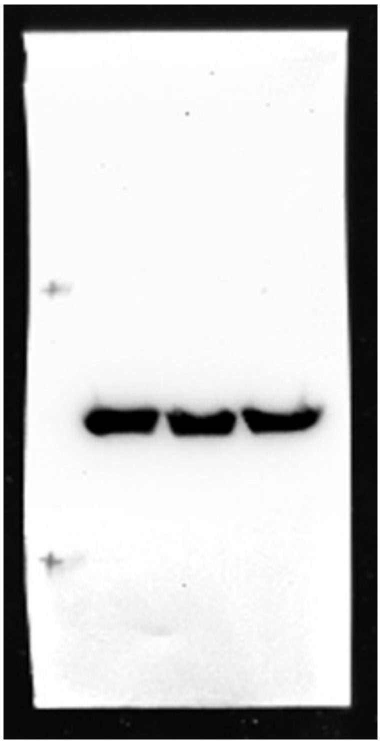

Figure S2B

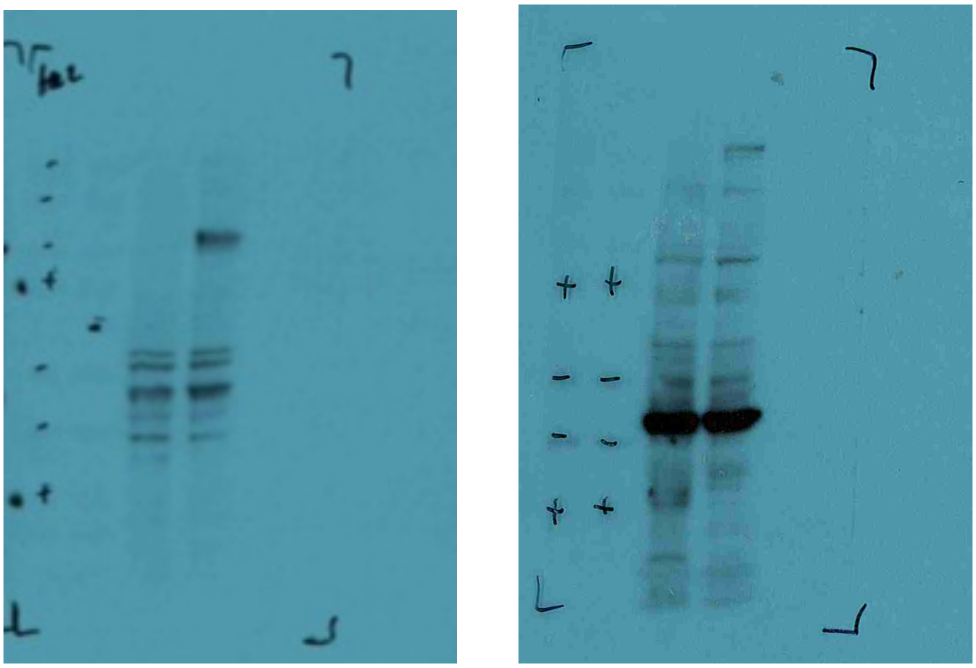

Figure 6A/left

HIF1a

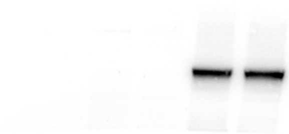

Elongin B (TCEB2)

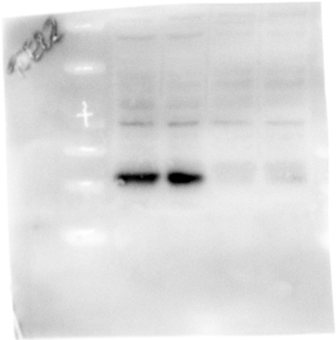

B-actin

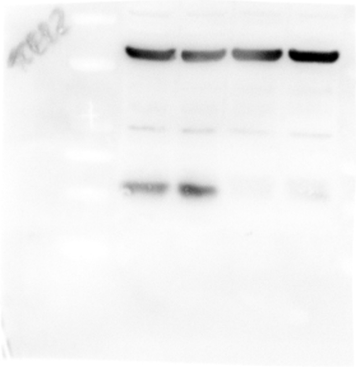

Figure 6A/left (overlay with ladder)

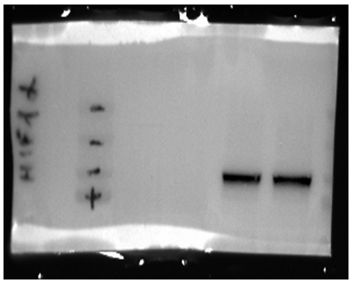

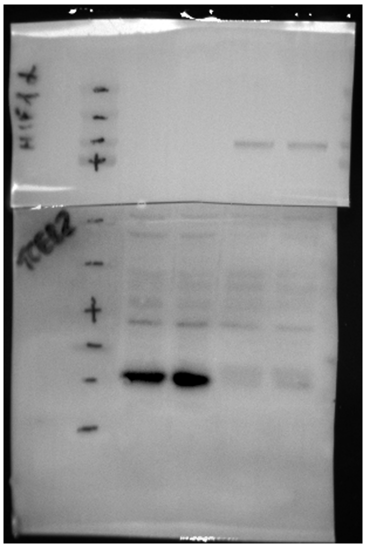

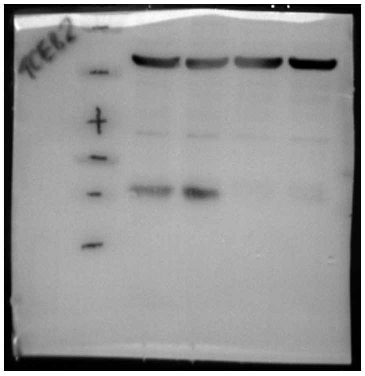

Figure 6A/right

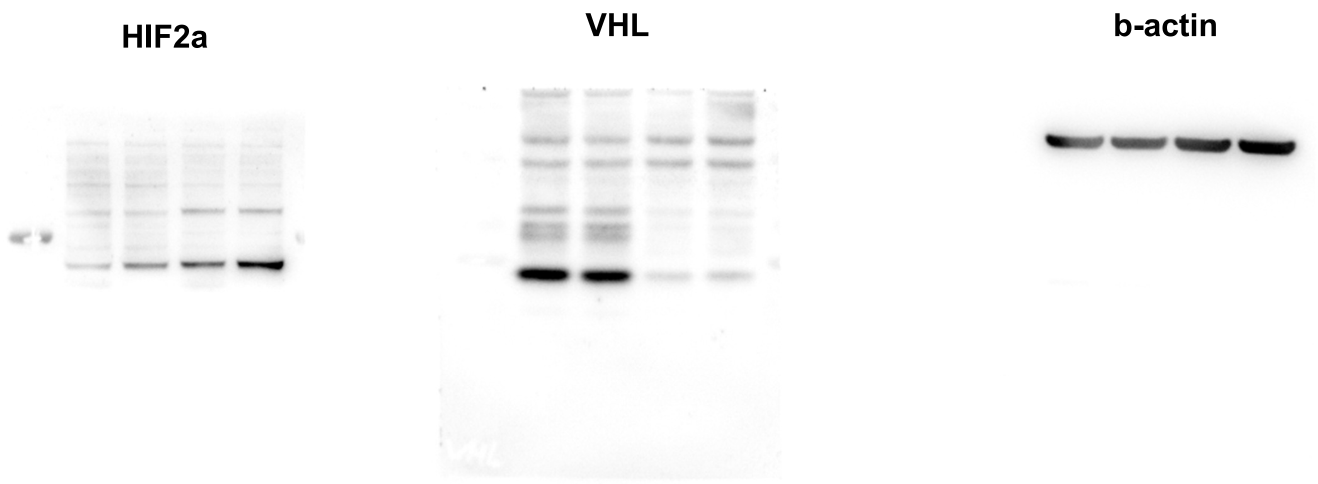

Figure 6A/right (overlay with ladder)

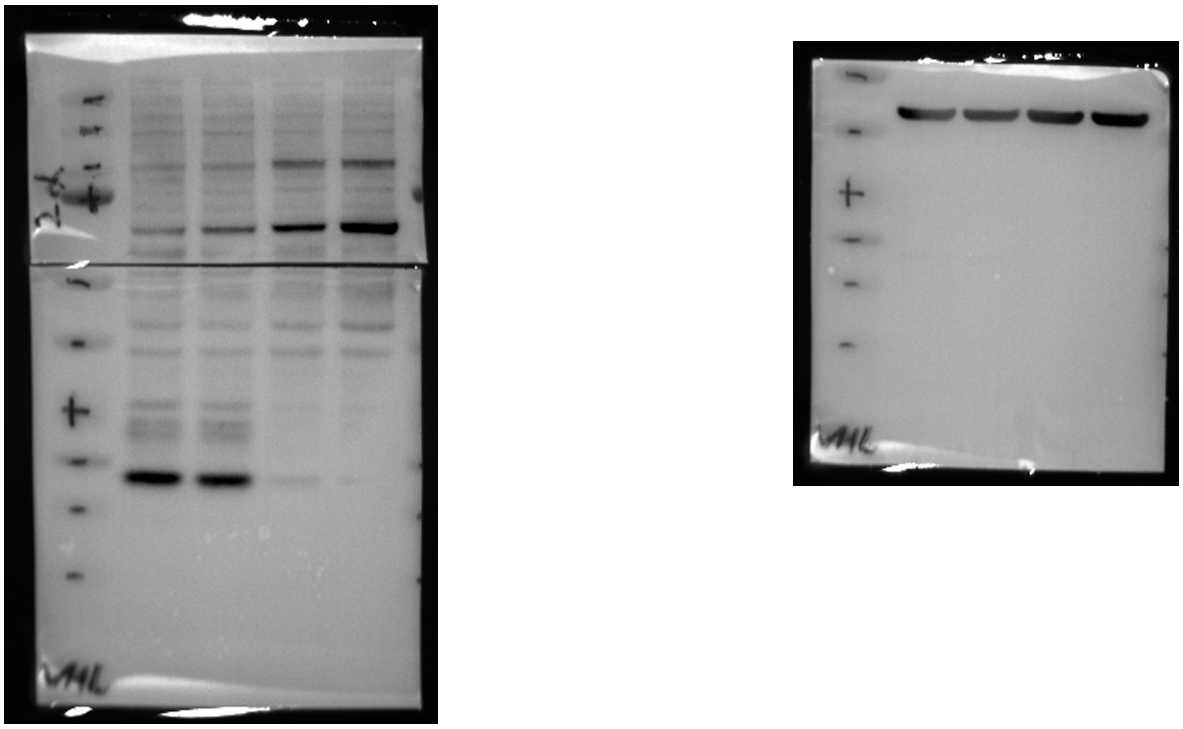

Supplement: Supplementary file 1 — Supplementary Information. [file 41598_2025_95644_MOESM1_ESM.pdf]
