# Supplementary material for: CRISPR-Cas9 genetic screens reveal regulation of TMPRSS2 by the Elongin BC-VHL complex
Source: Sci Rep. 2025 Apr 7;15:11907. doi: 10.1038/s41598-025-95644-0 (PMC11976923; doi:10.1038/s41598-025-95644-0)
Supplement: Supplementary file 2 — Supplementary Information. [file 41598_2025_95644_MOESM2_ESM.pdf]

Supplementary table 1. List of sgRNA sequences from the EMTR CRISPR library

guide_ID	sgRNA
A1CF_1	AGTTATGTTAGGTATACCCG
A1CF_2	ATGACTCTCATACTCCACGA
A1CF_3	GGTGCAGCATCCCAACCAGG
A1CF_4	TGCGCTGGACCAGTGCGCGG
A1CF_5	ACAGGAAGAATTCAGTTATG
ABTB1_1	GCAGCTGGCGAACAGGTCGC
ABTB1_2	GCAGGGCATCCACAGTGACG
ABTB1_3	TGTAATCGCGTAGAGCCCGG
ABTB1_4	TGTGTACAGGTACTGCAGCA
ABTB1_5	CGCACTTACTCGGAGCTCGG
ACIN1_1	AAGGAGAAAGACGATCATCT
ACIN1_2	AGGTCTGTGAGCCAAAGGAG
ACIN1_3	GACGAGATGATCCATCCTGA
ACIN1_4	GAGGAATTAGCACTGGCCAA
ACIN1_5	CCTGGTCAAGCGGCTCAAAG
ACTA1_1	CTTCGAGAACGAGATGGCGA
ACTA1_2	GGTGAAAGCCGGCTTCGCCG
ACTA1_3	GTGTCTAGAAGCATTTGCGG
ACTA1_4	TGCCAACAACGTCATGTCGG
ACTA1_5	CTTCTACAACGAGCTTCGCG
ACTB_1	CAGTCTCCACTCACCCAGGA
ACTB_2	GCCGCGCTCGTCGTCGACAA
ACTB_3	GCCGTTGTCGACGACGAGCG
ACTB_4	AAGAGCTACGAGCTGCCTGA
ACTB_5	CGTACAGGGATAGCACAGCC
ACTL6A_1	ACTGCAATTCCAGTCCACGA
ACTL6A_2	CCGGCAGCAGCCATGAGCGG
ACTL6A_3	GGCGATAAAGGCAAACAAGG
ACTL6A_4	TCTCAGGAAGCTGTTCGTGA
ACTL6A_5	ACCACCATACCAATAGCTGT
ACTL6B_1	ATGACCTCCGCTCCATCCCG
ACTL6B_2	GAGACTGGAAGGCTCACCTT
ACTL6B_3	GCACCCAGTGCTCATGTCCG
ACTL6B_4	GCAGCGGGCACTATGAGCGG
ACTL6B_5	AGTGGAACACACGGGCCAAG
ACTR3B_1	AGGACATGACTCCAAAGACG
ACTR3B_2	CTCAGCGAGGAGCTCAGCGG
ACTR3B_3	GAGAGAGGGACTTACAGTCG
ACTR3B_4	TAGAATGTCGTACTCTCAGG
ACTR3B_5	AGGTAGTTGACCAAGCTCAA
ACTR5_1	AGGAGCTCAATGCCCGGCGG
ACTR5_2	GAGCGCAGCATCCAGCGCAG
ACTR5_3	TGAGCACAGCTACATCGCTG
ACTR5_4	TGAGTGCTACGGGATTCCCA
ACTR5_5	CGGGACACGCCCAGCCAGCG

ACTR6_1	AAGAACATTGTCTTGACAGG
ACTR6_2	AGCTCCATTATCCAGCACTA
ACTR6_3	TAAACCCGATCCCTAAATCC
ACTR6_4	TGAGATGACGACCTTAGTGC
ACTR6_5	AGATGTATGCTATGTGTCTC
ACTR8_1	GATGGCTTTATCCAGGCCCA
ACTR8_2	GATGTACAGCTCCATCCTAG
ACTR8_3	GGACAGTTGGCTCCTAAGGG
ACTR8_4	GGTCAGTTAAATATTCACCC
ACTR8_5	AGCCAACTGTCCTTGTATAG
ADA_1	AGAGGGCGTGGTGTATGTGG
ADA_2	GAAGGCGGGCGTCTGGGCCA
ADA_3	GCTGCTGAACGTCATTGGCA
ADA_4	GTGGCGCATGCAGCACAGGA
ADA_5	CACAGACTGGTCCCCCAAGG
ADAR_1	AGACCAGACAGTCATAGCCA
ADAR_2	GCTAGAGGAAGCCAAAGCCA
ADAR_3	TACCTGGTTATCAGAAGCCA
ADAR_4	TCGCGGGCGCAATGAATCCG
ADAR_5	AGGCAATCAACACCTCTCTG
ADNP_1	AATCTCTAACGATAACCCAG
ADNP_2	AGTTGAGGAAGTGTTACCTG
ADNP_3	ATGGGCTTGGGTCAGAGCAG
ADNP_4	GATGGCCGCACACATGCGGA
ADNP_5	GTTGGTCAGTCAATGAGACT
ADNP2_1	AACAGTCCAAGCCCAGCCGC
ADNP2_2	ACAGCAGGAATAGGCACCTG
ADNP2_3	GCAGTGGATGCACTTGAAGG
ADNP2_4	TGGGATTCCAACCTACACGC
ADNP2_5	GCTTATGCGCTACCTCCATG
ADPRH_1	AGTGCAGCTGGAGATGCCCT
ADPRH_2	ATAGCAGACCACCCTTGCAG
ADPRH_3	GAAGGGAATCCTCCAGCCAT
ADPRH_4	TAGAATCACTGTCTCCACCA
ADPRH_5	AAGTGAGCATCGAGAGTGGT
ADPRHL2_1	GATGGCGCCATTGTAACCCA
ADPRHL2_2	GCAGCCTCGGAAGCGCGAGA
ADPRHL2_3	GGCTAGCAGGGACTGCACCA
ADPRHL2_4	TACAAGAAAGACCCTGACAG
ADPRHL2_5	ATGGCAATGGAGGTGCCATG
AEBP1_1	GAGATCGAGGAGTTCCTCGA
AEBP1_2	GCCCAAGGAGAAGCCACCTA
AEBP1_3	GGAGACCGAGCAACCCACAC
AEBP1_4	TAACTGGCAGAATCCAGGAG
AEBP1_5	CCAGACCTTTCATGGGAACG
AEBP2_1	AGCAGCGTAGTCTCCAGCGG
AEBP2_2	GCGGCGCTGCTGCTGAACGG
AEBP2_3	GTAGACAGCACAATTTCCAG

AEBP2_4	TAGACAGCACAATTTCCAGT
AEBP2_5	CTCCATCTACCAGTCAAAGT
AFF1_1	AGGCGGGAAGCAGACACCTG
AFF1_2	CATGGGCCGGACATAAGCCG
AFF1_3	GAAGATAAACAGCCGCCCGC
AFF1_4	GGGCCTCGGCCTGTTCCCAA
AFF1_5	AATCTGTCACCGAAGCACAG
AFF2_1	ACTTTGTAAGGTGAATCCTG
AFF2_2	CCTCCAGAAATAATGCACGG
AFF2_3	CCTGCAGTGCAAGCCAGCGG
AFF2_4	GTACTGGCAAGCCAGGCCAG
AFF2_5	GAGCTTCATGACCCACCAAG
AFF3_1	CAGATAGAAATGCATTACGG
AFF3_2	GCCGGAGCCTGCCAGAGCCA
AFF3_3	GCTTGAATAGCAGAAAGTGG
AFF3_4	GTAGTGCTACAGATAGACGA
AFF3_5	CAGCCTCTGATCATTCCCGG
AFF4_1	ATTGTGATAAGACAATGCCG
AFF4_2	CATGGGCCGCACATAGGCAG
AFF4_3	GTATGAATAGCCGTTAGAGG
AFF4_4	TCGGGAGATGCACTCTGGGA
AFF4_5	ACAATGCCGAGGAGTACACC
AGAP3_1	AAACACCACTGCATCCACCC
AGAP3_2	GATGCACGGACTCGAAGCGC
AGAP3_3	GCATCACCTGGAGCTCAGGG
AGAP3_4	GTCTAGCGGGAAGTCAGCCC
AGAP3_5	CTGCTGTCGTCGATAACCCG
AGO1_1	ACTGTCACAGCACTGCCCAT
AGO1_2	AGGGATCTGGCCGCTGACCA
AGO1_3	CGACGTGTACCACTACGAGG
AGO1_4	TGGGATGGAAGCGGGACCCT
AGO1_5	AAGAGAACTGTACCATACAC
AGO2_1	AACCTCTGACAGATTCCCAA
AGO2_2	GGTGAAGAAGGAGCGGCCCA
AGO2_3	TACACGATGCACTTTCAGGG
AGO2_4	TCAAGCCAGAGAAGTGCCCG
AGO2_5	AAGGTGGAGATAACGCACTG
AGO3_1	GATATGACCACCCTCTGGGA
AGO3_2	GTAGCACCACATCAACGGCA
AGO3_3	GTGCCCAGAAGACCTGGCTA
AGO3_4	TGAATGGAAATCGGCTCCGC
AGO3_5	ATGTCGTAGCACCACATCAA
AGO4_1	AAGGGAGGTAGTAGATACAA
AGO4_2	CAAGGTACTAACCTCATGGA
AGO4_3	GGCTGAAACAGGCTAGCCGG
AGO4_4	GGGTGGTAGTAACCTTCCGG
AGO4_5	AAAGTTGAGGTGACCCACTG
AHCTF1_1	ATTTACATCCAAGTCTGCGA

AHCTF1_2	CACAAGTAACAGCAATTCCG
AHCTF1_3	GTTGAAACAGCTGTTCCTGT
AHCTF1_4	TGTGGAACACACGCCAACAC
AHCTF1_5	CCACCCAAAATGGATTCCTG
AHR_1	CCTACGCCAGTCGCAAGCGG
AHR_2	GAAGGCCTGAACTTACAAGA
AHR_3	TATAACAGACTACTGTCTGG
AHR_4	TCAAGTCAAATCCTTCCAAG
AHR_5	ATCGCAAACAAAGCCAACTG
AHRR_1	AGACACCCACCTGTCTCCAA
AHRR_2	GAGGACGATGATCCCGCCGG
AHRR_3	GGAAGAAGCTCTTCACCCGG
AHRR_4	GGACAGCTGTCTCCGGGCGA
AHRR_5	AGGAAGGCCGAGTACTCGGT
AICDA_1	AAGTCATCAACCTCATACAG
AICDA_2	ACAGCCTCTTGATGAACCGG
AICDA_3	GGACCGCAAGGCTGAGCCCG
AICDA_4	GGAGGAAGAGCAATTCCACG
AICDA_5	ACCGCAAGGCTGAGCCCGAG
AIRE_1	GAAGCCTCTGGTTCGAGCCA
AIRE_2	GAGCAGCAGCGCCTTCCACT
AIRE_3	GCACCGCACGGAGATCGCGG
AIRE_4	GCTGTTCAAGGACTACAACC
AIRE_5	AGTTCGAAGACTCCGGCAGT
AKAP1_1	GACGCTCTGGGTGTAAGGCA
AKAP1_2	GAGACTGCAACAGCTATCCA
AKAP1_3	GCGGAAGCAGATCATTCAGG
AKAP1_4	GCTGCCTGATGGCATCACCG
AKAP1_5	ACAGACATGAGATTGCGACC
AKAP8_1	AAGCAGAGCCCACTTACCCT
AKAP8_2	ACAGGCGCAACCTACAGCTA
AKAP8_3	GAAGACATGGACCAGGGCTA
AKAP8_4	GAAGGGCTTACCTTGCCAGC
AKAP8_5	CAAGCTGCCCGACAAGACCG
AKNA_1	CCTGGCAGAAACCTTGCCAG
AKNA_2	GCTCCTAGAGGACTTCCGCC
AKNA_3	GCTCCTCACCAAGTACGCTG
AKNA_4	TCTTCTCCCGGACTCACCTG
AKNA_5	CCTGGGGCACCATTCCCGTG
AKNAD1_1	AACACGTTCAAATACGGCCA
AKNAD1_2	ATCCACCAAGAACTTCTCAC
AKNAD1_3	GCAGAACTTTCTGGCCACCA
AKNAD1_4	TAACTGGAGAACTCACAGGA
AKNAD1_5	AAGCACGAATCAACAATCGT
ALKBH1_1	AAACTGCGTTGGGTGACCGT
ALKBH1_2	ATAGGCTTGCCACTTGCTGA
ALKBH1_3	TGATTGACAAGACTTACCTC
ALKBH1_4	TTTCCGAAAGGCGTCCTCCC

ALKBH1_5	AGTTTCTCCAGTAAACTTCG
ALKBH2_1	AAAGTCTCTGCAGGCACCGA
ALKBH2_2	ACTCAGCAGGCCCTAGCTGG
ALKBH2_3	GCAGGCACCGAAGGAGACAG
ALKBH2_4	TCACGCTGTCTCCAAAGCCC
ALKBH2_5	AGGCAACGTATGGCGACGCT
ALKBH3_1	ACAGAGGACTGGCATCAGAG
ALKBH3_2	GGAAAGAGTGAAGATACCCT
ALKBH3_3	GTGATGATGAACCCTCACTA
ALKBH3_4	GTGGAGATGGCTCTTAGCAG
ALKBH3_5	ATTACTTACTCAATCACTCG
ALKBH8_1	AAGAGCCTGGTTGTTGCCAA
ALKBH8_2	ATCACCAGCAAACTCCGACG
ALKBH8_3	TGATAAAGAAGCCTCACGGC
ALKBH8_4	TTGGCAGTACCAGTCCGCAG
ALKBH8_5	AAAGATAAGCCATTATCTGG
ALX1_1	AGAATAAGGTGTCATACAGG
ALX1_2	AGCGCACGTGATGCTCGGCG
ALX1_3	TATCCAGCAGTAAGAAACGG
ALX1_4	TCAGAGAACAGCTTGCTCTG
ALX1_5	AGCATCACGTGCGCTTGGAG
ALX3_1	AAGCCAGCCACACTCCCATG
ALX3_2	CATGGAGTCAGGGAGTCCCG
ALX3_3	GCCGCGCGTTACCTTCCGCG
ALX3_4	GCGTTATGGGAAGATCCAGG
ALX3_5	GCTGCCCTTGGACTGCCGAG
ALX4_1	CCGAGCTGAGAACTACGCCC
ALX4_2	GCAACAAGGGCAAGAAGCGG
ALX4_3	GGAGCAGCCAGCCTCAGCCC
ALX4_4	GTAGTAGGCGTCCATGGCAG
ALX4_5	ACTACCCAGACGTGTATGCG
ANKAR_1	AATGAAAGGTGCAATGGCTG
ANKAR_2	AGACAACCCTCTTTCAACAG
ANKAR_3	ATGCTGAAAGCACTATGGCA
ANKAR_4	GAAGTGGCAGCAAGTAACAG
ANKAR_5	AGTGTGAAAGCTATAAACGA
ANKEF1_1	AAACCGCAAAGGCTTCCCGG
ANKEF1_2	ACTGGGCCATGAATTGTCAA
ANKEF1_3	TAGTGCCATGGACGTTGCAA
ANKEF1_4	TGGTGTACCAACCACATCAG
ANKEF1_5	CACCTTCATGAGAAAACCCG
ANKFY1_1	ACGAGCAGGAGCAGTACAGG
ANKFY1_2	GCCGGGCCAGACATGGCGGA
ANKFY1_3	GCTACATAAAGCCATCAAAG
ANKFY1_4	TTAACCAGATGCTAATCCTG
ANKFY1_5	AGCTTTGTGACTAACCAGCG
ANKHD1_1	TTCAGATTTGGCCTGGCACG
ANKHD1_2	AATAGAGACCAATCGGAACA

ANKHD1_3	AGGAGCAGACTTACGCACTG
ANKHD1_4	GCCAACGCATACACTTGCCA
ANKHD1_5	TGCAGAGATTCCAATCGTAG
ANKHD1-EIF4	GAAGAGAATTGGACGGCCGA
ANKHD1-EIF4	GACCGAAGAAGAGAATTGGA
ANKHD1-EIF4	TGGTGGCCTCTCCTAACCAG
ANKHD1-EIF4	TGGTTAGGAGAGGCCACCAT
ANKHD1-EIF4	AATCTCACTAGCCCTAAAAG
ANKIB1_1	GACCCTGTGGATATGCCCTG
ANKIB1_2	TACACAGGCTTTCATCCCTG
ANKIB1_3	TGCTGCATTAGACAAACGAG
ANKIB1_4	TTGGCAGCAGTTCTCCGGGT
ANKIB1_5	CAGTAAGACTAACGAAACAA
ANKRA2_1	AGCACACGGGCAAATAGCTG
ANKRA2_2	CCATGTACAGCATAAAGCAG
ANKRA2_3	GTAGCCCTAGGCTATAGAAG
ANKRA2_4	TGTGCTGCAGCCCACATCAG
ANKRA2_5	CATTAAAATAGAACATCCAC
ANKRD10_1	GCAGACATTGCACAAACCCA
ANKRD10_2	GGAACTAACGGAATTAGCAG
ANKRD10_3	GTGACGGACCATGTCGGCGG
ANKRD10_4	TCTGCCGTGCCACTCACGAA
ANKRD10_5	AACAATGGCATCTTAAATGG
ANKRD22_1	ACTTTGGACAAGTGTGGCGG
ANKRD22_2	CATGGGAATCCTATACTCTG
ANKRD22_3	GCACGGGCTTCCAAGAGCAG
ANKRD22_4	TAAGCAGAACAGGCATTAAG
ANKRD22_5	AGAATGACTTTGGACAAGTG
ANKRD32_1	ACAACCAGTGTACAACGTAG
ANKRD32_2	AGAGACCATGTATAGAACCC
ANKRD32_3	AGGAAAGTGGATACTAACCA
ANKRD32_4	GAAGCACATATATAGAGCTC
ANKRD32_5	CATGGCTATTAAGACAGATG
ANKRD33_1	GAAGATGGGATAGGACAGGC
ANKRD33_2	GGGTGGCCACGCACTCACAG
ANKRD33_3	GTGGCAGGCAGTGGTGACGA
ANKRD33_4	TGAGCAGGGCCACAACACTC
ANKRD33_5	CCGACAGCTTCGACACCGTG
ANKRD34B_1	ATTAATGAGAGCAACGACCG
ANKRD34B_2	GCTCCAGGATATTACACCAG
ANKRD34B_3	GGAGCAAGGAAACAACTTCA
ANKRD34B_4	TTAATGAGAGCAACGACCGT
ANKRD34B_5	TAATGAGAGCAACGACCGTG
ANKRD42_1	GAGGTGTTGCGCCATGCCCG
ANKRD42_2	GCCACATAGAGTGTTTGCAG
ANKRD42_3	TGAGGGAAGCACTGATGCCA
ANKRD42_4	TGGAGCAAATCTGACAGCCC
ANKRD42_5	ATCACACACGTAACAACGAG

ANKRD49_1	GGTACTCAAAGTCTTTGGGT
ANKRD49_2	TCAGGAGCTCATTGCACAGG
ANKRD49_3	TGAACCGTTACGTCAAACCA
ANKRD49_4	TGTTCAGGAGCTCATTGCAC
ANKRD49_5	AGAAAGGAGTCTCCGCACTG
ANKRD52_1	ACGTAGGCAGCAGCATGCAA
ANKRD52_2	ACGTTGAGGCTGCTCAACAG
ANKRD52_3	GAGGCAGCAGCACGATGAAG
ANKRD52_4	TTTGTCACAGACATTCAGGC
ANKRD52_5	CTGGACGTAAGGGACCACAA
ANKRD54_1	AAAGCTGAATATCCTGCAGG
ANKRD54_2	ACCGTGCACCTCCTTGCCCG
ANKRD54_3	ATCACCACACTGCTACGAGG
ANKRD54_4	GCTGGTTAGGATCAGCACCA
ANKRD54_5	AGGACGTGCAAGTAGCGCAG
ANKRD6_1	AGTGAGGAGGAGCCTAATGA
ANKRD6_2	ATCGCGGCGCTCATCCACGA
ANKRD6_3	GCTCATCAACAAGGGCGCCA
ANKRD6_4	TGTGGTCCAGATCTTGCTGA
ANKRD6_5	ACAAAATGAATACAAAGCTG
ANKRD61_1	AACCAGGCAACAGAACGCAC
ANKRD61_2	ACTGTGCTGTCTCTTCCACG
ANKRD61_3	CGGAGCCAAAGTCAACGCCC
ANKRD61_4	TCTGGCTGCCAAGTACCACA
ANKRD61_5	AGAAGACTGCACTACGATCG
ANKS3_1	GCTCGGGACACAGGTCAGCG
ANKS3_2	GTAGGAGGCATACATCAGCG
ANKS3_3	GTGGACGCGAGAGACCACAG
ANKS3_4	GTGGAGCCAATGCCAACGTG
ANKS3_5	AAGGGTGTCAGCATCCACGA
ANKZF1_1	AAGTCCAGGGCAGACAGGAG
ANKZF1_2	AGACCGGAGCATCCGCGCTG
ANKZF1_3	GATGAGGGCCTAGGACACAG
ANKZF1_4	GCCAAGCTGGGCTAAGGCGC
ANKZF1_5	ATACCTGAACAGGAAGTACG
ANP32A_1	GGGTCCGAATGACTTACCAG
ANP32A_2	TAAGCGATAACAGAGTCTCA
ANP32A_3	TCGAATGAAGGCAAACTCGA
ANP32A_4	TTGGCAAATGCTCACATCAG
ANP32A_5	AAGCGATAACAGAGTCTCAG
ANP32B_1	ACTGAAAGATATCAGCACCT
ANP32B_2	CGACGATGAAGTCAGTGAGG
ANP32B_3	GGAGCTGAGGAACCGGACCC
ANP32B_4	TCGGTCATAGCCATCCAAGT
ANP32B_5	AAGACAAGTTCTCGAACCTG
ANP32E_1	AAACGAGATGCTGAAGACGA
ANP32E_2	AATGAAGCTGGTCCACCGGA
ANP32E_3	GAGTCATCTCACCTCCTCCG

ANP32E_4	TCAGGAGGATAATGAAGCGC
ANP32E_5	AGTGATAATATAATTTCTGG
AP2A1_1	ACCCGGCCACCAGGATGCGG
AP2A1_2	ACGCGCCGTCCACTCGCCCA
AP2A1_3	CCAAGGGCGATGGGATGCGG
AP2A1_4	TGCCCTAGGAGACAAAGCCT
AP2A1_5	ACTACTTCGTCCCAGCACCC
APBB1_1	AGGCTGGGTAGAGATGACCG
APBB1_2	AGGGCCAGAACCAGCTCCGG
APBB1_3	GACTGAAGGAACCTGAGGAG
APBB1_4	TCCCAACAGGGACCACCCAG
APBB1_5	AGCTGGGCTACCTTATCCCA
APEX1_1	CAAGGAAGGGTACAGTGGCG
APEX1_2	TAGGACAGTGATCACTGCCG
APEX1_3	TATGTACCTAATGCAGGCCG
APEX1_4	TCGGACAAGGAAGGGTACAG
APEX1_5	ACAGCATATGTACCTAATGC
APOBEC1_1	CAGGAGAAGAATCGAACCCT
APOBEC1_2	TACAACATCATCCACAGAGG
APOBEC1_3	TAGAAGACGTCAAACTCCCA
APOBEC1_4	TCAGGAGAAGAATCGAACCC
APOBEC1_5	ATGGATCAACAAAATCGGCA
APOBEC2_1	CCTGTGACAATCTCAAAGGG
APOBEC2_2	GGTCAGCACACGCTGCACAG
APOBEC2_3	GTGCTTCAACCACATAGCAG
APOBEC2_4	GTGGTTGAAGCACAGGGCAA
APOBEC2_5	GGCATCTCGGGGATACCTAG
APOBEC3A_1	GGCGCTCCACTTCGTAGCAC
APOBEC3A_2	GTCAAGATGGACCAGCACAG
APOBEC3A_3	GTGCTGGTCCATCTTGACCG
APOBEC3A_4	TGGAAGCCAGCCCAGCATCC
APOBEC3A_5	AGAAGCGCAGCTCCGCATGG
APOBEC3B_1	AGTAGGTCTGGCGCCGTCGA
APOBEC3B_2	GACCTACTTGTGCTATGAGG
APOBEC3B_3	GTCCTGATGGACCAGCACAT
APOBEC3B_4	GTGCTGGTCCATCAGGACCC
APOBEC3B_5	ATAGTCCATGATCGTCACGC
APOBEC3C_1	AACTTGGCTGTGCTTCACCG
APOBEC3C_2	ATACTGGAAGTAGTAGAGGC
APOBEC3C_3	GATACTGGAAGTAGTAGAGG
APOBEC3C_4	GCGGCGCTTTATACCTTCCA
APOBEC3C_5	AATGGGTCTCAGAATCCACC
APOBEC3D_1	ACCGGGATAGAGATTGGCGG
APOBEC3D_2	ACGTCAGTCGAATCACAGGC
APOBEC3D_3	TCACCACACAGGGCAGGCAG
APOBEC3D_4	TCAGTCGAATCACAGGCAGG
APOBEC3D_5	CCGGGATAGAGATTGGCGGT
APOBEC3F_1	ACCTACGCAAAGCCTATGGT

APOBEC3F_2	ATTCCTGGACAGCAAGCTGC
APOBEC3F_3	TCCGACCATAGGCTTTGCGT
APOBEC3F_4	TGCAGGAGATTCTCGAGTGA
APOBEC3F_5	AAGATCTTTGCGTCCAAACG
APOBEC3G_1	AAAGAGAGCTATTTGAGCCT
APOBEC3G_2	ATCAAGGAAGATGTCAGGAG
APOBEC3G_3	GTCCTGCTGAACCAGCGCAG
APOBEC3G_4	TATGGCCACGTTCCTGGCCG
APOBEC3G_5	CCCCTGCACAAAGTGTACAA
APOBEC3H_1	AAGGCCTTACTACCCGAGGA
APOBEC3H_2	ACAGTCGAGCCATAAAGCGA
APOBEC3H_3	GATGAAGTCAACCAGCTCCC
APOBEC3H_4	TCAGAAGGCCTTACTACCCG
APOBEC3H_5	AATGGCTCCACGCCCACGAG
APOBEC4_1	ACAACAGGAGCCCTGAGGTC
APOBEC4_2	ATCTCCACTGACCTTCCAGT
APOBEC4_3	GACCTTGGAAGGCTTCCCAC
APOBEC4_4	TATCTCCACTGACCTTCCAG
APOBEC4_5	AGTGGTTAGCTTCATTACAA
AR_1	AGAGAGACAGCTTGTACACG
AR_2	CAGCAGGAAGCAGTATCCGA
AR_3	GGTGAAGGATCGCCAGCCCA
AR_4	TCATGCACAGGAATTCCTGG
AR_5	AGGGTACCACACATCAGGTG
ARGFX_1	AGAGATACTGATACAGACAG
ARGFX_2	AGAGTACAAAGCAGGAACCG
ARGFX_3	GCAGGAGATAGGCCTACAGC
ARGFX_4	TTGATGAAAGGGTCTGGCTG
ARGFX_5	ACTGGTAGAACTCTCAGTGA
ARHGAP35_1	AGAAACCAAAGCCCAAACCC
ARHGAP35_2	AGACTTTACGGTGAATACCG
ARHGAP35_3	AGAGCGGTGATCCTGCCCGG
ARHGAP35_4	GAAGTTGCATGCCCTTAAGG
ARHGAP35_5	AAATGTATGAGCTTTCCCTG
ARID1A_1	AACCTCACGGAGCCGCCCGG
ARID1A_2	AATACTCACAGGCAAGCTGG
ARID1A_3	GCAGCGCTTCCCTCCACCGC
ARID1A_4	TTTGTAGCCATCCAGTCCAA
ARID1A_5	CACATCCCTGACCCAACCTG
ARID1B_1	ATGGCCCTCCAGGCCCACAG
ARID1B_2	CTTGCCCAAGCCCATGCCCG
ARID1B_3	GGACGGCAGATACTGCGCCT
ARID1B_4	TCATCTCTCCAGCATCCCGG
ARID1B_5	AAGTTGCTTCCGTTCCCGTG
ARID2_1	AAATAATGGCAAACTCGACG
ARID2_2	ATTGCACCAATAGGAAGCTG
ARID2_3	TACTTTAGGCGGATTCGCGA
ARID2_4	TTTACTACTTGCTAATGCCG

ARID2_5	AGAAGTTGTTACATACTGTG
ARID3A_1	CCCTGGGAACGCCATCGCCG
ARID3A_2	GCTGTGGCGTGAGATCACCA
ARID3A_3	TCACAGCTCTACGAACTCGA
ARID3A_4	TGAGGACATGGCCTCCGACG
ARID3A_5	AACTCACATGTCCTCGTCGG
ARID3B_1	AAACACTTGGGCCACTGCTG
ARID3B_2	AATTGATGGCAACCGCAGGG
ARID3B_3	GAAGATCAGGATCAACGGCA
ARID3B_4	TCATCAACAAGAAGATCTGG
ARID3B_5	GCTGTGGCGGAAAGGAGAGT
ARID3C_1	CAGGCAGCTCGGCTGGCCCA
ARID3C_2	CATGGCCCTAGAGATCAACG
ARID3C_3	GTGTGGCGGGAAGTCACGCG
ARID3C_4	TAGCGGTGTAAGCCTGGCGA
ARID3C_5	CCAGGCGAAACAGAGCGTAC
ARID4A_1	ATTGCAAAGAAGACGAACAG
ARID4A_2	GATGCTAGTTGGTATACCGT
ARID4A_3	GATGTCAGTGCCAAGTACCG
ARID4A_4	TGTAAGTGTGGTGTCTGCAA
ARID4A_5	AAGAAAATGAGCTACTACTG
ARID4B_1	AAAGATTCTGATGTCGAAGG
ARID4B_2	GATGATGAAGACTCTTCCTC
ARID4B_3	GATGTGAGTGCTAAATACAG
ARID4B_4	TGGTCCCAGAGTCATCATGA
ARID4B_5	ATAAACTAACAGATGCGAGT
ARID5A_1	GAAACAGTACAAGATGGCTA
ARID5A_2	GAACGTGTACGACGAGCTGG
ARID5A_3	GCGACACACGCCCATCGAGA
ARID5A_4	GTGGAAGATCTACAAAGCAG
ARID5A_5	CCCCGCCGTACCTCTCGTAG
ARID5B_1	ACAGGTTTACCACCTCGCCA
ARID5B_2	AGGCAGTCAACCCTAAACAG
ARID5B_3	GATGGAGCCCAACTCACTCC
ARID5B_4	GATTCCAACAACAATTCCGA
ARID5B_5	GCAGACCCCAAAGGTCCTTG
ARIH2_1	ACAGTGCAATCGGTGCAACG
ARIH2_2	ACTGCATACACACTGCACAG
ARIH2_3	ATATCTCTGAAACTTGCCAG
ARIH2_4	TCAGTGGACATGAATAGCCA
ARIH2_5	CCTGTACCCGAATAACCATG
ARMCX3_1	AAAGTAGGCTGGGTGACCGC
ARMCX3_2	TCAGTCCCAATCCGAGCCCA
ARMCX3_3	TGAAGCTGGAACCAGAGCTA
ARMCX3_4	TGGAACCAGAGCTAGGGCCA
ARMCX3_5	ATCAGACCAGTCATTATACC
ARNT_1	ATCACAGTGAAATTGAACGG
ARNT_2	GAGCTCCAGCTCCTCACCTG

ARNT_3	GTGGAGGAGCCATTGTCCAG
ARNT_4	GTTGGCAGTAGTCGCCGCCA
ARNT_5	AGATGTCTAACGATAAGGAG
ARNT2_1	AAGGGCAGCAGTCATCCATG
ARNT2_2	ACAGGGAACAAGTCCACCGA
ARNT2_3	CCAGGCCGCTACTCACCCGG
ARNT2_4	CGGACAGGTGAGGATGGCGG
ARNT2_5	AGGAGAAGCCCAATATGCTG
ARNTL_1	ACAAAGATGACCCTCATGGA
ARNTL_2	GCATCTATGAGCCGCTCCCG
ARNTL_3	GCGGTTGCAATCCACACCAC
ARNTL_4	TTGTCGTAGGATGTGACCGA
ARNTL_5	CTGGACATTGCGTTGCATGT
ARNTL2_1	AAACCACAGGAGCAATGCAC
ARNTL2_2	AGTGGCTCCTGCGATGGCGG
ARNTL2_3	ATAGAAATATCAGCCTCCAG
ARNTL2_4	GTTCACAGTAATCTCCACGC
ARNTL2_5	ATAGCCAAACTGAAAAGCGG
ARRB1_1	CCTGGGCCTGACCTTTCGCA
ARRB1_2	GAGTATCTCAAAGAGCGGAG
ARRB1_3	TCTCCACAGAGTGTTCAAGA
ARRB1_4	TGTGGACCACATCGACCTCG
ARRB1_5	AGGTTCAGTATGCCCCAGAG
ART1_1	GAAGGAGTAGCCCTTGATAG
ART1_2	GATTCAGCTGGACATGGCCC
ART1_3	GCAAAGACCAGACTGCAGAG
ART1_4	GTGGTGAGGGCCTTTCCAGA
ART1_5	AGAGGTCTCGTCGTGTGATG
ARX_1	CATGAGCAATCAGTACCAGG
ARX_2	CATGAGGCTGGACTTGACCG
ARX_3	TAGGCTCGGGAAGGCGGCGG
ARX_4	TGGCGGCCACAGGACAGCGC
ARX_5	ACTACCCGGACGTCTTCACC
AS3MT_1	ACTGGAATAGACATGACCAA
AS3MT_2	TAAACAACAAGTGCTTCAGG
AS3MT_3	TCGGCAGACCTCCAGACCAA
AS3MT_4	TTGGCAGTGACCAAACGTGG
AS3MT_5	AAAGTATCTTGACTATCACA
ASAP1_1	ACTTGACAAACGCGGTGCCA
ASAP1_2	GGATCCGGAAAGTATGGCAG
ASAP1_3	GGGCGAGTTGTAGTCCTCGG
ASAP1_4	TACAGCATGCATCAGCTCCA
ASAP1_5	AATTCAAGTCTATGCAGAAG
ASB1_1	ATGGATGCTGTTCTGCGCCA
ASB1_2	GGCGGAAGCATCCATGGCGG
ASB1_3	GGGCCGGGCAGACATCCTGA
ASB1_4	TGCAAGAGGAGAGCTACCGG
ASB1_5	CAGTGCAGGTCGTAATCTGA

ASB10_1	ATACCAGCGACCCAGAGCGA
ASB10_2	GCAGCCGCAGGACTTCCGTG
ASB10_3	GTGGAGCTGCTCCTGTCCTG
ASB10_4	TCTCCACCAGCCTGGCACAG
ASB10_5	AGATCTGCTTCTAAGACGGG
ASB12_1	ACATGGGAGGAATATCCAGC
ASB12_2	AGGTAGCTAATCAACATGGG
ASB12_3	GGAGGAGGACACTGACACAG
ASB12_4	TAAGAAGCAGCCAAGCGCAA
ASB12_5	AGACTGCGGCCAAATAGAGG
ASB15_1	AAAGCAAGCACATCACCACC
ASB15_2	TAAACGACGAGGTAATGCTG
ASB15_3	TAGAGGAAGCTTCTCCACTG
ASB15_4	TCATTTACCAATCAGAAGGG
ASB15_5	CCCAGAGCTATGACGATGAG
ASB2_1	AGACGCGGTGGCCTCAGCAG
ASB2_2	CGGGCCGGCGAACTCTCAGG
ASB2_3	GTGACAGGAGACAGTCCAGG
ASB2_4	TCTCGCAGAGCCCAACAAGG
ASB2_5	AGACACAGACTCGTGCAGAG
ASB3_1	ATTGCCTAGAAATATTACTC
ASB3_2	TATCAGCAACATCGACACTT
ASB3_3	TCTTGAGCAGCAATGAACAA
ASB3_4	TTAAGGCTGTTGCTTCAACA
ASB4_1	AAGTGGGCAGCCGTGTGCAA
ASB4_2	GCACCAGAAGACATTCCACA
ASB4_3	TAAGGAGCAGGAGTACAGCA
ASB4_4	TTACCTCCAAGTCATCATCG
ASB4_5	GAAGACCAACAACCAAGATG
ASB5_1	AAAGAAGCTTCCAGATGCAA
ASB5_2	CATAGTGAAAGGCAACCGCA
ASB5_3	GTAGGTGCTGACGTACAGAA
ASB5_4	TATGGAGTAACCCAAGGACA
ASB5_5	CACATGCCACGTGATCTCCA
ASB6_1	AAACTTCGATATCCACCCTG
ASB6_2	AAGGATTCGGCTCTCCTCGC
ASB6_3	AATGCCGCTGACAAGCATGG
ASB6_4	GCGGAACCAGCCGGACATGG
ASB6_5	CCCATGCCGCAAGAGAACGT
ASB8_1	AATGTAGAGGACCTCATCAG
ASB8_2	ACCATACAGGCACAGTGCAA
ASB8_3	TGACTAGCAGAGCCACCAGG
ASB8_4	TGTAGAGGACCTCATCAGAG
ASB8_5	AATTGTTCGGATTAAGCGCT
ASB9_1	CATGGATGGGAGCAAGCCCG
ASB9_2	CTGACACAAGCATTAAACAG
ASB9_3	GAGAGTTGACACACTCCACG
ASB9_4	TCAGAGACAGCTGATGTCCG

ASB9_5	AATAGAGTGGAGTGCCCAGG
ASCC3_1	CAGCCTGGGAATCATACACA
ASCC3_2	CTTCAAACTTATATCAGCCG
ASCC3_3	GATGCAGCAGTTGAATAACA
ASCC3_4	GTACGAGAATTATTCCCAGA
ASCC3_5	AGCAGACGTCTAGAGCCACT
ASCL1_1	AAGATGGAGAGCGGCGGCGC
ASCL1_2	GCAAAGAAACAGGCTGCGGG
ASCL1_3	GTGGCAAAGAAACAGGCTGC
ASCL1_4	TCGGCCGCCGGTCTCAGCTG
ASCL1_5	AAACGCCGGCTCAACTTCAG
ASCL2_1	AGAGACCGGAGGCGGCGCAG
ASCL2_2	GATGCAGGCGCGATGGACGG
ASCL2_3	GCTGCAGCGCAACAGTTCCG
ASCL2_4	TGGACGGCGGCACACTGCCC
ASCL2_5	CAGCGCGCGGATGTACTCCA
ASCL3_1	AGATAGAAGGACCTGGTCAG
ASCL3_2	GAGGAAATGATGGACAACAG
ASCL3_3	GCTCCGCCATCATCTGCCAG
ASCL3_4	TGAACATAGGGTCAGCATGG
ASCL3_5	AGAGTAAGGGGATGACACCG
ASCL4_1	GAAGGCGCTGTCCAGCGGCA
ASCL4_2	GACGCAGGGCGACCCTGAGG
ASCL4_3	GCGGAGGAAGGCGGGCTCGA
ASCL4_4	GGCAAGGCCAGCCGTTCCGC
ASCL4_5	AGACAAGCGCCTCAGCAAAG
ASF1A_1	ACAAACATATGCCTTCCTGC
ASF1A_2	CAGGTCCTCGATGCACTCGA
ASF1A_3	TACAAACATATGCCTTCCTG
ASF1A_4	TTAGTGGGTCCTGTTCCCGC
ASF1A_5	CTAATTACTTGTACCTATCG
ASF1B_1	GAAGCTGATCTCGAACCGGA
ASF1B_2	GAGGACCACAGTCACACCCA
ASF1B_3	GCGAGGCGAGGCGATGGCCA
ASF1B_4	TATGGAAGCGGGTCACCCGG
ASF1B_5	ATCCCAGAGACTGATGCCGT
ASH1L_1	AAAGCAGAAGCCATTACCAG
ASH1L_2	CCTCTGATAAACCCTCCCAG
ASH1L_3	GGATTGGGTTCTGATTCCGA
ASH1L_4	TAGTTGGCAGAAGATCTCGG
ASH1L_5	CCTATGACCAACGTTCAAGT
ASH2L_1	AACTTGGTCGATGTAAGCGG
ASH2L_2	GAGCGGTCGCAAATGCAACA
ASH2L_3	GGTGAGGTAGAGCTGCAATG
ASH2L_4	TGCAACGTCTGCCATCACAG
ASH2L_5	GCTCTACCTCACCCAACTGT
ASXL1_1	ATAGCATTGAGGCATGCGAG
ASXL1_2	GAAGCTCTGTAGCTGTCCCT

ASXL1_3	TGGATGGCGAGACCACTGCA
ASXL1_4	TGTCCGCCTCACCAGGCGCG
ASXL1_5	ATCTGACGTACACTTTCCAG
ASXL2_1	ATCACAGAAGCACAGCAAGA
ASXL2_2	ATGCTTCACACAAACTCCAG
ASXL2_3	TAGGAAGAAGGGCAGGACCT
ASXL2_4	TTTCAGAAAGATGTGCCGGA
ASXL2_5	CCCAACTGGTCAAAGCACAG
ASXL3_1	ATTCAGACAGGCTAATGGAG
ASXL3_2	CTTGAAATGGACCAAAGCTG
ASXL3_3	GGATGGTACAGATATGGCCG
ASXL3_4	TGGAAACAGCGACTGGCAGA
ASXL3_5	AATGGTGTCAAAACAACACG
ASZ1_1	AACAAGGAGAGCAACAACCT
ASZ1_2	GACCAATGAAACATCTCCGA
ASZ1_3	TGGAGCAGAAGTTAATACCC
ASZ1_4	TTTGCAAGCTAACTTTCCAG
ASZ1_5	ACACTAGCAGCATACATAAG
ATAD2_1	AGGAAGTTGAAACCTACCAC
ATAD2_2	ATGAAGTCGAGTCACTACTG
ATAD2_3	CGCGGCGCAGAAGAAACCCG
ATAD2_4	TAGGCAGTTGGCCAGACAGC
ATAD2_5	AGATACCGATTATCTTCCGC
ATAD2B_1	AGAGCAGAGGACTTAGCTAG
ATAD2B_2	CATCTTACAGATCCATACCA
ATAD2B_3	GAAACTAGAATCTCAAGAGG
ATAD2B_4	GCTCTTCCGGGTGTTCACCA
ATAD2B_5	GATTCTGATAATTGACGAGG
ATAT1_1	ACTGGAAGAGTTCTCGCCCA
ATAT1_2	ACTGGGCAAGGCTTCTGCCA
ATAT1_3	TCTGGAGACCACAGTCCCAC
ATAT1_4	TGATGCACTAGTGATAGGAG
ATAT1_5	AGGGTCGGTCAATTGCCAGT
ATF1_1	ACTTGTGCCCAGCAATCAGG
ATF1_2	GAGAGTACTCACCTGTAAGA
ATF1_3	GGCGCCGTGCTAGGATCCCG
ATF1_4	TTGGCAAGTCCAGGCACAGA
ATF1_5	AAGTATCTGCTGTCCATCAG
ATF2_1	AAAGAGCAGGCTCTGTACTC
ATF2_2	ACTGTCAAAGGTCATGGTAG
ATF2_3	AGTTCGGTCAGCACATCCAA
ATF2_4	ATAGCCAGATTTCTTCTGCA
ATF2_5	ACATACCGGAGTTTCTGTAG
ATF3_1	AAAGTGCCGAAACAAGAAGA
ATF3_2	CCAGCGCAGAGGACATCCGG
ATF3_3	TCAAACACCAGTGACCCAGG
ATF3_4	TGAGCCCGGACAATACACGT
ATF3_5	GGCAGAAGCACTCACTTCCG

ATF4_1	AGATGACCTTCTGACCACGT
ATF4_2	AGTCCCTCCAACAACAGCAA
ATF4_3	CTAAGGCGGGCTCCTCCGAA
ATF4_4	GGTCAGAAGGTCATCTGGCA
ATF4_5	TAATAAGCAGCCCCCCCAGA
ATF5_1	AAGGACCTCATAGGGAGCCA
ATF5_2	GAAGGAACGGGCAGAGTCCG
ATF5_3	GAGAGACCAGAACAAGTCGG
ATF5_4	GGAGGGCGGGCTTCCAGTGG
ATF5_5	GCTCCCTATGAGGTCCTTGG
ATF6_1	ACACAGACAACTCTTCGCTT
ATF6_2	GAATAAGAGTCCACTGACCG
ATF6_3	TGAGCCCTGCAAATCAAAGG
ATF6_4	TTTGCCAATGGCATAAGCGT
ATF6_5	TTGTAGGACAGGTTTAGTCA
ATF6B_1	AGATCGGCAGGAGTTCCCAT
ATF6B_2	GAAGAGCTGCGTCTGCTCCT
ATF6B_3	GACGCGATGACTCGGAGCTG
ATF6B_4	TTGTCGGTGAAGAAACGCGT
ATF6B_5	ATGTTCTTCCGTCAACTCTG
ATF7_1	CAGCTGCAGAGCGAACACTG
ATF7_2	GAACACTGAGGCCATTGCTA
ATF7_3	TGAGCTGTGCTGAATCACAG
ATF7_4	TTGGCTCTGAGCTTTCCTTG
ATF7_5	AGCCCACCCCTAGTACTGGG
ATF7IP_1	ACAGCAAGTGTTGTTCCCAA
ATF7IP_2	ACTAGAGGTGGGATCACCAG
ATF7IP_3	ATCAGAGGCCAGTTCACCAG
ATF7IP_4	GCTAGAGGTGGGATCACCAG
ATF7IP_5	AAAAGATTGAATGTAACAAG
ATF7IP2_1	ATTGGGAGTAATGTTCCAAG
ATF7IP2_2	GAGTGCAGAAATAAGCATGA
ATF7IP2_3	TGAGATAAAGTACAGGCCAT
ATF7IP2_4	TTTCGAATGTGACTCCAGGG
ATF7IP2_5	ACTCTGTCTTCCCAATGCGT
ATM_1	AAAGTCAAACAGCATACTGC
ATM_2	GACACAATGCAACTTCCGTA
ATM_3	GATGGCAGATATCTGTCACC
ATM_4	TTGCTTGAGCAACTGTACCA
ATM_5	CCAAGGCTATTCAGTGTGCG
ATN1_1	AAAGCCGCCTACCACTCCAG
ATN1_2	CAAGAAGCGGGCCGACCTGG
ATN1_3	CCGGGAAGAACTGAGATCGA
ATN1_4	GGAGCGAGAACGTCTAGCGC
ATN1_5	GAGAAGGCTGAGTATACTTG
ATOH1_1	ACGGCTAGCAGCCAACGCCA
ATOH1_2	GAGCTGCTACAAACGCCCAG
ATOH1_3	GCTGTGCAAGCTGAAAGGCG

ATOH1_4	GTTGCGGGAGATGATGCGGC
ATOH1_5	CTCGCCTGCAAAGTTGCAGG
ATOH7_1	CCAGCCCGAGCCCTTCCAGA
ATOH7_2	CGCGGCCGAAGTGCTCACAG
ATOH7_3	GATGGCCCTGAGCTACATCA
ATOH7_4	TCTGACCCGGATCCTGGCCG
ATOH7_5	CAGAGCCATGATGTAGCTCA
ATOH8_1	AAGGTCAGCCAGCCGCGCCA
ATOH8_2	GAGGCTCCTGGCGAACGCCA
ATOH8_3	GGGCGCACAGTGGACTCCGG
ATOH8_4	GTTACAGGCGATCCTCAGGA
ATOH8_5	ACGAGTAGGAACTTTCCCCG
ATR_1	CTTGTGTAACAAATGACAGG
ATR_2	GAAATCAAGCAACATCACGG
ATR_3	GTGATGGAATATCACCCAAA
ATR_4	TGTTCAGAAACTGGACCTGG
ATR_5	CTGTGTGAGATGGTCAAGCA
ATRX_1	AACCAAAGAACCTTTAGTGC
ATRX_2	AAGGGTGTAGTCTTTACACG
ATRX_3	GTGAATCCGAAGATGAACAG
ATRX_4	TGAAGATCCATCCTCATCAG
ATRX_5	AATTAGTGCGGAATAAGAGT
ATXN3L_1	AACATCAGACCAAGATGAGG
ATXN3L_2	ATCGAGCCAAGAAATTTGCA
ATXN3L_3	TGTAGGTATGAACTGTGGCC
ATXN3L_4	TTTCGTGTAGGTATGAACTG
ATXN3L_5	CTTCGACACTGATGATCTGC
ATXN7_1	GAAGTGGGAGGAACGGCCAA
ATXN7_2	GAGCGGAAAGAATGTCGGAG
ATXN7_3	GTTAAGGCCAGGCTTGACTA
ATXN7_4	TAATCAGGTTGTCAAACCGC
ATXN7_5	CATACTCACATTCTACCATG
ATXN7L3_1	CAAGAAGAGAAAGTCAGACA
ATXN7L3_2	GCAGCCGAATCGCCAACCGC
ATXN7L3_3	TGTAGGGCCACTTACGCCGA
ATXN7L3_4	TTGGAGGAGCCTGAATCAGA
ATXN7L3_5	CACGGACCCTGATAGCATGA
AURKA_1	CTTCGAATGACAGTAAGACA
AURKA_2	GCTAGTTTACCAGGTGCCGA
AURKA_3	TCTGAGCTGATGCTCCACTC
AURKA_4	TTACCAGGTGCCGATGGCAG
AURKA_5	CCATATAGAAAATAATCCTG
AURKB_1	AGGCCACCATACCTCAGGGA
AURKB_2	GGGTGACAGGCTCTTTCCGG
AURKB_3	TGACGAGCAGCGAACAGCCA
AURKB_4	TGTGATGCACTCTCAAAGGG
AURKB_5	ATTCTAGAGTATGCCCCCCG
AURKC_1	ACTGTGGGAGGCGCTCTCAA

AURKC_2	AGATGAACAGCGCACAGCCA
AURKC_3	TCGGGAGTGGGCCTGAACCC
AURKC_4	TGGGAGGCGCTCTCAAAGGG
AURKC_5	ATTCTGGAATATGCTCCAAG
BABAM1_1	AGCGTGGGCAGCCGCAGCGA
BABAM1_2	GGCAGCTACAGAGCTCGCGG
BABAM1_3	GGTGAACGATGACACGGCCT
BABAM1_4	GTGCGGACCACATATGGCGG
BABAM1_5	CAAAAGCCACGAGTTTGCAC
BACH1_1	ACAGCACATCTTTCTTCCGC
BACH1_2	AGAATCGTAGGCCAGGCTGA
BACH1_3	GTGAGCCTGGCTACGCGCGA
BACH1_4	TATGGGCAAGGTTCTTGCTG
BACH1_5	AACTGACAGAGTCCGTACTG
BACH2_1	CAGGAGCCAAACAGTGACCG
BACH2_2	GCGCAGCAAGAACCGCATCG
BACH2_3	GGACTCATACACATACATGG
BACH2_4	TGACGCCAAGGACAGAGCGG
BACH2_5	AATTATGGACAGCCCCACGT
BAHD1_1	AGACCTGAGCACTTACAGGG
BAHD1_2	CAAGGAGCAAGGCTGCCCGC
BAHD1_3	CAATGGCAAGAACTATCCCA
BAHD1_4	CATCCGAAAGAGCTACCAGG
BAHD1_5	ACAGGCCATTGTAGTCGGCG
BANF1_1	AAGGCATCCGAAGCAGTCCC
BANF1_2	AGGCGTCGCACCACTCTCGA
BANF1_3	GCTTCGGATGCCTTCGAGAG
BANF1_4	GTCCCGGGACTGCTTGGCGT
BANF1_5	AGTCCTGGGCAAGAAGCTGG
BANF2_1	AATGAGCCACCTCTGAAACT
BANF2_2	ATGAAGCCGAGTTTCAGAGG
BANF2_3	GGAGAGGAAGGCTCTCAGCC
BANF2_4	TCAGAAGGAATTGTCCCAGC
BANF2_5	AGATGGACAACATGTCTCCC
BANP_1	ACACAGCCTGCACCTCGCGG
BANP_2	AGACGTGCAACAAAGTGCGA
BANP_3	GAAGACAGCCACCACGAGGA
BANP_4	GCAGCACGAGGCCTACCTGA
BANP_5	AGGCTGTGTCCAACCTCTCG
BAP1_1	ATGGGCCTTGGCCAACTCCG
BAP1_2	CAAGGTAGAGACCTTTCGCC
BAP1_3	GCGGGAAGATGAATAAGGGC
BAP1_4	TCGCATGAAGGACTTCACCA
BAP1_5	CACGGACGTATCATCCACCA
BARD1_1	AGCCGAGGAACCGGCAGCCG
BARD1_2	CAAGTCTTGTATCCAGGCCG
BARD1_3	GTCAAGCTGTTACTTTCCTA
BARD1_4	TGAGGACTGGAGATAACAGA

BARD1_5	ATGTCCAGTGTGTTACACCC
BARHL1_1	AAAGCCACGCAAGGCGCGCA
BARHL1_2	ACAGGTAGAGCGCCGCGCCG
BARHL1_3	GATGTCCCTGATCAGAAAGG
BARHL1_4	TGAGCGTGGACTCAGCTCCA
BARHL1_5	GCAGGTGGGAGTCCAAACCT
BARHL2_1	AAAGGCTGCAAACTTTGCGT
BARHL2_2	CAAAGTGCCTCCCTTACCTG
BARHL2_3	GCTCGGTGAGGCCAGGACCG
BARHL2_4	GTGGAAGCGGCAGACAGCGG
BARHL2_5	AGGCTGCAAACTTTGCGTCG
BARX1_1	ACGGAGCCACCCGGGCCCAA
BARX1_2	GCAGAAGTACCTTTCCACGC
BARX1_3	GGAGTCTCCCACCAAGCCCA
BARX1_4	TGCGATAGCGGTGCGGCCGG
BARX1_5	AACAGCCCAGCGGCGCCAGT
BARX2_1	AGAGGAGCGGATATGCCCGC
BARX2_2	GAGGGTATTGGCAACTCCTG
BARX2_3	GATCGACGAGATCCTCTCCA
BARX2_4	TTGGGTCGCACGACCAGCGA
BARX2_5	AAGATGGTGCGACTCCGGCG
BATF_1	AGGACTCTACCTGTTTGCCA
BATF_2	GAAGGCCGACACCCTGCACC
BATF_3	GCTGTCGGAGCTGTGAGGCA
BATF_4	GGCGCTGTACACCACCTCGG
BATF_5	ACAGAACGCGGCTCTACGCA
BATF2_1	CCGGCAGAAGCACACAGACA
BATF2_2	GAGCTGGAGCATCCCACCAG
BATF2_3	GGTGGGATGCTCCAGCTCGA
BATF2_4	TTAGAAGTGGACTTGAGCAG
BATF2_5	AAAAAGACAACCTCGCCCTG
BATF3_1	AGAGATCGGGAAGCTGACAG
BATF3_2	ATGATGACAGGAAGGTCCGA
BATF3_3	GGAGCTGAAGCACCTGACAG
BATF3_4	TCGGAAGAAGCAGACCCAGA
BATF3_5	AGCAAGAAAACACCATGCTG
BAZ1A_1	GCATCACCAAAGATTTCAGG
BAZ1A_2	GCATGGCCACAGTTACACCA
BAZ1A_3	TGTGAGACAGAAGCCGCCCG
BAZ1A_4	TTTCATGTAAGCGCGAACGA
BAZ1A_5	AGCAGGACTGAAGATAAATG
BAZ1B_1	ACCATCAGATTTCTTCTCAG
BAZ1B_2	CAACGGCTTCACCAGCGGGA
BAZ1B_3	GATCATGACCAAATATGCTG
BAZ1B_4	GCTAACACACAAGGAAGCCT
BAZ1B_5	AATTCATCTTCTACGACCCA
BAZ2A_1	AGGAGAGGGTTTCAGTCCTG
BAZ2A_2	CATAACACTAACCTTCGGGC

BAZ2A_3	GCAGTAGCATTATTGAGGGA
BAZ2A_4	GGTGTAACGATTCCACAGAA
BAZ2A_5	ACGGTACCCAGAGTCCCATG
BAZ2B_1	GCTAAGTGAAGCAACTCCAG
BAZ2B_2	TGAAGCCAGAGATGGACCGC
BAZ2B_3	TGGGAAGGCACTCGACACTG
BAZ2B_4	TTACAGGTGTCTGATTGCCA
BAZ2B_5	AGAACAAGTCCTGGAATACG
BBX_1	ACCTCGGCAAACTGAAACAA
BBX_2	CCTGACGTACAAGAGAGCGA
BBX_3	GAAATGCCTCAGCTTAACTT
BBX_4	TACCTCTGCAAACTGAAACA
BBX_5	AAAGCAAAAGAATCCGATGG
BCL11A_1	ACAGATGATGAACCAGACCA
BCL11A_2	CCTGAAGGGATACCAACCCG
BCL11A_3	TAAGCGCGGCCACCTGGCCG
BCL11A_4	TGGCATCCAGGTCACGCCAG
BCL11A_5	AGTGCAGAATATGCCCCGCA
BCL11B_1	CAGGAAAGTGTCCGAGCCGG
BCL11B_2	GCGGGTTGCCCTGTTTGCGG
BCL11B_3	GTGGTGGCGGAAGTCACCGT
BCL11B_4	TCAGGGTGAGGGTCAGACGG
BCL11B_5	AGCAAGTCGTGCGAGTTCTG
BCL2_1	ACCTGGATCCAGGATAACGG
BCL2_2	GAAGAGCTCCTCCACCACCG
BCL2_3	GGTGATGCAAGCTCCCACCA
BCL2_4	GTGGCCCAGATAGGCACCCA
BCL2_5	CTGACGCCCTTCACCGCGCG
BCL3_1	CGGTGCCGACATCGACGCAG
BCL3_2	GCCATGGCGGTCCAGCGCCA
BCL3_3	GCTGGTCAACCTCTTCCAGC
BCL3_4	GTAGTAAAGCGCCTCCGGCC
BCL3_5	CAGGTTCACCAGAGGAAACG
BCL6_1	AGATCCTGAGATCAACCCTG
BCL6_2	GAGTACTCAGATTCTAGCTG
BCL6_3	TGAGAAGAACATCACTGGCA
BCL6_4	TGGGTGAATAGATATTGCTG
BCL6_5	AAGTGATATGCACTACAGTG
BCL6B_1	GACGTGCTCTCTCTGCCCGG
BCL6B_2	GAGCGCCTGGGACTACCTGG
BCL6B_3	GTTGAGGTTGCCCAGCACGT
BCL6B_4	TAGTGGACGAGCCCGTTCAG
BCL6B_5	CACTGCACCAGCAGTCCTAG
BCL7A_1	ATGCATGTCCATCATCCCTG
BCL7A_2	GCAGAATTCACAGTCCTCGA
BCL7A_3	TCGTAGGGATGTGTCACCAA
BCL7A_4	TGTGCCCAGCGACGGCACCG
BCL7A_5	ATACTCACGTCATCAACCTT

BCL7B_1	AAGCTGATAGACGTCAGACA
BCL7B_2	ACACACCTCCGACTTCCGCA
BCL7B_3	CTGTGGGTGACACGTCCCTG
BCL7B_4	TGAACCTCCTACCCTCACCA
BCL7B_5	AACCCACTTAAATATCCTCA
BCL7C_1	CCATCTCCGGACCTTCTCGA
BCL7C_2	CTTGACGTTCCAGATCCCGG
BCL7C_3	TAAGATCCAGCAGGATGAGA
BCL7C_4	TTTGCAGGGAACCTTCCGAA
BCL7C_5	ATGAGAGGGCCACCCCCTCG
BCLAF1_1	GAAGCATACCCGTTTAGCAG
BCLAF1_2	GGAGGTAGATATCATCGAGG
BCLAF1_3	GGCGATCAATGTCATGCCGA
BCLAF1_4	TCAGAGAGGAAAGCCCACTT
BCLAF1_5	AGACGACCTTATGGGTACAG
BCOR_1	AAAGCCAAAGTCGGTCACCC
BCOR_2	ACTGACCTCACAGTAAGCAG
BCOR_3	ACTGGGCGATACCACAGCAG
BCOR_4	TGTGAACGTTCCCATACAGG
BCOR_5	AAAGAGAATCGCCAACTCAG
BCORL1_1	AAAGAGCACAATGGAGTCAG
BCORL1_2	GAAGTGGCAGCAATGCCCGG
BCORL1_3	TCTACAGCACCGCTCTACAG
BCORL1_4	TTTCCGTGACTTTATTCCTG
BCORL1_5	CTGGAACAAGCCCTGAATGG
BDP1_1	CTCAAGGGAGAAGACACGAG
BDP1_2	TCAATGGCATCAATCACCTC
BDP1_3	TCAGTAGCATCAATCACCTC
BDP1_4	TGAAAGAGAAATATCGCCAC
BDP1_5	AATGACCCCATATTTGAGCG
BHLHA15_1	GCAGATCCACAGCTTCCGAG
BHLHA15_2	GCAGCAAGGGCTCCGGGCGA
BHLHA15_3	GCGGCTGGAGAGCAACGAGA
BHLHA15_4	GTGACAGCAGCATCCAGCGG
BHLHA15_5	ACGGGTCCCTGCCGAACCCG
BHLHA9_1	AAGCTGGCGCCGCTGTCCGG
BHLHA9_2	ACCGCACTGGGCCGTGCCAG
BHLHA9_3	AGCGCGGATGGCCCATCCCG
BHLHA9_4	GGCCTAGCACAGGCCTCCGG
BHLHA9_5	AGCACCCCCGAATCGCCCCC
BHLHE22_1	AGGGACAGGTCCATGCCCGG
BHLHE22_2	GAAGAGGTCGTCCTCGCCGG
BHLHE22_3	TACGGCGAAAGCGCGAGCCG
BHLHE22_4	TCCAATGCCCACCTCCACGG
BHLHE22_5	AGCCGCTTCCAAGCGCTTGG
BHLHE23_1	ACAGTGTTTGCAAAGCGCGG
BHLHE23_2	ACTAAGCCACGGCTACGCGG
BHLHE23_3	GCACACAGTGGCCTGGCCGA

BHLHE23_4	GCGTACCTGGCACTAAGCCA
BHLHE23_5	AGGACGACGCCTTCGAGCAG
BHLHE40_1	AAAGCACCGGGACTGGAGCA
BHLHE40_2	AAAGCGGAGCGAGGACAGCA
BHLHE40_3	GGGTAGGAGATCCTTCAGCT
BHLHE40_4	TCAGTGCCAGTGCTATACCC
BHLHE40_5	CAAGTGTACAAGTCAAGACG
BHLHE41_1	ATGAAGCTGGATTCCCGCGG
BHLHE41_2	CAGCAGTTGAACATGGACGA
BHLHE41_3	GAAGTATCTGTACCCGGCGG
BHLHE41_4	GATCAACCACTTGCACGCCG
BHLHE41_5	AGGATGAAGCTGGATTCCCG
BLM_1	AAGACAGTCTGTCTTGGCTG
BLM_2	CCAACACCACAAATCAGCAA
BLM_3	GGAACGAACTGCTTCAGCAG
BLM_4	TACGGCCACAGCTAATCCCA
BLM_5	CGTGCAACAACTTACACCTG
BLOC1S1_1	AATGGTGGAGAACTTCAACC
BLOC1S1_2	GCGGGACAGCATGGTCACGT
BLOC1S1_3	GGAAGCTGGAACGCTCACCT
BLOC1S1_4	TTGCCAAGCAGACAGGCCAG
BLOC1S1_5	CCAGAGAAAGCTGGACCATG
BMI1_1	AAGATCAGTCACCAGAGAGA
BMI1_2	AAGGCCAACAGCCCAGCAGG
BMI1_3	GATTGATGTCATGTATGAGG
BMI1_4	GTGGAGGGAATACCTCCTGC
BMI1_5	AATGGCTCTAATGAAGATAG
BMP2_1	AGTCCACCGCATCACAGCGG
BMP2_2	GGGCCGCAGGAAGTTCGCGG
BMP2_3	TGAGGTGATAAACTCCTCCG
BMP2_4	TGGCTGCCCTCTCCAACCGG
BMP2_5	TACAGGTCTAGCATGTAGGG
BNC1_1	CAGCGGTCGCAGGATGGCCG
BNC1_2	GAAGTGTCCAGTCCAAGGCA
BNC1_3	GTTGCCAAAGTTTCAAACCC
BNC1_4	TGAAGAGGACAACAGCGGGA
BNC1_5	AATGACCCTATCAGTGCAGG
BNC2_1	AGCCAGGGAAGATTAACCTG
BNC2_2	GAACGACTACGGCTGAACCA
BNC2_3	TGTGCGGCTAAAGATCCTGC
BNC2_4	TTTGGAGGCAACCCTAGCAG
BNC2_5	CTTTGAAGATGGTACAGCCA
BOLL_1	GACAGAGCTGGAGTATCCAA
BOLL_2	GATGGAAACCGAGTCCGGGC
BOLL_3	GGGCTGAGCTACCATCACAG
BOLL_4	TTACTTACAGGCCAAGGCGG
BOLL_5	ACAGAGCTGGAGTATCCAAA
BPNT1_1	GGAACCAAGGAATATACCGA

BPNT1_2	GGTACTGAAGAACATTCCCA
BPNT1_3	TTAATAGCACTGTACTGCGA
BPNT1_4	TTGAGCAATAGAATATGCGG
BPNT1_5	AGTAACCAACTTGTTGCTAT
BPTF_1	AGATGGGATGACGTGGCCAG
BPTF_2	CAGTAACAAATCCTTTCTGG
BPTF_3	GGAGGACATGGTCTCCGAGG
BPTF_4	TCAGCTGAAGAGCCAGCAGG
BPTF_5	AAAGGAGCAGAGCAAAACCG
BRCA1_1	CAAGGAGCCAACATAACAGA
BRCA1_2	GAACTCTGAGGACAAAGCAG
BRCA1_3	TAACCTGATAAAGCTCCAGC
BRCA1_4	TAGAAACTACCCATCTCAAG
BRCA1_5	AAGGAGCCAACATAACAGAT
BRCA2_1	AACAAACTCCCACATACCAC
BRCA2_2	AATATCTGGAACTTCTCCAG
BRCA2_3	GAGCACAGTAGAACTAAGGG
BRCA2_4	TTTACAGGAGATTGGTACAG
BRCA2_5	AAAATGTCAGACAAGCTCAA
BRCC3_1	GAAGGCCAAACAGTTATATG
BRCC3_2	GCCCAAGATCCTGTGCCAGG
BRCC3_3	GCTGTCTGCAGCTTCAACAG
BRCC3_4	TCAGCGGGCCTCTCCTACAG
BRCC3_5	AGTAATGGGGCTGTGCATAG
BRD1_1	AAATAGGATTGCGAATCAGG
BRD1_2	GGAACATCCCTCCAGCCCGG
BRD1_3	GGGCTGCGCAAATATCCTGG
BRD1_4	TCCCAGGAGAAAGGCCGCCG
BRD1_5	AAGACAGATGACGACCGCTG
BRD2_1	GCAGTAACAGCAAGGAGCGG
BRD2_2	TAAGAACAGCCACAAGAAGG
BRD2_3	TTAGGCTCAAGACTCCCAGG
BRD2_4	TTTGGGATTGGACACCTCCG
BRD2_5	ACAGTGGTAGGTATCTCAGG
BRD3_1	AGACCACGAGGTTGTGGCCA
BRD3_2	GAAGGACCTGGAGGACGGCG
BRD3_3	GCAGTACATGCAGAATGTGG
BRD3_4	GGGACGATGGGTGTGGCAGG
BRD3_5	ATACAATCCCCCAGACCACG
BRD4_1	ACAGGAGGAGGATTCGGCTG
BRD4_2	GGAGCCCAAGACCACCAAGC
BRD4_3	TTAGGGTTGGAGGTCTCTGG
BRD4_4	TTCGACTGATGACTCTGAGG
BRD4_5	AGTCGAACTGTCACTGTCCG
BRD7_1	GAAGCGAGATCGAGACCGGG
BRD7_2	GAAGTCACCGAACTCTCCAC
BRD7_3	GAGACAGATCATGTTCGGAG
BRD7_4	GTCGGACAAACACCTCTACG

BRD7_5	AACTGATGAGACAATTGCAG
BRD8_1	AGGGCGGCCAGGTTCTGCAA
BRD8_2	GAAAGTGATAAAGGAAACCC
BRD8_3	TAAACATAGCTTCTCTCGGA
BRD8_4	TTTGCAATGACATTGCAACG
BRD8_5	AGGAGGTGATTATCCACTTG
BRD9_1	GAGGGAGCACTGTGACACGG
BRD9_2	GCGAAGAAGATCCTTCACGC
BRD9_3	TAGCTTTAGAGGCTTCTCCA
BRD9_4	TTTACCTCTGAAGCTGGCGG
BRD9_5	ACTCCAGTTACTATGATGAC
BRDT_1	AACAAGGGTAGTATTGGCAG
BRDT_2	AGGAAGAACAGGCGTCACAC
BRDT_3	ATGCAGAAATACATACCAGG
BRDT_4	ATTAGTGTTCTCTCTACCAG
BRDT_5	AACTCCCTGGAGATAAACTT
BRE_1	AATCGGTCACAGTTGGGCCC
BRE_2	GGAGGGCCACATCTTCTCCA
BRE_3	TCGGTTCAAGGCCACTTCTG
BRE_4	TGAGGCGGGAGCTCTCCCGG
BRE_5	AAACTGCACATACCATATGC
BRF1_1	AGAGCGCAGACCCTGCAGAA
BRF1_2	AGAGCTCTGCATCAATGCGC
BRF1_3	CAGGGACACGAACTGGCCCA
BRF1_4	GTGAGCAGGCACCTGACCCG
BRF1_5	AAGCGTGGAATATACAGGCA
BRF2_1	AGCGACGAGGGCAATCTCCG
BRF2_2	CCAGTGGATATCCATCAGGG
BRF2_3	GCAACTGCAGAACTCGACAA
BRF2_4	GTTAGTCGCAGCCAGCAACG
BRF2_5	AACATATTCCCGAAGCACAG
BRIP1_1	AGATTACTAGAGAGCTCCGG
BRIP1_2	AGCACAGAGATTCCGACCCT
BRIP1_3	ATAGAAGAACTTGTCAGCCT
BRIP1_4	TAACATCGAGGACTGTGCTC
BRIP1_5	AGATGAAGCTCATAACATCG
BRMS1_1	GGGAACGACTGAGTCAGCTG
BRMS1_2	GGGAGCCAAACAGCACCTGG
BRMS1_3	TCAAGAGATCGACATCCTGG
BRMS1_4	TCTCACTGACACACTCGCTG
BRMS1_5	AAGGGGCTCCGTGTATTCAG
BRMS1L_1	AAATGCCAGTCCATTCCCGA
BRMS1L_2	AATGCCAGTCCATTCCCGAG
BRMS1L_3	ACCAGAATACTTGGAACCGC
BRMS1L_4	AGACTGGACAACAATTAGGA
BRMS1L_5	AAATATGCAAATTCGTACAA
BRPF1_1	AACAGCTCAAGTCCTGGCAG
BRPF1_2	ACAGCTACTGGACACTGAAG

BRPF1_3	GAAGCGGGCAGCAGCACCTG
BRPF1_4	GCCACCATACGAGTGCCCGG
BRPF1_5	AGGGTGACTGCAGGCAACGG
BRPF3_1	AGAACCAGTCAACTTGAGTG
BRPF3_2	GCAGATGGGAGTGCAAGCGC
BRPF3_3	GCAGGCGGACAGCTGCTCGG
BRPF3_4	TAGTAGGCAGCAGGCAGCGG
BRPF3_5	GAGCGCCATGCGGTCCAGTG
BRWD1_1	GTAGCAGAGATGGAACAGCA
BRWD1_2	TAATTCACTGGCATTTCAGG
BRWD1_3	TACATGATCAGTACTACCTG
BRWD1_4	TAGGGACTTATCTTCCGAAG
BRWD1_5	ACAAACCTAAATATGCCTAG
BRWD3_1	ATGGCCCTGAAGGACTGCAA
BRWD3_2	CCTACCCAGATCGAAGCCGG
BRWD3_3	GAAGAGTATAGTGCTAGACA
BRWD3_4	TTGGTCGGCAGTCTCTGCTA
BRWD3_5	AGAGCATACTCTGTTAATGG
BSX_1	AAACCGTGCGGGCTTTGCGG
BSX_2	AGGTTCCTGTTACTTACCCG
BSX_3	GGTCAGCACGAAGGGACCGG
BSX_4	TACACCCGGCGTCTTCTCAG
BSX_5	CAGCTCCACTCGTTCTGGCG
BTAF1_1	AAGAAATCTTACCTCCCAGG
BTAF1_2	GAACAATGGGAAGTTAGACA
BTAF1_3	GACCCACCTGGAGACCGCCA
BTAF1_4	GTGAAGTGGATCCTAAAGAG
BTAF1_5	CACATGGCTGCTCGTTGTGT
BTBD11_1	AAAGAGCGGGAGGCGCCCGG
BTBD11_2	ACAGCGGTCAAACAAGCCAG
BTBD11_3	GCTGATGGAGTGGATCCGGG
BTBD11_4	TTCCAAAGTCAACCTCCAGG
BTBD11_5	AGATGCACAAAAGCAGTCGT
BTBD3_1	AACCGTTGAGTCCCACCTGG
BTBD3_2	ACTGGAAACGGTGACAGCGC
BTBD3_3	TGGAAACGGTGACAGCGCTG
BTBD3_4	TTTGTGGTTGGGCCACCAGG
BTBD3_5	CACTAGAAAGTATTCTCCGT
BTF3_1	ACATTGTTTGGGCAGAGCTT
BTF3_2	TCAGACTATCCGCACCAAGC
BTF3_3	AACTGCTCGCAGAAAGAAGA
BTF3_4	CTGCAGGCACAAGTGCGCAT
BTF3_5	TCAGCTGCTTTGTCTCAGCA
BUB1_1	AACGGAGAAAGCATGAGCAA
BUB1_2	ACCAGACGGACACTTACTGA
BUB1_3	GCAGAAATAGCATTTGCCAA
BUB1_4	GGTGGTGGAGACATCCCATG
BUB1_5	CAAGGAGAAGCTTATTCGTG

BUB3_1	ACGCACGGACGTGTCCCAGG
BUB3_2	CAAGCTGAACCAGCCACCCG
BUB3_3	GGGATCAGACAGTTAAACTG
BUB3_4	GTATTTGGACCCAAGCCCTG
BUB3_5	AGTCCCACACCAACACTCTG
C11orf30_1	CTCTCCGAACTTCAGCACGG
C11orf30_2	GAAGCAGTTATAACAACTGG
C11orf30_3	GACCTAATAGCTCTTCAGAA
C11orf30_4	TCCAAGTGGCAGCATAGCAA
C11orf30_5	GCACACAAGCAACCTATACC
C11orf98_1	AGAAGCTGTTCAAACGCCGG
C11orf98_2	GAAGCTGTTCAAACGCCGGC
C11orf98_3	GCGACTATGGGAGCTCCGGG
C11orf98_4	TCGCGACTATGGGAGCTCCG
C11orf98_5	AAAGATCAACCGGCCCCGAA
C14orf93_1	ATACTCACAGGATTGAGCAG
C14orf93_2	ATTGAGCATGTCCTCAGGGC
C14orf93_3	GAAGAGGAGTGTGACAGCGT
C14orf93_4	GCAGAAGGCAGTCTGTCTGA
C14orf93_5	AGAGTTGCTTGGCATAAGCA
C17orf49_1	CATGACGTCAGCGTCCACAA
C17orf49_2	AGACGGAAATAGAGATGCTG
C17orf49_3	CGCCGCCTTCACGAAGCTCG
C17orf49_4	GGTCGGAGAGATCTTCTCGG
C17orf49_5	TGGCCGACTCTTCTCCTGCG
C1orf85_1	CAAACAGAAGTAGAGTCCAA
C1orf85_2	GAGGTAGCCACATTGGGCCA
C1orf85_3	GCCACCATTACCACTGCCAG
C1orf85_4	GGAGGATATGGTCTTCCCAA
CALCOCO1_	GCTGATGGAACAGTACAAGG
CALCOCO1_	GTTCCGAGAGCCAAGGCCCA
CALCOCO1_	TGAGAAAGTTGACTCCACCA
CALCOCO1_	TGTGTGGTAATCCCGAACAC
CALCOCO1_	CAGGCTTAGAGACACAGTGA
CAMK4_1	GAGATACTTCATCCCACCAG
CAMK4_2	GAGCATACCGCAGTACCCTG
CAMK4_3	GCTGAGCGATTTCTTCGAGG
CAMK4_4	TCTCTGGTTTGAGATCACGA
CAMK4_5	AGATACTTCATCCCACCAGG
CAMKMT_1	AAAGAGATTACTCCAGCCCA
CAMKMT_2	ATCACAAGGAATCAGAAGGC
CAMKMT_3	CAGTTGGCTGCACCACTCGG
CAMKMT_4	TTTAAAGTATTCCCTCGGCG
CAMKMT_5	ATGTGCAAGACATCATCACA
CAMTA1_1	AGGCTTGCCGCAGTCCTCGA
CAMTA1_2	GAAACTCAAGGTCCAGGGAG
CAMTA1_3	GAGGAGGATGTGGCGCGCGG
CAMTA1_4	GGAGCCAGTAGCACCTCCGG

CAMTA1_5	GATGCCCACGGTGAAAACGG
CAMTA2_1	GAATACCAAGGACACCACCG
CAMTA2_2	GCAGCCAGTAGCAGCGCCGA
CAMTA2_3	GGATGGGAAGACCACCCGAG
CAMTA2_4	TGCCTCCAGAGAGGCTACGG
CAMTA2_5	CATCCTCTACAATCGCAAGA
CARF_1	AAGAACTTGGTAGATGCTGG
CARF_2	GCACAAGTGATTATACCTCA
CARF_3	GTGGAAACAAATCAGACCAG
CARF_4	TCTCACTTACACAGTAGCCA
CARF_5	AAAATTGGAGATTCATACCG
CARHSP1_1	GAATGAGAAGCTGCAGGCCG
CARHSP1_2	GGTGAGCGCTCACGGCTCCG
CARHSP1_3	GTGAGCGCTCACGGCTCCGA
CARHSP1_4	GTGAGTGATGACGACCTCCA
CARHSP1_5	CAGCTGGAGTAATGAAGCCA
CARM1_1	CGCGGTGGAGGCCAGCACCA
CARM1_2	GAACACCGACACGGTAGCGC
CARM1_3	GCAGTCCTTCATCATCACCC
CARM1_4	GCTGAACACAGACCGCTCCA
CARM1_5	TAGAGCTGTTCATCCGTGAA
CASZ1_1	CGATGAGGAAGACCACGCGG
CASZ1_2	GCTGAGGGTATCCTCGGAGG
CASZ1_3	TACACTCAGGGTCAAGGCAG
CASZ1_4	TAGACGCTGCAGTCGTCCAG
CASZ1_5	AATACCCAGCTCATTAACGA
CBFA2T2_1	CCGCCACAGAGATTCAGCAA
CBFA2T2_2	GCTGGAAGGCGGCCGCTCCA
CBFA2T2_3	GGAAGAGGCCCAGTCCAGAG
CBFA2T2_4	GGAGTGAAGGACACTGGCCT
CBFA2T2_5	CAATAAATCCTGGAGGACCG
CBFA2T3_1	CACCGCCTGCCGCATCCCAG
CBFA2T3_2	GGAATGACAAACGGCCGCAG
CBFA2T3_3	GGAGCCACAGGTGGATCCCG
CBFA2T3_4	TCAACGAGAACGGCAAGAGG
CBFA2T3_5	CAAGCTCACAGAGCGTGAGT
CBFB_1	AGCGTGCCTGGCGTTCCTCG
CBFB_2	GAGAAGCAAGTTCGAGAACG
CBFB_3	GAGAGACAGATTGGTTCCTG
CBFB_4	TAGAGACAGGTCTCATCGGG
CBFB_5	AAGTCGACATACTCTCGGCT
CBL_1	ATGAGGAGAATTCTCAGCCT
CBL_2	CCTGTCGAAAGCTCTTCCAA
CBL_3	GACGGTGGACAAGAAGATGG
CBL_4	TTCCTGAGCAAAGAGGACCA
CBL_5	AGACCATATCAAAGTGACCC
CBX1_1	AAACCTCGAGCAAAGCCTCG
CBX1_2	CTTTGCCCTTTACCACTCGA

CBX1_3	TGATTCTGAAGATAAGGGAG
CBX1_4	TTTCAGTGAGGACAACACAT
CBX1_5	AAAAGTTCTCGACCGTCGAG
CBX2_1	ACATAACAGCTGGGAGCCGG
CBX2_2	CTTGCTCAGGATGCACTCGG
CBX2_3	GAGCTGCCAGGCCTGCAACG
CBX2_4	GAGGTGCAGAACCGGAAGAG
CBX2_5	AGTGACTTAGATGCTAAGAG
CBX3_1	AAACAGGCTGACAAACCAAG
CBX3_2	ACACAGTGCTGACAATACTT
CBX3_3	AAAGCTGGCAAAGAAAAAGA
CBX3_4	AGAGCCTGAAGAATTTGTCG
CBX3_5	AAACCAAGAGGATTTGCCAG
CBX4_1	ATATAACACGTGGGAACCGG
CBX4_2	CGAGAAGAAGCGGATCCGCA
CBX4_3	GTGGGAGAGATTCAGGCCGA
CBX4_4	TGGCGAGCACGTCTTCGCGG
CBX4_5	CCTTGCAAGATATAACACGT
CBX5_1	ACCCAGGGAGCACAATACTT
CBX5_2	GACATGGCATGCATATCCTG
CBX5_3	GCAGAGCAATGATATCGCTC
CBX5_4	GGAGGAGTATGTTGTGGAGA
CBX5_5	AGAAGTCAGAAAGTAACAAG
CBX6_1	CAAGATGGAGCTGTCTGCAG
CBX6_2	GGGCGGCCAGAAGTAGCCCA
CBX6_3	TGAGGCCTCAGCCTTGCCGG
CBX6_4	TGGGCCAGGTTACCTTCAGG
CBX6_5	AACCTGAAGGTGATCGACAA
CBX7_1	CGTAGGCCATGACGAGGCGG
CBX7_2	GCAGATGCCGACCTGGCCGA
CBX7_3	GCGGAGCTCCCACAAGGCCA
CBX7_4	GGAGGAGAGAGACCGAGCAT
CBX7_5	AGGAGAGAGACCGAGCATCG
CBX8_1	ACTGCCAACCTACTTTGAGG
CBX8_2	CAACATGGAGCTTTCAGCGG
CBX8_3	GTACAGCACATGGGAACCGG
CBX8_4	TGACAAGCCCAGCTCACCGG
CBX8_5	CTCTATGGCCCCAAAAAGCG
CCDC101_1	CGAGCAGTGGATCCTGGCCG
CCDC101_2	GCGGGAATCGGCAGACACGA
CCDC101_3	TCTTGTCCAGAGCTTTCCGA
CCDC101_4	TGGCCTCTACACAACCGCCA
CCDC101_5	ACAATGACTCGGAGCCACCC
CCDC169-S(	AAGACACAAGATTACGGAAC
CCDC169-S(	AAGACAGTGAGTGCCAAGAG
CCDC169-S(	ATCACATTTCTACTCACCAC
CCDC169-S(	AAAAGGGAGAAAGAATGATG
CCDC169-S(	CACCATCAGTGACACGAAGG

CCDC59_1	AAAGAAGGCTCAAACGTCAC
CCDC59_2	ACTGTTCTTCAAGCAACGGC
CCDC59_3	TGAGGCGGTCCGCGAAGTGG
CCDC59_4	TTAGTCGGCTGCAATGGCGC
CCDC59_5	AGAATGTGAGACAGAAGACA
CCDC71_1	CAGGTGACAGGTTCACGCGG
CCDC71_2	GTGGCTCGGACCCAGCCCAG
CCDC71_3	TCAAGTGCCAGCCATGCCCG
CCDC71_4	TGTTCGAGCTACCTTGGCCT
CCDC71_5	CAACCTACCATCCTGCGCAG
CCDC79_1	AACCACTGCAAGTTTAGCAC
CCDC79_2	AAGACCACAACTGATAACTC
CCDC79_3	AATCAAGAAGCTGCACACGT
CCDC79_4	TCAGCAAGGACGGAAGGCTG
CCDC79_5	CATGCAGATCGGATTGGTCG
CCNH_1	AAGGATTGTGGACAATAAGG
CCNH_2	CAAAGCCGTGGCCAACGGGA
CCNH_3	CAAATTCAGATGCAAAGCCG
CCNH_4	GTCCAAGAGGACTCTCCCGG
CCNH_5	CAGTAATGGAATATCACCCC
CCNK_1	GATGGTTCGGAGCTTTGAGA
CCNK_2	GCACCACCTTGTCACTGCAG
CCNK_3	GGGTGGGCTGGAGGCAACGG
CCNK_4	TGAACCGAGCGCCCTCTCGG
CCNK_5	ATCCTGGATCTTTACTCACA
CCNT1_1	CCTGCAGTACACACCTCCTG
CCNT1_2	GAGGAGATTCCACCAGACCG
CCNT1_3	GCTGAGTGGGCTGAACACCC
CCNT1_4	GGAAGAACAACAACAAACGG
CCNT1_5	AAAACAAAAGCAGATGACCG
CCNT2_1	AACACGCCGAGCCGCCGCTG
CCNT2_2	GAAACTGGACAAGAAGCCAG
CCNT2_3	TATCCGCCTCCACTCCGCAG
CCNT2_4	TGAAGAAGATTCGAAACTGG
CCNT2_5	AATAATTCTAGAGTAACTGT
CDA_1	CGTTCAGCACAGATGCCCAG
CDA_2	GCTGCTGGTTTGCTCCCAGG
CDA_3	GTGTACATGACCAAGCCGGA
CDA_4	TGAACGGACCGCTATCCAGA
CDA_5	AATCATCTTGCATGTCACTG
CDC34_1	CCACTTCCTGTACATCACGG
CDC34_2	GGGCGATCTATACAACTGGG
CDC34_3	GTGGCACCCTAACATCTACG
CDC34_4	TATCTCCATCCTCCACCCGC
CDC34_5	CATCTTGGTCAGGAACCGAA
CDC45_1	GAAGCGCACACGGTTAGAAG
CDC45_2	GGTCCGCAGAACTCACCTGA
CDC45_3	TGACCACGTGCAATATACGC

CDC45_4	TTTCCGCAAAGAGTTCTACG
CDC45_5	CAATGTATACAACGATACCC
CDC5L_1	AATTGCACGTCAAACTGCCG
CDC5L_2	AGTTGATGCCAACTCAGTGG
CDC5L_3	GAGAAGTCACTCTCATGCAA
CDC5L_4	GGGCGTATGGAGGAATACCG
CDC5L_5	ATCCAGAAACAAAACCAGCG
CDC6_1	AAGTGAGAATTGCTTAGCCA
CDC6_2	GGTGGGAAGTTCAACAGCTG
CDC6_3	GTTTACCCAGACGTTTCCTG
CDC6_4	TTTGCGACAGACTTTACTGT
CDC6_5	ATAGCGTGTACAATACATCC
CDC73_1	AAAGACCAGATGCAACCAGG
CDC73_2	ACATAACTGCCCTTAAACAG
CDC73_3	ACCCAGAGAGTACTACACAT
CDC73_4	GAAGAAGGAGATTGTGGTGA
CDC73_5	AAGACCAACTATGTTGTTTG
CDCA2_1	CTCTAGACACATCTTAACAG
CDCA2_2	GAGCAATGAAACGAATCAGA
CDCA2_3	GGTGTTCCTCCTTTACGCAA
CDCA2_4	TCGGGAATAGGACTGAGGAG
CDCA2_5	GCAGCTCGGGAATAGGACTG
CDCA7L_1	AGTGAGGCGGGCCTTCTCGG
CDCA7L_2	AGTGGAGGATATCACCGAAG
CDCA7L_3	AGTTCTGTGGACCATGCCTG
CDCA7L_4	CAAACTCTTCATCATCACTG
CDCA7L_5	ACTGTAGACAGGTGATACAA
CDK1_1	AATCCATGTACTGACCAGGA
CDK1_2	GAATCCATGTACTGACCAGG
CDK1_3	GATCTCCAGAAGTATTGCTG
CDK1_4	GCACTCCCAATAATGAAGTG
CDK1_5	ACCCTTATACACAACTCCAT
CDK12_1	AAAGAAGTGGCTCTTACAGC
CDK12_2	CTTCACAGAAACCTGTACAG
CDK12_3	GACAAACAGAAAGCGACTGG
CDK12_4	TCATGTTAACAACACTTCGG
CDK12_5	ACTGACCGACTGCCTTCTCG
CDK13_1	ACCGCCCAAGGCCTACCGGG
CDK13_2	ATGGGAGGCAAAGGTAACGG
CDK13_3	CGGACAAGTTTACAAAGCCA
CDK13_4	TGATCTTTCAAAGAGTCCAG
CDK13_5	AGAATATGTGGGCCTCGCTA
CDK17_1	AATCTGTGTTGAGTACTCCG
CDK17_2	ACACTGTGTGGTCTCCGCAA
CDK17_3	ACATAGACGGATCTCAATGG
CDK17_4	GCAGACATCAGAATACCTGA
CDK17_5	CATGAGTATGCACAACGTAA
CDK2_1	AAGCAGAGAGATCTCTCGGA

CDK2_2	AATGGCAGAAAGCTAGGCCC
CDK2_3	TAACAAGCGGATTTCGGCCA
CDK2_4	TCAGGCATAGAAGTAACTCC
CDK2_5	CAAATATTATTCCACAGCTG
CDK2AP1_1	ACCGTCCCTAGGCTACACCC
CDK2AP1_2	GGAGCAAGAGTGCCATGGAG
CDK2AP1_3	TCACTCACCGGCGTTGAGGG
CDK2AP1_4	TGAAGACGTTGCCATGCTGG
CDK2AP1_5	GGTGCCCCAAAGCAAATACG
CDK3_1	GAAGGTAGAGAAGATCGGAG
CDK3_2	GAGCAGCGAGATCTCCCTGA
CDK3_3	GCTGCCCAAGAGAATCTCGG
CDK3_4	TCATGGGTGTAGGTGCGCAG
CDK3_5	GGTGGATGACCCGATGTGAG
CDK5_1	ACAGCGGACGGGAATCCCAA
CDK5_2	AGAGGGCCACTGCTCCTCGG
CDK5_3	GATGGACGTGGAGTACAGCT
CDK5_4	TGAGTAGGCAGATCTCCCGG
CDK5_5	CCGGGAGACTCATGAGATCG
CDK7_1	ATCTCTGGCCTTGTAAACGG
CDK7_2	TAGCTCCAAATAGTAACTCG
CDK7_3	TGACATCCAGGTGTTGGCCC
CDK7_4	TGTGATGCAAAGGTATTCCA
CDK7_5	AGCTCCAAATAGTAACTCGG
CDK8_1	AATGTAATGGGATTTCCTGC
CDK8_2	CAGCAATTCACCTTACCCAG
CDK8_3	GAAGCCAGTTCAGTTACCTC
CDK8_4	GAGGGCTGCAAAGTTGGCCG
CDK8_5	AAGCCAGTTCAGTTACCTCG
CDK9_1	AAGGATCTTGATCTCCCGCA
CDK9_2	GCACCGCAAGACCGGCCAGA
CDK9_3	GGCAAAGCAGTACGACTCGG
CDK9_4	GGTATATACTACCCTTGCAG
CDK9_5	CCAGAGTGTCACCACACGGT
CDKN2A_1	ACGCACCGAATAGTTACGGT
CDKN2A_2	AGATCATCAGTCCTCACCTG
CDKN2A_3	GTGGCCAGCCAGTCAGCCGA
CDKN2A_4	TGACTGGCTGGCCACGGCCG
CDKN2A_5	CACCGAATAGTTACGGTCGG
CDKN2B_1	ACGGAGTCAACCGTTTCGGG
CDKN2B_2	GGTGCATGATGCTGCCCGGG
CDKN2B_3	GTGGACTTGGCCGAGGAGCG
CDKN2B_4	TGAGGGTCTGGCCAGCGCCG
CDKN2B_5	CCGGTCGGGTGAGAGTGGCA
CDKN2C_1	CCAGGAACTCCACCACCCGG
CDKN2C_2	GAGCCTGGCCAAATCACAGG
CDKN2C_3	TGGCGTCCGCAGCTGCCAGG
CDKN2C_4	TTGGCGTCCGCAGCTGCCAG

CDKN2C_5	ACAAACTAGTAAGTTGCTCT
CDKN2D_1	CTGCTGGAGGAGGTTCGCGC
CDKN2D_2	GCTGATGTCAACGTGCCTGA
CDKN2D_3	TCAAGAGGGTCACACTGCTG
CDKN2D_4	TGGCAGCACCGCCATCGCCC
CDKN2D_5	ATGGACTGGACTGGTACCGG
CDX1_1	ACCGGGCTAAAGTCTGGAGG
CDX1_2	GACCAAGGACAAGTACCGCG
CDX1_3	GCCGTTACATCACAATCCGG
CDX1_4	GCTGCGCCGCATCCACTCGT
CDX1_5	AAGGATTCGCCCGTGTACCC
CDX2_1	GACGAAAGACAAATATCGAG
CDX2_2	GCCGGGAGACAGCTGCTCGG
CDX2_3	GTCGCTACATCACCATCCGG
CDX2_4	GTGGCTCACGGCCTCAACGG
CDX2_5	CAAATATCGAGTGGTGTACA
CDX4_1	AAAGAAGCAGGCATGTACCC
CDX4_2	GTCGGTCGAGCAGAAAGCGG
CDX4_3	TCAGAGCTGGCAGTTAACCT
CDX4_4	TGGCGGGTCGCTTGTCCCGA
CDX4_5	CCCATTGTACTAGACGGCCC
CDY1_1	TACAATGTCTCTGTATGTGC
CDY1_2	TCTGCACCAGGACGTGACAA
CDY1_3	TGCTGGCAGCAAATAACTTG
CDY1_4	TTTGACCACAAAACTGTGAG
CDY1B_1	ACAGGTTCAGCTACCCGAAA
CDY1B_2	AGAAGATGCACTTCACCATA
CDY1B_3	CAGGACGTGACAAAGGGTCC
CDY1B_4	TTGCTGCCAGCAAGAACGTT
CDY1B_5	CAGTGACTGATAAACACCAC
CDY2A_1	AATATTACTGATGACAGCAG
CDY2A_2	GACCACAAGAAAACTGTGAG
CDY2A_3	TCTGCACCAGGATGTGACAA
CDY2B_1	ATAGACCCATTAGCAGCCAA
CDY2B_2	ATGACAAACAGGATGACACT
CDY2B_3	CTGCACCAGGATGTGACAAA
CDY2B_4	TACAATGTCTCTGTATGTGA
CDY2B_5	AGCAGATCAGCAGGACACGG
CDYL_1	ACAACAGAAGACGCTGCCAA
CDYL_2	GTACAGCTCCTCGGAAGCCA
CDYL_3	TAGGTCCATGTTCTTCCGGC
CDYL_4	TGGACGGAAGCTGACAGCGC
CDYL_5	CGACTTCAACAGACGCCACA
CDYL2_1	AATGGGAGTATCTTATCCGA
CDYL2_2	AATGGGCCGGCCCTGGGCCT
CDYL2_3	ACTGCTGCTCCTCAGCGCAG
CDYL2_4	GAAGGCTCTGCTCTGACCAA
CDYL2_5	AATTCGCTTCCGTTTATGGG

CEBPA_1	CCTGCCGTCCAGGTAGCCGG
CEBPA_2	GTAGAAGTCGGCCGACTCCA
CEBPA_3	GTCGGCCGACTTCTACGAGG
CEBPA_4	TGAAGCCAAGCAGCTGGCGC
CEBPA_5	CACCCCGACCTCCGCGCGAG
CEBPB_1	GAAGTCGTGGTGCTGCCCGG
CEBPB_2	GAGCAAGGCCAAGAAGACCG
CEBPB_3	GATGCTGCCCAGCTCGCCGG
CEBPB_4	TTAAAGGCAGGCGGCGGCGG
CEBPB_5	CCTCTTCTCCGACGACTACG
CEBPD_1	GCTGCTTGAAGAACTGCCGG
CEBPD_2	GGTCGGCGAAGAGCTCGTCG
CEBPD_3	GTCGTGGCACAGCTCCAGGG
CEBPD_4	TGCGGCCGCCAAGCTCACCA
CEBPD_5	AACAGCAATCACAAGGCGGG
CEBPE_1	GAGGCGGCCAACCTCATCAA
CEBPE_2	GCAGCCACTCGAGTTCTCAG
CEBPE_3	GGAGGGTAGGCAAAGGGCCG
CEBPE_4	TCTGGCGGAAGAGGTTGCGG
CEBPE_5	CAGACTCGATGTAGGCGGAG
CEBPG_1	ACATGCCAGCGGCTTACAGC
CEBPG_2	ACGCCGAGAGAGGAACAACA
CEBPG_3	CATACCCAGGCACATGCCAG
CEBPG_4	TAAGCCGCTGGCATGTGCCT
CEBPG_5	AATTAGTGTTATCCATACCC
CEBPZ_1	AAACTGGAACAGTTGTCCAG
CEBPZ_2	CAAATTATCAAAGTGCACCC
CEBPZ_3	GAGGAAGTGTTACGGCTCGG
CEBPZ_4	TTGGAACACGTCATCAACCC
CEBPZ_5	AAAGCTCCACATATAAATGG
CECR2_1	AGGACATCATCAACTACCGC
CECR2_2	ATTGGGTGAAGACAATTCTG
CECR2_3	CAGGCAGGAAGTCCTCACTG
CECR2_4	GAGCGAAGAGGAGAAAGCTC
CECR2_5	AGGTGGTCAGACATCCACCT
CECR6_1	ACAGCCGCGGACTTCGGCGG
CECR6_2	ACGCGGAGGAGACCGAGCGA
CECR6_3	CGGGAAGGAACCGGACGCGG
CECR6_4	GGAGGAGAAGGCGACAGGCG
CECR6_5	ACAGCGCCTGGTAGCCCCAG
CELF3_1	AAGTACACCGGGCTGCACAA
CELF3_2	CGAACAGAAGACGCTTCCAG
CELF3_3	GCACTCGTCGATGGTCCCGA
CELF3_4	GTGGGTCTGGAACTTCACGA
CELF3_5	CACTCGTCGATGGTCCCGAA
CENPA_1	AGGATGTGCAACTGGCCCGG
CENPA_2	GAAGCTGCCCTTCAGCCGCC
CENPA_3	GCGGCCGGTTACCTAAGGAG

CENPA_4	TTGTAGGGCCAATAGGGCCT
CENPA_5	ATATGTGTTAAATTCACTCG
CENPB_1	AGATGAGGAGAGCTCCTCGG
CENPB_2	GAAGAACCACGCCAGGCAGG
CENPB_3	GAAGGCGCTGCGCATAGCCG
CENPB_4	GAAGTCACGGATCATCCAGG
CENPB_5	CATAGCCGCCTGCTTTCGTG
CENPC_1	AAGTACGTAAATCACCTGGA
CENPC_2	AGATCCTTTGCAGCCAACGA
CENPC_3	GAGTGCAGTGCTCTCAGCCG
CENPC_4	GGAGAGCGAATAGATTATCA
CENPC_5	AAAAAACAGATGCCACCTGT
CENPE_1	AAACTCACCTCCCTTATAGA
CENPE_2	TAAGATCCCAGTGCTTCATG
CENPE_3	TAATTCCTGTTGCAACGTGA
CENPE_4	TGAGACCCAGAAGTTAAACC
CENPE_5	AAAGGACCTAGAAAAACAAG
CENPF_1	AATGTCGCGGTGATTCATGG
CENPF_2	ATACGACCAGGCGTCAACCA
CENPF_3	GATGAAGGCCAGACTCACCC
CENPF_4	TTAGTGGAAGACATACCAGG
CENPF_5	ATTTGTCAGAAAATGAACAG
CENPH_1	AAGGGAACAACCTGAGGAGC
CENPH_2	GAGTAAAGTCAATTGGGCAG
CENPH_3	GCAAGACGCCGACGAGCCCG
CENPH_4	GCTGCAGAACAATTTCCTTA
CENPH_5	AGAAAATTAGCAGACAGTCT
CENPI_1	AAAGAGAGTTAAGAACGTCC
CENPI_2	AGTCACATACCTCTAAGCAA
CENPI_3	GAGATGAGCCAGTCTTGCTG
CENPI_4	GCCGTGAAGCAAAGAAACCG
CENPI_5	CAAGTGTTCTGGTAGCACCA
CENPJ_1	ACAAGAGAAGCTGCTCACCA
CENPJ_2	TAAGTAGCTCACCTGTAGGA
CENPJ_3	TGCGAGTGAATTCAAAGAAG
CENPJ_4	TGGATAGACAGCAACTCCAG
CENPJ_5	CCTGTGCAGCCAGTATCGCA
CENPK_1	ATGTCGAAAGGGAACAACGG
CENPK_2	GCTGAGACAAGATCTTGAAA
CENPK_3	TAACCGCTGAACTCAGTCAA
CENPK_4	TATGAGAACGTCTTCAGTCA
CENPK_5	ACTCACCGATTCAAATGCTC
CENPL_1	GAATAGGTCAACTTCTTCCT
CENPL_2	GATGCACTAGGAGTTGACTC
CENPL_3	GTAGCTTCAGCACATACTGA
CENPL_4	TTATCCTGACTCCACCTCGA
CENPL_5	ACCTGCAACTGCGAACACTG
CENPM_1	AAGGGAGGGACTTTGCCAAG

CENPM_2	AGCTGGCATCCACATGGCGC
CENPM_3	AGGCCGGGCAGCTTGTCCAG
CENPM_4	TGCGCAGCTGGTGGGCACGG
CENPM_5	AACACGATCAGGTCAATTCG
CENPN_1	CTGGGAGTAGTACACCACGT
CENPN_2	TAAGCTGACCACTCACCCGC
CENPN_3	TGAACTGACAACAATCCTGA
CENPN_4	TTGGGTATGCAAGTGAGCAG
CENPN_5	CTTACAAATGATGTCTAACA
CENPO_1	AATGACAGCAAGTTACACAG
CENPO_2	GAGGCAAACACTTGATGCAA
CENPO_3	TGTAGAACCCAACCAAACAG
CENPO_4	TTTGCAGCTATCTCTTCCAG
CENPO_5	AGTACCTGAATGCTTACTCT
CENPP_1	AAGCTGAGATCGCGGCCCTG
CENPP_2	AGATGCCGTGTACCTCTCGG
CENPP_3	CATGGACGCAGAGCTGGCAG
CENPP_4	GATGCCGTGTACCTCTCGGA
CENPP_5	AATTAAGTGAATTTGTGTCT
CENPQ_1	ATAGGCACTTACCTGAAGTG
CENPQ_2	ATTCCCAGTCATTAACTCTG
CENPQ_3	TCAGATTCTGGCAAGTGAGG
CENPQ_4	TCTCTGGTACTCTTTGACAG
CENPQ_5	AAGCACACTAACCTAAAACA
CENPT_1	TACCCAGCCCGAGCACTCCG
CENPT_2	TACCGACTCAGGCATCAGGA
CENPT_3	TCGGCCCATATTCAGGCCAG
CENPT_4	TTGGCCACTCAACTTCCTGG
CENPT_5	ACTTGGAGGAACAGACACCT
CENPV_1	AACGCCACATCTCTTACAGA
CENPV_2	CGGGAGCAAGAGCCAGGCGG
CENPV_3	GCGGCAGAAGCTTACCTCCG
CENPV_4	TGTGCGGAGTATGGTCACTG
CENPV_5	CAAAACGAACTGCTCCACAG
CENPW_1	AGACTCGCTTTAGAAAGCCA
CENPW_2	GAGGAAGCAGATAAAGCGGA
CENPW_3	GCATGTACTGGCCGCAGCAA
CENPW_4	TGTCGACCATAGTCTCCCAG
CENPW_5	ACTCGCTTTAGAAAGCCACG
CERS2_1	GAACATTTCTACCTGACCAG
CERS2_2	GAAGAGCAGGGACCAGTAGA
CERS2_3	GAGGAAGCCTGACCTCCAGC
CERS2_4	GCTGCCTGTGAACTTGACCT
CERS2_5	CAAAGAAGTATCGAACGATG
CERS3_1	ATCGTGTACAATCATCACGA
CERS3_2	TAACTTGACGGAGCGCCAGG
CERS3_3	TCAGATCTTGAGGATCACGA
CERS3_4	TTGGCTATATGACTTATGGG

CERS3_5	GGAGTCGGCGGAATCAAGAG
CERS4_1	ACAGAGCTAGAAGACCGGGA
CERS4_2	CGAGAGGCCGCCCACGAAGG
CERS4_3	CGCTGCAGCAGACCCAGCGA
CERS4_4	GAGAAACACTTCCTCACGGA
CERS4_5	AGAAACACTTCCTCACGGAA
CERS5_1	AATGCTGGTTTCGCCATCGG
CERS5_2	AGAGGATGTGCCGGCCGCGG
CERS5_3	GGAGAAGGAGATAAGCCCAA
CERS5_4	TGTCGGATGTCCCAGAACCA
CERS5_5	ATCAACAATATGGTTCGAGT
CERS6_1	AGAAGCCAAGCACGCTGACG
CERS6_2	GGCGGACCTGAAGAACACGG
CERS6_3	TGCCTCGTATTCCACAACCA
CERS6_4	TTTCAGGAATCTGACTCCGT
CERS6_5	GGTCTTCACTGCAATTACAA
CHAF1A_1	ACTCCCAGGAATGTTCGCCA
CHAF1A_2	CCAGAAACCAAAGACTCCAC
CHAF1A_3	GTGGAGCGTGGGAAGGGCGA
CHAF1A_4	TGGAAAGCGGTCCGACGCCG
CHAF1A_5	AGGGCGACGGTGTTCCCGAG
CHAF1B_1	ACAGCAGCGAACAGCAAGAG
CHAF1B_2	CCTGACATTGGTGTCCACGC
CHAF1B_3	GCTGAACAAGGAGAACTGGA
CHAF1B_4	GTAGCAACATATTGACCCAA
CHAF1B_5	AGACTGAGTTTCACTCCCGA
CHD1_1	AATTACAAAGCCCTCAGCAA
CHD1_2	AGAGGATGTTCCTCAACCTG
CHD1_3	TATAACAACAGTGTCAGCAG
CHD1_4	TCAGCTCCATCAACTTTCGG
CHD1_5	AAGCAGCCATCCTATATTGG
CHD1L_1	AGGAGCAGGACTTACGGCAG
CHD1L_2	AGTTGGAGACCACCTGACTG
CHD1L_3	GCTGCATAAGACCTTGTCAG
CHD1L_4	TCTGCAATGCTGACATGCCA
CHD1L_5	ACAGAAGTAGTGATATACCA
CHD2_1	AAGCAGCCGAAGACTCAGCG
CHD2_2	GAAGCTAATGGCGACCCTAG
CHD2_3	GTTGGTGGAACAGGTAGGAG
CHD2_4	TGAACCCGAAGCTTCTTCAG
CHD2_5	AAGGCAGACTCGTCGAAGAG
CHD3_1	ACGAGGACCCAAGAAGCAGA
CHD3_2	TAAGATGATGACCATCCTTG
CHD3_3	TGTGGAGCATGTGTTCTCTG
CHD3_4	TTTCTCTTAGGACAGTGAGG
CHD3_5	ACCATATGACCAGTCCACGT
CHD4_1	AAAGAGGTGATGCTTCAGAA
CHD4_2	GGACAGGCAGACTTACCAGC

CHD4_3	GGAGAGAGGGCAGATGAGCG
CHD4_4	TATAGGAGGAGACAACAAAG
CHD4_5	ATGTATGTCGTAACCTATGT
CHD5_1	CGTGGACTACCTGTTCTCGG
CHD5_2	GAAGGCTCACGGGCTCCACA
CHD5_3	GCACCGAGGAGGAGCTGCCG
CHD5_4	TTGCGGATAGGCACAGGCTG
CHD5_5	CCAGAGCCGTAGTCAAAGGG
CHD6_1	AACAGCTCCAAGTCGAACGG
CHD6_2	GCCTGACGAACCCATCTCTG
CHD6_3	TGAGGACCTGGACTTCAAAG
CHD6_4	TTGGTGCCCGAGGCCTCCCG
CHD6_5	CCCGGAAGCACTCCGCTCGG
CHD7_1	GAAGGAACCGAAAGAACCCA
CHD7_2	GGAGAGGCATAAGTTCCCTG
CHD7_3	GTTGCAGAAGGAATCTGGAG
CHD7_4	TGACTGGGAGGTGTTGGAGG
CHD7_5	GGATCTGATCACACCCACAG
CHD8_1	GTGGCACAAATTCAGGCCCA
CHD8_2	TATAGGCTGGCTCTTCCCAA
CHD8_3	TATGCGGATTGTGAAGAAGG
CHD8_4	TGAATCGAAACGCATCACCC
CHD8_5	ATGAGAAATATAATACCATG
CHD9_1	AGTGGAGCAATAATCAGGAA
CHD9_2	ATAGAAGATCTGCTTCGAAG
CHD9_3	ATAGATATAGAGGCCATCAG
CHD9_4	GAAGAAGACCCTCCTGAAGG
CHD9_5	AATGTTGACTCATCAAAGTG
CHEK1_1	CTTCCATCAACTCATGGCAG
CHEK1_2	GGAATGGTAATTCTTGCTGA
CHEK1_3	GGTGTGTCAGAGTCTCCCAG
CHEK1_4	TTGGTGCAAACCCTGGGAGA
CHEK1_5	ACACCACCTGAAGTGACTCG
CHMP1A_1	AGGAGGACCAGCTGTCACGG
CHMP1A_2	GGAGGCCACTGCGTCTACGC
CHMP1A_3	GGCCCAGGTGACCAAAGCCC
CHMP1A_4	GGCGGAGAAGGACTCCAAGG
CHMP1A_5	AGACAGCTGTGACTATGAAG
CHMP1B_1	CATCCGCCAGAAGAACCAGG
CHMP1B_2	GAGTGCGCGAGTCGATGCAG
CHMP1B_3	GCCGTCACACTTGATCCCGA
CHMP1B_4	GGGCAAGGTGACCAAGTCGA
CHMP1B_5	AACCACACCAGCCATCGACT
CHMP2A_1	GAAGGGTGTCACCAAGGCCA
CHMP2A_2	GATGCCATTGATGATGCCAT
CHMP2A_3	GATGGCCAAGCAAGGCCAGA
CHMP2A_4	GCTACTGCGGCAGAACCAGA
CHMP2A_5	AGACGCCAGAGGAGCTACTG

CHMP2B_1	AGAGTTACGAGGTACACAGA
CHMP2B_2	CTTCTTGAAGAGGGACGCCA
CHMP2B_3	GAACAGAATCGAGAGTTACG
CHMP2B_4	GAGAGTTACGAGGTACACAG
CHMP2B_5	AACAATGCAGAATTTCCAGA
CHMP4B_1	AAGCAGCTGGCGCAGATCGA
CHMP4B_2	AGAGGAGGACGACGACATGA
CHMP4B_3	ATAGAGGGAACATTTGGTAG
CHMP4B_4	GTTTGGAGAAGAGTTTGACG
CHMP4B_5	CACGGCACCAAAAACAAGCG
CHMP4C_1	AATCCAGAGAGAAATCGCCC
CHMP4C_2	AGGAAGGCACATTTGGAAGG
CHMP4C_3	ATGTTCCAGCATCTTCCCAG
CHMP4C_4	GAACTCACACACCAACACTG
CHMP4C_5	ACACCAACACTGAGGTGTTG
CHMP5_1	CAGTCAGTCAGGCTGGGCGG
CHMP5_2	CTTCGGGAAAGCGAAACCCA
CHMP5_3	GATGAGAGAGGGTCCTGCAA
CHMP5_4	TTTGAAGGACACCAAGACCA
CHMP5_5	AGCTGGTCTTGTAAATCCTA
CHMP6_1	GAAGAGGATCGCCCAGCAGC
CHMP6_2	GCGGCCGAACAGGTTACCCA
CHMP6_3	GGATGAAGACGCCATCCTGG
CHMP6_4	TCAGGGTGCCACCTACCCGC
CHMP6_5	CCAGATCGAAATGAAAGTGA
CHMP7_1	ACAGCAGCTGGATCTCCTGG
CHMP7_2	AGATTGTGAAGTTTGCCCGA
CHMP7_3	GCTCTGACAGGGCCACCACG
CHMP7_4	TGAGCCACAGCCGCCGCCAG
CHMP7_5	ACGGACAGAGAAGCGCATCG
CHRAC1_1	GGTGTCCAGCATCAACCAGG
CHRAC1_2	GTCGTGGGTAAAGACAAGGG
CHRAC1_3	GTTGATGCTGGACACCTCGG
CHRAC1_4	TATTCCTACAGACACGGCAG
CHRAC1_5	GCCACCTATTCCTACAGACA
CHTOP_1	AAGAGCTTAAAGCAGCGCCT
CHTOP_2	ATCCAGAGAGGCTTGCCCAG
CHTOP_3	GAAGAGCTTAAAGCAGCGCC
CHTOP_4	GAGCTACGGCTCGTCCACCT
CHTOP_5	ACGGTTAGGCCGACCCATAG
CHUK_1	AATTGGGACCCTCAGCAGAG
CHUK_2	ACAGACGTTCCCGAAGCCGC
CHUK_3	AGGCCTTTACAACATTGGCA
CHUK_4	GAATGAAGACTTTCATCAGG
CHUK_5	AAAGCTCCAATAATCAACAG
CIAO1_1	ACAGTGTGTAACCACTCTCG
CIAO1_2	ACCCTGCTGGCCTCGTGCGG
CIAO1_3	AGAGGCCAGGTAATTACCGC

CIAO1_4	GGTTTGGCACCCAAGTCAGG
CIAO1_5	AGGGCCATGAATCCACTGTG
CIC_1	GACCGAACATGCCGAGGCCA
CIC_2	GCGAGTGGTGGTATGCCCTG
CIC_3	GCTCAGACACCAAGGCTCCG
CIC_4	TAAGGGTCCGCCTCCCACCA
CIC_5	ACTGTCACTAACCTACTGGT
CIITA_1	AGGTGATGAAGAGACCAGGG
CIITA_2	GAAGAGATTGAGCTCTACTC
CIITA_3	GAGACTGCCAGTCACCACAG
CIITA_4	GAGCTGCCTGAATCTCCCTG
CIITA_5	ACATCAAAGTACCCTACAGG
CIR1_1	ATGTACAGAGAGCACCCAGG
CIR1_2	CTTGCACATGAAGTTGGCGA
CIR1_3	GAGCAGCAGAAGCCACGGCA
CIR1_4	TAGCGGAGATGAGAAACAGT
CIR1_5	AGACCGAATACAAATTTGAA
CIT_1	ACTTCGAAGTCCTTTGCCGA
CIT_2	GAAGAACAGATTCAGCCTGG
CIT_3	GTCATGGAATATCAGCCTGG
CIT_4	TATCTCGAAGCACAAGCCCG
CIT_5	AGACTCGGTGATCCGCTGAT
CITED1_1	CAACCAAGAGATGAGCTCCG
CITED1_2	CCTGCACTTGATGTCAAGGG
CITED1_3	GGTGAAAGATCGCAAAGCAG
CITED1_4	TCAAATGGAACCAAGGCCAG
CITED1_5	AACTTAATAGCCAGTATCAG
CITED2_1	ATTGGGCGGCAGCATTGCAG
CITED2_2	GAGTTGTTAAACCTGGCCGC
CITED2_3	GGAGTTGTTAAACCTGGCCG
CITED2_4	TGGGCGGCAGCATTGCAGCG
CITED2_5	ATGGGCGAGCACATACACTA
CIZ1_1	AACTCACCGGCTGACAGCCA
CIZ1_2	GCAGCATGGAGCCGTTGAGG
CIZ1_3	GCAGTGTCATACGTGGCTGG
CIZ1_4	GGGCCCTGTGAATGTGCCTG
CIZ1_5	ATATCCACAGGTCCACACAC
CLEC4A_1	AAGGTCAGCGACATTGGCAA
CLEC4A_2	AGTGAGGCACAAAGCAGCTT
CLEC4A_3	ATCTTGGCAAGACAGTGAGA
CLEC4A_4	GAAGGACTGTGCTAGAATGG
CLEC4A_5	AGAATGAGATTGCCAATAGC
CLNS1A_1	AACGGGAAGGGCCTCGGCAC
CLNS1A_2	CAGCCAGTATAATATGGCTG
CLNS1A_3	GAGGATTCAGATGACTACGA
CLNS1A_4	TGAGGCTGTGCTGAACGGGA
CLNS1A_5	GTACCCTTTACATCGCTGAG
CLOCK_1	AACACCAACCAAGATCCCGA

CLOCK_2	AAGCAAGGGTACTCACAGGA
CLOCK_3	AATTCCACAAGGCATGTCCC
CLOCK_4	GCGAGGAACAATAGACCCAA
CLOCK_5	CTAGTGAAATTCGACAGGAC
CLPB_1	AAAGGAGCACATCATTGGCC
CLPB_2	GCAATGAACCACCAGCGCTG
CLPB_3	GTTTGGCCGTAGCGATCCGG
CLPB_4	TGAAATGGGACACACACCCT
CLPB_5	AGCAAGAGTCCGTCCAACAA
CLUH_1	CCTGTTTGTGATCACAGCCG
CLUH_2	GGACGCCTACACCTCGAGGC
CLUH_3	GGAGAAGGGCTCGATCCCAG
CLUH_4	TGAGCAGCTCCACTAGGGAA
CLUH_5	ATTCAGGTAAAAGCCCCGTG
CNBP_1	GGAATGAGAAGCCGTGGCAG
CNBP_2	GGTGCCCTGACTCGCCACAG
CNBP_3	TAGAGGTGGCCACATTGCCA
CNBP_4	TAGGACATTCCCGGGCCCAG
CNBP_5	CAAGGATTGTGATCTTCAGG
CNOT3_1	AATGGAACGGTTCAAAGTTG
CNOT3_2	GAGGGACCAAATCAAGACAT
CNOT3_3	GCGGACAAGCGCAAACTCCA
CNOT3_4	TGCCTCAAGAAGGTGTCCGA
CNOT3_5	CATCGACACGCTCAACATGC
CNOT4_1	ACAAAGTGACACCACTGCAG
CNOT4_2	ATGCAAAGAGGGCACTCCAC
CNOT4_3	GAACGGTGATAATTCCCAGC
CNOT4_4	TCAGCGCCTAGCAGACCCAG
CNOT4_5	ATAAACCTACAACAAAGACG
CNOT8_1	ATGATGCCAAGTACTGTGGG
CNOT8_2	ATTGGAAGGCAGCACCAGGC
CNOT8_3	GAGAAGGGAGAGTATCCTTC
CNOT8_4	TAGAGAGGACATGTACTCCC
CNOT8_5	ACTGTAGTCCTGAGTTAGCA
COPS2_1	AAATCCATTTGACTCACAGG
COPS2_2	ATGTCAGATGGATTTACTGC
COPS2_3	CTGATTATGGGAGTTATCAG
COPS2_4	GAACTGGATCATCAGAAGAG
COPS2_5	CCAGTTACATCAGTCGTGCC
COPS5_1	ACAGCAGCAAGAAATCCTGG
COPS5_2	CCAACAAGAACAATATCCGC
COPS5_3	GATGGGTCTGATGCTAGGAA
COPS5_4	TCAGAAGGTCCTTCATCAGG
COPS5_5	AGCAGCCTGAGCATTTACTC
COQ3_1	GACTCTACAGATTAAACCAG
COQ3_2	GCACACACACTCACCCGCCG
COQ3_3	GGGATGAAGATTCTTGACGT
COQ3_4	GTACTGTACAGTCTCGCCCA

COQ3_5	AGCCAGGAAAACCTTTGTTG
COQ5_1	GCAAATCCTTCCAAACACGA
COQ5_2	GCCAAGAGCTACGAAGCCCG
COQ5_3	GGTTGGAAAGCAGAAAGCCT
COQ5_4	TGTGACATTCCGGATCCCAA
COQ5_5	CACGACACGAGACCCGCCCA
CORO1A_1	AATGACAACGTCATTGCCAG
CORO1A_2	ACGAGGCCACCTTGCCACAG
CORO1A_3	GACACGAACACTGCACGCAC
CORO1A_4	GTGACGACGGGCTCCCGCAG
CORO1A_5	CCCAGACACGATCTACAGTG
CORO2A_1	AGTGGTTGTCGTGAACGCTG
CORO2A_2	GAAGAGGATGTTGGCGGCCG
CORO2A_3	GGAGGACGGTCCCTGCTCGG
CORO2A_4	TGTCCACAGGCACATCCCGA
CORO2A_5	CCGATGGAACAACCGGCAGG
CPSF6_1	AAGGAAGATGGCGGACGGCG
CPSF6_2	GGTAAAGCTGGTCCTCCAGG
CPSF6_3	GGTGGTCCAGGAAGATGCGG
CPSF6_4	TCTCAGGAAGCTGAATATGG
CPSF6_5	ATATATTGGAAATCTAACAT
CPSF7_1	AATTGTTGGAACTCCTACCA
CPSF7_2	ATGAAGACAGTCCTTAAGAG
CPSF7_3	GAAGTAGGGCAGCACACTGG
CPSF7_4	GTAGGTATACAGAATTGCAG
CPSF7_5	CCCCACCAAACGCTACAGTG
CRAMP1L_1	AAGAAGCTGTGCGATCCAGA
CRAMP1L_2	AGAAGACCCTAAGTTCCGGG
CRAMP1L_3	AGGCCTGAAGAAGTCATCCC
CRAMP1L_4	GACGCGGCCGAGGAGAGCAG
CRAMP1L_5	CACTCAGCGGAAGACACTCG
CRB2_1	AGAGGCAGCGGAAGCTCCCG
CRB2_2	AGGTGGACGAGTGTGCCTCA
CRB2_3	GCAGTAGCAGCGGAAGCCGG
CRB2_4	GTGTGCGCGCCAGGCTACGG
CRB2_5	AGATGTGGATGAATGCCTGT
CREB1_1	ACAGATTGCCACATTAGCCC
CREB1_2	AGTCCATGGAGTCATTCAGG
CREB1_3	GAAGGGAAATTCTTTCAAGG
CREB1_4	TGGAGTTGGCACCGTTACAG
CREB1_5	AGCTGTACTAGAGTTACGGT
CREB3_1	AAATTCTGAAACGTGTGCGG
CREB3_2	AGCGGCAGCGCCCAGTCCAG
CREB3_3	GAACAAAGTACAGCTTCTGG
CREB3_4	TCCATAGAGACAGTTTCCCG
CREB3_5	AACAAATTCTGAAACGTGTG
CREB3L1_1	AAGATGGAGGACACCACCCA
CREB3L1_2	AGAGCATGGAGCATGGGCGC

CREB3L1_3	AGTCCAGGAAGCTGGATCCG
CREB3L1_4	CAGCTGAGTCATCTCTCCCG
CREB3L1_5	AAGCGGACCCTGATTGCTGA
CREB3L2_1	GAAAGAATACATGGACAGCC
CREB3L2_2	GACAGACGGAACGAGTCCTG
CREB3L2_3	GAGCGAGCTGTCAGAGCCCG
CREB3L2_4	GTGAAGGGCGACTGGGCCCG
CREB3L2_5	AGGCCCTGAAGAAAATTCGG
CREB3L3_1	AAATGTGGAGCCCAGGAGGA
CREB3L3_2	AGACACGTGGAGCTGGGCGA
CREB3L3_3	TCAGGAGCTGGTGCTCACCG
CREB3L3_4	TGAGGAAGTCGTCAGAGTCG
CREB3L3_5	AGTTGCCAGGATGATAGGAG
CREB3L4_1	CAGCTCAGGACAGTCGGCGG
CREB3L4_2	GAAGCATGTTACCTTGGTGA
CREB3L4_3	TAGGAGAACTGGTTGCCCTG
CREB3L4_4	TTACCGGAACCTCTGGAGGG
CREB3L4_5	GGAAGCATGTTACCTTGGTG
CREB5_1	AAATGGGAACATGAACACCA
CREB5_2	ACAGAGCTGGACTGAGGCGA
CREB5_3	ACTTACTGGCATCAAGACGG
CREB5_4	CCTGGAGCACGAGTTCAGGA
CREB5_5	AATGGGAACATGAACACCAT
CREBBP_1	ACGAGAGCAAGCAAACGGAG
CREBBP_2	GAGTTCCTTCACCTACCCAG
CREBBP_3	GCTCAGAGGGCTCTTGCCCA
CREBBP_4	TTTGTCGTGAAGATGCACAA
CREBBP_5	ATTGCCCCCCTCCAAACACG
CREBL2_1	CAAGCAGTGGTGCATGGCAA
CREBL2_2	GAAATAAAGGCCCTACTCAC
CREBL2_3	GAACTCAAGCAGGCATACCA
CREBL2_4	GGAGGCAAAGTAAAGAAGCC
CREBL2_5	ACATATAGCTCTTTCTCGAC
CREBRF_1	GAAGTTCATTGCCTATCTGG
CREBRF_2	GATGAGAGAGGACCCAACAT
CREBRF_3	GGAGTGGCATGACCTTGAAG
CREBRF_4	GGATGCTGAGACCATCTGAG
CREBRF_5	AGTGATACTTTCTCTGAACC
CREBZF_1	CGTTGGCTAAGACTGCCCGT
CREBZF_2	GAAGAAGAAGGAGTACGTGA
CREBZF_3	GAAGAGGACGACTCGGCGGG
CREBZF_4	GCTGGAAGAGGACGACTCGG
CREBZF_5	AGAAGAAGGAGTACGTGATG
CREG1_1	GAGCGAGAGGACGTCGGCGA
CREG1_2	GCCATGGCCGGGCTATCCCG
CREG1_3	GCTGTCAGGAACTGTGACCA
CREG1_4	TCTGGCCACCATCTCCACGC
CREG1_5	AGCGAGAGGACGTCGGCGAA

CREM_1	GCAGCCATCACCACTCCCTG
CREM_2	GCATATATCAGACTAGCACG
CREM_3	GGGAACACCAGGCACATCAG
CREM_4	TAAGTTGCTATAGCCCAAGG
CREM_5	ACAATCCAGATTTCTAACCC
CRIP2_1	AGCAAGACGCTGACGCCCGG
CRIP2_2	CGAAGGCAAGGTCCAGCCCT
CRIP2_3	GATGTAGCTGCCCACCGCAC
CRIP2_4	GCAGCAAGACGCTGACGCCC
CRIP2_5	ACCTTTGGGTCCGAACAGGG
CRNKL1_1	ACAATGGGAAGAAAGCCTAA
CRNKL1_2	AGACATGGCGGCCTCCACCG
CRNKL1_3	AGTTATCTGTACCTCAGCCG
CRNKL1_4	TGAGCTGAGATACAAAGAGG
CRNKL1_5	ACTACCGAAATATTACACTC
CRX_1	GAAATTCACCTACAATCCCA
CRX_2	GCTCGGAGACCCATAGGCGG
CRX_3	GGCCAGGGAAGGTCCCACGG
CRX_4	TAGGAATCTGAGATGCCCAG
CRX_5	AGGGTCTGGACACACATCTG
CRY1_1	ACCAATGGTGAACCATGCTG
CRY1_2	GGTAGGAGAACAGATCCCAA
CRY1_3	TGAGGCTTTAGCCAAATGGG
CRY1_4	TTGGAGGAGCCGGCGAACCA
CRY1_5	AGGATCATAGAACTCAATGG
CRY2_1	AGCCATCATGAAGATGGCCA
CRY2_2	CATGGCGGCGACTGTGGCGA
CRY2_3	GAACAGCCTTGGGAACACGT
CRY2_4	GAGAACCACGACGAGACCTA
CRY2_5	AAGCGCTTGTATGTAAGGGG
CSNK2A1_1	ACACCTACTATACTCACCAC
CSNK2A1_2	CACCTACTATACTCACCACA
CSNK2A1_3	GAACTGACCTGACATCATAT
CSNK2A1_4	AGACATTGTAAAAGACCCTG
CSNK2A1_5	AGTCACATGTGGTGGAATGG
CSNK2A3_1	AGAAATCCCTGACATCACAT
CSNK2A3_2	GAAAGCTACGACTAATAGAC
CSNK2A3_3	GTATGAGATTCTGAAGGCCC
CSNK2A3_4	ACGTGACAATTATGATCAGT
CSNK2A3_5	AGGCGGGGGTTCGTGACACA
CSNK2B_1	AACCAGCCAATGCTTCCCAT
CSNK2B_2	GAAGATGAGCAGCTCAGAGG
CSNK2B_3	GGTGGATGAAGACTACATCC
CSNK2B_4	TCAAGACACCATCACACGGA
CSNK2B_5	CAGTTGGAAAAGTACCAGCA
CSRNP1_1	ACTCAGCCAAAGAGAAGCGA
CSRNP1_2	GGAGCGAGACTGGCACCCAG
CSRNP1_3	TGACAGCCTGGACAACATCG

CSRNP1_4	TGAGGGCTTCAGGTGTGCGA
CSRNP1_5	AGAAGCGACGGCAAGCACTG
CSRNP2_1	AAGGCAGAAGAAGATTCCAG
CSRNP2_2	GCTCACGCAGAATCTCTCGA
CSRNP2_3	TCCGTACAGAGTTATGGCGC
CSRNP2_4	TCTCTTCAGCATCAATACGG
CSRNP2_5	GAAGAATGTACGCTTTGACC
CSRNP3_1	CATTGACCTGGACAACACAG
CSRNP3_2	GAAGAGGGACAACTTCGGCA
CSRNP3_3	GCAGTGAAAGTGCTGACAGT
CSRNP3_4	GTTCTCTCAACATCTCCCGG
CSRNP3_5	ACAAGTGTGCCCAGTCAAGG
CSRP2BP_1	AATTTGGAGAGCCAGGATGG
CSRP2BP_2	GAAGCTACTGACCCAGCAGG
CSRP2BP_3	GAAGGAGATGGGCTTGTCAG
CSRP2BP_4	GACGTCAAGGTTATTTCAGG
CSRP2BP_5	AGTCCCTCAACAGTAATGAT
CSTF1_1	AAAGAGTTCGAATCACTGGG
CSTF1_2	AGATGCAGGAGCTGCTCCGA
CSTF1_3	CCAGGTCAATCCCTGTGCCA
CSTF1_4	GGGACTCGAGGACAGCCGAG
CSTF1_5	ACAAGTATAAAGTCTCCAGA
CSTL1_1	CAGGGCAATCAGCAGCAGCA
CSTL1_2	GAAATAGTTTATCCAGGGCA
CSTL1_3	GCTGGGTCACTTCCAAAGGT
CSTL1_4	TCGGCAGCTGACGACGGGAG
CSTL1_5	CCTACTTATATCGAGTCCAG
CTBP1_1	AATCCGGACGATGATGCGGA
CTBP1_2	ACCAAGTCCGATGATGCCCA
CTBP1_3	GAAGAGCACGTTGAAGCCGA
CTBP1_4	GGATGGCCGGGACTGCACAG
CTBP1_5	GATGTGGCACAGCGTCGAGT
CTBP2_1	AGGGTGGGCCCACCTTCTCG
CTBP2_2	CAGGTTCTAAACGAAGCCGT
CTBP2_3	GACGCAGGAAATCCACGAGA
CTBP2_4	GGACGGCCGCGACTGCACTG
CTBP2_5	ACATCCTCAACCTGTACCGG
CTCF_1	GACATGGCCTTTGTGACCAG
CTCF_2	GGCGGAAGGTCTTATCGCAG
CTCF_3	GTTAAGGTGATTCCTCAGGA
CTCF_4	TTTGAGGAAGAACAGCAGGA
CTCF_5	CGATCCAAATTTGAACGCCG
CTCFL_1	AACATGGCATTTGTCACCAG
CTCFL_2	CAAGAGGATCAACCTACAGC
CTCFL_3	TGAACATGCAGACATCACAG
CTCFL_4	TGTGCAGAGAGAAAGACCAT
CTCFL_5	CAAGTGTAACGACTGCAACA
CTNNB1_1	AAGGTTATGCAAGGTCCCAG

CTNNB1_2	ATGGCAGTGCGTTTAGCTGG
CTNNB1_3	GAAGCTGAACAAGAGTCCCA
CTNNB1_4	GATGGAGTTGGACATGGCCA
CTNNB1_5	ATACTTACGAAAAACTACTG
CTNNBL1_1	AAGCAAATCGACCACAGCTA
CTNNBL1_2	AGACCTGTACCACCTTCTGG
CTNNBL1_3	AGATGGCGTCCACAACACTC
CTNNBL1_4	TCGGGAAGAAGAAATGACTG
CTNNBL1_5	AGCTCGGATTCCATGAACCT
CTR9_1	AAAGCATTGCGTACTAACCC
CTR9_2	TAGCTGGAATACTACAAGCA
CTR9_3	TGACTTCGATCAGTTACCGG
CTR9_4	TTACCTCGTCAGTGTCCCGG
CTR9_5	AGAAAAACGAGATATTGCCA
CUL1_1	CACCAGTCAAACCAAGCACG
CUL1_2	GTATCGAGCCAGCAACTCAG
CUL1_3	GTTCGCCGTGAATGTGACGA
CUL1_4	TTTCAGTTCCAAGAACCCAG
CUL1_5	AACCCAATGTCTATTGAGGT
CUL2_1	ACAGAAGCGGACCTTCAGTA
CUL2_2	CCAAACCAGAAAGTAATCCA
CUL2_3	GACGTATTCCAACATGACCA
CUL2_4	TACTGGGAAGAATACAGCAA
CUL2_5	AGATATCTATGCTTTATGTG
CUL3_1	ACCATGTCGAATCTGAGCAA
CUL3_2	ATCCAGCGTAAGAATAACAG
CUL3_3	GAGCATCTCAAACACAACGA
CUL3_4	GGAAGAACCAATTGTAAAGG
CUL3_5	ATAACTTGTACATGCAACCA
CUL4A_1	CAACGGCCTCACCAAGCCCG
CUL4A_2	GAGGACACTCACCAGATGGA
CUL4A_3	GCTGCCCGACAACTACACGC
CUL4A_4	GGTCAGACAGCATGCCCAGG
CUL4A_5	AGTTCTGCAGCACATAGGTG
CUL4B_1	ACACTGCAGTCATTAGCCTG
CUL4B_2	AGCATGTGGTACTTACTGGG
CUL4B_3	ATCCAGCAATTCTTGAACCA
CUL4B_4	GCTGTTAGCAAAGCTAGAGA
CUL4B_5	CTCTTCAGTAGAGTTCGAGG
CUL5_1	ACTATCTTAGCTGAGTGCCA
CUL5_2	ATAGGAAGTTGGTATGCCAG
CUL5_3	GAGGAACATATCATTAGTGC
CUL5_4	TCACCCAAAGACTTTACAGA
CUL5_5	AGCTTGTTTACATAATCCGC
CUX1_1	GATGTCCACCACCTCAAAGC
CUX1_2	GCTGGCCTCACAGATCCAGA
CUX1_3	GGAGCGGGAGATCGCACAGC
CUX1_4	GTTCAAGAAGAACACTCCAG

CUX1_5	ATCTCCAACAGCGACCTGAG
CUX2_1	AGAAGTACAGATCACTTTGG
CUX2_2	GAAGTGTGGAGGTCTCGCCG
CUX2_3	GCAGCAAGGTTTCTGTCAGG
CUX2_4	TAAGAGAAGTCAGGAGGCGG
CUX2_5	CCTTAGTAAGAGAAGTCAGG
CXXC1_1	AGCGGGACAGCAGTGAGCCC
CXXC1_2	GATCACTGAGAAGATGGCCA
CXXC1_3	GTATCAGGACTTCTGTGCAG
CXXC1_4	TAAATGGAAACACCCAGAGA
CXXC1_5	AAACGGTCAGCCCGCATGTG
CXXC4_1	AACACTGCAGACCTTTGGCG
CXXC4_2	AAGGAAAGCCACTTGCCCGA
CXXC4_3	ACACTGCAGACCTTTGGCGG
CXXC4_4	CAAGGAAAGCCACTTGCCCG
CXXC4_5	AAAAAGTCCCCACGCGCTCG
CXXC5_1	ACCATGTCGAGCCTCGGCGG
CXXC5_2	CAATGGGCATGACCTGGCGG
CXXC5_3	CCAGAAGGTGATGCTTCCGA
CXXC5_4	CCGTCACTGAAACCACCGGA
CXXC5_5	GCATCTCCCGCTGATGAGCG
DACH1_1	CATGATGTGAGAGTTCTCTG
DACH1_2	GAAGAGCTCTCAGTCCGAGC
DACH1_3	GATGTCTCAACTCTGGATGG
DACH1_4	GCGGCTGGAGATCACGCCGG
DACH1_5	ACAATGACTGCACCAACGCA
DACH2_1	AAAGATCAAAGACTTGCGGC
DACH2_2	AGGTATAACAGCTGCAGCGA
DACH2_3	GCCATGGCAACTGATGCAGG
DACH2_4	TAGAAGAGAATCATCGTCCT
DACH2_5	ACTCCCAAAGAACGCTTAGG
DAPK3_1	CATCGCGCACAAGATCGAGG
DAPK3_2	CGTGAACTACGACTTCGACG
DAPK3_3	GGAGAAGGAGTCGCTGACGG
DAPK3_4	GTTCAGGCAGGAGGACGTGG
DAPK3_5	CTCCTTCCTAGGCGATCCGG
DAXX_1	AAGAGAAGAGCTCGGCTCCA
DAXX_2	CACGATGATGCTGTTAGCGG
DAXX_3	CTAGTGGAATGGCATCCCAG
DAXX_4	GATGTTGCAGAACTCCGCCG
DAXX_5	AATGTTGCAAGACAAAAGTG
DBP_1	AGAAGGAGAACGCCCTGCTG
DBP_2	CAGTGGCGCCAACAGACCCG
DBP_3	GGTTGCGGAGCCTTCTGCAG
DBP_4	TCGGGACACACCCAGCCCTG
DBP_5	ACGTACTCCACATCGCCGAA
DBX1_1	ACGCGTGGTAGGCCAGGCGG
DBX1_2	CACGGCCCTCACCGACACGG

DBX1_3	GAAAGGCGAAGGTCTTGGGA
DBX1_4	GCGGCCAGGAGAAGGTCCCG
DBX1_5	AAATAAGAAGATCTGATGAA
DBX2_1	AAAGCCCACCGCGTTCCGTA
DBX2_2	ACTCTAATTCCAAAGCTCGG
DBX2_3	ATGGCGGAATTCCAAAGAAA
DBX2_4	GCACGCCGAGTAGAATGGCG
DBX2_5	AAGGCTGGAAGGTACAGTCT
DCLRE1A_1	AAAGGCATCAACTGTAAAGC
DCLRE1A_2	ACAATACATTCAGTGTCCAG
DCLRE1A_3	GAAGAAGATGATGACAGCTG
DCLRE1A_4	TGATAGGGCAGACACCTCGA
DCLRE1A_5	ACGACATGGTCCCTTACTGA
DCTD_1	AACGGGACGACTATTTGGAA
DCTD_2	AAGGACAGCAGAGAATAAGC
DCTD_3	GCTCCTGTTTAATATGGCCG
DCTD_4	GTGACGAGGCAACTGCTGCG
DCTD_5	CATACTACAGCCTTTCACAT
DDB1_1	ACCTCACCTGGACATTGCCA
DDB1_2	GAGTCAATCACCTATCACAA
DDB1_3	GATCTATGTGGTCACCGCCG
DDB1_4	GGTCACGCAGCCGTTCACGG
DDB1_5	CATTGTCGATATGTGCGTGG
DDB2_1	AAAACTGGTTGGTATTGAGA
DDB2_2	ATGTAGCCCTCCTGTCAAAG
DDB2_3	CAAAAAGAAAGTGACGCATG
DDB2_4	TGGTCACAGGAGACAACGTG
DDB2_5	CCCAACTCACCCCAGCACCG
DDIT3_1	GGAAATCGAGCGCCTGACCA
DDIT3_2	GGAGCTGGAAGCCTGGTATG
DDIT3_3	TCAGCCAAGCCAGAGAAGCA
DDIT3_4	TCTGGCTTGGCTGACTGAGG
DDIT3_5	ATTTCCAGGAGGTGAAACAT
DDX1_1	AATGTAACCATCCGGTGCCT
DDX1_2	ACTCTGAAAGACCAACAGGA
DDX1_3	TGTTCAGCTAACTCCCGGGA
DDX1_4	TTTCCATCAGAGGTAACCTG
DDX1_5	ACACTTACCTAGGTCCAAAG
DDX10_1	ACCTTGCAAAGCCAATCCAA
DDX10_2	CAGCGATTGAAGCTCCGCAC
DDX10_3	GAAGAACCCAATTCACGGCC
DDX10_4	GCTACTGATATTGCAGCCAG
DDX10_5	AGTGCAAGGATAGAAACACC
DDX11_1	AAAGTCACGGAGCCAAGAGA
DDX11_2	CAGCCGAACATCCTTGCCAA
DDX11_3	TGATAAGCTGCACAGAACCT
DDX11_4	TGTAGGCGGAGCAGGCCAGG
DDX11_5	GCTTATCAACGACCGCTGTG

DDX17_1	CTTTCCAAGTCTCGAATCTG
DDX17_2	GATTACACCCAGATCAACGT
DDX17_3	GCTGTGATGATCTGACTCGA
DDX17_4	GGTCTGGTGACGACCGATGG
DDX17_5	GCATGATGGAAGGCAAACAC
DDX18_1	AAGGATACTCAGATCAACGA
DDX18_2	GACGTTACCTGTCAGTCCCA
DDX18_3	GTTACCTTAGGGTCATCCGG
DDX18_4	TACGCAGGAGTTTCATCGGC
DDX18_5	AAACAAAAGCCCATGAATGT
DDX19A_1	ACTGGGCAATCAGATTCTGT
DDX19A_2	GCTGAGCATGGCTAAGACAA
DDX19A_3	GGAGGGTCGATTGAAGCCCA
DDX19A_4	TATGAGCTGGCGCTTCAAAC
DDX19A_5	AAAGTCAAAGCAGATACCAA
DDX19B_1	GATTGGGCAATTAAGTTCTG
DDX19B_2	GGACCATGGCCACTGACTCA
DDX19B_3	GGAGGGCGAGCTCATACGTT
DDX19B_4	TGGAGGGCGAGCTCATACGT
DDX19B_5	AAAATCAAACCAGATACCAA
DDX20_1	ACAGTGACCTACTGTTGCCG
DDX20_2	ATTGGGAGACATACATGCAT
DDX20_3	TAAAGAGGCGTATACTGCCT
DDX20_4	TGACGGCGCGGCTACCATGG
DDX20_5	AAGCAGCAGTGACTCGAAGT
DDX21_1	GAAATAGGAAGGTCACTCCT
DDX21_2	GGACAGCGGGAAGGCAGTCG
DDX21_3	TAAGTGCCACTGGACTCAGA
DDX21_4	TATGGTGGAACTCCCTATGG
DDX21_5	CAACATTAAATACCCAATGA
DDX23_1	AGGCTGAAGCTCTAAAGCGA
DDX23_2	TCAAGGGATAGACGTCGAGG
DDX23_3	TCAGGCCCAGCCATTATCCC
DDX23_4	TGCGGTCTTCCAGAGAACGA
DDX23_5	ACCGCTAGGTATCCGCACTG
DDX24_1	ACCTGGAGAGACCAGAACTG
DDX24_2	ACTGGAGGGATTCTTGGCAG
DDX24_3	CAACCACAATCTCAGGACGA
DDX24_4	TGTTGGCAAACACTAAGCTG
DDX24_5	CATGGTTTGTGATGATCCGG
DDX25_1	ACGGTGACTGGATTCTACTA
DDX25_2	CATAAGTAGGAGCTAGGCAG
DDX25_3	GATGTGATGATTGACACTCA
DDX25_4	GTTAAGCAGAGTTAATGCCT
DDX25_5	CAGTACTCTTAATACCCCAA
DDX27_1	AAAGGAAGGCTCTGAACCAA
DDX27_2	AAAGTTAAGGGCCGGACCGC
DDX27_3	CAGGGTTGAAATCAGCACTG

DDX27_4	GAAGCTGCTACCTTCAGAAG
DDX27_5	CGGATCCGGCCTAATCGTGA
DDX28_1	AACACCAGAACAGTTCCTGA
DDX28_2	AAGGAACAAGGACCAGGCCT
DDX28_3	GTAGGAGCCACATTTCCCGA
DDX28_4	TGTGCTTGTGGCCACTCCAG
DDX28_5	AGGGAGGCCACGGCATGCGT
DDX31_1	GGGAACAACGCAGCAAGCAA
DDX31_2	GTTGTCGAAGAGCGAACCGT
DDX31_3	TCTCGTTGGCACGAGCACCA
DDX31_4	TCTTGCCTATTGCATCCCTG
DDX31_5	AGGTACTTACTAAATGTGGG
DDX39A_1	GGAAGGCCAGCTCCCTCGTG
DDX39A_2	GGCACAGGACGTCCATGCCC
DDX39A_3	TAGGTGTCTGTGTTCTTCGG
DDX39A_4	TGTGTGCAGGAAGTTCATGC
DDX39A_5	CCCCCTAAGAAAGACATCAA
DDX39B_1	GAGGCTCTTATTTCGAGCCA
DDX39B_2	GCAGTACTACGTGAAACTGA
DDX39B_3	GCTACCAACCTATTTGGCCG
DDX39B_4	TCTTTGTGAAGTCTGTGCAG
DDX39B_5	CCTGCCAAGAAGGATGTCAA
DDX3X_1	GTTGGCAGTACAGATCTACG
DDX3X_2	TACGATGGCATTGGCAGCCG
DDX3X_3	TCCATGGAGACCGTTCTCAG
DDX3X_4	TCTTCAGATAATCAGAGTGG
DDX3X_5	CGTGGACGGAGTGATTACGA
DDX3Y_1	AACGGAGTGGACATAGTCGT
DDX3Y_2	AATCGTGAAAGACCTGGCTT
DDX3Y_3	ATGTGGAGGACAGTTACTGC
DDX3Y_4	GGTGGTTACGGCAACAGCAG
DDX3Y_5	AGGACGAACTCTAGATCGGT
DDX4_1	AAAGTGGATTTGCCTCTGGG
DDX4_2	GATAATCCAACACGGAACAG
DDX4_3	GCGAGATAATACATCCACAA
DDX4_4	GGTGGAAGAGGTAGTTTCCG
DDX4_5	ACACTGACCAGAATTGCTGG
DDX41_1	AAGACGCAAGGGAGCTGCGG
DDX41_2	AGCGCGCAAAGAGTCTGCCA
DDX41_3	GGAGTCGGAACCCGAACGGA
DDX41_4	TGCCGGAGGAAGCCGCTCCG
DDX41_5	AGCCTGTGACCATCAATGTG
DDX42_1	AAGGAGTTGGAACCAGGTGA
DDX42_2	AAGGCACTGTGGGACTGCTG
DDX42_3	CAGGCCAAGGCCCTTCAGGA
DDX42_4	GATCTGCTTCATCAAACACA
DDX42_5	AGTGATTGTGTGTCCTACCA
DDX46_1	AGAGTGGTCTGTGTTTACGG

DDX46_2	GACATGTTAGCCGCTAACAG
DDX46_3	GGAAATTCCACACTGGACCC
DDX46_4	GGAGAATGACCAGGATGCCA
DDX46_5	CCAAAGCCTCCATAGCTCTG
DDX47_1	ACTAGGGCAAACAAACGCTG
DDX47_2	GAGGAAACGGCACATTTCAC
DDX47_3	GCAACTGACGTTGCCAGCCG
DDX47_4	GGAGACCTCACACAAGATGG
DDX47_5	AACTAAAACATTTAAAGACC
DDX49_1	AAAGACTGCATCATCGTCGG
DDX49_2	ACGTGGTCATCGCCACGCCG
DDX49_3	GACGAGGCAGAAGATGCCAT
DDX49_4	GCCATACTCACCCTCCAGGA
DDX49_5	AGCCCAAGCAGTCTCGACCT
DDX5_1	AAAGAAGTTTGGAAACCCTG
DDX5_2	AAGTCTACTTGTATCTACGG
DDX5_3	CTTGTCACCATGGATACCCA
DDX5_4	TATTCGAGTGACCGAGACCG
DDX5_5	CATTCCTAGAGAGAGGCGAT
DDX50_1	AGTTCAGAAAGCATACCTGA
DDX50_2	ATGCCTGGGAAACTCCTCTG
DDX50_3	TACCTGTGCGTCCAGAGCGA
DDX50_4	TATAACTAGGAAACTCAGCG
DDX50_5	AAGATTTAATAGCTCAAGCA
DDX51_1	AAGCAACGAGGAGGCGCCAG
DDX51_2	ACTGGCCTTCGTCATCCCTG
DDX51_3	CGCGACGTAGAACAGCGCCA
DDX51_4	GGTCTGCCACATCCGTGCCC
DDX51_5	ACGTCAGGGATGTCCTCGAT
DDX54_1	AAAGGGCTCCGGAAGCGCCG
DDX54_2	AGGTGCCAACACCCATCCAG
DDX54_3	GCAGACCCTGAAGTTCACTA
DDX54_4	GGTGTCCGGCTCCACATCCG
DDX54_5	CAGGTAGGGGATTTCATCAG
DDX55_1	GAATGGCCAGCTCTCGAGTG
DDX55_2	GCTTGGAGGACATGTTCCGG
DDX55_3	GTTTCGCATGAACAGAGGGA
DDX55_4	TCTCAGAAGAATTTCCAGGA
DDX55_5	GCGACCGCAGCGATGCACGA
DDX56_1	AAGTGGCTTAATATGCGAGA
DDX56_2	ATTCTTACAGACAGGTCCGG
DDX56_3	CCAGGAGAAGGCCATCCCAC
DDX56_4	GTGGCAGACCTGGAGCGCAG
DDX56_5	ATCATCTCACAGTTCAACCA
DDX58_1	AAACAACAAGGGCCCAATGG
DDX58_2	ACTCACCCTCCCTAAACCAG
DDX58_3	ATGGCAATAGGCTTACCTGT
DDX58_4	GGAACAAGTTCAGTGAACTG

DDX58_5	CATCTTAAAAAATTCCCACA
DDX59_1	TATATCCAGAAGTCGCCCAG
DDX59_2	TATGAAGTTGTAGTGAGCAC
DDX59_3	TCAAATGCAGATGATTCCTG
DDX59_4	TGTTGCAGACGATAAAGCTG
DDX59_5	AGTTGTGATTTAAGACCTCA
DDX6_1	CAGGTCAGCAAACACATGGG
DDX6_2	TAAGATCCAGGATTCTCCCA
DDX6_3	TGAACGGCTAGACCTGAAGA
DDX6_4	TTTGAAACGGGAGTTACTGA
DDX6_5	ACGTGGTGATTGCTACCCCT
DDX60_1	AAGAAATGGAGGTTCTGCCC
DDX60_2	CTAGAGTCATCACTTCCCAT
DDX60_3	GCACAGATCTAAGAACACGA
DDX60_4	GCTGAAGGAGAGCGACGACG
DDX60_5	CTGTTGTTAGTGCAGCACAT
DEAF1_1	AAACTCACAGGTGGTAGCCG
DEAF1_2	AGTGACAGTGGCCAACGTGG
DEAF1_3	GATGGAGGACTCGGACTCGG
DEAF1_4	GGGACGGTGCATCAAGCAGG
DEAF1_5	AAGGCACAAAAAGCCTGACT
DEK_1	AAACTGCTTTACAACAGGCC
DEK_2	AAGAATGTGGGTCAGTTCAG
DEK_3	GAGCCATTTACAATTGCACA
DEK_4	TCCCAGAGAGGAGAGCGAGG
DEK_5	AAGTGTCCAATATAAAAAGA
DENND4A_1	ACTTTGCAGAACGTGTGTGG
DENND4A_2	GAACATACGGGCATGGCCAG
DENND4A_3	TAAACCACAGTCCATAACAG
DENND4A_4	TAGTAAGCACGGATGGCCGT
DENND4A_5	AAACCGTAATCTTAGTGGAG
DGCR8_1	ATAGAGTCAGGCTCATCCAG
DGCR8_2	GAATGTCATGATCCACCCGT
DGCR8_3	GACAGCGACCATCCGTCCGA
DGCR8_4	GAGGAGGCGGCCCTTAGAGA
DGCR8_5	AGACAAGGGATGCTACTCAG
DHX15_1	AGAGTGCGAGGATGTCCAAG
DHX15_2	ATGTACTCCACACACCACTG
DHX15_3	GAATGGGAAGCTTTCAAAGG
DHX15_4	TACACCATAACGCTCCAGGA
DHX15_5	CGCCCAGGAATAGTTAGGAG
DHX16_1	ACAGCGCTGCACCTCTGCCG
DHX16_2	AGATCGGTTCTTCTCCAGCA
DHX16_3	CAATCGCTACCACATGCCCA
DHX16_4	GAAGCGCCTCAAGATGGCCG
DHX16_5	CTCGAAATGGGAACACCGGG
DHX29_1	AAAGGGCTAAAGTAGCTCCC
DHX29_2	CAAGAAACACAAGGCTCCAG

DHX29_3	GTTTAAACAGAGCCAATCCA
DHX29_4	TGACAGCTTACCCTTCCTCC
DHX29_5	AATTCTACAAATGATTCTAG
DHX30_1	ACGCCACCAGCTGTCGGCAG
DHX30_2	CTGGCCAGAAATGTTCAGCC
DHX30_3	GACCCAACTCACCTTAGCTG
DHX30_4	GTAGGGTTGACACACATGGG
DHX30_5	AATCCAGAGAGTATTCGACC
DHX32_1	GAATCCCTGGATTCCAGCGA
DHX32_2	GTACCAACGAAACAATCCTG
DHX32_3	GTGATTGAGTAACCAGACAG
DHX32_4	TAAGGTACACAACCTCCACA
DHX32_5	AGTCAGATTTGTTATCGATG
DHX33_1	CTGCTACCGGCTCTACACGG
DHX33_2	GAGATGGCAGCTACTCGACG
DHX33_3	GCAGCTCCGAGTCTTCCAAG
DHX33_4	TCAGGGTAGGGACTGGCCGA
DHX33_5	AAAGCAAAGAAGTATAACCC
DHX34_1	ACTGGGCAGGCACTTGCCCG
DHX34_2	AGTCAGAGAAGCTGGACCCG
DHX34_3	GAAGAGCGAGATGTTGATGG
DHX34_4	GATGCATTTCCGGACTCCAG
DHX34_5	CCCCGTCCCAGAAATTCGGA
DHX35_1	AGTGTACAAGGTCCTCTCGT
DHX35_2	GAAGAGCACACAGATTCCTC
DHX35_3	GGATGCAGTAGCCCACCTCG
DHX35_4	TACATGTGTGATCCTTACAG
DHX35_5	AAGGCAGTCCTGCATACATG
DHX36_1	AGGGCAGAATCTTGTGGCAG
DHX36_2	CCTGCATTTCAATATACCGA
DHX36_3	TATGACTACCATCAGAACTG
DHX36_4	TCATTCACAGTCGGTTGCCA
DHX36_5	AATGAACTGAGTAACTTGAG
DHX37_1	ACAGCGGGAGCACGTGGAGC
DHX37_2	GAAGTCCTCCACCCGCAGCG
DHX37_3	GTATGAAGGAAACGTGACAG
DHX37_4	TTAGCCAGGGCCCTGGGCAG
DHX37_5	CGAAGAACAAGTAATCATGG
DHX38_1	AGAGAGCAAGGATGACCAGA
DHX38_2	GAGACTCCATCCCATCCGGG
DHX38_3	GCTCCTCAAGGCGCTCACAG
DHX38_4	TTGTAGGAGGGAGTTGGCAG
DHX38_5	ATCGAGACTATGACCGCAAG
DHX40_1	AATAGCAGCAATGTTGTCTG
DHX40_2	AGCGAGGCATTACAGCGAAG
DHX40_3	ATTAGGGTTGGACATCCTGG
DHX40_4	GAAAGGATGCACGTCCCAGG
DHX40_5	AAATACTGCGTATATTCAAG

DHX57_1	ACTGGCAGAAAGTTCACAGG
DHX57_2	GAGAGGGTGGGTCTGACCGT
DHX57_3	TCTCATGGGAGTGGTGGCGG
DHX57_4	TGGAACATAAGGCTCCACTG
DHX57_5	ATATGTAAACGACAAGCCAG
DHX58_1	GATGCACCACGTGTCCAAGT
DHX58_2	GTTGACCAATACAACCACCT
DHX58_3	TCAACTCAGGCATCTCCAGG
DHX58_4	TGAGTGCCACCACACGCACA
DHX58_5	ACCTAGAACTTAAACTCCAG
DHX8_1	ACCGGGACAAATATGGAGAG
DHX8_2	GTTTGGCAAGTTCTTCCGCG
DHX8_3	TAGACGGCGAAATCTTGTCG
DHX8_4	TGGCCACACGACCCTCCCGC
DHX8_5	CCCAGGTCGAACATATCCAG
DHX9_1	AAGCTTCAACCACTCCAAGA
DHX9_2	GATGCTCTAAATCTTGAGAG
DHX9_3	TCAATGTTGCTACTAGTCCA
DHX9_4	TCAGGAGTATCAGTAAGTGG
DHX9_5	CAAAACATTATACTGGCATG
DICER1_1	AAATGGGAAATGTGATCCAG
DICER1_2	GCTGCAGGCCTGCCATGCTG
DICER1_3	TCAGCCATGTGAGATTGTGG
DICER1_4	TCAGCCTCGCAACAAACAGA
DICER1_5	GCAGTTAGTCCCAAAATGCG
DIDO1_1	CATGAAACCATTCTTCACAG
DIDO1_2	GACGCCGAGACAGCCTCCGA
DIDO1_3	GCGCCACGTGACAGCACCCG
DIDO1_4	GCGGAGTGAAGCACTCGGGA
DIDO1_5	AGCTGAAGCACCACTCGATG
DLX1_1	AAGGGCTCAGGAGGAAACGC
DLX1_2	AGTGCATGGAGTAGTGCCCG
DLX1_3	GCGGGCCATTCGCAGCCCGA
DLX1_4	GGACTCGGAGAAGAGCACGG
DLX1_5	AGTTCGGTGCAGTCCTACCC
DLX2_1	AGCGTAGGAGGTGTAGGCGG
DLX2_2	CAGGTGTGAGGCGGATCCCG
DLX2_3	GGAAGGGTGGGCGACTCCTG
DLX2_4	TTCGCCGCCAGTCTCAGCGC
DLX2_5	CCTGAAATTCGGATAGTGAA
DLX3_1	AGTGACAGAAGACTCGGGCA
DLX3_2	GAGGCACTGTATGGGAGCGG
DLX3_3	GGTGAATGGGAAGCCCAAGA
DLX3_4	TCACCGTCTGGCCATAGGGC
DLX3_5	ATACGCCCCGTATTGCCGGT
DLX4_1	ACACAGAGAAGGTCCTCCCA
DLX4_2	CCTGTCCTGCCAGCAACCCG
DLX4_3	GGGTAGGCAGCCGCTACCGA

DLX4_4	TCTGGGATCTACCCAAGGCA
DLX4_5	ACTGCGTGTGCTGGAAACGC
DLX5_1	GAAAGAAGTGACCGAGCCCG
DLX5_2	GGAGCTGGGACTGTGCTCCG
DLX5_3	TGACTACTACAGCCCTACGG
DLX5_4	TTTGGCTGGGTAGCTCCCGG
DLX5_5	AGACGGATGGTGCATAGCTG
DLX6_1	ACTGTGTCTGCTGAAAGCGA
DLX6_2	CATACTGACACCCTTGGCCG
DLX6_3	GATGATGACCATGACTACGA
DLX6_4	GCACTGCCTGCACTCGGCGG
DLX6_5	AGGCTGGAATAAATGGTCCG
DMAP1_1	ACTGACTTTCAAGAGGCCCG
DMAP1_2	ATGCTGGGCACGAACGACGG
DMAP1_3	CCTCCGCTGCACGTCGCCAG
DMAP1_4	CGGGACATTCTAGAACTCGG
DMAP1_5	CATGGATAACAACAAAACGC
DMBX1_1	TAAGCGCATGCACTGAAGGC
DMBX1_2	TCAGGTGAAGCTCAGCAGGG
DMBX1_3	TCTTCCGGAACTTGGCCCGG
DMBX1_4	TGGCAGTGCAGTGAGCCCAG
DMBX1_5	CTGACTGCTCAGACAGACTC
DMC1_1	GAAGGAGGATCAAGTTGTGG
DMC1_2	GAGTGGATTTCAGTGGCCGT
DMC1_3	GGAGCTGGTGGCTACCCAGG
DMC1_4	TCCTCCAGCAGTTATTGCGA
DMC1_5	AGAGCTCTATGCAATGTCAA
DMRT1_1	AGAAGCAGAATCAGCAGCCA
DMRT1_2	AGCTCGGCCGCACTGGGCAG
DMRT1_3	CAGACACCTACCGCTCGCCC
DMRT1_4	TGAGCGGCGAGGCGTAGCCG
DMRT1_5	AGTAGGTGGAGTCTGAAACC
DMRT2_1	AATGGGAGAGAAATTCCGTC
DMRT2_2	ATGGGAGAGAAATTCCGTCG
DMRT2_3	GACGCGGAAGAAGAGGGCGA
DMRT2_4	TCAGGCAGGACACCACGCCG
DMRT2_5	AGAGGAGGGTGTCGCTAATG
DMRT3_1	AAAGCAGTCCATTTACACCG
DMRT3_2	GATGAGGATGCACTTCTCGC
DMRT3_3	GGCAGTCCAACGCAGCGCGG
DMRT3_4	GTGACAAAGACACTGACCAG
DMRT3_5	GAGCCCTGAGATAGTGTCCG
DMRTA1_1	AAGAACACAAGCCAGACAAC
DMRTA1_2	GGGCAGCCACCACTAGCCCA
DMRTA1_3	TACGGCGTAAATCCTAGAGT
DMRTA1_4	TGAGCGCTGACACCACACCA
DMRTA1_5	CCACACCATGGTTACGACAG
DMRTA2_1	AAGGAGCCGACCTACGGCGG

DMRTA2_2	CAGGTGCTGAACCACCACCG
DMRTA2_3	GGGCAGCCGAGAAGTACCCG
DMRTA2_4	TGAGGGCCGACACCACGCCA
DMRTA2_5	CCTGCGCCGCCATGACACGC
DMRTB1_1	AAGGCTGGGCATCGCCACGG
DMRTB1_2	AGCGCAGAGAGGTAGCCTGG
DMRTB1_3	GTGTCCCGCAGCCAGTACCA
DMRTB1_4	TGACGGGCACCAGGAAGCCA
DMRTB1_5	GCGGCCTCCGCCCCAAAAGG
DMRTC1_1	CAGTAGAAGAGGGTCAAGGG
DMRTC1_2	CCTCACGCTACTCACATGCT
DMRTC1_3	CCTGCAGGGGAGCTCCCAAG
DMRTC1_4	TGATGGTCAAATTCCTGACA
DMRTC1_5	AAGCCTGGTCAGAGACTTTG
DMRTC2_1	AAAGGGAACCACTCAGCCAC
DMRTC2_2	GACAAGGGCGAGGCTTCCGG
DMRTC2_3	GGCCAGGCACGAGGCTCCAG
DMRTC2_4	GGTGTCACCGCCCATCTCAA
DMRTC2_5	ATCATCTTACCAGGGAAGGG
DMTF1_1	AGTGCAACAACTGATATGCA
DMTF1_2	TAACTGGGATCTGTTAGCTG
DMTF1_3	TATCTGAACATGCTGCACAC
DMTF1_4	TGAAACTTCCTCGTTACCCA
DMTF1_5	ATGTAACCCATTTAGGCACG
DNA2_1	ACAGAGAGAGCTGCATCTGA
DNA2_2	GAGACTGCACATCTGACACT
DNA2_3	GATGGAGCAGCTGAACGAAC
DNA2_4	TAGAGAAGCAAGCCAGCCTC
DNA2_5	AATTGGTACGGTTCTCCATG
DNAJC1_1	AGGAAGTATGAACTCACCTG
DNAJC1_2	AGGATGATGAACGAAGGCAG
DNAJC1_3	AGTATGGTTAAGTTCCCAGG
DNAJC1_4	GGAGTTGTTTGACTTAGTGG
DNAJC1_5	CCACTAAGTCAAACAACTCC
DNAJC2_1	ACAGAGAAATCAACTGGTGG
DNAJC2_2	GCTGCTTCTGCCAAGCGCCG
DNAJC2_3	GGATAAGAAAGAGTTATCCG
DNAJC2_4	GTTGGGTGGGCACTTACCAG
DNAJC2_5	AAAACTTCGAAACTCATGCA
DNAJC28_1	AGACTGCTGAACGTGGAGGA
DNAJC28_2	AGCTATAGAACGTTTAGTGG
DNAJC28_3	GGAGACTTTGACAATCTCAG
DNAJC28_4	TTATAGACTGCTGAACGTGG
DNAJC28_5	ATTTGTTTGTTCTATCACAT
DND1_1	ACCTTCAGCGGCCTGAACCG
DND1_2	AGAGCGATCTGCCCGGGCGC
DND1_3	GCTGGGTCCCGGCTTGCAGG
DND1_4	TCAGGCGGAACTCGTAGAGG

DND1_5	CCCCAGCCTACCTTCCACCA
DNMT1_1	AAAGGGATTTGACTTTAGCC
DNMT1_2	CCTGCAGAGCTGCCAAACGG
DNMT1_3	GGAGCCGGACAGAGAAGCCA
DNMT1_4	GGGCAGCGAGATGGCCGGGA
DNMT1_5	GAGGCAAAAAGAAATCCCCA
DNMT3A_1	GAGCAGCTGAAGGCACCCGC
DNMT3A_2	GAGTGACACTGCCAAGGCCG
DNMT3A_3	GCTACCACGCCTGAGCCCGT
DNMT3A_4	GTACCGCAAAGCCATCTACG
DNMT3A_5	CCGGGAACAGCTTCCCCGCG
DNMT3B_1	AAGCTCGCGACTCTCCAAGA
DNMT3B_2	AATGGAGAGGAGGACGCCGG
DNMT3B_3	GACTTGACAGGCGATGGCGA
DNMT3B_4	GGTAGCCGGGAACTCCACGG
DNMT3B_5	CCATGTGGACGAGTCCCCCG
DNMT3L_1	ACGGTCACAGGTGTGGCCCA
DNMT3L_2	ACTGGCTGTCTCCTCCACAC
DNMT3L_3	CATGGCGGCCATCCCAGCCC
DNMT3L_4	GAAGTGGCGCAGCCAGCTCA
DNMT3L_5	ATCTGCGCCCCATGTAAGGT
DNTTIP2_1	ACAAGCAGCAGCATAGACCC
DNTTIP2_2	GAAGAACCAGGAGTATGCAG
DNTTIP2_3	GATTAAACTTTGCTTCCCAG
DNTTIP2_4	GGAAATGAGAAACAGATCGT
DNTTIP2_5	AAAGATAGTAAGATTGTACC
DOT1L_1	CAGGCCGTGCACTCACCGGA
DOT1L_2	GATATGGCGCAGGAGTCCAG
DOT1L_3	GCTGAGACTGAAGTCGCCCG
DOT1L_4	TCAGCTTCGAGAGCATGCAG
DOT1L_5	GCAGCAGGTCTACAACCACT
DPF1_1	AAGGAGGGTGGCCTCCCGGA
DPF1_2	GAGCGGGTTCTGAATGGCGG
DPF1_3	GGATGGAGAAGACCCACCGC
DPF1_4	GTACGTGTAAATCTGTCCCG
DPF1_5	CCTCGACTCGCAGACCGGCG
DPF2_1	AAGAAGATACTCCCAAGCGT
DPF2_2	GCAGTACTACAAAGATGCCA
DPF2_3	GCTGGATGCTTCCATCCTGG
DPF2_4	GTAGGAGTACAGCTGTCCGG
DPF2_5	AGAGGCGAGCATTGTAATTG
DPF3_1	ACGGAGCAAGGCTTCCAGCG
DPF3_2	AGAGCGTCCACAGTCTGCGC
DPF3_3	AGCGCTACAAGAACCGACCG
DPF3_4	TCTGTCCACAGGCCTTGCCC
DPF3_5	AAGCGAAAGAACAGGACTAG
DPPA3_1	AAGATGAGTGGCTTTACAGC
DPPA3_2	ACTCATCTTCGATTTCCCTG

DPPA3_3	ATTAGACACGCAGAAACTGC
DPPA3_4	TTAATCCAACCTACATCCCA
DPPA3_5	GAGTAAAGCTTCCGATAGAG
DPRX_1	CAACCGCCAATACCAGAGGG
DPRX_2	GGCTCAGAGGATCTTCGTAA
DPRX_3	TCCACAACCGCCAATACCAG
DPRX_4	TCGAGGCCATTTCTTTCTGA
DPRX_5	CATACACCCAACAGTACTGC
DPY30_1	GAGCCAGAGCAGATGCTGGA
DPY30_2	GATGCTGGAGGGACAAACGC
DPY30_3	GGGACTTGCTGTGCTTGCAA
DPY30_4	GTGAGACCGTACTCAGAGTG
DPY30_5	CACAACTGTCTGATCCAGGT
DQX1_1	AGACACCAGCACCACTCCAG
DQX1_2	CAGGAGGTGGCCTCGACCCG
DQX1_3	GATGAGATGGACCTGACCCT
DQX1_4	GTGCTGTGAATCCTTGTCCA
DQX1_5	AAGCAGTGCTTGAATTGTGT
DR1_1	AGCCAGTGCATCTAATCAGG
DR1_2	GCATTTGAAGCCATTCCTGT
DR1_3	TATGACATGCTCTGGTGAGA
DR1_4	TCACCAGAGCATGTCATACA
DR1_5	ACTCTTCTTCAGGAATGCCA
DRAP1_1	AGAAGAAGAAGTACAACGCG
DRAP1_2	GACGGACGAAGAGATTGGGA
DRAP1_3	GCAGCAGTTTGACTTCTTGA
DRAP1_4	TAGGAAGAGCTCGAGCGCCC
DRAP1_5	ATCTGTTCCCGACATGCAGG
DRGX_1	CTTTGGCAATCACTCTTCGG
DRGX_2	GAACACGGCCACCTACGCCC
DRGX_3	GAGTTGATGTTTCTCACTGG
DRGX_4	GGGACGCAGCAAGAGCACAG
DRGX_5	AAGAAGGTTTACCTGCACTC
DROSHA_1	ACAGATCTGATTATGACCGA
DROSHA_2	GAAGATCACCATCTCTGGAA
DROSHA_3	GGGAGAAGAAGAGAGCTCGT
DROSHA_4	GTCTTTGGAGGTTCCCACGG
DROSHA_5	TACAGATCTGATTATGACCG
DSN1_1	GCAGACGGACTGGAAACTGA
DSN1_2	GTATGAAAGAAACGAACCGG
DSN1_3	TACAGTCAAAGACTTTGCTC
DSN1_4	TTAGCAGAAAGCAAACGGCT
DSN1_5	CCTTTCCTAAGGGACACTAA
DTX3L_1	AGAGGGTCATGAAACACCGA
DTX3L_2	GGTGATATGCATCAACATGA
DTX3L_3	TTCACTGAACTCCACCCGGA
DTX3L_4	TTTGCCAAATCACCTTGCAA
DTX3L_5	CAGGTCACCTGATCGACTTG

DUXA_1	AGAGCAGGGCAAGATTCCTG
DUXA_2	CCCTAGGTAGAGAAGCCAGA
DUXA_3	GAGCAGGGCAAGATTCCTGA
DUXA_4	GATGAGAGTGTGTAACTGAG
DUXA_5	GTGTAACTGAGAGGCGCTGT
DZIP3_1	AGATGCCATGATCCACCCAA
DZIP3_2	ATACTTCAGGACTTACCCTG
DZIP3_3	GGAAGACAAGTTCTATAGCC
DZIP3_4	TAGCAGAGCTTTAACAGCCG
DZIP3_5	ACTAGGTTAAGAGTAAACAA
E2F1_1	GAGGCCGAAGTGGTAGTCGA
E2F1_2	TCAGAAGTGACCTCCTGGGA
E2F1_3	TGAGACCCAGCTCCAAGCCG
E2F1_4	TTTCGCCACAGGTGAAGCGG
E2F1_5	AAGGTCCTGACACGTCACGT
E2F2_1	ACTTCCAGTCTCGTCTGCGG
E2F2_2	AGCGGCCGAAGCCAAGGCCC
E2F2_3	CTCGGTATGACACTTCGCTG
E2F2_4	GAGCTTCAAGCACCTGACTG
E2F2_5	CACACCGCTGTACCCGCAGA
E2F3_1	GCAGTACCTGGTGACCGCCG
E2F3_2	GGACCTCAAACTGTTAACCG
E2F3_3	TATGGGAAACCTTTCTCCTC
E2F3_4	TCTTATTACAGGCAAAGCGA
E2F3_5	AATCTCCCTCAGAAAAAACG
E2F4_1	CCTGGAGGTGCCCATCCCAG
E2F4_2	GAGCATCCGGAACGTCACAG
E2F4_3	TAGCTGTACGCCAGAAGCGG
E2F4_4	TGCCGGCTTGGAGTGCCCGG
E2F4_5	ACTAGACCAGCACAAGGTGT
E2F5_1	GCAGGCACGAGAAGAGCCTG
E2F5_2	GCTGTGTGAGGTCATCAGGT
E2F5_3	GTAGGAGAAAGCCTTAAGAG
E2F5_4	GTCGAGTTCATCTAAGCCCG
E2F5_5	AACTTGATCAGCAGAAGTTG
E2F6_1	AGACTGGGTAACTTCCTCGC
E2F6_2	CTTGTCAGATCTGCTCCCGG
E2F6_3	TCTCCTCCTGGACCCGACGG
E2F6_4	TCTGAGAGCACTCATCCAGA
E2F6_5	ACCAGCGATACATCAAAACG
E2F7_1	ACAGACAGCAAGCGGAACCA
E2F7_2	ACTGACAGCAACTTCATCTA
E2F7_3	GCTGCATCTGGTCAGCCGGG
E2F7_4	GTGCATGTAACAGAAGAGCG
E2F7_5	GAACATTGGCTATGTCATAG
E2F8_1	AAAGAGGGCAAGCATGCTCG
E2F8_2	AAGGCTCTCAGGGAGAGCCG
E2F8_3	GAAATCAGTCCAAATACCAG

E2F8_4	GATGCTCTTCAAGGTGCCAA
E2F8_5	ACTTCTAGGCTTACTATCTG
E4F1_1	AGATTCAGAAGGCCTGCCAG
E4F1_2	GGGCGAGGGTGCAGTTGCGG
E4F1_3	GTGCCACAAGACCTTCAAGA
E4F1_4	TGTGGCCCACATCGTGGTGG
E4F1_5	AGGTGGTGGGCACATCAAAG
EAF2_1	ACAGTGTGGAAGGCGCAGCG
EAF2_2	ACTTGAGAACCCGCTCGCGA
EAF2_3	GAAGAAGTGTCAATAGAAGC
EAF2_4	GAAGGCAGAAGCTAGTCTAA
EAF2_5	ACCATGATACTGGAGAATGT
EBF1_1	ACAACACCAGGGATGTGCCG
EBF1_2	GGTGCTGGACGCCAACACGG
EBF1_3	TAAGAGGGCGTACCTTCCGA
EBF1_4	TGACATGCGGAGATTCCAGG
EBF1_5	AGTGAAGGATGGACGACGGG
EBF2_1	AAACCGAAATGAGACTCCAT
EBF2_2	AACTCTGAAAGAGAAATCGC
EBF2_3	ATTCTCCACAAAGTCCACGA
EBF2_4	TCACAGCCCATCGCTTACGA
EBF2_5	GACTATGCTTGTATGGAGCG
EBF3_1	CAGGAGAATATTCCGCGCGG
EBF3_2	CTTCTGACGGGTCTAGGCGG
EBF3_3	GCGCAGGCCATCGTCTACGA
EBF3_4	GGAATCTCCGCATATCTCGA
EBF3_5	GGCCGGTACGTACCTCTGGG
EBF4_1	ACAACTCCAAGCATGGCCGC
EBF4_2	CATTGTTATACACCAGCCGG
EBF4_3	GAGCGCTTACCTCTGCGCCG
EBF4_4	GTCGCCGATGACAATGACGG
EBF4_5	AGAACTGCCTGAAGAATGCG
ECD_1	AAAGGTTTGTGCCAGACCGG
ECD_2	AGAGTGGTGAGGCAGTCACA
ECD_3	CTGGTGAGAGATCTAACCAC
ECD_4	GCTGTGAATAGGCGCATCAG
ECD_5	AATTCATGGGCTCGGTACTG
ECE2_1	AGCGAGAACGCCCATCGGGC
ECE2_2	TGAGAGCATGGCCTCTCCAG
ECE2_3	TGTGGACTACTCATCAGTCG
ECE2_4	TGTGGGCCCACCTACCTCTG
ECE2_5	CTTGTGCAGACTCAAAGCGT
ECSIT_1	CAGGCCACCCTACTGGCCCG
ECSIT_2	GCAGGAGACCAACCCAACCC
ECSIT_3	GCCTTGGTGGGCCTCTGCCG
ECSIT_4	GGAGCCTGACCTTAGTGCCA
ECSIT_5	ATGCGCTGGATGATGTTGCG
EDF1_1	CATTGGAAAGCCCATCGAGA

EDF1_2	GCTGCGCAAGAAGGGCCCTA
EDF1_3	GTCCCAGTCGCTCTCGGCCA
EDF1_4	TACGCAGAAGGACCTGGCCA
EDF1_5	ACATTCTATTACCAAGAACA
EED_1	GATCCAGCTACAGCCAGCAG
EED_2	GGCGGGAACAGACATGCCTG
EED_3	TATCCAGACGGACACTCTGG
EED_4	TTGTAGCACTATGTTGGCCA
EED_5	AAGAGAATGATCCATACCAC
EGR1_1	AACGGGTCAGAGATCTGCAG
EGR1_2	AAGGCCTTAATAGTAGACAG
EGR1_3	GAGTGAGGAAAGGATCCGAA
EGR1_4	GGGAACAGAGGAGTACGTGG
EGR1_5	ACGCCCTTACGCTTGCCCAG
EGR2_1	AACAGCAGCAGTCTTGGCGG
EGR2_2	GAGCTGCTACCAGGCAGCCG
EGR2_3	GGAGCAAATGATGACCGCCA
EGR2_4	GTCTGACAACATCTACCCGG
EGR2_5	CTCCGTTCATCTGGTCAAAG
EGR3_1	AAAGACAAGCAGATCCACCC
EGR3_2	ATGGGCTTGAGGGTGAGCGG
EGR3_3	GGAGAAGAGGTTGAGCGCGC
EGR3_4	GGTCACGGTCTTGTTGCCGG
EGR3_5	GAAGGCGAACTTTCCCAAGT
EGR4_1	CAAGCAGAAGGCGCGCGCCG
EGR4_2	CTTACCTCCAGGGTAGCCGG
EGR4_3	TCCAGGGTAGCCGGTGGCCG
EGR4_4	TTTAGGGAAGTCCGCCGCGG
EGR4_5	CGGGTTCGGAAAACTCGCTA
EHF_1	ATGACAGCTTACCAACAGGA
EHF_2	GAGGATGAATTTCATGCCAC
EHF_3	GGTGTAATGAATCTCAACCC
EHF_4	TTGGTGTGGCACTTGGCAGG
EHF_5	GGATACAATTGGCATCCAGC
EHMT1_1	AAGAATGGGAACCTATAGCC
EHMT1_2	AGAGTGCTGGTAGTCCTCAA
EHMT1_3	GCTCACCTCGGCATCGGCGG
EHMT1_4	TAACAGGCAGTTCCGGCGAG
EHMT1_5	ACTTATACGACTCAGAACCT
EHMT2_1	GCTGCACTCACCTCTCTCGG
EHMT2_2	TAGTGAAGAAACCCTGCCCA
EHMT2_3	TCACTGGGAAAGGTGACCTC
EHMT2_4	TCCAGCCACCACGAGCCCAG
EHMT2_5	CAAGAGGTGACCATCCCCCG
EID1_1	ATGGATGTGATGCCTGGCGA
EID1_2	GACTTCGAGAGCGAGGACGA
EID1_3	GCTCGAGGAGGAAGGCCCAA
EID1_4	TGCTAACGGGCCCAACGCTG

EID1_5	ACTGCTCTCTTCATACAGCT
EID2_1	AGGTCCAGACACGGGAACGG
EID2_2	AGTGGACGAAGAGGGTCCCG
EID2_3	CAGGGAAGCCCGGATGGCGG
EID2_4	GTGGACGAAGAGGGTCCCGA
EID2_5	CAGTGTCCCGCAGACAGGCG
EID2B_1	GAGGGAAGCAGCCTTTGACG
EID2B_2	GCAGCGAAGTCCATCTGCGG
EID2B_3	GCGTCAGCGACGTACTGCGG
EID2B_4	TGTGGAAGGCTGCAAGGCGA
EID2B_5	AGCTACCATCCAGATACTCG
EIF3C_1	AGGATGCGGATATAGAACCG
EIF3C_2	ATTGCAGCGGAAAACAACCT
EIF3C_3	GGAGGAGAAGGGCACTACCG
EIF3C_4	TGATATGGGACTCGAAATCA
EIF3CL_1	AACTTGCGACTCTCCCCAGA
EIF3CL_2	GCAGAGCATGGTGGTAGATG
EIF3CL_3	GGATGCGGATATAGAACCGG
EIF3CL_4	TCTTGGCAGCCAGAGATGTG
EIF3CL_5	ATGTGCGGATCCGGTCTGTG
EIF3E_1	CAACATGGTAGACTTTGCTA
EIF3E_2	GATGCTCTTTGACTACCTGG
EIF3E_3	TAACCGTGTAAGGTCTTCCA
EIF3E_4	TGTGAATGACTTCTTCTTGG
EIF3E_5	AATGCTTTAAGTTCACTCTG
EIF4A1_1	AGATTTCACTGTATCCGCCA
EIF4A1_2	AGTTCCCTCTACTCACCAGC
EIF4A1_3	CCGCCAGTTCTACATCAACG
EIF4A1_4	GGAGCCCGAAGGCGTCATCG
EIF4A1_5	AAACATCTTGATGTATTTGG
EIF4A2_1	AGAGAACATGGCGGCCCAGA
EIF4A2_2	CAGAGAACATGGCGGCCCAG
EIF4A2_3	CGAGACACTGACCATTACAC
EIF4A2_4	CGTAAGCATAGATGCCACGA
EIF4A2_5	AGATTTGATCCTTAAAACCA
EIF4A3_1	AGAGCGGGAGTCCATCATGA
EIF4A3_2	CATGGCGACCACGGCCACGA
EIF4A3_3	GAGGACTGAGATACTGAAGG
EIF4A3_4	GTTGGCTGTGCAGATCCAGA
EIF4A3_5	ACTGATGAGAACCACCTGTG
EIF4G2_1	AATGCAAATGAGGCTGTCAA
EIF4G2_2	ATTAGACCATGAACGAGCCA
EIF4G2_3	CAACATCTTGATCTTAGCAA
EIF4G2_4	GAACGATGACGTCCCATGGT
EIF4G2_5	TGTCCTTTCTTAGAAAACCG
EIF6_1	AATGAGGGAATCCCGCATGC
EIF6_2	GGAGGAGCGGCTCTCAGCCT
EIF6_3	GGCGATAGACGCGTGCACCA

EIF6_4	TCTGGCAGATGTGCTCAAGG
EIF6_5	AGACACAGTGCAGATTAGGC
ELAVL3_1	AAGGAGTGGCCCGTTGGGCA
ELAVL3_2	AGTTCACAAACCCGTAGCCA
ELAVL3_3	ATGGGCTCAGCTGCGCCCAG
ELAVL3_4	CGTGTACAACCTGTCACCGG
ELAVL3_5	ACCTCTACCAGTCATCCGCC
ELAVL4_1	GAAGTGGACCTGGGTAGCGC
ELAVL4_2	GGAGTGGAATGGCTTGAAGA
ELAVL4_3	GTACCCGTTGAGGCTGGCGA
ELAVL4_4	GTCGGACCATTTGACACCTG
ELAVL4_5	CCTGGTGGTGAAGTGGACCT
ELF1_1	ATACCTGAGTGCTCTTCCCA
ELF1_2	GAAGTGATTACAGTGCCAGG
ELF1_3	GCCAGTACAGGCTGTCCCAG
ELF1_4	TTTCATCCAGCATAGGGCCA
ELF1_5	ACATGTTCCACAATTACGGC
ELF2_1	AACTGAAGAGTCTGAACCCA
ELF2_2	CAGCAATCACCAATTTCCAA
ELF2_3	GGATGAAGCATCGTGCCCAG
ELF2_4	TTACCTCAAAGCTCGTCCCA
ELF2_5	ATGTACTAGGACAGTTCGTG
ELF3_1	AAGGGCACAATTGCAGAGGG
ELF3_2	CAGTGCGATGTACAGCTCGG
ELF3_3	GTGGACCTGGATCCCACTGA
ELF3_4	TCCAGGAGGCCCTAGACCCA
ELF3_5	AACCCCCAGATGTCATTGGA
ELF4_1	CTCGCACACCATGTCAACCG
ELF4_2	GAGTTGGACGACGTTCACAA
ELF4_3	GGAGGAGAAGGCCTCCAGAG
ELF4_4	TGAGTTCGCAAGCAACGGGA
ELF4_5	ACATGAACTATGAGACAATG
ELF5_1	AGATCAGTCCACGACATCAG
ELF5_2	AGTACAGGTACTCGCCGCAG
ELF5_3	GCTTAGTCCAGTATTCAGGG
ELF5_4	GGTGGTTAAATCGGAAGCCC
ELF5_5	CAGATCAGTCCACGACATCA
ELK1_1	CTTGCGGTACTACTATGACA
ELK1_2	GAAAGAGGATCCTACCTGGA
ELK1_3	GCACTCACCAATGTATGCGT
ELK1_4	GGAAAGCCAGGCACACCCAA
ELK1_5	GAAATCGGAAGAGCTTAATG
ELK3_1	CAAGCACGTCACCAGGCCGG
ELK3_2	GAAGATGGATCCTCACGCGG
ELK3_3	TCAGCGGAGCAACTGGACTA
ELK3_4	TGGAGAGTGCAATCACGCTG
ELK3_5	AAACCGACAAGCACGTCACC
ELK4_1	AAGTGGATACTGGAGAGCAA

ELK4_2	GCTGAAGAAGGAACTGCCAC
ELK4_3	TCTGGGTGAGAACTCAGTGG
ELK4_4	TGGACAGTGCTATCACCCTG
ELK4_5	CTTTACATAATAGTATCTGA
ELL_1	GCAGAGCATGGCCCAGGCGG
ELL_2	GCAGGCGGACAAGGACGCGC
ELL_3	GGACTGGCCTGGCTACTCGG
ELL_4	TCGTGCGGGCGGGTTAGCGA
ELL_5	AGAACGTCCGCGCCTCTGCG
ELL2_1	CATAGCGCTGCTCCTCCCGC
ELL2_2	GACACGAGAAAGAATGACCC
ELL2_3	TCAGAGGCCATACAGGGACA
ELL2_4	TGTGGGCAAAGACAACCCTC
ELL2_5	AATCCTTTAAGGTATATGAG
ELL3_1	AACACAGGAGGCTATTCTGA
ELL3_2	ATTGCTTTCCAAGGCCACCG
ELL3_3	GGCTCAACGACGCTGCCCTG
ELL3_4	TATCTGAGACTCCCAGGCCC
ELL3_5	AGCTCGCACTTACCCCTCGG
ELMSAN1_1	ACTGAAGCGGGAGAAAGCGG
ELMSAN1_2	CCGTGCGGAAACCAAAGCAG
ELMSAN1_3	GAGCTTGAGCATCTGTCCCG
ELMSAN1_4	TTACGGCTCGATGCTCACTG
ELMSAN1_5	CAGTCATCGTCACCCGCAGG
ELP2_1	ACAGAGACTGACCGTTGAGA
ELP2_2	CAAGATAGTGATCTTCCAGA
ELP2_3	CACGTGAGAAGTCTCCAGCA
ELP2_4	TATTCTAGAGCCTCCCACTG
ELP2_5	AATTTCATGCCAAGTCACCT
ELP3_1	AATCAGAATCAGGTCCACCA
ELP3_2	ACTGATTGAAGCCCACGAGC
ELP3_3	CAGCAATGATATCCACCAGG
ELP3_4	GGAAAGGGTCATATCTGGCA
ELP3_5	AAAACAACTTGGTCATAGTG
ELP4_1	AAGCAGCTTTGTGTAGCCAG
ELP4_2	AAGTTCCTCTATGTTCTCCG
ELP4_3	CCAGGTGGAGGTTTAGCCGT
ELP4_4	GCAACGTCACCAGTTTCCAG
ELP4_5	AATGCCACAAGAACTAATTG
ELP5_1	AAGCAGCACCAGGCCGCCCA
ELP5_2	AGCGAGGAAGAGTTTCGTGA
ELP5_3	GCTGAGTGAATCGAGAGCAA
ELP5_4	TTGTCAAGAAATCTGCACTG
ELP5_5	AGGATCTGTCCTCTTGCACA
ELP6_1	AAGTGAAGCTCTGATCCCGG
ELP6_2	GATGACTGAGGCCATTCAGC
ELP6_3	TAGACAGTGGAGAGGCTCGG
ELP6_4	TGTCAGCCTGACCATGGCGC

ELP6_5	ATTGTTTGAGTTTGTACGGG
EMX1_1	GCGCCGAAGAAGGAGTGCGG
EMX1_2	GCTCCCATCACATCAACCGG
EMX1_3	GTGAGGGTAGTTGAGCGCCG
EMX1_4	TGCAGAAGCAGCCCGTCCTG
EMX1_5	CAACTACCCTCACCCCAGCG
EMX2_1	CACCAAGCAGGCGAGTCCGG
EMX2_2	GACCCAGATATCGGTAGCGG
EMX2_3	GGACGCACCATATTAACCGG
EMX2_4	GTTAGCGTAGCTGAGTGCCG
EMX2_5	CGCTGGCCCGAAAGCCCAAG
EN1_1	GCGAACTGTGGCCCACCCGA
EN1_2	GGAGTGGTTGTACAGTCCCT
EN1_3	GTACAACCACTCCACCACCA
EN1_4	TTTAGACGCGCCCGCGCCGG
EN1_5	CGTTGGCTGAGCCCATAAGT
EN2_1	AAGACGCTCTCGCTGCACGG
EN2_2	ACCCGGAGAGTCGCTGCCGG
EN2_3	CAACCACTCCACCACAGCCA
EN2_4	CACGGGCAACAAGAACACGC
EN2_5	CCGAGTCCGAGCTCACCGAC
ENY2_1	AAAGGCTGGCATGCTGAGCA
ENY2_2	CAGTTGAAGGCACACTGTAA
ENY2_3	GTGGCTGAAATCACTCCAAA
ENY2_4	TGGCTGGAAGGATCAGTTGA
ENY2_5	AACCAAAAGTTGATAGAAAC
EOMES_1	AAATCTCCTGTCTCATCCAG
EOMES_2	GAACACATTGTAGTGGGCAG
EOMES_3	GAGGTCGAGGTTCTTACCAG
EOMES_4	GGTGTCACTAAGCATGGCCG
EOMES_5	ACTTCAACAATATGCAGTCG
EP300_1	AACCAGCAGAATCAGCAGCC
EP300_2	ACAGAATTGGGACTAACCAA
EP300_3	ATGCTCACAAGTGCCAGCGC
EP300_4	GGACAGCAGATTGGAGCCAG
EP300_5	ATGGTGAACCATAAGGATTG
EP400_1	AGATTTATTTAGTCAACGAG
EP400_2	AGTGGTCATAAGGTTACACA
EP400_3	ATCAGTTCAAAGCTGCCGAG
EP400_4	GTCATTGTCATAAAACACGA
EP400_5	AAATAAAATCACTCGTCACG
EPAS1_1	AAGTGCACGGTCACCAACAG
EPAS1_2	ATGATGAGGCAGGACAGCAG
EPAS1_3	CTTGGAGGGTTTCATTGCCG
EPAS1_4	TTAGGAGTAGCTCGGAGAGG
EPAS1_5	ACTGGCACCCTATATCCCCA
EPC1_1	AGAGCTATTGCACTTAACAC
EPC1_2	GAACAGGAACAGCACAACCT

EPC1_3	GCACGAAGACGGGTTGGGCG
EPC1_4	GGAGTAGCAGCGGCAGACGA
EPC1_5	GCAAATCCAATACACCTTTG
EPC2_1	ACTCCAGCAACTCTTCATAA
EPC2_2	GAAAGAAGAGGCTTCTGATG
EPC2_3	GATCAGGCATGTCCTTGCCG
EPC2_4	GCAAGGAGGCGAATTGGCAG
EPC2_5	GAAAACGTAAAAACTGCAGG
ERBB4_1	AAAGCCACAGCTTTACCCGC
ERBB4_2	GAATGGTGTCTGCATAACAA
ERBB4_3	GGTGAGCCTTCTCGTGGCGG
ERBB4_4	GTGTGTGCAGAACAATGTGA
ERBB4_5	AGCGGCGACACGACAGACAT
ERCC1_1	ATTACGTCGCCAAATTCCCA
ERCC1_2	CTGGGCCGAGGTGTCCACAG
ERCC1_3	GAAACCAGCGGACCTCCTGA
ERCC1_4	GAGGGACCTCATCCTCGTCG
ERCC1_5	ACCCAGACTACATCCATGGG
ERCC2_1	AAGGAACAGGTGCTCACCTC
ERCC2_2	ATCCAGGTTGTAGATGCCAG
ERCC2_3	GCGGGAGCTCAAACGCACGC
ERCC2_4	TTATCGGCAGGCATATCCGC
ERCC2_5	ACCTCATAGAATCGGCAGTG
ERCC3_1	ATATGCCCAAGACTTCTTGG
ERCC3_2	GATGCTGGATTACATCAGGG
ERCC3_3	GGAAGCGGTTCCCTCGGCGG
ERCC3_4	GGGATGTCAGATTTACCCTG
ERCC3_5	AGCGGCATTCTCGGATCACG
ERCC5_1	AACATTCAGGACACATCCGA
ERCC5_2	GAACTGGTAGGAACTAGGGA
ERCC5_3	GAAGGACTTAAAGTGAGAGA
ERCC5_4	GAAGGGAAGATCCTGGCTGT
ERCC5_5	AAATGTCACTTGTTCACGTG
ERCC6_1	ACTAGATCACGCCAGTCTGG
ERCC6_2	GGAGTCAGACATGAGGCCAG
ERCC6_3	GGTCATGTACGACATCCCTA
ERCC6_4	GGTGCGCATCAGCAGCTCCG
ERCC6_5	ACTGATTACGAGATACAATG
ERCC6L_1	AGCAGGCTGCTCATTACCTA
ERCC6L_2	GGGTCATGGAGGCATCCCGA
ERCC6L_3	GTTATGGAAATAATGGGCCA
ERCC6L_4	TATGAGACTCTTGTAAAGCG
ERCC6L_5	AACCTTTCATGGTCCTAGCA
ERCC6L2_1	ACAAAGAGAGAACTAGCCAC
ERCC6L2_2	GAAGGATGAATTGGACACCT
ERCC6L2_3	TATGGACACTACATCCATGG
ERCC6L2_4	TTTGCCTGAGGTTTCCGCGC
ERCC6L2_5	AAGCATAAAATCTATCACAG

ERF_1	AGGGATCCAGGACCGGCCAG
ERF_2	AGGGCGTCATTGCCTGGCAG
ERF_3	ATCGGGCTCAGCGTGGGCGA
ERF_4	GCCGGTGCTGACAAGAGCGG
ERF_5	CGGAAGTGGCTACCACCCGA
ERG_1	GAGACAGCCAATCCTGCTGA
ERG_2	GCTGCCGTAGTTCATCCCAA
ERG_3	GGAACTGTGCAAGATGACCA
ERG_4	TTTCTAGCATGCATTAACCG
ERG_5	AACACTGTACCTTTCGACTG
ERI1_1	ACCTCGGGACTGGGACGCGG
ERI1_2	ACTGAAGTCACTCGCACTGG
ERI1_3	GAGTAAAGAGCCTGCCGGCG
ERI1_4	GAGTCATCAAGACCACAGTG
ERI1_5	CAGAGCTGATACCTTCCCTC
ESCO1_1	AGCTTAACCGGAGATCACAA
ESCO1_2	ATTCTGGCTGAATACCCTGA
ESCO1_3	GGTTGACGAGATTAGAGAGA
ESCO1_4	TGTTCAGGCACTGATGGCTG
ESCO1_5	TCGTAATAAGCCTAATTTAG
ESCO2_1	AAACCACAAGTTACACTCCA
ESCO2_2	AAATGGCAGCTCTTACTCCA
ESCO2_3	AATTGCAGAACCCATCAAAC
ESCO2_4	CCACAAGTTACACTCCAGGG
ESCO2_5	ACCCCCCAATTATAGGACGC
ESR1_1	AATTCAGATAATCGACGCCA
ESR1_2	GAAGTGGGAATGATGAAAGG
ESR1_3	TACCTGGAGAACGAGCCCAG
ESR1_4	TCATGAGCGGGCTTGGCCAA
ESR1_5	CTGACCGTAGACCTGCGCGT
ESR2_1	ACAGATGTGATAACTGGCGA
ESR2_2	AGGCCTGGCAGCTCTTGCGC
ESR2_3	GGTCAGGGACATCATCATGG
ESR2_4	TACGCATCGGGATATCACTA
ESR2_5	AGCAGGGCTATAGAATGTCA
ESRRA_1	CACACCATAGTGGTAGCCGG
ESRRA_2	CCTTGAGCATGCCCACCCGC
ESRRA_3	GAAGTACAAGCGGCGGCCGG
ESRRA_4	TGATGGGCACCTCCCAGCCG
ESRRA_5	AGACACCAGTGCATTCACTG
ESRRB_1	AGTGCGAGATCACCAAACGG
ESRRB_2	GATTGTCTCATACCTACTGG
ESRRB_3	GCCGTCAGAAATACAAGCGA
ESRRB_4	GGGTGGCGAGTCCAGACCGT
ESRRB_5	CCCATGCCGCAAGAGCTACG
ESRRG_1	AGCGTCAACCACCACAGCCC
ESRRG_2	CCTGAGGCAGACCTCTACCA
ESRRG_3	GAAATACAAGAGCATGAAGC

ESRRG_4	GATAACCACCAACTCTCGGT
ESRRG_5	AAGAGACTGTGTTTAGTGTG
ESX1_1	ACAGGCGCCATGCGTGGCCC
ESX1_2	ATGGGCGGCCCGGTTGGCAC
ESX1_3	GTTGGCACAGGCGCCATGGG
ESX1_4	TGTGCCAACCGGGCCGCCCA
ESX1_5	AATATCCCGACGTTGTGGCG
ETS1_1	GAGAAAGCAGTCTTTACCCA
ETS1_2	GAGAGTCGGCTTGAGATCGA
ETS1_3	GGGTCTCGGAGAATGACCGA
ETS1_4	TTACCTCCAGGTAAACTCGG
ETS1_5	CTTACTAATGAAGTAATCCG
ETS2_1	AGAGCAGTAGGTGACGCTGA
ETS2_2	GCTGTCCGCACCGTTCTCAG
ETS2_3	GTTAATCCAATGAGGAACGG
ETS2_4	TGTTGCTCACTCCACAGCCA
ETS2_5	CAGACACAGAATTACCCCAA
ETV1_1	AGTGTATGAACACAACACCA
ETV1_2	GGAACGTCACATCAACGAGG
ETV1_3	GGGAAGGACCCACATACCAA
ETV1_4	TTTGGAGATGCATGATGCAG
ETV1_5	TAAGTCAATTACAGGAAACA
ETV2_1	CAGCTGTGGGAAGCTTGCCA
ETV2_2	CCAGCTGGTGTTGCTCCCGG
ETV2_3	GACATCGTGCGCAAGAGCGG
ETV2_4	GGTGGTACAGTCCGGGCCCG
ETV2_5	CCAGCTGGGACTGTTCTGTG
ETV3_1	AGGACACGGGATCCACACCC
ETV3_2	AGGGATTGGAGAGACAGCGA
ETV3_3	GCAGTGCCAAATGCATCCTG
ETV3_4	TCAGCTGCTGACTGGCGCCG
ETV3_5	ATCCCTGGCCTAGCAAACAG
ETV4_1	AAATTCCGTTGCTCTGCCCG
ETV4_2	AGTAAAGGCACTGCTCGCCA
ETV4_3	CGAAGCGCTGATCGGCCCGC
ETV4_4	GAGGGAGCGGCCGGATGGAG
ETV4_5	ATCAATGTACCTCCACACAG
ETV5_1	ACAAGACGACAGCTCAGAGG
ETV5_2	GAATCGACGCAGTAATCCCG
ETV5_3	GGCGAGGTTCCCTTCAGCTG
ETV5_4	TATGATAGGAAGCCTCCCTC
ETV5_5	ACCAGTATCCATCAGAACAG
ETV6_1	GAGGAAGCGTAACTCGGCAC
ETV6_2	GCAGGGATGACGTAGCCCAG
ETV6_3	TGGGCAGTGATTATTCTCCA
ETV6_4	TGTGTATAGAGTTTCCAGGG
ETV6_5	AATGGTGAAAAAAGAATCCG
ETV7_1	GAGCTTCCTCACCTTCCGGG

ETV7_2	GATACACGTAATCCCACAGC
ETV7_3	GTACTCTCTGCCATGCACCG
ETV7_4	TGAGGGACTCCTCCTTGCCA
ETV7_5	CTCTCAGATGGACACCCGAA
EVX1_1	ACAGGTGAAGTCCGAGGCGG
EVX1_2	AGAACTACGTATCCAGGCCG
EVX1_3	CTGGTACTCGGCGTTCCCGG
EVX1_4	TGGACTCGCAGGCAGCGCGG
EVX1_5	TCAAATTTGTCCGAAGCCGT
EVX2_1	AGGGCGCGGAAGGTGTCCAG
EVX2_2	CAGGCGCGCGATCTGCTCGC
EVX2_3	GAAGCCGGGCCATTATTCAG
EVX2_4	GGCGGCGGAGGAGATTTCGG
EVX2_5	ACCGGGAGAACTATGTGTCG
EWSR1_1	ATGAGTGGCCCTGATAACCG
EWSR1_2	GACAGGCTGGCTGTATGCCT
EWSR1_3	GCATATGGGCAACAAAGCTA
EWSR1_4	TCAACCTCAATCTAGCACAG
EWSR1_5	AAAAAGTAGACTGACCTGGT
EXOSC1_1	ACGTCTGTGTAACTTGGAGG
EXOSC1_2	AGCGGCACCTACACCCGCCA
EXOSC1_3	ATTTATAAGAGTTTCCGCCC
EXOSC1_4	GTTGGACTGTGCATCACCTA
EXOSC1_5	AAGTACACATCCTGTATGTG
EXOSC2_1	AACACCTGAGCACAAAGAAG
EXOSC2_2	GAAACATCTAGTGGTGCCGG
EXOSC2_3	TCGTCCATGAACCTTCCTGG
EXOSC2_4	TGAAGTAGGAGACATCGTAG
EXOSC2_5	AAGCGCAGGATACACTGATA
EXOSC3_1	GTCGCGGCTGAATCTCTCGC
EXOSC3_2	TAAAGACATGGAACCAGAGA
EXOSC3_3	TAGGTTGGAGATCTCATCTA
EXOSC3_4	TGCCGGGCTCCTTGTGACGG
EXOSC3_5	ACGCACAGTACTAGGTCAGG
EXOSC4_1	AAGCAGCGCAATCTGTCCTG
EXOSC4_2	GCACAGGTGAGCGCAAGCGA
EXOSC4_3	GCAGGGCAACACCAAGGCAC
EXOSC4_4	GTCGGACCAGGGCTACCGGG
EXOSC4_5	ACTATATTGACAGTTCACTA
EXOSC5_1	GAAGTTCTTACCTTGCAGGA
EXOSC5_2	GATCAGGAACACGTGCGAGG
EXOSC5_3	GGATGTAGGATCCAGCACGA
EXOSC5_4	GTACACACCCGCCAGGACAG
EXOSC5_5	CCGGCCCGTACACACCCGCC
EXOSC6_1	CAAGGGCTCGGCCTACCTGG
EXOSC6_2	GCTGCGATTCTTCAGGGCCG
EXOSC6_3	TCGGCCGCGTACAGCTGCGG
EXOSC6_4	TGGGCGGAGGCCGTACGCCT

EXOSC6_5	GAGCAGGCGACCGCGCAGCG
EXOSC7_1	AAGCTGGAGAAACCAAATGA
EXOSC7_2	AGCCACGGCCATCCACACGG
EXOSC7_3	GAGAGCAGCCTTTACAGCAA
EXOSC7_4	GCTCCCGAGGACTAATGCAG
EXOSC7_5	AGATCGCTAACACCCTCTAT
EXOSC8_1	AAAGAGAACTGCCGTCCTGA
EXOSC8_2	AATCTCCTGTAATACTCCAG
EXOSC8_3	GAGGAGGAACATCTGGCAAC
EXOSC8_4	TAACACGTGTTTCAGAACCG
EXOSC8_5	ACCAACGTATCCTTTATCAG
EXOSC9_1	ATTTGTACTATCCAGTCCAG
EXOSC9_2	GTTCCAGGAGTCCATAGCAC
EXOSC9_3	TAACCTTGAACTCTCTCAGA
EXOSC9_4	TGAACGAGAAGAACGTGTGA
EXOSC9_5	AAACTCAATCGGGCAACAGA
EYA1_1	ACAGCTTCAACGACAGCCGA
EYA1_2	AGAAGGCAGACACTCACCTT
EYA1_3	GGGCAAACACAGTTTACCAC
EYA1_4	TTGGAAGGGTAAATCTGTGG
EYA1_5	CCTACGCCAACAGATATGGG
EYA2_1	ACAACAGGGTTCTATCAAGG
EYA2_2	ACTGTACTGCGTCTGGCCGT
EYA2_3	AGAGCTGGGACACTCTCAGG
EYA2_4	GAGGAATCCACTCTGGCCAG
EYA2_5	ACACTGACCGTGCATCTGGT
EYA3_1	ACATGGGAAGGTTTGAAGCA
EYA3_2	AGAAGAAGTAGATATCAGGC
EYA3_3	AGAGCTGCGGTGCTTACCAG
EYA3_4	GTTTCGGAAACTGCTTACCC
EYA3_5	AACATGACTAGCAAGAACCG
EYA4_1	AATCCTGGGAGTCTTCCATG
EYA4_2	ACATCGAATAACACAGCCGA
EYA4_3	GCTGCCTCAGTTACAGCCCA
EYA4_4	GTTACTACAAATGGGACAGG
EYA4_5	ACTCAGTCCCCATTACAGAG
EZH1_1	AGAGCACTGGGACTGCAAGG
EZH1_2	CCAATGCCAAGTCTGTGCAG
EZH1_3	TACCACTACCTTCTTCACCA
EZH1_4	TTTAGTCTCAGCCACAGCAG
EZH1_5	ACCATTCTCAGGGAACATTG
EZH2_1	ACCAAGAATGGAAACAGCGA
EZH2_2	ACTGGGAAGAAATCTGAGAA
EZH2_3	GATCTGGAGGATCACCGAGA
EZH2_4	TCAGAAGGAAATTTCCGAGG
EZH2_5	ATGTTGGGGGTACATTCAGG
FABP1_1	AAGTCTGTGACCGAACTCAA
FABP1_2	GCCGGAAGAGCTCATCCAGA

FABP1_3	GTGAGCTGGAGACAATGACA
FABP1_4	TTCAAGTTCACCATCACCGC
FABP1_5	CAGAAGGGGAAGGATATCAA
FAM170A_1	AACGCAGGAGCAGTATTCGG
FAM170A_2	CAGGAAACCGCTGAGAAGGG
FAM170A_3	GAAACCGCTGAGAAGGGAGG
FAM170A_4	TCTGGGTGTGTCCTCAACGG
FAM170A_5	GAGACATGTTGTGAGCGGGG
FAM175A_1	AGGCGGCGGTAGCATGGAGG
FAM175A_2	GAGGCGGCGGTAGCATGGAG
FAM175A_3	GATTGTCTACTACATCGAGA
FAM175A_4	TGATCTGATCTGAATGACGA
FAM175A_5	AATGTATGCTTCATTACAAG
FAM175B_1	ACTGGGAGAGGTAAGACAAG
FAM175B_2	GCTGGAGATTTCCATCATGG
FAM175B_3	GTGAGTGGAATTGTTGGCAG
FAM175B_4	TCAGCTGCAAACCCAGAGGA
FAM175B_5	CACATATTCTAAAGCGTGAG
FAM189A2_1	ACCACAGCTTCATATGGCGG
FAM189A2_2	AGCGGCCACCAAGCAGACGG
FAM189A2_3	GTTAAGGTGGACTTAGGTGA
FAM189A2_4	TTCGACCTGGTCCTCAAGGG
FAM189A2_5	ACCTCCAGATATTCGCAACC
FAM208A_1	AACATCGCTGAGCCCAGCGG
FAM208A_2	AAGAGAGACCACCTACCATA
FAM208A_3	ATGCTCTCATGCCTCTCCTG
FAM208A_4	ATGGTCAGTGCCCACTTCGC
FAM208A_5	ACAAGCGAATCCTTTGGACA
FAM208B_1	AGAACTGTCAAACAGACAAG
FAM208B_2	GTATCCTCACCTGTTGACAG
FAM208B_3	TGAGGAGCAACACTCAATGA
FAM208B_4	TGTGGAGATTTCTTCACAGG
FAM208B_5	CATTCACCAGGACTTACGAT
FANCM_1	AGGTCATTTACCATGACCTG
FANCM_2	GAAATTGTACATGACCACGG
FANCM_3	TACCCAGAAGGAGCAACTGG
FANCM_4	TGACGGTGGTTACAACACGC
FANCM_5	AAAGAAAACTGAGAATAACC
FANK1_1	GCAGAGCTGTACAGCCTCCC
FANK1_2	CATATGTTGCAGTCTAATGC
FANK1_3	CTGGGCAGCAACCATCAGAG
FANK1_4	GCTGTACAGATTTCGCCTGA
FANK1_5	TCACAGCATTGAATTATACT
FBL_1	ATATGAAAGAGACCATGCCG
FBL_2	CAAAGTGTCCTCCATTACGC
FBL_3	CCAGATCCACATCAAACCGG
FBL_4	TGAGGTCACGGCCAGAGCGG
FBL_5	ACCAACGATGTCAGAGACAT

FBRS_1	GAGACCCGAAGCTGCAGCTG
FBRS_2	GAGGGTGGCGAAGCTGGCGA
FBRS_3	GGCGGCAGAAACCAGGGAAG
FBRS_4	GGTGGGAGAGCTCCTGCCCA
FBRS_5	CATTCCGAAAGTCAAGCTTG
FBRSL1_1	ATGAAGGTCACCGTGTCCAA
FBRSL1_2	CAGGAGGAGGAGGTCATCGA
FBRSL1_3	CGTGTGTCGGGAGCAGGCCG
FBRSL1_4	GTTCTCACTGGGCTCCGCAG
FBRSL1_5	CAGGATGGAAGCGCTCAGTG
FBXL19_1	AGGAGCCGCAGGGCTGGCAG
FBXL19_2	CCAGCTGAGGTCCAGGGCAC
FBXL19_3	GAGCCACTGCGCCCACGTCG
FBXL19_4	TGAGAAGCAGCTTCTCCTCA
FBXL19_5	AGAAGCTAGAGCGTTTCAAG
FBXO10_1	GAAGAAAGATGCCTGACGCT
FBXO10_2	GATGGAAGACTCCAAGTCCA
FBXO10_3	GCAGAAGGATAAGGAGGCCA
FBXO10_4	GGACGACAATGCCAGAGCGA
FBXO10_5	ATACCCACCTTATACATGAT
FBXO11_1	AGTATGAACATCCAAATCCC
FBXO11_2	GATACTATTGAAGATGCCCT
FBXO11_3	GATGGACGAGGCCTTATTGA
FBXO11_4	GTTCGATGTGAAATTCACCA
FBXO11_5	AACCACTGTAGGGTTAGCAT
FBXO17_1	ACGGATCCTGACCCTCACAC
FBXO17_2	GAAGATGAGATTGCGGCCGA
FBXO17_3	GGGCCGCGCACTCTACGCAG
FBXO17_4	TGGGCGGCATCGCGTGACCA
FBXO17_5	AAGATGAGATTGCGGCCGAA
FBXW7_1	AAGAGCGGACCTCAGAACCA
FBXW7_2	AGGAGAACTCAAATCTCCTA
FBXW7_3	GTTGGAGTAGAACCTAGACC
FBXW7_4	TGAACATGGTACAAGCCCAG
FBXW7_5	ACAGAATTGATACTAACTGG
FBXW9_1	AGCGCCCATCCTCTGCCCAG
FBXW9_2	AGGAGCCGGAGCACACGCGG
FBXW9_3	CGTACGCGCGCCCTACCCAG
FBXW9_4	TCGGCGGTCCACCACCACCA
FBXW9_5	CCATCTACGACCCCAGAGGT
FEM1A_1	AAGGCCCGCATGGAACGTGA
FEM1A_2	CAGGGAGGTCAACACCACCG
FEM1A_3	GTAGGAGAAGAGTTCCGCGA
FEM1A_4	GTCACGCTGGCCGCGAGCAG
FEM1A_5	ATTGCTGGGAGCTACGTATG
FEM1C_1	AGAATACTACATGTGTAGGG
FEM1C_2	GCAGAAGCGGCCCATAAAGG
FEM1C_3	TGATGGCGAAACCATTGAGG

FEM1C_4	TTAAGAGCAGCGATATGCAG
FEM1C_5	AATGGCTTATGATTATGCCA
FEN1_1	GAGCCGCCAAGGCAGCACCC
FEN1_2	GGCTGGCAAAGTCTATGCTG
FEN1_3	TCTGAGGAGCGAATCCGCAG
FEN1_4	TGGCCAAACGCAGTGAGCGG
FEN1_5	GACTACTGTGAGAGTATCCG
FERD3L_1	CATGAAGGAGATATAGACGA
FERD3L_2	GAGGAGTGCGAAGTGGACCA
FERD3L_3	GGAGTGCGAAGTGGACCAGG
FERD3L_4	TATAGACGATGGCCAGGCGG
FERD3L_5	CCCTATTAGGCCGCCCCAAG
FEV_1	AGGACGGGAAGAACCCGAGC
FEV_2	CGGCCAGTAGGAGAAGCCGG
FEV_3	GAACGCCGGCTGCATCGCGT
FEV_4	GACGGGAAGAACCCGAGCTG
FEV_5	CGAGTTCAAGCTCACGGACC
FEZF1_1	ACTCATACCCGAATACACGC
FEZF1_2	AGAGGAGGCAGCGTCATCGG
FEZF1_3	CCTGCAGGGAGCCTTACCCA
FEZF1_4	GCACAAGATCATTCACACGC
FEZF1_5	AAAAATCGCGTTCAAAACCT
FEZF2_1	ACGCATATCCGCATCCACGC
FEZF2_2	CCTAGTCAGGTCCTTTGCGG
FEZF2_3	GAAGTAGTAGAGCGAGCCGC
FEZF2_4	GGCTTGATGACCCTGCCCGC
FEZF2_5	CCGGCCTGGAGCGCTAGAGG
FIGLA_1	AGTGGACGAGTAGCCGCCCG
FIGLA_2	GCCGCTCCAGCACCAACTGG
FIGLA_3	GCGGCGTGTGGCCAACGCCA
FIGLA_4	GTGGACGAGTAGCCGCCCGA
FIGLA_5	AGGTGCGACTGAATATATAC
FIP1L1_1	ATCTTACCAGGTTTACGCCA
FIP1L1_2	CTAGTGTCGGAGCTGAGCGG
FIP1L1_3	TGTGCACATGCACGTCCCAT
FIP1L1_4	TTAGGCCGAAGACTGTACTA
FIP1L1_5	AAACAAAAGAGGATACGAAT
FIZ1_1	ACTGCAGTGAAACGGGACCC
FIZ1_2	AGGCACCTGAAGCTGCACCG
FIZ1_3	CAGGTGACTGGCCAGGGCCG
FIZ1_4	GCAGTGAAACGGGACCCTGG
FIZ1_5	GCACGCAAATGGGGGCAAGG
FKBP1A_1	ACCGGTGTAGTGCACCACGC
FKBP1A_2	GAAACCATCTCCCCAGGAGA
FKBP1A_3	GGCAAGCAGGAGGTGATCCG
FKBP1A_4	GGGCGCACCTTCCCCAAGCG
FKBP1A_5	AAGCCCTTTAAGTTTATGCT
FKBP1C_1	GAAGGTGCGCCAGTCTCCTG

FKBP1C_2	GGAAGGTGCGCCAGTCTCCT
FKBP1C_3	GGGAAGGTGCGCCAGTCTCC
FKBP2_1	GTCCATCTGCCTGAGCGCCG
FKBP2_2	TGCAACCCTGGTGTTCGAGG
FKBP2_3	TGTCCCATCAAATCGCGCAA
FKBP2_4	TGTGCCAAGGGAGAAGACAA
FKBP2_5	CAAAAGGAAGCTGCAGATCG
FKBP5_1	ATTGGCACTGTCCAGTCCAA
FKBP5_2	CACCACAGCGGCCTTCCAGG
FKBP5_3	GGCCAAAGAATCCTGGGAGA
FKBP5_4	GGGAGGCAAATACATGCAGG
FKBP5_5	AAAAAGAGAGTTGCATTCGA
FLI1_1	AGGCCGACATGACTGCCTCG
FLI1_2	GCAGAGCCTGGGCAATGCCG
FLI1_3	GGCGGCACTTACTTTCCCTG
FLI1_4	TCACTTTAGGGAGTCTCCGG
FLI1_5	ACTCAATCGTGAGGATTGGT
FLYWCH1_1	CAAGTGGACACGCTGCTCCG
FLYWCH1_2	GATCTGCAAGGTGCTGGCGA
FLYWCH1_3	GTACAAGCAGGAGAAGGCAG
FLYWCH1_4	TGACAGTCACCCGTCGGCCC
FLYWCH1_5	ACGAGTCCTTCCTCTACCGG
FMR1_1	AGGGAGAGCTTCCTGCGCAG
FMR1_2	GAAAGAGAAGCCAGACAGCG
FMR1_3	GTAGACCTTACAGAAATAGG
FMR1_4	TTAGCTAACCACCAACAGCA
FMR1_5	AGAAGCTAGTAAGCAGCTGG
FOS_1	CAGCCAAATGCCGCAACCGG
FOS_2	GACTGGGAGCCTCTGCACAG
FOS_3	GGGCTCGCCTGTCAACGCGC
FOS_4	GTTGTGAAGACCATGACAGG
FOS_5	GCTGACTGATACACTCCAAG
FOSB_1	ACAAACGAAGAAGTGTACGA
FOSB_2	CAGATATTGAGACTCGGCAG
FOSB_3	GGTCGGTCAGCTCCCTCCGC
FOSB_4	TCTTCCATGGCCCAGTCCCA
FOSB_5	CGAAGACAGATATTGAGACT
FOSL1_1	CGAGCGGAACAAGCTGGCTG
FOSL1_2	GAGGGTAGGTCAGAGGCCTG
FOSL1_3	GGGAGATACAAGGTACAGGG
FOSL1_4	TATTCCTTAGAAGTTCCACC
FOSL1_5	AGTACAGCCCCCCACAACCC
FOSL2_1	AGGAGAAGCGTCGCATCCGG
FOSL2_2	AGGTGTGGAGGTCACCACGA
FOSL2_3	GCCGAGTCCTACTCCAGCGG
FOSL2_4	GGACATGGAGGTGATCACTG
FOSL2_5	AGTGTGCAAGATTAGCCCCG
FOXA1_1	AAGGGCATGAAACCAGCGAC

FOXA1_2	GAGCTAGGAAGTGTTTAGGA
FOXA1_3	GTTGGACGGCGCGTACGCCA
FOXA1_4	TACGAGCGGCAACATGACCC
FOXA1_5	AACATGTCCTATGCCAACCC
FOXA2_1	AAGGGCACGAGCCGTCCGAC
FOXA2_2	GAACAGGTGATGCACTACCC
FOXA2_3	GTTGCCCGAGCCGCTGCCCA
FOXA2_4	TAGTAGCTGCTCCAGTCGGA
FOXA2_5	ATGAACATGTCGTCGTACGT
FOXA3_1	AGTGAGATATAGGAATACGG
FOXA3_2	CGAGTGGAGCTACTACCCGG
FOXA3_3	GCACGGGAAGGAGATGCCGA
FOXA3_4	GTGGAGCTACTACCCGGAGG
FOXA3_5	AGATGCCGAAGGGGTATCGG
FOXB1_1	ACAGGGAGAACACGCAGCGC
FOXB1_2	GAAGCCGCTGTGCCACGCGG
FOXB1_3	GCGGGAATACAAGATGCCTG
FOXB1_4	GGCCGAGTCGATCATGCCGG
FOXB1_5	CCATCGAGAACATCATCGCG
FOXB2_1	AAATAGTGTACCATGTGCGG
FOXB2_2	ACCGCGAGCACACACAGCGC
FOXB2_3	GGGAAGCACCAAGAGCGCGC
FOXB2_4	GTGGTGCATGACGGACGCCA
FOXB2_5	CGACCATACTCACTTGCACG
FOXC1_1	AAGGCCGGAGCTGAGCTCCG
FOXC1_2	GAGCTGCTGCTGGTGACCGG
FOXC1_3	GGAGTGGTGCCCTACCTCGG
FOXC1_4	TCGTCCCTGAGTCACGGCGG
FOXC1_5	ACAACATCATGACGTCGCTG
FOXC2_1	GAAGGTGGTGATCAAGAGCG
FOXC2_2	GCTGAAGCCAGGCAGCCCGT
FOXC2_3	GGTGATGACCGGCAGCGCCG
FOXC2_4	GTGGCCGGAATAGACGCCCA
FOXC2_5	CAAAAAGAAGGACGTGTCCA
FOXD1_1	CGAGGAAACAGACATCGACG
FOXD1_2	GCTGCTCCCACCCAACGCCG
FOXD1_3	GGAAACAGACATCGACGTGG
FOXD1_4	TAGCGGCGCCAAGAACCCGC
FOXD1_5	GCCGTTGTCGAACATGTCGG
FOXD2_1	ATAGACCACATCATGGGCCA
FOXD2_2	GAAGCCGGGCAGGAAAGCGC
FOXD2_3	GCACGCCTTCGCTTTCGCCG
FOXD2_4	GTGGCCAGCAACGGGAGCCA
FOXD2_5	AGCCGAGGCAGACTTAGCCG
FOXD3_1	GGACTTGATGAGCGACGCGG
FOXD3_2	GGAGGGATGACCCTCTCCGG
FOXD3_3	TTGCGGCCCAGCTCGCCCGA
FOXD3_4	TTTGCGGCCCAGCTCGCCCG

FOXD3_5	CAGGTTGCGATAGCCCCGCG
FOXD4_1	ACTCGGTGCCAAACTCTGAG
FOXD4_2	AGCGCGATGTACGAGGAGGG
FOXD4_3	CCGGGGGCCTACCCCAACAC
FOXD4_4	GCTGGAAACGCTTCCTACGC
FOXD4_5	CCAGCGCCACCAACCGACCC
FOXD4L1_1	CAGCCTCCGGGACTCCGATG
FOXD4L1_2	CCCTGCCGCAGCCAGTCCCG
FOXD4L1_3	GAACTCGGTGCCAAACTCTG
FOXD4L1_4	GGCGGGGTCCAGGCTCCAGT
FOXD4L1_5	ATTGTCGAACATGTCCTGGG
FOXD4L3_1	ACTTGGTGCCAAACTCTGAG
FOXD4L3_2	AGACGAGGTGGAAGACGAGG
FOXD4L3_3	ATTGTCGAACATGTCTTGGG
FOXD4L3_4	GAGAGCAGTAGGTAGCGAAG
FOXD4L3_5	AGAGTTTGGCACCAAGTTCA
FOXD4L4_1	ACTTGGTGCCAAACTCTGAA
FOXD4L4_2	GCCATTGTCGAACATGTCCT
FOXD4L4_3	GGAACTTGCGGCGGTAGTAT
FOXD4L4_4	TGAAGACGAGGTGGAAGACG
FOXD4L4_5	CCCAAGCAGAGGGCCTGGGT
FOXD4L5_1	AGCGTAAGGGCATCTCCCGG
FOXD4L5_2	CAAACTCTGAGGGGTCACTC
FOXD4L5_3	TAGAGCAGTCACTCCAGCCG
FOXD4L5_4	TGCGGCGGTAGTATGGGAAG
FOXD4L5_5	GTGCGCAGCAGGTAGAGGGA
FOXE1_1	CATGCTGCCGCTTATCCCGG
FOXE1_2	GCGCCCGTAGAAGTCCACCG
FOXE1_3	GTAGCCGGTGGTGGTAGCGG
FOXE1_4	TGGCGGCGTGGAGACCACGG
FOXE1_5	AGCCTGCATAGACGGCGCCC
FOXE3_1	AAGCCAGAGAACGCGGCGGG
FOXE3_2	AGGGAAGCCAGAGAACGCGG
FOXE3_3	GAGGCAGCCGGGCTTCGCGT
FOXE3_4	TCGACGCTGAACAGACGCGC
FOXE3_5	CAAAGCGTTCGGTGATGAAG
FOXF1_1	AGGAGGGCTCCTTTCGGCGG
FOXF1_2	GCGACTGCGAGTGATACCGC
FOXF1_3	GTGCATGGAAGAGGACGCCA
FOXF1_4	TCGAGGAGGGCTCCTTTCGG
FOXF1_5	AAGGCCAAGAAGACCAACGC
FOXF2_1	CCAAGCCGCTCACCACGCGG
FOXF2_2	CGGGTCCCAGATGACCACCG
FOXF2_3	GATGGCGCTCGCCATGGCCG
FOXF2_4	GCTGGAGTGCAGGCCTGCCG
FOXF2_5	AGCCCGCGGGCATCATGTCG
FOXG1_1	AAGGAGAAGGGCGCCGGCGC
FOXG1_2	AGCGCGTTGTAGCTGAACGG

FOXG1_3	GAGGGAGTAGGTCCCAGAGC
FOXG1_4	TTTACGACGGGACTGTCTGG
FOXG1_5	AGCGACGACGTGTTCATCGG
FOXH1_1	GACCGCCCAGAAGTTGCCCT
FOXH1_2	GCCAGCTTCAGTCTGCGGGA
FOXH1_3	GGGAGCGGCCTGAATCACCA
FOXH1_4	TTCTTCAGGGAAGACTACGA
FOXH1_5	AGCATCAAGTCCCTGCTAGG
FOXI1_1	AACCTCAGCAACCACAGCGG
FOXI1_2	ATAGTAGAGGTTCATCTCGG
FOXI1_3	GACAGAGAGCAGTCTCCCGG
FOXI1_4	TCAGCGGCCCTCCTTCGAGG
FOXI1_5	CAAAGGGAATTACTGGACCC
FOXI2_1	AGGCCCGGGACCCGAAGCGT
FOXI2_2	CTGAGCGTCAGCTTCCGCAG
FOXI2_3	GTGACTGAGGCGGAAGCCGG
FOXI2_4	TTGACAACGGGAACTTCCGA
FOXI2_5	CCATCCAGAGCGCGCCGCTG
FOXJ1_1	ACAGCCGCTGCCCAAGCGGG
FOXJ1_2	ACAGCCTGACCAGCCTGCAG
FOXJ1_3	GAGAAGGACGAACCAGGCAA
FOXJ1_4	GAGTAGGGAGGCTTCACGTG
FOXJ1_5	CCACGTCGTCGTGCACGTCG
FOXJ2_1	AGTAGTATCTTACCTTCCCA
FOXJ2_2	GCAGCCGCAAGTGTTCACCA
FOXJ2_3	GGAAGAGGACTTCTCCAGCA
FOXJ2_4	GGAGGCAAGCAAGAGCCCAC
FOXJ2_5	CCAGCGGTAAATCTCGCTGA
FOXJ3_1	CTGTCCAACAGTCATGGCAG
FOXJ3_2	GAAGTCCAACAGCACAAAGA
FOXJ3_3	GGCAATAGACACCAATCCGA
FOXJ3_4	TGGGATATGAGGATAACCAG
FOXJ3_5	AGGCTGGCATAACTGTATGG
FOXK1_1	AAAGCGGTAACTGGAGGAGC
FOXK1_2	ACTCACCTGATGGTGCCCGT
FOXK1_3	GCAGGCCATCTCCTCCGCCC
FOXK1_4	GCATGAGGAACTCGAACTCG
FOXK1_5	AACAGGCATTCCGGAAACGG
FOXK2_1	CTCACGCAGGCCCATGGCGG
FOXK2_2	GATAGTTCAGGCGATTACGA
FOXK2_3	GCCGCACCTGATGGTTCCCG
FOXK2_4	TTCAGGTGGAGACAGCCCGA
FOXK2_5	AGCTGCGCGTAGGAGTAAGG
FOXL1_1	AAGCGTTGAAAGGCGGACGG
FOXL1_2	AGGCAGCTAGAGCAGCCGCG
FOXL1_3	GACGCTGGTGACGCTGCCCA
FOXL1_4	GTACAGATACAGCATGGGCG
FOXL1_5	GCGTCCCCGAACGAGGACGC

FOXL2_1	AGCGCCACGTACGAGTACGG
FOXL2_2	ATTGTACGAGTTCACTACGC
FOXL2_3	GTACGAGTTCACTACGCCGG
FOXL2_4	TGTACGAGTTCACTACGCCG
FOXL2_5	AGAGACCGGTCGCACAGTCA
FOXM1_1	ACAGGAGTCTAATCAAGCAG
FOXM1_2	ATAGCCTATCCAACATCCAG
FOXM1_3	ATGGCCATGTAAGAGTAGGG
FOXM1_4	CCAGAAGGAGACCTTGCCAT
FOXM1_5	ACCAGGTATGAGCTGACCCG
FOXN1_1	AGACTGGAGGGCGAGCGCCA
FOXN1_2	AGAGTGGGAAATGTACCTGT
FOXN1_3	GATCCCAGTGGACGTGGCGG
FOXN1_4	GGAGCAGTACATATGCTGCA
FOXN1_5	ACACTATCAGTACCAGCGAA
FOXN2_1	ATGTACGAATTCCAGCAAGG
FOXN2_2	TAGCAAGCTGGTCCAAAGGA
FOXN2_3	TATCCCAAAGAATAGTCACG
FOXN2_4	TTGGAAGACCCTCACTTCCG
FOXN2_5	AGAAGACTAAAGGAGTATGG
FOXN3_1	GAAGGTACTGCCCGGCCAGA
FOXN3_2	GAGCGCGGGTCCTGAGCCGA
FOXN3_3	GTAACACTGACTCAGTCCAC
FOXN3_4	TATGCCTGAGGACAGGTGGG
FOXN3_5	CATCGTAGGGCATGTCAGAG
FOXN4_1	CAGGGTTGGCCATACTCCGG
FOXN4_2	GCTGTCGTGGCTCACGGCGG
FOXN4_3	GGGCCATAGAGGCCCACAGG
FOXN4_4	GGTGGCGTGAGTCAGCACGG
FOXN4_5	CACCGGAGTATGGCCAACCC
FOXO1_1	ACAGCGGCAGCCGAGGCCGA
FOXO1_2	GGTCAGGCGGTTCATACCCG
FOXO1_3	TTGGCAGCCGCGCTGCCCGA
FOXO1_4	TTGGTGGATGCTCAATCCAG
FOXO1_5	ACAGGTTGCCCCACGCGTTG
FOXO3_1	AGTAAGCAAGCCGTGCACGG
FOXO3_2	ATGGAGACAGCCCGCCGCCG
FOXO3_3	CGAGGAAATGTTCGTCGCGG
FOXO3_4	GCGGGCGGCGAAGATGGCAG
FOXO3_5	AGAGCGGAAAAGCCCCCCGG
FOXO4_1	ATATGCAGAACTCATCAGCC
FOXO4_2	GCTGTAACAGGTCCTCGGAA
FOXO4_3	GTTGGAGTGGCACCTTCGGG
FOXO4_4	TATCCATGGAGGCGGCCCGG
FOXO4_5	CCTTGAAGTAGGGTACAGTA
FOXO6_1	ATGGACACGGCCCTGCGCCG
FOXO6_2	GAAGCTGCGAGCGCATCAGG
FOXO6_3	GCTTGGGCCGACTTCCGCGG

FOXO6_4	GGAAAGCGAAGAGCTCTCGG
FOXO6_5	CCTTGAAGTAGGGCACGTAA
FOXP1_1	AACCACCAGCATGCACACGG
FOXP1_2	ACTGCTGTGATTGTTGCCTG
FOXP1_3	GAGAAAGCTTACCTTCCACG
FOXP1_4	GAGTGCGGCGGTCTTCGGGA
FOXP1_5	AGAGGAGGAGACACATGTCG
FOXP2_1	CCAATGTGGGAGCCATACGA
FOXP2_2	GAGACAGGACTTGTTGCTGA
FOXP2_3	GTGTCGAGTGCAAATGCAGG
FOXP2_4	GTTGGAGGAGGTAGTCGAGG
FOXP2_5	CCAGTCACCCCGATTACCCA
FOXP3_1	GAAGGTCTTCGAAGAGCCAG
FOXP3_2	GGAGGGTGCCACCATGACTA
FOXP3_3	TCATGGCTGGGCTCTCCAGG
FOXP3_4	TTTGGGTGCAGCCCTCCAGC
FOXP3_5	ACCCAGGCATCATCCGACAA
FOXP4_1	ACGGGCAGGGAAGTGACCAC
FOXP4_2	AGCGGCAAACGAGGTAGCGG
FOXP4_3	GATCTGCTGCATCTGTTGCG
FOXP4_4	GGAGTACTACAAGAAGCAGC
FOXP4_5	ACCACGGGTGCAGACAGCAA
FOXQ1_1	CCACTGAAGCGTCGTCCCGG
FOXQ1_2	GAGCTGGAGAACTTGCCCGC
FOXQ1_3	GCAGCAAGCCATATACGCGG
FOXQ1_4	GGAGCTGGAGAACTTGCCCG
FOXQ1_5	GAGCGAAAGGTTGTGGCGCA
FOXR1_1	AAACCCAACCCTGATAAGGA
FOXR1_2	AAAGGCAAACCGCTTCCGAG
FOXR1_3	GGGACACCGCCGCTTTGCGG
FOXR1_4	TCGGCAAGCCAGCAGCCAGG
FOXR1_5	AGCTGGAGGTCTCAGGACGT
FOXR2_1	ACCAAGAGAAGTCCTGGCAA
FOXR2_2	AGGTGGCTACAATTGAGAGG
FOXR2_3	TAAAGGAGACTGCTCACTGG
FOXR2_4	TGTGAGCTCAAAGTCACTGG
FOXR2_5	AGCCCCCACAAAAAGACGAA
FOXS1_1	AGGGCGACCAGCCAGGACCC
FOXS1_2	CCGGCAGGAAAGCCAAACGC
FOXS1_3	GATGACCGCAAGCCAGGCAA
FOXS1_4	TGGCCATCCAGAGCTCACCG
FOXS1_5	CCCAAGGGCCTAAGCTTTGG
FSBP_1	AGGCCCTGTGCTGTTCGAGG
FSBP_2	GAGACAGGAACCACATACAA
FSBP_3	GATCCTGCAAATGTTGAAAG
FSBP_4	TATGTTCAGGAGAAGAGGGA
FSBP_5	AGAGGAAAGTGAAGGCGACA
FTSJ3_1	GAAGGAGCTGAAGACCTGGA

FTSJ3_2	TGCTGAAAGATCCATAGCAG
FTSJ3_3	TGGACCTGTGTGCTGCGCCA
FTSJ3_4	TTTACTGTCAACCTTGTCAG
FTSJ3_5	AGGGTCTAAGGGACCAAAAG
FUBP1_1	ACAGTGGTGGCCTTCCAGAA
FUBP1_2	CCTGGCTTCCATCATGGCGA
FUBP1_3	GAGTTAATTCGTGATCAAGG
FUBP1_4	TCTGGCTCAGCTGGTGGCGG
FUBP1_5	ATGATGGGACAACACCCGAA
FUBP3_1	AAGAGGCTTGTCTGCTCCCG
FUBP3_2	GCATGCTGACACCGATCCGG
FUBP3_3	GTGGGTCTGGTCATCGGCAG
FUBP3_4	TAATGGCGGAGCTGGTGCAG
FUBP3_5	AAAGACCAAGCTGACTTTCG
FUS_1	AACACCACCGTACCTTCCCG
FUS_2	ACTGTAACTCTGCTGTCCGT
FUS_3	ATACCCAACAAGCAACCCAA
FUS_4	GGTGGACAGCAGCAAAGCTA
FUS_5	CAAAGCTATAATCCCCCTCA
FXR1_1	AACATTCAGAATCTACGGAG
FXR1_2	AATAGTGGACAAATCTGGTG
FXR1_3	GGAAGAGGCAGAGGTCGTCG
FXR1_4	GTTGGCACTAAAGAAAGCAT
FXR1_5	CAAGGTTCTAGGTCTTATAG
FXR2_1	CAGATCCTCGGGCACAGCCA
FXR2_2	GTTGGAGCCGCGCACCTCGA
FXR2_3	TAAAGAGTTCAAGAAAGCCC
FXR2_4	TAAGTAGCAGTTTGGTGCGC
FXR2_5	AACTGGCCGAAGTCGCTCCA
FYTTD1_1	AATTCAGGATGAGAGTGCGA
FYTTD1_2	ACAGCAGTACGAGTTAATCG
FYTTD1_3	CGGCGGCGAAGAAGTCGCCG
FYTTD1_4	TTGACATGGGACCTATCCCA
FYTTD1_5	AAGACTAGTCTGAATCGTAG
G2E3_1	AAATTACCCAGACAGTCACC
G2E3_2	ACCTCAGTAGATCTTTACTC
G2E3_3	GCCATTTCTTTAGTTCACGG
G2E3_4	GTGGGTTTAAATCCAGCTGG
G2E3_5	AACCAAGCGTTCTTACAACA
GABPA_1	ACAACCTCAACAGTGTCCGC
GABPA_2	CCAAAGTACAAAGAGCGCCG
GABPA_3	TCTTATTGGATACAGTGCAG
GABPA_4	TGACTGGCTGGGCAATTCCA
GABPA_5	AAGCAGAGTGCACAGAAGAA
GABPB1_1	ACAGAGGTACTGCTGCGAGC
GABPB1_2	GATGGGCTGACCAATTCCAC
GABPB1_3	TAAAGATGACAGCTCTCCAT
GABPB1_4	TGGTCATTATTCCACCACAG

GABPB1_5	AAACCTCTACTATGCTGGCA
GADD45A_1	AAAGTCGCTACATGGATCAA
GADD45A_2	ACTTTGGAGGAATTCTCGGC
GADD45A_3	AGAGCAGGAGCTCCGCCAGC
GADD45A_4	TCAGCGCACGATCACTGTCG
GADD45A_5	GCAGCAAAACGCCTGGATCA
GADD45B_1	GAAGAGCCACGGCTTGGTGG
GADD45B_2	GATGCAGACGGTGACCGCCG
GADD45B_3	GCGCCAGGATCGCCTCACAG
GADD45B_4	GGAGAGCCGGCCGAGACCCA
GADD45B_5	ACAACGACATCAACATCGTG
GADD45G_1	AAACAGGCTGAGCTTCTCCA
GADD45G_2	GCTGCGGGTGAGGAGGACGA
GADD45G_3	TCTGGAAGAAGTCCGCGGCC
GADD45G_4	TTTGGCTGACTCGTAGACGC
GADD45G_5	CACAGAAGGTCACATTGTCG
GATA1_1	AATAGGCTGCTGAATTGAGG
GATA1_2	AGACGACCACCACGACACTG
GATA1_3	GAGCAACAGCCACTCCACTG
GATA1_4	GAGCAGGATCCACAAACTGG
GATA1_5	GCGGGTGGGACACACAGTTG
GATA2_1	ATGGCGCCGAAACGCCAACG
GATA2_2	TCTGGAGGCAGCAGCTGCGC
GATA2_3	TGGGCAGGATGTGTCCGGAG
GATA2_4	TGGGCGGCACATCTGGCCTC
GATA2_5	AAGACGTCCACCTCGTCTGG
GATA3_1	AGACCACCACAACCACACTC
GATA3_2	GAACTTACCAGCCTTCGCTT
GATA3_3	GCTGGACGGCGGCAAAGCCC
GATA3_4	GTGCTGCCCGTTGAGCACGG
GATA3_5	AGGTACCCTCCGACCCACCA
GATA4_1	CGCTCATCAAGCCTCAGCGC
GATA4_2	GAGAGCCTTCCTCCCGCCAG
GATA4_3	GCTGTGGCGCCGCAATGCGG
GATA4_4	GTAGGACAGGCCCAGCACGG
GATA4_5	AGCCGCTTACACCCCGCCGC
GATA5_1	ACACGACCAACACCACGCTG
GATA5_2	GAAGCCAAAGACCATCGCCA
GATA5_3	GAAGGCCGACGAATCCGCGG
GATA5_4	GGGCCCTGTCCACACCGCTG
GATA5_5	ACAGGTAGGACAGCATCGAG
GATA6_1	GACGCCTCAGCTCGACACGG
GATA6_2	GTTTCTAGCCTTCATCACGG
GATA6_3	TCCCGCACCAGTCATCACCG
GATA6_4	TCTCGGACAGGTCCTCCAGC
GATA6_5	CCCCTACTCGCCCTACGTGG
GATAD1_1	AGAAGAGTGCAGCACTGACG
GATAD1_2	CATGTGGAAGAAGGGAGCGC

GATAD1_3	GGCCAGTGCAATGATGGCAG
GATAD1_4	TGTGGAAGAAGGGAGCGCAG
GATAD1_5	AGAGTAAGCAGGAAATTCAC
GATAD2A_1	ACAGGCACGCACCTTCTGGG
GATAD2A_2	AGTGAATGGGCTGACCACGG
GATAD2A_3	GGCAACAGAGGCCACGGCCA
GATAD2A_4	GGTGCTCCCAACAGAACCTG
GATAD2A_5	CCACAGAGGCCACACTAGTG
GATAD2B_1	AAAGCTGTGCCTCACTTCTG
GATAD2B_2	AAGGTTCATATAGATGGCAG
GATAD2B_3	ATAGGCCCTGCTGTCCCACA
GATAD2B_4	GAGACAACAGGACTGCTGGA
GATAD2B_5	AACTCGCTATTGGCTGCACT
GBX1_1	AAAGTCTCTGAGAAGTGCGG
GBX1_2	AACAAGATGCAGCGGGCCGG
GBX1_3	GAAGGTGTACAGCTCAGATG
GBX1_4	GCACCAACAAATGGAGCAGG
GBX1_5	CGGCCCGATTAGGGAGTCGA
GBX2_1	CAAGGAGGAGAGCTTCTCGC
GBX2_2	CGGTAACTTCGACAAGGCGG
GBX2_3	CTACGGGCAAGAACCGGCGG
GBX2_4	GATGATGATGCAGCGCCCGC
GBX2_5	AGGGAAAGACGAGTCAAAGG
GCDH_1	ATAGATAGGGTGCATGACGA
GCDH_2	GTTTGACTGGCAGGACCCGC
GCDH_3	TCCAGGGCAAGTTCTCGCTG
GCDH_4	TCGCACGTGGGTCTCGTCGG
GCDH_5	ACAGGCATGATCATCATGGA
GCFC2_1	AAACTCACCGCTCACACCTG
GCFC2_2	ACTTAGACAAAGGATGGCTG
GCFC2_3	AGAGACATAGATCTTTCCTG
GCFC2_4	GCTGATTCCAGCGACAGCGA
GCFC2_5	AAAGATGAGACATCCACAAG
GCM1_1	AAACCGACTGGTTCCAGGAG
GCM1_2	ATGATCAGAGTAGACTCCGG
GCM1_3	CAAACACATCTACAGCTCGG
GCM1_4	TCTTCATAAAGATTACCCGC
GCM1_5	AAGCAGGTCTCCACTACTGT
GCM2_1	AAAGAGAATTCGAGAATCCG
GCM2_2	CAGGCCTGCACCCTGCCCGA
GCM2_3	GCGAGTTGGGCAGATGCCGG
GCM2_4	TATCCGCTGTGCCCTCGACA
GCM2_5	ACTCTCGGAATTGGTCAAAG
GDAP2_1	GACCAGCGAATACAATCACC
GDAP2_2	GAGAAACCTGGTGCTCCAGA
GDAP2_3	GGTGGAGGCTGTCCACATGG
GDAP2_4	GTGGATACACTACCAAGCTG
GDAP2_5	AAGGATTCAATCTAGCTGCC

GFI1_1	ACAGCACTTCAAATGCAGGC
GFI1_2	AGAGGAAGGTGGACCTCCGA
GFI1_3	CCGGTACATTCTCTAAACGG
GFI1_4	TGGGCACATTGACTTCTCCG
GFI1_5	ACGTGCACCTCGAGCCCGTG
GFI1B_1	GCACACGCACGTCCACTCCC
GFI1B_2	GCGGAGGCTGAAGTCCAAGG
GFI1B_3	GGTTTGGGAATAGAGTGCTA
GFI1B_4	GTGGTACGCATCCATGCCTG
GFI1B_5	CCTTGTTGCACTTCACACAG
GLI1_1	AGAGACCAACAGCTGCACCG
GLI1_2	CTGGACATATCACCTTCCAA
GLI1_3	GATGTGGGATCATCCCACCC
GLI1_4	TCGCGATGCACATCTCCAGG
GLI1_5	CGGCGTTCAAGAGAGACTGG
GLI2_1	ATTGACATGCGACACCAGGA
GLI2_2	GCAGCAGCGGTAGCTGCCCA
GLI2_3	GGAGGTAGTGCTCCATGTGG
GLI2_4	TTACCACCAGATGACCCTCG
GLI2_5	CAGGGATGACTGTAAGCAGG
GLI3_1	ATTGATGCCAGACATCATGA
GLI3_2	CCATGGCAAACACCGTCCCG
GLI3_3	CGTGGGCTGAGCCCTACAGA
GLI3_4	GTAATGAGAAGACATCATGG
GLI3_5	AGGGCAGGTCCGGATACGTA
GLI4_1	ACTCGCGGTAGTGCACCCGC
GLI4_2	ATACTTGAAACTCTTGCCGC
GLI4_3	GAGTACCATGTTGATGCCCA
GLI4_4	GCTGCGTGAAGTGCGAGCTG
GLI4_5	AACTTCGGTCGGAGCCGGCA
GLIS1_1	ATCCCGGGTGCTCTCAGCCA
GLIS1_2	GCAACTTGAAGCCTGCCGGA
GLIS1_3	GCATGCGGGCCCTGACACCG
GLIS1_4	GGGCCTTGACGTGCTTGCGG
GLIS1_5	CAGACACTCGGTCAGGACGT
GLIS2_1	ACACCATCCAAATAGCGCAG
GLIS2_2	ACAGGCTGAGGTCCACGAGA
GLIS2_3	AGAGAAGCGGGAGAGGACGC
GLIS2_4	TGGCGTTGAAACCTCGGCCA
GLIS2_5	GACAGGCTGAGGTCCACGAG
GLIS3_1	GACCTGGTGGATGAGTGCCG
GLIS3_2	GATGTGCTGCCACAGGGCGA
GLIS3_3	GTGACCGCGCCAAACACCAG
GLIS3_4	TTTCCAATGGACTTGCACTG
GLIS3_5	AGGCAGCTGCAACAATCTAG
GLYR1_1	AAGAAGCATTTCTTTCCGCG
GLYR1_2	ATGGCAGCCAACCGCAAGCG
GLYR1_3	GAATCGACGTAATTCCAGTG

GLYR1_4	GCGGCTGTGAGTCTGCGGCT
GLYR1_5	CACACCCACAGACAAAAAGT
GMEB1_1	AAGAGAGCTATTCGTCTGGG
GMEB1_2	GACATACAAACTTCTTCCAG
GMEB1_3	GCAGAAGTGAGTGTCCCAGT
GMEB1_4	TTTCTTTACACCCTCCTCCG
GMEB1_5	AGCTGGGTCGGAGAACAACA
GMEB2_1	CGAGCATGTAATCAGCCCAA
GMEB2_2	GACAACTCCGGACACTGCAG
GMEB2_3	GCAGAGCCAACCTCATCTGG
GMEB2_4	TGTGCACCTACCGTCGGCTG
GMEB2_5	GGACGGCTTCCTACCTAACA
GON4L_1	AATGACACGCTCTAAACTGA
GON4L_2	ACAGCATAAAGGCAATCAAG
GON4L_3	AGGAATCATCTTCTTCAAGG
GON4L_4	GAGAGGCACAAACATCTGGC
GON4L_5	AGTAATCACAAATGAACACG
GPANK1_1	GATTGGCACGGCCCTCACCC
GPANK1_2	GCTCGAGCAGCACACATCAG
GPANK1_3	GCTGCTGCCCATCCTTCCAG
GPANK1_4	GGTGAGCAGTGGATGTGCGG
GPANK1_5	CAGGGCCTCATAGAAAGCTC
GPBP1_1	AACATGTGCTCCCTTAACTG
GPBP1_2	CATGAGTCCACATTTGGCGT
GPBP1_3	GAGAACGGGAACTTCCACCA
GPBP1_4	GATCTGCTGGGAAATCACTG
GPBP1_5	GCTATTGGGCGTCCTAATGG
GPN1_1	AACAGACACACTGGGTGCCG
GPN1_2	CATTGACCACAGCTTTGCAG
GPN1_3	TATGGACTTGGACCCAATGG
GPN1_4	TCCAGGTTGATCACATACGG
GPN1_5	TATGTGTTGATTGACACACC
GPR75-ASB3	CAAACACTGCTGAGTAAACA
GPR75-ASB3	GCATACTTACCTAAAAACAA
GPR75-ASB3	GCGGCCTCTCACCTGCCGGG
GPR75-ASB3	GTCGATGTTGCTGATAACAG
GPR75-ASB3	AATGGATCCCATTCTATGTG
GPS2_1	AACGGAGGCGAAAGGAACAG
GPS2_2	AATGTCATTAGAGGAGACCA
GPS2_3	AATGTGCCGGTGCAGCGCCC
GPS2_4	CCTGGAGGACACAATCGCCC
GPS2_5	ACCGGCACATTATGATGGAG
GRHL1_1	AAGACAGAAACCCAGCCACA
GRHL1_2	AAGCACTTTATCCACAGCGG
GRHL1_3	GATGCCTGGCATGAATTCAG
GRHL1_4	GTCATCAACAGCAAAGCCAG
GRHL1_5	AAAACCACCACCCGCTCAGT
GRHL2_1	AACACGGATGATGAACGAGA

GRHL2_2	AAGCAGAGGGTCCTTGACAT
GRHL2_3	AATGGAGGGTCACTGGGCAT
GRHL2_4	GAGCCTGGGACTCACCTCTG
GRHL2_5	AGATGGGGAAGAGCAACGAG
GRHL3_1	AATGACCCGCTGCTTGGCAG
GRHL3_2	CAAGTCAGGCGAGTCACCCA
GRHL3_3	TAACCTGATGAGCTTGGAGG
GRHL3_4	TGTGTGGAACGTGAATGAAG
GRHL3_5	AGTGACTTTGAATACACCCT
GSC_1	CAAGGACTCGGTGTTGCCGG
GSC_2	GAAGGCGCCATAGTCCGAGG
GSC_3	GCCGCCTCCATTTGGCGCGG
GSC_4	GGGACACCAGCACCGAACCG
GSC_5	GATGCTGCCCTACATGAACG
GSC2_1	CGCGAAGCCAGAGGAGCCCG
GSC2_2	GCTGGAGAGGATGTGCTCGA
GSC2_3	GGTGTCGCCATTTGGCCCGG
GSC2_4	TCTCTGGGTGCGCCAGCCGG
GSC2_5	CCGCAGGCGCTCGTCTGGCG
GSE1_1	AATCCTGCACAACTGAGCTG
GSE1_2	AGTGGCCGGGTCCAGGCCGA
GSE1_3	GTGGAAAGCATCCCTAGCGA
GSE1_4	TGGAGCGATGGTCACCACGG
GSE1_5	AGGTCGGGAACGCCTCATTG
GSG2_1	AAGGAGAATAACAACCGCTG
GSG2_2	AGAAGGAGAATAACAACCGC
GSG2_3	CGGACGGCAGGAGACAGCGG
GSG2_4	GGTGTGCTGCACTTCTGCGG
GSG2_5	GCAGCCTCAGCCGTCCCGAG
GSPT1_1	ATAAGTGAAGCAGAGCCAGG
GSPT1_2	ATGGATCCGGGCAGTGGCGG
GSPT1_3	CCAGCGGGAGAACCTCAGCG
GSPT1_4	TTTCTGTTAAACAGCACCTG
GSPT1_5	AATCCCAAAACCTAAGTCTG
GSPT2_1	AATGGTAATCCAGTGTACCA
GSPT2_2	AGTAAGTGAAGCCGAGCCTG
GSPT2_3	GGATGGAGTCTCCTCTGCGG
GSPT2_4	TACGGCTGAAAGCCGAGCTG
GSPT2_5	ACTAACGGTTCCTCTCGGGA
GSX1_1	AAGGGCAGCAACCATCGTGG
GSX1_2	ATCCCGGTCGTCCTTCCCTG
GSX1_3	GGAGGTCTGGTAGAGCGCGG
GSX1_4	GGCGAGAAGAAGGCGCCCGA
GSX1_5	AGTACTGCGAGCCGAAGGGT
GSX2_1	ACGCAGAGGAACAGTCACGC
GSX2_2	GATGCGCATGGTTCACCCGC
GSX2_3	GGATGAAGAAATCCGGCCCG
GSX2_4	GGTGCACTACGCGCGCTCCG

GSX2_5	AGGTGCGAAGTGACGCAGAG
GTF2A1_1	AACTGGCCAGCAGCAACCGC
GTF2A1_2	AGTGGATGAACAAGTACTGA
GTF2A1_3	ATACCACAAATGCAGCCTGG
GTF2A1_4	CTCGGCAAATACAAACACCG
GTF2A1_5	GAATCTGGAACAATAACTTG
GTF2A1L_1	CATGGCCTGCCTCAACCCGG
GTF2A1L_2	AGATACAAGTATATCACTGG
GTF2A1L_3	CTTGGAAGAGTTCTACCAGC
GTF2A1L_4	GCTGAATATCAGTCACGTGC
GTF2A1L_5	GGTACATGAATGGGATAACC
GTF2A2_1	CCTAGATGAGCTCATACAGG
GTF2A2_2	GAACTTGAAGGGCAAGTTGG
GTF2A2_3	TAAATGCAGCACTGGCTCAG
GTF2A2_4	TAAGGCTATAAATGCAGCAC
GTF2A2_5	AACTTGAAGGGCAAGTTGGG
GTF2B_1	AAATCTCCATCACTCAGAAG
GTF2B_2	ATATGAACAGAAGAGCCTGA
GTF2B_3	GGCGGGACATACTAACCGGC
GTF2B_4	TTAGTGGAGGACTACAGAGC
GTF2B_5	ACAAAAGATCCATCTCGAGT
GTF2E1_1	AAATCCCAGCCCTGAAACAG
GTF2E1_2	GATCAAGATGTCCAAGGCCA
GTF2E1_3	GCACAGCCACAGCTGCCGGA
GTF2E1_4	GGAGCTGCTAGCCTAGCAGG
GTF2E1_5	GAAGTAGTAGTTATGGCGAG
GTF2E2_1	GAAACAATGGCTAATGACTG
GTF2E2_2	TGATAGACACGGCATCAGCG
GTF2E2_3	TTAGATCAGCATGACCAGCG
GTF2E2_4	TTGGACTCAAGCAGAAACAA
GTF2E2_5	AAAACAAAGGTAGAACATGG
GTF2F1_1	AAGAAGGCGCCGCTGGCCAA
GTF2F1_2	AGAAGGAGAAACGTGGCCGC
GTF2F1_3	GCATCGCACGCTCACTGCCG
GTF2F1_4	GTTCAACCGCAAGCTTCGGG
GTF2F1_5	ACAAAGTCAACTTTGCTACG
GTF2F2_1	ACCGGCGCCAAACAGAACAC
GTF2F2_2	AGTGAGGCTATCACAACAGC
GTF2F2_3	TAGGACTCAAGGAAGGACTG
GTF2F2_4	TCTCCTTTAGGACTCAAGGA
GTF2F2_5	AAGACAAATGGATGTTCTCT
GTF2H1_1	ATTGGAAGCAGATGGCACAG
GTF2H1_2	ATTTAACCATCACAGTGCCA
GTF2H1_3	GAGTCAAGTGATCAGTGCTG
GTF2H1_4	TCAGCTGGTCCTACATGCAG
GTF2H1_5	ATATTTAGGACCTATCCAGC
GTF2H2_1	AACGTCTTTGAAGAAAGCTG
GTF2H2_2	ATTAGATCATAAATATTAGA

GTF2H2_3	CATCTACTACCACATAAAGG
GTF2H2_4	GGCTTTCATCTAAAATAACA
GTF2H2_5	AAAGAAGCAATGGTGTGCTG
GTF2H3_1	ACAGCAAAGGGCTTTGGCCA
GTF2H3_2	CACGGGAGGACTGTACCTGA
GTF2H3_3	CAGAGGGATTAAATTCAGGA
GTF2H3_4	TTGATGCCAACCCAATTTGG
GTF2H3_5	AGACATTCATAGAATGAACA
GTF2H4_1	AAGTGACATACCCACCCAGA
GTF2H4_2	AATCTGCAGGAATTCTTAGG
GTF2H4_3	CATCACCCAGTTCTTAGCCA
GTF2H4_4	GGGACCAGACAAGCATGCCC
GTF2H4_5	ACAATCGGTCCAATACCCCA
GTF2H5_1	ATTCACCCACTCGCTCCTGG
GTF2H5_2	CTTGGATGAGTCCAATGCCC
GTF2H5_3	GTACAGCAGAAACTGCTTCA
GTF2H5_4	TGGATGAGTCCAATGCCCTG
GTF2H5_5	AACATGGTCAACGTCTTGAA
GTF2I_1	AAAGTCGATGGTCTTCGGAA
GTF2I_2	AGAACCGTTAGTCTGGGTCG
GTF2I_3	AGGTCAATACTATCTCCAGG
GTF2I_4	GTATGTGGAAGGCTTGCCAG
GTF2I_5	AACACTCAGAAAAACAGTTG
GTF2IRD1_1	GCCTCCGGATCCTTCCACGG
GTF2IRD1_2	GCTGTCCAAACTGAACGCCG
GTF2IRD1_3	TGAGCTTGAAGCGGATGCGG
GTF2IRD1_4	TGGAGTCTAAGGCAGACACG
GTF2IRD1_5	GGGGCCGGATCACGTCACCG
GTF2IRD2_1	AACCCATGGAAGATTCTGGT
GTF2IRD2_2	AGGAAGATCACATTCAACCC
GTF2IRD2_3	CTGGTCGAAATTAGTAAGCG
GTF2IRD2_4	GTATTTCCGTTTCGCCTGAG
GTF2IRD2_5	AAAACGCAAGATAGACCAGG
GTF2IRD2B_[illegible]	CTGGTCGAGATTAGTAAGCG
GTF2IRD2B_[illegible]	GTATTTCCGTTTCGCCCGAG
GTF2IRD2B_[illegible]	GTCCTCTACAGGCTGCACGG
GTF2IRD2B_[illegible]	AAACGCAAGATAGACCAGGA
GTF2IRD2B_[illegible]	ACTGATTTCCAGATAGCCGG
GTF3A_1	CTATCCGTACACACCCTCGT
GTF3A_2	GCCGACGCGTTCATTGCAGC
GTF3A_3	GTGTGGCAAGGCCTTCATCA
GTF3A_4	TTGCCAATGCAGGTGTACCC
GTF3A_5	CTCCTGTGTGAGTCAGAATG
GTF3C2_1	AAACAGAAACCACACTGCCG
GTF3C2_2	AAGAGGTGGATGGTGCTCCA
GTF3C2_3	ACTTGGGCAGCCCTTCGGCG
GTF3C2_4	GAATCCTCAAATCCAGGCAA
GTF3C2_5	GGCACTGACCACACTCAGAG

GTF3C4_1	AAACAGGCAGTCTGTTCCAA
GTF3C4_2	CCGTGGACACAGACACGCGG
GTF3C4_3	TGGTCCTCGGACCAAGCCAG
GTF3C4_4	TGTGGAGATGCACTTGACCA
GTF3C4_5	AACAACGAATGCCGGGACGT
GTF3C5_1	ACGCACAGGGATGGCGGCGG
GTF3C5_2	GCATCAGGAAGAGAACGAGG
GTF3C5_3	GGTGCCCAAGCAGCCACTGG
GTF3C5_4	TGTAGAGCGGCAGCTCCTGG
GTF3C5_5	CACAACGGATTCGGAAATCG
GTF3C6_1	GACGAGCGGAGTCCAGAGGA
GTF3C6_2	GGCGGACGAGCGGAGTCCAG
GTF3C6_3	GGTGGAATGGCTGCAAATAA
GTF3C6_4	TGAGAGGCCCATTCTGCAAG
GTF3C6_5	CATCCCCTCCAAATTAGGTG
GZF1_1	ATGACAAGGAAAGACTTCCA
GZF1_2	CCGACAAGTCCACTCTTCGG
GZF1_3	GCAGCGGTACACCACCTCGG
GZF1_4	GCTGGTACAGTGAATTCCGC
GZF1_5	AAATCAGGAGAGCTAGTGGA
H1F0_1	CAGGCCGAGAAGAACCGCGC
H1F0_2	GGTCTGCTTGAGGACACCGG
H1F0_3	GGTGTCCTCAAGCAGACCAA
H1F0_4	TATCAAGAGCCACTACAAGG
H1F0_5	CGATCATGTCTGAATACTTG
H1FNT_1	AGCCCAAGAAGCCCGCACAG
H1FNT_2	CCAGACCCTGAAGTAGCCGG
H1FNT_3	GAACGCGAGGGCGAAAGCCA
H1FNT_4	TGAAGGAAGACACCACGCCG
H1FNT_5	CAGAGTCAGCCCTTTGTGAG
H1FOO_1	AAGGAGGACCCTCCCAACGT
H1FOO_2	CATCCAAAGTGTCCAGCCAG
H1FOO_3	GAGCCACAGCAGCCTCCCGG
H1FOO_4	GAGGCCACGGCGCATGCCAG
H1FOO_5	CCTCAACTCCAAAGCCAGGG
H1FX_1	AATGGCCAAGAAGGTGACCA
H1FX_2	CCTGCCAGTGACGACCGCCG
H1FX_3	GCGGACAAGAAGCCCGCCAG
H1FX_4	GCTGGCCAAGATCTACACCG
H1FX_5	GTTGAGCTTGAAGGAACCGT
H2AFJ_1	CAAATCCCGCTCCTCCCGCG
H2AFJ_2	GGGCGGCAAAGTGCGAGCAA
H2AFJ_3	GGTGTTGGAGTACCTTACGG
H2AFJ_4	TACGGCGGAGATCCTGGAGC
H2AFJ_5	GCTGCGCAAAGGGAACTACG
H2AFV_1	AAGACTCGCACCACAAGCCA
H2AFV_2	AGCACTGTACACGGCAGCAG
H2AFV_3	GCGAGATACTGCCTTAGCCT

H2AFV_4	TATCAAGGCTACCATAGCTG
H2AFV_5	CAGAATCGCAGCACTGTACA
H2AFX_1	AAGAAGACCAGCGCCACCGT
H2AFX_2	CAAGAAGACCAGCGCCACCG
H2AFX_3	GGGCGGCAAGAAGGCCACCC
H2AFX_4	GGTGGCCTTCTTGCCGCCCG
H2AFX_5	CCGCGGCAAGACTGGCGGCA
H2AFY_1	AAGGAGTCACCATAGCCAGT
H2AFY_2	AGCAGGAGTCATCTTTCCCG
H2AFY_3	AGCGCCGACAGCACAACCGA
H2AFY_4	GCAGCGAGAGACAACAAGAA
H2AFY_5	CAGGAGTCATCTTTCCCGTG
H2AFY2_1	AATCCAAGGCTGCCAAACCA
H2AFY2_2	ACTGAAGCAAGATGTCGGGC
H2AFY2_3	ATTGGCCGGCAATGCCGCGA
H2AFY2_4	TTACCGACAGTCCAAACCAA
H2AFY2_5	AGCCCCGAGACACATCTTGC
H2AFZ_1	ACCCATTTACCTCTGCGGTG
H2AFZ_2	CTCCGGAAAGGCCAAGACAA
H2AFZ_3	GGCTGGAAAGGACTCCGGAA
H2AFZ_4	TGTCGATGAATACGGCCCAC
H2AFZ_5	ACAACTACTGACCTGCAAGC
H2BFM_1	CAGAAGAGGCGAGGGTGCCG
H2BFM_2	CTCAGAGGAAGCCTCAGCCA
H2BFM_3	GGCCATCTGGATGTCCCGGG
H2BFM_4	TCTGAGACAACCTCGGAGGA
H2BFM_5	CAAAGAGGCCAACTCCACGA
H2BFWT_1	AAGGCACGAAGGCTGTCCTC
H2BFWT_2	GGAAGGTCCAGCCATGGCGG
H2BFWT_3	GTGCCACTCCAACTGCCGCG
H2BFWT_4	TAGATGGCACGACCAGACAA
H2BFWT_5	AGAGGAAGGTCCAGCCATGG
H3F3B_1	AAAGCGCTCCCTCTACCGGC
H3F3B_2	AAGCGCTCCCTCTACCGGCG
H3F3B_3	ACTGCTCGTAAGTCCACCGG
H3F3B_4	GAAGCCTCATCGCTACAGGT
H3F3B_5	TGCGCTCTGAAACCTCAGGT
H3F3C_1	AGCCGTCGGTGCGCTGCAGG
H3F3C_2	AGCCTCCTGCAGCGCACCGA
H3F3C_3	ACGAATCTCTCGAAGCGCCA
H3F3C_4	AGGTTTCAGAGCGCAGCCGT
H3F3C_5	CTGCTCGTAAATCCACCGGT
HAND1_1	AAGAGGATTAAAGGACGCAC
HAND1_2	CTTTGGCAGCAGCAGCACGA
HAND1_3	TGGGTCCTGAGCCTTTCCGC
HAND1_4	TTGGCGGCCGTCTTGGCCGG
HAND1_5	CCTGGCGTCAGGACCATAGG
HAND2_1	ATGGGAGTGGTCCAGGCCGG

HAND2_2	CTGGACCACTCCCATTACGG
HAND2_3	GGTGGCCGATGAGCCAGCCA
HAND2_4	TGGACCACTCCCATTACGGG
HAND2_5	CACCCGGTGGTGCACCACGA
HAT1_1	ACTGCCAATTACCTTACACG
HAT1_2	GGTAGAATATAAGAGTGCAG
HAT1_3	TCAGTTCTCAGTCCAACAGG
HAT1_4	TTTCAAGAAGTTGAGCACCA
HAT1_5	ATTAGACAAATCATTCCACC
HBP1_1	AAGACAAAGATGCACGACGC
HBP1_2	CAATGAGTCATTCATTCCTG
HBP1_3	GAACTGCCTGAAGTTCAGGA
HBP1_4	GAAGAAGAGGACACTGGTGG
HBP1_5	ACTTACCGTAGAGTGTAAGC
HCFC1_1	CGAGACTGGCACCACCAACA
HCFC1_2	GCCATGCGAGACCCACGAGA
HCFC1_3	GCCCTGCGAGACCCACGAGA
HCFC1_4	GCGGGTTGGAGCACACCCTC
HCFC1_5	GCATTAAGGACCTTCCTGCG
HCFC2_1	GAGAAGCTATGTCCAAGCCG
HCFC2_2	GATGATCATCAGCTCCCGGA
HCFC2_3	TATGAGTTGGAGCTACAGCA
HCFC2_4	TCAAGACCAAGACCAAGAGC
HCFC2_5	CATCTCCATTCACAATCATG
HDAC1_1	CCCTCACAAAGCCAATGCTG
HDAC1_2	GAGCAAGATGGCGCAGACGC
HDAC1_3	GCACCATGCAAAGAAGTCCG
HDAC1_4	TAAAGGGCGTTCTCACCCGT
HDAC1_5	CATCCGTCCAGATAACATGT
HDAC10_1	CTCGTCCCAGAGCAGCCGGG
HDAC10_2	GGAGACCCAGGTCCTAGGCA
HDAC10_3	TGAGCGCCTGACCGCAGCCC
HDAC10_4	TGGCAGCCGCCCTCTGGCCA
HDAC10_5	AGGATTTGACTCAGCCATCG
HDAC11_1	GCGAGGCTGGGCCATCAACG
HDAC11_2	TCTGTCTGACAGCATGCTGG
HDAC11_3	TGCTCCAGCGACCGTGGCGG
HDAC11_4	TGTGATGGTAGCAACAGCAA
HDAC11_5	ACAACCGCCACATCTACCCA
HDAC2_1	AAACCGACAACAGACTGATA
HDAC2_2	ATGGCGTACAGTCAAGGAGG
HDAC2_3	GATAAATCCAAGGACAACAG
HDAC2_4	GATGTATCAACCTAGTGCTG
HDAC2_5	CCTCCTCCAAGCATCAGTAA
HDAC3_1	CATGTGCCGCTTCCACTCCG
HDAC3_2	GACGTGGGCAACTTCCACTA
HDAC3_3	GAGCAGAACTCAAAGAGCCC
HDAC3_4	TATGGGTCAATGCCAGGCGA

HDAC3_5	ACCTGGAGCACAATGCACGT
HDAC4_1	CAGGTCCATGGGCACTGCCG
HDAC4_2	GAAGCTGGAGAGGCACCGCC
HDAC4_3	TGCAGTGGTTCAGATTCCGG
HDAC4_4	TTCTGGCCGAGACCAGCCAG
HDAC4_5	CTTACCCGTACCAGTAGCGA
HDAC5_1	AGTAAAGCCATTCTCAGCGA
HDAC5_2	ATGCTGTGCAGAGAAGTCCG
HDAC5_3	TAGGAGGGAGGCGTCCCAGG
HDAC5_4	TCAAAGGAGCCCACACCAGG
HDAC5_5	ACGTTCACCCGTCACTAGTG
HDAC6_1	AATGGAAGAAGACCTAATCG
HDAC6_2	GAAGTGACACTGGAGTCCTG
HDAC6_3	TCTGGACCAGACCACCTCAG
HDAC6_4	TGAGGTGGTCTGGTCCGGAG
HDAC6_5	AGGACACGCAGCGATCTAGG
HDAC7_1	AAGGCCTTACCTCCGAGCCC
HDAC7_2	GCAGAGGGAAGTGCTCTGGG
HDAC7_3	GGTCAAGCAGAAGCTAGCGG
HDAC7_4	TCAGCCGGCCCTCACCTCCG
HDAC7_5	AAGGACTGGGCAAAGTGGAA
HDAC8_1	ACTATGCAGCAGCTATAGGA
HDAC8_2	GAAGGTGGCCATCTCCTCCA
HDAC8_3	GCGGAAGATGGAGGAGCCGG
HDAC8_4	GTGGATTTGGATCTGCACCA
HDAC8_5	ATAGTCAAATATCCCTTCAG
HDAC9_1	ACGGACAACAGGGTCCACCA
HDAC9_2	ATTGAGCTGGGATGGCACTG
HDAC9_3	GCAACAAGAACTCCTAGAAA
HDAC9_4	TGAGCCGCCATCCCAAGCTC
HDAC9_5	AACAGCATGAGAACTTGACA
HDGF_1	AAGAGGAAAGGGTTCAGCGA
HDGF_2	AGCGTTGAAGAGGAGAGCAG
HDGF_3	GCAGAAGGAGTACAAATGCG
HDGF_4	GGGCCTCTCAACCTCCAAGG
HDGF_5	CGAGAACAACCCTACTGTCA
HDGFL1_1	AAGAGGCGCGGCTTCAGCGC
HDGFL1_2	CAAGAGGCGCGGCTTCAGCG
HDGFL1_3	GACAAGCCGACCCACGCTGG
HDGFL1_4	GGAGGAAGAAGTCGCGGACG
HDGFL1_5	CGCCCCCAAACGACCCAAGG
HDGFL2_1	GGCTTCGGTTTCCGCCCTCG
HDGFL2_2	GTGTGCCAAAGAAAAAGATG
HDGFRP2_1	ACTGCAGATCGACGACATCG
HDGFRP2_2	GACAAGAGTAGCGACAACAG
HDGFRP2_3	GCCACACGCCTTCAAGCCCG
HDGFRP2_4	GTCGGAGAAGACCAGCGACC
HDGFRP2_5	CAGCGTGTCCCCATCCGAAG

HDGFRP3_1	ATAGATTGATGAACTCCCAG
HDGFRP3_2	GATGCAAGCAGTGAGGAAGA
HDGFRP3_3	GGAAAGTCAAACAAACGGAA
HDGFRP3_4	TAGATTGATGAACTCCCAGA
HDGFRP3_5	AAACGGAAAGGATTTAACGA
HDX_1	GAAACTACCATTTGACACCA
HDX_2	GCAGGGCCTCCTCTTGGAGG
HDX_3	GCTGTATGTAATGAGGTCCG
HDX_4	GTCCCAATACTTCTTAAGGG
HDX_5	ACTAGATGCTGTATGTAATG
HELLS_1	AGGCTCGGAGGCTCCAGCAA
HELLS_2	GATGCAAGTGAAGAGAAGCC
HELLS_3	TCACTGGAGGAGTGATGCGA
HELLS_4	TGAGTGTCCAGGCATGCCAG
HELLS_5	AATGTTATATCATGGAACCC
HELQ_1	AATGTGGTTCCCGCATCCGC
HELQ_2	ATGATTGGTGAAGGAAGCCG
HELQ_3	GGAGTTGCCTATCACCACAG
HELQ_4	TCTTGTTCAGGAATTCACCA
HELQ_5	CTTATCTCTTACCTTCGAGC
HELT_1	CAAGACAGTGCCCATGGCCT
HELT_2	GCTGGGCAAGACAGTGCCCA
HELT_3	GGTGCATTACCTCACCACGG
HELT_4	TCCGGGAGCGAACCCAGCGG
HELT_5	CCTATCAGCTGCACCCTGCG
HES1_1	AACGCAGTGTCACCTTCCAG
HES1_2	ATGGAGTCCGCCGTAAGCGA
HES1_3	GACAGGGCCGCTGTGCGCGA
HES1_4	GAGGAAAGCAAACTGGCCAT
HES1_5	AGCCAGTGTCAACACGACAC
HES2_1	AATGACCGTGCGCTTCCTGC
HES2_2	CCTTGCGACAGCTACCGCGA
HES2_3	GCCTTGCGACAGCTACCGCG
HES2_4	TCTGGTTGATGCGCGCGCGC
HES2_5	AGAGCCTGAGCCAGCTTAAG
HES3_1	GGAGAAACACTACTCGCACC
HES3_2	GGAGCAGGAGCCCAGACGGC
HES3_3	GTGAGCCAGCTCCTTCGGCG
HES3_4	TCGGCGCAGCGACTCGACGC
HES3_5	AGCCTTCAGAACTCCTTGCA
HES4_1	CCATGGCCGCAGACACGCCG
HES4_2	GAAACCGAGCGCCTCGCCGA
HES4_3	GCGGTACTTGCCCAGAACGG
HES4_4	GTTCTGGGCAAGTACCGCGC
HES4_5	AGATGACCGTGAGACACCTG
HES5_1	AGCTACCTGAAGCACAGCAA
HES5_2	CTGCACCAGGACTACAGCGA
HES5_3	GCACCAGCCCAACTCCAAGC

HES5_4	GCTCCACGGCCACAGTGCTG
HES5_5	ACTACAGCGAAGGCTACTCG
HES6_1	AAGCTGCTGCCCTCACGCAG
HES6_2	AGAGCGCGAGCAGCTGCAGG
HES6_3	TGCTGGAGCTGACGGTGCGG
HES6_4	TGGAGAAGAAGCGGCGCGCG
HES6_5	CACCGTCAGCTCCAGCACTT
HES7_1	CTTGAGGGAGCGAAGCCGGG
HES7_2	GATCGAGCTGAGAATAGGGA
HES7_3	GGTCTCCAGAAAGCGGGCGG
HES7_4	TCGCTCCCTCAAGTAGCCCA
HES7_5	CACCCCCGCGCTGTACCCGG
HESX1_1	CGAGCTGAGCGCCTTCCTGA
HESX1_2	GCTAGGGAATGAAATCCCAC
HESX1_3	GGGAATGAAATCCCACTGGG
HESX1_4	TTGGGTGATCCACCACGCTA
HESX1_5	AATTGAGAGAATCTTAGGAC
HEXIM1_1	ACGGCAGCGAGTTTCTGCAG
HEXIM1_2	CCGAGCCGAGATGTTCGCCA
HEXIM1_3	GGTGTCGTCGGATTTGGCGG
HEXIM1_4	TGACAAGAATGGCTCGGCCA
HEXIM1_5	AGAAGTGGAACCGACGCCCG
HEXIM2_1	ACTTACCTTGGCCTCCTCCA
HEXIM2_2	AGAGTCACCAGTGGCCCTGG
HEXIM2_3	GAAGCGGCTGTCGCAGGCGG
HEXIM2_4	GATGAATGACAGGGACCCGG
HEXIM2_5	CCAGAGTCATGACGCTCAGG
HEY1_1	CGAGATCCTGCAGATGACCG
HEY1_2	GCTGGACGAGACCATCGAGG
HEY1_3	GCTGGGCTCGGCACATCCGG
HEY1_4	TCAACAACTACGCTTCCCAG
HEY1_5	ACTCCGATAGTCCATAGCAA
HEY2_1	AAGATGCTTCAGGCAACAGG
HEY2_2	CATGGACGAGACCATCGACG
HEY2_3	GAAGCTGTGTGCAGCCGCGG
HEY2_4	GGAGCGAGAACAATTACTCG
HEY2_5	AGTGCCTAACAGAAGTTGCG
HEYL_1	AAGCAGGAGAGGAGACACCG
HEYL_2	AAGGAGCCGAGCGGCTCCGA
HEYL_3	GTCCGACGGACCCATCGACG
HEYL_4	TTCCAAGCTGGAGAAAGCCG
HEYL_5	AGAGAAGGCGAATCCGGACG
HFM1_1	AATTGCCTATAATGGAGCAG
HFM1_2	ATTGCAGAATGGCTTTCAGA
HFM1_3	GATACAGCTACAAATCGCAT
HFM1_4	TAATGGAAGTACCATTGCCA
HFM1_5	AAATATCTGACAATATACAT
HHEX_1	AGAATCGACGCGCTAAATGG

HHEX_2	ATCGAGGACATCCTGGGCCG
HHEX_3	GAAGGCTGGATGGATCGGCG
HHEX_4	TCGCAGGCAAACCTCTACTC
HHEX_5	AATATCTCTCTCCGCCCGAG
HIC1_1	AGGCTGCCAGGGTAGCGCGG
HIC1_2	GCAGGCCTGGATGACCGGCG
HIC1_3	GCGGCCGAGGTGCTGAGCCA
HIC1_4	TCGGCCGTGAACTTGGCCAG
HIC1_5	AAGAAACGCCTCAAGCGCCA
HIC2_1	AGTGCGCAGAGCGGGAGCGA
HIC2_2	GAGTGCCGGAGGCTCTTCCG
HIC2_3	GCACGTGGAGACTCACACGG
HIC2_4	GCTGAACCAGCAGAGGACCA
HIC2_5	ATCTTGGACTTCATCTACAC
HIF1A_1	AAGTGTACCCTAACTAGCCG
HIF1A_2	ACAGTAACCAACCTCAGTGT
HIF1A_3	ACTAAAGGACAAGTCACCAC
HIF1A_4	GATGGTAAGCCTCATCACAG
HIF1A_5	GGGTCAGCACTACTTCGAAG
HIF1AN_1	AACAGGGAAAGCGTGGCTGG
HIF1AN_2	AGGCACTCGAACTGATCCGG
HIF1AN_3	ATTTCCAGAACTTTAAGCCG
HIF1AN_4	CAATAAGCTCCTCTGCCCGG
HIF1AN_5	AACTGGATTAATAAGCAACA
HIF3A_1	AGGGAACTCACTCTGCTGGG
HIF3A_2	CACACGCTGCCCTTCGCCCG
HIF3A_3	CGACTGGCGAGCCATGGCGC
HIF3A_4	GAGAATGTCAGCAAACACCT
HIF3A_5	ACAGACGATACTCTCCGACT
HINFP_1	ACCACACCAAGCTGAAACAG
HINFP_2	AGTCGGCAAGGACAACCCGG
HINFP_3	GAAAGCGCATGTGGTTGCGG
HINFP_4	GATCCAATGAGGTCTGGCGA
HINFP_5	CACACCAAGCTGAAACAGTG
HIRA_1	CACCGCCTGAATGATTCCGG
HIRA_2	CATGTGTGAACTGTGTGCGG
HIRA_3	TATGTCTCCAGTCCTCCAGG
HIRA_4	TCAGTTGATATTCACCCTGA
HIRA_5	ATGTCTGAAACGGCCGCTGG
HIRIP3_1	CAGGGCCCGACACTTCCCTA
HIRIP3_2	GACAGGACCCAGCTTAAGGG
HIRIP3_3	GCGCTTCCAGTTCTGCCCGG
HIRIP3_4	GGTGGATGAAGCCGCTTCCA
HIRIP3_5	GAGGTTGAGTGGAAGCAGCG
HIST1H1C_1	GGTGATGAGCTCTGACACCG
HIST1H1C_2	GGTGTCAGAGCTCATCACCA
HIST1H1C_3	TTTAGAGGCGGCCACAGCCT
HIST1H1C_4	TTTCTCCACATCATAGCCGG

HIST1H1C_5	CAAGCCCAAGGTTAAAAAGG
HIST1H1D_1	AAGAAGGCAGGCGCAACTGC
HIST1H1D_2	AGGCGGCCAAGCCTAAGTCG
HIST1H1D_3	AGTATCTGAGCTTATCACCA
HIST1H1D_4	TGAGCTTATCACCAAGGCAG
HIST1H1D_5	AGGCTGGCGCAGCCAAGCCT
HIST1H1T_1	AGAAAGGCTAAAGGAGCCAA
HIST1H1T_2	GAAGAGCCCAGTGAAGGCAA
HIST1H1T_3	GGAATCCTGGTGCAAACCAG
HIST1H1T_4	TGTGTCCAAGTTGATCACCG
HIST1H1T_5	CTCAAGAGCTTAGTGAACAA
HIST1H2BA_1	AAAGCGCAAGAGGACCCGTA
HIST1H2BA_2	AGGTGCTACCATTTCCAAGA
HIST1H2BA_3	GCACCTTTAGATGACACCTC
HIST1H2BA_4	GTGTGTGGTAGCTATGCCGG
HIST1H2BA_5	CATCTACAAAGTGCTAAAGC
HIST1H2BJ_1	ACTCACTGTTTACTTAGCGC
HIST1H2BJ_2	AAACGAATTCATGATGCCCA
HIST1H2BJ_3	ACGAAATGCCGGTGTCAGGG
HIST1H2BJ_4	AGATGGAATAGCTCTCCTTG
HIST1H2BJ_5	GAAAAAGGGCTCCAAGAAGG
HIST1H2BL_1	CTTGAGAATTTACTTGGAGC
HIST1H2BL_2	CATGGCCTTAGAAGAGATGC
HIST1H2BL_3	CTTCGAGCGCATCGCAAGCG
HIST1H2BL_4	GAAGAAGGGCTCCAAGAAGG
HIST1H2BL_5	GGTGACCAAGGCCCAGAAGA
HIST2H2AB_1	AGAGCTCAACAAGTTACTCG
HIST2H2AB_2	CGGCTAGTTGCAGATGGCGA
HIST2H2AB_3	GACCGCGGAAATTCTGGAGC
HIST2H2AB_4	TCTTGGGCAACAGGACAGCC
HIST2H2AB_5	AGCGCGACTTGGCCTTAGCG
HIST2H2BF_1	ACGAAAGTGCAGAAGAAGGA
HIST2H2BF_2	TGTTACGAAAGTGCAGAAGA
HIST2H2BF_3	CGACATCTTCGAGCGCATCG
HIST2H2BF_4	CTACTCCGTTTACGTGTACA
HIST2H2BF_5	TCCGCTCCTGCTCCCAAGAA
HIST2H3D_1	CCGCTACCGGCCCGGCACCG
HIST2H3D_2	CCGTAGACTTCTGGTAGCGC
HIST2H3D_3	CCTTGCCGCCGGTCGACTTG
HIST2H3D_4	GGTAGCCAGCTGCTTCCTCG
HIST2H3D_5	CCGGCGCTACCAGAAGTCTA
HIST3H2A_1	AGTCAAGTACTCGAGCACCG
HIST3H2A_2	CGGGCGGCCTCACTTGCCCT
HIST3H2A_3	GCACCGCGGCCAGATAGACC
HIST3H2A_4	TATTCGGAGCGCGTGGGCGC
HIST3H2A_5	CACCGCGGCCAGATAGACCG
HIVEP1_1	AAAGATGTACTAGTGAGCTG
HIVEP1_2	ATAGGAAGACACTCTCTCCG

HIVEP1_3	GATGAGAGACAGCATGACCT
HIVEP1_4	GCAGGACCGAGTCTCCTCAG
HIVEP1_5	GGAAAGCAAGACTCTCACGT
HIVEP2_1	GATCAACGAAGAATGTCCCA
HIVEP2_2	GGGAGTCTCAATGACATCGG
HIVEP2_3	TAAGGACTCGGACGATGCGG
HIVEP2_4	TATGTCAGAGGACGTGGCCG
HIVEP2_5	CCCTGGATAGAATACATCGT
HIVEP3_1	CGGGCGGGAAGAGAACCCGT
HIVEP3_2	GACTAAGCACATGAAGTCGA
HIVEP3_3	GAGCAAAGAGACATACACCA
HIVEP3_4	TATGTGCGAGGCCGCGGCCG
HIVEP3_5	AACACGGACAGGCACAACCA
HJURP_1	AAAGGCAGCACTTACTGCTA
HJURP_2	AAGGAAATACTTGACCCAAG
HJURP_3	GAGCCATCTGTCCTGTCCGC
HJURP_4	GCTTCCAGAGGCGCATGCAG
HJURP_5	ACCATCAGTGACCTGTACGC
HKR1_1	AATGTGGGCGAGGCTTTACG
HKR1_2	GTCCAAGGAAGACAACACAG
HKR1_3	TAGGGTCCAGCCCTGAACGG
HKR1_4	TCATTGCTCAGCTGGAGCGA
HKR1_5	CACACCTGGTCAGACACAAG
HLCS_1	AAGTAAAGAGGAGTTAAGGC
HLCS_2	ATCGCGGCCCGGCAGACCGA
HLCS_3	GAAGAACCCAAACAAAGGAG
HLCS_4	TCAGGGATCCTCTTATGCAG
HLCS_5	ACAGTGCTCTCAGAGACCCG
HLF_1	AGTCAGCCACCTCCTGGCGG
HLF_2	GTAGGGAGGCGGGATAAAGG
HLF_3	TCGCAAACGCAAGTTCTCTG
HLF_4	TGATGAGAGTAACAGCCCGA
HLF_5	GACCTGAGCACGCCGTAGGG
HLTF_1	AATACAGCGTCCTGTCACAA
HLTF_2	GTTGGACTACGCTATTACAC
HLTF_3	TACAGTCTGAAACTAAAGGC
HLTF_4	TAGGAGCTTGTGCAGTGGAG
HLTF_5	CATTTATTGATAGAGAATGG
HLX_1	CAAGTGCGCGGTGAGGGCGG
HLX_2	GCCGGACCGAAAGCAGCTGG
HLX_3	GCGAAGAACTGGCCGGCCGA
HLX_4	TGCGCCAGCAGCCTTGGCGG
HLX_5	ATCTCTAGATCCCATTAACG
HMBOX1_1	AATGCAGTTATACAGAAGCC
HMBOX1_2	AGTTGGTGACATCTTTCCAT
HMBOX1_3	TATGCTTAGTTCCTTTCCAG
HMBOX1_4	TCAAACAGTGATGATGTCGA
HMBOX1_5	ATAACTGTTGCTAGGTGACG

HMG20A_1	AAAGGCAAATCTCATAGGCA
HMG20A_2	ATTCAGGTTAAATCACCCAG
HMG20A_3	TAAAGCAGCTGCTTACCCAG
HMG20A_4	TAGCAACGAAGTAAACGAGG
HMG20A_5	ATGGAGTAAACTGCCTCCTG
HMG20B_1	AAGGCTCCGGGCCAGCATGG
HMG20B_2	AGAGAAGCAGCAGTACATGA
HMG20B_3	GAAGAAACGCGGCTGGCCCA
HMG20B_4	GGATCAGTGCACTCACGCGG
HMG20B_5	AAGAAACGCGGCTGGCCCAA
HMGA1_1	ACTGCCCACCTCACAGGAGA
HMGA1_2	GCCCACCTCACAGGAGAAGG
HMGA1_3	AAAAGGACGGCACTGAGAAG
HMGA1_4	AGCGCTGGTAGGGAGTCAGG
HMGA1_5	CCAACACCTAAGAGACCTCG
HMGA2_1	CAAGAGGCAGACCTAGGAAA
HMGA2_2	GCAGCCGTCCACTTCAGCCC
HMGA2_3	GCCCTCTCCTAAGAGACCCA
HMGA2_4	TGTTCAGAAGAAGCCTGCTC
HMGA2_5	TCCCTCTAAAGCAGCTCAAA
HMGB1_1	AACCTATATCCCTCCCAAAG
HMGB1_2	GAAGTGCTCAGAGAGGTGGA
HMGB1_3	GGCGATACTCAGAGCAGAAG
HMGB1_4	GGTGATGTTGCGAAGAAACT
HMGB1_5	AGATACTCACGGAGGCCTCT
HMGB2_1	ACAGAAAGCAGCTAAGCTAA
HMGB2_2	GAACGAACCAGAAGATGAGG
HMGB2_3	GAAGAACGAACCAGAAGATG
HMGB2_4	TCCACAGACCATGTCTGCAA
HMGB2_5	CCTCCCAAAGGTGATAAGAA
HMGB3_1	AAAATTCCGCAAAATTGACA
HMGB3_2	AACAGGAAGAATCCAGACCT
HMGB3_3	CTGCACAAAGAAGGCATAAG
HMGB3_4	GAAGTGCTCTGAGAGGTGGA
HMGB3_5	AATAGAGATGCCGGGGTTTG
HMGN1_1	GCCGCCGCTCACCTTCCTCT
HMGN1_2	AAAGGGAAAACAGGCCGAAG
HMGN1_3	CCGCAGGTCAGCTCCGCCGA
HMGN1_4	CTCCAAGCCCAAGAGGAGAT
HMGN1_5	TCAGAAACCTCCTGCAAAAG
HMGN2_1	ACGTACCTTTCTCTTGGGCA
HMGN2_2	GCGCCACGTACCTTTCTCTT
HMGN2_3	GTTAGCACTTACCTTTGCAG
HMGN2_4	ACTTACAGCAGACAACCTCG
HMGN2_5	TGCTAAGGGAGATAAAGCAA
HMGN3_1	AATCTGGCAGACCGTCTTGT
HMGN3_2	CCTGGAGCAAAGATTAGCAG
HMGN3_3	CTAGTCTCCAGAGAATACAG

HMGN3_4	TAGTCTCCAGAGAATACAGA
HMGN3_5	ACGGTCTGCCAGATTGTCAG
HMGN4_1	AACCGAGCTGATCTCCTCTG
HMGN4_2	AGCCACAGAGGAGATCAGCT
HMGN4_3	CCAGAAAGCGGAAGGCACTG
HMGN4_4	TCCCAGAAAGCGGAAGGCAC
HMGN4_5	AAGGCAAAAGGAGATGCTAA
HMGN5_1	AGCCAAAGAGAAGATCTGCC
HMGN5_2	AGGTCAAGGTGATATGAGGC
HMGN5_3	TCAAGGTGATATGAGGCAGG
HMGN5_4	AGATCAAAACGAAGAGAAAG
HMGN5_5	ATTACTTACAGCAGACAACC
HMGXB3_1	ACTGCTGTACCACAATTCCC
HMGXB3_2	AGACCACTACATATACCCGC
HMGXB3_3	CTAGGAGCACATCCTGGCTG
HMGXB3_4	GAGTTGGAGGCTTCTCAGCG
HMGXB3_5	ACCACTACATATACCCGCCG
HMGXB4_1	AAAGATCAAGGTTAGCACTG
HMGXB4_2	CACGGAGTCATCATAAGCCA
HMGXB4_3	GAGGACATAGGACTTGCAGC
HMGXB4_4	TGAGGCTGGTAACAGCATGG
HMGXB4_5	ATAGTGAACTTTACTTCTTG
HMX1_1	CAGAAAGGGCACGGAGGCGG
HMX1_2	CGAGGAGATGGGCCGTGCGG
HMX1_3	GCCTCCGCAGCCCAGAGCGA
HMX1_4	GCGGCGGCGACAGTTGCTCG
HMX1_5	CGGCGGCGACAGTTGCTCGC
HMX2_1	AAAGAGAGGCTCCTGCCCGC
HMX2_2	ATCCAGTCCATCCTGGGCGG
HMX2_3	GAAAGAGAGGCTCCTGCCCG
HMX2_4	GGAGCCGGACGACGGCTGGA
HMX2_5	AGGCAAGGAAAAGGGATGGG
HMX3_1	GCGCCGGGATCTCAAAGCGA
HMX3_2	GGCAGGGCAAACCTCTGCGC
HMX3_3	TCTCAGCAAGGCCTTCTCCG
HMX3_4	TCTGGAGGAGAGCGACTCCG
HMX3_5	GCAGTGGCTCCGGAGAGTCG
HN1_1	ACGAGGCTGGACTTGCCGCC
HN1_2	GAGGCTGGACTTGCCGCCAG
HN1_3	GTCAGAGCTAACCCAAGACG
HN1_4	TTAGGTGCCAAGTCTAGTGG
HN1_5	AACCAACAGAACAACCTGTG
HNF1A_1	CTGCAGAAGGGTCTCCACCA
HNF1A_2	GAGGCAGAAGAACCCTAGCA
HNF1A_3	GCAGCACAACATCCCACAGC
HNF1A_4	GGGCTCCAACCTCGTCACGG
HNF1A_5	AGACACGCACCTCCGTGACG
HNF1B_1	AGGGCAGAATGTTTGCAGCG

HNF1B_2	GAGGTGCGACTGGTTCAGGC
HNF1B_3	GAGTTCAGTCAACAGAGCCA
HNF1B_4	GCTGCAGGCGCTCAACACCG
HNF1B_5	AGCAACACAACATCCCCCAG
HNF4A_1	ATTTAGCCGGCAGTGCGTGG
HNF4A_2	CAATGCGCTCCTGCAGGCGG
HNF4A_3	GCAATGACTACATTGTCCCT
HNF4A_4	GGACCCAGCCTACACCACCC
HNF4A_5	CAGCTCGTCAAGGATGCGTA
HNF4G_1	ACTGTTGAGAGCTCACGCAG
HNF4G_2	GATCGGCAGTATGACTCCCG
HNF4G_3	TTATGTCAGTGCTTGACCCA
HNF4G_4	TTTATGTCAGTGCTTGACCC
HNF4G_5	ACAGTTGACACCGTTGTCTG
HNRNPA0_1	AAGGAGGATATCTACTCCGG
HNRNPA0_2	AGGTCACGAAGCCAAAGCAA
HNRNPA0_3	GACCAGAACGGCCTTTCCAA
HNRNPA0_4	TATGGGCCCATGAAGAGCGG
HNRNPA0_5	CTTCGTGACCTACTCCAATG
HNRNPA1L2_	CATCCAGCCAAAGAGGTCGA
HNRNPA1L2_	GAATACAACGCCACACAAGG
HNRNPA1L2_	GGGAACGCTCACAGACTGTG
HNRNPA1L2_	TCAGAAATACCATACTGTGA
HNRNPA1L2_	AGCCCTGCCAAAGCAAGAGA
HNRNPA2B1	CCAGGAAGTAACTTTAGAGG
HNRNPA2B1	CTAACAGATGGATATGGCAG
HNRNPA2B1	GAAGTTCAGAGTTCTAGGAG
HNRNPA2B1	TGAGGAACTACTACGAACAA
HNRNPA2B1	ACTCTCCCATCAATTGAATG
HNRNPAB_1	AGAAGAAGAACCCGTGAAGA
HNRNPAB_2	AGCGGGCGAGGAGCAGCCCA
HNRNPAB_3	CGGGTCCTTCTTCATAGCCA
HNRNPAB_4	GCCGTGGAAACCGCAACCGA
HNRNPAB_5	CCAACACTGGACGGTCAAGA
HNRNPD_1	AACAAGACCAATAAGAGGCG
HNRNPD_2	ACGACACTCTGAAGCAGCGA
HNRNPD_3	GCAGGAAGAGCTCGTGGAAG
HNRNPD_4	GTCGGAGGAGCAGTTCGGCG
HNRNPD_5	ACAAGACCAATAAGAGGCGT
HNRNPK_1	AAATGAGCCTACCTCTTCCA
HNRNPK_2	AGACCTGGAGACCGTTACGA
HNRNPK_3	ATCCCACTGGGCGTCCGCGA
HNRNPK_4	GTTTCAGTCCCAGACAGCAG
HNRNPK_5	ATGATGTTTGATGACCGTCG
HOMEZ_1	ACCAGCTAATACCACAGCGG
HOMEZ_2	ATGGCCCAGCGCCTCCGCTG
HOMEZ_3	GAACCCACTTACCTCTCCTG
HOMEZ_4	GCTGCCCAGTACCTCTCCAA

HOMEZ_5	AGCAAGCCTACCCAGACGAA
HOPX_1	CCTGGAGTACAACTTCAACA
HOPX_2	GCGCGCAGGGACCATGTCGG
HOPX_3	GCTGTGCCTCATCGCGGCCG
HOPX_4	GGCCGAGGCAGGCCTTTCCG
HOPX_5	ATGTCGGCGGAGACCGCGAG
HOXA1_1	AGGAAACGACGAGAAGGCCG
HOXA1_2	ATGCAGCGATCTCCACCCTG
HOXA1_3	CAGGAAGCAGACGTAAGTGG
HOXA1_4	GGAGTTATTATACGTAGCCA
HOXA1_5	ACCACAACTTGAGTGGGAGT
HOXA10_1	GAAGCACCAGACACTGGAGC
HOXA10_2	GAAGGGAGCCAGTTCGGCGG
HOXA10_3	GTTGTGGGATAATTTGGCGA
HOXA10_4	TACATGCCAGGCAATTCCAA
HOXA10_5	AGATCGAAACCGCGCCCCGG
HOXA11_1	AGTGGGCCAGATTGCCGCGG
HOXA11_2	GAAATTGGACGAGACTGCGG
HOXA11_3	GGCCCACTGCTACTCCGCGG
HOXA11_4	TCTGGCCCACTGCTACTCCG
HOXA11_5	AGAAATTGGACGAGACTGCG
HOXA13_1	GAAGCGCGTGCCTTATACCA
HOXA13_2	GAAGGCGAACTCCTTAGCGC
HOXA13_3	GTCGTAGAGAAACATGACGG
HOXA13_4	TCATTACTAAGGACAAACGG
HOXA13_5	ATGTTTCTCTACGACAACGG
HOXA2_1	AGTCACCCTCGCCACGGCGC
HOXA2_2	CCCGGCAGTCACCCTCGCCA
HOXA2_3	GAAATCGCCGATGGCAGCGG
HOXA2_4	GAATCCCTGGAAATCGCCGA
HOXA2_5	AGGGAAATGTAAAAGCCTTG
HOXA3_1	CTGGCGACAAGAGCCCGCCG
HOXA3_2	GAAGCGCTACACGGCGGCAG
HOXA3_3	GGCATTATAAGCGAACCCGT
HOXA3_4	GGGTCGGTGGTACTCGCCGT
HOXA3_5	CAAGGCACACGAACTGAGTG
HOXA4_1	AAGAAGATCCATGTCAGCGC
HOXA4_2	AGAGGCCGAGGCCGAATTGG
HOXA4_3	CAAGAGCAGCGGGCACGCGG
HOXA4_4	TGGGCCCTTGGCTTGCGCCG
HOXA4_5	CGCGCTGTACCCCGCGCATG
HOXA5_1	AACGCTCAGATACTCAGGGA
HOXA5_2	AACTCCCTAAGCAACTCCAG
HOXA5_3	AATGGCATGGATCTCAGCGT
HOXA5_4	GACAACATAGGCGGCCCGGA
HOXA5_5	CGGTCGCTATCCAAATGGCC
HOXA6_1	ATCAATTCCACGCAGCCCAG
HOXA6_2	CAAGAAGGAGTCCTGGCCGC

HOXA6_3	GCAGCGGATGAACTCCTGCG
HOXA6_4	GGCAAAGCACTCCATGACGA
HOXA6_5	AGGCTGGCTATGACGCGCTG
HOXA7_1	AACGGTCGAGGCGAAGGCGC
HOXA7_2	ACTGCCGCCGCAGCTCCCGA
HOXA7_3	CGTGGGCGATTTCAATGCGG
HOXA7_4	TGTCGCAGGCGCCTTTGGCG
HOXA7_5	CTCACAGAGAAGCGGCTACG
HOXA9_1	ATGGTGGTGGTACACCGCAG
HOXA9_2	GCGTTGGCCGCTATGCGCCG
HOXA9_3	GCTGGAGGGCAAGCCCGCGA
HOXA9_4	GTGGAACCCAGTGCACGCGG
HOXA9_5	ACCACAAGCATAGTCAGTCA
HOXB1_1	ACTGAGAAGGCCCGTAGCTG
HOXB1_2	GCAAGCGAGGGCCGCTACGG
HOXB1_3	TGTTTCATTGAGCTCCAGGG
HOXB1_4	TTGTGGTGAAGTTGGTGCGG
HOXB1_5	GAGTACCCACTCTGTAACCG
HOXB13_1	AGAGAAGAAGGTTCTCGCCA
HOXB13_2	GATGGAGCCAAGGATATCGA
HOXB13_3	GCAATGCCACCCATGCCCTG
HOXB13_4	GTCAGGAGGGTGCTGCCCGC
HOXB13_5	ACGTCCAGGTAACTGGCCAT
HOXB2_1	AAGCGAACGGCTAAAGGCCG
HOXB2_2	AGGGTCGCAGATGTCCTCCA
HOXB2_3	TTGAAATGTCTCCAAGACAG
HOXB2_4	TTTCATCCAAGGGAACTCGG
HOXB2_5	ACCGGCGCATGAAGCACAAG
HOXB3_1	ATGGCGCCCAGCCAGCACCA
HOXB3_2	ATTCTGGTGGGCTTTACCGA
HOXB3_3	GCAGCGAGCAAGCTGAGCGC
HOXB3_4	GTAGGTGCGGCACTGGGCGG
HOXB3_5	AGCTCAACGGCAGCTGCATG
HOXB4_1	AACACCAAGATCCGCTCGGG
HOXB4_2	AGAGAGCAGCTTCCAGCCGG
HOXB4_3	GTGCGGCAGGCTCAGCCGGA
HOXB4_4	TCGCCCGGGTACTACGCCGG
HOXB4_5	AAACCCCAATTACGCCGGCG
HOXB5_1	AAAGTGGCTGGAGGAGGCCG
HOXB5_2	AGGAAGCTTCACATCAGCCA
HOXB5_3	GAAGCCGCTTGCCTGAAGCG
HOXB5_4	GCTGCGCCCGAGCTAGGCTG
HOXB5_5	AATTTAGCAACTGATAGTCC
HOXB6_1	AATAGAGCGGTAGCTGGCCC
HOXB6_2	AGAGCAGAAGTGCTCCACTC
HOXB6_3	CAGGAAGGACTCCTGCCCGC
HOXB6_4	GCGGAAGTCGGACTGCGCGC
HOXB6_5	CTTCTACCGCGAGAAAGAGT

HOXB7_1	AGAACAAGACCGCGGGCCCG
HOXB7_2	CTTTGAGCAGAACCTCTCCG
HOXB7_3	GCAGGAACTGACCGCAAACG
HOXB7_4	GTGTCCCGGCGACTCCGCCA
HOXB7_5	GGTAGCGAAAACCGAACTTG
HOXB8_1	AATCGAGGTATCGCACGCCC
HOXB8_2	CCAGCAGAACCCGTGCGCCG
HOXB8_3	GCTGCTGGGACCGTACACCA
HOXB8_4	GCTGGGACCGTACACCACGG
HOXB8_5	AGCCGCAGTCATAATAATTG
HOXB9_1	AGCGTGCAGCCAGTTGGCGG
HOXB9_2	CAGCTTCCAGCCCAAAGCGC
HOXB9_3	GGAACTCCAGGTGCTCCGCG
HOXB9_4	TTGGGCTGGAAGCTGCACGA
HOXB9_5	ATGTAAGGGTGGTAGACGGA
HOXC10_1	AGTTCGGAGTTACATTGCGA
HOXC10_2	CAGCCCGAGCTACTCCGCGC
HOXC10_3	GAGCGCTATAGCCGGAGCGC
HOXC10_4	GGGAGTAGAGAGCTGCCTCG
HOXC10_5	GATTCCAGATGTTCGGCGCG
HOXC11_1	AGGAGGAGTAGCTGTTCCGA
HOXC11_2	GGGATAGGAGATCTGACGAG
HOXC11_3	TAGGAGACCTCCCGGACCGG
HOXC11_4	TGAGGAGTAGAAGCCGGCGG
HOXC11_5	AAAAACGAAGGCTCCTACGG
HOXC12_1	AACGAGGGCAACAAGGGCGC
HOXC12_2	ACGGCGATACCAAGCTGCCG
HOXC12_3	AGCGGTCTGAGAGTTCCCTC
HOXC12_4	AGTTCATCACACGCCAGCGC
HOXC12_5	AGCACACGTTGTCGCGGCGT
HOXC13_1	AGTGCTGGTAGCCTTCGACG
HOXC13_2	CGTCTATGAGGACAGCGCGG
HOXC13_3	GAAACGCGTGCCCTACACTA
HOXC13_4	GAGGTGAGCAGCTACCGGCG
HOXC13_5	CCGGGTATTTATCGCCCGGG
HOXC4_1	ACAACCGCTACCTGACCCGA
HOXC4_2	ATAGCTGCACCAGTCTCCAG
HOXC4_3	GTGGTCTTCAGAAGTACCCG
HOXC4_4	TCAGGGTAGCTAGGGCGCGG
HOXC4_5	ACAGTCCGGAATATTACGGC
HOXC5_1	CCACGGGTAAATCTGTGGCG
HOXC5_2	GAAGCGGCTCCTCTGAACCC
HOXC5_3	GAGGAGCGAGCTAAGAGCAG
HOXC5_4	GAGTTGGAAGGCGCAGGCGG
HOXC5_5	AACTTGTGGGAACTATGGAT
HOXC6_1	CTTATCCTGCCACCTCGCCG
HOXC6_2	TACGGAGCGGACCGGAGGCG
HOXC6_3	TCGGCTACGGAGCGGACCGG

HOXC6_4	TTATCCTGCCACCTCGCCGG
HOXC6_5	ATGCCTCACTGGATCATAGG
HOXC8_1	ATTGAAGTCTCTCATGCCCT
HOXC8_2	GCTACTGTTAGTGTTGGCGG
HOXC8_3	GGTGGAAGAAGTCTTGAACG
HOXC8_4	GTGGACGGCAAACTTACAGC
HOXC8_5	AACCGGCAGTCGTAATAGGC
HOXC9_1	ACAGTCCGGCACCAAACCGC
HOXC9_2	CCCGGGCCACCTCATACCGA
HOXC9_3	GCAGACACTGCCCTCGCCCG
HOXC9_4	GGCGTGAATCCAGTTGGCCA
HOXC9_5	AGCTACCCGGACTACATGTA
HOXD1_1	GAAATTCGTGCGGATCGCGC
HOXD1_2	GAAGGCGGGCTGCAGAGCCA
HOXD1_3	GTACGCGCAGTGCACCCTGG
HOXD1_4	TCTGCAGCCCGCCTTCCCTC
HOXD1_5	ACCTCGGCCGTCTTCTCGGG
HOXD10_1	CAACAAACTCATTTCGGCCG
HOXD10_2	GTTGAGCTGGAGCATGACAG
HOXD10_3	TGGACACGGAGACCTCAGCC
HOXD10_4	TTACACTAAGCACCAAACGC
HOXD10_5	AAGATGAACGAGCCCGTGAG
HOXD11_1	AAAGTCATCTGGCAGGACGA
HOXD11_2	CTACGCTCCCTACTACGCGG
HOXD11_3	GAAGTTGGAGGCGGCGCCCG
HOXD11_4	TGAAGAGCACGTCCGGCCGG
HOXD11_5	CTTGCTAGCGAAGTCAGACG
HOXD12_1	GAACATGACAGTGCAGGCGG
HOXD12_2	GCGTATGAAGAAGAAGCGCG
HOXD12_3	GCTGTGTTGCAGGCCTGCCG
HOXD12_4	GGGCTGCGAGAAGCCGCCGA
HOXD12_5	ACTGTCATGTTCAAGTTGAG
HOXD13_1	AAGGAGGTATCCTTCTACCA
HOXD13_2	CAGGCGCGGAGAGAAAGCCG
HOXD13_3	TCATTAACAAGGACAAGCGG
HOXD13_4	TGTGCGTCTACCGAAGAGGG
HOXD13_5	CAGCAGCGTACCGGCCAACG
HOXD3_1	ACAGAAGCGCTACGCAGCGC
HOXD3_2	TCACCCACCAATCCTGGAGG
HOXD3_3	TCTTCACCCACCAATCCTGG
HOXD3_4	TTGGCGAAAGCAGGCGGCGA
HOXD3_5	CTATGGCTACAGCAAAACTA
HOXD4_1	CCAGTTCTAGGACTTGCTGC
HOXD4_2	GGAACCCAAGCGGTCCCGAA
HOXD4_3	GGTCCCGAACGGCCTACACC
HOXD4_4	TAGGCCGTTCGGGACCGCTT
HOXD4_5	CTGCTTGAGTGCCGTCCCGG
HOXD8_1	GTACTCCAAGTACAAGGCGG

HOXD8_2	GTGACAGGCAATCCCGCCGC
HOXD8_3	TCTCGGTGAGGGCTAGGGCG
HOXD8_4	TGACAGGCAATCCCGCCGCA
HOXD8_5	AAGTGACAGTCGTAGTAAGT
HOXD9_1	ACACGAGGCGAACTCGGCGG
HOXD9_2	ACTACACGAGGCGAACTCGG
HOXD9_3	AGCGTGGATCCAGTTCGCGG
HOXD9_4	TCCTGGCCACCTCGTAGCGC
HOXD9_5	CAAACGGACTGAGTGCTCCG
HP1BP3_1	AGTGAGGCAGAGCAGCCAAA
HP1BP3_2	GATGGATGCAATCTTAACTG
HP1BP3_3	TAAGTGGGAGTGCCTTAGGA
HP1BP3_4	TCAGAATAGGAGCTCTGCAG
HP1BP3_5	AAAGGCGAGTAAAGGCCAGT
HPRT1_1	GAAAGGGTGTTTATTCCTCA
HPRT1_2	GATGTGATGAAGGAGATGGG
HPRT1_3	GTTATGGCGACCCGCAGCCC
HPRT1_4	TCACCACGACGCCAGGGCTG
HPRT1_5	AATAAATCAAGGTCATAACC
HR_1	GAGGAAGTGAACAAGGCCTC
HR_2	GCAGCAAAGAGGGACTGCGC
HR_3	GCCACTCTCAGCAAGTGCGG
HR_4	GGTCCTGTTCACCTTCCCGC
HR_5	GAGCAGTTTGAATGTCCACG
HSF1_1	CGACCAGGGCCAGTTTGCCA
HSF1_2	GCTCCAGCAGATGAGCGCGT
HSF1_3	GTGGTCCACATCGAGCAGGG
HSF1_4	TATGTCTTCACTCTTCAGGG
HSF1_5	ACAGAAAGTCGTCAACAAGG
HSF2_1	ATTCTGACACCTCCTTCCAA
HSF2_2	CAAGCGTCCACAGCTTGCTG
HSF2_3	GGACTGAAGGTTTAAAGCCA
HSF2_4	TATGCACCTGTCATTCAGAG
HSF2_5	AAAGTAGTACATATCGACTC
HSF4_1	CCAGCGCCCATAGCTTGCCG
HSF4_2	CGACCAGAGCCGTTTCGCCA
HSF4_3	GGGAGTGCAGGAGAGCACCG
HSF4_4	GTGGTGAGCATCGAGCAGGG
HSF4_5	CAGCACCCGAGCTTCGTGCG
HSF5_1	AGCACAATAATCCTTACCAG
HSF5_2	GAAGAGCTCGGGCTCGGCCC
HSF5_3	GCAAGCTCATGTCAAACTGA
HSF5_4	TCCGATCTAAACCTTTCAGA
HSF5_5	ACTACTTAGAGCATCCATGT
HSFX1_1	CCTCAAGTTAGGGCTCCCAG
HSFX1_2	TAGGTCTCTGACTGCAACTG
HSFX1_3	TCCACAGTTTCTGTGGAAAG
HSFX1_4	TTCTCTTCATCCTCACGAGG

HSFX1_5	GAGAGGACAGGATCATGGCT
HSFY1_1	ACAGAAGATTCCCTTGTTAG
HSFY1_2	AGAATCGTCTGTCTTAAGCA
HSFY1_3	GTAAGAGTGAAGAGAAGAAT
HSFY1_4	TTCACACAATGGAGACCTAG
HSFY1_5	ACACAATGGAGACCTAGTGG
HSFY2_1	GTAACTTGGACTTTCTAACA
HSFY2_2	TATAATCCAAATTTCAAGCG
HSFY2_3	TTTAAATATGCCTCTAACAA
HSFY2_4	ACCGTAAGTCTGAGTCCCCA
HSFY2_5	GGGAAGGTGTGTTCACACAA
HSPA1A_1	CCGCAAGTTCGGCGACCCGG
HSPA1A_2	CTGCTGCAGGACTTCTTCAA
HSPA1A_3	GACCAAGGCATTCTACCCCG
HSPA1A_4	GCCGTCGTCGATCGTCAGGA
HSPA1A_5	CAAGATGAAGGAGATCGCCG
HSPA1B_1	ACACCGTGTTTGACGCGAAG
HSPA1B_2	AGGATGCGGGTGTGATCGCG
HSPA1B_3	GAGCTACAAGGGGGAGACCA
HSPA1B_4	TCCATCCTGACGATCGACGA
HSPBAP1_1	AGGAAAGGAGTATCTTCAGG
HSPBAP1_2	ATGGACAGGAAAGTACATTG
HSPBAP1_3	GATCACAGGAGTGGTCGCCT
HSPBAP1_4	TGTGTGCAACCACATGGAGG
HSPBAP1_5	AGAAGCCGCCTACCTCAGTG
HTATIP2_1	AAGTCACGCTCATTGGCCGG
HTATIP2_2	GAGGAAGCTCACCTTCGACG
HTATIP2_3	GATAGGCAAGAATCTCGCCC
HTATIP2_4	TACCACCAGAGGGAAAGCTG
HTATIP2_5	CCTTGCCTAGAATCAAGAAG
HTR5A_1	AATGAGGATGCCCACCATGA
HTR5A_2	AGTTGGAGTAGCCAAGCCAC
HTR5A_3	GGTGGAAGGTGCGTACACGG
HTR5A_4	TTGGAGACCAACCACAGCCT
HTR5A_5	AAGGCTGCCAAGTTCCGCGT
HUWE1_1	AAAGCTGTACTCAATGAGCA
HUWE1_2	ACACACATACGACTGGCCCA
HUWE1_3	ACTGAAGAAGACACCTACTG
HUWE1_4	TCGGCTACAACATTTGGCAG
HUWE1_5	AAGCGCTCAAATCGGACCAA
ID1_1	ACAGGCGGCATGCGTTCCTG
ID1_2	AGAATCATGAAAGTCGCCAG
ID1_3	GAACCGCAAGGTGAGCAAGG
ID1_4	GATCAGCGCCCTGACGGCCG
ID1_5	CGGCAAGACAGCGAGCGGTG
ID2_1	AACGGACCTCACGGGACTGA
ID2_2	AGCCTGTCGGACCACAGCCT
ID2_3	GCTGATATCCGTGTTGAGGG

ID2_4	TGAAAGCCTTCAGTCCCGTG
ID2_5	AATAGTGGGATGCGAGTCCA
ID3_1	AAGGCGCTGAGCCCGGTGCG
ID3_2	ACGCAGTCTGGCCATCGCCC
ID3_3	AGGCACTCAGCTTAGCCAGG
ID3_4	TCAGGACACGGCCGAGTCAG
ID3_5	ATGTCGTCCAGCAAGCTCAG
ID4_1	ACTGCTATAGCCGCCTGCGG
ID4_2	AGCGCGCTGTAAGGCGGCCG
ID4_3	GCCCTGCTTGTTCACCGCGC
ID4_4	GCCGAGCACGGCCACAGCCT
ID4_5	ACGACTGCTATAGCCGCCTG
IER2_1	AAAGCCCGCTTGCCGCCCGG
IER2_2	GAAACGGGTGCTCACCGTCG
IER2_3	GGAGCCAATGGACACGCAGG
IER2_4	GGAGCTCTACCTCTCGGCCA
IER2_5	ACAGCGCATCATGACCCTGT
IFI16_1	AGGATGCAGATACTGAAGGA
IFI16_2	CCGAAGATGGCTCTCAGGGA
IFI16_3	GAAGGAGCAGAGGCAACTCC
IFI16_4	GGGAGGCTGAGTCTGTTCCT
IFI16_5	AAAACGCCCAGTGATAGTGA
IFIH1_1	AACTGCCTGCATGTTCCCGG
IFIH1_2	AGTACCTGAATCACTTCCCA
IFIH1_3	AGTGGCTGTTTACATTGCCA
IFIH1_4	AGTTGAACAGCTCTTCCGCA
IFIH1_5	CAGTGTATAATAACATCATG
IGHMBP2_1	AATGATCTCAACCACAGTCG
IGHMBP2_2	GAAGCTCTCCACAGCTGCCG
IGHMBP2_3	GACCCGGGTCAAGATCCCAG
IGHMBP2_4	GCTTCAGCAAACACACGCCT
IGHMBP2_5	CAAGCTCAACTGGAAATCGT
IKBKAP_1	AGGAAGGAGACACAGTTCCA
IKBKAP_2	GACCACAAGTTACCTGGCGG
IKBKAP_3	GTTCGGAGAGAGAAGCACTG
IKBKAP_4	TGTGTTGGGAGTGTAGCCAG
IKBKAP_5	AATGGTGAATGACAGACCGG
IKZF1_1	AACAACGAGGAGCAGCGCAG
IKZF1_2	CAGTGAGGGCGTCCCTCCGG
IKZF1_3	GCTGCACAAGCCGCTCGCGG
IKZF1_4	TCTGGAGTATCGCTTACAGG
IKZF1_5	GAAAATGAATGGCTCCCACA
IKZF2_1	CAGGTCATGAGTCACCATGG
IKZF2_2	GAAATGTGACGTCTGTGGCA
IKZF2_3	GGAGAGGCCTCTCTTTCCTG
IKZF2_4	GTCCAGGAGCTTCAAGGCGA
IKZF2_5	GAACTTAACATATGAGAAGG
IKZF3_1	GAAAGACCTGATGTTACCTG

IKZF3_2	GATAGTAGCAGGCCAACCAG
IKZF3_3	TGTGCAGTTTAATGTGGCGG
IKZF3_4	TTCCAAACTAGCAAGTGCGG
IKZF3_5	AAGATGAACTGCGATGTGTG
IKZF4_1	AAACGGCGAGGGAGTGCGGG
IKZF4_2	ACTCCCATCACAGTAGCCCA
IKZF4_3	TCCGGAACACACCCTCCTGA
IKZF4_4	TCTGGAGAAGGAGTTCCTCG
IKZF4_5	AAGCTGGAGAGGGATCCCTC
IKZF5_1	AGGGCAGGGAACTACATCAG
IKZF5_2	AGTTACTTCGATCACTGCAG
IKZF5_3	CAGGGCAGGGAACTACATCA
IKZF5_4	TCAAAGGAACTATAGTCCAG
IKZF5_5	ATCAGCTCTCGACTCTAGCA
IL4I1_1	AGGGTGATTGTGGTTGGCGC
IL4I1_2	CTTCTATCTCAGCTTCGCCG
IL4I1_3	GAAGCTGCGCAACTATGTGG
IL4I1_4	GAGGCAGATAACAGGATCGG
IL4I1_5	CCAGATGGCTCTCAACCAGG
ILF3_1	AAAGGGCCTCCTACTCAAGG
ILF3_2	AGAGGACGACAGTAAAGAAG
ILF3_3	GAACCACTTGGCGTGTCGGA
ILF3_4	GACACAAGACTTCAGCCCGT
ILF3_5	AGCACATGACCAGAACCCTG
INCENP_1	ACAGGCTCAGCCTCCTCGGG
INCENP_2	GAGGAAGCGCAGCTACAAGC
INCENP_3	GCAAACCTGGCACTCTGCGG
INCENP_4	GGAAAGGCTGGCGAGTCCCG
INCENP_5	ACCTTGGGGTCCATGCGCAG
ING1_1	ACCATGTTGAGTCCTGCCAA
ING1_2	CCAACAGCAAGCGCTCACGG
ING1_3	GAAGAAGCGCTCCAAGGCCA
ING1_4	GAGATCGACGCGAAATACCA
ING1_5	AGCTGGTGGAGAACCGCACG
ING2_1	AATGGTATTGCCCAAAGTGC
ING2_2	GAGGCTCGTTCACTTTCAGC
ING2_3	GCAGCAGCAACTGTACTCGT
ING2_4	GCAGCGACTCCACGCACTCA
ING2_5	AAGCCGTGATTTATGTCACA
ING3_1	ACACCAAACTTACTTTCCAC
ING3_2	AGAGGCATCTGACGTAAGGG
ING3_3	CCAATGTTATAGGATCCCAG
ING3_4	TCAGCAGCCGACTCACGGAG
ING3_5	GAATTACAGAAATATTAGAG
ING4_1	ACACTGCCAAAGGTCACTGA
ING4_2	ACAGGGCCATACCATCTCAT
ING4_3	GGACCTAGACCAAAGAACAG
ING4_4	TCGGCGGCTGGACACAGACC

ING4_5	AGGTGGACAAACACATTCGG
ING5_1	ATAGTGCTCCAAGTACATGG
ING5_2	TCTGCAGGCGCTCCACGCGC
ING5_3	TGATGCGAGAGCTGGACCAG
ING5_4	TGATGTGCTGGACATGCCCG
ING5_5	AAAGAAAAAAGAGGGTCCCG
INMT_1	AGTGGCTGAAGAAGGAGCCG
INMT_2	ATGAAGGGTGGCTTCACTGG
INMT_3	GCTACAGCAGGCACACTCCA
INMT_4	TAAGTAGCCAAGTAGTCCCT
INMT_5	ACAGGCGAATTTCACCGCTG
INO80_1	AGTGGTAGTTAACATCACAC
INO80_2	ATCCCTGGCACCCAACTCCG
INO80_3	GCTAAGTGATGAATCCAGCG
INO80_4	GGAGCAGCGGAAGTTGGATG
INO80_5	CAGAGACAAATCGATATAGG
INO80B_1	AATGAAGAGGAACCTATGGA
INO80B_2	AGGACCTCATGAGTAAGCTG
INO80B_3	TGACAATGGAGACCTCAAGA
INO80B_4	TTTGAGCTGAGGCTTGGCAG
INO80B_5	CAAACTCAAAATCAAGCTTG
INO80C_1	CTTCTGAAAGGGCATTGCCG
INO80C_2	GATGCTCGTGGCCTTCCTCA
INO80C_3	TAGTCCGGAACAGCAAGAAG
INO80C_4	TCCCACAATGGCAGCAGCGG
INO80C_5	CTTCCACTCCCGGAATAGTC
INO80D_1	AGAGAGCGGAGAGGAACCAG
INO80D_2	GAGATTCTGCCCGCTCCAGG
INO80D_3	GTGGAACCAGCAGCATGCAG
INO80D_4	TTGCAGACTGAACCCTGCGG
INO80D_5	AGACCCATTTGCTTTCAATG
INO80E_1	ATCATCAGATAACAGCGAGA
INO80E_2	CAGGAAGAGAAGCCCTCCGC
INO80E_3	GCATCGCTGAACATCTGCCG
INO80E_4	GGAGCACGAGTGCTTCCAGG
INO80E_5	AAGAGAAGCCCTCCGCTGGG
INSM1_1	ACCGCCGCTGGCACAAACCG
INSM1_2	CAAGATCAAGGAGGGCCCGG
INSM1_3	GCCGCGGTTTGTGCCAGCGG
INSM1_4	TGCGTAGGTAGGCCTGGCGG
INSM1_5	CGCCGCTCTACAGTCCCACG
INSM2_1	GAGGGAAGACACGCTCCGCA
INSM2_2	GCAGGAGAAAGCGGCCACGG
INSM2_3	GCAGGCCAGGGACAGCTCCG
INSM2_4	GGAGAAAGCGGCCACGGCGG
INSM2_5	CAGCCGAATAGAGCGGACTG
INTS1_1	GAACCGGGCCAAGCCCACCA
INTS1_2	GGTGCTGTCAGGCTTGGCGC

INTS1_3	TGAGCGCAAGCGGGATGCGG
INTS1_4	TGATGGAGGCGTCCCGCCGC
INTS1_5	GAAGATCACCAACTTGATGG
INTS10_1	GAATTTCCCAGACCAGCCGG
INTS10_2	GAGGCGCTTCCCTGAAACGG
INTS10_3	GCTCGGGTCATGTCTGCCCA
INTS10_4	GTCGTACAGCAGCCTCCCGG
INTS10_5	CCTTTAGTAACATTTCACAC
INTS12_1	AACTTACCTACAAACAACGC
INTS12_2	CACAATACCACACCAGGCGA
INTS12_3	GCAGGCCAATCCCATCTCCA
INTS12_4	GGTCTGGCAACATCATCCAA
INTS12_5	CCGAGATTGTCATAAACCCC
INTS2_1	AAAGTCTTGGAGGAAGGCGC
INTS2_2	ATTAGGCATAAACTTGGAGG
INTS2_3	CCAATGTCAAAGCCACACCA
INTS2_4	CCACTCCAGAAAGAAGGCGA
INTS2_5	AATGTGGGTAGTTAGTAGCA
INTS3_1	GAGCAGCATCCTCATTGCCA
INTS3_2	GAGTTTAGTCTCCATGTCCG
INTS3_3	GTGTTACCGGGACTTAGCTC
INTS3_4	TGCCAAGGATGAGTTAGAGG
INTS3_5	AAACGTCATACAAACACCAT
INTS4_1	ATAGCTGCTGTTCTGACACG
INTS4_2	GAGGTAGGCATAGTGCCTGA
INTS4_3	GTATTGCAAAGCATCTGCTG
INTS4_4	TGTCAAGCTGATCTTCTCGG
INTS4_5	CAAGGAACTTTACAGTTCGG
INTS5_1	ACAGGCTGAGGCCACACCTG
INTS5_2	GAGGAGCCAGGTCAGACAGA
INTS5_3	GCGGAGATCCCGCTCCACGG
INTS5_4	GGTGGCGAGAGGCTTCCCAA
INTS5_5	AGCTACCAATGAGAGCCGAG
INTS6_1	AGCAAGTTGACTACCACCAG
INTS6_2	CCTGGACACGGCCAAAGGCG
INTS6_3	GGTGCAGAAAGTACAAAGTG
INTS6_4	TGGCATAGACAACTATGGGC
INTS6_5	AAAGTCCGGAGCCGATCACA
INTS7_1	AGGGTGATGGCTCTTGCCAC
INTS7_2	GAAGTAATGTTTGATGGCGA
INTS7_3	TGTATGGTGAAGTTGGCCAA
INTS7_4	TTTGAAGAATGATCCCAGGA
INTS7_5	ACTGTGCAATTGAGTCAACA
INTS8_1	CAGGATGAGCGCGGAGGCGG
INTS8_2	CCAACTTACACACTTAACTG
INTS8_3	GAGAAGTACAGCCATGGCAG
INTS8_4	GGCGCAGCATACTTCCAGCA
INTS8_5	AACCAAAGAACTAATTGCAG

INTS9_1	GATCCAGTTGGAGCTCCCAA
INTS9_2	TAGGAGCTAAAGGAGTGCTC
INTS9_3	TCCGCAGTCCAACATAATGG
INTS9_4	TCTGAGAATATCCCACCAGC
INTS9_5	AAGATGCTATACAATGCAAG
IRF1_1	GCATGGCTGGGACATCAACA
IRF1_2	GGAGCACCAACCTTCAGAGG
IRF1_3	TCTAGGCCGATACAAAGCAG
IRF1_4	TTAATTCCAACCAAATCCCG
IRF1_5	GAACTCCCTGCCAGATATCG
IRF2_1	AACCAGTTGAGTCATCTCTG
IRF2_2	CAGCGATGAAGAGAGTGCCG
IRF2_3	GTCTAGCCGCATGCATCCAG
IRF2_4	TAAACTCCAACACGATCCCG
IRF2_5	ACCTGATCCCAAAACATGGA
IRF3_1	AGAAGGGTTGCGTTTAGCAG
IRF3_2	GATCTGATTACCTTCACGGA
IRF3_3	GCAGACCATCTCCTGCCCGG
IRF3_4	TTGGAAGCACGGCCTACGGC
IRF3_5	GAGGTGACAGCCTTCTACCG
IRF4_1	AGGAGCCAAGCAGCTCACCC
IRF4_2	CACAAGCTGGGCCTTGCCAG
IRF4_3	CCGCAGGCGCGTCTTCCAGG
IRF4_4	GCAGGACTACAACCGCGAGG
IRF4_5	CAGACCCGTACAAAGTGTAC
IRF5_1	CATGAACCAGTCCATCCCAG
IRF5_2	GAAGCCAGCAGGGTTGCCAG
IRF5_3	GAGACAGGGAAATACACCGA
IRF5_4	GTAGCTGGAGGATGAGCCCG
IRF5_5	AGAAGCGCTGCTTGTCACTG
IRF6_1	GAGACAGGGAAGTACCAGGA
IRF6_2	GCTTTAGCCGGACTCTGCGG
IRF6_3	TCTGCCCGTACTCCTTCCCA
IRF6_4	TGTGGGCAACTGCAGCCCGG
IRF6_5	AAGGGCTGTATAGGTGCCTG
IRF7_1	ACTCTCCGAACAGCACGCGT
IRF7_2	GCTGAGACTGCGGAGCGCGC
IRF7_3	GGCAGATCCAGTCCCAACCA
IRF7_4	GTGGTGGTGGGACAGCTGCG
IRF7_5	CACCTTGAAGATGCGCGCGT
IRF8_1	ACTCCGACAGGCCTCCGGCG
IRF8_2	GCGTAACCTCGTCTTCCAAG
IRF8_3	GTGGTCGGCGGCTTCGACAG
IRF8_4	GTTCTGCAGCGGCAACGCCG
IRF8_5	ATGGCTCGGAAATGTCCAGT
IRF9_1	ACAATTCCACAGGCCAGCCA
IRF9_2	AGGCAAGCAGGACTTCCGGG
IRF9_3	AGGGCTCAGCAACATCCATG

IRF9_4	GCTGCACGAAGAGGCCTCGG
IRF9_5	AACTGAGGCCCCCTTTCAAG
IRX1_1	AAGACCCTTAATCAGGCGGA
IRX1_2	GGTGCTGGGCATGTACGCGG
IRX1_3	GTGCCGGCGAATCTGGCCTG
IRX1_4	TGAACTGAAGGACAACCCTG
IRX1_5	AAGGAGAACAAGGTGACATG
IRX2_1	AAGCAGCCGAGCCTGGGCCC
IRX2_2	GATGGCGCCGGTCATGCCGG
IRX2_3	GTAGGACGGGAAGCCGGCGG
IRX2_4	TGCCAGCGAGGGCTGCACCG
IRX2_5	AGGCCGCGCTGCCGTACGGG
IRX3_1	CAAGCCGGGAACTTGCCCAG
IRX3_2	GCAGGAAGGCGCCGTAGCCT
IRX3_3	GCTTGTGGCAGTCTCCGCGA
IRX3_4	TTACCCGTCCGAGCGCCCGG
IRX3_5	CAAACGCGAGCTAGAGCTGG
IRX4_1	ACAAGATGACGTGGCCGCCG
IRX4_2	ATGGTTCGGGATCTGCGCAT
IRX4_3	CCACGAGCTCAACTCGGCCG
IRX4_4	GAGCTGTCTCCGCAGCGCGG
IRX4_5	CGTGCTGGTGGTCTCGCGCG
IRX5_1	AGGCGGCGAATGGATAACCG
IRX5_2	CACGTACGAGGAGAAGGCGG
IRX5_3	GTACCGGAAGAACGCCACAA
IRX5_4	TGTGGCCATGTCCTATCCGC
IRX5_5	ATAAAATGACGTGGACGCCG
IRX6_1	AGAAGCTGACTTCCTCTCGG
IRX6_2	GGCGCCCAAGAACAAAGGTG
IRX6_3	GGGTGCTCCATAGATGCCCA
IRX6_4	TGGGCGCCCAAGAACAAAGG
IRX6_5	ACATGGGCGCCCAAGAACAA
ISL1_1	AGTCCAGAGAGACACGACGG
ISL1_2	GAGAGGGCCAGTCTAGGCGC
ISL1_3	GATTGCCGCAACCAACACAT
ISL1_4	TCATGAGCGCATCTGGCCGC
ISL1_5	ACACTCGATGTGATACACCT
ISL2_1	AACCAGACGCGGATGACCCG
ISL2_2	GATGGGACTGCCCGCCACCA
ISL2_3	TACCGGGCAGATGCAGGCCG
ISL2_4	TCCACGACCAGTTTATCCTG
ISL2_5	CGGAGAAGACGACCCGCGTG
ISX_1	AGAGCAGTGGATGTCCACGT
ISX_2	ATCAGAAGACACTTACCCTG
ISX_3	CCTGCACCCGAGCTTCTGGG
ISX_4	GGAGAAGATTGGCAACCTGG
ISX_5	AGAATCAGCGAGCCAAGTGG
ITGB3BP_1	AGTACAGGACTTCCTCACAA

ITGB3BP_2	GATGACTTTCTTGTAGGCTT
ITGB3BP_3	ACTTTCTTGTAGGCTTTGGA
ITGB3BP_4	CTTTGCTTTCAGTTAAACTG
ITGB3BP_5	GAAATTGTCAGAAGAAATCA
IWS1_1	AAGCGCAGACGGAACCGCGA
IWS1_2	AATGAGGATGTCAATCAGCA
IWS1_3	ATGGACTCGGAATATTACAG
IWS1_4	GCCTGCACTCAAGATCCGGG
IWS1_5	ATGAGGATGTCAATCAGCAT
JADE1_1	CACCCGTAGCGGAACCAAGT
JADE1_2	CAGCTCACATAGGAAACCCG
JADE1_3	TCCGGATGAGTACTATGTGT
JADE1_4	TGCGGGTCACCATGTAAGTG
JADE1_5	AAGAGGACAATCTAGCCAAG
JADE2_1	CACTAGAAGTGGGACCAAGT
JADE2_2	GACCCCCAAGACCGACGAGG
JADE2_3	TATATTAGCAGACAACGATG
JADE2_4	TCATCACTACCCACCTCGGA
JADE2_5	CAGATGTGGGCTCATTACGT
JADE3_1	AATAGCTATCAGGATTAATG
JADE3_2	ATAAAGTCCACAGCTAGCGT
JADE3_3	GAGCAGTGAATATACTTCCG
JADE3_4	TCAGCATTGCTTGTCCTGAG
JADE3_5	AAAGTGAGAAAACCAGCCTG
JAK2_1	ATAGAGTTATAGATGGCCAG
JAK2_2	ATCTGCCTCAGATTTCCCAA
JAK2_3	GAGGTGCTACTTCTTTACAG
JAK2_4	TATCGGCATGGAATATCTCG
JAK2_5	AATGAAGAGTACAACCTCAG
JARID2_1	CAGAAGAGGCAGCATGCGGA
JARID2_2	CCTTAGACTGGTCATTACCA
JARID2_3	GTTGCAAAGTCTTACCTCGA
JARID2_4	TGAGCAAGGAAAGACCCAAG
JARID2_5	ACCGACTCGATGTAGATGAG
JAZF1_1	AGGCAGCGAGTATGACGAGG
JAZF1_2	ATAATCTCAGCCGACACCGG
JAZF1_3	GAGTAAGGCTTCCACTGCTG
JAZF1_4	TTAGGGACTCCTGCTCTCGG
JAZF1_5	GGAGCTGATGGCACTCTCTG
JDP2_1	GAAGGGTCCGGGATCTGCCC
JDP2_2	GTCGGTCCGGACGATGCAGG
JDP2_3	GTGGGTTGCCTTCTGACTCG
JDP2_4	TGGGTTGCCTTCTGACTCGG
JDP2_5	AGGGTGCAATCATGGCCCCG
JHDM1D_1	ATAATACTTGCCTCTTCCCA
JHDM1D_2	GATTTCCACATTGACTTCGG
JHDM1D_3	TACGCTCTCGAGTCTTCCCA
JHDM1D_4	TTTAAAGATGGCCGGAGCGG

JMJD1C_1	AATGACCCTAGCCAGACTCA
JMJD1C_2	TAAGCGGTTCCTGTGTGTGG
JMJD1C_3	TATGCCAGTAAACCACTGGG
JMJD1C_4	TTGGCATTACATCACGACGC
JMJD1C_5	AATGGAACACATGCATACTC
JMJD4_1	GAGATGGTGTTTGTGCCCAG
JMJD4_2	GGAGCTATGCGCCGTGCAGG
JMJD4_3	GGCGGGCTACTCCTCTCCCA
JMJD4_4	TCCTCACAGGGACTTTCCGG
JMJD4_5	CTGGAAAGAGTACATACAGG
JMJD6_1	GAGCAAGAAGCGCATCCGCG
JMJD6_2	TAGTTCAGGGCCACAAGCGC
JMJD6_3	TATGGCACAAGACGGTAAGA
JMJD6_4	TGAAAGACCTTACAAGCCCG
JMJD6_5	AGCAAGACGAAGCTATTACC
JMJD7_1	AAGGCCTGAAGCCTTGGTGA
JMJD7_2	ACGTGGTGGAACCACAGAGC
JMJD7_3	GCTGCTCGACTCCCTCACCA
JMJD7_4	GCTGGAAGTAACTATACTTG
JMJD7_5	AGAGCGTTGCGGATAATGCA
JMJD8_1	AGCGCGAGCAACCGCGACGC
JMJD8_2	CAGGATGACGGGCCTGACGA
JMJD8_3	GGAGGAGGAGCGCTGCACGG
JMJD8_4	GGGACAACAACTTCACCGAG
JMJD8_5	AGGCCGCGTACCTTTGTGGT
JUN_1	AACCCAGGCGCGCTGAGCAG
JUN_2	AACGTGACAGATGAGCAGGA
JUN_3	GCTGGAGAGAATCGCCCGGC
JUN_4	GTCATGAACCACGTTAACAG
JUN_5	GCCCCACGTCGGGCGAGGTG
JUNB_1	ACTCCTGAAACCGAGCCTGG
JUNB_2	GGAACCGCAGACCGTGCCGG
JUNB_3	GTACGAGCTCCCGGTCCCGA
JUNB_4	GTGTAGAGAGAGGCCACCAG
JUNB_5	CACAGCTACGGGATACGGCC
JUND_1	ATGTCGATGGGCGACAACGG
JUND_2	CGAGGAGCAGGAGTTCGCCG
JUND_3	GAGGAGCAGGAGTTCGCCGA
JUND_4	GATGAGGCGCTCGAGCTCGG
JUND_5	GCGAACCTGAGCAGCTACGC
KANK1_1	AAAGGGAGTGAGGTCTCCAG
KANK1_2	AAAGTTACCATAGAGGCCAG
KANK1_3	CCAAGTTGATGACATAGCGG
KANK1_4	GAGGAGGATGAAGACACTCG
KANK1_5	AGGCCAGTCATCATTCCAAG
KANK2_1	ATAGATCGCACGCCTGCCGG
KANK2_2	ATTCGGCAGGAACTTCTGGG
KANK2_3	GAAGCTCTCGGTGCTCCAGG

KANK2_4	TCAGCACATACCCACTGCTG
KANK2_5	CACTCAGCCTATTCCTACTG
KANSL1_1	AGGGATTCACATCACAGCCA
KANSL1_2	GGAGCACTCAGACCTGTCAA
KANSL1_3	TACTGAGCTGTAAGAAGCGG
KANSL1_4	TCTGTGCTGAATGGCTGCGA
KANSL1_5	ATGAACACAAGAGTCCAACT
KANSL2_1	AAATGAAGAAGACCAACCCA
KANSL2_2	ACGTCTTGCCAACCAATCGG
KANSL2_3	AGGCTTCCACTCCATAGCGC
KANSL2_4	GGAGCAGGAACCCATTACTG
KANSL2_5	ACAACGAACATCATCCACAA
KANSL3_1	AGTGACAAAGAGCATGCGGG
KANSL3_2	GAGAAAGCACACCCACAGCA
KANSL3_3	GCTGAGATTGCCAGAAGCGG
KANSL3_4	GGGATACCAAGCTGATCCAG
KANSL3_5	ACAGCTGAGATTGCCAGAAG
KAT2A_1	AACATCGTCTGCCGCTCCCG
KAT2A_2	AGAGGAAGGCGCAAGTCCGG
KAT2A_3	AGAGGACACAGACACCAAGC
KAT2A_4	GGCGCTGCCTGCCTTACCCA
KAT2A_5	ATATACTCCTTAGGCATGCG
KAT2B_1	GCGAGCGCGTACCTTGCAGG
KAT2B_2	TATGAAATTGTCCCAGCCAC
KAT2B_3	TCCGATGGAATTAATCAACG
KAT2B_4	TTAGGAACTCACCTAGGGCA
KAT2B_5	ACCTCGGTACGAAACCACAC
KAT5_1	AGTGCCCAGCGAGACAGCCC
KAT5_2	GAGCGGGAGGCCATTCCCGG
KAT5_3	GCTGCCGCCTACCCGTGCTG
KAT5_4	GGCAGAATGGAGCCGCCCGT
KAT5_5	ATGAATGGGTGACGCATGAG
KAT6A_1	ACCAAGACAGAAACTACAGA
KAT6A_2	TTAGGAAGTGCTATTCGCCC
KAT6A_3	TTCGAGTGAAGGCCTTACGG
KAT6A_4	TTTGGCATACGGGTGAGTGG
KAT6A_5	CAACACTAAAGCAACCAACG
KAT6B_1	ATGCGGTCAGTACTTCCCAT
KAT6B_2	ATTGGAATGGGATCAGCACG
KAT6B_3	TCAGGGAGGGCACACTGCAC
KAT6B_4	TCTGGTTGGATACTTCTCTA
KAT6B_5	AAAGGGGTCGTAAACGCAGG
KAT7_1	AATGCAGGCAGTAGTTCAGA
KAT7_2	AGCCGCCGGCAATGCCGCGA
KAT7_3	TATCGAAGCTGTCTCTCCGT
KAT7_4	TCACGATGAGAGCATTGCCA
KAT7_5	ACCAGGTATCAAGCTCATAG
KAT8_1	CTTGGAGAAGGAGCATGAGG

KAT8_2	GGCACAGGGAGCTGCTGCGG
KAT8_3	GTGGGTAGACAAGAACCGGC
KAT8_4	TCTCGAGTGAACGACCAGGA
KAT8_5	CCATACTTACAGTTAACCGG
KCMF1_1	AAAGTGGTGCAACAACAACA
KCMF1_2	AGAATGTTTCACCCTGGCCG
KCMF1_3	ATATGTGCAGCGTTACCTGG
KCMF1_4	GCTAGAACGGCAGCATGCCC
KCMF1_5	AATGCAGTGCATATTAACAA
KCNIP3_1	ACGGTGCGCCACCAGCCAGA
KCNIP3_2	GAGGAAGTGTGCATAGGTGG
KCNIP3_3	GATGAACAGCAGCACCACCA
KCNIP3_4	TTACGCGCAGTTCTTCCCTC
KCNIP3_5	ACACCACTTAGCAAGAAGGA
KDM1A_1	GAATAGCAGAGACTCCGGAG
KDM1A_2	GGATACTACAACTCACCCGA
KDM1A_3	TGGACCACAACAGACCCAGA
KDM1A_4	TTCAAGACGACAGTTCTGGA
KDM1A_5	CTAAATAACTGTGAACTCGG
KDM1B_1	ATAGCCAAGACTTATCGATG
KDM1B_2	ATGTAATGGCAACTCCACGG
KDM1B_3	GAAGCACTTGCAAAGCACAC
KDM1B_4	GGAGAGATGATACCATCGGG
KDM1B_5	ATTCCTCACATCATCGTCCG
KDM2A_1	AGGGTACTGCATCTCCAAGA
KDM2A_2	ATGGATGTGATACCAAACAG
KDM2A_3	GAAAGGCATTGAAATGACCA
KDM2A_4	TCAGAAAGAGTCCCTCAGCA
KDM2A_5	AATGATTACACTAACCGTGG
KDM2B_1	ATGGCGGGTCCGCAAATGGG
KDM2B_2	CGGGTACTTCATCTCTGCAA
KDM2B_3	GAACACCAAAGAGTCTACAG
KDM2B_4	TCGCAGCCAGCTGTACCAGG
KDM2B_5	CTTGGTCAAGCGTCCGACTG
KDM3A_1	AGACAGGAAGAAATTTCCTC
KDM3A_2	GCTTGGACCCATCTACTCAG
KDM3A_3	GTTGTAGGTACAGACTGCAC
KDM3A_4	TCCGCAGCCGACGGCAGCGA
KDM3A_5	CATTCTGTAAGAGCGAAATG
KDM3B_1	CAGCAGCCGCTTGCCCACCG
KDM3B_2	CTACAAGAGATATTCAGCCG
KDM3B_3	GAGGACCCAGTCCTTCTCCA
KDM3B_4	TATGGAGGTGTCTGTAACTG
KDM3B_5	CACATTTGCGACAAACCCAG
KDM4A_1	ACATCAGCTCACCTTGTCAA
KDM4A_2	GAGACACAGTACCTTTGGCG
KDM4A_3	GGAGAGTGGGATCACCATTG
KDM4A_4	TCATCGGGCAGGGCTAGCCA

KDM4A_5	ATATTTCTTCAGCATTAACG
KDM4B_1	CCAGAAGAAGGCCATGACAG
KDM4B_2	GAGTGCGGCACCATCATCGA
KDM4B_3	GTAGTCAATCCACCGCAGGG
KDM4B_4	TGAGGGTCATCTTATGCCGC
KDM4B_5	ACCCGTGATTGAAGCCGGCG
KDM4C_1	GAACTCCCGGAACTCCTCCA
KDM4C_2	GAAGAAATGCATCACACCCT
KDM4C_3	TACCTTGCAGATAACCCAGG
KDM4C_4	TGTTCCATTCATCCACACCC
KDM4C_5	AGCTATTTCTCTCTCCACTG
KDM4D_1	AAGCCTGTACCACTGAGCCC
KDM4D_2	AGGTTGCAGGATGCGCACGA
KDM4D_3	TGAGACAACTCCCTTCCCAC
KDM4D_4	TGGGAGTGAAGAGCACACAA
KDM4D_5	AAATCGGTGAATTATAGATG
KDM4E_1	AGACCCAGTCACTTACCCTG
KDM4E_2	CAGACCCAGTCACTTACCCT
KDM4E_3	GTTGTTGTACTCGAGAACTG
KDM4E_4	TGTGGAACACACAGAGCCCA
KDM4E_5	CACGTTTGCCTGGCACACAG
KDM5A_1	ATGCAAATGAGACAACGGAA
KDM5A_2	GCTGGGATTCAAATAACTCG
KDM5A_3	TAAATCATTACCGACGCCTA
KDM5A_4	TCTCTGGTATGAAAGTGCCG
KDM5A_5	ATCCAGAATGCTTAGATGTG
KDM5B_1	GATCTTGTGGATGAAAGCGA
KDM5B_2	GCAGTGGGCTCACATATCAG
KDM5B_3	TATTGAGGAGGATGTCACAG
KDM5B_4	TCAAGCAAGAACCTATTGAG
KDM5B_5	ACACCTTCGCCTAGTCACAC
KDM5C_1	AATGCCCGATTTCTCTGCGA
KDM5C_2	AGATTCCCAATGTAGAACGG
KDM5C_3	CTGGCAGCCACCCTTTGCTG
KDM5C_4	GGGCTACCCGAGCCCACCGA
KDM5C_5	CCAAGGCTACAACTTTGCCG
KDM5D_1	ACGGTGAAGGATGAGCAAAG
KDM5D_2	ACTCTGCAAACTGTACTCCT
KDM5D_3	TTGTTTCAAAGACTTACCGC
KDM5D_4	TTTGCCAAGTATGCTCCCGT
KDM5D_5	AGTGCATTGAACACTACCGC
KDM6A_1	ATCTCACGAACCCAAAGAGG
KDM6A_2	CCTGGGAGATAAAGCCACCA
KDM6A_3	GTTCATGGCACCCTGCCTGG
KDM6A_4	TTCCTCATCACCGAAAGCGG
KDM6A_5	CAATTGTCAGAAGTATTCTG
KDM6B_1	GAGCATAATATGGTTTGCTG
KDM6B_2	GAGGAAGCTTCGCTGAGCTG

KDM6B_3	TCAGCAGCTCGCTTCACCGG
KDM6B_4	TCGGGCAGTGGACCCTCCAG
KDM6B_5	GACAAAAGTACTGTTATCGG
KDM7A_1	AAGTTCCACCGAAGTCAATG
KDM7A_2	CATCCTAGAGCTCCACACAA
KDM7A_3	CATCTAGTACTTACTCAAGG
KDM7A_4	TACAAATGTGTGGTAAAGCA
KDM7A_5	ACATCCATCACAGAAAATGT
KDM8_1	AAAGTGCCTTCTCTGGCCAG
KDM8_2	AGGGACGGACGGTGCAGCCG
KDM8_3	TGGCTGCCGAACTGTCCCAG
KDM8_4	TGTGCGGCAGGAGCGCCCTG
KDM8_5	AGACCTGAAGTTGGACCTCG
KEAP1_1	AATGAACACCATCCGAAGCG
KEAP1_2	GAGGACACACTTCTCGCCCA
KEAP1_3	GGGCCGCCTGATCTACACCG
KEAP1_4	TCAGGACCAGCTGAACAGCG
KEAP1_5	ACAACCCCATGACCAATCAG
KHSRP_1	GAAGAGTACAGGGTCCCAGA
KHSRP_2	GTCAAGGACACACTGCGCTC
KHSRP_3	GTTGTCGTGGAACTGTCCTG
KHSRP_4	TTGCTTGTAAAGGAACGCGC
KHSRP_5	CAAACCTCTCCGCATCATTG
KIAA1045_1	CCAAGGAGACAGTGCAGCGG
KIAA1045_2	GCCACCAAGCAGCCAAGCGG
KIAA1045_3	GTCATCATCCAGCTTCCGGG
KIAA1045_4	TCAGTGAGGCAGAGTGCCGC
KIAA1045_5	ACCGCCACCAAGCAGCCAAG
KIAA1429_1	CCAACAGCTGATCTTCCCAC
KIAA1429_2	GAATTACTGGACCTCCAGAG
KIAA1429_3	GCCCGCGGCAAACATGGCGG
KIAA1429_4	TCAGAATATTGCCCTTCCTG
KIAA1429_5	AGTGCCACTGATATATCATG
KIAA1551_1	AATCATACTGGACCTTGCAG
KIAA1551_2	ACAGCAGACAGCTTTGTCGA
KIAA1551_3	TAGCAACCTGTCTTTCCCTG
KIAA1551_4	TCAAATGAGAAACACAGCAG
KIAA1551_5	AGACAGGTGCCTTATCACTG
KIAA1958_1	AATTGGTCTGGAGATCACAG
KIAA1958_2	AGAAAGGGCTACCTCACAGG
KIAA1958_3	ATAAGGAGTATGCCCAGCGG
KIAA1958_4	GTATGGCTATAGCATCACCA
KIAA1958_5	CATTGCTCGCAAAAGACCTG
KIAA2026_1	ACTGAAGTGCTACTTGGCAG
KIAA2026_2	CATGTACCTCAACAAGGCGT
KIAA2026_3	GATGTAAGGTAGGTCTGCGA
KIAA2026_4	TTCGCCAGCGGCCAGTACGG
KIAA2026_5	AACTTACAGAGAAAGCGTGA

KIF22_1	AATGCCAGTGTGCTTGCCTA
KIF22_2	GATGGCGGCAGCTTCAGCGG
KIF22_3	GGAGGTCCATGAGAGCCCGC
KIF22_4	GTAATCCGAGAAGACTGCCG
KIF22_5	AAAACTCTACCTGATTGACT
KLF1_1	ACAGGATGACTTCCTCAAGG
KLF1_2	GAAGCCATACGCCTGCACGT
KLF1_3	GAAGTCTGAGGACCAGCCCG
KLF1_4	GGGCAAGGCGGTCTCGGCTG
KLF1_5	CCGCGGGAAGTAGCCACCCG
KLF10_1	AGAGGCACCGAAGTTGAGCA
KLF10_2	GAAGCTACCAAACTGGCAGA
KLF10_3	GCAGAGTTCAAAGCCTCCGG
KLF10_4	TGTGCCCATGAACACAACAG
KLF10_5	AAGAACCCACCTAAATGTTG
KLF11_1	ATGCCGCTTCCTCTCCAGGA
KLF11_2	GAAACAGGTGTCCTTGTCGA
KLF11_3	GAAGCCTTTCAACTGCAGCT
KLF11_4	GTGGCCAGAGCTCTGAGCGG
KLF11_5	ACAGCAGGTCCCACGAACAC
KLF12_1	ACACCAACATATGCCTCCGG
KLF12_2	AGAGCACAAGACGCCAGAGA
KLF12_3	GTCTGGGAGATAGGCCTCGG
KLF12_4	TATGTCTCTTACCTTTGCCA
KLF12_5	ACAGATAACGAGTCCTCCGG
KLF13_1	AAGCACAAGTGCCACTACGC
KLF13_2	AATGCTGCAGCGGCGCGGCG
KLF13_3	GAAGTGGTCCACATAGGCGG
KLF13_4	GCGGCACTACCGCACACACA
KLF13_5	CCGACCTCGAGTCCCCGCAG
KLF14_1	CAGGTGGTGAAGCTGGGCGC
KLF14_2	GGGCTCCTGGGAGAACTCGG
KLF14_3	GGTGTGACAGACCTCCTCCG
KLF14_4	GTAGTCCAGGCACGCCACGG
KLF14_5	CCTCCGGGGCGCCTGATCCG
KLF15_1	GCACGGCCAGGCCAGCATGG
KLF15_2	GCAGCATGTGATATGCCCGC
KLF15_3	TCAAGGTGCACCGCTTCCCG
KLF15_4	TGTGCGAGAAGAAGTTCGCG
KLF15_5	ACGTCATCAGGATCACCCAA
KLF16_1	ACAGCGGTGGCTCTTGGCGG
KLF16_2	AGGGCCCTACAGGCCTGCGG
KLF16_3	AGTCCGGGAAGGGACAGCGG
KLF16_4	GCAGGGCAGCGAGTCGCTGG
KLF16_5	CAGGTGCGACTTTAGGTGCG
KLF17_1	ACACCAGAAGACTCATCGGC
KLF17_2	AGGAGAGCCCAATATTCCAA
KLF17_3	AGGGTAGCCAGGCCCTTCGG

KLF17_4	AGGTCATGGGCATCTTGCGG
KLF17_5	AGTATGCCACTGCCTGAGCG
KLF2_1	AGACACCAGGCCACCCGCGG
KLF2_2	GATGGCACTGGAATGGCCGG
KLF2_3	GATGGGTTCACTCAGCGCCA
KLF2_4	TCAGACACCAGGCCACCCGC
KLF2_5	AAACCAGGGCCACCGAAAGG
KLF3_1	CGGATACTCAAAGGAAGCGG
KLF3_2	GAGGCAATCAGACTAGCATG
KLF3_3	GAGGGCGAATTCCCAGCCGA
KLF3_4	TCTTGCAACAATGCTTGCGG
KLF3_5	AACCTGGGATCGAACCACAG
KLF4_1	AGTGTCTTCTCCCTTCCCGC
KLF4_2	GCCATGTCAGACTCGCCAGG
KLF4_3	GTGGCGGCCACTGACTCCGG
KLF4_4	TCTGGCATGCAGGAACCGGG
KLF4_5	AGCGATACTCACGTTATTCG
KLF5_1	AAAGCCATACAAGTGTACCT
KLF5_2	ACTGCAGTGAAACAATTCCA
KLF5_3	AGGTCCTCTGGAGGCAGCCG
KLF5_4	CGTGCTTCAGCTCCTCGCCG
KLF5_5	GAAGAACTGGTCTACGACTG
KLF6_1	CAGGCACGTACCTGTCACAG
KLF6_2	GCACTTCCGAAAGCACACCG
KLF6_3	GTCGGAGGTAAACTTGGCCG
KLF6_4	TCGGAGGTAAACTTGGCCGT
KLF6_5	TCTAAGTTGTAACAAAAGCT
KLF7_1	AAGCTTTCCTCAATGCACGG
KLF7_2	ATTGGAACGCTACCTACAGA
KLF7_3	CTGCTGCCAGGTCTCCTCCA
KLF7_4	GATCTCAGAGACCTTTGGTG
KLF7_5	ACCTACAGACGGAGCCCCGG
KLF8_1	GAGGAGAGTACAATTGAGAG
KLF8_2	GAGGTCCAACTTAATTCAGA
KLF8_3	GCAGCAATGGCCCAAATGCA
KLF8_4	TGTTGACGTGGACACCACAA
KLF8_5	AGTCTGCCAAATAAGATGGG
KLF9_1	ACCTCACAAAGCACGCCCGG
KLF9_2	CCACTACCGGACCCACACTG
KLF9_3	GAAGTCCATGTAGGCGGCCG
KLF9_4	GTCCAACAAGCTCTTGGCGA
KLF9_5	AGGACTCGACCCAGATTCGG
KLHDC3_1	ACGGATGGCAGACTTCACCG
KLHDC3_2	ACGGTGGACAGTGCACCTGG
KLHDC3_3	AGTGTCAGGGACAGTTCCTG
KLHDC3_4	TAGATACCAGCACCATGACA
KLHDC3_5	ACAGATAAGAGTCCATGTCA
KMT2A_1	ACATGGCGCACAGCTGTCGG

KMT2A_2	AGAAAGGACGTCGATCGAGG
KMT2A_3	ATTGTTGGAGGCATAAGCCA
KMT2A_4	TCAGAGTGCGAAGTCCCACA
KMT2A_5	AAGATCAGTAGCGGTCCCGG
KMT2B_1	AAGATGCGCATGGCTCGATG
KMT2B_2	CGATTCGTAGATCTTCAGGT
KMT2B_3	GGAGCCACATCTGCTAGGCG
KMT2B_4	GGGGCCCAGCTATCCAACCC
KMT2B_5	ACGACCCCAAGTACTGGCGA
KMT2C_1	ACAGCTTCAGATGATCAAGC
KMT2C_2	GGTGACTAGGATGTCGTCGG
KMT2C_3	TGAGGAATCGGCAAACTCCC
KMT2C_4	TTAGAAGGTTGGTTTCTCCA
KMT2C_5	ATTCAGACATTAGGCCATCG
KMT2D_1	GAAGTGGGCAATTCCTCAGG
KMT2D_2	GACAGGCGAGATGCTTCAGG
KMT2D_3	GAGAGGCGTGAAGCCTCAGG
KMT2D_4	GGTGTGGCTCCTCAGGCCGG
KMT2D_5	CCCCTATCCTAGTCCTACGG
KMT2E_1	ATAGCTCTGAGATTAGGCAA
KMT2E_2	ATCCGTAGAAGCTAGCCCTG
KMT2E_3	CATGAGCATAGTGATCCCAT
KMT2E_4	TATGTGAACGTTGTCAGCCT
KMT2E_5	GGATCTGTAGTTATGCGTAG
KNTC1_1	GAGGCAAAGACTTCAACAGA
KNTC1_2	GATGGAGGGAATAAGCTCTG
KNTC1_3	GTAAGAAGGACTGTGCCTGA
KNTC1_4	TAAACACGCTATTACTGCCA
KNTC1_5	AAACATTCGGAACACTATGG
KRBOX1_1	CCATCATGGTGCCTGCCGAG
KRBOX1_2	GGAATGACTGGAGAGCAGGC
KRBOX1_3	GGCTGTGTACTTCACTACGA
KRBOX1_4	TTCCTGCTTAGGACTCCCTG
KRBOX1_5	CAGAGAGGCCTTACCTACAA
KRBOX4_1	TTAGAAACTCACCTGGGACA
KRBOX4_2	ACCAGGTGAAGAGTCCTGGA
KRBOX4_3	CCTATGAATGCAGCTTGCCA
KRBOX4_4	CCTCACCCACGGACACCAGG
KRBOX4_5	TGAGCAGATAGATCACTACA
L3MBTL1_1	GCAGCCGGCCACTTGCTCCG
L3MBTL1_2	GGAATACCAGAGCCCATCAG
L3MBTL1_3	GTGCGGGCCTCACCAAGCCG
L3MBTL1_4	TTGGTGGCCGGAGACAGCCC
L3MBTL1_5	AGACAAGGGACGGGTTCATG
L3MBTL2_1	GGGCTCCAGGACGCTTCCCG
L3MBTL2_2	GTTGATGGCACACCAGCCAA
L3MBTL2_3	TGAGAGCAGCTCCTATCTGG
L3MBTL2_4	TGTGATGAAAGGGATGAAGG

L3MBTL2_5	AACCTGACATCTTGATGCCG
L3MBTL3_1	GATTCGGCTGTACTAAAGCA
L3MBTL3_2	GCCGGCGAAGCTGTTCAAGG
L3MBTL3_3	GTAGATGAGTGTCTTTCTGG
L3MBTL3_4	TATGGACTGGAAAGATGGAG
L3MBTL3_5	AGCACGGAGGAAAAGACGAG
L3MBTL4_1	AAAGAGCGTTTGGATCAGGA
L3MBTL4_2	GAAAGCACACAGAATACCGA
L3MBTL4_3	GTAGCAACACGAATTAACCT
L3MBTL4_4	TATGGTCGCCACACACACCA
L3MBTL4_5	AGCCCTGATATCCACCCGAT
LARP1_1	GCTGCCCTGGCAGACTCCCA
LARP1_2	GCTGTTCCTAAACAGCGCAA
LARP1_3	GGTATGTGTCTAGGCTCCGG
LARP1_4	TAGTGAATACTACTTCAGCG
LARP1_5	AACCTAAAGACACTACCCAA
LARP7_1	AAGATCCAGACCCACATCTG
LARP7_2	AATGTCAGAGCCCTCAGATG
LARP7_3	TCACCCACAACTCTTAAGGC
LARP7_4	TGGAAGGCACCAGAATCCGG
LARP7_5	GAAGAAGCACCAAGAAAACC
LAS1L_1	GAGGAAGACAAAGTTTGCCA
LAS1L_2	GCAGTAGAAGCCACTGCCAG
LAS1L_3	GCCAGGCGACCACGATGCCG
LAS1L_4	TGTGGGTCAACTCATGGCGA
LAS1L_5	CAGGACACATTCTACACGTG
LBR_1	ATACCCATCCAATCAATCCG
LBR_2	CCAAGTAGGAAATTTGCCGA
LBR_3	CGTAATGAAACTCTACGCCC
LBR_4	GAAGGAACGCCTCTTATTGA
LBR_5	CTTCACAGTGTAAAGCTGGG
LBX1_1	CAGCAGCCAGGACTGCTCGG
LBX1_2	CTGCGCGTGCTTGTCCGCGG
LBX1_3	GAAGCGGCGAAAGTCGCGCA
LBX1_4	GCGGACAAGCACGCGCAGGG
LBX1_5	ACTGACGCCGTTCAGCATCG
LBX2_1	ACTCGGCCTGGCCAACGCGC
LBX2_2	GAGCGCGCGCGTCAAGTCCG
LBX2_3	GCGATGCTTAAGAGTGTCCG
LBX2_4	GTCAGACGAGGAGATACAGG
LBX2_5	GACGGGCTAGCTACGCGACT
LCOR_1	ACTGGAGGAAGCAATCTCAG
LCOR_2	GATGAAATTAATGAGGTCGG
LCOR_3	GTTCTGAGTAGTTGCAGCCG
LCOR_4	TCCAGTCACCACCTCTCCCA
LCOR_5	AAGCTTACAGGATGGAACCA
LCORL_1	AGGGCTCCGGCACTGAGCGG
LCORL_2	CGGAGAGAAGAGGATTCCGG

LCORL_3	TAGAAGGGCTTTATGGACCA
LCORL_4	TTCACAGTCAACACCAACAG
LCORL_5	ATTAATTCCTGAGCAACTAG
LDB1_1	ACGGCTTCAGAACTGGACAG
LDB1_2	AGGAGTGTGACAATCTCTGG
LDB1_3	GCATGGCAAGGATGCTGCGG
LDB1_4	TGGCAAGCCCATGTTCACCC
LDB1_5	AGATGCTGCGGAAGTAGCGT
LEF1_1	AGATGGAGGCCTCTACAACA
LEF1_2	GCAGTCAAATAAAGTGCCCG
LEF1_3	GGACCCGGAACTCTGCGCCA
LEF1_4	TCAGGAGCCCTACCACGACA
LEF1_5	CCTGTATATCCCATCACGGG
LEO1_1	ATTGTGGTCGCCCTGCAGTG
LEO1_2	GAAGACAAACCACCTACTCC
LEO1_3	GCGGACGTGAGCGATAATGG
LEO1_4	TCAGATGTAGATCAGCACAG
LEO1_5	AGGCCTCAGATGAAGAACAC
LEUTX_1	AAAGATCTATGACTTGCCAG
LEUTX_2	AGGAGCCAGCGCCTCTGCGA
LEUTX_3	CCAGGATGAAACCCTCGCAG
LEUTX_4	TTGGGAACAGACCTTTACTA
LEUTX_5	ACGAATGTTTGCAGTAGTTA
LHX1_1	AGAACCTGGGCGCCAAGCGG
LHX1_2	GATGTAGAGTTCCTCGCCAG
LHX1_3	GCAGCCGGCACAGTGAACCA
LHX1_4	TGCGGAAGAAGGCGTGGCGC
LHX1_5	GAATAAGTTCGTCTGCAAAG
LHX2_1	AAAGACGGGCCTCACCAAGC
LHX2_2	AGGCCGCCAGATCCGCACCG
LHX2_3	CAGGCGCAACCTCTTACGGC
LHX2_4	GGACCGCAGGGCCAAGAGCG
LHX2_5	ACACTTCAACCATGCCGACG
LHX3_1	AACATGAAGCGCTCCCGCGG
LHX3_2	ACAGAGATCCCGCTGTGCGC
LHX3_3	GACGGGCCTGGACATGCGCG
LHX3_4	GGTACACGAAGTCCTGGGCG
LHX3_5	ACAAGGACAGCGTTCAGGAG
LHX4_1	AATGAATGGGAGCTTCTCCA
LHX4_2	GATGCAGGGCGGCACCGCTG
LHX4_3	GGCGGACAGGTGCTTCTCCA
LHX4_4	TGGGAGCGTCTACTGCAAGG
LHX4_5	GAGGTAGAATTCGTCCCCCG
LHX5_1	AACTCGGGCACCAAGCGGCG
LHX5_2	GACGTAGAGCTCCTCGCCGG
LHX5_3	GCTATGATGGTGCACTGCGC
LHX5_4	TCGTCCAAGCGGCCGCCCAG
LHX5_5	ACCGACGGTCCAAAGAACGC

LHX6_1	AACGGACTCACCATGGCGGG
LHX6_2	CAAGGAGATCTTCTGCAAGA
LHX6_3	GAAGCATGAGAACGCCGCCC
LHX6_4	GCAGGCTCAGTCTGACGCCG
LHX6_5	AGGTTGGGTACCGTTCTCGG
LHX8_1	CAAAGCAAACTCCTCTCCTG
LHX8_2	GAGCCCGAGGCCATGGACCG
LHX8_3	GAGGACTCGCAAAGGCGCCG
LHX8_4	TTTGCAGAACCTCCCTAGGA
LHX8_5	AAGGCAACTTTCCACAGGAG
LHX9_1	CATGCGCTTGGTCTTCTGCG
LHX9_2	GAAGCGGAAGAGCCCAGCGC
LHX9_3	GAGAAGCCCGCCCTGTGCGC
LHX9_4	GGAGCGCAGATCCAAGACTG
LHX9_5	AGCCCTTCCTTACCGCGTCG
LIN28A_1	AAAGCCAACCTACTTTCGAG
LIN28A_2	ACGGATGGATTCCAGACCCT
LIN28A_3	GGTGCACAAAGACATCCACT
LIN28A_4	GTGCACAAAGACATCCACTG
LIN28A_5	AGTGGTTCAACGTGCGCATG
LIN28B_1	AAGGTGGTGGAGAAGAGCCC
LIN28B_2	ACATCGACTGGAATATCCAA
LIN28B_3	ATACGGGTAACAGGACCTGG
LIN28B_4	TCAGAGCATCATGCACATGG
LIN28B_5	CATCGACTGGAATATCCAAG
LITAF_1	CAGCAGGCACAGGCTCCCGC
LITAF_2	GCTGGCTGGGTATAATACGA
LITAF_3	GGACAGCCAGGTCAGAGCAC
LITAF_4	GGTTCCAGGACCTTACCAGG
LITAF_5	AAGGACAACACATTTGGATA
LMNA_1	GGAGAAGACTTATTCTGCCA
LMNA_2	TCTCAGTGAGAAGCGCACGC
LMNA_3	TGAAGAGGTGGTCAGCCGCG
LMNA_4	TTCAGGATGAGATGCTGCGG
LMNA_5	AGTTTAAGGAGCTGAAAGCG
LMNB1_1	GAAACTCCAAGTCCTCAGTA
LMNB1_2	GAAGAGAGCTCACTTTGGCA
LMNB1_3	GCTGGCGGTGTACATCGACA
LMNB1_4	TCTGAAGGATCAGATTGCCC
LMNB1_5	AAAGGCTCTCAATTCTCATG
LMNB2_1	CCGGAAGAGTGTGTTCGAGG
LMNB2_2	GAGGGAGGGCGAGCTTACGG
LMNB2_3	GCTGAGGGCAGAGTTGGACG
LMNB2_4	GGAGGAGGTGACCACGCGCG
LMNB2_5	CATCAAGGCGCTGTACGAGT
LMO1_1	GCTGATCCCAGCCTTCGAGA
LMO1_2	GGGCTCCACCCTCTACACCA
LMO1_3	GTTGGCCTTGGTGTAGAGGG

LMO1_4	TATGAGGAAGGGCAGCTCAA
LMO1_5	CCCACCTCAGGTAGTCGCGT
LMO2_1	ACAGAAATGCTTCTGACAGG
LMO2_2	CTCGTGCCAGTACTGGTCGA
LMO2_3	GCGAACAGGACATCTACGAG
LMO2_4	GCTCTTCCTTTCGATGGCCG
LMO2_5	CGGCTGCCAGCAGAACATTG
LMX1A_1	AACAAACTCATTGGGAGCGA
LMX1A_2	AAGATCAGCAGAACACCCAG
LMX1A_3	GCTGTAGACACTCTGCTCGA
LMX1A_4	GGCAGGGAAAGGAACTGCTG
LMX1A_5	AGATGGTTCTCGGACGTTTG
LMX1B_1	ATGACGGGAAGGACCCGCGG
LMX1B_2	CTGGCAGCCCTCGCAGACGG
LMX1B_3	GATGTTGGACGGCATCAAGA
LMX1B_4	GTGCAAGGGTGACTACGAGA
LMX1B_5	CACCGGAGTCGGACTCGTCG
LNX1_1	GCAGCCAGCAATCAGCCCAG
LNX1_2	GTTGCCAACCATGCCGACCA
LNX1_3	TGGATAATGATATGGACCAG
LNX1_4	TGTCCACACGGAGTGTCCAG
LNX1_5	AATAAAACTGGTGCGCAAGG
LRPPRC_1	GCAAGGCATGACTACCACCT
LRPPRC_2	TAAGGATCTCCCAGTTACAG
LRPPRC_3	TATCTGCCCGCCGCTCGCGC
LRPPRC_4	TGGCGAGTAAAGCACAATGG
LRPPRC_5	CATATGCATTCAATAATGCG
LRRFIP1_1	GATCTCGCGAGCCTCCGCGC
LRRFIP1_2	GCTCAACCAGATCGCGCGGG
LRRFIP1_3	GGCAGCGCTGTACAGACAGG
LRRFIP1_4	TGAAGACGAGCGCATGTCAG
LRRFIP1_5	AAAGACAAACTTCATGTACC
LRWD1_1	GAATCGGGAGCTGACCAGCA
LRWD1_2	GCTCGAGGAACTCAGCCTGG
LRWD1_3	GCTGTCAGGGATGTCCGCTA
LRWD1_4	TTAGCAGCCGCGCCGAGAGG
LRWD1_5	CCAGGTGCGGATGATCTCTG
LSM1_1	AAATACGGTGATATTCCTCG
LSM1_2	AATACGGTGATATTCCTCGA
LSM1_3	GGTGGAACAGCAGACCAAGC
LSM1_4	GTGCAGGCCCTGAAGGACCG
LSM1_5	CTGACCACAAAAATCCCTCG
LSR_1	AAGGACGATGAGCTCTGCGT
LSR_2	ACTGGCACCAGCAGATGCCC
LSR_3	GCTGGAAGAGGATCACCACG
LSR_4	TTGGCCGGCGGGCTCTCCAG
LSR_5	CCTTTGACCAGACGGCGTGG
LYL1_1	CAAGCCGCAGCTCTGGCCGC

LYL1_2	CAGCCAGGTCCAGCTCACAG
LYL1_3	GCTGCTGCAACTCTCCACCC
LYL1_4	GTGGAGAGTTGCAGCAGCGG
LYL1_5	ACCCCTTACAGTGTCTACAT
LZTFL1_1	AACAGCAGAACTCCTAAACA
LZTFL1_2	ACTATAGGCTACAAATGCAC
LZTFL1_3	GAAGTCTCTGAAGTCCTCAA
LZTFL1_4	TGTTTGCACAAGCTGAGAAG
LZTFL1_5	CCTCAATGGATTACAAGCTG
MACC1_1	AATACAATTAGTCAGAGCCC
MACC1_2	GCATATGAAATTCCTCATCG
MACC1_3	TATACCTGGAGTGCTCACTA
MACC1_4	TGCAAAGTAAACCATCAAGG
MACC1_5	AGATCATGGTTAAGCATGTG
MACROD1_1	GAACACGCCCAGGAACGCCG
MACROD1_2	GCCAACAGCTCCCTGCTCGG
MACROD1_3	GGAGGAACATTACTTCTGCA
MACROD1_4	GTGGACGCCATCGTCAACGC
MACROD1_5	CCGCCGAGGTCCGCGCACGG
MACROD2_1	AATGCCAGTCTTCTTGGAGG
MACROD2_2	ACTGTAGGGCCAATAGCCAG
MACROD2_3	CGTGACCAAGATCATCCCGA
MACROD2_4	GAAAGATGAGAACGGTCCAG
MACROD2_5	AGGTGACATCACATTGCTAG
MAEL_1	AATGAGGAGGTAGCTCGCCA
MAEL_2	ACGGCGACGAGGCCTGCCTG
MAEL_3	GACAAAGCTGACATATCTGG
MAEL_4	GATTTATTCATCCCAACCCA
MAEL_5	ATTACCTTGCAGTAGATAGG
MAF_1	AATGTGGCGTATCCCACTGA
MAF_2	GATCACGGCGGACACCACGG
MAF_3	GGTGTCCGCCGTGATCGCCG
MAF_4	TGATGGCTCCAACTTGCGAG
MAF_5	GAAGACTACTACTGGATGAC
MAFA_1	ATGGGCGCCGGCCACCACCA
MAFA_2	GATGGCCGCGGAGCTGGCGA
MAFA_3	GGAGCGGACGACATGGGCGC
MAFA_4	GGTGAAGAAGGAGCCTCCCG
MAFA_5	CATGGACACCAGCTGGTCGT
MAFB_1	AAGCTGTCGAAGCTTTGCAG
MAFB_2	GAAGACGCAGCTCATTCAGC
MAFB_3	TAAGCAGGAGGTGTCCCGGC
MAFB_4	TCACAGAAAGAACTCGGGAG
MAFB_5	ACACGGAGTGCTGAGCGGTG
MAFF_1	GAAGCGCGTGTGCCAGAAGG
MAFF_2	GAGCCGTGTCACCTCCTCGG
MAFF_3	GGAGCGCGAGGTGGACAAGC
MAFF_4	TCTGGCACACGCGCTTCACG

MAFF_5	CGAGAACACGCCGCACCTGT
MAFG_1	CAAGACGGATGCCCGATCGT
MAFG_2	GCAGACCTTCGCCCGGACGG
MAFG_3	TAAAGGAAACAAGGCCTTGA
MAFG_4	TGAGAATGGCACCAGCCTGA
MAFG_5	AGCTGGTGACCATGTCGGTG
MAFK_1	GAGCTCGGTGGACTTGACGA
MAFK_2	GCAGACCTTCGCGCGCACCG
MAFK_3	TGAGAACAGCAGCATGCGGC
MAFK_4	TGCAGCCGAGAAGGGCACGG
MAFK_5	CCTGCGGGGTCTCACCAAGG
MAML3_1	ACACCGTGAGCCTCTACCAG
MAML3_2	ACTGGGTATGGATTCCGCTG
MAML3_3	GAAGAAGAAGGCCTCGCCTG
MAML3_4	GCAGAAGCAACAGTTCCTGC
MAML3_5	ACCTGTTCTCGCAGTTGACG
MAMLD1_1	AATCGAATAGCTGATCCCTG
MAMLD1_2	ACAGAGTCAAGTCTGCCCAG
MAMLD1_3	AGGCAGACCAGACCAACTGG
MAMLD1_4	TTGCCCAGAATTCTGGGCTG
MAMLD1_5	CAGTACTAGGATGAGCACCT
MAP3K12_1	CATGCCACCAGCGATGCCCA
MAP3K12_2	TCACCAGTTCCTCTTCTAGG
MAP3K12_3	TCATGGTCCAGACAGGGCGC
MAP3K12_4	TGTGGAGAGTACATCAGCTG
MAP3K12_5	AACATCATCACTTTCAAGTG
MAP3K7_1	AAAGTTGAGGACCTGGGAAG
MAP3K7_2	GACACACATGACCAATAACA
MAP3K7_3	TAAACACCAACTCATTGCGT
MAP3K7_4	TTTGGGAAGTGATAACGCGT
MAP3K7_5	AATATTAGGATGGTTCACAC
MAP7D3_1	AAGCATGGAAGCATCTCCCA
MAP7D3_2	CAAGCCGAAAGGAAGCCACG
MAP7D3_3	TGCAGCAAACGAGATTGCTA
MAP7D3_4	TTTATCGTACTTTGGAACGG
MAP7D3_5	ATAAGATCGACATTTAAACC
MAPK1_1	CCCGGCAGCCAACATGGCGG
MAPK1_2	CCTACTGCCAGAGAACCCTG
MAPK1_3	GATCCAGACCATGATCACAC
MAPK1_4	TTTGGGAACAGCCTGTTCCA
MAPK1_5	ATCCAGACCATGATCACACA
MAPK3_1	AGTAGGTCTGATGTTCGAAG
MAPK3_2	GCAGTTGCAGTACATCGGCG
MAPK3_3	TGACCACACCGGCTTCCTGA
MAPK3_4	TGGAGGGCTTTAGATCTCGG
MAPK3_5	TTCCGCCATGAGAATGTCAT
MAPK8IP1_1	ATGGCGGAGCGAGAAAGCGG
MAPK8IP1_2	GGACCTGATCGACGCGACGG

MAPK8IP1_3	GGTCAGGTAGATCTCCTCAG
MAPK8IP1_4	GTTCTCCTGCATCATCAACG
MAPK8IP1_5	ACTCATCAGTGATCTCCGAG
MAPKAPK3_1	ATGAAGAGGAGGATTCGCCT
MAPKAPK3_2	CATGGATGGTGAAACAGCAG
MAPKAPK3_3	GCCTCACCTTGACATCTCGG
MAPKAPK3_4	TGAGGAGACAGCGCTTGCCA
MAPKAPK3_5	ACATAATAGGGAGTATAGCA
MARCH5_1	ACAGCAGCAATATTTACGAC
MARCH5_2	ATTAGGCAAGATGATTCGCT
MARCH5_3	CAGGTTGTAGGTCATAAAGA
MARCH5_4	TATTAGGCAAGATGATTCGC
MARCH5_5	AGGACTATGTGCTTAGACTG
MASTL_1	ACAGACATGGAATCATCCAC
MASTL_2	ATAGTGAAGCCCATTAGCCG
MASTL_3	GAAACATCACAGCTTTCTCA
MASTL_4	TAAGCGATAACACTTGTCCT
MASTL_5	AAGCGATAACACTTGTCCTG
MATR3_1	CTTCCATGGACTCTTACCGA
MATR3_2	GAGATACCAGCTATTACAGC
MATR3_3	GAGTGGAGTCAACATATCAA
MATR3_4	GGTTGTGTAATAATCCACTG
MATR3_5	AAATTTATGATAGTTATGCG
MAU2_1	ATGCAGCAAAGCCGCTGCTG
MAU2_2	GCAGGCAGTGCACGCACAGG
MAU2_3	GCGCTGTTCCTCCTCAGCAA
MAU2_4	GTATTCAGATCCCACCACCC
MAU2_5	AATTTAACATCTTCGAACTG
MAX_1	AAGCAGGAAGAAGCTCCGGA
MAX_2	CAGCCTCTACACCAACGCCA
MAX_3	CGATAACGATGACATCGAGG
MAX_4	TATTCCAGGAAGAGCAACCG
MAX_5	AATATATCCAGTATATGCGA
MAZ_1	ACAGGATGCGGTCAGCCCGC
MAZ_2	AGAACGGCTACAATCTCCGG
MAZ_3	TGAGGCAGCTTTCGCCACGA
MAZ_4	TGCTGGGCCTGGACTCCCGG
MAZ_5	CCCTGCCGCCGCCTCTACGG
MBD1_1	AAGAGGGTGAGATCACACGC
MBD1_2	GCTCAGACACCTATTACCAG
MBD1_3	GGGATGGCACCCAAAGGCAG
MBD1_4	TACTTTCCAGGCCCATCCCG
MBD1_5	ACGTTGTGCAAAGACTGTCG
MBD2_1	AAAGGGACCGGCTCCCGCCG
MBD2_2	AGGGCAAGTCTCCGCTGCTG
MBD2_3	GATGTGTTAAGCCAAACAGC
MBD2_4	GGAGCCGGTCCCTTTCCCGT
MBD2_5	AGCCGGTCCCTTTCCCGTCG

MBD3_1	GAGCACCTTCGACTTCCGCA
MBD3_2	GAGCGACCCGCAGAAGGCGG
MBD3_3	TGAGGAGCTGGTCAAGACCA
MBD3_4	TGGTGTTGAGCCATACGCCG
MBD3_5	AAGACCATGGACCTCCCCAA
MBD4_1	ACCAAACTGAGCAGAAGCGA
MBD4_2	GCGCTCACTAGAGGTGACGG
MBD4_3	GTAGCGATGAGAAGCTTCCA
MBD4_4	TCAGAGTTGCAGGAGAGCAG
MBD4_5	ACTTACCGGAGGTCATTCGG
MBD5_1	AATGGGAGATTACTTGGCAA
MBD5_2	CTGGTGCTCACCAGTCCCGG
MBD5_3	GAGGCAAAGAGTGTGACGGA
MBD5_4	GCAAGATAACAAAGACCCAC
MBD5_5	CTTGAGTAGCAATAGTACCC
MBD6_1	GTAGCTCCGGGTTTGCTCCA
MBD6_2	GTAGGAGGAACTCGACAGGG
MBD6_3	GTGCTTCAAAGCCCATCCGA
MBD6_4	TAGCAACAGCTTTCCGCCGG
MBD6_5	AATGCTCCCTCATACAACTG
MBIP_1	ATCAAGAGAAGCAGAATCCA
MBIP_2	CGTAGAGCACCTCTCGGGAG
MBIP_3	GAAGTAGTTCAGATAAAGGC
MBIP_4	TTAGGTTTATGACCTGACCC
MBIP_5	AATCAATGAAAACAACGTCA
MBNL2_1	AAAGGATGAAGAGCACCAGG
MBNL2_2	CCAGCGAGGAAACTGTGCCC
MBNL2_3	GAGCCCGGGACAGTGACCGG
MBNL2_4	GGAGCAAACCTGATTGGCTG
MBNL2_5	CTCAACTAGAAATTAATGGA
MBTD1_1	ATTGAACTTACTCCACCCAG
MBTD1_2	GTGGTGGAAAGTGTAATTGG
MBTD1_3	TCCATGGAAGGTTTCAGCTG
MBTD1_4	TTACTCGTCAAACTCCAAGA
MBTD1_5	AGCTTGATACTGAGCATATG
MCM2_1	AGAGAACCGTGAGAGCCTGG
MCM2_2	ATAGGCGTCCAGCTCTGGGA
MCM2_3	ATGGTGGAAGGTCACGGCCA
MCM2_4	GGTGGTGGATCTCCAGCCGG
MCM2_5	AGAATGGCGTCCTTGGAGCG
MCM3_1	AAGAAGGGAGGCTACACCTC
MCM3_2	GAAGGCAACCAGCTCCTCAA
MCM3_3	GGTAGGATAGACAGAGCTGG
MCM3_4	TCAGCCCTCTTTCTCTGCTG
MCM3_5	AGAATGACGTCCACAGAGCG
MCM4_1	AGTGGATCTGCAGTCTGACG
MCM4_2	CGAGGATTCCACCTCCACGG
MCM4_3	GGACTCACGCGTCTGGGCGG

MCM4_4	GTGTGCCCACACGACCCGGG
MCM4_5	GATGTTCTAAGATTGAGTCA
MCM5_1	CAAGCGGCATTACAACCTGG
MCM5_2	GAAGGTGAAGCCCGTGCGGT
MCM5_3	GCACTGGATAGAGATGCGGG
MCM5_4	GCTGGAGGAAGCTGCCAAGG
MCM5_5	CCTGATGGACTTACTCGCCG
MCM6_1	AAGAAAGGTCCCTAACACCA
MCM6_2	AGCAAATATGGACCTCGCGG
MCM6_3	GAGAAGGGACCTCTCTTCGA
MCM6_4	TAAAGAGATCCCTCTTGCCA
MCM6_5	AGAAGGGACCTCTCTTCGAG
MCM7_1	GAAGGACTACGCGCTAGAGA
MCM7_2	GCGGAGTCGGGACCCTGGGA
MCM7_3	GGACCTGGACGACGTAGCCG
MCM7_4	TGAAGTCAAACCCAAGATGG
MCM7_5	AAGCCCAACTACTTACTGCG
MCRS1_1	AACGCTCCTGGACCTCCCGA
MCRS1_2	CATGAGGCAGCTGCACCCAG
MCRS1_3	GCAGAAGCGAGCCTCCTCCC
MCRS1_4	GTCCTGGAAGGTCTCCAAGG
MCRS1_5	ACACAGCATTTATGAGCAGG
MDC1_1	GAGGTGGAAGGCTGAAGCTC
MDC1_2	GCAGAGAGACATCCAGGCGA
MDC1_3	GTAACGTGGAGCCAGTAGGG
MDC1_4	TGCGGGCACAACTGTTTCAG
MDC1_5	AATGATACAAAAGTCAAGAG
MEAF6_1	CATGGCGATGCACAACAAGG
MEAF6_2	CGGGTGTCCGGGATCTGCGG
MEAF6_3	GCAGATCCCGGACACCCGGC
MEAF6_4	GCAGGGAGTGAAACCTCAGA
MEAF6_5	ATGTATGGCAATATTATTCG
MECOM_1	ACAGCATGAGATCCAAAGGC
MECOM_2	AGTAGTTGCAGACATTGCGC
MECOM_3	GCTGAGTGAGGAGTACTGCA
MECOM_4	TCGAGAGTCAAATATGCCTG
MECOM_5	ACTGCCCATACTTAGATCCA
MECP2_1	AGAAGGGTCAGGCTCCGCCC
MECP2_2	GAAGCGCAAGACCCGGGAGA
MECP2_3	GAGCGGCACCACGAGACCCA
MECP2_4	GGACACGGAAGCTTAAGCAA
MECP2_5	AGGGTGGGGTCATCATACAT
MED1_1	AAGATCAACACTGTTGGCAC
MED1_2	GAAGTGCAGTTAGATCCTGC
MED1_3	GTGTTGGCTATCTCACACCA
MED1_4	TGAGTGATCAGTTCATACAG
MED1_5	CCAACCAACACCTTTCCGGG
MED11_1	GCGGCTCCTAGACCGGCAGG

MED11_2	GCTCCTAGACCGGCAGGCGG
MED11_3	TGTGATCCTAGAATTGTCCA
MED11_4	TTCGAGGAAGGACTGTCAGA
MED11_5	ATCCCGAACCTGCATTCTGA
MED12_1	TAAGCAATTCATCCTCTCCA
MED12_2	TACAGAAGTAGGCAAGCCGG
MED12_3	TAGAGGTGGCAATCCGGCAG
MED12_4	TTCGTAGCTCAAGATCCCGA
MED12_5	ACATCGACTGCTGGACAATG
MED13_1	ACAGTGACAATCTTCCAGGC
MED13_2	GAACAGTGAGTGGATCCCAC
MED13_3	GATAGTACTAGCCACCATGG
MED13_4	TGGCGGCGAGATCAAAGACC
MED13_5	ACAAGGGTGAGGACTAAGTG
MED14_1	GGTGCAGCAGAAATTCAATG
MED14_2	GTTGAGGATAAGGAAACAGG
MED14_3	TAACTACTGGATCTTACCCA
MED14_4	TAATGTTAATCCGAGAACGG
MED14_5	ATCACACATAGCGACGAAGT
MED15_1	ACTGACCAGCTTCTGCCGGA
MED15_2	AGGAGGCATGCCAATTCCAG
MED15_3	GGACGAATACCTTTCTCTCG
MED15_4	TTTCCTGAAGGCCAAGACCC
MED15_5	GGAATTGGCATGCCTCCTCG
MED16_1	AAATCTCATCGCCTTCACCA
MED16_2	CCAGCACTTGATCTGCCCGT
MED16_3	GGTGCAGCGCATGAACAGGG
MED16_4	TGACTGGTCCCACTCCAGGC
MED16_5	CGACGGTGAGAACTTGACTC
MED17_1	CAGCGGATAGACTTCAGCCA
MED17_2	GAGTCCCAAGGCCAAAGGGA
MED17_3	TATGCTCTAGGACATAAAGG
MED17_4	TGAAGAGGTTAACTGTGCCG
MED17_5	ACTTCAAATGTACCATGATG
MED18_1	ACAGGCATCATGGTGACTGG
MED18_2	AGAGACCATGTCCTGCCCAG
MED18_3	GATGGAAATGGGCTTCCGCA
MED18_4	TAAGGAGGAATACCATCTCA
MED18_5	ACCATGATGCCTGTCACTGG
MED19_1	GACAAGTCAGGAGCTGGCTG
MED19_2	GAGGCGGAAGCCGGCCAGCA
MED19_3	GCACAAACAGAGCCGTACCC
MED19_4	GCTTCTCAATGAGAGAGCGG
MED19_5	CACCTGGCAGTTCCCTCATG
MED20_1	AAGAGGGCGAAACAGCTCAA
MED20_2	GTCCTACCAAGTCACTCCCA
MED20_3	TCTAGGCAGCCACACACCAG
MED20_4	TTGCTGAACACTCTTGCCCT

MED20_5	AGGTGGGCACGGTCACAATG
MED21_1	ACATTGCTGCAATACTCCAA
MED21_2	AGTCTGGAAGAGACTGGCTA
MED21_3	CAGCCAGCTAACCCTACAGA
MED21_4	TTGCTGCGGTAGGAACATGG
MED21_5	ATATTATTGAAAGAGGCAGG
MED23_1	AATGGAGTTTATCCTTGCTG
MED23_2	ACCGAGGAGAAGTTTGACGA
MED23_3	ATTGGAGCAGCCTTATTCCA
MED23_4	CTGAAGGCAAACTTCCACAC
MED23_5	AAGGCCATGCGTAGTCACGA
MED24_1	ACTAATGGCATACTTCAGGT
MED24_2	CAGGTTGAGAATATCCCAGG
MED24_3	TAGCTGTCACGGCAAAGCAG
MED24_4	TCGGCCTGACTCCGGAGCTG
MED24_5	CCTCGAGCAGGATCACGGCG
MED25_1	AACCTGGGACCCTACTTCGA
MED25_2	ACAGCAGGCAACAAGTATGG
MED25_3	GGGAGCGTGACATTGTACGT
MED25_4	TGAGAGCTGCAGCCTCATCG
MED25_5	AGTTGTGCATCCAGAGTACG
MED26_1	AATAGGGTATTTCTCCAGGC
MED26_2	ACAGATCCGGAACATGGTGG
MED26_3	GACGAGAATGACAAGCACAG
MED26_4	GAGGACTGAAAGAGCAGCGA
MED26_5	CCTCGGAACTCACGGCATGA
MED27_1	AAATCCATCACTCACTGAGG
MED27_2	AACCATCCTCTTCATAACAG
MED27_3	GAAGCGTTCCGCTAATCAGA
MED27_4	GGTGTCATGGAAGGCTTCGA
MED27_5	AGCATATAAGTGGTCAAACA
MED29_1	AAGCCGCTGAAGCTTGCTGC
MED29_2	CAGTCGTAACGCACTTCCGG
MED29_3	GTTGGCACAGTCCAGCAGGG
MED29_4	TTGGGCCGCAACCTTCATCA
MED29_5	AGCATCTTATAACGCTGCAC
MED30_1	AATGGTGTCACTTACCACAC
MED30_2	CAGGAGCTGGAAGATCTCCA
MED30_3	GGACATCGTGTACCGCACCA
MED30_4	TCAAAGAATGATGATCGGGC
MED30_5	ACACTGGAACATATCAAGAC
MED31_1	ATGATGCTGGAAATCGACTT
MED31_2	CCAATATGAACACTTCCGAA
MED31_3	TATGGCCGCTGCTGTCGCTA
MED31_4	TGTCTCCATAGCGACAGCAG
MED31_5	TCTAACATGTGTAAACACTG
MED4_1	AACAGCTGTTTACCAAGCGA
MED4_2	GAGTTGTTAATTCACCGAGA

MED4_3	GTGGTAACAGCACACGAGAG
MED4_4	TTACCTGGAACCCAGGTCAG
MED4_5	AAATAATTAAGTATGCACAT
MED6_1	ATGGCGGCGGTGGATATCCG
MED6_2	GAAGTGCCACCAATACCCTT
MED6_3	TCATCATTCGGAAGCAACAG
MED6_4	TGTCGATATCATCCTTCCAA
MED6_5	ATCAGTTATAAACTCTAGAG
MED7_1	GACCTTGAGAGTCATGATGG
MED7_2	GGAAGTGCACTCACTTGCTG
MED7_3	TCGATGCCCTGACTTTCCAA
MED7_4	TGGAGGTCCAGAAACGTCAA
MED7_5	GGATGATAAGATCATCACAT
MED8_1	AAAGGCAACAGCTGCCACCA
MED8_2	GAGGAGCGAGAATCAGAGAG
MED8_3	GATGGTCAGGGACTACCTCA
MED8_4	TAACCAGGTCATCATTCCTC
MED8_5	ATGACCTGGTTACGGAACAG
MED9_1	GGTGACTGAGGCCGTGGCGC
MED9_2	GGTTGAGGCGCAGGGACCGG
MED9_3	GTCAGCCTCACTCTACTCCT
MED9_4	TGGTCCTGACTTGCTCCCGG
MED9_5	GGCAGCGGCTGGTCGGACGT
MEF2A_1	AATGGATGAAAGGAACCGAC
MEF2A_2	GTAGGAATTCACTCACCCAC
MEF2A_3	GTTAGTGTAGGACAAAGCAT
MEF2A_4	TGAGCACAGTCCACTCTCGG
MEF2A_5	GAGTTGGTTCTGCTTTCATG
MEF2B_1	CTGGGCTGGGAGGACACGGC
MEF2B_2	TCAGGGCACTTACCTGGGCT
MEF2B_3	TGCCCACCAGAATATGGCCT
MEF2B_4	TGGGCTGGGAGGACACGGCG
MEF2B_5	AGGCCCCGTATACCACATCT
MEF2C_1	AAGCGCATGCGACTTTCTGA
MEF2C_2	ACCGCCAGCGCTCTTCACCT
MEF2C_3	GACAAACTCAGACATCGTGG
MEF2C_4	TGGCTCACCCTTCTCTGCAG
MEF2C_5	ACTCCTACTTTACCAGGACA
MEF2D_1	CAACGCCGACATCATCGAGG
MEF2D_2	CTGCAGTTCAGCAATCCCAG
MEF2D_3	GAGCCCGTCGAGCTCCTCGC
MEF2D_4	GATGACTGCACTCACCAACA
MEF2D_5	CAAGTACCGACGCGCCAGCG
MEIS1_1	GACAGAGGAGCCCATGCTGG
MEIS1_2	GGTGGCCACACGTCACACAG
MEIS1_3	TCAGTGCTAAGAGAGGGAAG
MEIS1_4	TTGGTGATAGACGATAGAGA
MEIS1_5	ATCATGATCTCTGTTCCAAG

MEIS2_1	AAACAGTTAGCGCAAGACAC
MEIS2_2	ACGGAGACCCTCACGCGCCG
MEIS2_3	AGAGCTAACAGAGGAAACAA
MEIS2_4	GCAACCTCAACCCACTCAGC
MEIS2_5	CCTTCAAACAGCTAATGTAT
MEIS3_1	GAGACCTACCCTCCGGGCCA
MEIS3_2	GAGCAGACGTCACCTCCAGG
MEIS3_3	GATGAGCTGCCGCACTACCC
MEIS3_4	GGTGACAGAAGTTGTCGCAC
MEIS3_5	AGACAGTGCCCGCAGTACCA
MEN1_1	AAGGGCAACGAGGACCGCAG
MEN1_2	CCGATAGTAGGTCTTGGCTG
MEN1_3	GAGTGGGTCTGGCCGGCCAG
MEN1_4	GATGATAGACAGGTCGGCCA
MEN1_5	AGGATAGAGGGACAGGTCGA
MEOX1_1	AATGGGCAGGACCCTGAGGA
MEOX1_2	AGACTTCCTGGCGACAGCGA
MEOX1_3	GGAGAGGACGGCCTTCACCA
MEOX1_4	GGAGGCCCAGGCGATGACTA
MEOX1_5	AGGCCCAGGCGATGACTACG
MEOX2_1	AGTGCGCATGCTCTGAGCTG
MEOX2_2	CAGAAGTCAACAGCAAACCC
MEOX2_3	GAGGCGGAGAAGCGAAGCGG
MEOX2_4	TTTGCCAGCCAGCATCACAG
MEOX2_5	GATGTCTTCCCCACCGAGTG
MEPCE_1	AAGTGGGTGCATCTGAACTG
MEPCE_2	GCTGGAGAAGCCCACGTCTG
MEPCE_3	GGAAATGATCGAGATGGCGG
MEPCE_4	GGAACAGGTACACAGGACGC
MEPCE_5	AGTGAGCCGCACTCTCAACG
MESP1_1	GAGCCAGCACGTCGCCCGGA
MESP1_2	GCGGCCGCAGTCCTTGTCGG
MESP1_3	GCTGGAGACTCTCCTCGCTG
MESP1_4	TCCTCAGGCAGCCACTCCAG
MESP1_5	CCCGCGCCCCCTCCGTAGGT
MESP2_1	TAGGTGGCCGATGTAGCGGA
MESP2_2	TGAACCGCAGGCCTGAGCTC
MESP2_3	TGGCCGAGGAGGCTCTGCGG
MESP2_4	TTTAGTAGAAGATGCCCAGG
MESP2_5	ACGGGGGCGACTGTATCTTG
METTL1_1	AGAGGCACACGCTCAAACAG
METTL1_2	TAGGCACCTCAACTGAGGAG
METTL1_3	TCAGAGCCACGATGACCCAA
METTL1_4	TCGGAACGTGGCCGGAGCAG
METTL1_5	GACCACACGCTGCGCTAGTG
METTL11B_1	AGAGGCCTGGATGTCTGGGC
METTL11B_2	GAAGGACAATGTGGCCCGGG
METTL11B_3	GATGACATCATATCTCCTGA

METTL11B_4	GCTCTGACTCACTTACCCAG
METTL11B_5	ACATCATATCTCCTGAAGGG
METTL13_1	AGTGGGCCACTTCTCCCGGG
METTL13_2	ATTACAGTGGCCCTTCACCG
METTL13_3	GAGAAGTTCTTCCAGCAGCG
METTL13_4	GCAGCGGCCTGCTGATGCGG
METTL13_5	AGGAATGTAATGCCACCCGA
METTL14_1	CGTGGAGGAGCACACAGAGG
METTL14_2	GAAAGAGGTGGCTTTAGAGG
METTL14_3	TATTTAGGAACACTGCCTCA
METTL14_4	TGTGAAGCGTAGCACAGACG
METTL14_5	ACCATCTTACCACTCTTCCA
METTL15_1	ATTCACAACATCAGCAGCAG
METTL15_2	CAAAGGGCCATCTTTCCGAA
METTL15_3	CCAAAGGGCCATCTTTCCGA
METTL15_4	GATATGACATTTGGTTCGGG
METTL15_5	GCTGCTGGGTTCTGGTGATG
METTL16_1	GCCACAGAAGACACTCCTGA
METTL16_2	GCTGGGAAAGAAATGCAGCC
METTL16_3	GTATTAACAGAACTAGGCGG
METTL16_4	GTGGGAATTAGTCTCTCCAA
METTL16_5	AGATGGTATAGCTGCATGCT
METTL17_1	ATGGCAGCAAGACTGGATGG
METTL17_2	GAAGAAGAAGCCTTACCTTA
METTL17_3	GCGGCAGCTTTAGGATGCCA
METTL17_4	TAGGCACTCACCCGGGCCTG
METTL17_5	AAAAAATTCCTGGAAAACCC
METTL18_1	AGAACAATTTGACTTGCCTC
METTL18_2	ATTACAGGTGTTTATGAGGG
METTL18_3	TAGAGATGGAGCTTTGACCC
METTL18_4	TCTTTGGCAGCTCTCAAGGA
METTL18_5	CCTTTGTGAAATAAGCCAGG
METTL20_1	GGTGGGAGAGAGCTGACCTG
METTL20_2	TCTGTATTAGATCTTGGGAG
METTL20_3	TGAGATAAAGGCTTTCCTGG
METTL20_4	TTGCAGATAGTCTTCATCAG
METTL20_5	AAGCCGCAACTGGATTTCAG
METTL21A_1	ACACCTGGGAGTCGCAGCGG
METTL21A_2	CAGGTATGTGGAAAGAACGA
METTL21A_3	CGTGCCAGCACCCAGCTCCA
METTL21A_4	GAGACACCTGGGAGTCGCAG
METTL21A_5	AGGTGCTCATGTGACTATCA
METTL21B_1	AAGGTGATCGAACTGGGTGC
METTL21B_2	AGGCCAGATAGATGGTGCCA
METTL21B_3	CTGGCCCTAGAACAGATCCA
METTL21B_4	GGACCCAAAGTTCTGCGTGA
METTL21B_5	AGGTGATCGAACTGGGTGCG
METTL21C_1	ATACTTGGAGGAACATGCCG

METTL21C_2	CATAGAGAGTTACGGAGCGG
METTL21C_3	TCCGCAGAAAGACAGCACCG
METTL21C_4	TGGCTGGGAAAGGTACACCA
METTL21C_5	AGCCTCTAACCAGCCACCCG
METTL22_1	ACAATAAACGGTCCGTGCCA
METTL22_2	ACAGGAAGAAGACGACGTCC
METTL22_3	CATGGTACGGCTGAACAGCG
METTL22_4	TGCTTGCCAACATCCTCCAG
METTL22_5	ATAAACGGTCCGTGCCATGG
METTL23_1	CAGGTGGTAGGACTAACATG
METTL23_2	GCAAGGATAATATCTTGTGG
METTL23_3	TCAGAGACTGTCCTTTGCAA
METTL23_4	TTTGATGCACAAGAATCCCA
METTL23_5	GATGTGTTCTTTGAACCAGA
METTL24_1	TCGTCAAGACACACTGACCA
METTL24_2	TGGACAAGCGGTGATACCAA
METTL24_3	TGGAGATCTATATGCCACCG
METTL24_4	TTTGCAACTGACCTTCCGGC
METTL24_5	CAGGTGACCTACGTGCGCAG
METTL25_1	GAGCCAGATCACCACAAGTG
METTL25_2	GAGCGGGTTGCAGCTGGCCA
METTL25_3	GAGTGTGGAAGCCTTTGCTC
METTL25_4	TAAGCCTCGAATGAATGAGC
METTL25_5	AAAATCAGGCAAAATAGCTG
METTL2A_1	CGCAGAAGCATCATCCCGCC
METTL2A_2	GCTGCTCCACAGGATGCAGA
METTL2A_3	AGCCACCTACCGAATACTGG
METTL2A_4	ATCACACAGGTCGTGAACAA
METTL2A_5	GCGCCGGATCTCTCAGGAAC
METTL2B_1	GTGTCTCCACAGGATGCAGA
METTL2B_2	TAAAACACAGACACCTCCTG
METTL2B_3	TCCAGGAGAACAGTATCCAG
METTL2B_4	TGTTCTTCCATTATTAAACC
METTL2B_5	TTGTGGTGGAAGACGCGCGC
METTL3_1	ATTCACATGGAACTGCCCTA
METTL3_2	GAAGCAGCTGGACTCTCTGC
METTL3_3	TCTGAACCAACAGTCCACTA
METTL3_4	TTAAGGCCAGATCAGAGAGG
METTL3_5	ATTCTGTGACTATGGAACCA
METTL4_1	AAAGACTACATCAAGCCAGA
METTL4_2	ACTAAGCCAGAGAATGATGA
METTL4_3	TCAAGGTGAATTGGATGCTA
METTL4_4	TTTGCTCGAAATTTACAGCC
METTL4_5	ACATCAGACATTACAATTGG
METTL5_1	AGAGAGTCGCCTGCAACAAG
METTL5_2	AGTCGCCTGCAACAAGTGGA
METTL5_3	GTCGTTGCAGATCTAGGATG
METTL5_4	TGACATAGATGAAGACGCAT

METTL5_5	AATTACTGTATCGAATGACT
METTL6_1	ACAATGCTTGAAGCTGGCTG
METTL6_2	CAGAGATGGCTTCTTTGCAA
METTL6_3	GCTGGCTTTAAACCTAAGCA
METTL6_4	TAGCTCCTCAAACTCTCTGG
METTL6_5	ACAACATCCACAGACTCTGG
METTL7A_1	AGAGAAGCTGGCCCGCTCCA
METTL7A_2	ATACAGGTCACCCTGCACCC
METTL7A_3	ATGAGGGCGCACCAACTCCC
METTL7A_4	CAGCAGGGAGAGTTTCCCGG
METTL7A_5	AGGTGGGTAGAACTTGAAGT
METTL7B_1	ACAGGTAGCCACTTCAAGGG
METTL7B_2	AGTCAGCACGGCCATCAGGT
METTL7B_3	CAGTAGGGCCACTTTCCCGG
METTL7B_4	GTAGAACTGAAAGTTGGCTC
METTL7B_5	AAGCTACTTCCCCTACCTGA
METTL8_1	CCAAAGTGGTTACCACCCAG
METTL8_2	GAAGGCGAGAGAATCATCAT
METTL8_3	GTCCAAGTTACTGAAACCTG
METTL8_4	TTAATAGGGATCACATGCAG
METTL8_5	GGAAACAATCCAGTCTCCAT
METTL9_1	AACATGACTAGCGGCCCGGG
METTL9_2	ACAGCATTGAGAAATCGGGC
METTL9_3	GTTCAGAGTTACCTTGATCA
METTL9_4	TCTTGGAGCCAACTAGAGGC
METTL9_5	CTGTACGTGAACATGACTAG
MGA_1	AAAGAATTCAGTCTGTGGGA
MGA_2	AAGAATACACCTGTAAGCCC
MGA_3	AAGCTAGTAGCTGGAGCCAA
MGA_4	ATACATCCTGGTCTTCAAGA
MGA_5	ACTGGAATCAACAACAATCG
MGEA5_1	CAACCCTGCCGCCTCTGCGG
MGEA5_2	GATCCACAGAACTCATCCCA
MGEA5_3	TCAATGTGGATACTTCCTTG
MGEA5_4	TTGGCGAGAGATGTATTCAG
MGEA5_5	AACTCACTGCTGTATTGATG
MGMT_1	AAAGCCGCGCGAGCAGTGGG
MGMT_2	GAAATAAAGCTCCTGGGCAA
MGMT_3	GCCGTGGGCAACTACTCCGG
MGMT_4	TGAAATGAAACGCACCACAC
MGMT_5	ACTCTTCGATAGCCTCGGGC
MIB2_1	AAACTGTGGGACCCGAAGGG
MIB2_2	ACGCGCACCAACTACCGCGC
MIB2_3	GCAGCCCAGGAGCCTCAAGG
MIB2_4	GCTGCAAGAAGCACGGGCTG
MIB2_5	CAGGTGGACACCAAGAACCA
MIER1_1	AGGCGAAGGGTAAACAGCAA
MIER1_2	AGGGCTGAAGGCCTATGGAA

MIER1_3	CAGTACGGCAAATATGGCGG
MIER1_4	TGAGAAGCAACAGATTGTGA
MIER1_5	AAATGATGATCAGCTCCTGT
MIER2_1	CGAGCACAGCCTGTGCCCAG
MIER2_2	CGCACACACGGCCATGGCGG
MIER2_3	GAACCTCCCAGACATGACCC
MIER2_4	GGAGAAGGACTTCATCTCCC
MIER2_5	GAGTGCAGGAACTTTGAGCA
MIER3_1	ACAGTACTTACGTAACTCCA
MIER3_2	ATGAAACAGAAGCTTTGGGT
MIER3_3	ATGGACCAACATCTCAGCAG
MIER3_4	TTTCTGCAGGAACACACACA
MIER3_5	AATGCCAGTAAATCTTCTAG
MINA_1	AATGTGTACATAACTCCCGC
MINA_2	GATTTGTTGTACTTTCCCAG
MINA_3	GGCACGAGAGTACAGCGTGG
MINA_4	TGAAGAGTCTGTGCAGCCGG
MINA_5	ATCAAGCGGACACTCCTGCG
MIS12_1	GCTCTTCTAATTCTGCAAGA
MIS12_2	GTGGCGTGAAGCCAAAGAAC
MIS12_3	TAAGGAGACACCTTATAGTG
MIS12_4	TGTGGATCCAATGACCTACG
MIS12_5	ATCCGAAGCATGCACGTTTG
MIS18A_1	CATGAGCGAAGACGCGTCGG
MIS18A_2	CCGGAGCACAGGAACACCAG
MIS18A_3	GAGTCTCTTGCCCAACAGCG
MIS18A_4	TTTCAATGGCTTCAACACTG
MIS18A_5	ACAGCGAAGCAGGATGCAGT
MIS18BP1_1	ATAGTGAGAGGACAACAGAA
MIS18BP1_2	ATGAGGTATAATCTGTCCGC
MIS18BP1_3	CCGTAAGTTAAATTGTGCAG
MIS18BP1_4	TCAAAGGAGAAATCTACCCA
MIS18BP1_5	ACAATCAAAATCGTAAGTGG
MITF_1	AGAGTCTGAAGCAAGAGCAC
MITF_2	AGGGCAGAGAGCGAGTGCCC
MITF_3	ATACTGGAGGAGCTTATCGG
MITF_4	GTTAAGCGTAAGCATAGCCA
MITF_5	AAGTACCTAGTTCTTTAATG
MIXL1_1	ATAATCTCCGGCCTAGCCAA
MIXL1_2	CGTGCGCTCCAGTTTGCCGA
MIXL1_3	GAGCGCACGGGACTCGGCTG
MIXL1_4	TCTACCTCAAGAGGCAGCTG
MIXL1_5	GCCAAGTCTCGGCGTCAGAG
MKRN1_1	ATGGCGGAGGCTGCAACTCC
MKRN1_2	CGTGCAGCGCAGCAAGGACA
MKRN1_3	TCCCGAAGCGGCGCTCACTG
MKRN1_4	TTTCCAGCAACAAGGCGTGC
MKRN1_5	ACATGTCACAAGAATCTCCG

MKX_1	ACAAGGCAAAGAAACCACTC
MKX_2	ACCTCGGCCTGAGACACCGG
MKX_3	GAAGGTGAGGCACAAGCGGC
MKX_4	GGTTTGCTAATGCAAGACGT
MKX_5	AGGACGGAGGCGCCTCGGAG
MLH1_1	AGTGGTGAACCGCATCGCGG
MLH1_2	CTGGGTGAAGTACATCCTGG
MLH1_3	GGTTAATGATCCTTCTCCGG
MLH1_4	TAAGGTCTATGCCCACCAGA
MLH1_5	AATCTGTACGAACCATCTGG
MLH3_1	GATGTGACTAGAGCAAGCGC
MLH3_2	GTGATGAGACAGGATAACAG
MLH3_3	GTTGCTGTTGATGTAAGCAG
MLH3_4	TAAGACTGAAGAGAATGGCG
MLH3_5	AATGTTTCTTGGGCACGTGT
MLLT1_1	AAGAAGACCAAACCATCCCA
MLLT1_2	CTTGTACCGGAACTCCGTGG
MLLT1_3	GCGCAAGAAGCCCACCACGG
MLLT1_4	TTGAAGGCAGCCTTGGGCGG
MLLT1_5	GCTGGATGTCACATTGCTCG
MLLT10_1	GCCGATAATGTCCAATACTG
MLLT10_2	TCTGTAGTAACAGTCAAGGA
MLLT10_3	TGCCCGTCGCAATAAACCAG
MLLT10_4	TTGTGCCCTGTATATTCCAG
MLLT10_5	ACCTTATTATAACGATCATG
MLLT3_1	AACTGGTAGAGCTTCACAGA
MLLT3_2	GAACAAGTGAGTATCAGTGG
MLLT3_3	GAGGAGGCAGCCGAAGTCGC
MLLT3_4	GTGGGTGAAGCCCTCCACGG
MLLT3_5	AGCTTTCCTAGGCCAAAAAG
MLLT6_1	ACAGGTCATGCAGGCTCCCG
MLLT6_2	AGGCACGTACTGCAGCACGA
MLLT6_3	CAGTCTGGAACTCACCACCC
MLLT6_4	GCTGTGCCCACACAAAGACG
MLLT6_5	CATGGGGGTTCCAAAGATGT
MLTK_1	CCTTGGTTGGAACTTTCCCA
MLTK_2	GATATCACAGGACAAGGAGG
MLTK_3	TCTCTGGGAGATGCTAACAA
MLTK_4	TGGGCCACTGATGTAGCCAA
MLX_1	AGCGACAACAGCCTGGACCC
MLX_2	AGGAGTCCTACAAAGACCGG
MLX_3	GGAGGTGTCCACGTTACGCA
MLX_4	TTTGTAGAAAGCACCCGCAA
MLX_5	AGGTCATCATAGCCTCTCTG
MLXIP_1	GAAGGGCTACGATTTCGACA
MLXIP_2	GGATGAGGACCTCTCCAGCC
MLXIP_3	TGCCAAGTACCTCCGGCCGG
MLXIP_4	TTTGGGTTACCTATTTCCCG

MLXIP_5	GACGTCATGATCACCGCTTG
MLXIPL_1	CCTGAACAACGCCATCTGGA
MLXIPL_2	CTGGAAGCGGCGCATCGAGG
MLXIPL_3	GGACTCGGACACAGACTCGG
MLXIPL_4	TGATGCGCACCGGAAGCCGG
MLXIPL_5	AGTGTGGGGTCGATACTGCG
MN1_1	AGACGACGTGGGTGACGCCA
MN1_2	GACGTAGCAAAGTCCGGCGG
MN1_3	GCAGACGTAGCAAAGTCCGG
MN1_4	GCAGCAGTTCAGCATCTCCG
MN1_5	CAACAAGAAGAACCCGCCAG
MNAT1_1	AAGAAGTATAGCCTCCAGCA
MNAT1_2	ACAGGGTCAACATATTTCAC
MNAT1_3	TATTTGGTGGTCTTACACCG
MNAT1_4	TGAGTGGAGTACCACACTCA
MNAT1_5	GAGCCAAAAGCAGAGCAACA
MNDA_1	AAACGACCCAGTGACAGTGG
MNDA_2	TGACATCGGAAGCAAGAGGG
MNDA_3	TGATGTCGAAGACTTTCACA
MNDA_4	TGTCAGTTTGTTTCTTGCGG
MNDA_5	AAACTGACATCGGAAGCAAG
MNT_1	AGAAGATTGCCACGCAGCAG
MNT_2	TCTCAAAGCACTCTTTCAGA
MNT_3	TGAACAGAAGAAGAGGCCCG
MNT_4	TTTGCCATGACAGTAGCCGG
MNT_5	CAACATAGACGAGGATATGG
MNX1_1	AGGCAGGATCATGCCCGCGG
MNX1_2	GAAGCGGAGAAACAGAAGGG
MNX1_3	GCGGCCCAAGCGCTTCGAGG
MNX1_4	GGTCTACGGCTACTCCGCGG
MNX1_5	CCCGGTCTACGGCTACTCCG
MORC1_1	ATTGTGCTCATGGTGCCCAG
MORC1_2	GATGGATGTGGCATGAGCCC
MORC1_3	GCTCTGAATGACCACCTCGC
MORC1_4	GGAGAGCTCAACCAACTGAA
MORC1_5	AAAGCCTCAATTTATACCAG
MORC2_1	AAAGCATAAATCCTCCTCGA
MORC2_2	AAGCCGTTTCAAGACCCGTG
MORC2_3	TGGATGAAGATCCTCATCCG
MORC2_4	TTCAGCTGAAGAGAAGGCGC
MORC2_5	ACATTAGAAGTACGCCTAGG
MORC3_1	AAGAAGGCTTAATTACCCGA
MORC3_2	AAGTGGCGGAAATTACCTGA
MORC3_3	AATGGAGAAAGCATGAGCGT
MORC3_4	CTGAGAGTGACTATTCCCTG
MORC3_5	AAGTCGGGTTCTATGCGTCT
MORC4_1	CGTGCTCAGGCGGATCCCGA
MORC4_2	GAAGCTCAATGCTTACTGGA

MORC4_3	TCTAAGCCAACTCGTGGAGA
MORC4_4	TCTGGAATGTGTCCAGGCCC
MORC4_5	ACAGAAGAAAAAATGACTGG
MORF4L1_1	AAGGCGCACACTCACCCTCC
MORF4L1_2	CCTCTCACACAGCTTTCCGA
MORF4L1_3	GAAGGGAGCAGTATGCAGAG
MORF4L1_4	TTGAAGGGAGCAGTATGCAG
MORF4L1_5	AAATACTTCATACATTACAG
MORF4L2_1	AAGAGGATTTCAGCATACTG
MORF4L2_2	AAGCAGGGTTCTCAACCTCG
MORF4L2_3	ACGCTCACAGGGCTTTGCGG
MORF4L2_4	TCCTCACGGATCCTGAAGGG
MORF4L2_5	ACCATGGCTTGTTGAGGACT
MOV10_1	CCTGGAGTTAAGTATGGCGC
MOV10_2	GCAAAGAGGTGCTGACCCTG
MOV10_3	GGAACCGCACGTCGGCCCAG
MOV10_4	TGGGAAGTAGCCCACAAAGC
MOV10_5	AGCCGCCCCTCAGTGCTACG
MPHOSPH10	CAGACAAGAAACGAGAGCGA
MPHOSPH10	GAAGCACTCGGGCCGACCCG
MPHOSPH10	GACGGAAGTCGGCAAAGCCA
MPHOSPH10	TGCATCACTAACACTCACTG
MPHOSPH10	AAGGTGCAAAACAAAGGACA
MPHOSPH8_	GAGGAGGATACCGATGTCAG
MPHOSPH8_	GAGGTCGTCCTGCCCTCCGG
MPHOSPH8_	GATCCTGGACATGAAGACCG
MPHOSPH8_	GGAGCCCGAGATTCACCTGG
MPHOSPH8_	ATACATCGGATGATGATACC
MPL_1	AGCTGCTGTATGCCTACCCG
MPL_2	GGTGACCATGAAGAGGGCCC
MPL_3	TCAGCTCTGACAGCAGAGGG
MPL_4	TGGAGACTGGTCCAGAGCAG
MPL_5	ACGTATCGGGTTCCAAAGTG
MRE11A_1	ACCAACAAAGGAAGAGGCCG
MRE11A_2	CCATGGGTGAACTATCAAGA
MRE11A_3	GCAGCAGAATAATTACCTCA
MRE11A_4	TTTCAGGTGGACTATAGTGG
MRE11A_5	AATGCTGAACGGGAACGTCT
MRGBP_1	AGAGAAGAACTCCTCAGACT
MRGBP_2	AGTTCAGCCAGAACATCGGG
MRGBP_3	GAAGAGGCACACCTCCACCT
MRGBP_4	TCCAGAAGAGATCATTCAGG
MRGBP_5	AGAGGCACACCTCCACCTCG
MRI1_1	GAGACCCGGCCCTACAACCA
MRI1_2	GCAGAGCCGCTACGAGGCGG
MRI1_3	GCGGGCCATGTTGACAGCGG
MRI1_4	TAGGGCTGTACCATAGCCAG
MRI1_5	CAGAGACAACCGAAGCATTG

MSC_1	CGTAGCTGCGCTCTACGCCG
MSC_2	GCAGCGCTCCTCCTCGCCGT
MSC_3	TCTGGGCACAGCCGGCAGCG
MSC_4	TTCCAGTCCGATTTAAGCGG
MSC_5	CAAGAGGAAGCGGCCCCGTG
MSGN1_1	AAGTCAGGATGTCTGTCCAG
MSGN1_2	AGGAGCCCAAGCCATCCTCG
MSGN1_3	GAAGAATAGGATTCCAGCGA
MSGN1_4	GAGGCGGAAAGCCAGCGAGA
MSGN1_5	CGAAGGCTGCAGTGTCGGTG
MSH2_1	GGTATGTGGATTCCATACAG
MSH2_2	GGTCTTGAACACCTCCCGGG
MSH2_3	GTAATATCCAAACTGTGCAC
MSH2_4	TGAGAGGCTGCTTAATCCAC
MSH2_5	ACATCATTGAGTGTCTGCAT
MSH3_1	AAGAGCCAAAGAAATGTCTG
MSH3_2	ATCAAACACAACCTCGCCTG
MSH3_3	GCAGCCAAAGAGCAAATCAC
MSH3_4	TAAATTGCCCGACATAGAGA
MSH3_5	ATGACAGAATTCGAGTCGAA
MSH4_1	ACAAAGCTGCAACAGCTGCA
MSH4_2	GATCAGAGTGAACAACTTGG
MSH4_3	GCAGAGACCACCTGGACCGA
MSH4_4	TAAGAGCTGGACACCACAAG
MSH4_5	AAGAGCTGGACACCACAAGT
MSH5_1	AATTCCACAGCACACACAGA
MSH5_2	GAACCCAAGGAGGACACCGC
MSH5_3	GCACTCGTAACAACAGACTG
MSH5_4	GGACCAATCTCACTGTGAGG
MSH5_5	ACGTCCGACTCATGACCTGG
MSH6_1	AAACCAGACAAGGCCACCAG
MSH6_2	AAGGCGAAGAACCTCAACGG
MSH6_3	GATCTGTAGGATCATTACCA
MSH6_4	TCAAAGGAAGCCCAGAAGGG
MSH6_5	ATCACCACCTCCACTAACGT
MSL1_1	AATTAAACTGGAGTGCCAGC
MSL1_2	AATTCAGGAATCTGAGCCTG
MSL1_3	ACTGAGTGGTCCCTCCAAGA
MSL1_4	GGGACCCAGCACCCATCCCA
MSL1_5	CCACTCAGTAGAGCCTCTAA
MSL2_1	ACTGCAGATATCAATGCCTG
MSL2_2	CCTATAGCAACTGTTCCCAA
MSL2_3	TTATCCGAGGCCCAACACTG
MSL2_4	TTGGAAGCCACTGTATCCAA
MSL2_5	ACTGCCAATAGCAATGCTGA
MSL3_1	CAACGGGAGAGTGTAATCAA
MSL3_2	GAAGAGCTTGGGCATGCTGG
MSL3_3	GAAGATGATCTGCTATGCAC

MSL3_4	GAGCAAATGAGCGCGAGCGA
MSL3_5	ATTGTTACTACATTAACAGG
MST1_1	AATGACTTCCAAGTGCTCCG
MST1_2	GCGGCCATGGTGCTACACTA
MST1_3	GGTGCTGAAGATCCCAGCGC
MST1_4	TGTAGCACCAAGGACCTCCG
MST1_5	ACTCGCGCCCTGACTCCGTG
MSX1_1	ACAAGACGAACCGTAAGCCG
MSX1_2	GGCCGACCACAGGAAGCCGG
MSX1_3	GTAGAGCGAGGCACCCGCCG
MSX1_4	TGGCCGACCACAGGAAGCCG
MSX1_5	CGGACGCGCCCTCTTCGCCG
MSX2_1	GAAGCCCTTCGAGACCGCCT
MSX2_2	GGCGCGCACTCACTTGGCGG
MSX2_3	GGGACTCTATGCCACGCCAG
MSX2_4	TATATGGACGCTGCCTGCAG
MSX2_5	ATTCAGAAGATGGAGCGGCG
MTA1_1	AGAAACTAAAGCACCAGCTG
MTA1_2	GACGGCCAATGGGAACGTGG
MTA1_3	GAGAATCGAGGAGCTCAACA
MTA1_4	GATAAAGGAGAGATTCGAGT
MTA1_5	ACAGACGGCCAATGGGAACG
MTA2_1	AAGAGGAATCAAAGCAGCCA
MTA2_2	ATGCTGGCAAGTGACTTCCA
MTA2_3	GAACGGCTACGACCTGGCTA
MTA2_4	TACTAGAAATGTCCCTGCGC
MTA2_5	CTGCTGAATATACCGGTCTG
MTA3_1	AGTGTCTTAGTACCACTCGG
MTA3_2	GACACGTGACTTCTAACACG
MTA3_3	GGACAGGAGAATCAGATGAG
MTA3_4	TGGGACATTCGCCAGAGCCC
MTA3_5	AAAACACTATTAGCTGACAA
MTERF_1	ACTATTGGAGAAGGTACGAG
MTERF_2	ATGCTTGAATCCAGAACCCG
MTERF_3	CATTGGTCAACAATCGACAA
MTERF_4	TAGGATGATTACCAATGAGC
MTF1_1	AAATTTAGGTGCGATCACGA
MTF1_2	ACCCACCAGAAGACTCACCG
MTF1_3	GCACATTCGAACTCATACAG
MTF1_4	TTGGAGGATGAAGATGACGA
MTF1_5	AATGCACTTCCACAACACAA
MTF2_1	AATGTCCTTCCAGAGAACCC
MTF2_2	AGTGCTGCAAATGTAAGCAG
MTF2_3	GCAGACATTACCCTATAGTG
MTF2_4	TCTAAACAGAGACTCTACAG
MTF2_5	AACTGGGATAGATTGCACCC
MTR_1	AGAGCACATGTTCATCCGGT
MTR_2	CAGGCCAAAGGACTTCTGGA

MTR_3	CTAAACGAAGAACACTTCCG
MTR_4	GAATTAAGAGGTTTGTGGCA
MTR_5	CACCTGCATTGGGATAACAG
MUL1_1	GCCGCCGTCATGGAGAGCGG
MUL1_2	GGAGAAGTGATACATACCAA
MUL1_3	GTACACGGAGTACAGGGCGG
MUL1_4	TCCGGAAGCAGTATCTGCAG
MUL1_5	ACATCCACGCCATCCTCGTG
MUM1_1	AAAGCGATTATGGCCTGCGA
MUM1_2	GAGGACTTCAACCAGGACAT
MUM1_3	GCAAGTGTGCTATACATCGA
MUM1_4	GCAGTTCCAGACCTCACAGG
MUM1_5	AAATACGCTCTTACCTAACG
MUM1L1_1	AATGCTAGTGGACAAAGCCA
MUM1L1_2	ATTCCCTTGAACAACTCCCA
MUM1L1_3	TAAATCACAAGCACCCACAA
MUM1L1_4	TGGAAGCAATGACTTGTCAG
MUM1L1_5	AAAGTATCAGACGAAAAGAG
MXD1_1	AATAGCCCTCATCACTGCCG
MXD1_2	CCGGTGCAGAATGGCGGCGG
MXD1_3	CGGCCGACTATCTGGAGCGG
MXD1_4	GGAGCGCTCCGACTCCGACA
MXD1_5	ACAGAGATGCCTTAAAACGG
MXD3_1	ACGGCGCAGCAGGCTCAGCG
MXD3_2	GACCTGGATGTTGCTGGCCA
MXD3_3	GAGCACAGCTACTCGCACGG
MXD3_4	GAGCGCTCAGACTCAGACCA
MXD3_5	CCAGGATGCACATCCAGGTG
MXD4_1	AACTGGAGGAGCAGGACCGC
MXD4_2	GAGCCTCCTGAAGCGGGCCA
MXD4_3	GAGGGCACGGGCCTACGAGA
MXD4_4	GCCGTCGAAGGGCAGCACCG
MXD4_5	ACAACGAGCTAGAAAAGCAC
MXI1_1	CAACACCAGCGAGAACTCGA
MXI1_2	CGGGCACAGAAACACAGCAG
MXI1_3	GAAAGCACAGAGTTCTCCCA
MXI1_4	GGAGATGGAACGAATACGAA
MXI1_5	CGGCATGGACGGGAATGAAG
MYB_1	ACTGGAATTCTACAATGCGT
MYB_2	ATGTGCAGTGCCAGCACCGA
MYB_3	GAAATCGCAAAGCTACTGCC
MYB_4	GAAGCAGCCCATCATAGTCA
MYB_5	ACCAGGCACACAAGAGACTG
MYBBP1A_1	AGAGGGCGAGCACTCCAAAC
MYBBP1A_2	ATACTCCAGCAGCTTCTCCG
MYBBP1A_3	ATTGCAGGAGATCCTGCCGG
MYBBP1A_4	GCACCACCTCACCTGTGCCA
MYBBP1A_5	ACCAGCACCTCTACCCACGG

MYBL1_1	ACCCACAAAGTTCCTGGCCG
MYBL1_2	CCAGGCAGCGAACTTAGGGA
MYBL1_3	GTGACACATACTGATACCCA
MYBL1_4	TGGCGAAGAGGTCGCGCAGG
MYBL1_5	AATTCTCATTAATTTAACTG
MYBL2_1	GCACAACCACCTCAACCCTG
MYBL2_2	GGTCAAATGGACCCATGAGG
MYBL2_3	TAAGGCAGGCTCGTTTCTGG
MYBL2_4	TTGAATCCAGACCTTGTCAA
MYBL2_5	CAAGGGGCCATGGACCAAAG
MYC_1	AGGGCGAGCAGAGCCCGGAG
MYC_2	CGAGGACGGAGAGAAGGCGC
MYC_3	GAAGGGTGTGACCGCAACGT
MYC_4	GCTGCACCGAGTCGTAGTCG
MYC_5	AGAGTGCATCGACCCCTCGG
MYCBP_1	CGAAGCGTGAGCAGTTCCGG
MYCBP_2	GAGGTACTTGGAGAAGTCGG
MYCBP_3	GTGCTGGACACGCTGACCAA
MYCBP_4	TCAGTATGAACCACCTCAGG
MYCBP_5	AGAGCTGCTTCGCCTAGAAC
MYCL_1	GAGGCTGGGAGTGCAGTCCG
MYCL_2	GATGTGGAAATGCTTCATGC
MYCL_3	GCGCCAAGAATCGCGAACGC
MYCL_4	GCTGGGCGAACCCAAGACCC
MYCL_5	CATCTGGAAGAAATTCGAGC
MYCN_1	AAAGAATGAGAAGGCCGCCA
MYCN_2	GAGGGAGTGGACATACTCAG
MYCN_3	GCTGTAGCGAGTCAAACTCG
MYCN_4	GGACTGGGCGGTGGAACCGG
MYCN_5	CCCCGACTCGACCCCCCCGG
MYEF2_1	AAAGACAAGAATTCGGGCGC
MYEF2_2	ACAGGAGGATCATTTCCAGG
MYEF2_3	GCATAGCTGGAACTGTGAAG
MYEF2_4	GGCGGACGCCAACAAGGCCG
MYEF2_5	AATTGTGGAACCAAGTCTAC
MYF5_1	AGTCCACCACCATGGATCGG
MYF5_2	ATTCCTGAGGATCTCCACCT
MYF5_3	CAGCCACTATGCGCGAGCGG
MYF5_4	TGAGGCAGTGACCAGCCTGG
MYF5_5	CCACGACCAACCCCAACCAG
MYF6_1	AGTGTTTCCGATCATTCCAG
MYF6_2	CGAGGAAATACTGTCCACGA
MYF6_3	GCTCCGCAGAATCTCCACCT
MYF6_4	GTGTGGAGGCTGCAGGCCCG
MYF6_5	AAGGCTCTCCTTTGTATCCA
MYH9_1	AAACTTCATCAACAATCCGC
MYH9_2	AAATACCGCTTCCTGTCCAA
MYH9_3	GAAGGAGCGGAACACTGACC

MYH9_4	TACAGGAGTATGATGCAAGG
MYH9_5	AATGTGACCGATTTCACCAG
MYNN_1	AACTCTAGACTCCAGTGCAG
MYNN_2	AGAGAGACTGAACAAACAGC
MYNN_3	GACAGCAAATGCCTTCCCAC
MYNN_4	TTATGAGGCGGAGAACTCCG
MYNN_5	ACTCGAGTTGACATCAGTTG
MYO1B_1	AGCTGAGTCTGTCTTGGCGG
MYO1B_2	GCAGGAGGAGTATATACGGG
MYO1B_3	TTCGGCCAAAGTGAATGGAG
MYO1B_4	TTTAAAGGCGATCCACTAGG
MYO1B_5	AGTACTACATCTTACCCGCA
MYO1C_1	GATTGACAGAGACCAGGACG
MYO1C_2	GCTGGAGAACTTCACCAGCG
MYO1C_3	TCGGTACACAGTGTCCGCCA
MYO1C_4	TGCTGGAGTTATCGTTCCGG
MYO1C_5	AGTGTTTCAGCATAACAGGT
MYO3A_1	AATTCTGCTGACCATAGCTC
MYO3A_2	AGAATCTGACCACATTAGGG
MYO3A_3	ATTGGCATCCATCAATGCAT
MYO3A_4	TCTGGGTCTTTACTCCACAA
MYO3A_5	CACCGTCGGAACACATCCGT
MYOD1_1	CGAAGGCGCCTACTACAACG
MYOD1_2	CGTCGAGCAATCCAAACCAG
MYOD1_3	GCAGCCTCTTGCCTGCGCGG
MYOD1_4	GCATGGTGGCGGCCTTGCGG
MYOD1_5	AGGTCCGGGGAGTCGAAACA
MYOG_1	CATCGTGGACAGCATCACAG
MYOG_2	GAGGCGCTCGATGTACTGGA
MYOG_3	GGAGTTCAGCGCCAACCCAG
MYOG_4	TAAGAGGAAGTCGGTGTCCG
MYOG_5	CCCTGCTCAACCCCAACCAG
MYPOP_1	GGAGAAAGCGTCCTCCGCGG
MYPOP_2	GTTGTCGGAAGACCGCCGGG
MYPOP_3	GTTTGGCCAGTCCCTGTCGA
MYPOP_4	TGGAGGCAGGGAGTCAACTG
MYPOP_5	ATCACCAGCTGGAAGCGCAC
MYRF_1	AGGCATCGCAGGCTGCCCGT
MYRF_2	CCTGGAGGAGTACATCAGCA
MYRF_3	GCAGCGGGATCTGTACATGA
MYRF_4	GGGCAGGCGGGACATGGAGG
MYRF_5	ACACCACCGAGCAATTGAAG
MYSM1_1	ACTGAAGCCTCTAACAGCCA
MYSM1_2	CAAGCTCAGAAATTACACTG
MYSM1_3	GACGTAGGGTCCGAGACCCA
MYSM1_4	GAGGCGGATGTGGATATCGA
MYSM1_5	ATGGTGTTATCCAAGGTCCA
MYT1_1	ACAGGCTCAGGGCACGTCCG

MYT1_2	AGAGCCGTCACAGCCGGGCG
MYT1_3	GAGCCGTCACAGCCGGGCGT
MYT1_4	GCCAGAGCCGTCACAGCCCG
MYT1_5	AGAGTAGCTCCAATTCCGAT
MYT1L_1	CCAGATGGAGGTGGACACCG
MYT1L_2	GAAAGCAGACAGCTCCTCAG
MYT1L_3	GACGGCAGTGGTCATGTCAG
MYT1L_4	GCTGGAGAAAGCCATCGCTT
MYT1L_5	ATATGAATTACGTCATGTTG
MZF1_1	AGATCCAGGCCCGTGTGCAG
MZF1_2	GAGCGTAGGCCGCTGGCGGA
MZF1_3	GCTGCGTGAGCGTAGGCCGC
MZF1_4	GGTGGGTACAGACTCCTGGG
MZF1_5	CACTGTGATTTCAGAGATGT
NAA10_1	AGGCTGTGAAGCGTTCCCAC
NAA10_2	ATTCAGAAGCTGCCCACCTG
NAA10_3	GTTGGAATAGAGGTGCAGGG
NAA10_4	TATGAACATCCGCAATGCGA
NAA10_5	AATGAGGTGATATGTCCATG
NAA15_1	GATGCTGCAGATGTTTATAG
NAA15_2	GATGGATGAGGCCCAGGCCT
NAA15_3	GCCTGGACTAAATATCCCAG
NAA15_4	TTGGGCGGGAGGCTCACGGC
NAA15_5	GAAATATTAAAGAAGCTGCA
NAA60_1	AATCCGCTCCACTTACTCGA
NAA60_2	ACAGGTGTGAATGACAGAGG
NAA60_3	CAGGTGCTGGATGTAGTCCG
NAA60_4	TTTGCAGTGGTCCTGGGCGG
NAA60_5	AGCTACTATCATTCCCACAA
NAB1_1	AGAGACTGCAGCATGCCGAG
NAB1_2	GATGCCTTTATCCAACAAGG
NAB1_3	TGATGATCCACACAAAGAGG
NAB1_4	TGTGCAAACACTCTTCCAGC
NAB1_5	GAAACATCTCACACTTCATG
NAB2_1	AGTGCCCATGCCCACAGCTG
NAB2_2	GCCGTTTCGACTCTAAGCGG
NAB2_3	GTCTGGGCTGCAGGGTCCGG
NAB2_4	TCACCTGGAGCCCTTCAAGG
NAB2_5	AGGTCTCATAGTAGGAAAGG
NACC1_1	GATGCGATTTCGGGACTCGG
NACC1_2	GATGGCCAAGTTCTCCACGC
NACC1_3	GGCACAAGGTCCTACTGCGG
NACC1_4	TGGAGCACGCGGCTGTCCAG
NACC1_5	AGCCCGATGTCTGCGCCACG
NANOG_1	AGCCTACCTGTTTGTAGCTG
NANOG_2	TCAAAGCAAGGCAAGCTTTG
NANOG_3	GAATAGCAATGGTGTGACGC
NANOG_4	GGCCCACAAATCACAGGCAT

NANOG_5	GGTTCACCAGGCATCCCTGG
NAP1L1_1	CAATTTCGAAGTCTGCAGCA
NAP1L1_2	GACCATCAAGTCTTTCTTGA
NAP1L1_3	GGTCTGGTAGAAACACCAAC
NAP1L1_4	TCTAACCTTTCAGCCTGCCT
NAP1L1_5	CACCAACAGGATACATTGAA
NAP1L2_1	GAATATGTGGATGAGGACGA
NAP1L2_2	GCGGTGAAGAAGCTGCCGCT
NAP1L2_3	TAAAGAAGAGGAGGATCCTA
NAP1L2_4	TGCGGTGAAGAAGCTGCCGC
NAP1L2_5	AAAAGGTGGCGATACTGATG
NAP1L3_1	AAGCAACAGAGGCTAAGGCA
NAP1L3_2	ATCCGACAAGAACTTCAGAA
NAP1L3_3	TAAGGGAGGCATTTCACTAG
NAP1L3_4	TCCACGAAATTTGTTCCCAA
NAP1L3_5	ACAAGCCTCTGTATGATAGG
NAP1L4_1	AAGATGGTAAACCAGAACTC
NAP1L4_2	GAATTAGAAGGTGACGAGGA
NAP1L4_3	GCTGAAGAGCCAGATCCCAA
NAP1L4_4	TGAAGTACAGCACAGCCCGC
NAP1L4_5	CGATGTTGAACCAACAGATG
NASP_1	GGAGAGACAGCTAATGAGTG
NASP_2	GTCGGCAGAAACCAGCTCCG
NASP_3	GTTGATACCAAGGTAGCCCA
NASP_4	TGGGAACCTAGAGCTTGCCT
NASP_5	GAACAGGTTTATGACGCCAT
NAT10_1	ACTGTAGAAACAGTGGAAGG
NAT10_2	CTGCAGCACACACATGCCGA
NAT10_3	GACGAGACACTTCTTACAAG
NAT10_4	GGTGTTATAAGAAAGAGCTG
NAT10_5	GAGTGCAACAGTACTCCTCA
NAT6_1	CAAGAGCTGACTCTGAGCCC
NAT6_2	GCGGGCATGGCCCACCACAA
NAT6_3	GGATCCTGAACACCAGCCAG
NAT6_4	TGAGGCGGCGGCCAAAGCCA
NAT6_5	AGGGTAAGCTCAGTTGGACC
NBN_1	CAAGAAGAGCATGCAACCAA
NBN_2	CCAGGACCAAGCCTTTCACA
NBN_3	GTGGAAACTGCTGCCCGCCG
NBN_4	TTTGCAGGACTCCTTTACAG
NBN_5	AGAATGCACTCACCTTGTCA
NCBP2_1	ACTGGCACAGAACCAGTGAG
NCBP2_2	GAGGGCAGGCAATACGGCCG
NCBP2_3	GCTGCGCAGCGACTCCTACG
NCBP2_4	GTACTGGCTCAGCTCCACGT
NCBP2_5	AAAAGCTGTACGTTATATGT
NCL_1	AACGACCACCTTCTTTGCTG
NCL_2	CATCATGGTGAAGCTCGCGA

NCL_3	GAAAGCCAAGAACGTGGCTG
NCL_4	TGAAGACCTGGAGAAAGCGT
NCL_5	AAGAGATTGAAAGCCGTAGT
NCOA1_1	ACTGCATTACTTCATAACGC
NCOA1_2	AGACATGCAACCTTTCATCA
NCOA1_3	GTCTACAGCATACTGCACGT
NCOA1_4	TAAGATGGTTCTCTGCCCTG
NCOA1_5	GAGATACTAGGATTGACCGA
NCOA2_1	AGGGCAGGGTGTCATCGACA
NCOA2_2	GCTAAGAGGCATCATCATGA
NCOA2_3	GGTCTGGCGAACCTCCGAGG
NCOA2_4	TGTGTTCAAGATGAGTGGGA
NCOA2_5	AGTGCATAGTTACTACCCTG
NCOA3_1	AAGCCGATGTATCTTCTACA
NCOA3_2	CCTGTAACAAATGATCGACA
NCOA3_3	TCAGCCACGAGCTATGATGG
NCOA3_4	TTTCGTGAATCACTGGCCAG
NCOA3_5	CAACGAGAATCGATATACTG
NCOA4_1	ACAGACCTGACTGTTCTCCA
NCOA4_2	AGAGATCACAAACTGCAGGG
NCOA4_3	TCAGGCACTTCAGATTGCCC
NCOA4_4	TGAGGTGTAGTGATGCACGG
NCOA4_5	AGATTGGCTAGTGACTCCCC
NCOA5_1	ACAGAGATAGCTTTGATGGA
NCOA5_2	ATTTCTGGCCACCAGCACCA
NCOA5_3	GAAGGTGCGAGACCTGGGCA
NCOA5_4	TGTGGTCCCGCAAGTCTCGG
NCOA5_5	GCCTTGGAGGATGTTAGCAG
NCOA6_1	AACAATGGAAGACTCAGAGA
NCOA6_2	GATGACACATTTCCACCCGG
NCOA6_3	GTGGGAAGGCGTCTGTACCG
NCOA6_4	TGTGAGAATGAATGGACCCA
NCOA6_5	ACTGTTATGATACCCCCGGG
NCOR1_1	GAAACGCAGAGGCAGAAACC
NCOR1_2	GATGAGAGGAACGATAATCA
NCOR1_3	TAACCAGCCATCAGATACCA
NCOR1_4	TTAGTGCACCCGCTGCCAGA
NCOR1_5	AAATAATTTGGACAATACTG
NCOR2_1	AGAAGGCTGAAGCTGCACAT
NCOR2_2	CAGGCAGCAGCTCTAGCCGA
NCOR2_3	CGTGGACCGAGAGATCACCA
NCOR2_4	GCACTGGGTAGGAAAGGCTG
NCOR2_5	AGGGATCCCTCGGTCCTACG
NDC80_1	AAGCGCAGTTCAGTTTCCAG
NDC80_2	AAGCTCCATGTACACAGTGG
NDC80_3	AGCAAAGAGAAACCAACCTT
NDC80_4	CTTCAGGAAGTCTTTAACAG
NDC80_5	GGATCCCGGAATAGTCAACT

NDN_1	CGTGCGGAAGCTCATCACTG
NDN_2	GAACGACGAGGGCGACCCGA
NDN_3	GATGCGCAGCACGTTCCAGA
NDN_4	GCTGCGAGGGTAGTGGGCAG
NDN_5	AGAAGGCGCACGAGCTCATG
NDUFAF5_1	ACAACCAAGATCCAAAGCAA
NDUFAF5_2	GTTGCGGAACCTCTTTCAGC
NDUFAF5_3	TCTGGGCGAGGTGCTACCGC
NDUFAF5_4	TTAGCGGAAACGGAAAGGGA
NDUFAF5_5	AGAAAATACATTTGACCTGG
NEDD4L_1	ACAACAACCGGACCACTCAG
NEDD4L_2	ACAGACAGAAGATCCAACCA
NEDD4L_3	ATGAGACTGTCCTTACCGGA
NEDD4L_4	GTACCTCCATGTCATCCCTC
NEDD4L_5	AAGTTACTGTTGGCGAGCTG
NEK6_1	ACAACCTCTGCCACACCCTG
NEK6_2	AGGCCGAGGACAGTTCAGCG
NEK6_3	GCTGGAGTTGGCTGACGCAG
NEK6_4	GGCGAGGCAGGACTGTGTCA
NEK6_5	GCAGCGAAAAGACAGCGTGT
NEK9_1	CGTGCAAGGAATTCGGGCCA
NEK9_2	GACTTTGGGAGCGAGTCCGG
NEK9_3	TCTGCAGTAGGTCTCTGCTC
NEK9_4	TTCCAGCTCAATCAGCAGCG
NEK9_5	AGTGAAGTCTATGTTTGGGG
NELFA_1	AACCCAAGAGCGCCACGCTG
NELFA_2	GCAGTTGAAGCGGAGCGCCG
NELFA_3	GTGGCTGCACAACAAGCTGG
NELFA_4	TCGGCGACCATGAGCACCCA
NELFA_5	CGTGGAGGGAAGGTAGCTCG
NELFB_1	CGTGGGTGAAATCTGCACCG
NELFB_2	GACTGGTGGAACTCCAGCCG
NELFB_3	GATGCCGTCCCTGCAGCCCG
NELFB_4	GCTGTATCGAGCCTGCGCCG
NELFB_5	ATGTGCACTACTGCACGCTG
NELFCD_1	AAATAGAATCTGCTTTGCGG
NELFCD_2	AGAAGGAGAGGATGATGCGG
NELFCD_3	AGATGATTGCACATACCACG
NELFCD_4	GCCGGGCGCCATCATGGACG
NELFCD_5	CAACTTACAAGTTCAACCGG
NELFE_1	AAGAATCCCATTCTTACCGA
NELFE_2	ATGACACCCACCCTTCTCCG
NELFE_3	GGAAGTTCCAACCTCAGCAA
NELFE_4	TCATACAGAGATTTCCTCTG
NELFE_5	AGAGGAAATCTCTGTATGAG
NEUROD1_1	CTTGCAAAGCGTCTGAACGA
NEUROD1_2	GCTGCGCTGTAGGCGTGCGG
NEUROD1_3	GGGAAGGAAGCGCTGGCCGT

NEUROD1_4	TTGCCATTGATGCTGAGCGG
NEUROD1_5	CCGCGTTCAGTCCGTGCATG
NEUROD2_1	ACGAGCCGAAGACTAGCCCG
NEUROD2_2	AGAAGGACTGGACGAGGCGG
NEUROD2_3	GACGGAGGCCACGTTGGCCG
NEUROD2_4	TTGCCATTGAGACAGAGCGG
NEUROD2_5	ACGGCGAAGACGACGAGCCG
NEUROD4_1	CTATAGGAACATCATAACGG
NEUROD4_2	TAAGGAGGGCTAGGAAGCCC
NEUROD4_3	TATAGGAACATCATAACGGG
NEUROD4_4	TCAGCCCACAAGCAACCTGG
NEUROD4_5	AGTCAACACACCATCCTGGA
NEUROD6_1	GAACATGGCACCCTGCCCAA
NEUROD6_2	GTAAGGGAAGTGGCTGTCGG
NEUROD6_3	TGAACATGGCACCCTGCCCA
NEUROD6_4	TTATCAAGAGTCCCATGCCC
NEUROD6_5	AACGCCAGGAGTTTCCTGAT
NEUROG1_1	ACGACGAGCAGGAGAGGCGG
NEUROG1_2	GCGGTAGGTGAAGTCTTCGG
NEUROG1_3	GTAGGTGAAGTCTTCGGAGG
NEUROG1_4	TGTGGAGCAAGTCTTTGGGC
NEUROG1_5	AGCAGCGGCAGTGACCTATC
NEUROG2_1	CGCCTGGCGGATCACTGCGG
NEUROG2_2	GGACACGGAGGAGGACGGCG
NEUROG2_3	GGTGGAATTGGAGGACACGG
NEUROG2_4	GTTGGTGCAACTCCACGTGG
NEUROG2_5	CAAGAAGACCCGTAGACTGA
NEUROG3_1	AGGTGGGCAGGACACCGCGC
NEUROG3_2	GGACCACAGCTTGTACGCGC
NEUROG3_3	TCGCTCCTCCAGCGACGCGG
NEUROG3_4	TTGGACAGTGGGCGCACCCG
NEUROG3_5	CAGCCGGCCTAAGAGCGAGT
NFAT5_1	AGAGCCCTGTAACACAGCAA
NFAT5_2	GAGTGAAGATGTTACTCCAA
NFAT5_3	GGAGAGCTGCTCCTCAGCCG
NFAT5_4	TACCTGACTGAGGGCAGCCG
NFAT5_5	ATATGTTGGTCATGATAGGG
NFATC1_1	CCACCGAGCCCACTACGAGA
NFATC1_2	GTTCACGTCCCGCAACCCAG
NFATC1_3	TCGTCGGTCACGCTGACCCG
NFATC1_4	TCTCCTGTAGAATTCTCTGG
NFATC1_5	CACGAGGTTATCTCGATGCG
NFATC2_1	AGTGGGCAACACCAAAGTCC
NFATC2_2	GAGTATTTGAATCCGAACGA
NFATC2_3	GATCCGAGGGCTCAGGCCCG
NFATC2_4	GCTGTCAAAGCTCCAACTGG
NFATC2_5	ACATTGGAAGAAAGAACACG
NFATC3_1	ACTAAGAGAAGACTCCCGGT

NFATC3_2	GAAGTTCTTGAGCAGACCGC
NFATC3_3	GTACACATCCCACAGCCCAG
NFATC3_4	TATGAAACTGAAGGTAGCCG
NFATC3_5	ACTGCTGGGTTATGATATGG
NFATC4_1	AATATGAGCAGCTGGAGCTG
NFATC4_2	GCTGCCTGAGAACAACATGG
NFATC4_3	TAGGTGCAGCGCCATAGGGA
NFATC4_4	TGGCGGGTAGGAGAGATGGG
NFATC4_5	CCACATAGTCAAAGGGACCA
NFE2_1	AATCTGGGTGGATTGAGCAG
NFE2_2	ATATGTAGAGATGTACCCAG
NFE2_3	GGGCTAAGCCCACTGCACGG
NFE2_4	TGTTGCCTGATTCATCCCGA
NFE2_5	AAGTTGACAATCTTGTCCGT
NFE2L1_1	ACAATTACTTCACTGCCCGG
NFE2L1_2	AGGGCGCGGAAGCTCTGGCA
NFE2L1_3	GATGTGACAGGCCCAGACAA
NFE2L1_4	GTATGCGCTCCAGTACGCCG
NFE2L1_5	CATCTAACAGACCCCCCAAA
NFE2L2_1	ATTCTGCTGTGCTTTCAGGG
NFE2L2_2	CATACCGTCTAAATCAACAG
NFE2L2_3	GCGACGGAAAGAGTATGAGC
NFE2L2_4	TATTTGACTTCAGTCAGCGA
NFE2L2_5	CACATCCAGTCAGAAACCAG
NFE2L3_1	AAGCGGTGGTGGTCGGCCGG
NFE2L3_2	CGATGAAGCACCTGAAGCGG
NFE2L3_3	GGAAGAGGAGAAGGCACCCG
NFE2L3_4	GGAATTAGACTTGATGACAG
NFE2L3_5	CTTCGTCCCTCGCACCAGCG
NFIA_1	AAAGCGCCTCAAGTCTGTGG
NFIA_2	CCTGTCATTACAGGACCCAG
NFIA_3	TGAGTCCAGGAGCAATGAGG
NFIA_4	TGTAGGCAAAGGCTCGGACG
NFIA_5	AATCCTCTCGATATTCGGGT
NFIB_1	ATACCTGGAGTTGCACACAG
NFIB_2	GATTGGATAAGACACAGCAC
NFIB_3	TCCTCAGCGCCCATAACCCA
NFIB_4	TGGAACACATATTTACCTGG
NFIB_5	ACCAAGTATAGGCAATTGCA
NFIC_1	ACAGGACCCAACTTCTCCCT
NFIC_2	AGTGACGCTGAACACGCCGG
NFIC_3	CCTGTTCAAGGGCATCCCGC
NFIC_4	GGACAGCAAGCCCATCACGC
NFIC_5	ACCTCGCTCACGCACGAAGT
NFIL3_1	AGGGAGCCAAGAGATGACCG
NFIL3_2	GATAGCAAACTTATCTGCAG
NFIL3_3	GGAATTAGAGAGCTACACAA
NFIL3_4	GGACGAGCACGAACCCTCGA

NFIL3_5	ACAAATCTTCTGCATGTCGG
NFIX_1	GACATCAAACCACTGCCCAA
NFIX_2	GCTGGCAGCAGCCAGTCCAG
NFIX_3	GGCGCAGGCAGTCAATCCGC
NFIX_4	GGGCCACTGGGCTTACCTGG
NFIX_5	AGGCCGGGGTTCGAGCACTG
NFKB1_1	ACAGCTGGATGTGTGACTGG
NFKB1_2	ACTGGAAGCACGAATGACAG
NFKB1_3	CAAGGAGATGGACCTCAGCG
NFKB1_4	GAACAAGAAGTCTTACCCTC
NFKB1_5	AAGTAGGAAATCCATAGTGT
NFKB2_1	AAGACAGCAGGCTCTGTGCG
NFKB2_2	ACTGCCCAGAGGCCGAGCAG
NFKB2_3	ATCCTGGCTCACCTTGACAG
NFKB2_4	GGGACCAGCCAAGATCGAGG
NFKB2_5	ACTCGACTACGGCGTCACCG
NFKBIE_1	GAAGGAGTGAGTCAAGGCCC
NFKBIE_2	GCAACAGAGCAGCACCGCTG
NFKBIE_3	GCTCTGGCCGCCCTTCCAGC
NFKBIE_4	TGGTTGGTTCTTCTGAAGGG
NFKBIE_5	GCTGTACATCTGGACCAACC
NFKBIZ_1	AAGCTGCTGGACGACAGCCG
NFKBIZ_2	AGGAAAGTGCCCTTCTCCCT
NFKBIZ_3	GAAGTCCTGACACTGCTCTG
NFKBIZ_4	TCGGGTAAAGAACTCAGTGA
NFKBIZ_5	AAAGAGGTTTGGTTCATAAG
NFRKB_1	GAATTTGCTTCAGCAGCCGA
NFRKB_2	GCAGGACTAGGAGAACGTGC
NFRKB_3	TCTGCCCGAGGACCTTCTGG
NFRKB_4	TGAAGGGCGTTTCTGCCGGA
NFRKB_5	AACCAGCTAAGTCTAGCTCG
NFX1_1	AAGAGAAATGCACTGTACGG
NFX1_2	GCAAGGTAAAGAATCCTGAG
NFX1_3	GCTCTGACAACTCCACACTG
NFX1_4	TCTGCGGCACGGGATGGCGG
NFX1_5	CTACATTTATGTGTGACAAG
NFXL1_1	AGAAAGATTGTCCCTGGCCT
NFXL1_2	GAAGTTGTGCAAGTCCACTA
NFXL1_3	GCGGGAGCCATTCTCACCGC
NFXL1_4	TGTTCACAGCGTTGTCACCG
NFXL1_5	AGTAAACATTTATGGCCACA
NFYA_1	GATGGCACCATTCTCCAGCA
NFYA_2	GCAGTTGCAGACTGAGGCCC
NFYA_3	GGACAGATCCAGATCCAGGG
NFYA_4	GTTTGCTGTATACTGCTCCA
NFYA_5	ATTAACTGGTTGATAGACGA
NFYB_1	ACTAAGTGAAGAGCTTACAG
NFYB_2	ATCTCTGCAGACTATATTGG

NFYB_3	GGAGCAGTCACAGCTACAGA
NFYB_4	TTAGCTAACCAGTTACCAGC
NFYB_5	AATGATCATGAAGACACAAA
NFYC_1	ACAGAGAAATGATATCGCCA
NFYC_2	AGCGCCTGTACTCTTTGCCA
NFYC_3	AGTCCAGTACTATTTCACGC
NFYC_4	TCAGCCAACCAGCCCTCCGA
NFYC_5	ACACAGAAGATAACAAGCGC
NGDN_1	ATTCTGGACAAAGCCTCAGG
NGDN_2	GCAGTACTCAGATGCTCCAG
NGDN_3	TAACTATGAGGAGAGCATGA
NGDN_4	TGTACTGGAACCAAGCGTGG
NGDN_5	GAGACTGGTGGAGATTCGCA
NHLH1_1	CACGCGAGAACGCATCCGCG
NHLH1_2	CAGGTAGGAGATATAGCAGA
NHLH1_3	GAGTTCAGACGTCCAGCACG
NHLH1_4	GATGCTCAACTCAGACACCA
NHLH1_5	GAGTCGGGCTTCAGTGACTG
NHLH2_1	CAATTTGCGGAGCTCGGCGA
NHLH2_2	CCTGGGCGGCACGGACACCA
NHLH2_3	CGTGTCGGACCTGGAGCCGG
NHLH2_4	GAGATAGGAGATGTAGCAGA
NHLH2_5	AGATTCGGACCATCCCAGCT
NIN_1	CGAGTTCCAAGAGTCCGTGG
NIN_2	GAACAGCCAGGAGATCCTGA
NIN_3	TAAACCATACTGCTCACAGA
NIN_4	TCAGGACAACCTCTTGGGCA
NIN_5	ACAGCTAATGATGTTATGTG
NIPBL_1	AAGTGAGGACTACCTACACA
NIPBL_2	ACACAGGCATGACAATAGGA
NIPBL_3	ACTGGCTGTGGAGATCTCAA
NIPBL_4	ATTGAGCAGCATTTAGTGGG
NIPBL_5	CTTTAGCTTGATATGCAACG
NKRF_1	AAAGAGCAGCATAAGCGGGA
NKRF_2	AATTCAGGGCCGCTCAGCAG
NKRF_3	GTGCTGTCCAAACCTTCCAA
NKRF_4	TTGAAGTTAGAGTTGTCCGG
NKRF_5	AAATGTACAGGCTCAAAACG
NKX1-2_1	ACGGCGCGAAAGGGCTCCCG
NKX1-2_2	AGCCCAACTGCGCCAAGCCG
NKX1-2_3	ATGCTGGCATGGCAGGACGG
NKX1-2_4	GCCGCCTTGCATCTTACCAG
NKX1-2_5	AGGATGCGGAGGATCCGAGG
NKX2-1_1	AGACGGCAAACCGTGCCAGG
NKX2-1_2	CCAGCTCTCGCACTCCGCCG
NKX2-1_3	GGCCATGCAGCAGCACGCCG
NKX2-1_4	GGTGCCGTAGTCCGAGCCCG
NKX2-1_5	CAAGCAACAGAAGTACCTGT

NKX2-2_1	CAAGGCGCAGACCTACGAGC
NKX2-2_2	GAGGAGGGCTCTGTGGCCGA
NKX2-2_3	GGCAGCCGCCACCTTCCAGG
NKX2-2_4	GGTCCGGAGGAAGAGAACGA
NKX2-2_5	AATGACAAGGAGACCCCGGG
NKX2-3_1	CATGGCAGTGGTCGCCGCGG
NKX2-3_2	CTATGGCTGTGCGTACCCGG
NKX2-3_3	GGAACATGAAGAGGAGCCCG
NKX2-3_4	GGACCGTGTGGACATAGCCC
NKX2-3_5	AAATTGCGTCCCCTCAGCGG
NKX2-4_1	AGAAGGCTGCATGCCCGCCA
NKX2-4_2	ATGGGCAGCTACTGCAACGG
NKX2-4_3	CAAGATGAAACGGCAGGCCA
NKX2-4_4	CCAGCACAGGCACCGCCACG
NKX2-4_5	CATGGCGGGTCACAACGCGG
NKX2-5_1	GCGATGGCAAGCCATGCCTA
NKX2-5_2	GCGGGCAGAGAGCTCTCCGG
NKX2-5_3	GGCACGTGGATAGAAGGCGG
NKX2-5_4	GTGCGCGCTGCAGAAGGCGG
NKX2-5_5	ACCGGCGCTACAAGTGCAAG
NKX2-6_1	ACGGGCACAGCTACTCGGCG
NKX2-6_2	AGAATGGACGCAGAGCCGCG
NKX2-6_3	AGCGCGGGCTTCGGACACGG
NKX2-6_4	GAATGGACGCAGAGCCGCGA
NKX2-6_5	GCCTGCGAAAAGAGCACGCG
NKX2-8_1	AAGCGCCGAGCCGGGACCGA
NKX2-8_2	AATCCAGCCAGGCGGCGCAG
NKX2-8_3	GCGCTCCGAATCCAGCCAGG
NKX2-8_4	GTGCCGGTGCTTGTTCGCGA
NKX2-8_5	AGGACGCGCAACACCTGCCG
NKX3-1_1	ATCCAGGACATCCTGCGGGA
NKX3-1_2	GAAAGAGGAGGCCTTCTCCC
NKX3-1_3	GAGGAAGGACGTGAGCGGCT
NKX3-1_4	TAAGCGAAAGCAGCTCTCCT
NKX3-1_5	AGCGGCTTGGACGGGGTCGG
NKX3-2_1	AACAAGAAAGAGGAGCGCGG
NKX3-2_2	ACAGCAGACAGCGGGAGCCG
NKX3-2_3	GTCGCTACAAGACAAAGCGC
NKX3-2_4	TACGGCCACCTTCTTGGCGG
NKX3-2_5	CCGGTACCAGAACAGCTGCG
NKX6-1_1	CGGACAGCAGATCTTCGCCC
NKX6-1_2	GGAGACAGAGCGCCTCAAGG
NKX6-1_3	GTTGTGGGTGCCCAGAGGCG
NKX6-1_4	TGGCCGTGGGATGTTAGCGG
NKX6-1_5	CGGGTGGTAGTACTCACGAG
NKX6-2_1	CAAACCCTCGAACTTGGCGC
NKX6-2_2	GGGCCAGCAGATCTTCGCGC
NKX6-2_3	GGGCCCGAAGTAAACGCCGG

NKX6-2_4	TCAGCACGAACGCGCCCGGG
NKX6-2_5	ATCTGCTGGCCCGAGAAGGT
NKX6-3_1	CCTGAGCAAAGCCTAGACCC
NKX6-3_2	CGACGAGTACAACAAGCCGC
NKX6-3_3	GTGGCGGAAGAAGAGCGCCC
NKX6-3_4	TGAGCACCGAGAAGGCGGCG
NKX6-3_5	AGAACCGCAGGACCAAGTGG
NMI_1	AGGCGGAGAGGTGGACCGCG
NMI_2	CAACGGGCAAAGAATGGAGG
NMI_3	GCTTGAAACGGAGTTACAAG
NMI_4	TCAAAGTCCCGAAATGGAGG
NMI_5	ACTGTCTGTCATAGTCCACG
NNMT_1	ACACACATAGGTCACCACTG
NNMT_2	GCTGAGCACGCAGTCAGCCG
NNMT_3	GGAATCAGGCTTCACCTCCA
NNMT_4	TAAAGGATTCACAAGCAGAG
NNMT_5	ACTAATCCAGACGGTGTGAA
NOBOX_1	CCGAGAGATTGCCCAGACGG
NOBOX_2	GATGGCTTCAAAGCCCAGGA
NOBOX_3	GGAAGATTGTTCCCTCCCAG
NOBOX_4	TGAGGGTCTCCAGAGCACAA
NOBOX_5	CCAGCGCATCATGGTAAAGG
NOC2L_1	CAGAGGCACGGCCTTTACGC
NOC2L_2	GCAGCTGTGGCCACCACCCG
NOC2L_3	GCTGCGGCACATCAGCGTGC
NOC2L_4	GGAGACACGGGAAGCACGCG
NOC2L_5	AGCTGTGGCCACCACCCGAG
NOL6_1	ACACTCACAGTGCCAAGAGG
NOL6_2	AGAGGCAGACCAGCCTGAGG
NOL6_3	GCAACAGTGAGGGTTTCAGG
NOL6_4	GGCCTGGTGGAAGTCAGCCA
NOL6_5	GATTGATGCCTTCCTACGGG
NONO_1	AGTGGACCGCAACATCAAGG
NONO_2	GCCGTGAAGAAGAGATGCGG
NONO_3	GGCGCCAAGAAGAACTTCGG
NONO_4	TCTTCCTCCCGACATCACTG
NONO_5	CTGGACAATATGCCACTCCG
NOTCH1_1	GAACGCCGGGACATGCCACG
NOTCH1_2	TCCTCGCCCTGCAAGAACGG
NOTCH1_3	TCCTGCCAGAACACCCACGG
NOTCH1_4	TGAAGCGGCCAATGGCACGG
NOTCH1_5	TCGCACGCCTCCTCGATCAG
NOTCH2_1	AAGTTCTGCTTACTCACCCA
NOTCH2_2	CTTCTCTTGCATGTGCCCAG
NOTCH2_3	GCAGCGCCCACAGCAGAGCG
NOTCH2_4	TTGATGACTGCCCTAACCAC
NOTCH2_5	CATTGGTGGATACAGATGCG
NOTCH3_1	AGACACCACGGCCAGCACAG

NOTCH3_2	GAGGTCGCCACTCTGCCTGC
NOTCH3_3	GGACACCGATGTCTCAATGG
NOTCH3_4	GTGGCGGCGACATCGGGCGA
NOTCH3_5	CACGCTGTGTGATCGCAACG
NOTO_1	GCTGCTGGAAGGCTCATCCA
NOTO_2	GGTCGGACCCGAGAGCCCGA
NOTO_3	TGGACGCAGGCGGAGCCCGA
NOTO_4	TTGGAGCTGGCTCACTGCTC
NOTO_5	CAGCGGTCCAGAAGGCCGGG
NOVA1_1	AAGAGGACCAATACGGGCGG
NOVA1_2	GATGCGATCTGGATTAACGG
NOVA1_3	GCAGAACGGGACCCACACTG
NOVA1_4	GTTGGTGCAATACTTGGCAA
NOVA1_5	AGAGGACCAATACGGGCGGT
NPAS1_1	GACTTCGGAGACCAGCGCTG
NPAS1_2	GGCGAACACAAAGCCATCCA
NPAS1_3	GGTGACGCTGAGGCGCACGA
NPAS1_4	TCAAGGCGCCGTCCGGACCG
NPAS1_5	CAGGTGCTGCTCGAAGACTT
NPAS2_1	ACAAGTCTGAGAAGAAGCGT
NPAS2_2	ACAGTATCACGCCTCTCCTT
NPAS2_3	GGGTGAATTCTTCATTACTG
NPAS2_4	GTGGTAGTAGTCATAGCCTG
NPAS2_5	ATTATCGCAGTGACAACAGA
NPAS3_1	AAGTTATAGGTGCACAGCGA
NPAS3_2	ACGCTCAAGAGTGTTGTCAG
NPAS3_3	GATGCCCTGGACGTTCTGCG
NPAS3_4	GATGCTGCTCGCTCCCGCCG
NPAS3_5	CTTCCCTACGATATCTACAG
NPAS4_1	CAGGTGGACCTGGTTGCCCA
NPAS4_2	GGTCATGTACCGCTCCACCA
NPAS4_3	GTAGAGCCAGGTCTTTAGCA
NPAS4_4	TCTGCTTGTGTTCACAGCCG
NPAS4_5	GAGAGTGTGAGCGAGCATCT
NPM1_1	TCTCCCTTCTAGGTTTCCCT
NPM1_2	CAAAGATTATCACTTTAAGG
NPM1_3	TCTTAAGTATATCTGGAAAG
NPM1_4	TTCAACCTTAAGACCACTGG
NPM1_5	TTGTTCACAGGTCAGTTTAG
NPM2_1	AGAAAGCTGCACTCCTACCA
NPM2_2	AGAAGCATCAGACCTAACCT
NPM2_3	CAAAGAGGAGATGCATCGCG
NPM2_4	GCAGTGACGACCGTGCTCTG
NPM2_5	CCGGAGCTGGAAAGTAACTG
NR0B1_1	AGAGGATGCTGCCCTGCCGT
NR0B1_2	GGTAAAGACCACCCACGGCA
NR0B1_3	GGTGAAGACCACCCGCGGCA
NR0B1_4	TGGTGAAGACCACCCGCGGC

NR0B1_5	GAGCACAAATCAAGCGCAGG
NR0B2_1	CATGAGCACCAGCCAACCAG
NR0B2_2	GAAGTGCGTAGAGAATGGCG
NR0B2_3	GGTCGGAATGGACTTGAGGG
NR0B2_4	TGAGGAGGACACGGGTCAGG
NR0B2_5	ACCTCATCGCACCTGCCGGG
NR1D1_1	GTTGCGATTGATGCGGACGA
NR1D1_2	TACGGTGTGCACGCCTGCGA
NR1D1_3	TGGCCACTTGTAGACTCCCA
NR1D1_4	TGGTGAAGACATGACGACCC
NR1D1_5	GAGTCTACAAGTGGCCATGG
NR1D2_1	AAAGGAAGAAGTGATTGGCA
NR1D2_2	CAAGCAAATCGAGTGCACCT
NR1D2_3	GTGGCGTCAGGATTCCACTA
NR1D2_4	TGAGAGAGATCTCTGAACCC
NR1D2_5	AGTTACCTGTGCAACACTGG
NR1H2_1	AATGATCCAGCAGTTGGTGG
NR1H2_2	AGAGTCACTCACCAGGCAGG
NR1H2_3	AGGAGGAGGGTCCGGAGCCG
NR1H2_4	CACGTTGTAGTGGAAGCCGG
NR1H2_5	CACAGACACGGCAAAGCTCG
NR1H3_1	AACATTGTAGTGGAAGCCCG
NR1H3_2	GAAACTGAAGCGGCAAGAGG
NR1H3_3	GATCGCAGAGGTCTTCAGCA
NR1H3_4	TTGGAGCTCAGACTCTGCGG
NR1H3_5	ACCAGATCCCCATAGCCGGG
NR1H4_1	AGTGTCAAGAGTGTCGACTA
NR1H4_2	ATGACATCAGCATCTCAGCG
NR1H4_3	CCAGCTGAGACTCTCTACCA
NR1H4_4	GCATGGGCGCGTCAGCAGGG
NR1H4_5	AATGGCAACCAATCATGTAC
NR1I2_1	CAGGACTCACCACTTCAAGG
NR1I2_2	CGAGGGAAGAAGCTGCCAAG
NR1I2_3	GATCATGTCCGACGAGGCCG
NR1I2_4	TGAAACGCAACGCCCGGCTG
NR1I2_5	AGGTTGACATGTCAGCCATG
NR1I3_1	AATCCTAGTATCTCCCACAG
NR1I3_2	GAGCAAAGCAGGCCCAGCGG
NR1I3_3	TCTGCGAAGTGTGTGACCAG
NR1I3_4	TGTAGCGAAGAGGCCCGCAG
NR1I3_5	GTGAAGTCAGCAAGACTCAG
NR2C1_1	ACAGATAATTCTCCAGACCA
NR2C1_2	GCACTTGATCATAATACCCA
NR2C1_3	TATGGAGCAGTAACTTGTGA
NR2C1_4	TATTGAACAACAGATGGGAG
NR2C1_5	CCTGAATGTGCACTACATTG
NR2C2_1	AAACAGGTCTTCTTGATCCA
NR2C2_2	AATCCAGATAGTCACCGCAG

NR2C2_3	GCAGATGGGATAGACACCAG
NR2C2_4	TAATAGGCTGAAACAAGCCA
NR2C2_5	CCAGTCGACACCCATCATTG
NR2E1_1	AAGGCCGCTGACCTTGGGCG
NR2E1_2	GGTCGCCACACACTTTGCAG
NR2E1_3	GTTCCAGGGAGGCTGTCCGG
NR2E1_4	TGATTCACACACCGACTCCG
NR2E1_5	CGGTGTCCACCACTCCAGAG
NR2E3_1	CATGAAGAGTAGGCGAGCCG
NR2E3_2	GAGACCACTGGATGGCCCCG
NR2E3_3	TCTTCAAGAGGAGCGTACGG
NR2E3_4	TGGAGTCCAACACTGAGTCC
NR2E3_5	GTAGGTACCTGATCCCGGAA
NR2F1_1	ACCTGACGTGAACAGCACGA
NR2F1_2	CGGGCCCAAAGATATGGCAA
NR2F1_3	GAAGAGCTGCTCGATGACGG
NR2F1_4	TCTTCAAGAGGAGCGTCCGC
NR2F1_5	ATGTGTAAGTTAAGTTCCTG
NR2F2_1	AAAGGCGACCACCCGGTCGG
NR2F2_2	GAGCCGGGCACCTCGTCCTG
NR2F2_3	GGTCAGCGCGAACTGCCCGT
NR2F2_4	TCTTCAAGCGCAGCGTGCGG
NR2F2_5	ACATTTGCGAACTGGCCGCG
NR2F6_1	AACGGCGTGGACAAGGCGGG
NR2F6_2	GCGGCGGAGACCTCTTCCCG
NR2F6_3	GCTATGGCCATGGTGACCGG
NR2F6_4	TCTCAAGAAGTGCTTCCGGG
NR2F6_5	AAGTCGAGCGGCAAGCATTA
NR3C1_1	AAAGCCATTGTCAAGAGGGA
NR3C1_2	AAAGTGATGGGAAATGACCT
NR3C1_3	CCTGAGCAAGCACACTGCTG
NR3C1_4	GATGGAGGAGAGCTTACATC
NR3C1_5	ATCAACAGGTCTGATCTCCA
NR3C2_1	AACCAGTGCTGTGTTGACCG
NR3C2_2	CAGCGGGCAGTCACTTCCGG
NR3C2_3	GCCATGTATGAACTATGCCA
NR3C2_4	TGAAGGCAGCGGATTCCCAG
NR3C2_5	AGAATCCATATATAAACCCA
NR4A1_1	GCTGGGCGAGAAGTGGCCGA
NR4A1_2	GGCTAACAAGGACTGCCCTG
NR4A1_3	GTGACAGCTCAGCAAAGCCA
NR4A1_4	TGTCCGAACAGACAGCCTGA
NR4A1_5	GTCCAGGTGTGCACGGACCA
NR4A2_1	CATCTGGCAACTAGACACCG
NR4A2_2	GCACTGATCAGACTCACCGG
NR4A2_3	GTTGGAGGAGAATTCAACAA
NR4A2_4	TTGTCAATTATTGCTGGCGG
NR4A2_5	AGCCGGGTTGGAGTCGACAT

NR4A3_1	ACTGAGGAAGATCTGCACCC
NR4A3_2	GAAGAGCGGGAAGCGCGCGC
NR4A3_3	GCTCGAGTAGCCCTCCACGA
NR4A3_4	GTACAGATAGTCTGAAAGGG
NR4A3_5	CCTGCGTGTACCAAATGCAG
NR5A1_1	AAGGTGTCCGGCTACCACTA
NR5A1_2	GCAGGGCTTCTTCAAGCGCA
NR5A1_3	GGTAGCCAGCCAGTGGCCCG
NR5A1_4	GTTCGACCACATCTACCGCC
NR5A1_5	ACGTTGGGCCCTCCAGAGAA
NR5A2_1	AAAGCCCTCATCCGAGCCAA
NR5A2_2	AAAGGAAGGATCCATCTTCC
NR5A2_3	ATGCGATCGAGCCAGTCCCA
NR5A2_4	CATTGTGTAGTCCAGCAGGG
NR5A2_5	AGGGCCGACCGAATGCGTGG
NR6A1_1	CATAGTGCAAGCCTGTAGCG
NR6A1_2	GAATTGGCAGAGCTTGACCC
NR6A1_3	GACGAACCGCCGCCTAGCGG
NR6A1_4	GGAGGCCGGAATAAGAGCAT
NR6A1_5	ACGAACCTGTCTCATTTGTG
NRARP_1	AAGGCGAAGTACGCGGCCAG
NRARP_2	GACGCAGCGCATCTTCCAGG
NRARP_3	GCTGGCCGCGTACTTCGCCT
NRARP_4	TGTGCGCAAGGGCAACACGC
NRARP_5	CTGCACCAGTCGGTCATCGA
NRF1_1	AAAGCTGCAAGCCCATCTGG
NRF1_2	CTGACAGTAGAACTTCATGG
NRF1_3	GCAGCAGCGGCCATTCCCAC
NRF1_4	TGACTTACCACATTCTCCAA
NRF1_5	AAGATGAGCTATACTATGTG
NRG1_1	CAAGAAGCCGGAGTCCGCGG
NRG1_2	CAGAACTGGTTTCACACCGA
NRG1_3	CATGATGCCGACCACAAGGA
NRG1_4	TACAAGATGGCTTGTCCCAG
NRG1_5	ACTTGCACAAGTATCTCGAG
NRIP1_1	AACCAACAGGTCCTGAACCA
NRIP1_2	AATCTTGCAGCACATGACAA
NRIP1_3	GAAGAAGGAATGATACCCAT
NRIP1_4	GGATTGAGTGAAGTTATCCA
NRIP1_5	AGCAACACTAGGTTTAGGTG
NRL_1	AGCCCAGTGAGGCTGTAGGG
NRL_2	GAACGGCTCAGAGGAAGAGG
NRL_3	GGGAGCTAAACCGGCAGCTG
NRL_4	GTAAAGCGGGAACCCTCTGA
NRL_5	AGGCACTGAGCTGTAAGGTG
NSD1_1	AAATCCTGTAGTCTCCGCAG
NSD1_2	AAGCACATAAAGATGAACGG
NSD1_3	CTATCGGCAGTACTACGTGG

NSD1_4	GAGGAAAGCCTTCTAGCCCG
NSD1_5	GAATTGCTAGTTAAAACGCC
NSL1_1	CCAGTGGATGTACAACAGGG
NSL1_2	GAGTGGCGGAGACCAAGGCC
NSL1_3	GGTCCAGTGGATGTACAACA
NSL1_4	GGTGCGCTGCACCTCGAAGC
NSL1_5	CCGCTCTGCGAGATGCGCAG
NSMCE2_1	ACTGGTTTCATCTCCTTCAG
NSMCE2_2	AGAGAAGCTGACGGAACAGA
NSMCE2_3	GGACTCAATCATGCGAACAA
NSMCE2_4	GTGTGGCCACACCTATGAAG
NSMCE2_5	AGCAGACTCTACACCACTGA
NSUN2_1	GAGCTCAAGATCGTGCCCGA
NSUN2_2	GCTGCGGATTGCAACACGCG
NSUN2_3	TAAGGAATTGGAGGACCTGG
NSUN2_4	TAGCTGCAAGCTATTTAAGG
NSUN2_5	ATGTGTCTAATGAACTGCCA
NSUN3_1	CCTGTAGGATAAGTCAAAGG
NSUN3_2	GAGCCCACATTCCTGGCCAG
NSUN3_3	GAGGTGGCTAAGGCAGACGT
NSUN3_4	TTGGCTCTGGAATTAAGGGA
NSUN3_5	CAGCTTCGATCATTTGAACA
NSUN4_1	CCTCAGACTGCAGTTCCCAG
NSUN4_2	TCTTATATCGATGTCTCCGC
NSUN4_3	TGTAGTCTGGCTATTCGGGA
NSUN4_4	TGTCATGGAGTACTACCTGA
NSUN4_5	GCAAAGCCAGTCGAACAGCA
NSUN5_1	ATGACATGGGAGCCTGGCGG
NSUN5_2	GAGCCGCCAGGGCTCTATCA
NSUN5_3	GATCACAGCATCCAGCACGG
NSUN5_4	GATGACTTACGAGCCCTCAA
NSUN5_5	ATGGTAGCGTGGATCCGAGG
NSUN6_1	AAATGAAGAACAGGTTGCCT
NSUN6_2	AGCCGCAAAGAAATCTTCAG
NSUN6_3	GTGATGCTGACACAATTCCT
NSUN6_4	TTAGGGAGAAGTTATAGCAC
NSUN6_5	CATGTACTAAATCCTCAACC
NSUN7_1	AAGGTTGTCCTTTATTCCCA
NSUN7_2	ACTTCCCTGCCTCTGTCCGG
NSUN7_3	GAGCACAGTGGAAGAACAGG
NSUN7_4	TGGAGTACAATCACAAGCTA
NSUN7_5	AGTTAGGAAACAGGAACTAA
NTMT1_1	CATCAAAGACAACATGGCCC
NTMT1_2	CTGGAAACAAATCCCACCCA
NTMT1_3	GAAACAAATCCCACCCACGG
NTMT1_4	TCAAGCCAAGACCTACCTGG
NTMT1_5	AACCCTCACCTATCACCCAC
NUDT16_1	GCAACACGCGCCAAGGACCA

NUDT16_2	GCGTGTCCACGAATCCGCCG
NUDT16_3	TAGAAGTGGGCCACAACGCG
NUDT16_4	TAGGCGAGGCCCTGGCGCTG
NUDT16_5	CAGACGCTTGGCATAGAAGT
NUDT16L1_1	CAGTGCCAAGATGTCGACGG
NUDT16L1_2	CTGAGCTCGCACCTGACCGA
NUDT16L1_3	GCTGAAGCAGATCAGCCGGG
NUDT16L1_4	GGTGCTCAACATGATGCCCG
NUDT16L1_5	CCTGAGCTCGCACCTGACCG
NUDT21_1	AATGAGGAGGACTGTAGAAG
NUDT21_2	CCTGGTGGTGAACTTAACCC
NUDT21_3	GAATGAGGAGGACTGTAGAA
NUDT21_4	GATGGTGCGCTCCAGGGTGA
NUDT21_5	ACTAAAACGCTTAATGACAG
NUF2_1	AAGATCTTAACAGGAGCTGA
NUF2_2	AAGGGAGAAACTGAAGTCCC
NUF2_3	GCTTTGGAGAAATACCACGA
NUF2_4	GTTAAACGCCGCACACCAGG
NUF2_5	ACAGTTAAACGCCGCACACC
NUFIP1_1	ACAACATTCTGTGATACCCA
NUFIP1_2	ATTTCGAGACTCCTATCGGG
NUFIP1_3	GAAGCTAGAAGGTCCACCGG
NUFIP1_4	GATCAAGTTAGACACTCCAG
NUFIP1_5	CTGAGAATCAAGGGGAGACT
OARD1_1	GAGGATTGTCGCATGGGCGC
OARD1_2	GTGGTCTTGATCGTCTGCAA
OARD1_3	TATCAGTGAGGATTGTCGCA
OARD1_4	TGTATCTGCGATGATCGAGG
OARD1_5	AGCCTTAATGAAGATCCAGA
OGT_1	ACTGTGTTCGCAGTGACCTG
OGT_2	GCTCCAGATGGCGTCTTCCG
OGT_3	GTAGCAGCGGGTGACATGGA
OGT_4	GTGCCAACTCAGCTAACCCT
OGT_5	CTATACACTTCCAGTGTCGA
OIP5_1	ACCGCGACAGGTCCCAGGCG
OIP5_2	CATGGAGTGGGATACGCAGG
OIP5_3	GAGGGACCGCGACAGGTCCC
OIP5_4	GCACTCACTGGAGAAGACCA
OIP5_5	CCACAAAAGTCCCCCCGGGG
OLIG1_1	AGAGCGAACTGGCCGCACGG
OLIG1_2	AGGGCAGGATGACCTCGCGC
OLIG1_3	GGAGAATTGCGCCTGCACGG
OLIG1_4	GTGGCCGCAGCATGGTCCCG
OLIG1_5	CGCAACTACATCCTACTGCT
OLIG2_1	AGAGGCGCTGGACACAGCCG
OLIG2_2	CGTACGGCATGACCTCGCGG
OLIG2_3	GCACGACCTCAACATCGCCA
OLIG2_4	GGACGGATGGAGCCGACCGA

OLIG2_5	CATCCTGGGGACAAGCTAGG
OLIG3_1	GATGGCGGGAAGTGAGGCGG
OLIG3_2	GGGAGGCCGGATGGTGCCGA
OLIG3_3	GTACTTGCTGCTCTCTCCCG
OLIG3_4	TATGATGCAGAAGATGCCCG
OLIG3_5	CAAAATCAAGAAGCAGCTGT
ONECUT1_1	CAGCAGGGACGCCATGCCCA
ONECUT1_2	GAAGGAGGAGTCTGGACAAG
ONECUT1_3	GATGGGCACCATGCCGGCCG
ONECUT1_4	GCATCCACAACTCCCAGCAA
ONECUT1_5	AGAGATCAATACCAAAGAGG
ONECUT2_1	ATGGCCTCGATCCTGGACGG
ONECUT2_2	GAGGTCAGTGAACACCAGGC
ONECUT2_3	GCAAGACGATCTGAGCACAG
ONECUT2_4	GGTGAGCAACACCTGTCCCG
ONECUT2_5	CAGCAGAGTCTGCCCAACTA
ONECUT3_1	CAAGTTCCACCAGCACGCGG
ONECUT3_2	GAAAGTGGCCGTGGCGCCGG
ONECUT3_3	GGAGAAAGTGGCCGTGGCGC
ONECUT3_4	GGAGATCAACACCAAGGAGG
ONECUT3_5	CCTCTACGGACCCTACGGCA
ORC1_1	AGAAACGTGCTCGAGTACAG
ORC1_2	AGGTGAGGTGATCTCCGAGA
ORC1_3	AGTTTGGCGAGATTTGGAGG
ORC1_4	TCAAGACAGAACTCTTTCTG
ORC1_5	AACTCCTTGACCATACCGGA
ORC2_1	AGTAAGGGAGCTAAGTGGCA
ORC2_2	CAGAAGAGAGTTCTCATAGG
ORC2_3	TACTGAGAGGAGCATTGAGG
ORC2_4	TTCAGACAAGGTTCAACCGA
ORC2_5	AAAAATGGCTCTGCTACAGG
OSR1_1	ACTTTGGAGAAAGAAGAGCG
OSR1_2	GAAGCGCGCATCCACCAAGC
OSR1_3	GATCCGGCCAAGCTCGGTCG
OSR1_4	TGAATGACATGAGGGAACCA
OSR1_5	AAGCCCGAGATCACCGCTGG
OSR2_1	GAACACCTTCCCGGCCACGG
OSR2_2	GGCGAAACTGTGATCTGCGG
OSR2_3	TCTCTAGAAGTCCTGCCGCG
OSR2_4	TGACTTTGCCAATTTGGCGG
OSR2_5	AGGGGAAGCGCGCGTCCACG
OSTF1_1	AGAGTGTTTGGACAACAGAG
OSTF1_2	AGCATTGGTAGCCATGTCGA
OSTF1_3	GGAAAGGCACCTCCAAAGGC
OSTF1_4	TCGAACATTAAGCAATGCCG
OSTF1_5	AAATATTGAACTGAACCAGC
OTP_1	CAACTCGTTGAGCTGTGCGG
OTP_2	CAGGCTGTCGCCCATGGCGG

OTP_3	TACACTTCACCGCCTCCCGG
OTP_4	TGTGCTCTAGCGCCTTGCGG
OTP_5	AGGCCTGCCTCAGTTCCCGT
OTX1_1	GAAGTTGAGTTTCCAGGCGG
OTX1_2	GGACCTCCTGCACCCATCCG
OTX1_3	GGGACTAGGTGGGAACCCGG
OTX1_4	TGGGTGCAGGAGGTCCATGG
OTX1_5	CAGCTGTGAACGCGTGAAGG
OTX2_1	ATTGAAGTTAAGCTTCCAGG
OTX2_2	CTTACCCGGGTAGCCCACGG
OTX2_3	TACCCGGGTAGCCCACGGAG
OTX2_4	TCTCCCGGCGCTGTTTCCGG
OTX2_5	AAATCAACTTGCCCGAGTCG
OVOL1_1	CAAGTGGCCCATGTCTTCAG
OVOL1_2	CATGTTCAAAGCCAGCGGGC
OVOL1_3	GCAGCAGCGGGCTGTCAGGG
OVOL1_4	GCTCCAGTTCCTCTTGCACG
OVOL1_5	CGTCGTTGTGACACTTCATG
OVOL2_1	AAACTGGCAGCCCTTCTGCA
OVOL2_2	AGGCACGTGCAGCGACTCGG
OVOL2_3	CCGGGAGCTCATCCCAGCTG
OVOL2_4	GTCGCTGCGGCAGTCCTCGG
OVOL2_5	ACGACACCTTCGACCTGAAG
OVOL3_1	CAGGGCGCGGTGCTGTGCGT
OVOL3_2	CATGTGGCGCTTGAGATCGA
OVOL3_3	GAAAGCCCTTGCCACAACAG
OVOL3_4	GTTTACGCAGCGCTGCTCCC
OVOL3_5	CTGGGATATAGGCATCTCCC
PA2G4_1	ACAGGAGCAAACTATCGCTG
PA2G4_2	GGGTTGGCACCTACTTCTGC
PA2G4_3	GTACCTTAAAGTAAACGGCA
PA2G4_4	TAGTAGGAAGATGTCGGGCG
PA2G4_5	CAAGGACCGAAGTACCCCTG
PABPN1_1	ACTGGTCGAGGGTGACCCGG
PABPN1_2	AGTCTGAGCGGCGATGGCGG
PABPN1_3	GAGGAGCCGGGACTGGTCGA
PABPN1_4	GGGCTCCCGAACCAGGCCCA
PABPN1_5	GCCGGAGCCCGAGCCCGAAG
PADI1_1	CAGTGAGGTAAAGCACGCTG
PADI1_2	CCAGCGACATCAGCCAGCTG
PADI1_3	GCAAGGTGAAGAGGAGCCAA
PADI1_4	TGTGAAAGAGCCCATAGGCA
PADI1_5	GCAGACATGGTCGTATCTGT
PADI2_1	CAACCATGCCAACTCACCAA
PADI2_2	CATGAGCCAGGCGAGCACCG
PADI2_3	GCTGCAGTACGGGAGCCGCG
PADI2_4	GGAGAAGAACAACCCAAAGA
PADI2_5	CACCGTCAACTACTATGACG

PADI3_1	AGCGTGCAGACACCAGGCGG
PADI3_2	GCGACCTGAACTGTGAGGGA
PADI3_3	GTAGAGCACCGCATAGGCCA
PADI3_4	TTACCCATAAATGTCCACGA
PADI3_5	ACGTGTTGTTCCTCACACTA
PADI4_1	GAAGGACGTGCAGTCCTCAG
PADI4_2	GATGAAGTGCTTGACAGCGA
PADI4_3	GCACAACATGGACTTCTACG
PADI4_4	GCTAGCCCGACGATGGCCCA
PADI4_5	AGCTCTACTCTACCTCACCG
PAF1_1	ACCTCTCAGGCAGAGTCCGG
PAF1_2	ACCTGTGGCCATCCTCCCGC
PAF1_3	GAAACGAAAGCGGGACCAGG
PAF1_4	TGTGAAGCAGCAGTTTACCG
PAF1_5	ACCATTGGGGTCGATGCGGT
PAG1_1	AAGAAGCCGCGACAGCATAG
PAG1_2	AGCATAGAGATCATTACAGG
PAG1_3	GAAGCCGCGACAGCATAGTG
PAG1_4	TGTGAAAGAGATCAAGGAGG
PAG1_5	AACTGTGAAAGAGATCAAGG
PAGR1_1	AGACACTGCGGCCAGTACGG
PAGR1_2	AGCCCGGAGCCAGAAACGGG
PAGR1_3	CTGGCCGTGGAGGATACCGG
PAGR1_4	TCCCAGACCACACATGCCCA
PAGR1_5	GTGCGTGCCCTGCAGCGACG
PAK2_1	ACTGCGTTTGCTCTGCTCAG
PAK2_2	ATGGTGCTGCTCATTCGCAC
PAK2_3	GGTCATGGAATACCTTGCTG
PAK2_4	TATGCCACTCTCTTACCAGG
PAK2_5	GTGTGCTCAAAATCAGATGG
PARG_1	AAGTGAACCCGAGTCACCGA
PARG_2	CACACAGTAACTATTCGGGT
PARG_3	CACTAAAGGAATCAAGACAG
PARG_4	CAGCAAGTCTTGCGATCCTG
PARG_5	AGGAGATGAGAAGAATGCCT
PARP1_1	AAGAAGACAGCGGAAGCTGG
PARP1_2	AGCTAGGCATGATTGACCGC
PARP1_3	CAAGCAGCAAGTGCCTTCTG
PARP1_4	GACACAGACACCCAACCGGA
PARP1_5	CGATGCCTATTACTGCACTG
PARP10_1	AAACCGCCGACGCTCTGGAG
PARP10_2	AAGGAGGCAACAGTGCCCAG
PARP10_3	AATCCCTCTACCCTGCACCA
PARP10_4	GGACATGCTGCTCCAAGCGC
PARP10_5	AAGTCAGCTCGATGCCCATG
PARP11_1	AAGCAAATGAATCTCACCAC
PARP11_2	ATGGTGTCAGCTTGCAACAG
PARP11_3	TATTAAGCGCTGCTTTCCAG

PARP11_4	TGGACACGTCAGATACCCAG
PARP11_5	AGCTTCAAAACAAACCCTTG
PARP12_1	GCAGCGTGGGCGCTTCGTGG
PARP12_2	TCCGGCCTGGAACTTCAAGG
PARP12_3	TTTGAAAGGCACAAGAGCCG
PARP12_4	TTTGGTGACAGAGGAGGCCG
PARP12_5	ATAGCTCATTATAGCTCAGG
PARP14_1	AGAGAAGCACTTCTGCCCAG
PARP14_2	CCTGGTGTTCTTCTACCCGG
PARP14_3	GGAAGTGACAAACACAATCA
PARP14_4	TGTCTAATCCACCATCAAGA
PARP14_5	AACGGAGTTGACAACAACAT
PARP16_1	AAAGAACTTCTCCAGTCCTC
PARP16_2	AAAGTCCTTACAGTCGCCGC
PARP16_3	CAAGTGCCAAACCAAGAAGA
PARP16_4	GCACTGCCATCTGAACAAGG
PARP16_5	AAAGATCCAAAAGCTGACTG
PARP2_1	AGGACAGAAGACAAGCAAGA
PARP2_2	GGACCCAGAGTGTACAGCCA
PARP2_3	TATTAGAAGATGATGCCCAG
PARP2_4	TCAGCGTTCGAATTCCATGG
PARP2_5	AATACCAAGAAAGCCCCACT
PARP3_1	ACTTATCGAAGTACAGGCAG
PARP3_2	AGGGCCCTCAGTCTGTACCC
PARP3_3	GAGCAAGCAACAGATTGCAC
PARP3_4	GGAGATGTTCAAGAACACCA
PARP3_5	AGCAAGCAACAGATTGCACG
PARP4_1	AGTAGAGATGATTTGGGCAG
PARP4_2	ATGACACTTGGAAATACCTG
PARP4_3	GATATCCTCACACTTCCTCC
PARP4_4	GGAGGTGGTGTGATGTCCAG
PARP4_5	CACACCAACTTACATCGTTG
PARP6_1	ACACAATAGGTTCTGTCCGG
PARP6_2	CTGGAATGATGACGACTCGG
PARP6_3	GGGCAGTCAGTTCTCACCAT
PARP6_4	TGAAGTAGAGTCCCACCTGA
PARP6_5	AGGAATCCATACTCCAGAGT
PARP8_1	AAACGCATAAGCTGCTGCGG
PARP8_2	AAGACATCAACAGTGGGCAC
PARP8_3	ACAGTGGAGGAAGATTCTGA
PARP8_4	TGTGCAGGAGGAGATTGCTG
PARP8_5	AGCTCCCGTTCACATACGGT
PARP9_1	AGGCATTGACACCTACCGCA
PARP9_2	ATCACAGAAATTATCAGCCC
PARP9_3	TGTAGAATTGACTTTGCCAC
PARP9_4	TGTGGCCCTGGACAATCTGG
PARP9_5	ACAGTTTCAACGGTCCCAGT
PATZ1_1	CAGGAGCCCATCCTGAATGG

PATZ1_2	GAGAAAGGTGAGCCAAGGGC
PATZ1_3	GCAGATGTAAGGCTTGCCCA
PATZ1_4	GGAGCGCAGACGGTCTCGGG
PATZ1_5	CCCATCTCTGAAGACCCCGA
PAWR_1	AGGGATGTTGACCACGCCGG
PAWR_2	CTCAACAACAACCTCCCGGG
PAWR_3	GTTGAGCTCGTTGGCAGCGG
PAWR_4	TTTGGGAATATGGCGACCGG
PAWR_5	ACCGCCGCCCCAGCGTGACG
PAX1_1	AATCGGATGAAGTTCACCCT
PAX1_2	AATGCGCAAGCGGATGGCGT
PAX1_3	GAAGGCCGTGCGGTTCACGC
PAX1_4	GCACGGCGTGTACAGCGCCC
PAX1_5	CCGCGTCACCACTCCCAACG
PAX2_1	AGTGGCGACGCCCAAAGTGG
PAX2_2	GAGAAGAGGAAACGTGATGA
PAX2_3	GATGCGCTGCCTCACCACGT
PAX2_4	GCAGCCTTTCCACCCAACGC
PAX2_5	AGCGCCTCCAATGACCCAGT
PAX3_1	AGACAAATTACTCAAGGACG
PAX3_2	ATTCGGGAAAGGTGAAGAGG
PAX3_3	CCAGCGGGAACCCGCTACGC
PAX3_4	GTTGACGCGGCCCTGGCCGA
PAX3_5	AATCCGAGACAAATTACTCA
PAX4_1	AGAAGATAGTCCGATTCCGG
PAX4_2	AGCAGCATGAACCAGCTTGG
PAX4_3	AGTCCTGCGGGCATTACAGG
PAX4_4	GAGGGAAGGGTACTTACACT
PAX4_5	CTAGGGCGTTACTACCGCAC
PAX5_1	AATTGGAGGATCCAAACCAA
PAX5_2	AGTGCTGCCTCTCAAACACG
PAX5_3	GGAGGAGTGAATCAGCTTGG
PAX5_4	GGCCGAATCCGTGCTCACCG
PAX5_5	GATGGAACTGACGCTAGGCA
PAX6_1	AACGGGCAGACCGGAAGCTG
PAX6_2	AACTCTATCACCTTTCTCCA
PAX6_3	AGCGGAGTGAATCAGCTCGG
PAX6_4	TTTACTACCACCGATTGCCC
PAX6_5	AAGTCCCCGGATACCAACCA
PAX7_1	CCAAAGGGAATCCCGTGCGG
PAX7_2	GGAGGATGAAGCGGACAAGA
PAX7_3	GGCCGGGTCAATCAGCTGGG
PAX7_4	GTAGGGAACCGGCTGGACGA
PAX7_5	AGCGCAAGCAGCGACGCAGT
PAX8_1	GAACCCTACCATGTTTGCCT
PAX8_2	GAGCCCAGGGAATCCGACTG
PAX8_3	GAGCTGGCGAGAGATGTCGC
PAX8_4	GCAACCATTCAACCTCCCTA

PAX8_5	CAGAGGTCTGCCATTCACAA
PAX9_1	CAAGGTGCCCACGCCACCCG
PAX9_2	GCGGCATGGCCACCGAACCG
PAX9_3	GTAAGGCGACACTTGGGCTG
PAX9_4	TGTCCAGCAACATAACCAGA
PAX9_5	ATCCGCTCCATCACCGACCA
PAXBP1_1	AAGATGACGATGAGAAACGC
PAXBP1_2	AAGCCGAGTGGACTCTACCA
PAXBP1_3	AAGCTGAGCAGGCTGGCCCG
PAXBP1_4	AGGATGAAGAGCTCAGCCGA
PAXBP1_5	ATGCCATAGGATGAGCCGTA
PAXIP1_1	ACGGACAGAATCACCCAAGA
PAXIP1_2	AGCCTCACACATAATCTCAG
PAXIP1_3	CTGAAGACAGAAGTGCCCTG
PAXIP1_4	GGAGGTCAAGTATTACGCGG
PAXIP1_5	GCACACAAGTTAATCAACCC
PBK_1	GAAACCCAAAGAAGCTGTGG
PBK_2	GAGGCCGGGATATTTATAGT
PBK_3	GAGTGGCTTTCACAATGGAA
PBK_4	GTTACATTGGCACAGAGCCA
PBK_5	AAGCTTCTGCATAAACGGAG
PBRM1_1	ATCTCAAGACCATTGCCCAG
PBRM1_2	GAAGATGGGCAAGACAATCA
PBRM1_3	TCATTAGGGCACCAAAGCGA
PBRM1_4	TGGGATTTCTCTCTTCACCA
PBRM1_5	AGGAGTTGTCGGAATAACCA
PBX1_1	CAAGCAGAGCACGTGCGAGG
PBX1_2	TATCAGAGTGAACACTGCCA
PBX1_3	TCGCAGGAGATTCATCACGT
PBX1_4	TCTGGCTGATAACATGGCGA
PBX1_5	CAGCAACCCTTACCCCAGTG
PBX2_1	AGAGAGCAGGCTTCATTCGG
PBX2_2	CATCCAGGAAACGGGAGCGC
PBX2_3	GCGATAGTCCGAGTGTTCGA
PBX2_4	TAGGATATGGGTTACTCAGG
PBX2_5	AGGGGACACACCACCACCAG
PBX3_1	ATCGGGAGGGTCCTCCTCCT
PBX3_2	GTGGCCAGCCAGGTTCACCC
PBX3_3	TCTGATTGATAACATGACGG
PBX3_4	TCTGTGACTTCTCCTACAGA
PBX3_5	ACTCTTGTCATAGACGGAAA
PBX4_1	AAGAGGAGGAGCGGTGGCCA
PBX4_2	ACAGAGCAGGCTTCATCCGA
PBX4_3	CATCGAGCAGCCGCGAACGC
PBX4_4	GGGCGGCCTCACCATCTCCC
PBX4_5	AGCATGCTCTGAATTGCCAT
PCBP4_1	AGAATGAGCGGCTCGGACGG
PCBP4_2	GATCTGCGCTGTTATCCTGG

PCBP4_3	TGCCCGGATCACCATCTCCG
PCBP4_4	TGGCAGGGATGACAAGGCGC
PCBP4_5	AGCTGCTGTAGACCCGGTGA
PCDHB13_1	ACCCTTCCTCCTGAAATCCG
PCDHB13_2	ATTGACATCGGCGATCAGCA
PCDHB13_3	GAGCTGTGTTATCAGCATAG
PCDHB13_4	TCAGGTCTACATCGAAGTCC
PCDHB13_5	AGAGGAAACGTAGTCCCAGG
PCGF1_1	ATTGTTTGCTGCCTATGCGC
PCGF1_2	GACCGGGTCACCCAGCCCAC
PCGF1_3	GCTCCAGTCAGTGTACAAGA
PCGF1_4	TGAGCAGTGGCTGTGTCTCG
PCGF1_5	CCACGAAGTAGCCGGCGCAT
PCGF2_1	CAGATGAGATGAAACGGCGG
PCGF2_2	CAGGCACTCCACGATAGTGG
PCGF2_3	CCTGCTCCAAGACCTCGCCG
PCGF2_4	TGTGCCCAGCAAGTACAAGG
PCGF2_5	AACGGCTCCAATGAGGACCG
PCGF3_1	GGTGCCGGGAGACATCAAGG
PCGF3_2	GTGCAGACACTCGGTCACCG
PCGF3_3	GTGGATCACAATCCTGCAGG
PCGF3_4	TCAGACACTCACCTTCTTGG
PCGF3_5	AACAGCACTTAGATTCCCAT
PCGF5_1	AAGATAGCAATGATTGCCCA
PCGF5_2	AAGTTGGATGTGCTGTGCAA
PCGF5_3	AGCCACGAATGGCTACCCAA
PCGF5_4	GATCAAGCCAACAACAGTGA
PCGF5_5	ACGAAATAATGGGCAATCAG
PCGF6_1	GGTCTAGAAGTACCTAAACC
PCGF6_2	GTCTCTCCCGACCATGGAGG
PCGF6_3	GTGGTGACGGCGGGCAGCGT
PCGF6_4	TGGAGCAGTATCAAACTCTA
PCGF6_5	CCTCACCCCTGCACCCGCAG
PCMT1_1	ATAGATCAGTTAAAGCCCGG
PCMT1_2	GCGATGGCCTGGAAATCCGG
PCMT1_3	GCTCCGAGTGTGCTTAGCGA
PCMT1_4	TAACAGCGGCAGCTACAGCG
PCMT1_5	AACCACCTTCAGCGCGACGA
PCNA_1	ACTCAAGGACCTCATCAACG
PCNA_2	ATACGTGCAAATTCACCAGA
PCNA_3	TGCTTCAAATACTAGCGCCA
PCNA_4	TGTCGAAGCCCTCAGACCGC
PCNA_5	GCTGGAGCTAATATCCCAGC
PDP1_1	AACACCAGAAACTTATCCTG
PDP1_2	GAAGGTCAACACCATCCACA
PDP1_3	GAGCTTATAGACCTCAACAC
PDP1_4	TATCCTGTGGCCTTAATCGG
PDP1_5	AAAATGCCACTCGAAGCACC

PDX1_1	AGTCTACCAAAGCTCACGCG
PDX1_2	GAGGACAAGAAGCGCGGCGG
PDX1_3	GAGGAGGACAAGAAGCGCGG
PDX1_4	GTGGTGAAGGTGCGCCACCG
PDX1_5	GACCCATGCGCGTTCCAGCG
PEG3_1	AAGGAGCCGGATGTCATCAT
PEG3_2	GCTAGACAGTGACTTGACTA
PEG3_3	GTGCAGCTTGCTGAAGACGA
PEG3_4	TCTGCAGCACGATTCCTCCG
PEG3_5	ATGAGGCAAAATCATATGAG
PELP1_1	ACAGTGTGAGCAGTCAGCAT
PELP1_2	ATGACGCAAACACACCGCGA
PELP1_3	GCAGCTGGGCTGCATATCGG
PELP1_4	GCAGGAGCTACACAGTCTGC
PELP1_5	CCAGCGAGAAGATAGCCTTG
PES1_1	ATAGGGAGTGATCCACACGA
PES1_2	GTTCGTCCGGAAGCTCCGGA
PES1_3	GTTGACAAAGCCCAGCAGCG
PES1_4	TGAACTCCACAGTGAGCCGG
PES1_5	AATGCACAAAGATTTGTCCC
PGR_1	GAAGACCTTACAGCTCCCAC
PGR_2	GTGGATGAAATCCATCACCG
PGR_3	TGAAGCTTGACAAACTCCTG
PGR_4	TGACTTCGTAGCCCTTCCAA
PGR_5	AGACGAAAGTTACGACGGCG
PGS1_1	AAGGGAGCCAATAACAGCCA
PGS1_2	CATTCTCTTAGACTTCACGC
PGS1_3	GAAGCGGCGAGTCTCCATGG
PGS1_4	GATAAGAGTAGCCAAGAGGC
PGS1_5	ATTCTCTTAGACTTCACGCG
PHB2_1	CAAGTGCTCAGACCTACCTT
PHB2_2	GCAGGACACTATCCTGGCCG
PHB2_3	GGGTCGAGACAACACTCGCA
PHB2_4	TTCGCGCACACCGTAGGCCA
PHB2_5	CACAGATTCGCGCACACCGT
PHC1_1	ACTCACCGAGGCCTGGGTGG
PHC1_2	GGATCGGCTGATGAGCTGGG
PHC1_3	TAGAGGCACATTCCGACCCA
PHC1_4	TGAGCTAGAACTCCCATTGG
PHC1_5	CACAGCTACACACCTCCAGT
PHC2_1	AGCCTGCTGAGCGATGCCTG
PHC2_2	CACCGAAGATGACTGGGCCG
PHC2_3	TGGAGTCCTCACCTGGGCGG
PHC2_4	TGTGCCACCAGCAGTACCAG
PHC2_5	CCACTGTACACAGAAATCTG
PHC3_1	AGACCATCTACATCTCCCAC
PHC3_2	GCTGGTAGAACTGGAAGCCT
PHC3_3	GGTACTGAGCAGCTGAGCTG

PHC3_4	TAAGGACCATAGTACAGCTA
PHC3_5	ACCAGTTAATAGCACCAGGT
PHF1_1	AGAGGAGTTCCTCTCCAGGG
PHF1_2	CGATCGCAAAGACACACTGG
PHF1_3	CTATGGAAAGACATTAGCCC
PHF1_4	GCACCAGAGCGGCTCAGCCG
PHF1_5	ACTGATGGGCTGCTATACTT
PHF10_1	AAAGGTCAGTTCTTACCCAG
PHF10_2	ATGGGACCCAGCCATCCAAA
PHF10_3	GTGGCTGGGTCGCTGTCGCA
PHF10_4	TTTGCCAACAGAGCGAACAA
PHF10_5	AGGTTATCCAGGTACCTCAA
PHF11_1	AAGGAGGAGCGCCTCCTGCG
PHF11_2	AGGAGGAGCGCCTCCTGCGC
PHF11_3	CTGCAGCACAGTCATGGCCC
PHF11_4	GACGCAGTTCCACAGTCTGA
PHF11_5	CCTCGAACTCCATCAGACTG
PHF12_1	ACGACTTGGACACATCAGGG
PHF12_2	GTGGCCCTGCCGCTTCTCCG
PHF12_3	TCAAAGGGCCGCCTCAGCTG
PHF12_4	TGACACATCCACTCTCCAGG
PHF12_5	ACGAGGCAGAAAAGCGCAGT
PHF13_1	AAACGGACTATCGTCCGGCA
PHF13_2	GCCAGCATAGGCCAAGACAA
PHF13_3	TCCGCGAGCGGTTGGAACGG
PHF13_4	TGGCTACATCCCTTATCCGA
PHF13_5	AATACTCCCCCAGTTGCAAG
PHF14_1	GAACTGTGTCCTAATCAGGA
PHF14_2	GAAGTGGTCGGTTTCGTCGA
PHF14_3	TGGATCGCAGCTCCAAGAGG
PHF14_4	TGTGGCATTACAGTCCATGA
PHF14_5	AGCTCGATCTACCAGACCCC
PHF15_1	CTCATCACTACCCACCTCGG
PHF15_2	GATATGCGAGATCTTGGTGA
PHF15_3	GTGCCACCAGAATATGGCCA
PHF15_4	TGCCTGGCTCACCTCACCAC
PHF16_1	CTTGTGCAAGTTGAAGACGG
PHF16_2	GCATGCAAATACCTGAGAGA
PHF16_3	GGAGGAGGAGTTCTATTCCT
PHF17_1	AATGTGTGACACCTTGGTGA
PHF17_2	AGATGAATACACCATGGAGA
PHF17_3	TTTCTCTTCAGACACAACCC
PHF19_1	ATGCAGCGTCGGCAGAACCA
PHF19_2	GGTGCTGTCCTACCAGCCCG
PHF19_3	GTGCAGGCAGTGGTTCCACG
PHF19_4	GTTGTTCTTGACCTTCGCCA
PHF19_5	AATTATCTTCGAAAGTCACG
PHF2_1	ACGTCACTGACATAGAACGT

PHF2_2	CCTGGCACAAACACGGCCCG
PHF2_3	CTTACAGGTGGACTTCCCAT
PHF2_4	GCGGCGCGGCAACATGGCGA
PHF2_5	GGATGAGTATGAGTACGTGT
PHF20_1	ACGAGGAATCAGCTTTGAAG
PHF20_2	AGAACCACTCATCATAACGA
PHF20_3	AGACACGTTGTCTAGTACCA
PHF20_4	TAACAGCAGTGACTTTGGCC
PHF20_5	ACACTGACAAAGACTTATCG
PHF20L1_1	ATAGGACAACACCAGCTTAG
PHF20L1_2	GCAAGAATCTTCAGTACCAG
PHF20L1_3	GCACATTAAAGCCATGCCCG
PHF20L1_4	TTACCGTAATGAATGTCCCA
PHF20L1_5	ACTGGTTACACGATCCAGCT
PHF21A_1	ATAGGTTCGACCCAAGCCTG
PHF21A_2	GATGGCGGTCACCATAGCAA
PHF21A_3	GGAGTTGCAGACTCTACAGG
PHF21A_4	TGTGGAAGAAAGTTTGGACG
PHF21A_5	ACAGCAAATCCGGTCTACAG
PHF21B_1	AGAGTCGCGGCCGCCCACAG
PHF21B_2	AGCTCCACGAAAGGCAGCCG
PHF21B_3	GCCCGAGGGCTGCTTACCTG
PHF21B_4	GCTGACAGTGTTGTTGGCGG
PHF21B_5	AGCGCTAGGCCTGGTTACCA
PHF23_1	AAGCAAGGTAGCACTGTCGG
PHF23_2	ATGCAAGGAACTGAGGCCAG
PHF23_3	ATGGCGGAGCCCAGTCCCGA
PHF23_4	TCTGGCTTAAGGGTCGGAGG
PHF23_5	AGTTAAAAAAGGCAGAACGG
PHF3_1	AAGGAAGAGCTTGAACACCC
PHF3_2	AGAGGACCCATCATACGCTG
PHF3_3	GCAGGAGAGAACGTTGACTT
PHF3_4	TCAAGCACAGCAGATGGGCG
PHF3_5	CTACCAGATAGTATTCAAGT
PHF5A_1	ACATATGCGCACCAGAGTGC
PHF5A_2	ACTTACGTCCTTCTCCTGGA
PHF5A_3	GTACACTTACGTCCTTCTCC
PHF5A_4	CACCAACACCAGCCTGCTTG
PHF5A_5	CAGGGGCGCTGTGTGATCTG
PHF6_1	AAGACTTACCATTACCACTG
PHF6_2	AGTGACACCAGGCCTAAATG
PHF6_3	ATACGAGAGAAACCTTCACA
PHF6_4	GTACTTCAGGAGATTAAACG
PHF6_5	AAAACTGCACATAACTCCGA
PHF7_1	AGACTAGGAGGGTAACCCAG
PHF7_2	TAGATGGCTTGACTACAACA
PHF7_3	TATCTAGTAAGCTGCCTCAG
PHF7_4	TGCCTGGGAACTCGAGCCAG

PHF7_5	AAAATCTGCCAAGACTAGGA
PHF8_1	AACGCCGTGGATCTTCAAAG
PHF8_2	CTCACAGTGAATGATGGCGA
PHF8_3	GAGGCAATACACCGGCACCG
PHF8_4	GAGGCAGTACTTCTGTACAT
PHF8_5	AGGGGCATGATACACACAAG
PHIP_1	GATCCGCGAGGTGGCCGAGA
PHIP_2	GGAAGATGGACCCTGTCAGC
PHIP_3	TAGTTAACTGGTGACTCAGG
PHIP_4	TATGGGCAACAGATGATGGG
PHIP_5	AGCATTGAAACAGACTATCA
PHOX2A_1	GCAGGCGCCAAAGTCGCCGT
PHOX2A_2	GCTCAAGATCGACCTCACTG
PHOX2A_3	GTGAAAGGAGGACAGAACCC
PHOX2A_4	GTTTGCGGAACTTGGCCCGG
PHOX2A_5	AGGCGGTGCTATCGGGCGTG
PHOX2B_1	AAACTCTTCACGGACCACGG
PHOX2B_2	AAGCTGAAGGTCCTTACCTG
PHOX2B_3	CATACAGGACTCGTAGGCAG
PHOX2B_4	CGAGGAGCCGTTCTTGGCCG
PHOX2B_5	CCTACGAGTCCTGTATGGCT
PHRF1_1	CCTGGTGTTGTCCTTGCCGC
PHRF1_2	GAACACCAAAGCGAGCGAGG
PHRF1_3	GTGTCTAGAAGAAAGCAGCG
PHRF1_4	TCCGAGGATTCTGAAGACGA
PHRF1_5	CCACTCGCTCTCGAATCGCG
PHTF1_1	ATGAGAGAGATGCTATATCG
PHTF1_2	CATGTGAATACCCTTCACTC
PHTF1_3	GAGTAGACACAATTTGACAG
PHTF1_4	TGAATCAGCAGCCTTCAGCC
PHTF1_5	AGAAAGCACCCGCCATGACT
PHTF2_1	AACACAGGAACACTGAGGAA
PHTF2_2	ACACAGCATACCTCTGACAG
PHTF2_3	GGGTGGGAACTATGGAGCCA
PHTF2_4	TGTGAGAGGTGCTTGTACCG
PHTF2_5	AAGGTGAACAGCCATATACC
PIAS1_1	AAATAGCTACAACCAGCCTA
PIAS1_2	AAGAAGAGCCATCTGCCAAG
PIAS1_3	GATGCCTATAGTCTTGGATG
PIAS1_4	TTAAGGAACTCTATAGGCGG
PIAS1_5	ATTCCACAACTCACTTACGA
PIAS2_1	AATGCAGCGCCCTCATCAGG
PIAS2_2	GGCACATCAAGGATACCCGA
PIAS2_3	TCACTGAACAAGGCTTACTG
PIAS2_4	TGTAGAACCTGACTTGGCCG
PIAS2_5	CAAACACTTACCTAAACTCG
PIAS3_1	AGATGACCAATTGACCACAA
PIAS3_2	AGTGCGCTTCCTCAAACCGC

PIAS3_3	GAAGCACGAGCTCCTGGCCA
PIAS3_4	GGAGAGCATTTACCGGCAGG
PIAS3_5	ACCACAAAGAGCCATACCAA
PIAS4_1	AACCGCATCACTGTCACCTG
PIAS4_2	GAAGCACGAGCTCGTCACCA
PIAS4_3	GAGGGACACCCGCACACCGG
PIAS4_4	GCGGGTACTGGTCCTCCTGA
PIAS4_5	GATGAGGGACACCCGCACAC
PITX1_1	ACTGCAGAGAAGGAGCGCGG
PITX1_2	AGTTCTCGAGCGGCTCGCGG
PITX1_3	CGTGTCAGACGACTCGCTGG
PITX1_4	GTTGTTGTAGGAGTAGCCGG
PITX1_5	AGCGCGGCGGGGAACCCAAG
PITX2_1	ACAGGCAGGCGTCGGCACCG
PITX2_2	GAATGAGGACGTGGGCGCCG
PITX2_3	GGGAGCCAGAACCGAAGCCA
PITX2_4	TCACTTGGAGCACCGAGCAG
PITX2_5	AGCAGCGGGGACCAAGCTGT
PITX3_1	GCTGAGCAGGCCGAACTCCA
PITX3_2	TAGTCCGTGCCAGTACGCCG
PITX3_3	TGGAGAGCATACCCGCACGC
PITX3_4	TTTCAGCGAACCGTCCTCTG
PITX3_5	AGTTGCCGTACGAGTAGCCG
PIWIL4_1	AATGAAGCATCCTCTAGCAA
PIWIL4_2	AGAGCCCGAGTGAAGGCCAG
PIWIL4_3	TAAAGATTATCCCTTTGGCC
PIWIL4_4	TGACCAGTCAATGTCATCAA
PIWIL4_5	AATCTGGAATATATGTCACA
PKM_1	AGTGTGACGAGAACATCCTG
PKM_2	CAAGAATGTGCGCACAGCCA
PKM_3	GAAGGGTGTGAACCTTCCTG
PKM_4	GTGGTGAATCAATGTCCAGG
PKM_5	CAAAATCGAGAATCATGAGG
PKN1_1	ATGATCCAGACCTACAGCAA
PKN1_2	GCTGCGGCACCACTTCCGAG
PKN1_3	GCTGCGGCGGGAAATCCGCA
PKN1_4	TCATCCGCATGCAACTCCGC
PKN1_5	ACCTCCCAGAGACCATCCCG
PKNOX1_1	ATGGTGAGCTCGCTCGGCGG
PKNOX1_2	GCAGCAGGGAAACGTAGCCA
PKNOX1_3	TCAGCATCCCTACCCAACAG
PKNOX1_4	TCTGTGTGACCACTTGGCCT
PKNOX1_5	AACAGTGAAACTCTGTTGAG
PKNOX2_1	GATGTGGACAGCCTGGGCCT
PKNOX2_2	GGCCATCATCGTCAGAGCGG
PKNOX2_3	GTCCACATCAAAGCTGGCGG
PKNOX2_4	TTGGGTGACCACCTGACCCT
PKNOX2_5	AAGAAGTCCAAGAACAAACG

PLAG1_1	AAGGTAAGGACATCACCGGA
PLAG1_2	ACTGATAGAGGATTATAGGG
PLAG1_3	ATTGGGCATGTCATTCCCAA
PLAG1_4	CATACAACAAGACTGCACCA
PLAG1_5	GCCTTGCATGCCGCAACAAG
PLAGL1_1	GAGGCCAGGGACTCTGGCAG
PLAGL1_2	GCTGCCAGAAGCCCAACAGG
PLAGL1_3	GTGAATCGTGAACTTCTCCA
PLAGL1_4	TGGCAAGACGTTCCTCACCC
PLAGL1_5	AAGATGCTTCTACACCCGGA
PLAGL2_1	CAGAGGGTAGCTCATACCCA
PLAGL2_2	GATTCAAGATGCAAAGCAGG
PLAGL2_3	GTGGGCACCATACATGCCCA
PLAGL2_4	TTTGCATCTTGAATCCAGGG
PLAGL2_5	AGCTGCCCTCAGCTGCACTG
PML_1	AGTCGGCCGACTCTTTGCAG
PML_2	ATCCAAGAAAGCCAGCCCAG
PML_3	GGAGCTGGACGCCATGACGC
PML_4	TGGGCTGGCGGCCTTCAGAG
PML_5	AGAAGGCGTACACTGGCACG
PMS1_1	AAACCTTCACATCTTGGTCA
PMS1_2	ACTAAGAACGATGTCAGCTG
PMS1_3	AGGAGTCGAACTGTTGCCGC
PMS1_4	ATACCTGAGATTCTTCAGAG
PMS1_5	AAGGTTTCTGAGAAAGTATG
PNMT_1	ACTGAGCAGCTGGTACACGG
PNMT_2	GGAGCGCCAGCTGCGAGCCA
PNMT_3	TTGGCGCAGACCTTCGCCAC
PNMT_4	TTTCCTGGAGGTCAACCGCC
PNMT_5	CACCATGACAGATTTCCTGG
POGZ_1	AAGAGTGGCCGGGATGACGG
POGZ_2	AATCCTTTGGTCCAGCAAGG
POGZ_3	ATTGTGCTGAACGTACAGCA
POGZ_4	GGTGCCCAGCAACAGAAGCA
POGZ_5	CATTCCAACAGCATTACATG
POLD4_1	ACACCTCAGAGACCTAGCTC
POLD4_2	CAGGTGCTGAAGACCCACCC
POLD4_3	GATGAGCCGCTTCCGGCCCA
POLD4_4	GGGCCCGCTGGGCACAGCAA
POLD4_5	CCTCCTCTTCACAACCGGGT
POLE3_1	CAGTGATGTGCTCTCAGCCA
POLE3_2	GGACGGTGTCAACATCTCCA
POLE3_3	GGCAATGGCGGAGAGGCCCG
POLE3_4	GTACAGCACGAAGACGCTGG
POLE3_5	CCGTCCTTACAGCATATAGG
POLQ_1	AAAGTTCCAGCAGATACCCT
POLQ_2	AGGCAGCGACCAAGGCCGGG
POLQ_3	ATTGAAGGAGCCTTTCGTCA

POLQ_4	GAGGCGATTGATCAGACCAT
POLQ_5	CTGACTCCAAAAGCGGTACA
POLR1B_1	GAACCAGGAAGTCCTCACAC
POLR1B_2	GCCGAAGGAGTACCTACCGT
POLR1B_3	GTACCCAAATGAGCAAGCTG
POLR1B_4	GTGGAGGTTCATATTGACAG
POLR1B_5	ACAGGCCGTACCAGTCTACA
POLR1C_1	AAACTCACTGGAGTTTGACA
POLR1C_2	CCAGGCTGATCTCTTTCCAG
POLR1C_3	GATGGCGGCTTCTCAGGCGG
POLR1C_4	GGACCAGGACCGCTTCGAGA
POLR1C_5	GGATATCATCATGCACTGGT
POLR2A_1	GGAAGTGGGCGAGTAGCTAG
POLR2A_2	GGACTGGTAGGCGAGTACTT
POLR2A_3	GGACTGGTTGGAGAATAGGA
POLR2A_4	GGTAGGCGAGTACTTGGGAG
POLR2A_5	CATGCGGCGGGAACACAACG
POLR2B_1	AAATAGCCACCAGGATCCAA
POLR2B_2	AATCACTGACACCAACATGA
POLR2B_3	GAGCACCATCTCTTTCCCAA
POLR2B_4	GTTTGGCAATATGTACGACG
POLR2B_5	ATAGACCGTGTAGTCACAAG
POLR2C_1	AAAGAGTGAGTACTCGGAGC
POLR2C_2	ACGCCAACCAGCCTACCGTG
POLR2C_3	CAAGTGGAACCCTACTGCAG
POLR2C_4	TCGGAGGGTCTTCATCGCTG
POLR2C_5	ACAATGTCATCACTAATGAG
POLR2D_1	GCAGCAGAATGAGAGTGCAG
POLR2D_2	GCGGGCTGGCGACGTAGAGG
POLR2D_3	TCACAACTGGTACTAACCTT
POLR2D_4	TCACCTACGAACACTGGCAA
POLR2D_5	TAGCACTCACCTACGAACAC
POLR2E_1	AATCCGCAAGACCATCATGC
POLR2E_2	GCAGGAGGAGAACATCACAC
POLR2E_3	GGAGCTGCTCATCAACATCA
POLR2E_4	TTGGGCTTTGAACTCCTCCA
POLR2E_5	ACGGTGAGGTCCGTGCGCCG
POLR2F_1	ATGGGAGCTATGAAGACTGG
POLR2F_2	CTCCAGCTCCACCATCACAG
POLR2F_3	GATGGGAGCTATGAAGACTG
POLR2F_4	GTACGAGCGAGCCCGCGTGC
POLR2F_5	GATGTGTGCCCCTGTGATGG
POLR2G_1	AATGTCATTCTTGTCCACAC
POLR2G_2	GAAGCAGAAGCTCTTCACCG
POLR2G_3	GCAGAAGCTCTTCACCGAGG
POLR2G_4	TAAAGGACAAAGCCTCGGCC
POLR2G_5	AGCACCAATATTGTCAATGG
POLR2H_1	GTGAAGGATATTGACCCGGA

POLR2H_2	GTTGTATTCACCATCATCCA
POLR2H_3	TGGAAGGCCTATCATCAGTG
POLR2H_4	TGTGAAGGATATTGACCCGG
POLR2H_5	CTGGAAGGCCTATCATCAGT
POLR2J_1	AGCAGGAAGGAATTGAGTAG
POLR2J_2	CCCGAAAGCGCTCCTCCAGC
POLR2J_3	GAAGAGCAAGAACGACTCGA
POLR2J_4	GATGGTGAATAAACAGGCAT
POLR2J_5	TCCTAGGATCACCATTAACA
POLR2J2_1	ATCACCGCCGCCTCTCCCTG
POLR2J2_2	ATGGTGAATAAGCAGGCCTT
POLR2J2_3	CAAGGACACCAAGGTACCCA
POLR2J2_4	GATGGTGAATAAGCAGGCCT
POLR2J2_5	TAAGCAGGCCTTGGGTACCT
POLR2J3_1	AACAAAGAAGACCACACACT
POLR2J3_2	GATCTTGTGCTCCAAGGGGT
POLR2J3_3	TACCTTGGTGTCCTTGTTAA
POLR2J3_4	TTTGTTGATGGTGAATAAAC
POLR2K_1	GCTAACAATGGACACCCAGA
POLR2K_2	TGGAGGTTGAACGTCCTTCT
POLR2K_3	TTGGAGGTTGAACGTCCTTC
POLR2K_4	ATCATTGGTTGCTGCTTTGG
POLR2K_5	CAACCAATGATATATATCTG
POLR2L_1	ACAAGTGGGAGGCTTACCTG
POLR2L_2	CGTGGGCCAGCAGCATCCGG
POLR2L_3	GCAGTAGCGCTTCAGGCCCA
POLR2L_4	GCTGCAGGCCGAGTACACCG
POLR2L_5	CAAGATCGTCGGCAACAAGT
POLR3K_1	AGTAGAGTCAACATTCTCCC
POLR3K_2	CCTGGCCCTAATCCCTCCAG
POLR3K_3	CGTGCACAACATCACCCGCA
POLR3K_4	GAACGGGCTGATCGTGGAGG
POLR3K_5	GCACAACATCACCCGCAAGG
POP7_1	GGAGCCAGAGACCGACACAC
POP7_2	GGAGGCTGAACTGGATCCAG
POP7_3	GGCTGCCAATACCTCCACCG
POP7_4	GTTCAGCCTCCACAGCACCG
POP7_5	AGAGCACGCGTTCTGACCCC
POTEC_1	CCAGTCTCCAGAAATTAGCA
POTEC_2	ACAACATACAGCAAGTATGA
POTEC_3	GGGACACGGACATGAACAAG
POTEC_4	GGGGAGCGGCACGAGCAACG
POTEC_5	GTGGACAGCATAGTGTAGAG
POU1F1_1	AGAGCCATGCACAGCTGCCA
POU1F1_2	GCAGAGGCGTCAGAATTCAG
POU1F1_3	GGTGGAAGAGCCAATAGACA
POU1F1_4	TTCTCAAGAGATCATGAGGA
POU1F1_5	AGGAGGAAATCCATGACTCA

POU2AF1_1	AAGCGAGGCCACGCCAGCAG
POU2AF1_2	AAGCTCCGCCACGCCCGCAG
POU2AF1_3	AGAGGCATAGGTCAACACTG
POU2AF1_4	GCGAGGCCACGCCAGCAGTG
POU2AF1_5	ACTGTGGTGTAGGTCGCCAG
POU2F1_1	AGCTGGAGGACAGATAACTG
POU2F1_2	TCAAGATGAGAGTTCAGCCG
POU2F1_3	TCAGCCCATACAGATCGCAC
POU2F1_4	TCCAAGGAAAGCCTGCGCCT
POU2F1_5	ATCATCTCACAGACGCCCCA
POU2F2_1	GGTAGCAGGAACTGAGCAGG
POU2F2_2	GGTGGCTCCAAGCATTTGGG
POU2F2_3	GTTGCCGTAGAGCTTGCCCA
POU2F2_4	TGTGTGGGAAGCCCGGCCCG
POU2F2_5	AGTGGCTCAACGATGCAGGT
POU2F3_1	GACTCAGGTGGCATGGCCGA
POU2F3_2	GAGGGCCTCAAATCGTGAGA
POU2F3_3	GATGCTGGGATGCTTCCAGG
POU2F3_4	TCGCAGCACTCTCAGCCAGG
POU2F3_5	ATCACAGTGTTACCTGACAT
POU3F1_1	GAACACGGCAAGGCAGGCGG
POU3F1_2	GAAGCGCACGTCCATCGAGG
POU3F1_3	GAGGAGACCGACTCGTCCAG
POU3F1_4	TATGCACCCGGACGCCGCGG
POU3F1_5	ATGTCCGTGTGCGTGTCCGT
POU3F2_1	GCCCTGCTGCACCACCACCG
POU3F2_2	GGCGCCGAGGATGTGTACGG
POU3F2_3	TAACCACCACCCGGGACCCG
POU3F2_4	TAGACAAGATCGCAGCGCAA
POU3F2_5	ATCACCGCGCTGTCCCACGG
POU3F3_1	CATGGACGGGAGGTGCGCGG
POU3F3_2	GAGTAGAGCAGACTCTGCGG
POU3F3_3	GTAGGCGCCCGAGGTCACGG
POU3F3_4	GTTGGTGATCTCCTGCGCGG
POU3F3_5	GGACGAGGACACGCCGACGT
POU3F4_1	AGCGGCATGCTGGAACACGG
POU3F4_2	GGTCACCCAGTGATGCCCGA
POU3F4_3	TGGGTGACCAGTCTGAGCGA
POU3F4_4	TGGTCACCCAGTGATGCCCG
POU3F4_5	CAGGCTGCGAGTACACGTTG
POU4F1_1	CCAGCTCCGAGGCCATCCGG
POU4F1_2	GAAAGGATGGCTCTTGCCCT
POU4F1_3	GAGGGAGCGCTTCTCGGGCG
POU4F1_4	GTCGCTGCACTCCAGCTCCG
POU4F1_5	ACGGCACGCTGTTCATCGTG
POU4F2_1	AAGAGTCTTCTAAATGCCGG
POU4F2_2	CAGGCGTTTAGCATGCCGCA
POU4F2_3	CAGGTCCAGCTTCTCCGCGA

POU4F2_4	GCGTTTAGCATGCCGCACGG
POU4F2_5	AGCAGCTCGAGCAACGCTGG
POU4F3_1	ACGCGCCGAAGCTCTGGCGG
POU4F3_2	AGGCCGCTCACACTCAGCGT
POU4F3_3	GAACGGATGGTTCTTGCCGT
POU4F3_4	GTGCGGGTGACTCATGCCCA
POU4F3_5	CACGCTGCTCATGGTATGGT
POU5F1_1	GAAGCTCACTTGCCTCCTCC
POU5F1_2	CAACAATGAAAATCTTCAGG
POU5F1_3	CCCACAGAACTCATACGGCG
POU5F1_4	CCCACCAAATAGAACCCCCA
POU5F1_5	GAAGCTGGAGCAAAACCCGG
POU5F1B_1	CAAAGCAGAAACCCTCATGC
POU5F1B_2	CCCGCCGTATGAGTTATGTG
POU5F1B_3	GCTGGGTTGATCCTCTGACC
POU5F1B_4	GGTGGAGAGCAACTCCAATG
POU5F1B_5	CCAGCTTCACGGCACCAGGG
POU5F2_1	ACAGCCGGGCCTCCTTGCCC
POU5F2_2	AGAGTTGCAGCAATTGGCCA
POU5F2_3	AGGTCAGAGTGTCAACCCGC
POU5F2_4	GCCTGGAGAAATTCTTCCAG
POU5F2_5	AGCTAAGCGTCGCCAACATG
POU6F1_1	GAGAAGAACCCACTGCCCAC
POU6F1_2	GGACACCAACTGGCTGACGG
POU6F1_3	TAAGGCCCAGCGAGAGCCGC
POU6F1_4	TGGGCGTTCAACAACAGCTG
POU6F1_5	AGTGAACTCAGCTAGTGTGG
POU6F2_1	AACATGGCGGGACAGCTAGG
POU6F2_2	GACTGACTAGGTTTCTGCGG
POU6F2_3	GATTCCACAGGCGCAAGCAG
POU6F2_4	TGTGGCCCAACTGGAAATGG
POU6F2_5	ATGTTGGTGAGATTCGCTGT
PPARA_1	AGAGAAAGATATCGTCCGGG
PPARA_2	CACAGGATAAGTCACCGAGG
PPARA_3	GATTTCGCAATCCATCGGCG
PPARA_4	GGAGCTCACGGAATTCGCCA
PPARA_5	GACTCCGTAATGATAGCCTG
PPARD_1	AAGGCATCGGGCTTCCACTA
PPARD_2	AGAGGTACTGGGCATCAGGG
PPARD_3	GAGCCAGTACAACCCACAGG
PPARD_4	TATCACTGAAGGGTTTGCGG
PPARD_5	AGGAGCCCCAGAGCTCAATG
PPARG_1	AGAGCCTTCCAACTCCCTCA
PPARG_2	ATTCACAAGAACAGATCCAG
PPARG_3	GCTGACCAAAGCAAAGGCGA
PPARG_4	GTTTCAGAAATGCCTTGCAG
PPARG_5	ACAGATGTGATCTTAACTGT
PPARGC1A_1	AAGTCCTCAGTCCTCACTGG

PPARGC1A_2	GACACTAGACAGTCTCCCTG
PPARGC1A_3	GATACAGACAGCTTTCTGGG
PPARGC1A_4	TATTGAACGCACCTTAAGTG
PPARGC1A_5	AGGACTAGCCTCATTGTCAG
PPHLN1_1	ACACTGGGATGACTTCGAGG
PPHLN1_2	GATTACCGAGACTATGACGA
PPHLN1_3	GCAGAAGCTACTCTCCAGAA
PPHLN1_4	TCTGGCTGGTCAGTAAACAA
PPHLN1_5	AGGTGTTAGACAAACCCAGT
PPIB_1	CTTGCCGCCGCCCTCATCGC
PPIB_2	TGAAGTCCTTGATTACACGA
PPIB_3	TGTGGCCTTAGCTACAGGAG
PPIB_4	TTGCCGCCGCCCTCATCGCG
PPIB_5	AAAGACTGTTCCAAAAACAG
PPM1G_1	GACGCGCCAGCCTTGCATGG
PPM1G_2	GATCAGAAGGCCTACAAGGA
PPM1G_3	GGAAGATGAGGATGACACCG
PPM1G_4	TACGGGCAGAACTGTCACAA
PPM1G_5	AACTTGGACTTAGCAACTCG
PPP1R10_1	GAAGGCCCTGGCGGAAGCAT
PPP1R10_2	GAAGGCCCTGGCGGTGGCAT
PPP1R10_3	GAAGGCCCTGGTGGTAGCAT
PPP1R10_4	GATGAAGGAAGCACGAAAGA
PPP1R10_5	AGCTCCGGGACTTCAACAGG
PPP1R12C_1	AAGGCGGAGATCGCCCGCCG
PPP1R12C_2	GTCCGGAGAGGATGGCCCGG
PPP1R12C_3	GTGAACCAGGCAGACAACGA
PPP1R12C_4	TACGACCCAGAGCTCCGGGA
PPP1R12C_5	AATGAATTGATGCAGGTCTG
PPP1R16B_1	CATCCACTGCATGATCGCAG
PPP1R16B_2	GCTGGAGAAGGTGCCCACGC
PPP1R16B_3	GGGTGGGTTCATCCTCGCAG
PPP1R16B_4	GTGAAGCTGCTCCTCTCCCA
PPP1R16B_5	CTCGCTGAGGAACGACGCCG
PPP2CA_1	AACGCATCACCATTCTTCGA
PPP2CA_2	ATGGGAGATTATGTTGACAG
PPP2CA_3	GGTCAAGAGCCTCTGCGAGA
PPP2CA_4	TACAGCTCACCTTCTCGCAG
PPP2CA_5	AAAAGAATCCAACGTGCAAG
PPP4C_1	CAGGTCGCTGATCTCCGCCA
PPP4C_2	GACCAACTACCTCTTCATGG
PPP4C_3	TAGAGGAGAGCAACGTGCAG
PPP4C_4	TGAGGTCATAGAATTGTCCA
PPP4C_5	CGATCAGGATAGCGAACCTG
PPP4R2_1	CCGGCAGCACTGTACAGAAG
PPP4R2_2	ACTTCAGCTCCTGAGCCAAG
PPP4R2_3	CAGGCCACTTAATCGACCAA
PPP4R2_4	TGAGGAAACTTCTGATTCAG

PPP4R2_5	TTTGTTATTGCAGAATGTGA
PPP5C_1	GAGGTCATAGAACTGGCCAT
PPP5C_2	GATGTACGAGCTCTTTAGCG
PPP5C_3	TGAGTGCTATGGCTACGCGC
PPP5C_4	TTGCGGCATGGCGATGGCGG
PPP5C_5	AGCGGACCCAAGCTTGAAGA
PPP5D1_1	CCAAGCTCTCATTTCCGCCA
PPP5D1_2	GTTCACATGCCAGCCATCGA
PPP5D1_3	TACCCACCACCACCTTGCAG
PPP5D1_4	TGCAGGGAAGGTCGGACCAA
PPP5D1_5	CAAGGTGAAGCCCCACGACA
PRDM1_1	AGGCGGAGGCATCCTTACCA
PRDM1_2	GAAACACTACCTGGTACACA
PRDM1_3	GTCTACAGCAATCTCCTCGG
PRDM1_4	TTTGGACAGATCTATTCCAG
PRDM1_5	AGGATGCGGATATGACTCTG
PRDM10_1	ACTGGGAGCCAGACCCGCCC
PRDM10_2	CAACCGGCCGGTGCTCACCC
PRDM10_3	GCTGGTGTACATCCACCCGG
PRDM10_4	GGACATAAGCTACCTGGCCG
PRDM10_5	AGAGGATGCATAATCCTGAG
PRDM11_1	ATTCACCGCAACCTGGCCAG
PRDM11_2	GAGACCGGACTCCTCGGCCA
PRDM11_3	GTAGGGTTCAATGTTGAAGA
PRDM11_4	TGTGTCAGACACAAACACCG
PRDM11_5	GCTGCGGGTCTGGTACAGCG
PRDM12_1	ACTCACACAACACCTTCCTG
PRDM12_2	AGATGCCGAGGCCCTCGCCA
PRDM12_3	GAAGGACTGCGCCAGCACCT
PRDM12_4	TGTAGGTCATCCAGCTCCGG
PRDM12_5	CCGCGCGTACCTCCCACATG
PRDM13_1	AAGGCGGACGCCTCTTCCGG
PRDM13_2	GATGTCGAACCACTGAGCCA
PRDM13_3	GCAGCAGTCGGCACTCACGC
PRDM13_4	GCTATTGCAGACTTACCCGG
PRDM13_5	GGTCTCAAAGCCTATCCGGG
PRDM14_1	ACGGAGACAATTCTGTGATG
PRDM14_2	GAAGCGGGCACAGTTGACAT
PRDM14_3	GACTCCCTTCAACTTCCAGA
PRDM14_4	TAACAGAGAAGCATCCGCAG
PRDM14_5	AGCAGCAGCCACGAGTACGC
PRDM15_1	ACGACTGGAAGATGGAGCCG
PRDM15_2	ATGTCCCGAGCTGGGCCCAG
PRDM15_3	GTGCTTCGACACCTCCAACG
PRDM15_4	TGCGCAGCACCGGAAGCCGG
PRDM15_5	AAGCACAGCAGGAACGAGGT
PRDM16_1	ACACCATGCGATCCAAGGCG
PRDM16_2	CAAGAGGAAGATGGAAGCCG

PRDM16_3	CGTGGATGCAAATCAGGCGG
PRDM16_4	TTTGTGATGCAGCCTTCCTG
PRDM16_5	AGGACGTCAACACCACCACG
PRDM2_1	AGCATCGGGACGCCAAGCGG
PRDM2_2	GGCCACCGAGACCCTGGCTG
PRDM2_3	GTGGCAACAGTTAATACCGG
PRDM2_4	TGTGCTGCGAGGACTTCCGG
PRDM2_5	CCTCAAGTAGGTTTAAGAGG
PRDM4_1	CCAGTGGCAATTCCAAACCT
PRDM4_2	GAGGGAAACAGATTCTAGGG
PRDM4_3	GTGTGACCGCGCCTATCCCT
PRDM4_4	TGAAATGAACCTGAGTCCAG
PRDM4_5	CCATGTTCGGGACAGTCCGA
PRDM5_1	AAGGCCTTCAGCCAGAAGCG
PRDM5_2	AATGCTGGGCATGTACGTGC
PRDM5_3	ATTGCACCAGAAACCCACAG
PRDM5_4	GCACCAGGAGACTTGCCGGG
PRDM5_5	ATGAACGAAGCGAAGCCAGT
PRDM6_1	ACGGCGAGTGCCCTATGCAT
PRDM6_2	CGAGAGACCAGCCAGCGCGG
PRDM6_3	CTGTGGTCGTGCCTTTGCCG
PRDM6_4	GCATCTGTAAGAGGATCCGC
PRDM6_5	CCTGGGCAATGCATTGTAAG
PRDM7_1	TAGCCAGGAATATCCACTGT
PRDM7_2	TCTCCTCTGAAGGCCATCCA
PRDM7_3	CCGATGAAGAATGGACACCT
PRDM7_4	TAGTCTAGAGTGCTGTCCAG
PRDM7_5	TATAATGCACTGATTACTGT
PRDM8_1	AGGAAAGAGGAAATTCCCGG
PRDM8_2	ATTCTCAGGGATGTCGCAGG
PRDM8_3	GAGGATACTGGCATCCAGCG
PRDM8_4	TGTTCTACCGCTCTCTCCGC
PRDM8_5	ATAAGTCCCCAAGACGAACA
PRDM9_1	CAACAATGGATACTCCTGGC
PRDM9_2	GTGTCCTCTGGTGATAGCAG
PRDM9_3	TCCACTCTTAAGGCCATCCA
PRDM9_4	AAAGGGTCATGCATACAAAG
PRDM9_5	ACTCTTAAGGCCATCCAAGG
PREB_1	CTGGAGTTCAAAGCCCACGA
PREB_2	GACAGGCATAAAGAATGGCG
PREB_3	GGAAGCGCAGGAGCTGACAG
PREB_4	GGAGCGGAAACCCAGCACGA
PREB_5	AGGAGCAGGGGCCTCGACAA
PRKAA1_1	AGAATGGTACTCTTTCAGGA
PRKAA1_2	GAGGTAGATATATGGAGCAG
PRKAA1_3	GCAACTATCGATCTTGCCAA
PRKAA1_4	GTGATGGAATATGTCTCAGG
PRKAA1_5	ATCACCATGAAAATATCAGA

PRKAA2_1	GAAGTTGATATCTGGAGCTG
PRKAA2_2	GATGATAAGCCACTGCAAGC
PRKAA2_3	GTAATGGAATATGTGTCTGG
PRKAA2_4	TCAACTCTTCACTTTGCCGA
PRKAA2_5	ATGACGTTAGCATCATAGGA
PRKAB1_1	GAATGATAAAGCTCCCGCCC
PRKAB1_2	GGACAGGCCCAAGATCCTGA
PRKAB1_3	GTGGACGCACGACCCTTCCG
PRKAB1_4	TATGGGCTTGTATAACAAGG
PRKAB1_5	AACGGTGTTTCGATGGACGG
PRKAB2_1	ATTGGTGCTCTCCCTCAGGG
PRKAB2_2	GCCAAGGCTGCACGCTCCGA
PRKAB2_3	GTCGCTGGTGGTGTTTCCCA
PRKAB2_4	TTCAGACCAGCGGATAACAG
PRKAB2_5	CAAAATCAGATTTCTTGACA
PRKAG1_1	CAACAGGCTAGTGGAAGCAG
PRKAG1_2	GCAGCGATGAGACTTCATGA
PRKAG1_3	TCGAGTCTCAGCCCTGCCAG
PRKAG1_4	TTAGGAGAAATGCAGACAAG
PRKAG1_5	CAGAGCCACATAGACGGGGG
PRKAG2_1	AAGCTGGAGTTCGAGGACGA
PRKAG2_2	GTCCAGGAGCGGCATGGCGA
PRKAG2_3	TAGTGTGTCGGTGATGCCAG
PRKAG2_4	TGGAGAAGAACCCTTTGGAG
PRKAG2_5	ACTGAAGCTCATGCGTCGAG
PRKAG3_1	GAATGTGGCCTCCAGCCCGG
PRKAG3_2	GCTGGAGCACGCACTGCGCA
PRKAG3_3	GGAGGGCCAAAGCCTTGAGA
PRKAG3_4	TTAGGAGAGATGGAGACCAG
PRKAG3_5	GCAGCTCAGAAAGAATCCGT
PRKCA_1	AAACAAGGCTTCCAGTGCCA
PRKCA_2	GAGGCAGAAGAACGTGCACG
PRKCA_3	GCTCCACACTAAATCCGCAG
PRKCA_4	GCTCCGAAACTCCAAAGGAA
PRKCA_5	AGGAAGGAAACATGGAACTC
PRKCB_1	ACGGACCACACGGAGCGCCG
PRKCB_2	ATGCCCATTACTTACATCGG
PRKCB_3	GCTGTATGGACTCATCCACC
PRKCB_4	GTAGGGATCTGACAGGCCAT
PRKCB_5	CAGAGGGCCAAGATCAGTCA
PRKCD_1	CTGAACAGACATCAACACCT
PRKCD_2	GATGCGCAGGAACGGCGCCA
PRKCD_3	GGCCAGCACCGACACACCCA
PRKCD_4	TTCCCAACGATGAACCGCCG
PRKCD_5	ATCTCTCGGGCAGACAACAG
PRKDC_1	CTCCATAATCCGGACCACAA
PRKDC_2	GCAACATCAGAATACTATGG
PRKDC_3	GCACATCATCATGCACCGTG

PRKDC_4	GTTCTCAGAAACGATCAACA
PRKDC_5	AGAAGGAATTCACTACATGG
PRM1_1	ACAGCATCTGTACCTGGCCA
PRM1_2	GAAGTCGCAGACGAAGGAGG
PRM1_3	GACAAAGAAGTCGCAGACGA
PRM1_4	GGCGGAGCTGCCAGACACGG
PRM1_5	GCTGCGACAGCATCTGTACC
PRM2_1	CACCGGAGGAGGCATCGCAG
PRM2_2	GACGCTCCTGCAGGCACCGG
PRM2_3	GCAGACACTGCTCTCGAAGG
PRM2_4	GGGCAAGAGCAAGGACACCA
PRM2_5	AGCACGTCGAGGTCTACGAG
PRMT1_1	AAAGCCAACAAGTTAGACCA
PRMT1_2	CAGTGAGAAGCCCAACGCTG
PRMT1_3	GAATGACTACGTGCACGCCC
PRMT1_4	GCAGTGGTGACCATCATCAA
PRMT1_5	GATGGCCGTCACATACAGCG
PRMT10_1	AAGATGAGTCGAGTTCCTGG
PRMT10_2	ATCATCAGGAAACAGTTCCA
PRMT10_3	GAGCAGGAACTGGAATACTA
PRMT10_4	GGAGCTGAAACACGACGTGA
PRMT2_1	AAACCACTGCAGATTGGTGG
PRMT2_2	AGTGAGATGGCACAGCACAC
PRMT2_3	GGTGGCAGCGTAGTCCGCGA
PRMT2_4	TATTCTGCAGGATGACACTG
PRMT2_5	ACCCCACCACCAATCTGCAG
PRMT3_1	CCTGTTACAGAACAGGCAGG
PRMT3_2	GAACCTGTTACAGAACAGGC
PRMT3_3	TATGAGAAGGGTACTGACAC
PRMT3_4	TGTCATTGCGGACCTCCAGG
PRMT3_5	CCAACATCCAAAACTACCTA
PRMT5_1	ACACACAGAGGAGTACAGTG
PRMT5_2	GATTCGCAGGAACTCCGAGG
PRMT5_3	GGTGGGAGCCGCGTGTCCAG
PRMT5_4	TCAGGAAGATAACACCAACC
PRMT5_5	GAAGATTCGCAGGAACTCCG
PRMT6_1	AGGCGAGGTGGACAGCACCA
PRMT6_2	GAAGACCAAAGACTTTGCCA
PRMT6_3	TGTAGCGCAGCAGCACGCGC
PRMT6_4	TGTGCAGGGATTGTCCGGCG
PRMT6_5	CGTTCCCGCTTAGTCCTCCG
PRMT7_1	AAGGGAATGGCTTACCTCGA
PRMT7_2	ACATGGGCAGCACATCGCTG
PRMT7_3	GTCGGGCCAATCCGACCACG
PRMT7_4	TGGACCTACAGTCACCTCGG
PRMT7_5	CGCACACAGGCATCTCGTGG
PRMT8_1	AGAAGACGGTCTGCTTCCAG
PRMT8_2	CAACACGGTGATCTTTGCCA

PRMT8_3	CAACGACTACGTCCACGCCC
PRMT8_4	GTCCAAGCTGCTGAACCCAG
PRMT8_5	AGACCGGGCAGCTTTGTACG
PRMT9_1	CAAACTGGTTGGTGGAACGC
PRMT9_2	TGAAACACGACGTGAAGGTG
PRMT9_3	TTCCAAAACATATTCCCGAA
PRMT9_4	TTTATCCACAAGTCCTAGTG
PRMT9_5	ACTGGTCTGAAGGTTAGCGA
PROP1_1	ACTTGGATGGCTCAAGGCTG
PROP1_2	AGAACCGCAGAGCTAAGCAA
PROP1_3	AGACTGGTGTGACAAAGCAA
PROP1_4	GCAGGGCAGATGGCCGGCAG
PROP1_5	AGATCAAGGTTCTCCCCGCA
PROX1_1	GACCTGAGGAACCTGGCGGG
PROX1_2	GCACGTCAAGCCATCAACGA
PROX1_3	GGTGGACGTGGTGAAGCCCG
PROX1_4	TTGACTGCAAATCTGGCCTG
PROX1_5	CCAAACTCCTTACAACCGGA
PROX2_1	AGGATCTGAGTCTCTCCCTG
PROX2_2	AGTCAAGGGAAAGTTTGCTG
PROX2_3	GAACCACTTGATCATCTGGG
PROX2_4	GACACTGAGATAAATCCAGG
PROX2_5	AAGGTACTATTGGATCCACC
PRPF31_1	AAGCAAGCCAAAGCTTCAGA
PRPF31_2	ACTATCCCATAGCTTGGCGA
PRPF31_3	GCGGATGTAATCCAGTGCAT
PRPF31_4	GGATGCCAACAACCTGACCG
PRPF31_5	CGTTGCTCCAGGATCTGCGG
PRPF38A_1	AAGAGCCAGAACCGAAATGG
PRPF38A_2	ACTTGTAGTCGATAAAGCCA
PRPF38A_3	AGTCTCGGTAGCTTCTCCGG
PRPF38A_4	TCTTCCAGAGCACTAACTCG
PRPF38A_5	GAATCTATGAGTCCAAGTAC
PRPF8_1	ATAGAACCAGTCCAACACAG
PRPF8_2	GCTACCGGACTACATGTCGG
PRPF8_3	GTCATGGCGGAACTTCCTGT
PRPF8_4	TAGACAGGTTCAATGACCCA
PRPF8_5	GACGGGTGCGACCAGAGCGT
PRR12_1	AAGCCCTTGAGGCTGACCGG
PRR12_2	GACACTGGCCTCCACCACGC
PRR12_3	GAGGGTGGCACACTCTTGGG
PRR12_4	GGGCGGGATGGAGTTACGAG
PRR12_5	AACTCGTAGGGTGTACCCGG
PRR14_1	ACTTCATCATCCCAGCACAA
PRR14_2	CAGTTCCCATACCTCAGCGG
PRR14_3	CCAGGAGCCCGAAACGGCCG
PRR14_4	GTCTCCCATGGACTTGCCCG
PRR14_5	CAGTCCTAGACCCCCCGCTG

PRRX1_1	CCGCCGGGTGAACCTCACCG
PRRX1_2	GAGTCGCCGGGACTCACCAG
PRRX1_3	TAATCGGTGGGTCTCGGAGC
PRRX1_4	TGTGGCAGAATAAGTAGCCA
PRRX1_5	CGGCAGCGACACCCCGCAGC
PRRX2_1	ACAGTGCCACCCTACAGCCC
PRRX2_2	AGCGGAAGAAGAAGCAGCGG
PRRX2_3	ATAATCTGGACTCAGGGCGG
PRRX2_4	GGACCTGGAAGAGGTGGCGG
PRRX2_5	CAGGTGAGTGTCCCAGCCCG
PSIP1_1	AAGTGAAGCAAGTTCATCCA
PSIP1_2	GAAGCCAGAAGTTAAGAAAG
PSIP1_3	GGGTCAAAGACTCTAAATGG
PSIP1_4	TAGGTAGACGAAGTTCCTGA
PSIP1_5	AAAGGAAACTAGTGTTTCAA
PSMD10_1	AATGCACCGGGCAGCAGCCA
PSMD10_2	ACAAACATCCAAGACACTGA
PSMD10_3	GCAGAAGCCGCAATATGAAG
PSMD10_4	GCTGGGACAGCGAAATGGAG
PSMD10_5	AGATGCTAAGGACCATTATG
PTCD3_1	AAACACATAAGGCACAGCTG
PTCD3_2	GCTGGCCAAGCCTGCTGCGG
PTCD3_3	TGGTAGTGCAACCCTCTCAA
PTCD3_4	TTTACTGGCAAAGAAATCCG
PTCD3_5	CTGATCAAACATGTCCACAG
PTF1A_1	GGCGGCAGTACTGTCTCCCG
PTF1A_2	GGTGAGCCAGACGACGGCGG
PTF1A_3	GTGGCTAAGGAACTCCACCT
PTF1A_4	TAGCAGGACACTCTCTCTCA
PTF1A_5	CCTGGCCTACCCGTGCGCCG
PURA_1	AGCGGAGCGCAGCATCATGG
PURA_2	ATCATGGCGGACCGAGACAG
PURA_3	CGAGGGCACCTCCTTGACTG
PURA_4	TCCGCCAGACGGTCAACCGG
PURA_5	CCTTACTCTCTCCATGTCAG
PURB_1	CAAACGGTCAACCGCGGCGG
PURB_2	CCTGTATGGAGAGCTCCCGG
PURB_3	GAACTCGGCGGCCACCGCCA
PURB_4	GAGGACGACGAGCTGGCAGG
PURB_5	AAGCTCATAGACGACTACGG
PWWP2B_1	GAACGGCGAGCCCACGGCTG
PWWP2B_2	GGACCATCAAGCGCACGCGG
PWWP2B_3	GTATCGGAAAGCTATAACCG
PWWP2B_4	TGTCCCGCAACCGCGACCCG
PWWP2B_5	ACAGGGGACTCATCGACCTG
PYCARD_1	ACCGGGCTGCGCTTATCGCG
PYCARD_2	CCGGGCTGCGCTTATCGCGA
PYCARD_3	GATGGCCGGGCAGCTGCAGG

PYCARD_4	GTAGGACTGGGACTCCCTTA
PYCARD_5	AGCTTCTACCTGGAGACCTA
PYGO1_1	AAAGCGCAAGGCAAATACAC
PYGO1_2	AATAAGCCACGACAACCAAG
PYGO1_3	GGAGGACCAGGTGTACAACT
PYGO1_4	TCAAATCCATATCTTGGCCC
PYGO1_5	CAAAATTAGATGCCAAATCG
PYGO2_1	ACATGGGTACACCAAGCCTG
PYGO2_2	CCTCCCTCCAGCTTGTCCGG
PYGO2_3	GGGAAAGTTCATGTTGCCTG
PYGO2_4	TCATGCCTGTGCACTCACGG
PYGO2_5	AAAACTTTAGTCCTCCCAGT
RAD18_1	AGGCCACCGAGACTCGGCCA
RAD18_2	ATTGGAACCTGACAGAGAAG
RAD18_3	GACAGCAAGATTCTTTGAAG
RAD18_4	TAGGATGCAAAGCATCGCAT
RAD18_5	AGTGGATTGTCCTGTTTGCG
RAD51_1	AGAAGCTGGATTCCATACTG
RAD51_2	GCCAGAACGGCTGCTGGCAG
RAD51_3	GTCGAGGTGAGCTTTCAGCC
RAD51_4	TTGGTGGAATTCAGTTGCAG
RAD51_5	ATACCTAGATTCTACCATCA
RAD51B_1	AAGCCAGGGCTCCACTCAGA
RAD51B_2	AATGAGGTGAAGGGAGCCAG
RAD51B_3	GGGAGGATTAGAAGGAGCTG
RAD51B_4	TTGGACGAAGCCCTGCATGG
RAD51B_5	ACATTACCCACCAACATGGG
RAD51C_1	AAACCCTCCGAGCTTAGCAA
RAD51C_2	CTTGAGCAGGAGCATACCCA
RAD51C_3	GAAACGGTCACGAGACCCAG
RAD51C_4	GTACAAGTCACCCAGCCAGA
RAD51C_5	AGAACACCGAAAAGCTTTGG
RAD51D_1	ACTGTGCCCTGGCCTTACCG
RAD51D_2	AGAGATCAGCGCCATTCACG
RAD51D_3	GAGAAGCTGGATCATCTCCT
RAD51D_4	TAGCCTGAAGCAGCTGGAGG
RAD51D_5	CACAAACCTGCCAATGCCAG
RAD52_1	ACGGGCTGAAGCGAGCCCTC
RAD52_2	ACTGAGGAAGCAATTCTTGG
RAD52_3	AGAGTACCAGGCCATCCAGA
RAD52_4	CAGCCATGCTGTGATACCGG
RAD52_5	AACAATGGCAAGTTCTACGT
RAD54B_1	CCTGGATTACTTCTTCCTGG
RAD54B_2	GAAGGCAAAGACATTGGAAG
RAD54B_3	GCAGTCTTACCTGTATGAGG
RAD54B_4	TCTCCAGATGACTTCAGCAG
RAD54B_5	GCTCCTATACATATTAGATG
RAD54L_1	AAGGAGACAGGAAACACTCC

RAD54L_2	GAATAGGGTCAACAACCACA
RAD54L_3	GAGGTTGGGAAATGGCTCGG
RAD54L_4	TGGTCATGAGCGCTCAGCGG
RAD54L_5	ATTACAAAGCTTCTTTAGCG
RAD54L2_1	AAGATGGGCAGCAGCCGCCG
RAD54L2_2	ACTTCCTGGGCCAAGACCCG
RAD54L2_3	GTTTAGAAAGCTACTCCGGG
RAD54L2_4	TGTCAGACGAATCTGCCTCA
RAD54L2_5	ACAAGGCGATAGATGTAACA
RAG1_1	ACATCTGCAACACTGCCCGT
RAG1_2	ATAGCGGAACCTCTTTGCAA
RAG1_3	GAAGACACATTCTTCAGAGG
RAG1_4	TAGTACCAAGCTCCTTGCAG
RAG1_5	ACACCAAAGCTGAATCATAG
RAG2_1	AACATCAACAGAAGATCCAG
RAG2_2	ATGAGAGCAGTAGATCATGG
RAG2_3	ATTAATGTGGTGTACAGCCG
RAG2_4	ATTCTTCCAGAACTTCAGGA
RAG2_5	ATAAGGGTTGATCTTCCCCT
RAI1_1	AGGTGGCCAAGGCTGACCGG
RAI1_2	CAAAGAAAGAGCCTGTGCCA
RAI1_3	GAAGGTGGCCTCGTTGCCCG
RAI1_4	TTCCAGCTGCCAAGAAGCCG
RAI1_5	AACTTGGAGAAGCTGTCGAG
RAI14_1	AAAGGAAATACCTTGAAGGG
RAI14_2	ACCTGGGTAGGACTGATAGG
RAI14_3	GATTGGCAGCTCTAACGCTG
RAI14_4	TGACCGGCTACTGCAGGCCG
RAI14_5	AGAAACTCAGAGCAAATACG
RALY_1	ACCAAAGGGACTGTGACCCG
RALY_2	ATAGTCAAAGATGTAGCCAC
RALY_3	GCACAGAACAGCCGGCCACA
RALY_4	GCCTAAGCCTGACAGACCCA
RALY_5	CACAGGCTCTTCGACTACCG
RARA_1	AGAGTGGTCAGAGCGCCTGG
RARA_2	GAGGGTGATCTGGTCGGCGA
RARA_3	GCAGTCGGCAGTACTGGCAG
RARA_4	TGAGAGCTACACGCTGACGC
RARA_5	AAGCAAGGCTTGTAGATGCG
RARB_1	CAGGCAGGCGGCCTTCAGCA
RARB_2	GCAGGGTTTGTACACTCGAG
RARB_3	TAGAAATCCAGGATTTGCCC
RARB_4	TAGCATCTCACCCAGCTCAG
RARB_5	AAGCAGGGTTTGTACACTCG
RARG_1	AAAGAGTCGCTCCTTATTGG
RARG_2	AAGCATGGCTTGTAGACCCG
RARG_3	GCTACAGAAGTGCTTCGAAG
RARG_4	GTTAGAAGAGCTCATCACCA

RARG_5	GGGCTCAGCATCTCGAAAGG
RASD2_1	AAGGCGCTTGACCTCATCGA
RASD2_2	ACTGAGCGTGCAGTTCCCGC
RASD2_3	CACGGGCCTGGCCTTCCCGA
RASD2_4	GCTGGACAAAGTCTTCATCA
RASD2_5	CAAAAACTCATACCGCATGG
RAX_1	GGCAAGCGAGAAGCTCCCGT
RAX_2	TGACAGTTTCGCTTCGCCGG
RAX_3	TGCGCGCCAGCCATGGCCGA
RAX_4	TTCAGGAAGGGCGGAGGCGG
RAX_5	CGGCAAGGTCAACCTACCAG
RAX2_1	ATGTTCCTGAGCCCGGGCGA
RAX2_2	CAGGGCGAAGCCATCTGCGA
RAX2_3	CCGCAGGGACGCCTCCTCCA
RAX2_4	GGAGCCATGTTCCTGAGCCC
RAX2_5	CCTGCACGCGCACCTCAGGT
RB1_1	AGAGCAGGACAGCGGCCCGG
RB1_2	GAACAGATTTGTCTTTCCCA
RB1_3	TCACCTCGAACACCCAGGCG
RB1_4	TTGGGAGAAAGTTTCATCTG
RB1_5	AAACAATCAAAGGACCGAGA
RBAK_1	AAGTGGCTGGTGCCCATCGA
RBAK_2	AGAGGAATCATTCAGGAGAG
RBAK_3	AGGGAACCCTCACTGTACAT
RBAK_4	GGGCACCAGCCACTTCCAAA
RBAK_5	GACTTCTATAATGTATAGTG
RBBP4_1	ATTGCCGTTACAGACCAGAA
RBBP4_2	CATTTAAGACCATCTGCCTG
RBBP4_3	GGAAGAACGAGTGATCAACG
RBBP4_4	TAACATACCAACAACTCTGG
RBBP4_5	CACTACGACAGTGAGAAAGG
RBBP5_1	CAAACATGTTGTTCTGCCGG
RBBP5_2	CTTTGGGCAGAACTATCCAG
RBBP5_3	TGACTTGCACCTTTAACAGG
RBBP5_4	TTTCAGGCGACTGTGACCAG
RBBP5_5	AAGATTCTCCATGGGACGAG
RBBP7_1	CAAGTGACTCGGCTTGGAGG
RBBP7_2	TGATGAGCAGAATCATCTGG
RBBP7_3	TGTCTGTGGGATATAAACGC
RBBP7_4	TTAAGAGGTCACCAGAAGGA
RBBP7_5	AGCCACTGAACGGTAAGACT
RBL1_1	ATTTGGGCACATAATCGCAT
RBL1_2	CAGGAGCTGAACCTGGACGA
RBL1_3	GCCAGGGAGAAGTTACACAC
RBL1_4	TACCACGAAGCCGGAAGCAG
RBL1_5	AATTTCGTGAACGTATAGAA
RBL2_1	CATGAGCGAAAGCTACACGC
RBL2_2	GAGCAACCTCGTCAGCAGCG

RBL2_3	GTAGAAACTGGAGTCACACA
RBL2_4	TCTGTTCCAACTGTAAGCAA
RBL2_5	CAGGCGTCCCTCCATCAGAG
RBM10_1	ATCTGAGGAGCAGAGTGCGG
RBM10_2	CAAGTGACTAAACTCGACGA
RBM10_3	CCGAGACTCAGCGTAGGCGG
RBM10_4	CGTGCGACTGCAGCTGGCCA
RBM10_5	CACCCACCACTCACTATCGA
RBM12B_1	GAGCGCCTGAAATGCTCCTG
RBM12B_2	GGCCGACGGAAATGCTCCTG
RBM12B_3	GGTCTCCGGAAGTGCTCCGG
RBM12B_4	TGGCCGCCGGAAGTGCTCTG
RBM12B_5	AGTCTTATGTAAACTCAGAG
RBM14_1	AATGGGCATCGGGCAGGCGA
RBM14_2	CAACGTGGAACTCTCCACCA
RBM14_3	GAAGGGCAAGCGCATCAACG
RBM14_4	GCTGCGACTCTGATAAACGG
RBM14_5	CTGGGCTCCATAAGCTAGTG
RBM28_1	AAAGGTGGTAATCTCCTTGA
RBM28_2	AGTGGCTGTGGATTGGGCCG
RBM28_3	GGAGGAACTGTTCAGTCAGG
RBM28_4	GTGCCCAAAGAAGGAGCCGA
RBM28_5	AAAAAGCAGTGATCATTCTG
RBM3_1	AGTAGCTGCGACCACGCCCA
RBM3_2	CATGAGAGCCATGAACGGAG
RBM3_3	GAAGGAAAGCTCTTCGTGGG
RBM3_4	TATTATGACAGTCGACCTGG
RBM3_5	AAGGAAAGCTCTTCGTGGGA
RBM5_1	AAAGATCTAGTGATGATCGG
RBM5_2	CTGTCTCTAGGTGTAAGCCG
RBM5_3	GAAATGATGGAGTCCTTCGA
RBM5_4	GAAGATGGCTACCATTCAGA
RBM5_5	AGATCTAGTGATGATCGGAG
RBM7_1	ATCAGTCTTCAAGCTCCCAG
RBM7_2	CCTTGAAACGAAAGTGACCG
RBM7_3	GAACCCAGCCGCTTACCTGG
RBM7_4	GGGTCTGACCATCATTACAG
RBM7_5	CTCAAAGCACTGTTCATCTG
RBMX_1	CCTTAAGCTCCTGTATCACG
RBMX_2	AAATATGGACGAATAGTGGA
RBMX_3	ACATCTCTACGAGAGGGCAG
RBMX_4	AGAAGTAGAGGCCCTCCAAG
RBMX_5	CTCTAGTATCACGAGAACTT
RBMXL1_1	TCTCTACTCAAGTTGTGACA
RBMXL1_2	AATAAACATCTCTACGAGAG
RBMXL1_3	AGGGGACCTCCTTCACGAGG
RBMXL1_4	CTGCTGCGAACTAGTCCTGA
RBMXL1_5	TCATTTGAAAGAGGTAGACA

RBPJ_1	AAAGAACAAATGGAACGCGA
RBPJ_2	CGAATCAAACACAAACAGCG
RBPJ_3	GAATTGCAGTTGAATGGCGG
RBPJ_4	TTTGCAGTATGCATTGCCTC
RBPJ_5	CACCTAGTAAGTCGTTTAGG
RBPJL_1	AGAGCACACCACCATTCTGA
RBPJL_2	AGAGGTAGACACAGGGCGGG
RBPJL_3	TCCCATGTAACCGCAGACCG
RBPJL_4	TGCAGAGCGAAGCCGACAGG
RBPJL_5	CGAACGTAGCCCTCTCGCGG
RBX1_1	AACACGACAGGTGTGTCCAT
RBX1_2	AATGGCGGCAGCGATGGATG
RBX1_3	GAGCGGCACCAACAGCGGCG
RBX1_4	TGAGCCAGCGAGAGATGCAG
RBX1_5	AGAAGAGTGTACTGTCGCAT
RC3H1_1	CAAATGGGCAAGCCTTGCGG
RC3H1_2	TACGAATTGCACCGGACCAG
RC3H1_3	TTAGAGGCTTGAGGAAACCG
RC3H1_4	TTCTGAGTCGCCCAATGCAG
RC3H1_5	CTGGATTCTGGTACATACGA
RCBTB1_1	CATGGATGAAGTCTGACCAC
RCBTB1_2	GATGGAGTGGTTTATGCCTG
RCBTB1_3	GCAAAGCTGCCACTCTCACA
RCBTB1_4	GGAGAAAGGCACGATACACT
RCBTB1_5	ATTTACTTGCAGATTGTCTG
RCC1_1	CCTGAGGCCACCTTTACCAC
RCC1_2	CTTGCAGCTCCACTTTCCCA
RCC1_3	TGATGGTGACCTCTACACCT
RCC1_4	TTGGTGCTGACACTAGGCCA
RCC1_5	AGAGGCCGAAGCCGTACACG
RCL1_1	ACTTCGCCTCCTCCTCCGGG
RCL1_2	CCACAGATCCACCATACAGG
RCL1_3	GGGTAGACAGGACCAGACGT
RCL1_4	TGGAGGCCAGTTCAGCACTG
RCL1_5	ACCGGATTGTGGATTCTGCA
RCOR1_1	AGAACCATATCGACTTCCAG
RCOR1_2	GCGGAGAGGGAGGAACAACG
RCOR1_3	GTCTAGTTGTCTCAGCACCG
RCOR1_4	GTGGTGCCCGACTTCGACCC
RCOR1_5	ACGGGACAATCTTGGCATGT
RCOR2_1	ACAGACAGCATGATCCGCGT
RCOR2_2	AGTTGACAAGTACATTGCGA
RCOR2_3	GTGATGGAGAAGCCGAGCGC
RCOR2_4	TCCGCTGGAAGCATTTGCCA
RCOR2_5	CCGCAGCCGAACTAGTGTGA
RCOR3_1	AGCGCCAAGAGCCCGGCAGG
RCOR3_2	GAAATTAGGGAGATCAGCAA
RCOR3_3	GAAGAAACACATCCAATGGA

RCOR3_4	TAAGGGCATGTATTTAACCC
RCOR3_5	CTAGCTAATAGACATAATCA
REC8_1	GCTCTGTAGGAACTTCAGGA
REC8_2	GGTGAAGCGCGAATACCTGA
REC8_3	TCAACAATGCCAGTACCTCG
REC8_4	TGAGGCCATCACGATCCTGG
REC8_5	AGAACAGTAACCGGCGACGA
RECQL_1	ACAGCAGAAGCTTTATGAGA
RECQL_2	GAATTGATAAGCCAGATGTG
RECQL_3	TACCCTGAAAGAGTTCTGCG
RECQL_4	TTTAGAGGATTCTGATGCCG
RECQL_5	AGAACTAGAAGCATTTAACA
RECQL4_1	AAGCTGGACCCCCATCGGAG
RECQL4_2	ATGACAGGGGCAATTACGTA
RECQL4_3	CCTGCCACCGTGTCTCAAGG
RECQL4_4	GTACAGCGAGCCTTCATGCA
RECQL4_5	TGGTGCCACAGTCACAACCA
RECQL5_1	AGTCAGCTTCCTGATCAGGA
RECQL5_2	GGACACCAGGGAGTTCAGGG
RECQL5_3	GGTGCAGAACGACTGGATGG
RECQL5_4	TTCTTCACAGAAGGTCACCA
RECQL5_5	GACCCCGAGCTGTATGAGGG
REL_1	ATTGGGTTCGAGACAACAGG
REL_2	GCAGGAATCAATCCATTCAA
REL_3	TAATTGAACAACCCAGGCAG
REL_4	TGTGAGAGAAATGCCTACAG
REL_5	ATGTGACAATCCACTTGAGA
RELA_1	AAAGGACAGGCGGCAGGCGG
RELA_2	ACGCACAGGAGCCTGCAGGC
RELA_3	ATGAAGAAGAGTCCTTTCAG
RELA_4	TCAATGGCTACACAGGACCA
RELA_5	ACTACGACCTGAATGCTGTG
RELB_1	ACTCGGCATGGCCCGGCCAG
RELB_2	CAAGGAGAACGGCTTCGGCC
RELB_3	CGAGAGTGAGGAGAGGTCGG
RELB_4	GCAGGGACAGATGCGCCGGA
RELB_5	ATTGAGCGGAAGATTCAACT
REPIN1_1	ACAGCGATGGGCCCTGCCTG
REPIN1_2	AGATTCACAAGCGATCCGAG
REPIN1_3	GAGGCAACAAGGCACGTCAG
REPIN1_4	GTGGCCGGAGCTTTGCCCAG
REPIN1_5	AGCCCTATCTGACTTCGCAC
RERE_1	CACGTGGCGAATCATGACAG
RERE_2	GGAGCGGGAGATCCGAGAGC
RERE_3	GGAGGCTGAACAGTACCCAG
RERE_4	GTGTGATGGAGGCTCTACAG
RERE_5	CAGAGTGGTGTCATCCCGAG
REST_1	AAAGGGTGACAGCAAAGTGG

REST_2	ATCTGAGCAGGCTCCACGGG
REST_3	GAAACACCACACCAGAGCTG
REST_4	GATGCCTCAAATACAGGCTG
REST_5	AACTCGAAAATCAGTAACAG
RFC4_1	ACAGATAAGACAGAATCGGG
RFC4_2	AGAAAGCCAAACCCGTTCCC
RFC4_3	GAAAGCCAAACCCGTTCCCT
RFC4_4	GTTGACAAATGCCTAGCAGA
RFC4_5	ACAATCTTAAAAGGCGGACA
RFC5_1	CTTCCACGACATGTTCCAGG
RFC5_2	GCACTAGTCACTCTTTCCAG
RFC5_3	TAGATGTCTTGCCTGTCCCT
RFC5_4	TGAAGCAGACGCCATGACTC
RFC5_5	CGAACCGAAACCTCGTGCAG
RFWD2_1	AAGCTCCTTCTCCATCACAC
RFWD2_2	AGTGCAGAAGAAGAAACAAC
RFWD2_3	GCAACTGGAACAGATCCAGA
RFWD2_4	GCCGCCGCTACCCGATACGG
RFWD2_5	ATCACTAGCATATGACAATG
RFX1_1	AACGCCGGTCTGGCTGGCGG
RFX1_2	ACAGGACCACCACTTACCTC
RFX1_3	GCTGGAGTACACGTGTGGGA
RFX1_4	TGGCCAGAAGCAGTACGTGA
RFX1_5	GAAGTCAGGGAGGCTCCGAG
RFX2_1	CTGAGCATGCAGAATTCCGA
RFX2_2	GATGAGATAGGCTCCCGCGC
RFX2_3	GCAGCACGTGTATCCTGCCC
RFX2_4	GCAGCCAGCTCCAATCCCAA
RFX2_5	AGCCAGCTCCAATCCCAAAG
RFX3_1	ACAGGAAGTGGTCAACAGAC
RFX3_2	AGCGATACTGTCTATACCAA
RFX3_3	ATTCAGATGGGCGTCACAGG
RFX3_4	TCAAGCAGCAGTGCCTACGC
RFX3_5	AGGAATAGGTCTAAGGACGT
RFX4_1	ACCAGAAGACTCGGGACCCG
RFX4_2	ATTCCAGCCACACATCTCTG
RFX4_3	GAACCCGACATGGATTCCAC
RFX4_4	GGAGAACTATGAGATTGCAG
RFX4_5	AATAAGAGCCAACTTTGATG
RFX5_1	GAAGCGGGCGACCTCAACGA
RFX5_2	GCTCGAAGGCTTGGTGGCCG
RFX5_3	GTGTTTATGATGCCTATCGG
RFX5_4	TTTAGGTCAAGTCCAGGCAG
RFX5_5	GGATCTGGAAGCCCGAACTG
RFX6_1	AGACTAGCAAGCCCTTGCCC
RFX6_2	ATACAAGGTGTTGAGCGCTG
RFX6_3	GTGAAGGAGTTTGCTTACCA
RFX6_4	TGAATACATTCTCCTGGCCA

RFX6_5	CCATAGAATTAACGACATGC
RFX7_1	AGAGCGAATGGTGAGTGCAC
RFX7_2	GCAGCTTTAATCCAAATGGA
RFX7_3	GTCCAGGAGCAGCAGTGCGG
RFX7_4	TATCATCCAATCCACAGCAA
RFX7_5	ACAACGATACCAATAGGTTG
RFX8_1	AAAGACGTACCTTTCCAACA
RFX8_2	GCCCTGGCAAGTGACCGGAG
RFX8_3	GGGCCAGGGCCGATATCCCG
RFX8_4	TCAACAACTTACCTTCCCAA
RFX8_5	AAATACTCCTGTAATATGGT
RFXANK_1	AACGGTCTCAATCTCTCCAA
RFXANK_2	ACTCTCACCAACCGGCAGCG
RFXANK_3	AGAGGAGGTATCCTACCCTG
RFXANK_4	GCGAGAGAGCGCCCTGTCGC
RFXANK_5	AGGTATCCTACCCTGTGGAG
RFXAP_1	AGGACGAGGAGACTCACTCG
RFXAP_2	CAAGGTGGGAGCCGCAGCCG
RFXAP_3	GACGAGGAGACTCACTCGGG
RFXAP_4	GGACGAGGAGACTCACTCGG
RFXAP_5	ACACCGCAACAAGATGTACA
RHOXF1_1	CATGGGTAATATGAACCCTG
RHOXF1_2	GCAGAAGGCCATGTTGGCCA
RHOXF1_3	GGTAATATGAACCCTGAGGG
RHOXF1_4	GTGGACGAGCGAACGCGCCA
RHOXF1_5	AGAACATGCAGCCACGAACT
RHOXF2_1	CCTATGGGAAGGAGACCTCG
RHOXF2_2	GACGACGAGAAAGAACTACA
RHOXF2_3	GCTTACCGCAGGAACTCACT
RHOXF2_4	TTGAGAATAGAAGAGCCAAA
RHOXF2_5	CTATGGGAAGGAGACCTCGA
RHOXF2B_1	CCTATGGGAAGGAAACCTCG
RHOXF2B_2	GGTGCTGTCGCTTACTGAAG
RHOXF2B_3	TGTGACTGAACTCGCAGTGC
RHOXF2B_4	AGTTCCCCAGTGAGTTCCTG
RHOXF2B_5	GGACAGCGACCAGAGCGAGA
RIF1_1	ACATGACACGAATTGCCCTG
RIF1_2	AGTGCTGCTCTACAAGCCCT
RIF1_3	GAAGAACTTGGATTTCGTAG
RIF1_4	GGTGGCCGACATGACGGCCA
RIF1_5	ATGGAACTAAGAGATCAAGC
RING1_1	ACAGGGCCCAGCGTGTGAGG
RING1_2	GAAGAATACGATGACCACCA
RING1_3	GCAGGGCCAAGTACTTGGAG
RING1_4	TAAGATCTATCCTAGCCGGG
RING1_5	CTCCTATTACCCGCTCCGTA
RIOX1_1	AACTTCAGTCAGGACGACCT
RIOX1_2	TCGTCCGTTGATGTAGCGAG

RIPK4_1	AACCGGAGATCCCATGGCAA
RIPK4_2	CAAACAGGCCATCCATGCTG
RIPK4_3	GCTGCATGAGGCGTATCAGG
RIPK4_4	TGCGCGATGGAGGGCGACGG
RIPK4_5	CTCCACGGCCATGTGCAACG
RLF_1	GAGGGAGCAAGAGGTGTCGG
RLF_2	GTTGTGAGTAATTACTGCGA
RLF_3	TCAACAGCCAGTAGAAACGG
RLF_4	TTTGATAAAGCAGTAGCCTG
RLF_5	AACATGGTATCCTCTGTACA
RLIM_1	ACAGAGGTCCCACCTACCAG
RLIM_2	AGTCGGACTAATCCAAACAG
RLIM_3	GAAAGGGCAGAGTCACGGAG
RLIM_4	TTCAACTGAAGCGTTAACAG
RLIM_5	CCCGGCACCATGTGACATTG
RMI1_1	AGGCTGACTTACATCAACCA
RMI1_2	GGGTGGAGAATACAAATCCA
RMI1_3	TCAGAACGATGTTTCACCAC
RMI1_4	TCTAATGGCCAGCAAACCAA
RMI1_5	GATGGAATCGTACAAATACA
RNF112_1	AAAGGAAGTGACTGACAAGG
RNF112_2	GGCGCCAATGGCCTCGCCAG
RNF112_3	GTGGCCACAGTCCAGCGAGA
RNF112_4	TTGATGCGAACCAGCAGCAG
RNF112_5	ACATCCATATGCCCCTGGCG
RNF141_1	AAAGCAGAGGAATCAGACCC
RNF141_2	GATACAACACTCCTCCTCAT
RNF141_3	GATATCACAAGTCAAGCAGC
RNF141_4	TGTACGGGTGGTCTGTACCA
RNF141_5	AGTAGCAAAACATGTTACGT
RNF168_1	ACAGCGTGTGGTTACACGGG
RNF168_2	GAGTCCACGACGATACCCGG
RNF168_3	GCACCACAGGCACATAACCA
RNF168_4	TTAAGGTGGCGGCAGAGCGA
RNF168_5	AAGAAATTCTCTCGTCAACG
RNF17_1	AGTGCACCAGGTGTGGAAGG
RNF17_2	GTCCACATTGAAGTTCACAA
RNF17_3	TAGACAACGCTACTACCCAA
RNF17_4	TCAGGGCTAGATCCCATCTG
RNF17_5	AAGTTAGTTCTCCACAACTA
RNF2_1	AATGGTATACTGCTTCTCAC
RNF2_2	CTGGAAAGTGAACAAACCCA
RNF2_3	GAAGAACACCATGACTACAA
RNF2_4	TTACAGGAGGCAATAACAGA
RNF2_5	AATGGCAATTGATCCAGTAA
RNF20_1	ACTTCGGCAAGACTTTGAGG
RNF20_2	GCATCGCACCATGTCTCAGG
RNF20_3	GTGGAAACAATTAAGCTAGG

RNF20_4	TTATCCCGGAAGCTAAACAG
RNF20_5	GGAGGGCACTACCACTACGC
RNF217_1	GAGCAGAGCACGGTGAGCGG
RNF217_2	GATCACGGACAGGTATGCAC
RNF217_3	GCGAAATTGAGCATGGGCAG
RNF217_4	TGGGCTCCAGCTCTTCGCCG
RNF217_5	AAAACACCAGACGAATTGGC
RNF40_1	AAGGCCGGCTCACTGAGAGG
RNF40_2	GGACTCCTCTCCCAGAGCCG
RNF40_3	GTAGGAGGAGATGGACCTGA
RNF40_4	TCACCTCCAATGAGATGCGG
RNF40_5	AGAGGAGTCCCATCACATGT
RNF8_1	AAAGGACCTGAAGCAACAGC
RNF8_2	GGTTTCGAGAAATCATCAGG
RNF8_3	GTCACAGGAGACCGCGCCGG
RNF8_4	TGAGCCAAGTAAGACCACAG
RNF8_5	AGGGTCTATTCCATTCATCA
RORA_1	ACCATCTCGAGACATCCCTA
RORA_2	AGGCGGCTCGCTCTTGCGGG
RORA_3	CAGGTAGAAGCTGCTGACGG
RORA_4	TGTTCTGAGAGTCAAAGGCA
RORA_5	AGTTGGGGAAGTCTCGCCGT
RORB_1	GACTCCGTAGTGGATCCCAG
RORB_2	GATGGCGTGAGTGATCTGGA
RORB_3	GCAGTCGGCAGTGTTGGCAA
RORB_4	GGAGGCAGAAGCCCTTGCCA
RORB_5	AACCTGAACAACGAGACCAG
RORC_1	ACAGGGAGACATGCCAGCTG
RORC_2	AGAGACAGCACCGAGCCTCA
RORC_3	ATATTCCAACAACTTGGCCA
RORC_4	CCTGCAGAAATGCCTGGCGC
RORC_5	AGGAAGTGACTGGCTACCAG
RPA1_1	ACAGTTGAACCCTCTCGTGG
RPA1_2	GAACATCCGTCCCATTACTA
RPA1_3	GTAAGGAGTGAGGCTGGCAA
RPA1_4	GTACCTGGAGCAACTCCCGA
RPA1_5	ACAACAGAGAAGTTGCCAAG
RPA3_1	GATGAATTGAGCTAGCATGC
RPA3_2	GGTTGGAAGAGTAACCGCCA
RPA3_3	TACGGGTTCCATCAACTCGA
RPA3_4	TGTACACAAGATGGTGGCCT
RPA3_5	TGGACATGATGGACTTGCCC
RPH3A_1	GCAGGCTGGTCCGTCCACCC
RPH3A_2	GTTCTCCACACAGTATGCAG
RPH3A_3	TCAGCAACAGTTCTAACCGT
RPH3A_4	TGTGGAAGCGTTCTGGAGCG
RPH3A_5	ACAGTCCTCACATACTACAC
RPP25_1	AGCGATCGCAACCCGAGCCA

RPP25_2	AGTACGCTGAGACTAGCGGC
RPP25_3	GCCGCAGCCGCTGAAGACGA
RPP25_4	GCGATCGCAACCCGAGCCAG
RPP25_5	CCAGGCGTCTGACCCTGCGT
RPP25L_1	AGGGTATCAGGAGGTAGCTG
RPP25L_2	CATGGCGGCGCACTGTGAGG
RPP25L_3	GCGCTGAGATTGTCAAGCGG
RPP25L_4	GCTGCGCTGAGATTGTCAAG
RPP25L_5	AGTCTGAAGGAAACGTAGCT
RPRD2_1	ACAAACAACCGAATAAGCAG
RPRD2_2	AGACATGGAACTCTCAGATG
RPRD2_3	GCAGTTGTCAACTATGAGGG
RPRD2_4	GTTTGGCAAAGCCAATGCTG
RPRD2_5	AGGAGACTCAGAACCAGTCG
RPS19BP1_1	GAAACTGCGGAACTCGGCCA
RPS19BP1_2	GACCAGGACGAGAAGCACCG
RPS19BP1_3	GAGCTGCTGGCGGCGTCCGA
RPS19BP1_4	TGAGGGCACCGTGTTCACCG
RPS19BP1_5	AAACTGCGGAACTCGGCCAA
RPS6KA3_1	AGGGCAGGGATCATTTGGAA
RPS6KA3_2	GAGCCCGTCCGACAGCGCTG
RPS6KA3_3	GCAACACTTACCTTTCAGTG
RPS6KA3_4	TATAGTGAACAGGACAGCAC
RPS6KA3_5	AGCTGATGTGCATTAGCACT
RPS6KA4_1	CCAGCGCCAGTACTTCAAGG
RPS6KA4_2	CGACGATGAGAGCTGCGCCG
RPS6KA4_3	GAGCAAGGAGTTCCTGACGG
RPS6KA4_4	GCTGCACTACGCTTTCCAGA
RPS6KA4_5	CGCCACGGGCCCGATCCGAG
RPS6KA5_1	ACAGCTCTGAAACTCTGTGA
RPS6KA5_2	GCACCAGATATTGTCAGAGG
RPS6KA5_3	TAGGATGGAAGGAGCAACAA
RPS6KA5_4	TCACTGTCAAGCACGAGCTG
RPS6KA5_5	CATGCAACTTCACAATATTG
RREB1_1	AGTGCAAATCTTCTCACACA
RREB1_2	ATGACGTCAAGTTCGCCCGC
RREB1_3	GTAGGCGATTGTCCTCCAAG
RREB1_4	GTATGGACTGGAGACCCACA
RREB1_5	CAGCACAACACAGACACTGG
RRP8_1	ACTGATGGGAACCAACATCA
RRP8_2	GATCCACCAAAGCAAAGCCC
RRP8_3	GATTACGGGCCCAAGGCCCG
RRP8_4	GCCGAAGTCAGCCACCACTA
RRP8_5	AAGAAGAAATGTCAAAAACA
RSF1_1	AACCCAAAGGCAAAGTTCGA
RSF1_2	CATCAAACTGACACTCACAG
RSF1_3	GAGGAAGAAACACCTAAACA
RSF1_4	GGACAGAAATATCATCACGG

RSF1_5	CCAATTGGTACCAGTACATG
RTEL1_1	CCAACAGGAGTACATGACCA
RTEL1_2	GCAGCTGTGCATCCATCCTG
RTEL1_3	TCAAGAGCGGAAGCAAGCAC
RTEL1_4	TGGCGAGAACACCTCCGAGA
RTEL1_5	CCTGCTCATACTCCACACAC
RTF1_1	GAAGAGTTCCATGATGGCTA
RTF1_2	GCTCAGCTCACCTTCCTCAG
RTF1_3	GCTGCAGGCTGACTCACAGG
RTF1_4	TGTGGGTCGAGCAGCGGCGG
RTF1_5	GCCAAAGTTTACCAACTAGG
RUNX1_1	AACCTGGTTCTTCATGGCTG
RUNX1_2	GATGAGCGAGGCGTTGCCGC
RUNX1_3	TAGATGATCAGACCAAGCCC
RUNX1_4	TGGTAGGTGGCGACTTGCGG
RUNX1_5	CACTTCGACCGACAAACCTG
RUNX1T1_1	AATTGAATTACTGGATCCGG
RUNX1T1_2	GGTGAGGCAGGCCATTGGGC
RUNX1T1_3	GTTTGGCCAGTCTTGCGCAG
RUNX1T1_4	TACCACTAGTCCCAGAACGA
RUNX1T1_5	CAGCTGTTGATTAGCCAGAG
RUNX2_1	AATGAGCGACGTGAGCCCGG
RUNX2_2	CTGGTCATAGGACCACGGCG
RUNX2_3	GTAGGTGGTAGCCCTCGGAG
RUNX2_4	TGCGCCCTAAATCACTGAGG
RUNX2_5	GAATGCGCCCTAAATCACTG
RUNX3_1	GGACGTGCCGGATGGTACGG
RUNX3_2	GGCAAGATGGGCGAGAACAG
RUNX3_3	GGTCGGCGAGTAGGTCGGGA
RUNX3_4	GTGGTAGGTCGCCACTTGGG
RUNX3_5	CACTGCGGCCCACGAAGCGA
RUVBL1_1	ACAGCCACGTGAAAGGGCTG
RUVBL1_2	AGTACCTTGCCAGTTCCAGG
RUVBL1_3	GCTGATGGAGAACTTCCGCA
RUVBL1_4	GTGTGAGACAGAGAATCCCA
RUVBL1_5	ACTACTTACCAATGGCCCTG
RUVBL2_1	AGGTTTCTTACCCATGGCGA
RUVBL2_2	GAAGGCCTGCGTCAGCGCCT
RUVBL2_3	GGTGCCCACTCCCACATCCG
RUVBL2_4	TGATCGACCAGCAACAGGGA
RUVBL2_5	AGATTGATCGACCAGCAACA
RXRA_1	CAAGACCGAGACCTACGTGG
RXRA_2	GAGGACGCCATTGAGGCCCA
RXRA_3	GGTGAGGGAGGAGTTCACCT
RXRA_4	TATGGAGTGTACAGCTGCGA
RXRA_5	AGGAAGCCATGTTTCCTGAG
RXRB_1	ACTGGAAAGGGAGAGCCCAG
RXRB_2	AGGCGGAGAACAACAAACCC

RXRB_3	GGACAACAAAGACTGCACAG
RXRB_4	GTGGCTTCACATCTTCAGGG
RXRB_5	ACGGCTATGTGCAATCTGCG
RXRG_1	ACGCTTGGCCCATTCAACGA
RXRG_2	AGGACATCAAGCCCTTACCA
RXRG_3	GAGGCAGAATGTGCTACCAG
RXRG_4	TGAGGGTGGGCCCATGGCAG
RXRG_5	CTTCAAGAGGACGATAAGGA
RYBP_1	ACCAGCTGAGAATTGATCCG
RYBP_2	GCGAAACAAATCACACCTCA
SAE1_1	AAAGTTGCCAAAGTTAGCCA
SAE1_2	GCCGACAAGAAGCACCCGAG
SAE1_3	TGGCGGCGGCATTAGCGAGG
SAE1_4	TTGAAGGAGAAAGTAGTCGG
SAE1_5	GGGATCTTCTGGAGTTACCT
SAFB_1	ACATCAAAGAGGTCTAGCAA
SAFB_2	CCACGGAAAGATGATCTCCG
SAFB_3	GATAGTAGAGAGCTAGTCGA
SAFB_4	GGGTGTGGAAGATAACGGGC
SAFB_5	AAACGGAATGTGGACTCGAG
SAFB2_1	AACGAACGCCCGCAGCCCGG
SAFB2_2	AAGAAGCGGAACCTGGACAC
SAFB2_3	AATGGAGCGGACGGTCGTGA
SAFB2_4	ATTAGGACTGAAGATGGAGG
SAFB2_5	AAGCAGAGGAATGGAGCGGA
SALL1_1	AAGGTGGTGGCACTGCCCGC
SALL1_2	GAGCACTCACCATCTCGCCG
SALL1_3	TATGCAGCAGCGCTCTCCAA
SALL1_4	TTTGAGCCAGCATGTCGCGG
SALL1_5	ATGGGATGCCAGTACTCGTG
SALL2_1	GAATGTCTCGGCGAAAGCAG
SALL2_2	GAGGAGGCAGCCACTCCAGG
SALL2_3	GGTACTTCTCACGATGCCGG
SALL2_4	GGTGGGATCCGTGGGCACGG
SALL2_5	CAATTATCACCATTACAGGA
SALL3_1	CCACTGGCCATGCTGCCCAG
SALL3_2	CGGCCGCGCCTTCACCACCA
SALL3_3	GCACTACCGGACGCACACGG
SALL3_4	GGGATGGACAAAGCACGCAC
SALL3_5	CCGACACGCCGGACTCCACG
SALL4_1	AATCCCAAGGACCTCACGGG
SALL4_2	AGTGGCGTTATTCACAACGG
SALL4_3	CACCAAAGGCAACTTAAAGG
SALL4_4	GCCACCGAACATCTCCGCGG
SALL4_5	GGTTCTGAGCCAATGACCGT
SAMD4B_1	CAGCCGGAACACCTTCCAGG
SAMD4B_2	GAAACGGGTCACCCGTACCC
SAMD4B_3	TAGCCAACCGCTCTTCCAGG

SAMD4B_4	TTATCACAGAACGTCACCAA
SAMD4B_5	AGCATTGCTCTCGATTGAGT
SAP130_1	CTAAAGTAACCACAGTCCTG
SAP130_2	GAAGCTCCAATGTGAAGAGG
SAP130_3	TCAAGCTTCAGCCATTCCTG
SAP130_4	TGTGGTAAGGCCCTATCCAC
SAP130_5	ACTTGCAAGTCGTTACCTCG
SAP18_1	CCGTAGGAGGAAGATGGCGG
SAP18_2	GAGGTGGTGCCCGATTTGGA
SAP18_3	GGAGTCGCGCGTTACCCAGG
SAP18_4	GGGAGAACTCGTCCATTCGG
SAP18_5	GAAACCGATCGACCGCGAGA
SAP25_1	AGGGGAACACGCTGCCCCCG
SAP25_2	CATACAGCGGCCAGAATGAG
SAP25_3	CCCAAGTATGAAGCCAAAGC
SAP25_4	TTTCCTCCTAGGGTTCCCGG
SAP25_5	GCTCTCTCCAGATCCAGGAA
SAP30_1	ACCGCTCACCATCCTCCCGC
SAP30_2	ACGCCTGACGAGATGAGCCG
SAP30_3	GCAGGCAGCACAGTTGCCCG
SAP30_4	TGACGAGATGAGCCGCGGCG
SAP30_5	GGCCGCGGCAGCGACCACTG
SAP30BP_1	ACTCACCCACGAGTTCCTGG
SAP30BP_2	GCTGTGGGAATAAGATGGCG
SAP30BP_3	GCTTGGCACCAACTACCCAA
SAP30BP_4	TGATTTGAACATCTGCCAGG
SAP30BP_5	ATATGAACTACATTATCCAA
SAP30L_1	AAGAGGAAGACAAGTGACGA
SAP30L_2	GATGAACGGCTTCAGCACGG
SAP30L_3	GCTGAAGGAGGCGTTGCCCG
SAP30L_4	TAGACTGGACCAGAAATCGG
SAP30L_5	AGCTGCTGCCTCATCGAGGA
SATB1_1	ACGAGCAGGAATCTCCCAGG
SATB1_2	AGAAATTCTGCATAGCCCGA
SATB1_3	ATTTGAACGAGGCAACTCAG
SATB1_4	GAATCAGAGTTCATTGGCCA
SATB1_5	ATGCTAAGTACCTGTGAAAG
SATB2_1	AAAGTTGGAAGACTTGCCTG
SATB2_2	GGAGACTGCGCTCCTGGCCC
SATB2_3	GTCGGAGCAGCATGGAGCGG
SATB2_4	GTGCATCTGTCACATAACTG
SATB2_5	AACAGACCTCCCTATTAAGG
SBNO1_1	ACAATTGTAGATCCCAAGGG
SBNO1_2	ATTGGTGAAAGCCAGTCTGG
SBNO1_3	GCAAGCAGTAAATCTTGCCC
SBNO1_4	TTATTCAAGCAGTAGAACGG
SBNO1_5	GCAGACCAATTACAACACAC
SBNO2_1	GAAGCAGCACCCAGACCGCG

SBNO2_2	GGGCGCACTGACCTTATCGT
SBNO2_3	TACAGGAGGCTGCCCGCCGG
SBNO2_4	TGGTGGCAAGCTGGAGGCGG
SBNO2_5	CAACGACCTCAAGTACGATG
SCAF4_1	AAGTTTGCAGTACTACCCTC
SCAF4_2	AGGAATCACAGGCTGAGCCA
SCAF4_3	GAGCAGATGATTCCTCCCAG
SCAF4_4	GATCTGGTTCTAGATCTCGA
SCAF4_5	CTTTCTTGAGATCGAGATCG
SCAND1_1	GCGGCCTGACATCCGCACCA
SCAND1_2	GCTCCTTCCAGTTTCTCCGG
SCAND1_3	GCTGCTCGCCATCCTGCCCG
SCAND1_4	TGATGCGCACATCCGTGCGG
SCAND1_5	CACCGCCGCGGGACTCCCAG
SCMH1_1	GAAGCGGCACCAGTAGTCAA
SCMH1_2	GGTGGGTTCAGCAAAGTCCG
SCMH1_3	TGGAACATCAACCAGGGCAG
SCMH1_4	TGTCGCTCCCATCAAGGCGC
SCMH1_5	AACTACTGTGGCAATACAGG
SCML1_1	AATGACGGTTACATAGAGGA
SCML1_2	GTTTCCATGCGAGATCCCTG
SCML1_3	TGGGCCAGCATACCTGAAGG
SCML1_4	TGTGGATGCTGTCAGCCCGA
SCML1_5	CAGCCGTATTATGCATCTGA
SCML2_1	ACAGAAGATGTACTTGACTG
SCML2_2	ATACTGAAGTGGCATTGCGA
SCML2_3	ATGGGACAAACAGTGAATGA
SCML2_4	CTTTCAAATACTCCTCCCAG
SCML2_5	AAATCGCTGACCAGAGACCG
SCML4_1	ACAGCTTGGCGAGGAAGCGG
SCML4_2	AGAGTACCTGGTGAACCCTG
SCML4_3	GTCCTCCACAGTCCAGGCGG
SCML4_4	TGCAGTCTCAAAGGATCCCG
SCML4_5	CCCCACAGGCTCTCACAGGT
SCRT1_1	CGTCAGCGAGGGCTACGCGG
SCRT1_2	GATGTAGCCGCCCGCAGCGG
SCRT1_3	GTTGATGTAGCCGCCCGCAG
SCRT1_4	TATCGTGCAGTGGCGCGCCG
SCRT1_5	ACGTACACCTTGCCGCACGT
SCRT2_1	AGTGACCGACAGCTACTCCA
SCRT2_2	GGACGGCTTCCAGTGCAGCG
SCRT2_3	GTCGGCGCGCTACTTCCGAG
SCRT2_4	TACGTGCTGCCTGGCGCCCG
SCRT2_5	ACGTAGGCCTTGCCGCACGT
SEBOX_1	GGAAGAGAACAGGAGCTCTG
SEBOX_2	GGAGCTATGTCGACACACTG
SEBOX_3	GGGCCAAAATAATCAAGAAC
SEBOX_4	GGTGGTCCGCTTTCTCCGGT

SEBOX_5	CCTGTACCTTGGCCTCAGGA
SENP1_1	ATTCCCAGACTCCAACTCCC
SENP1_2	GATGGAGCGAAGTAAAGAGA
SENP1_3	TGTAAAGATGAGCTTGACGA
SENP1_4	TTACAGACAGCTGCTACAGA
SENP1_5	AAGATAGGGAATATACACTG
SENP3_1	CCATCTTTCACCCCAGCAAG
SENP3_2	CCTCCACCTGACTTGAGTCG
SENP3_3	CGTTGGGCAGAGTACATGAA
SENP3_4	GGGCTCCTTACTCTGTACGC
SENP3_5	AGCTGATCCAGTCTTACCAG
SENP5_1	CAGTCAGAAAGCCTCTCCAG
SENP5_2	GGAAAGGCTGCTCTAAGGCG
SENP5_3	TCACAGTAATGAGAGACCAG
SENP5_4	TTGGCAGACTGCTGTTACGA
SENP5_5	AGTGCTTTAGGTCAGGCCAA
SENP6_1	CAAGAGCGGCGGTAGCGCAG
SENP6_2	CCAACTGCAAATGAATAAGG
SENP6_3	GAAGAACATGCTGAATCAGC
SENP6_4	TTTGAAGCAGGAGATAGAGG
SENP6_5	AGGCATCTCTGTTACCAATG
SENP7_1	AATTCAGAAGCTCAGAACGC
SENP7_2	ACAAGAGAAAGCTCGGGCGA
SENP7_3	AGGCTGTCAACAGATGTCGA
SENP7_4	TGGAAGTAACAGGACACCCT
SENP7_5	AGGACCCCACCTGTAACTGA
SENP8_1	AGGCAGCTGGAGGAACCCAC
SENP8_2	GCAGTCATGAAACTGACTGT
SENP8_3	GGTAGACCAATAAACTCCAG
SENP8_4	GTTACATGGACAGTCTACTG
SENP8_5	CAACTCAGTTCACGCAAAGC
SERP2_1	AAGCCATGGTGGCCAAACAG
SERP2_2	GATCATTCAGAGCATAAGGA
SERP2_3	GCAAGAGGAGAAATATCCTG
SERP2_4	GGCCAAACAGCGGATCCGGA
SERP2_5	ACAGCAAAAACATCACCCAG
SERPINE1_1	ATTTGTGGAAGAGGCGGCGG
SERPINE1_2	CTGGAGCATGGCCAACACCG
SERPINE1_3	GCTCCTTGTACAGATGCCGG
SERPINE1_4	GCTGAGTTCACCACGCCCGA
SERPINE1_5	AGGGTGAGAAAACCACGTTG
SERTAD1_1	AGGCACTGGAGCGACCGCCG
SERTAD1_2	GGAGCTAGAGGCCACGGCCG
SERTAD1_3	TAAGTTGTCAGCCACACTGG
SERTAD1_4	TGTGGCTGAGGTCCTCCAGG
SERTAD1_5	GCCACCAGGCCGTAGCATCG
SERTAD2_1	AGAGCTGGACCACATCATGG
SERTAD2_2	CAAGTCTGTCAGGAAACCCG

SERTAD2_3	GAGGTCGTCGGCAGACACAG
SERTAD2_4	TGTGAAAGGGACCTCCAGCG
SERTAD2_5	AAGGTGTAAGACACCTTGGA
SET_1	CAGCACCATGTCGGCGCCGG
SET_2	GAGCAGCACCATGTCGGCGC
SET_3	GAGCTCAACTCCAACCACGA
SET_4	GCTCAACTCCAACCACGACG
SET_5	ACATTTGTCAACCATCCACA
SETBP1_1	AAAGAGGAAACACAAACCGC
SETBP1_2	AAGACAGTCCATTAAAGCGG
SETBP1_3	CAAGGAGGCAAACTTCACAG
SETBP1_4	GCAGTGCATAGTAAGAACGA
SETBP1_5	ACCCGTTGCTCTATCTTCGT
SETD1A_1	ACGGCACATATCCTTCAGGA
SETD1A_2	GCAAAGATGGATCAGGAAGG
SETD1A_3	GCCAGGAGTGCCCACCGCAG
SETD1A_4	TGGACCTGACATGGCAACGG
SETD1A_5	AGAGCCATCGGAAATTTCCG
SETD1B_1	CATGGGCAACATTATCCACG
SETD1B_2	GATGTCCAGCAACCGCCCGG
SETD1B_3	TGGGCAGCATTTCAGCCTGG
SETD1B_4	TGTGGTTGAGCCTTGAACGG
SETD1B_5	CCAGCAGGCACGAGGCGATG
SETD2_1	GCGGAGCTGATACTTACTCA
SETD2_2	GTAGATCAGAAAGAGAGCGA
SETD2_3	GTGAACGGACAACTGAGGGT
SETD2_4	TGCGGATCAGCCAATTGCCG
SETD2_5	AATGAACTGGGATTCCGACG
SETD3_1	GAGGAGTGTCATATTCACTG
SETD3_2	GATATGTGTAACCACACCAA
SETD3_3	GCAGAGCCACACACTCACAG
SETD3_4	GTGGATCACAAACTCTGCGT
SETD3_5	GATCACTACTGGTTACAACC
SETD4_1	AAGGTGCACAGCGCCAGCAG
SETD4_2	ACAGCCGTTGATTATCGTGA
SETD4_3	GATGAGTCAAACATCCCTGC
SETD4_4	GATGGGATGGACCATCTTGG
SETD4_5	ACAGGGCAGGTATACGCCTT
SETD5_1	AGGTTATTAGACTTCATCGG
SETD5_2	GGCCATTCAGGTCAGAACGA
SETD5_3	TATGGGACCACTCAGAGGCA
SETD5_4	TCTGATGTGGTGACTCCCAG
SETD5_5	GTGCCCTTTACGTATCACAA
SETD6_1	GGAGGCCCTACTTTGCGCTC
SETD6_2	GGTGTGCTGCGACAGGAGCG
SETD6_3	GTTGGCCAAATCCTTCTCCA
SETD6_4	TGCAGCAGGCACCATCACGG
SETD6_5	CGCCAATCTAGAATACTCTG

SETD7_1	GAAGAGCTCACCGTTGCCTA
SETD7_2	GATGATGCCTTGCAGGGCCA
SETD7_3	GATGGGAGACTGATCTTCAA
SETD7_4	TTCAGGCCCACTCTTCCCGG
SETD7_5	ACGGAGAAAAGAACGGACGG
SETD8_1	CATCACGAAGTCAAATGCCA
SETD8_2	GCCCGGAGATGGTGGAGCGG
SETD8_3	GTGGAATCACAAGATGAGGG
SETD8_4	TGGGCAAATACCTCTAGCCA
SETD8_5	GCACGTGACTACCTGCAGCT
SETDB1_1	AAGGAAAGAGTCTACTGTCG
SETDB1_2	GAAGGACTTCTACTCCAAGC
SETDB1_3	GCAAGAAGAGAACTAAGACT
SETDB1_4	GCATCCAAACCAATGCACCC
SETDB1_5	AGATGTGAGTGGATCTATCG
SETDB2_1	AAGGAGTCTACGAAACGTGG
SETDB2_2	ATAGAGAAGAAACTCCACCA
SETDB2_3	TGTCTAGATGACATTGACAG
SETDB2_4	TTTCGTAGACTCCTTCCACA
SETDB2_5	AAAGTTCAGTAATAATCCCA
SETMAR_1	ACCGGCGACGATCAGCTCAG
SETMAR_2	ACTGAACAGACTCACCTCAA
SETMAR_3	GACGACACGGCCTTGTGGGA
SETMAR_4	GGAGTTTAAGGAGAAGCCTG
SETMAR_5	AGTGGTCACTGCATCGGCAC
SETX_1	AATGTTGGAGATACCTCCCG
SETX_2	ATGGTGATTCTGGATCGCCT
SETX_3	GCTGTTTGAAATTCACCGGA
SETX_4	TTTGTCTGAGCCCAACAACA
SETX_5	AGAACTCCGTGTAAACGCAG
SF3B1_1	AACAGCTTCAGGCTTGGCGG
SF3B1_2	AAGATCGCCAAGACTCACGA
SF3B1_3	ACTCCTCGAACAGATCGAGG
SF3B1_4	TAAGGAAGGAGTATGCCCAG
SF3B1_5	AAAAGGATCAAGACGCTCTG
SF3B3_1	AATGTGTCTCCTTCTTCCAG
SF3B3_2	ACTGGCCAGGAACGATGCGA
SF3B3_3	GGACCACATGATTTAAACCA
SF3B3_4	GGAGGGTCAGATGGTCCAAG
SF3B3_5	AAGAATAGCTCAACCATGAG
SFMBT1_1	ATACTCACGCCCTCTAGCAG
SFMBT1_2	ATTCTGCTCACACCACCCAA
SFMBT1_3	GATCCATTTCTTCATCACGT
SFMBT1_4	GGGTCCACAGCAGTTCCCTA
SFMBT1_5	ACTGTCGGCGTGGCACACAA
SFMBT2_1	AAGGTGTTGATGCCGCCGGA
SFMBT2_2	ATAGGGCCTTTCCCTCGCAG
SFMBT2_3	CAATACATACACCTTCCAGG

SFMBT2_4	GTCAGCCTCACTCCTCCCAA
SFMBT2_5	ACACTGAATCCTATGACCAG
SFPQ_1	AAGCCGCCGCATCGAGGCGG
SFPQ_2	AGAGGAAAGTTACAGCCGAA
SFPQ_3	AGTACGAATATTCTCAGCGA
SFPQ_4	GCGGGAAAGAGACATGCGAA
SFPQ_5	ATGATCGTGGAAGATCTACA
SGF29_1	GAAAGCTCTGGACAAGATCG
SHOX_1	AAAAGCAAGGACGGTAACGG
SHOX_2	CAGCCGCACCAACTTCACGC
SHOX_3	CCTCGCGCATGAAGGCGTCG
SHOX_4	GGTCCTTGAACAAATGCACC
SHOX_5	GGACCACGTAGACAATGACA
SHOX2_1	AAGGAAGCCAACGAAAGCTG
SHOX2_2	AAGTCGGACCAATTTCACCC
SHOX2_3	ACAAGCTTCAAACTGGCTGG
SHOX2_4	GGAGGCGATCACGTACCGGG
SHOX2_5	GCAGCTGGGTACCTGCACTC
SHPRH_1	AGAAACGCAGTCCATCCAAG
SHPRH_2	AGATGCTCTCACTCTTCCCG
SHPRH_3	GATGACGAGCAGCCCTGCCC
SHPRH_4	GTGAAACAGATCAAAGGCCA
SHPRH_5	AGTCGGCCATCACGCAAGGT
SIAH2_1	ACAGGGAAACAGGACTGCCG
SIAH2_2	GAGAAACAAGAGAAGTACGA
SIAH2_3	GCTGCAGGGTTTATTAGCGC
SIAH2_4	TACAGACATTAACTTGCCAG
SIAH2_5	CTTGTGGGCGTGCATGAGAT
SIK1_1	ATGGTCGTGACAGTACTCCA
SIK1_2	GAACTCCGACATGATAACCA
SIK1_3	GGACGTACAGCACCACGCCC
SIK1_4	GTGCTGCCGGATCTGGGCGA
SIK1_5	CCCCTCAAAGACTTCCGGGG
SIM1_1	AGACCACGACGAGATGACGG
SIM1_2	CAGCTGCGAGGTGATAGCCG
SIM1_3	CGTAACGCCGGCCTCACCTG
SIM1_4	GATGAGCTTCATGTCCAGGC
SIM1_5	AGGTTCCTGGCGAAACACGG
SIM2_1	GAAGTGATGGCCGACGGCAG
SIM2_2	GCTGGGATCGCACTTGCTGC
SIM2_3	GTACAGCTTGATCTCGGTGA
SIM2_4	TGGATGGAATACCTTGGAGC
SIM2_5	CAAATGGACAAACTGGAATG
SIN3A_1	ACTGCCGGAGCTCTGTGGCA
SIN3A_2	GGTGTGTGAGGCTGGACCGG
SIN3A_3	TGAGACCATGCAGTCAGCTA
SIN3A_4	TGAGGTGCAAACCAATGACA
SIN3A_5	ATCCCATTATCAGATCGGGG

SIN3B_1	GAACGAGCACGACAAGACCC
SIN3B_2	GAAGGAGCAGCTGAACACGA
SIN3B_3	GACATGGCGCACGCTGGCGG
SIN3B_4	GGAGAATTCGCACAACCACG
SIN3B_5	ATGTCTATTCTATATCCGAG
SIRT1_1	AGAGCGTGAGGTGCCGGCGG
SIRT1_2	AGTTGGATGATATGACACTG
SIRT1_3	GATCTGGGAAGTCTACAGCA
SIRT1_4	GTGACTGGACTCCAAGGCCA
SIRT1_5	ATAGCCTTGTCAGATAAGGA
SIRT2_1	GGACGAGCTGACCTTGGAAG
SIRT2_2	GGAGAAGGGTTACTTACATG
SIRT2_3	TAGGTTGTCATAGAGGCCGG
SIRT2_4	TCAGATTCAGACTCTGAGGG
SIRT2_5	AGGAGAAGAAACGCGCTGGG
SIRT3_1	GATGAGCAGCAGATCTGCCA
SIRT3_2	GTTGAACGGGTCGAGGCCGG
SIRT3_3	TCGGGCCAGAGCCTGCCAGA
SIRT3_4	TTGGCCAAGGAGCTGTACCC
SIRT3_5	GCAGAGGCCTCCCAGACCCG
SIRT4_1	AATCGGGAGCTTCTTCTCCC
SIRT4_2	ATGGGCCTCAGCACTCCAGG
SIRT4_3	TGCTGGCACAAATAACCCAA
SIRT4_4	TTTATGCCCGCACTGACCGC
SIRT4_5	CAGGAATCTCCACCGAATCG
SIRT5_1	CCACTAGACATAAATCACAG
SIRT5_2	GGGAGTTCTACCACTACCGG
SIRT5_3	GTAGGTGGGCACTTCCTCTG
SIRT5_4	TCAGGAAGAGTCGTTCCACA
SIRT5_5	ACCGGGGAAGTTTCTCAACT
SIRT6_1	CTTCCACAAACATGTTCCCG
SIRT6_2	GATGTCGGTGAATTACGCGG
SIRT6_3	GGGCCGCGCGCTCTCAAAGG
SIRT6_4	TTCCGCTCCAGCTCCTCCGG
SIRT6_5	GGACCTGGCACTCGCCGATG
SIRT7_1	CAAGTATTTGGCGTTCCGGA
SIRT7_2	CCGCTCCGAGCGCAAAGCGG
SIRT7_3	GCGCATCCTGAGGAAGGCGG
SIRT7_4	GCGTCTATCCCAGACTACCG
SIRT7_5	CAGACGGGTGATGCTCATGT
SIX1_1	GAGGCAAAGAGACCGGGCCG
SIX1_2	GCAAAGAGACCGGGCCGCGG
SIX1_3	GCAGAACCAACTCTCTCCTC
SIX1_4	GCTGCGAGGCTGTTAAGCCC
SIX1_5	CAAGAACGAGAGCGTACTCA
SIX2_1	AGGATGAGAAGACTCCATCG
SIX2_2	CAAGGCACACTACATCGAGG
SIX2_3	GTGCAGGGACGGCAGCCCAG

SIX2_4	TGAAAGCGTGCTCAAGGCCA
SIX2_5	GCCAGCTCACGCTTCTCGCG
SIX3_1	AACATGGACAACTCTTCCGG
SIX3_2	ACATGGACAACTCTTCCGGG
SIX3_3	GGCAGAGTCGCCGTCCACGG
SIX3_4	TAGCGGCGGCGGGAACGGTG
SIX3_5	AAGTTCACCAAGGAGTCTCA
SIX4_1	GAAGCTGCCACTGACACAGG
SIX4_2	GGCGCACCGAGAAGTGGCGG
SIX4_3	GTACAAGGCGCGCTACACCG
SIX4_4	TAGCCAACCAATGTCTGCAC
SIX4_5	CGAGAGCCACAGCTTCGAGT
SIX5_1	AAACATGGCTACCTTGCCTG
SIX5_2	CCAGCAGTGCTCCTCAACGG
SIX5_3	GCAGGGCTGGAACTAAGCGC
SIX5_4	TGTCCACTGCGCCAAGCGCG
SIX5_5	CCTGCGCGCGCGCTACCATG
SIX6_1	GCACTACGGGCGGAGGGCGA
SIX6_2	GCCATCGTGGCCTTTCACGG
SIX6_3	GCTTGAAGCACACTACCAGG
SIX6_4	TTCCGGGCGGGCACTACGGG
SIX6_5	GCTACGCGCACGAGCCATCG
SKA1_1	AAGTAACAGTAACCCAGAGC
SKA1_2	AGGCTATCAGAGGTCCGAGG
SKA1_3	GAAGAAACGAAGGATACCAA
SKA1_4	GAGGCTATCAGAGGTCCGAG
SKA1_5	AATTTGATTATAGGTTAAGC
SKA3_1	CAGCACGCTGGACTGCGAGA
SKA3_2	GAGTCTGAACTTCTGAATGA
SKA3_3	GCGGTCTCTGGCCAGCACGC
SKA3_4	TATGAATCACACCAGTGAGG
SKA3_5	AGTTCTACCAAACCCTCCAC
SKI_1	AAGCAGAGAGGCGCTGGCGA
SKI_2	ATGGCAACAAGTACAAGCGG
SKI_3	GGAGGACAAGGACTCGGAGG
SKI_4	TTGGGTCTTATGGAGGCCGG
SKI_5	AGTGCTTCGGCAAGTGTAAG
SKIL_1	AATGTAATCAGCCCACAGGA
SKIL_2	GAGATGAAGCGGCAGCAACA
SKIL_3	GCAGAGATTGGACCATGCTG
SKIL_4	TAAAGAGCTCACAAAGACAG
SKIL_5	AAATGGAAAAACGATAAACA
SKIV2L_1	AGGCCGGCGAAGAGACAAGG
SKIV2L_2	ATCCGAGCTCCACGGCCCGA
SKIV2L_3	GAGTGCTCCACACCATGCAG
SKIV2L_4	GTAACCCAGTATCTGGCCTG
SKIV2L_5	CACACGTGCCCAGTTGCCCG
SKIV2L2_1	ATAACAAGACAAGATGCCGT

SKIV2L2_2	GGAATTGGTATTCACCATGG
SKIV2L2_3	GGGCGACTCGACCACTGCGG
SKIV2L2_4	TTGGCCAAAGGAGACCAGAA
SKIV2L2_5	TCTGTGTAAATAACATGACA
SKOR1_1	AGGAGCAGTTGCCAGTGCAG
SKOR1_2	GAGAGGAGCCCGCCAAGCGG
SKOR1_3	TCAGAGCCAAGCCAAGGCCG
SKOR1_4	TGAGCACGTGAAGAGCGCGG
SKOR1_5	AGAGGTGATGCCCCTCAAAG
SKOR2_1	ACAGACTCTGATGCTTCTGG
SKOR2_2	ATGGCACAAGCTGAAGGCGG
SKOR2_3	CAACCTCAAACCCAACCAGG
SKOR2_4	GGACGAGCTGGTCTTCGCCT
SKOR2_5	CGCAGGATCTCCAGTTGCAC
SKP1_1	AAGGTCTTGCGAATCTCCTC
SKP1_2	AGGTACGCAAAGAGAACCAG
SKP1_3	TTGTTCTCATCATCTTCAGG
SKP1_4	ACTATTAAGACCATGTTGGA
SKP1_5	CATCATCTTCAGGAGGAGGA
SLBP_1	AGGCATCAGAGCCGCTGCGA
SLBP_2	GAGACACATGGACAGTCAAG
SLBP_3	GAGGCCCGAAGACGCCGAGG
SLBP_4	GCAAATCAAACTCTGGAAGG
SLBP_5	CAGATGAAAGTGTCCTAATG
SLC4A10_1	AATGGAGCCGCTGCTGCCTA
SLC4A10_2	ACAGTCAGAACCAAATTCCA
SLC4A10_3	ATTGGAGTACATGTGCCCTT
SLC4A10_4	GAAGCAGTTGTGGATAGAGG
SLC4A10_5	ACAGTTTATTCTTGGAACCG
SLK_1	AATACTGCTAGTATCAGCAG
SLK_2	ATTATAGGAGAACTGGGCGA
SLK_3	CATCCGAGAATTGATTGCAG
SLK_4	CATTAGCACAGCCATCCAGA
SLK_5	AGGATACGCAGAGTAATGAT
SLTM_1	CAAGAGGCACATGAGCAAGA
SLTM_2	GACGAGCAATGGAACTTCGA
SLTM_3	TAAGAAGGAAGACTGCGTGA
SLTM_4	TGAAGTCCGAGCTGAAGCGG
SLTM_5	AAACCTCTTTGCTTGACCAG
SMAD1_1	CATGAGGCAGTCCCTTCCGG
SMAD1_2	CTGCCCTCAGAAATCAACAG
SMAD1_3	TATGAGCTCAACAATCGTGT
SMAD1_4	TGAGCTAAGAGGCTGTGCTG
SMAD1_5	AGTTACGGAACTGAGCTAAG
SMAD2_1	GAGTGAGTATAGTCATCCAG
SMAD2_2	GATGGAAGAAGTCAGCTGGT
SMAD2_3	TATATTGCCGATTATGGCGC
SMAD2_4	TCACTGATATATCCAGGAGG

SMAD2_5	ACAGCTTCTCTGAACAAACC
SMAD3_1	ATGAACCACAGCATGGACGC
SMAD3_2	GAAGGGCGAGCAGAACGGGC
SMAD3_3	GAATGGCTGTAGTCGTCCAG
SMAD3_4	GGCGAACTCACACAGCTCCA
SMAD3_5	AGAAGCGCTCCGAATTGGAG
SMAD4_1	GATCAGGCCACCTCCAGAGA
SMAD4_2	GCTTACAGTAATGTCCGGGA
SMAD4_3	GGTAGCATTAGACTCAGATG
SMAD4_4	TCTGCAACAGTCCTTCACTA
SMAD4_5	GCAATGGAACACCAATACTC
SMAD5_1	GATATACTGGGCATAATCTG
SMAD5_2	GGATTGAATTCATTATGACG
SMAD5_3	GTGGAAAGAATCTGGAAACG
SMAD5_4	TCATCAGGTGGCATATAGGC
SMAD5_5	ACACGACAATATATAACATG
SMAD6_1	AGGAGGGCACACTTACCCAG
SMAD6_2	ATTGTCTTACACTGAAACGG
SMAD6_3	GGTCGGGCCAGCGAAAGAGG
SMAD6_4	TACGACCTACCTCAGGGCAG
SMAD6_5	CGCGACGAGTACAAGCCACT
SMAD7_1	AAGGCGGTGCGAGGTGCCAA
SMAD7_2	AGAAGGCGTACAGCCTGCAG
SMAD7_3	TAGGACGAGGGCGGCTGCGC
SMAD7_4	TCCTTACTCCAGATACCCGA
SMAD7_5	CGAATTATCTGGCCCCTGGG
SMAD9_1	CACCTGGAGTCTCCACCCGG
SMAD9_2	CATACACGACTTCAAAGCCG
SMAD9_3	GAGTGGCTCACTGTGCAGGG
SMAD9_4	GCGACCGAGCACCAGTGCTG
SMAD9_5	ATGTGATTTACTGTCGCGTG
SMARCA1_1	AAACAAATGACACGGAGAGA
SMARCA1_2	GATAGTGGCGGTCGCATCCG
SMARCA1_3	GCTGACAATATGCTCATCAG
SMARCA1_4	GTAAGGCCCACAGTTCATGC
SMARCA1_5	ATAGCTTGTAGATCAACCTG
SMARCA2_1	CTAGGCCCAAGAATTGGCCC
SMARCA2_2	GATGCCACCAAGCCAGCCGG
SMARCA2_3	GCTCTTGCAGAATTTCCACG
SMARCA2_4	GGTATGCGACCACCTCACCC
SMARCA2_5	ACACCTAGGCTATTCAAATG
SMARCA4_1	ATGGTCCCTCTCGCAGCCCA
SMARCA4_2	GAGGCTGGCCTGGCATCCCG
SMARCA4_3	GCATGCTCAGAGCCACCCAG
SMARCA4_4	GGCCGAGGAGTTCCGCCCAG
SMARCA4_5	CTAGGTATGAAGTAGCTCCG
SMARCA5_1	AGAACAACTTGCATGAGCTG
SMARCA5_2	GCAGCCTCGATCGCCAGCGG

SMARCA5_3	GGATGAGTGAATTCAAGAGA
SMARCA5_4	TGATACAAACAACTGCCTTG
SMARCA5_5	ATGCATCTAGTAACCAACAG
SMARCAD1_1	AAAGTGTTTGCAGAAGACCA
SMARCAD1_2	GTCACTCAAGCCCTATCAGA
SMARCAD1_3	TCTGAAGATGAAGAGTCCCA
SMARCAD1_4	TGCATCACCAAACATCAGCA
SMARCAD1_5	ATGACCGTAGTCTGTTTCGA
SMARCAL1_1	AAAGGAGTGAACTCTCAGAA
SMARCAL1_2	GAGCTGAGAAGCAAACGCCA
SMARCAL1_3	GCGATGCATGCTCATCTCCA
SMARCAL1_4	GGAGTGGCCGCTCCTGGTGG
SMARCAL1_5	CCCAGATTGCATCAACGTCG
SMARCB1_1	ATGGCACGGCATCTAAGTGG
SMARCB1_2	GAAGCCCGTGAAGTTCCAGC
SMARCB1_3	GAAGTGGAAGAGATTCTGGA
SMARCB1_4	GAGAACCTCGGAACATACGG
SMARCB1_5	GCAGATCGAGTCCTACCCCA
SMARCC1_1	GTTGGTGACATGCTTCCCAA
SMARCC1_2	TATTCTTCCTCACAAGACGA
SMARCC1_3	TGAGCAAGAAGATCTAACCA
SMARCC1_4	TGCGACGATGGCCGCAGCGG
SMARCC1_5	ATCTCGAATGGATCGTAATG
SMARCC2_1	ATGTTGTGTATCCTGTCCCG
SMARCC2_2	ATTGTAGCAACTGTACAACC
SMARCC2_3	CTGCACCAAGGACTTCTCAA
SMARCC2_4	GCGGTGCGGAAGAAGGACGG
SMARCC2_5	ATACCTATTGTAGTCAAACC
SMARCD1_1	AGGCAGCCGAATGACACCTC
SMARCD1_2	CAGTCTGTGGCTCCAAGCGG
SMARCD1_3	GAAACGGCTAGATATCCAAG
SMARCD1_4	GGTAGAAGGACGGCTCCTGG
SMARCD1_5	CCTGGTAATCCAGCATCAGT
SMARCD2_1	AAGGCGGAAGGCGATAGTGC
SMARCD2_2	CAAGCGGATGGAGATCCAGG
SMARCD2_3	GCTCACCTGGTACTGCGCCG
SMARCD2_4	TCGGAATGGATCCATCATGG
SMARCD2_5	ACCAGACCATTGCTCGCAAG
SMARCD3_1	AAATGTACAGTGCCAAGAGG
SMARCD3_2	GAAGCGGGTGGACATCCAGG
SMARCD3_3	GCCGCGGACGAAGTTGCCGG
SMARCD3_4	GGGCTCCAGACGGCATCCCG
SMARCD3_5	CGGGGATCCAGTTTGAACTG
SMARCE1_1	AGACGACGAGAACATTCCGA
SMARCE1_2	GGAGAGCAACAGTGCAACAG
SMARCE1_3	TATGTAAGCAAGGTACGCGG
SMARCE1_4	TCGACAGAGACAATCTCGCA
SMARCE1_5	ACCAACAGCCGGGTCACGGT

SMC1A_1	AAAGAACAAGGAGATCGAGA
SMC1A_2	AGATTATCGGACCATTTCAG
SMC1A_3	GCGAAAGAAGGAAATGGTGA
SMC1A_4	TTGGCAGCTGGCTTGCCCAC
SMC1A_5	ATTGCCACAAGCATACTGCA
SMC1B_1	ACAGCAAGAGGAAACCCTAG
SMC1B_2	ACATTTGCAAGGATTATCCG
SMC1B_3	CATCAAGGTAATCTAGAGCG
SMC1B_4	GCAAGGCATATTGCACTCAG
SMC1B_5	TTACCTGGTAAAAAGCAACA
SMC2_1	ACAGAAGAGATCCTGTACTC
SMC2_2	GATGGATAGTGGCTACAGGA
SMC2_3	TACCAAAGTACGCTCAGCTG
SMC2_4	TTTAGAAGATGCTCTTGCAG
SMC2_5	ACTAGAACTGTAACTCTCGG
SMC3_1	AACAGATCGAGACCCAGCAA
SMC3_2	ACAGTCCATAGTGAAAGCAC
SMC3_3	GACAAGAGCAGATTAAGCAG
SMC3_4	TAAGTTGGAGCTTAAAGCCA
SMC3_5	ACAAAAGCAGAAATAACACG
SMC4_1	ATTATCGGGCCAAATGGCAG
SMC4_2	GCGGCCCTTCCTCTCTGCGC
SMC4_3	TATGGTCCAAGTCAATTCCA
SMC4_4	TTGGTCATTGCTGGAGGCGG
SMC4_5	AACTGAACAAGAATTAAAGG
SMC6_1	AAGGGCCTATAATGAAGCTG
SMC6_2	ACATGAAATGGCTTGGGCAG
SMC6_3	GTTTCAGCAAGCCATAGAAA
SMC6_4	TAGGCTCCGTGGTTTCCACG
SMC6_5	AAAAGATATTAAACACAATG
SMEK1_1	ACGAGAGTTTGTCATGCAGG
SMEK1_2	GAAGGTGTACACGCTCAACG
SMEK1_3	TAAGAACTAGCACTCTCCGG
SMEK1_4	TCAAGGAAAGGACCCTTCCG
SMEK1_5	ATCACAACCACGAAAACACA
SMEK2_1	AGTTCCGATAACCTTACCGT
SMEK2_2	GATAGGTGAGGAGAGCACTG
SMEK2_3	GATCCATTAGAAGTTGCTGG
SMEK2_4	TTATCCGCCTCATAAAGCGA
SMEK2_5	AAAAATACATCAGACTTACA
SMN1_1	ATTAGCTACTTCACAGATTG
SMN1_2	TAAGAGAGAAACCTGTGTTG
SMN1_3	TCTGCCATTTGGTCAGAAGA
SMN1_4	TGTGCCGCGCCGGAACAGCA
SMN1_5	ACCTGTGTTGTGGTTTACAC
SMN2_1	ACAGATTGGGGAAAGTAGAT
SMN2_2	GATTCCGTGCTGTTCCGGCG
SMN2_3	TATAGAACAAAATGCTCAAG

SMN2_4	TCCAGTGTAAACCACAACAC
SMNDC1_1	AAAGTTGGAGTAGGAACCTG
SMNDC1_2	ACAGGCTTGAGGTTCAACAG
SMNDC1_3	AGAGGATTTAGCAAAGCAGC
SMNDC1_4	CAGAAGGTTGAGTTGACAGA
SMNDC1_5	ACCAGCAAAGGTGATTGCAG
SMYD1_1	AGAAGGCACCGGGCTCACGG
SMYD1_2	CAGGAGAAGCTCCATCGCTG
SMYD1_3	TCAGAGAGGCCTGCAGGCCG
SMYD1_4	TGAGCGGGCTTATTCCGCAG
SMYD1_5	CATCTCGCACATCTTCGGAG
SMYD2_1	AGAAGCGCTCCAGGCCGCCG
SMYD2_2	GTGCCAGGAGTGTACCACCA
SMYD2_3	GTTAGTCTTACAGTCTCCGA
SMYD2_4	TACAGCTCTGACTTCTGCCA
SMYD2_5	CCTGTGCAAAGAGTACTACG
SMYD3_1	AGCAGTCCGAGACATCGAGG
SMYD3_2	AGGAAGCCATCCCGTCAGAG
SMYD3_3	ATGTCATTACGAGTTGCCTG
SMYD3_4	GGAGCGCCGGAAGCAGCTGA
SMYD3_5	AGAAGTCGAACGGAGTCTGG
SMYD4_1	AAGCAGCATGGATCTGCCTG
SMYD4_2	ACATTCAAGGCCTAACACTG
SMYD4_3	GGGATGGCTCACCATAGCAG
SMYD4_4	TGGGACACCAGAGTCACCAA
SMYD4_5	GACACTTAATTATGGCCTAG
SMYD5_1	AATGCACTTTATCGCTACCG
SMYD5_2	GAAGGAGAACACGTCGCACA
SMYD5_3	TACCACCAGGTCCTGTGCCC
SMYD5_4	TCTCTAGTGCCCTAAGGCAG
SMYD5_5	AGCCACTGAGCAATACCACC
SNAI1_1	AACAAGGAATACCTCAGCCT
SNAI1_2	CCTAGAGAAGGCCTTCCCGC
SNAI1_3	GAGCCGGTGAGGGTGGGCTG
SNAI1_4	GCGAAGGCACGGCTGCAGTG
SNAI1_5	GATGAGCATTGGCAGCGAGG
SNAI2_1	AAAGCCAAACTACAGCGAAC
SNAI2_2	GAAATGCTTCTTGACCAGGA
SNAI2_3	GACAAGGAATATGTGAGCCT
SNAI2_4	TTGCAGACAGGTCAAATCTG
SNAI2_5	ACTCACTCGCCCCAAAGATG
SNAI3_1	ACGGCCGAGGAGCGGTCCCA
SNAI3_2	GAAGCACTCAAAGCCGCCCG
SNAI3_3	GCGTTTGCAGATGGGCCCGA
SNAI3_4	GGAGATGCAGGCGACGGCCG
SNAI3_5	ACAGGGTCCCCAACTACCGG
SNAPC2_1	ACAGCGAACTGAAATCGCCT
SNAPC2_2	ATTCAGAAAGTGCATCCGGG

SNAPC2_3	CAGGAGCAAAGGCTTTCCAC
SNAPC2_4	GCATGAAGCCACCTCCCAGG
SNAPC2_5	CAGGAGGGTGACCGGTTCCG
SNAPC4_1	CAAGGACAAGGTCACGGGCG
SNAPC4_2	CCAACAGTTACCGAGATCGG
SNAPC4_3	GCTGAACATGGTCTACCAGG
SNAPC4_4	TGATTCTGAGATCTCCACGT
SNAPC4_5	AGCAGCCGGCAGTATCGATG
SNAPC5_1	AAAGAGTCATGTGACGGAAG
SNAPC5_2	ACTCTTTGTGCTCAGCTCCA
SNAPC5_3	GAGCACAAAGAGTCATGTGA
SNAPC5_4	GAGTCATGTGACGGAAGAGG
SNAPC5_5	AATGATCAGTTCTAGAAGAG
SND1_1	TAACCAGGTAGTAATCTGGG
SND1_2	TCAGGAGGAGGCCCACCACG
SND1_3	TCGATTGCAGTTTACACCCG
SND1_4	TCTCGAGCTGGAAATGCCCA
SND1_5	AGTATACCATTGAAAACCCA
SNIP1_1	AAAGGAGAACGTCTAACGAG
SNIP1_2	AATAGACAGGAAAGATGACG
SNIP1_3	AGCCGGCGAAGACACCGGGA
SNIP1_4	TGAAGAGCGAACGGGAGCGA
SNIP1_5	CACCGCCGTCCGGACCACTC
SNRNP200_1	CTCCTGTGGGTTCATCCCGG
SNRNP200_2	GAAGAAGGATTTGCACCCTC
SNRNP200_3	TAGGCGAAGAAAGCGTGATG
SNRNP200_4	TCTGCAATACGAGTACAAGG
SNRNP200_5	GAAACCATGGATCTCGACCA
SNW1_1	AAGGCTCTTCAACCAATCCA
SNW1_2	GCAAAGAATACCTTTCGGCT
SNW1_3	GCATGACCGGAATCTTTCCA
SNW1_4	TAAACAGAGGGTTATTCGGA
SNW1_5	CCTTCCGATCAGCAATGTAG
SOHLH1_1	AATGTGGCACTCGTTGCAGG
SOHLH1_2	CCGGCGGGAGGACATGGCCT
SOHLH1_3	GCGGAACGTGATCAGCGAGA
SOHLH1_4	GTGCTCCGAGCCCTACCCGG
SOHLH1_5	GGCGGAACGTGATCAGCGAG
SOHLH2_1	GCACTGCCAGATCTCGGGCC
SOHLH2_2	GCAGATAATTGAGGAAGCCA
SOHLH2_3	GTGCTCCTGGCAGATAATTG
SOHLH2_4	AGGTCCTACAGCGAACACCT
SOHLH2_5	CATCACCATCAGTGACACGA
SON_1	AATGCTAGCGTCCAACACCA
SON_2	GATGCTAGCATCCAACACCA
SON_3	GATGTTAGCAACCAGTTCCA
SON_4	GATGTTAGCGTCTAGCACCA
SON_5	GCCAGTTGTAACAATGTCAG

SOWAHB_1	AGCCCTTGAAGAGCTCGCGG
SOWAHB_2	AGGAACGGCACCCAGCCCAA
SOWAHB_3	CAGGAACGGCACCCAGCCCA
SOWAHB_4	TCAGGAGGAGCTCTCTGGCA
SOWAHB_5	GAGAGTGCGAGGAAAGACAG
SOX1_1	AAGGCGCTAGATGTGCGTCA
SOX1_2	CAAGAAGGACAAGTACTCGC
SOX1_3	CGAGTGGAAGGTCATGTCCG
SOX1_4	CGCGCACCAGAACTCGGCCG
SOX1_5	CCCATGCACCGCTACGACAT
SOX10_1	CAGGCGGCGGAAGAACGGGA
SOX10_2	GAAGGAGCAGCAGGACGGCG
SOX10_3	GGTCAGAGTAGTCAAACTGG
SOX10_4	GTAGTGGGCCTGGATGGCGG
SOX10_5	AGGGCTCCCCCATGTCAGAT
SOX11_1	CCAGAGAAGAGCGCGGCCGG
SOX11_2	GCTGAGCGAGATGATCGCGG
SOX11_3	GTAGAGGCTCGCTCCCTCGG
SOX11_4	TATGGTCCAAGATCGAACGC
SOX11_5	AAGGGCTCCAGCAAGAAATG
SOX12_1	AAGGCCCTCCCGCTGCTCGG
SOX12_2	GCTGTGGAGGATGGTCCCGG
SOX12_3	GGTTACCGAGATGATCGCGG
SOX12_4	TCGAAGTGCGACGTGCCCGA
SOX12_5	CGGCCGCGCAAAAAGAGCAA
SOX13_1	ACTGGACAACAGCTTCTCTA
SOX13_2	GAACTGCACCATCAAGTCAG
SOX13_3	GCAGCAGGAGGCTTACCCAG
SOX13_4	GCAGGACTGTAGCTCTCCAG
SOX13_5	AAGGCTGGGATCATGACATA
SOX14_1	AAGGCGGCTGGCCTGCCCGT
SOX14_2	GAAGAGGATGTAGGCGACGG
SOX14_3	TACAGGGCACCACGTAGCCA
SOX14_4	TGGGAAGAGGATGTAGGCGA
SOX14_5	CGAATGGAAGCTTCTGTCCG
SOX15_1	AGAGTGACCCTAGGCTCCAG
SOX15_2	GAGCTCTTGGCCTTGCGCCG
SOX15_3	GCGACCACCCAACCGAGCAG
SOX15_4	TATGGAGTGGGAGAGCCAGG
SOX15_5	CCGACTACAAGTACCGGCCT
SOX17_1	AGGGCGAGTCCCGTATCCGG
SOX17_2	GAACAGCGGAGCACCGGCCG
SOX17_3	GCTGGCGTCGGACACCACCG
SOX17_4	TAGTGGCCGCCCATGTGCGG
SOX17_5	GAGCCCCATCGGGGACATGA
SOX18_1	CACGTGGTACGGGAGCCCGG
SOX18_2	GGGCCCATGCACAGTCGCGG
SOX18_3	GGGCTGAGGCCATAGCGCCC

SOX18_4	GTGGAAGGAGCTGAACGCGG
SOX18_5	CAGACGAGTCGCGCATCCGG
SOX2_1	GATAAGTACACGCTGCCCGG
SOX2_2	GGAGCCAAGAGCCATGCCAG
SOX2_3	TGGAGCCAAGAGCCATGCCA
SOX2_4	TGGGAGGAAGAGGTAACCAC
SOX2_5	ATTATAAATACCGGCCCCGG
SOX21_1	CAGGCCGTAGGGCACCGGGA
SOX21_2	CGAGAGCATGTCCAAGCCGG
SOX21_3	CGGGAAGAAGACGCGTGCGG
SOX21_4	TAGCGCGGCAGCGTAGGCCG
SOX21_5	AGCGTCTTGGGCTTGCGCCG
SOX3_1	AGGGACACCCACACAAGCGG
SOX3_2	AGTAGTGCGAACGCAGCCGG
SOX3_3	GATAAGCCTACCCTTCCCGC
SOX3_4	GGGAGGAGAAGGCGCTCCCG
SOX3_5	CAGAAGGCTGTACATTGCCG
SOX30_1	AATCCCATCACTCATCCAGT
SOX30_2	AGAGCTGATAAGATTGACCA
SOX30_3	GAAACGCTGGAATACTCCCT
SOX30_4	TATAACAGTCATAGCCACAG
SOX30_5	GAGAGACTCGATGCAAGGCG
SOX4_1	AAGCGCGTCTACCTGTTCGG
SOX4_2	AAGGTCGGTGGCAGTGGCGG
SOX4_3	GAGAAGGGAGACAAGGTCGG
SOX4_4	GCCGAGGAGGACGCGTGCGA
SOX4_5	CTTGCACCAGCTCGGGTCGT
SOX5_1	AGCAGATGGAGAGGTAGCCA
SOX5_2	GAGAAAGAAAGGCAACTCAT
SOX5_3	GGAACTCCTGAACGGCGCAA
SOX5_4	TATAGCTGAAGCCTGGAGGG
SOX5_5	AAATGAAGGAGCAACTCCGA
SOX6_1	GACACCTTTGAACATGGTGG
SOX6_2	GATCAGCTGGGTAATCATGG
SOX6_3	GCACAACAAACCTCACTCTG
SOX6_4	TCACCACATAAGCCTGACGA
SOX6_5	ATGACTCGGACTGAACAAGA
SOX7_1	GAAGAGAAGCGGCAGCCGGG
SOX7_2	GAGAAGAGAAGCGGCAGCCG
SOX7_3	GCTCCGAGAGCCGTATCCGG
SOX7_4	GGGAGCCTACCCTTGGCCCG
SOX7_5	ACTACAAGTACCGGCCGCGC
SOX8_1	ACTCTTGTGGGCCCACACGG
SOX8_2	GGGCGCCTACTTCCACGCCG
SOX8_3	TGTACACGGCACCGCCGCCA
SOX8_4	TTGGCGCTCTTCCTGCGCCG
SOX8_5	CACCATGGCGACCACACAGG
SOX9_1	ACTCACCCGAGTGCTCGCCG

SOX9_2	GTGTCCGAGCCGGAGCCCGA
SOX9_3	TCTGAAGAAGGAGAGCGAGG
SOX9_4	TTGTCCACAGGGCAATCCCA
SOX9_5	ACCATGTCCGAGGACTCCGC
SP1_1	AAGCCGGTCGGAGGACCCGG
SP1_2	ACAGCCACACAACTTTCACA
SP1_3	ATGGCAAGACCTCTCACCTG
SP1_4	TTTAGACCAAGATCACTCCA
SP1_5	CAACAGATTATCACAAATCG
SP100_1	AGTGACAAAGATGATTCGCT
SP100_2	GAGGCAGAGCTACACAACCA
SP100_3	TGATGAGATCACGATCACGG
SP100_4	TTGGACAGCAATTTGTTCGC
SP100_5	AGTCAAGCATCTGACATGAT
SP110_1	CCAGATTGGGATATTCACGC
SP110_2	GGTGTGCTGGACACCTCCTG
SP110_3	GTTCACCATGACAAGAGCCA
SP110_4	TTACCTGGCATAGAGCCCAA
SP110_5	ATTTACAGAAGCTTCAAACG
SP140_1	AAGGAGCTCAGTCTACTACC
SP140_2	AGATGAAGGAGCGGTCTCGG
SP140_3	GTTCAGCAGGATTAACCTGA
SP140_4	TAGGGAGCTAGAGGCTTCCT
SP140_5	CTAGCTCCCTAGCAAGACGT
SP140L_1	AAGGAAAGTGACCAAGCATG
SP140L_2	ACTCGAGACCAGCTGGCCAT
SP140L_3	ACTTACTTCTCACTTTCCAC
SP140L_4	CACACAGCCAAAGTCTGCAA
SP140L_5	CCAGATAACAGCAAAGCCGA
SP2_1	ACTGGGACTCACAGCAGCAG
SP2_2	GCTGCAAATGGAACAAGCCC
SP2_3	GGTGCTGATCGAGACCACCG
SP2_4	TGATGGTGACAGGCACGCCC
SP2_5	AGGCGTGCGGATGTAGACCT
SP3_1	ACAGCACGGAAACGGTGCGG
SP3_2	CGCGGGACTTACCGCTCCGG
SP3_3	GCTGCTGCAGATACTCGCCG
SP3_4	TAGGAGGAGCACCAAACCGA
SP3_5	ACTGCAGGCATTAATGCCGA
SP4_1	AAGAGGTACAACCTGGCAAG
SP4_2	AGATGAGTCCAGTTCTCCCG
SP4_3	GCAGCGGCGATGGCTACAGA
SP4_4	GTAGCTGCCAGTAAAGCCAG
SP4_5	ACAACCCTACCAATTAACAT
SP5_1	GCAAGCCGTAGCCATGGCCG
SP5_2	GTGGCGGCCAGCAATGCCAG
SP5_3	GTTCCGGAGGACGGCCACCG
SP5_4	TAAGAAGCTCAAAGTCGCTG

SP5_5	AGAACTCGTAGGGGTACGAG
SP6_1	AAAGGCAAACGCGAGGCCGA
SP6_2	CAAAGGCAAACGCGAGGCCG
SP6_3	CGCGTCCCAAAGGCTCCCGG
SP6_4	GCAGGGCTATGAGCTGCCAG
SP6_5	CTCCTCGCGGGTAACCTGCG
SP7_1	CAGGCAACACTCCTACTCCA
SP7_2	GCAACACTCCTACTCCATGG
SP7_3	GTAATCATTAGCATAGCCTG
SP7_4	TGTCTATAAACCCAAGGCAG
SP7_5	CCAGTGCTTGCACCCCGTGG
SP8_1	AACAGAGTAGTCGTTGGCGA
SP8_2	GAACGGCGGCTCGTCCTCGG
SP8_3	GCTGCAGGGCATCTACCCGC
SP8_4	GTAGCGGCAAGCATGGCCAG
SP8_5	AGGACGAGGAGCGTTTCCAG
SP9_1	AAAGGGCAGCGACAGTGACA
SP9_2	CAGCCTCTCGGGCTTCGCGG
SP9_3	GTGAAAGCCGCCTTTGGCGA
SP9_4	TATGGCCACGTCTATACTCG
SP9_5	CGCCATGATATCGGGCGCCG
SPC24_1	ACATAGAGGAGGTGAGCCAG
SPC24_2	GGAGGAGGACACCCGTCTGA
SPC24_3	GGTGCCGAGAAGCAGCTGCG
SPC24_4	TGGCAGAACCTACACGGCCG
SPC24_5	CCAGAGCCTTCTCAATGCGA
SPC25_1	AATGAAGCAAGGGACTATGA
SPC25_2	TACGGACACCTCCTGTCAGA
SPC25_3	TAGTCCCGCCATCTGACAGG
SPC25_4	TCTGCTAGGCCCTCAAGATG
SPC25_5	AGTACTGACTGCAAATATCC
SPDEF_1	AAGCTGCTCAACATCACCGC
SPDEF_2	CTCCTGGAAGGCCTTGCCCA
SPDEF_3	GGTGCACGAACTGGTAGACG
SPDEF_4	TAATGAAGCGGCCATAGCTG
SPDEF_5	CCACCCGCCACGCCCGAGCA
SPEN_1	GAAACCAGGCATCTCTGGGT
SPEN_2	GAGCCCTAGGTTCATATCGG
SPEN_3	GCAGACTGAACTGATCGAGC
SPEN_4	GGGTGACAGAGACCTACGCA
SPEN_5	AAGGAGCGTCTATGCAACCA
SPI1_1	AGCGGCGAAGTGCTGGGCCG
SPI1_2	GCAGCAGCTCTACCGCCACA
SPI1_3	GCCGTCAGACACCTCCAGTG
SPI1_4	GGAGTCCCGGTACTCACAGG
SPI1_5	AGACCTGGTGCCCTATGACA
SPIB_1	AGAGGAGGAAGACTTACCGT
SPIB_2	CTGGCTAGCGAAGTTCTCCG

SPIB_3	TCGGGTGTGCTCAGGCCCGG
SPIB_4	TGGCTAGCGAAGTTCTCCGT
SPIB_5	CCAGCTACCCTGATTCAGAG
SPIC_1	GCTGACTTACCAGTTCAGTG
SPIC_2	GTTCTGAGGCAACATTCAAC
SPIC_3	TGTGTTGAACAAGACAAGCT
SPIC_4	TTCCTTTGACATGAGGACGA
SPIC_5	ACTCTTCTCCAGCAAAAGGG
SPO11_1	AACGTCGAAGAACGAGGCCT
SPO11_2	ACCAAAGTGAATTGTACCTG
SPO11_3	GCACCAGCTCCTGTACCTGG
SPO11_4	TGAAACTTGACAGTATCCTG
SPO11_5	GATAGACAACAGATCAAGCT
SPOP_1	ATTGCTTCAGGCGTTTGCGT
SPOP_2	CCACGGACAGGTTACTGCAG
SPOP_3	GTAGCACCAACTCTCAGCTA
SPOP_4	TGAGAGTTGGTGCTACACAC
SPOP_5	ACAAGGCTATCTTAGCAGGT
SPZ1_1	GAACTCCATCATCAGAAACA
SPZ1_2	GCAGAACAAGCAAGCAATGA
SPZ1_3	TACTACAGGAAGCAGAACAC
SPZ1_4	TGGCTGCTATTCTTTAGGAA
SPZ1_5	ATAAGGCAATGGTAATCCTA
SRA1_1	AGCCCGTATGAGAACTGCGG
SRA1_2	CAGGCATTGGAAGACTGCCG
SRA1_3	CATCTGCTGCGTCCCACCGG
SRA1_4	TTGGTAAGCAGCGAGCGCCT
SRA1_5	AGACTGCCGTGGCCACACAA
SRC_1	GTCATAGAGGGCCACAAAGG
SRC_2	GTCTGACTTCGACAACGCCA
SRC_3	TAGCAACAAGAGCAAGCCCA
SRC_4	TCAATGCAGAGAACCCGAGA
SRC_5	GACCTGGAACGGTACCACCA
SRCAP_1	AATTGCTTCCACCATGGCCA
SRCAP_2	AGACTCGGATTGCTGAGCTG
SRCAP_3	AGGCAGCTGAGGAAGAGCCG
SRCAP_4	GAAGTTGAAGAACAACAGGA
SRCAP_5	AGCTGTCAGAAATCATTGAG
SREBF1_1	CCTGTAGAGAAGCCTCCCGG
SREBF1_2	GCAGACAGGGCCTTTGCCGG
SREBF1_3	GGACGAGCCACCCTTCAGCG
SREBF1_4	GGAGCGGTAGCGCTTCTCAA
SREBF1_5	ACCAGCTGCACACCATGGGT
SREBF2_1	AACTGTCTGCACCGTAGCCG
SREBF2_2	AGAGAAAGTGCCCATTAAGC
SREBF2_3	AGTGCAACGGTCATTCACCC
SREBF2_4	GCTGGGCGACGAGCTGACCC
SREBF2_5	ACTCGAATGACAGGACACTG

SRF_1	AAGCCGGGTAAGAAGACCCG
SRF_2	AGGTTGGTGACTGTGAACGC
SRF_3	GCGAGCTATACCTGTCCCGC
SRF_4	GTGTCGGAGTCTGACAGCAG
SRF_5	AGTTCATCGACAACAAGCTG
SRSF1_1	ATCGACCTCAAGAATCGCCG
SRSF1_2	GACCTCAAGAATCGCCGCGG
SRSF1_3	GACGCGGTGTATGGTCGCGA
SRSF1_4	GAGCGAGATCTGCTATGACG
SRSF1_5	ACCTCCAGACATCCGAACCA
SRSF12_1	CTCGTTCCAAATCATTACCA
SRSF12_2	GACGTTCCTGATGAACAGGG
SRSF12_3	GGCAGTCAAGAACTCCAAGA
SRSF12_4	GTTCCAAATCATTACCAAGG
SRSF12_5	ATGTTTGCAGGTCTCGACAC
SRSF3_1	GGAGTCCTCCACCTCGTCGC
SRSF3_2	GTAAGAGTGGAACTGTCGAA
SRSF3_3	TCCTACGATAATCATCTCGA
SRSF3_4	TTACCTACGAGACCTAGAGA
SRSF3_5	ATGTGGCTGCCGTGTAAGAG
SRSF9_1	AAGGACCCACCTCATGAGAG
SRSF9_2	CTCGAAGCGCACGAAGGCGA
SRSF9_3	GACTCTCCATACCAAAGCAG
SRSF9_4	GAGGCCCATTCCTCCCACCA
SRSF9_5	GAGTTCCCCAGGACTTATGG
SRY_1	AAACAGTAAAGGCAACGTCC
SRY_2	GAAAGCCACACACTCAAGAA
SRY_3	TAAGTATCGACCTCGTCGGA
SRY_4	TGTCCTACAGCTTTGTCCAG
SRY_5	AAGAGAATATTCCCGCTCTC
SS18_1	AATGAGTCAGGGACAACCAA
SS18_2	TCAGATGGAATGGTAGGTGG
SS18_3	TGACGGCGGCAACATGTCTG
SS18_4	TGTTGAGGAGGTCTATAGGG
SS18_5	AATCAGATGACAATGAGTCA
SS18L1_1	ATCCTGGAGTACCAGAGCAA
SS18L1_2	CAGAACATGAACCTGGGCCC
SS18L1_3	TACGCAGCAAACCATCCAGA
SS18L1_4	TGGGCCCGGCTACAGCCACG
SS18L1_5	CCATCGGCAACTACGTGTCT
SS18L2_1	ACTGCACGCACTCGTTCCCG
SS18L2_2	GAGGTTCCTCGGGATGTCGG
SS18L2_3	GAGTATCAGAACAAGGGCCG
SS18L2_4	TCAATCAAGAGACTATCCAG
SS18L2_5	CCTCAGCCAGTCCGGTACGA
SSRP1_1	AAAGTGGACAACATCCAGGC
SSRP1_2	GATGCAGATGGCATCTCCCG
SSRP1_3	GTTCAACGACGTCTATCAGG

SSRP1_4	TGCACTGGGACACATTGCTG
SSRP1_5	AAGCTCAACATCAAAAACCG
ST18_1	AAGTGGTACTCACTTTCCAG
ST18_2	ACTCGCTCTAAAGGAACCGA
ST18_3	AGTGACAGGACAGAGGACGA
ST18_4	CCTGCTGGAGCACCTAGCAG
ST18_5	ACACATGGTCACTCTACCGC
STAG1_1	AGTCTGACAAACCCGTCAAA
STAG1_2	GATGTGCCGAGTACACCAAG
STAG1_3	GGAATTAGAGGAGCAGGCCG
STAG1_4	TTCAATCCAGTCATCCACCA
STAG1_5	ACTACTTCAGAAACGCAAAG
STAG2_1	ATTTCGACATACAAGCACCC
STAG2_2	GCTGAATGTCATCCTCCCGT
STAG2_3	TAATAACTGAGGAAGGGCCA
STAG2_4	TCATCACCAACAGAATGGAG
STAG2_5	AATACTAACCTTGAACCGAC
STAG3_1	ATGAGGCTGAAAGAAACAAG
STAG3_2	CAGTCTGTGGGACTGTGCAG
STAG3_3	CCAGTACAGAAATTCGCCTG
STAG3_4	TAGGGAAGCCATCATAGAGG
STAG3_5	ATGGACCTGGAGCTATGAGA
STAT1_1	AACATGGAGGAGTCCACCAA
STAT1_2	AACGTCAGCCAGCTCCCGAG
STAT1_3	AGAACACGAGACCAATGGTG
STAT1_4	GATCATCCAGCTGTGACAGG
STAT1_5	CCTGATTAATGATGAACTAG
STAT2_1	CAAAGAGCAGAAGATTCTGC
STAT2_2	GAAGAATAGCATGGTAGCCT
STAT2_3	GCAGAGCCCAAATGGCGCAG
STAT2_4	TCTCCAGCTGTTCCAACCCG
STAT2_5	ACCTACCCTTGTAATTGAGG
STAT3_1	CTACAGTGACAGCTTCCCAA
STAT3_2	GAAGGCGTGATTCTTCCCAC
STAT3_3	GCAGGAAGCGGCTATACTGC
STAT3_4	GGAACAGATGCTCACTGCGC
STAT3_5	AACATGGAAGAATCCAACAA
STAT4_1	AAATCCAATGCATGTAGCTG
STAT4_2	CTATGATGACAACTTTCCCA
STAT4_3	GAGTGACAAGAATAGTGCCA
STAT4_4	TTGAAGAAGAATCGTTGCCA
STAT4_5	AACCCACCCTCAGAGGCCGT
STAT5A_1	ACAGAATGTGCCGGATGCAG
STAT5A_2	AGTGGATTGAGAGCCAGCCA
STAT5A_3	GGAGAGGCACAATTACTTGC
STAT5A_4	GTGGCTTGGGCTCTGTCCTG
STAT5A_5	ACATTCTGTACAATGAACAG
STAT5B_1	GCATCAGCAAGGCTTCCAGC

STAT5B_2	TAAGAGGTCAGACCGTCGTG
STAT5B_3	TAAGGCCACCCAGCTCCTGG
STAT5B_4	TGCGGCATTATTTATCCCAG
STAT5B_5	CAGCCAGGACAACAATGCGA
STAT6_1	AAGTGGCCACCAGCTTCAGG
STAT6_2	ACACATCAGCACCCTTGAGG
STAT6_3	AGTCACCCAGAAGATGCCGC
STAT6_4	AGTTTAAGACAGGCTTGCGG
STAT6_5	ATCAAGCGGTGTGAGCGGAA
STK31_1	GCAAGAGAAGGCAGCTGCTG
STK31_2	TTAACCTCAGAACGTACCAA
STK31_3	TTCAAGTCAGATGATCCTGA
STK31_4	TTTGGAACCATGCTACAAGA
STK31_5	GATAGCAAGTGATCCACACT
STK4_1	AACGCCAGGAATCCCAGCAG
STK4_2	AGCTGAGGAACCCGCCGCGC
STK4_3	TGTAGCAGACATCTGGTCCC
STK4_4	TTGTAAAGAGCCCTGAGCAG
STK4_5	AGCTTTGTATACGCTGCCAT
SUB1_1	AGAACAATGGAGCCAGCTGA
SUB1_2	CAGAAATCTGTTCCTTCAGC
SUB1_3	GCTCTGTTTAGAAGATGACA
SUB1_4	CAGAGATGATAACATGTTTC
SUB1_5	CCTGTAAAGAAACAAAAGAC
SUDS3_1	CAGTTGCAACAACTGCAAGA
SUDS3_2	CTGACAATGGAACTGACTGG
SUDS3_3	GAGGCGACATGAGTGCCGCG
SUDS3_4	TCATGACCAGAAAGTTGCGG
SUDS3_5	ACTCACCTTCGTCCGACTCG
SUGP2_1	CTTGTGAAACGTGTCATCGA
SUGP2_2	GCACCGATAACCCTGACCTG
SUGP2_3	TAGGAGGGTCTGGGTCCCGG
SUGP2_4	TATGATGACGTCCACAGCGA
SUGP2_5	AGTGAGCTACAGATCCACTG
SUMO1_1	GTTTATCAGGAACAAACGGG
SUMO1_2	ACCTTCAACTGAGGACTTGG
SUMO1_3	AGAATCATACTGTCAAAGAC
SUMO1_4	GTGTTCCAATGAATTCACTC
SUMO1_5	TAAACTCAAAGTCATTGGAC
SUMO3_1	CATGTCCGAGGAGAAGCCCA
SUMO3_2	CTTGATCTTGAACTGCACCA
SUMO3_3	GAATGACCACATCAACCTGA
SUMO3_4	TGACCACATCAACCTGAAGG
SUMO3_5	CAGATCAGATTCAGGTTCGA
SUPT16H_1	AGACTTGAAGCGAATACCCA
SUPT16H_2	ATGGGAATTGAATTCCGTGA
SUPT16H_3	TACAACAATGGCATCAACGT
SUPT16H_4	TAGACATCATGGCATACCCT

SUPT16H_5	ATATACCATCGCTGTAAAGG
SUPT20H_1	AAATCAAGTAACCTCAGCTG
SUPT20H_2	AGATCCACAGATGATTTCCG
SUPT20H_3	TGATAACCACAAATGGACCC
SUPT20H_4	TTAGTGGTCAATCTATACCC
SUPT20H_5	GATCAAAGACCGATGCTGAG
SUPT3H_1	GAGGCAAGACATGGTAACCA
SUPT3H_2	GATGAGGATGATCTTCTCGA
SUPT3H_3	GATTGTCAAAGGCATCGATG
SUPT3H_4	GGGAAAGTGGGCCAATCCTG
SUPT3H_5	AGTTAAACAAGAAAGAATGG
SUPT4H1_1	AACAGGCCCGCAGATGCCGC
SUPT4H1_2	AATGAAGGGTAACCGAGAGA
SUPT4H1_3	ACTGACACCGCATATACACC
SUPT4H1_4	CATTGCGATGATGAGTCCAG
SUPT4H1_5	AGACGGTGCCGAAGGACCTG
SUPT5H_1	GAAATACGCCAAGTCATCTG
SUPT5H_2	GCGCATCAAGGAAATGGCCG
SUPT5H_3	TGAGGACGAGGACCAGTGGG
SUPT5H_4	TGTAGGTAGACGAAGAGCGG
SUPT5H_5	AAATACGCCAAGTCATCTGT
SUPT6H_1	AGCCCAGGAAATCTTCGGTG
SUPT6H_2	CGAGACAGAGCAGTTTCCCG
SUPT6H_3	GCTGTGATTACCTAGACCGA
SUPT6H_4	TAGCCCAGCACTCTGGCAGA
SUPT6H_5	GGAATACGATGAATCAGCCG
SUPT7L_1	ACATGGGCCAGATTCCAAAG
SUPT7L_2	GTGCTGCCAGAACTTCTGGA
SUPT7L_3	TAAACCTGTGAAGATCAAGG
SUPT7L_4	TCCACCAGACGGAACTCCCG
SUPT7L_5	AATCAACTGAATCGTATGGA
SUPV3L1_1	AATGTGAATGTCCACATCCA
SUPV3L1_2	ATTGTGGGCTCGGCTCCCGG
SUPV3L1_3	CAGATGCTAGAGCCATGCAG
SUPV3L1_4	GGAGAAACTAACCAGTTAGG
SUPV3L1_5	AGTCGGCAGATTGAAATTCG
SUV39H1_1	CGTGGAGGACGTGTACACCG
SUV39H1_2	GTTCCTCTTAGAGATACCGA
SUV39H1_3	TAGAAAGGGAGCTGCTCCGG
SUV39H1_4	TCATAGACAACCTTGACGAG
SUV39H1_5	ACAGGAACAGGAATATTACC
SUV39H2_1	AAAGCTCTACAAGATGGCGG
SUV39H2_2	ACTAGCAATGGACGTGGCTG
SUV39H2_3	TCATAGATGGGAGTACCAGG
SUV39H2_4	TGTGGAGCTGTGACTTGCAG
SUV39H2_5	ATCTGTGCATGAACAACCAA
SUV420H1_1	AAATGCCTTGAGCTCCTCGA
SUV420H1_2	ATCCTCTGGAATGTCCGCCA

SUV420H1_3	GAGAACATGCTACTTAGACA
SUV420H1_4	GGGAAGGACACCCTGAAGGC
SUV420H1_5	AAATAGTTGCAACAAAAGAG
SUV420H2_1	ACTGTGCGAGAACGACGACC
SUV420H2_2	GCAAGCGGAGTGCTCAGCTG
SUV420H2_3	TTGAGGGCAGCCTCCTGCCG
SUV420H2_4	TTTCCGGCAGGAAGGCACGG
SUV420H2_5	CACGGAGGTAGCGATAGACC
SUZ12_1	AAGGAGAGCAAGAATCTCAT
SUZ12_2	CGGCAGTTCAGAGTACACCA
SUZ12_3	GCTTCGGGCGGCAAATCCGG
SUZ12_4	TTACTGGAAACTGCAAGGGA
SUZ12_5	GAGACTCTCTGAATTTCTAG
SVEP1_1	GAGGGCTATGATTTCACAGA
SVEP1_2	TCTGCCCACAGCTTGCCCAT
SVEP1_3	TTCCCACAAGATCAAATCCA
SVEP1_4	TTGTCAAGGAAACAGCCAGT
SVEP1_5	AGTTTGTAAAGACCTAGTTG
SYCP1_1	GAAAGTGGAATCGCCTCCCA
SYCP1_2	GTTTCAGGAATAATGCCACA
SYCP1_3	TAGACACAAACCTTTGGCAA
SYCP1_4	TCTGACTGTCACTATCAGGA
SYCP1_5	GCAAGCTGAGAATTCCAGAC
SYNCRIP_1	ACTGCAGAAGTAGTATCCAT
SYNCRIP_2	GAATAACCGGCTCTACCGCG
SYNCRIP_3	TATAGAAGATCCTGATCCTG
SYNCRIP_4	TTGATGTGACCACTGGACAG
SYNCRIP_5	GATGACAAGAAAAAAAACAG
T_1	ACAGAGCGCGAACTGCGCGT
T_2	TCACCAACAAGCTCAACGGA
T_3	TGATCACAAAGAGATGATGG
T_4	TGGCTGGTGATCATGCGCTG
T_5	CCGCTGGAAGTACGTGAACG
TADA1_1	AACGCGTGAGAATGGCCAGG
TADA1_2	ACTGGGCTAACCTAAAGCTG
TADA1_3	GGCCAAGAAGAACTTAAGCG
TADA1_4	TGGGCTGGACAATGTCACCG
TADA1_5	AAAGCTTATCGGTTACGAGA
TADA2A_1	AAGGAGTCAAAGGTAGGTCG
TADA2A_2	GCTGAACCTGAAACAAGCAG
TADA2A_3	TCTGATAAGCCACCTTGCCG
TADA2A_4	TGTAGCTCTGAAGATGGCTG
TADA2A_5	ACTTGATATAAGGCTCCATG
TADA2B_1	AGCTGAAAGAGAGACAGCGG
TADA2B_2	GCTGAAGCGCAAGATCACCA
TADA2B_3	GTGGTGGCCGATCTCGGCGC
TADA2B_4	TCCGAAGCCGAACTGCTCGA
TADA2B_5	ACAGACCACACCTGTCCCAG

TADA3_1	AAGTGTGCGGACCTCCTCGC
TADA3_2	ATTTGGGCCGTCCTGGGCCA
TADA3_3	GGAAGCACTACTCCCAGCGC
TADA3_4	TTTCGGCCTCAAGCACACGC
TADA3_5	ATGACCCTATCGACGTGCCA
TAF1_1	AAGCCAGGTCCACCTGTCAG
TAF1_2	GACAGCGACGAAGATTCCGC
TAF1_3	GAGGAGGAAGAGCAGCGCTC
TAF1_4	TGGGTATGAGGTATCAGAGG
TAF1_5	CATACGGACTACAAAAGATG
TAF10_1	CCGCAAGTACACTCTAACCA
TAF10_2	GCTGGAAGATTACACGCCTA
TAF10_3	GCTGGCCTTGTTCTCCGCGG
TAF10_4	TTCGGGCGGAAGCCCACCGT
TAF10_5	AGAACAAGGCCAGCCCCGCG
TAF11_1	CTTGAAAGAAGCTGCAGCGG
TAF11_2	GGAATCCCAGAGGAAACTGA
TAF11_3	GGACTTGAAAGAAGCTGCAG
TAF11_4	TCGCCGCTCAGCTTTCCCTA
TAF11_5	AGCGGCGATACATTTCATAA
TAF12_1	AAAGATACCAGGCACTCCTG
TAF12_2	ACAGGACTTAGTAAGAGAAG
TAF12_3	GCATCGCAAGTCTAGCACCC
TAF12_4	GGAGAGGTTGATTAGGGCTG
TAF12_5	ACTGCAGTGGTAAAGATACC
TAF13_1	AGTAAGCAAGTCTTTAACCC
TAF13_2	ATTGGAGGAGGTGCAGAAGG
TAF13_3	GAAATTGGAGGAGGTGCAGA
TAF13_4	GGACCCAAGGAAGTTTGCCA
TAF13_5	ATGTATGATGTATGGCTTTG
TAF15_1	GGTGGCTATGGAGGAGACCG
TAF15_2	GGTTATGGAGGAGATCGAGG
TAF15_3	TACGGTGGAGACCGAAGTGG
TAF15_4	TATGGAGGAGACAGAAGCCG
TAF15_5	AGCAGTCCTATCATTCACAA
TAF1A_1	AAGAAGAACACCGTAAACTG
TAF1A_2	GCTGTGGTTGAACACATCCT
TAF1A_3	TCTACGTATGTTTGAAGCCA
TAF1A_4	TTCCAGAAACCACCTTGCGT
TAF1A_5	ATAGATGCTGGAATTCTATG
TAF1B_1	AAGAACCCAGTTTATACCAC
TAF1B_2	GAAGGCAAGTGTCTGTGGCA
TAF1B_3	TATTGGATGACAGTTTCGAG
TAF1B_4	TTAACTCTGGGCCTACTCCA
TAF1B_5	AAATAAAAGCCCTCAACCGG
TAF1C_1	AAGCTGCTCCAGGACCTCGG
TAF1C_2	AGGGACGTCGCTCAGACCAA
TAF1C_3	TCTGGATGAGGCCTGCACTG

TAF1C_4	TTTCAAGCTGGAGGGAGCGG
TAF1C_5	ACAGACGAGATGAAGAGTAG
TAF1L_1	AGCGAGGAAGATTCATCTGG
TAF1L_2	GGGTGAGGAAGACTCTGATG
TAF1L_3	TGAAGGGTATGATGATGAGG
TAF1L_4	TGACTTCAAACGCACAGGGA
TAF1L_5	CAAAACAGGTGTCATCAGGG
TAF2_1	AAGGTGTTTAGCCCAATCCT
TAF2_2	GATGTGGTATGTTTCAGCAA
TAF2_3	TTAAGGTTCCATCAGAGCTA
TAF2_4	TTCAGCCGTTAATAAAGCAG
TAF2_5	ACTGTGTAAAAGATTTGTGC
TAF3_1	AGTCACCACGCTGCGCACGG
TAF3_2	GGGTGTAACAAGCCTGACGA
TAF3_3	TATCTGCAGCAGCTGGGCCG
TAF3_4	TCAGCTTCAGGACTATCCAG
TAF3_5	CCTTTAGTGCTTAATAGCCG
TAF4_1	AAGCTGAGGCCGCCGCCCGA
TAF4_2	ACGGTGGAGATCTGGACGGG
TAF4_3	AGTGTCTCAGGCCCAGACAA
TAF4_4	GCGCCACCCAGAACGAGCTG
TAF4_5	GACCGAGCTACTCAGCACCA
TAF4B_1	GAAACAACAGGTTTCACAGA
TAF4B_2	GCGGGTGGAGAGCACTCCGG
TAF4B_3	TGAGGAAGGCTTCGGAACAG
TAF4B_4	TGAGGAGATACCAACATCAA
TAF4B_5	ACATTGACCATTCAGAAATG
TAF5_1	ACCACCAAACGAGTGCGCCT
TAF5_2	GGCGCTGTCCACCTCTCCCG
TAF5_3	GTTGGGACAACTCTGCCCGA
TAF5_4	TAAGCAACAGATAGATGCGA
TAF5_5	AGCGGCGGCACTACCAACAA
TAF5L_1	AAACAGTCCGAAGAGCACAG
TAF5L_2	AGACGCCAAGTACTTACCGT
TAF5L_3	GCGTACCGAGCAGATTCAGA
TAF5L_4	GTAAGGTGAGGACTTTGCAC
TAF5L_5	CGGGACACGTCTACTTGGTG
TAF6_1	AAGGAATGAACTCCTGGGCG
TAF6_2	AAGGTGGTGGCTGAATCCAT
TAF6_3	GGAGGACTGACCTTTGCCGT
TAF6_4	GGCGTAGTCAATGTCACTGG
TAF6_5	GACAACTCGTGGATGCTCCG
TAF6L_1	GTAGAGTTCACCCTCCCTGG
TAF6L_2	TCATGAAGCACACCAAACGC
TAF6L_3	TGAGCCGGACAGACTCCCGA
TAF6L_4	TGTCAGTGAAGACACAGCAC
TAF6L_5	AGATCCTGGCAGATCCTGTG
TAF7_1	AATATGCCTCTACTGTGAGA

TAF7_2	ACAGTAGAGGCATATTCTGG
TAF7_3	ATAGAGATCACCATCAACTG
TAF7_4	TTAGAGGCAGAGTAATTCCG
TAF7_5	CTGAAGCTGTTAGTACTCGG
TAF7L_1	AACGCTGCAACTGTTCCTGT
TAF7L_2	CATAGGTGGTTTCAGACCCA
TAF7L_3	GAGATACAAGTTCTTACCAG
TAF7L_4	TTAGGATCAGTAGAGGCAGC
TAF7L_5	CGAGGACACTCAAACGGATG
TAF8_1	ATAACTATCATCTGGCCCGG
TAF8_2	GAACAAGATGGCCGACGCGG
TAF8_3	GGGAGAAGGCTGCATCCCAG
TAF8_4	TGTCTGTTCATCCTGCTCCG
TAF8_5	CCAGATGATAGTTATCGGCA
TAF9_1	ACTATATGAAACCAGCGTTC
TAF9_2	GTGGGTGATTTAGCTCGAGA
TAF9_3	AATTGTGGTCACATATCGGA
TAF9_4	ACTGAACCAACACTTAACCG
TAF9_5	AGGTAAAAGACTGATCAGCG
TAF9B_1	ACAGCACCAACACTTAATCG
TAF9B_2	ATCGTGGAACTAGTCTCCCT
TAF9B_3	GTTAAGCAGTATCTATCAGG
TAF9B_4	TATGGCTTAATCAGTGGCAA
TAF9B_5	ATCCCCTAGGGACCTAACCA
TAL1_1	AAAGGGCCGCGACGCGGCGA
TAL1_2	GTGGCCCAGACCCATCACCG
TAL1_3	GTTGGTGGTGAACATAGGGA
TAL1_4	TGTAACCGAGGCGGGCGCGG
TAL1_5	GATGTGTGGGGATCAGCTTG
TAL2_1	AAACGGGAGTGGCTGCTCAG
TAL2_2	AAGCCTGCAACAAACGGGAG
TAL2_3	TCACAAATACCAGGGAGCGG
TAL2_4	TGTCCTCCAGGCCTGGCAGG
TAL2_5	AAACGCTTCGCCTGGCAATG
TARBP2_1	GCAAGGCTCCGGCACTACCA
TARBP2_2	GCCCTCAAACACCTCAAAGG
TARBP2_3	GTACGACCTTCTCAAAGCCG
TARBP2_4	GTTGCACTCAGACTGCTGAG
TARBP2_5	GCTTTGAGAAGGTCGTACAC
TBL1X_1	CAATGGAACACTCTTGGCTA
TBL1X_2	GATCTGGAGCATGAAACAGG
TBL1X_3	GTTGAGGCACTGTATACGAG
TBL1X_4	TTGGTGGGAAACGCCGGCTG
TBL1X_5	ATAATTTGGGATGCCCACAC
TBL1XR1_1	AGGGCCTTTATGCTGCCCTA
TBL1XR1_2	ATAGAAATCAATGCAGCGGG
TBL1XR1_3	ATGCTAGGAGATCACTAACA
TBL1XR1_4	GAGGCCAAGAGATTGCCAGT

TBL1XR1_5	GCAAGATGTTCCAAGCAACA
TBL1Y_1	AAATCCTCCAAAGAACCGAG
TBL1Y_2	ACTAGCCAAGTACTTTCCAT
TBL1Y_3	ATCCTGACTTCCTTACCCAG
TBL1Y_4	TGATGGAACACTATTGGCTA
TBL1Y_5	AGGCTAGCAAATCACTGACA
TBP_1	AAACGCCGAATATAATCCCA
TBP_2	GATAAGAGAGCCACGAACCA
TBP_3	GATTCAGGAAGACGACGTAA
TBP_4	TCAGCAGTCAACGTCCCAGC
TBP_5	ACGTCCCAGCAGGCAACACA
TBPL1_1	AAAGGCTGTTGCTACTGCTG
TBPL1_2	AGAACAATAGACCTCATGCC
TBPL1_3	AGTAGCAACAGCCTTTACAT
TBPL1_4	TCAAAGACTTACCTGGCATG
TBPL1_5	ATTTAGCTTCTTCTTCACTA
TBPL2_1	GGTACAACTGGGCTGAACAG
TBPL2_2	GTAGGTCTCCTCCTGCTCCA
TBPL2_3	TAATGAGGATCCGAGAGCCC
TBPL2_4	TGTGAGATTTCCCATCAGGC
TBPL2_5	AACAGTGGGATTACGGTCCA
TBR1_1	AGTGAACGAGGACGGCACGG
TBR1_2	GAAGGACTTGCCTTGCACAT
TBR1_3	TGACTCCAAGGACTCACCAG
TBR1_4	TTTACGAGCAGGCCAAGCGG
TBR1_5	AGGAGTGGCCGTACTGCTGT
TBX1_1	AGGCCGCGTGCACTACCACC
TBX1_2	GAGCAGCATATAGTCGGCCA
TBX1_3	GCACGCCTACCCGTTTGCGC
TBX1_4	GCACTTCAGCACCGTCACCA
TBX1_5	AACAACCTACTGGACGACAA
TBX10_1	CGCCAGCCAAGGGTGCCCAG
TBX10_2	CTGAGGGTGCAAGTATGCCG
TBX10_3	GAGCAGGGCGTAGTCGGCCA
TBX10_4	GAGCTGGTTCACCCACCCTG
TBX10_5	TGGATGACAATGGCCACGTG
TBX15_1	ATTGCCAGCCACCATCCACT
TBX15_2	CGGAGACACGGAGGACGCGG
TBX15_3	TATCATGGAACCGCTTCCAG
TBX15_4	TCAGCAGTACTACATAGCAA
TBX15_5	ATTCTGATAGGCCGTAACTG
TBX18_1	CAAGGCGCACGCTTTCTCGG
TBX18_2	CCAGCAATATTACATTGCCA
TBX18_3	GAGACTCCGGGCGCAACAGG
TBX18_4	GCTCCCTGCAGATCCACCCG
TBX18_5	CATTCTGGCGACCATCACTA
TBX19_1	ACAGCAATGCTGGTTAGCGA
TBX19_2	GACCAATCAATCCAGGGCCC

TBX19_3	GCAACTTCAGATCATCCTGG
TBX19_4	TAGGGACAAAGTCCAGCAGG
TBX19_5	ACACGCACTATGTGAACCTG
TBX2_1	CGACAACAACCCGTTTGCCA
TBX2_2	CGGGTGGTAAGCCATGGCGC
TBX2_3	GCAGTGGATGGCTAAGCCTG
TBX2_4	TCTCCGGGAACACGTAGGTG
TBX2_5	CCAGTTCCACAAGCTAGGCA
TBX20_1	ACGAACCTGGCTGGCAACGG
TBX20_2	CAGGCTGGTCAGCTCACCCA
TBX20_3	GGGTAGCCCAACTTACCCAG
TBX20_4	TGAGCAACTACTCAAACAGA
TBX20_5	ATTGCCGCGCTCATGTCGAG
TBX21_1	AAAGCTCACAAACAACAAGG
TBX21_2	ACCTCAACGATATGCAGCCG
TBX21_3	GTGCAGTGTGGAAAGGCCGA
TBX21_4	TGAGGACTACGCGCTACCCG
TBX21_5	CCGGGGCTGGTACTTATGGA
TBX22_1	ACGTCTATCACAGCTCACAG
TBX22_2	GCTGTCGCTGCCGCAGCCAG
TBX22_3	TCGGGCGCGTGCCTTCTCCG
TBX22_4	TTTCACCTTGACCCGAACAG
TBX22_5	ACTCCAAGACCCAATACAGG
TBX3_1	AAAGAGGATGTACATTCACC
TBX3_2	GAGGTTCGATGTCCCTACAG
TBX3_3	GTGATCACGCTACGTGGCAG
TBX3_4	GTTGGGAGGCAGCGTCAGCG
TBX3_5	ATAAATTTCACAATTCTCGG
TBX4_1	GAAGCTGACAAACAACCACC
TBX4_2	GCTCTGGAAGAAGTTCCACG
TBX4_3	TGCCTAGCGCGGCTCCGCTG
TBX4_4	TGTCACAGAACTTGTAGCGA
TBX4_5	CAGGGGGCCCACGTCCGGTG
TBX5_1	ACAGGACAGAGTCGGCACAG
TBX5_2	ACCATGGCCGACGCAGACGA
TBX5_3	CACGGAAATGATCATAACCA
TBX5_4	GGTGAGCTTGAGTTTCTGGA
TBX5_5	CCTTCATCTAGGTCTGTGAC
TBX6_1	GACAGCCTACCAGAACCCAC
TBX6_2	GGCCCTCCATTCCCTCCCGG
TBX6_3	GTAGCGGTAGCCCTCCGCTA
TBX6_4	TCTTCTGGATGTGATTCCGG
TBX6_5	GAAACAAGTAGCGGGCCTCG
TCEA1_1	CAGGAGGAATATTCCCACAG
TCEA1_2	GCTGCAGCTCTTCGAACAGG
TCEA1_3	TATGACCCTGGAATTACTGC
TCEA1_4	TCAGAAGTGCTTGGTGCCCG
TCEA1_5	ACATACCTGCAGTAATTCCA

TCEA2_1	AGATTGCGCGGATCGCCCGG
TCEA2_2	AGGGAGCCATGGATTTGCTG
TCEA2_3	ATGCCAAAGCCAGGGAGCGG
TCEA2_4	GAAGCAGAGCTCGGATGAGG
TCEA2_5	CCAGGGCACCGTCGACTCCG
TCEA3_1	AAGCAGGCCTTTCTCCACCA
TCEA3_2	CATGGAAGAGGCAAACGTGG
TCEA3_3	TATAGCCAAGATGACGGCAG
TCEA3_4	TTTGATAAGGACTTTGGCCA
TCEA3_5	AAACAGCAGCAAATCAAAAG
TCEANC_1	AGCAGACTCACTCCAAAGCG
TCEANC_2	GATGGTGACCCTGAATCCAC
TCEANC_3	GCAGTTGAGAGCCTCCTACA
TCEANC_4	TTTAGGGCTGTTCCTCGCTT
TCEANC_5	ATCAACCCAAAGCTGATTTG
TCEANC2_1	CCTCAGAAGAAAGATTCTGG
TCEANC2_2	GGTACGGCCCATTAATGAGG
TCEANC2_3	TAATGGGCCGTACCGGCGGA
TCEANC2_4	TCATTAATGGGCCGTACCGG
TCEANC2_5	ATCCCAAAACCGAGTCGTTG
TCEB1_1	ACGATAAAAGCCATGTTGAG
TCEB1_2	ACTTTCGATAGCACATGTGA
TCEB1_3	AGGTCCTTCACAGCCACCAT
TCEB1_4	GTCAAATTGATATCATCTGA
TCEB2_1	CGAGCAGCGGCTGTACAAGG
TCEB2_2	GAACTGAAGCGCATCGTCGA
TCEB2_3	GCACTGCTTCCCGAGTCCTG
TCEB2_4	GGACGCCAAGGAGTCCAGCA
TCEB2_5	CGAACTGAAGCGCATCGTCG
TCEB3_1	AGCAGGAGACCTGTTGCCCA
TCEB3_2	GAAGGAGGATATCAGCTCGA
TCEB3_3	GCATGTTGGAAGCTTTGCCA
TCEB3_4	GCCAGTGACAGCGATGGCGG
TCEB3_5	AATTTGGATAGAAAGTCACT
TCERG1_1	AAAGGAGGACGTCCAAAGGG
TCERG1_2	GGTGGAAACATCATACCCGG
TCERG1_3	TCAGGCCCAGGCACAAGCTC
TCERG1_4	TGAAACACTAACTGTAGGCG
TCERG1_5	ATAGAACATTAGAATCAACC
TCF12_1	AAAGAGGAGGTACAACATCT
TCF12_2	AAAGTCAGAAAGGTGCCTCC
TCF12_3	GTTGGTGAAACTGAGTGTGG
TCF12_4	TCTCTGCTACAAGTTCCAGG
TCF12_5	AGAGGTGAAGGTGATCCAAC
TCF15_1	AGGGTCCAGGCTCATCTCCG
TCF15_2	AGGGTGGCCGTCGTGACCTG
TCF15_3	GCTGTCGCACCACCACCACG
TCF15_4	TGCCTCAGCAACCAGCGCAA

TCF15_5	ACGTGCTGTACCCGGACGTG
TCF19_1	AGGCCGACTTCCGATGCCCA
TCF19_2	GCTCGGCATGCAGTTCGGCG
TCF19_3	TGACCCTCCAGTCATCACCC
TCF19_4	TGAGGACTCCACTTACCCAG
TCF19_5	ACAAGTACGAGTCAAGCCTC
TCF20_1	ATGAGGAGAACTTCTCGGTG
TCF20_2	GAAATGTTCCCACTGCCAGG
TCF20_3	GCACAGAGCAGTCGGAGCGG
TCF20_4	GCAGACCCACGCCAATGCGG
TCF20_5	ATGGCAGAGTCATTAGATGG
TCF21_1	CCTCCAAGGTCAGGACGCGG
TCF21_2	GAAGGTCCTCCACATCGCTG
TCF21_3	GACTTCCAACGAGAGCACCG
TCF21_4	GCAGACGTGGCCCTTTATGG
TCF21_5	CGATGTGGAGGACCTTCAAG
TCF23_1	CAGGACAAGGCAGGACCCGT
TCF23_2	GAGGGCCACCAGCCATGCCA
TCF23_3	GCAAGTAGCGGAGTCCACGC
TCF23_4	GGAGAGCATCTGCCTCTCCA
TCF23_5	CCAGTCCTGAGAATGCCGCG
TCF25_1	AATGCAAGGCGCCGGGCCCG
TCF25_2	GAGAGGATTGAGGACAGCAC
TCF25_3	TACACACGTTGTCTCTGCCG
TCF25_4	TTGGTGCCCGGGCAATCCTG
TCF25_5	AGCTCTGCTCACACTCAGGG
TCF3_1	GAAGTGGGTGCCCTCGCTGA
TCF3_2	GCAGGCAAGAGCGGTGAGCG
TCF3_3	GGAGAATGAACCAGCCGCAG
TCF3_4	TGAACCTCCGAACTGCGCCC
TCF3_5	CACCAGCACGAGCGTATGGT
TCF4_1	ACAAAGGAATACCTTACCCA
TCF4_2	ACTGAAGATGGCAAACCTGG
TCF4_3	AGAGATTCTCACTTACCTGG
TCF4_4	GAAGTAGCTCAGGGTCCTGG
TCF4_5	CGATGGAAAGTGGACATCGG
TCF7_1	GCGGACATCAGCCAGAAGCA
TCF7_2	GGTCTCTTTGTACATGCCGC
TCF7_3	TCGCTCGTGAACGAGTCCGA
TCF7_4	TCTCCCTCCAGGCTCTCGGG
TCF7_5	AAGGCCAATCAGCCCCCCCA
TCF7L1_1	ATGTACTCCCTTCCTCCCGG
TCF7L1_2	CAGGTCCGGGATCATCAGGA
TCF7L1_3	CCTTCGGCGAAATAGTCCCG
TCF7L1_4	GAGCAGCAGCTCGGACTCGG
TCF7L1_5	AATACGGTGACAGCTCGGAT
TCF7L2_1	AAACTTTCGGAGCGAGGCGG
TCF7L2_2	AGCTAAATCCCTCTCTGCCG

TCF7L2_3	GGAGGCACTTACGGTTCGGG
TCF7L2_4	TGAGGGCTTGTCTACTCTGG
TCF7L2_5	ACATCATACGCTACACACGA
TCFL5_1	ACAAGGCCACAACTCTGCAG
TCFL5_2	GAGATTCAGAATGTGGGCGA
TCFL5_3	GAGCCTGGTGGAGATGACGG
TCFL5_4	TGGTGACCTGTCCTGCACAG
TCFL5_5	ACAAATGCTTGCTCTACTAG
TCP1_1	CAAATCCCAAACCTTACCCT
TCP1_2	CAGAGAATCAGATATCACCA
TCP1_3	GTTCGGTGACCGCAGCACTG
TCP1_4	TCTCCATTTGACTTCTCCCA
TCP1_5	AACTGGACCAAATTAGACAG
TDG_1	GGAATGGAAGCGGAGAACGC
TDG_2	TACAAAGGGCATCATTACCC
TDG_3	TGGATGATCACACTCTACCA
TDG_4	TTTGACCTACAGCTTGCCCA
TDG_5	CCGGCAGCAAAGATCTCTCC
TDRD1_1	GATTGTCGACATCTTGGAAG
TDRD1_2	GGAGAAGTAGTTGGCTCCAA
TDRD1_3	GGAGTCCAAATTCCTAATGA
TDRD1_4	GGATGTGGAGGTAAACAATA
TDRD1_5	ATTTATCAATAAGCTCCACC
TDRD10_1	AATGAACATGACCACAGCCC
TDRD10_2	AGGCCAGAAGACATACCAGC
TDRD10_3	AGGCTGCTGGATCATATCAG
TDRD10_4	GCGAGTCCCGCGCATAACGG
TDRD10_5	ACTCATCCATAGCGTCCGTG
TDRD12_1	AAACTGTCAACACTGGTAGG
TDRD12_2	ATTGGGCCTTCTTCCACCCA
TDRD12_3	CAGGCCAACAGCCACAGCAC
TDRD12_4	GACGAGTCAATGGTTAAGCA
TDRD12_5	ACACAATGTCAGTACTGTCT
TDRD3_1	AAGACCAAGACATTTGGAGG
TDRD3_2	TATCAAGCCCATTCAAACAG
TDRD3_3	TGACTACGGAAACTATGAAG
TDRD3_4	TGCCTGGTATCATTTCGAGG
TDRD3_5	GAAAGCTCTGAAGCACATAA
TDRD5_1	ACCGAAGCAAATAATGAGCA
TDRD5_2	ATTGCACAGATTGGACCTGG
TDRD5_3	CATCGGCGAGTACCTTACCG
TDRD5_4	CCATAGTGGACCGATACCCA
TDRD5_5	GCGAGTACCTTACCGAGGAA
TDRD6_1	ATCGGGATGCACGTCCACGA
TDRD6_2	ATTGGATAAGAGTCCACCTG
TDRD6_3	CAAAGGCCAAATATCAGCCA
TDRD6_4	GCTGACACCAGAAATAGCCA
TDRD6_5	AGAGTCCATACAAAGCACAA

TDRD7_1	AGAGGCAGGAGGCTTGACAT
TDRD7_2	TCAAAGACATTCCAGTGCAA
TDRD7_3	TCTCTCAAGAACCTCTACTA
TDRD7_4	TCTGAGAAGTGTGCCAGCAG
TDRD7_5	AAGTGCTAATACCTTTATGG
TDRD9_1	AATTTAGCCAAAGATGGCCT
TDRD9_2	CCAGTCGTTGATCTGCTCGA
TDRD9_3	GCAGTGTCCAACCAACCAGT
TDRD9_4	TAAAGAGCGTGCCTGGACCC
TDRD9_5	AAGGTCAATTCACCATCATG
TDRKH_1	GAAGACATGACAGAGCCAGG
TDRKH_2	GAGCGAGTGCTGCTTATCAG
TDRKH_3	GAGGCTGTGAAACTCATCAT
TDRKH_4	TTGCCTATATCCTATACCGC
TDRKH_5	CCAGCACTATGAGAATAGTG
TEAD1_1	GCAGCAGCGAGACCCAGACT
TEAD1_2	TAAGGCCCTGCAGCACATGG
TEAD1_3	TCCACGCCCGACCTTCCCAG
TEAD1_4	TTCCACGCCCGACCTTCCCA
TEAD1_5	ACATGGTGGATAGATAGCCA
TEAD2_1	CTCACTCTGGAACATTCCAG
TEAD2_2	GCCGCCCTGGACGATGGCAG
TEAD2_3	TGTCTGGAAAGCCTTGTCCT
TEAD2_4	TTTGGCCCGAAGGAAATCAA
TEAD2_5	ATCCAGTCCAAGTTGAAGGT
TEAD3_1	GAAGTGGAGAAGACGGCCTG
TEAD3_2	GGAGTATTCAGCCTTCATGG
TEAD3_3	GGGAACTTACTGCTGAGCGT
TEAD3_4	GGGCTGGACAACGATGCGGA
TEAD3_5	CTGGGACCCATGTACCCGCG
TEAD4_1	AGACCTCAACACCAACATCG
TEAD4_2	AGCGCCCACCCACCTGGCAG
TEAD4_3	AGTAGCATGGCCCTCGCCCG
TEAD4_4	TCAGCACCACATCTACAGGC
TEAD4_5	CCCATCGACAATGACGCAGA
TEF_1	AAGACGCAAGAAGAACAACG
TEF_2	ACTGGAGAGCACCAGGTCGG
TEF_3	GAAGCCGCCTGTGGACCCGC
TEF_4	TGATGGCGAATCTTTCCACC
TEF_5	AGATTCGCCATCATATGGGA
TEP1_1	GCTTCCTGACCTGAAGACCA
TEP1_2	GGATAAACTCAGGCTCCAGG
TEP1_3	TCTTGGCCCGGTGCTTCCGA
TEP1_4	TGGCACAGAAATATCGTCGC
TEP1_5	CACTTTCCGAGACATGCATG
TERF1_1	AAGCGAGCCATTTAACATGG
TERF1_2	CGAGCCATTTAACATGGCGG
TERF1_3	GCGGTAACTGAATCCTCAGA

TERF1_4	GGCGGAGGATGTTTCCTCAG
TERF1_5	ACTTCTCAAGATAAACCTAG
TERF2_1	ATTTACACTGACAGAAGCAG
TERF2_2	CAGAGTACTGAGCCCAGCGC
TERF2_3	GCGCTTCCTGGAGAGCCACC
TERF2_4	GGGCGTCGTGCGTGACCCAG
TERF2_5	AAAGTTCTGGATAACAGGAT
TERT_1	AGAGAGCGCACCCTCCAAAG
TERT_2	ATCGTCCAGGCCCAGCACAG
TERT_3	GAGTCTGGACGTCAGCAGGG
TERT_4	GCTGCGCAGCCACTACCGCG
TERT_5	CACACGCTAGTGGACCCCGA
TET1_1	ACAAAGTTCATGCAACACGG
TET1_2	GAGACATTGAATGATACCAG
TET1_3	GGAAGCCAAGATCAAATCTG
TET1_4	GGAAGGTCGTCCCTTCTCTG
TET1_5	CCACCCCATCAAGAGATCGG
TET2_1	ATTTGAGCTGTTCTCCAGGG
TET2_2	GATGGATTAGGACTCTGGGA
TET2_3	GGAAGGCCGTCCATTCTCAG
TET2_4	TTGCCAGAAGCAAGATCCCA
TET2_5	GATTCCGCTTGGTGAAAACG
TET3_1	AAGGAGGGAAAGAGCTCCCG
TET3_2	GAGGCTGGGAACAACAGCAG
TET3_3	GATCCGCAGGCACACGCTGG
TET3_4	TCGCAAGTTCCGCCTCGCAG
TET3_5	AATGCCCTGACTACCTCGAG
TEX10_1	ACAGGAGTAGCTAGTTGTGG
TEX10_2	ATGCAAATACAGCCAAACAG
TEX10_3	GAGAGATGGGCACTTACCAA
TEX10_4	GTAGTAAAGTGTTATCCCGT
TEX10_5	CCTTGGCAGATGGATCCAGT
TFAM_1	AAGATGCTTATAGGGCGGAG
TFAM_2	AGGCAAGCGGGCCTACCTGA
TFAM_3	CAAGCGGGCCTACCTGAAGG
TFAM_4	TTAGAAGAATTGCCCAGCGT
TFAM_5	AAGAATTGCCCAGCGTTGGA
TFAP2A_1	AACAAGGACAACCTCTTCGG
TFAP2A_2	ATAGGATTAAATCTGCCTGC
TFAP2A_3	GATCTGGGATGTTAATACCC
TFAP2A_4	TCTTACCGGGACCTCCTCGA
TFAP2A_5	ATCCTCGCAGGGACTACAGG
TFAP2B_1	AATCGGTTTGAATTTACCCG
TFAP2B_2	AATGCAGCAGCACGTCCGGC
TFAP2B_3	ACCTTCTGAGGACTCCGCCG
TFAP2B_4	GTTGAAGATGCCAATAACAG
TFAP2B_5	ATCGGTTTGAATTTACCCGC
TFAP2C_1	GATCAGACAGTCATTCGCAA

TFAP2C_2	GCTGTAGAGGTGCTGCCCGG
TFAP2C_3	TAGGAAGAACATGCTATTGG
TFAP2C_4	TGAGGAGCGACAATCTTCCA
TFAP2C_5	ATATTCGGCGACTCCAGTGT
TFAP2D_1	ACTCGTAATGGAAGGACTGG
TFAP2D_2	ATTGTGCAGGTTAATAAAGT
TFAP2D_3	CAAGGTGGAGTGATAAGAAG
TFAP2D_4	CTTGGCTTAAACTTACCAGC
TFAP2D_5	CTCCCAAGAGTGAAGCATTG
TFAP2E_1	ACGGCGCGGGTCAAGGCCCA
TFAP2E_2	CTTGGCCAAAGACAGCCTGG
TFAP2E_3	GGTGCACACCTACTCCGCCA
TFAP2E_4	TCACATTGGCGGCCTTGCGA
TFAP2E_5	CGGCATCACAAATCCTGGTG
TFAP4_1	AGACGGAGAGAAGCTCAGCA
TFAP4_2	AGCCGAGTACATCTTCTCCC
TFAP4_3	CAAAGAGGGCACCTTCTGAG
TFAP4_4	CTGGGAGGACGAGAAGGCGG
TFAP4_5	ACAGCTCAAGCGCTTCATCC
TFCP2_1	CACCTCAGGAAGCTCAGCAG
TFCP2_2	GATGATGTGATCCAAATCTG
TFCP2_3	TGAGACAACCATACTCACAG
TFCP2_4	TGTGGGTATAATCGATCCTA
TFCP2_5	AAATACAGTGGAGTTCCTGT
TFCP2L1_1	AATGCAGAAGCTCTCTTCGC
TFCP2L1_2	ACAACTCCGGCAGCTACCTG
TFCP2L1_3	CAGGCGGGCCTCGTTCTCGG
TFCP2L1_4	GCTGGCGGTGGAGTCGGCCA
TFCP2L1_5	GGGGACCGGATCCTGGACAT
TFDP1_1	ATAGACCAGAACCTTAGTCC
TFDP1_2	CCTTGCAGTGACCTGACCAA
TFDP1_3	GATGGGCATGGCTTGCGGGC
TFDP1_4	GGCAGCGTCAAACACCCTGG
TFDP1_5	ACCGGCAGCGTCAAACACCC
TFDP2_1	ACTTGCAACACTCTTACCCA
TFDP2_2	ATGGTAGAGTTCAGAGCCGG
TFDP2_3	GTAGGAGAAGTTCTTGCAGC
TFDP2_4	GTCAGGCAAATGCTCTCTGG
TFDP2_5	GCAAATCGCTTTCAAAAACC
TFDP3_1	AGCAGCATCAAACATCCCTG
TFDP3_2	GCTGAGAAACCAGTATGTGG
TFDP3_3	TACGTGACAGAAATGGCTCA
TFDP3_4	TCCGGTGGATCTCAGTACAG
TFDP3_5	CTGACTCGTTTGGTGAGGCG
TFE3_1	AGAAGATGACGACATGGCAG
TFE3_2	ATGCTCAGCTATCTGCCCGG
TFE3_3	GTCATACATACCTTGAGCGA
TFE3_4	TGGCGTAGAGGCCAGCGCGG

TFE3_5	AGTACCTGTCCACCACACTC
TFEB_1	AGTCGCCACCACCTGTGCCT
TFEB_2	ATTGGGAGCACTGTTGCCAG
TFEB_3	GCTGGTGACGCCCACCAGGG
TFEB_4	GGACGACTCACTGCTACCGC
TFEB_5	CCTCCGGATGTAATCCACAG
TFEC_1	AACCGTGCCAAGTGAAGCCA
TFEC_2	ATAGCTTGATCACAGAGCTC
TFEC_3	CATTCTACAGTGATATGCGC
TFEC_4	GAAATTCAGGCTCGTACTCA
TFEC_5	AAGAAGATGACAATGCACAA
TFPT_1	AGGTGCAGAGGATAACTCGG
TFPT_2	GAGAGTGCTGGACTCCTACG
TFPT_3	GGAAGAGGCGGCGAGTGCCA
TFPT_4	TGAGAACTCCTCAAAGCCCA
TFPT_5	CCACCCCATAGGTGAACGAG
TGFB1I1_1	ACGACGCCAAACTCACAGGC
TGFB1I1_2	ACTCTCACCCTCATCTCCGA
TGFB1I1_3	GAAGCTGCTGCCTCCCAGGG
TGFB1I1_4	TTTGTGGAGCTCACCACCGG
TGFB1I1_5	AGCGCTCAAAGTAGCACTCG
TGIF1_1	GATTTCTGAAACGAGCTCTG
TGIF1_2	GCGGATGAAGAAAGGTCCAA
TGIF1_3	GGACAGCATGGACATTCCCT
TGIF1_4	TAGGAGACAGTGGCCTCCCT
TGIF1_5	ACGAGCCAAAACTGATCCCG
TGIF2_1	ACAGGGAGAGCACATTGGTG
TGIF2_2	GGTGGAGGTGGCGCTACAGA
TGIF2_3	TGTCTGGGAGAAGCCGCCGC
TGIF2_4	TTTACCATTTCCCGCCGCGG
TGIF2_5	ACAGGTTGGTCTGTCCAGAA
TGIF2LX_1	CCGGCCAAGTCAGGGCCCAG
TGIF2LX_2	AGAGCACAAGAAGAAGCGCA
TGIF2LX_3	CGTAGAAACGACCCCATCAT
TGIF2LX_4	CTTTGTTGCAGATTTCTAAC
TGIF2LX_5	GATGCTTATACATCCAGTCG
TGIF2LY_1	AGACCAATTTGTCTTTGTTG
TGIF2LY_2	GCGACTGGATGTATAAGCAT
TGIF2LY_3	GCGGAGGATCTTAACGGACT
TGIF2LY_4	GTTATTTCTCGACATGATTG
TGIF2LY_5	GCTTATACATCCAGTCGCGG
THAP1_1	CTGTGACCACAACTATACTG
THAP1_2	GAAACAGGAGGCGGTAAAGG
THAP1_3	GCGCGCAGGGTCCTCACTTG
THAP1_4	GTAGCGGTTCTTGCAGCCGT
THAP1_5	CTGCAAGAACCGCTACGACA
THAP10_1	ATACCCACACTACGGTGACG
THAP10_2	GTCGCTGTTCCGCTTTCCCA

THAP10_3	GTGTACAGGAGACTGGACCT
THAP10_4	TGTACAGGAGACTGGACCTG
THAP10_5	CCCGCCCCGGCACCTAAGAG
THAP11_1	AAAGTAGCGCGCAGACCCGC
THAP11_2	AAGGGTGAGAAGCACGGCCG
THAP11_3	CTTGTCGTCAGGCACCACGG
THAP11_4	GAAGGATCGGCTGCTTGCCA
THAP11_5	AGTAGCGCGCAGACCCGCTG
THAP2_1	AACGCAGAGCAACTCGAAGA
THAP2_2	AATGCCGACCAATTGCGCTG
THAP2_3	AATGTGCTTGTTGTAGGTAG
THAP2_4	GCCGACCAATTGCGCTGCGG
THAP2_5	ACATTAACATCAGCTTCCAC
THAP3_1	ACGGAGGCCGCAAGCAACAG
THAP3_2	GCAACCGCTACAGCAGCCGC
THAP3_3	GGAAGGTGAGCTGCTTCCTG
THAP3_4	TGGGTGCTGAACATCGGCCG
THAP3_5	AAGGAATGGGTGCTGAACAT
THAP4_1	CAAGCCCGACACCAACAAGG
THAP4_2	GAAGATGGATGGCACGGCCG
THAP4_3	GGAGGGCGAGGTGAACGGGC
THAP4_4	GGAGGTTCACATCTCCCACG
THAP4_5	ACTCAAAACGTCTAATCCAA
THAP5_1	ATTTGTTGTAAGAACCGCCG
THAP5_2	CATGGAAATAAAGTCAGCAC
THAP5_3	GATTTGTTGTAAGAACCGCC
THAP5_4	TGGGAAACCAGAATCTACCT
THAP5_5	AACCAAGATACAGGTAGAGG
THAP6_1	AAGATAGAAGGTATGACTCC
THAP6_2	ATAGACTGGACACTTTCTGT
THAP6_3	ATGAGGAAGCACCAACAAGA
THAP6_4	GCAGCGAGAAGCACATCCAA
THAP6_5	CCAAATATTAAACTGAAACC
THAP7_1	AAGAAGGACAACCCGAGGCG
THAP7_2	GAATGAACACAGCTACCAGG
THAP7_3	GATATCACAGGCTAAAGGAG
THAP7_4	GTCCCGTGTGCAGCAGCCGG
THAP7_5	GGAGCAGAAGTAGATGTACT
THAP8_1	AGGGCACTGCATCAGGCCGC
THAP8_2	GCCGAACTGCTCCAACACTG
THAP8_3	TGGAGGGCCTGACATAGCCA
THAP8_4	TTGTCTTCCAGAGTCAGCGG
THAP8_5	ACTTCAGGTTGTGACCGCTC
THAP9_1	ACCCGACACAGACTTTCTGA
THAP9_2	ACGGGTGCTGCAGCCCACTG
THAP9_3	GACCCGAAGTTGCTCCGCAG
THAP9_4	TCTTGGCTTCAATTAACACG
THAP9_5	CCAGTGCTACTAACTCCACG

THOC1_1	AATGAGCAGGAAAGCACCCT
THOC1_2	ATTGTGTCACACTGATCCAA
THOC1_3	TCGGGCAAACTGAAGAGCGG
THOC1_4	TGGCTGAAGGTACTTAACAA
THOC1_5	ACGAGGAAGCTCCAACAACG
THOC2_1	CGTGAGAAAGGTGAGGACCA
THOC2_2	GAATACATCAGCAAGAATGG
THOC2_3	GATATCGTCTGTATGGCCAG
THOC2_4	TGCGGGAACCACCACAGCCG
THOC2_5	AATGACAATGGAGCAACTAG
THOC3_1	CTAACTCATCCACATCCCAG
THOC3_2	GATGGGCAGACCATTGCTGT
THOC3_3	GTCACCTTTAGTGTTCACAG
THOC3_4	GTTCCTGGCGCACAGCGCCA
THOC3_5	ACCACGGGGCCATCGAGCCG
THRA_1	GGAGTGTGGGTCAGACCCAG
THRA_2	GTTTACCAAGATCATCACCC
THRA_3	TGGCGTCGGAGACTACGCCC
THRA_4	TTGAGCAGAACCGGGAGCGG
THRA_5	GATCTTGGTAAACTCGCTGA
THRAP3_1	AAGGAGAGAGATCTTAAACG
THRAP3_2	AAGGAGCAGACATTCTCTGG
THRAP3_3	GAAGATGGCTGACTTCCACA
THRAP3_4	GCTTTGACAAATCAAGACTG
THRAP3_5	AGATCCAAAAAGGAAGATCG
THRB_1	ACAGCGGTAGTGATACCCGG
THRB_2	ATAAGGCTGATTCACTGCCC
THRB_3	CAAGCCAGAGCCCACAGACG
THRB_4	CATAGCGCACAGCAGCGCGA
THRB_5	AGAACCGGGAGAAAAGACGG
TIGD2_1	AACAGTTCAGATCCTACCAG
TIGD2_2	AAGTGCTGAGGACCAGACCA
TIGD2_3	ACTCGATTTAAGCAGCGCCA
TIGD2_4	GCAGCGCCATGGTATTCCAA
TIGD2_5	AAAAAGCCCCGAGCATTCAA
TIMELESS_1	GATTGAACGGATCCTACTGC
TIMELESS_2	GCTCATACAAGGTTTCACTG
TIMELESS_3	TCTGGCCAGCTCGTCTGCTG
TIMELESS_4	TTATTTGCAGGCCTACAAAG
TIMELESS_5	AGGGACGCTTAGCTCAGGAG
TIPARP_1	GATGAAGTTGCTTCAAGAGG
TIPARP_2	GATTGAAGAAGCCAACTCTC
TIPARP_3	GATTGGGAACTGGAACCCTG
TIPARP_4	TGGATGGAGAGAGTATCCCG
TIPARP_5	AAAGGACAACTACTCAAAAG
TLE1_1	AAGCGATGACAACTTAGTTG
TLE1_2	CAAAGAAGTGGAACTTGCCG
TLE1_3	CACGATGCAGAGCACCACAG

TLE1_4	GCATCAACAACAGGTGGCCC
TLE1_5	AGACCCCCTCGTTAACCAAG
TLE2_1	GCTGGCGGCGGCTGTCAAGG
TLE2_2	GGAGCTGAACAGCCTCATCG
TLE2_3	TCACCGGGTGCCTTCCCTGG
TLE2_4	TCTGCTTCCCGCCACCACCA
TLE2_5	CAAGAGTGATTACAATCTGG
TLE4_1	AAGCCCGGCACTGCTACCGA
TLE4_2	GCAACAAGTGGTGCAGGCTG
TLE4_3	GCACCTCGGAAACTGGCTGA
TLE4_4	TATAGACAGAGACTCCATCA
TLE4_5	AATAGAGGCTGGACTAATCG
TLK1_1	AAACAATCGGAATCATCCAG
TLK1_2	GACTTGAGCCATTTCCACCC
TLK1_3	GCTTTGGTTGTACAATAGGG
TLK1_4	GTAGCAGTGGAAGTTTGGAG
TLK1_5	AGGCTAACTGTGATCTCAGA
TLK2_1	AAAGCGGAAACCTCCTGCCA
TLK2_2	GAGCCTCATTTACTGAACAG
TLK2_3	TAAAGTTGATGTGTGGTCGG
TLK2_4	TCAGGGAAAGGCACTCCTAG
TLK2_5	AGAGCTGGAGAGACTAGAAA
TLX1_1	ACGCCTGCAGATCTGCGAGC
TLX1_2	GAATGGGCAGGCTCACTCGC
TLX1_3	GGAGGCGCCTACACTTACGG
TLX1_4	TATGGTACTGGAGGTCCCGG
TLX1_5	ACGTTGTAGGAGCCGGTCAG
TLX2_1	CAGCGGCCGCCGCTTTGCCA
TLX2_2	GCCGGTCCTTGGCAAAGCGG
TLX2_3	TATGCGGCGCGTCCCAGAGA
TLX2_4	TGCAGGCAGAGAGGGTCCGG
TLX2_5	AAAACCGGACCCCTCCGAAG
TLX3_1	CAGCTCGCAGATCTGCACCC
TLX3_2	GCGCTGGTGGGCTCACACCA
TLX3_3	GCGGGTAGCGCGCTTGGCAG
TLX3_4	GCTGGAGACCGTGGGCACGG
TLX3_5	ACCCGGGAAAAGGACGTGCG
TMF1_1	AGAAAGATGAGCAGATCCGA
TMF1_2	GATGGGATACTTCAACCTGG
TMF1_3	TACGCTGCAATGCAAGCCTA
TMF1_4	TGGGCCGAGACCATTCCGTA
TMF1_5	AACATTAGTTATACCCACTG
TMPO_1	GAAGAAGAAATTGTTGGCAG
TMPO_2	GAGCCGAGCAGCCGTCGGCA
TMPO_3	GAGGGTCAAGAAGAACTCCA
TMPO_4	GTTGCCGCAGACCAATCAAA
TMPO_5	AGTTCGGTACTGCCCAAAGG
TNKS_1	ACAGACTCACAACCTCAGCA

TNKS_2	TGAAGGATCTGCCAGGTCCA
TNKS_3	TTAGCAGCACAGCACCACTG
TNKS_4	TTGGAAGCAGCCTCGTGCAG
TNKS_5	CCTACATTAGTCAACTGCCA
TNKS2_1	AATGTACAGCAACTCCTCCA
TNKS2_2	AGTGGTGAAACATGAAGCAA
TNKS2_3	GGTGAACAGCCGCGACACGG
TNKS2_4	GTATAACAGAGTGTCCGTGG
TNKS2_5	CACACCCATAGCTCAGGAGT
TNP1_1	AAGAGCCGATCTCCTCACAA
TNP1_2	CAAGAGCCGATCTCCTCACA
TNP1_3	GTGGGAGCGGTAATTGCGAT
TNP1_4	TAAAGAGTCATGGCATGAGG
TNP1_5	AGCCGCAAATTAAAGAGTCA
TNP2_1	AATGGCCAAGAGGATCCAGC
TNP2_2	TCTGGCTGAAGGTTTGGCAA
TNP2_3	TCTGGTGGCCGGATGAGCTG
TNP2_4	TGGGCCGCATGGGAGAGTGG
TNP2_5	GGGCTCCAGTTGGGTTGCGG
TNRC18_1	GAAGACGGTGTAGACGCCGG
TNRC18_2	GCAGTGGCACTGGGCTCCCA
TNRC18_3	GCCATGGACAGCCACCGCGT
TNRC18_4	GTAGGATGGGTACAGCCCAC
TNRC18_5	GCTCGGGCAACGCATCCATG
TNRC6A_1	AATAAGATGGACCTTTCTGG
TNRC6A_2	ATGGAAGTAGTACTGGGCCA
TNRC6A_3	GGTACTTCACGAGGATACCG
TNRC6A_4	TGGATAATGGTACTTCAGCA
TNRC6A_5	ACTTGAAAGTTAGTGCCCAT
TOE1_1	AAGGCCATTGTGTAGCACCA
TOE1_2	CCAGATAGGAATGTTCACCC
TOE1_3	TCAGCTCCCGCAGCTTCCGA
TOE1_4	TGGCAGCCCACACCTTACCC
TOE1_5	AGGTCAATATCGTGAGACTG
TONSL_1	AAAGCCAAGGCGCAGAGGGC
TONSL_2	AATAGCCAAGCTCTTCTCAA
TONSL_3	CTTCCAGGGACACTGGCCCA
TONSL_4	GGCGCTCTCCGATCTTGCGG
TONSL_5	AGACATGAAGGACCACCATG
TOP2A_1	ATGCTGCGGACAACAAACAA
TOP2A_2	TATGGGAAGAGCTGGTGAGA
TOP2A_3	TGTACGCTTATCCTGACTGA
TOP2A_4	TTAAACACACTTCCCACCTG
TOP2A_5	AGCATTGTAAAGATGTATCG
TOP2B_1	ATGCTGCTGACAATAAACAG
TOP2B_2	GCAGCCACCCGACTTGGCCA
TOP2B_3	GTCAGTGGAGCCATTGACGC
TOP2B_4	TGTAGGAATGAATTGCAGGG

TOP2B_5	ATCAACGTGTAGAGCCTGAG
TOPBP1_1	AAGCATGGAGGTCAATACAT
TOPBP1_2	TACACTGTGTGACCACACAG
TOPBP1_3	TGTGAAACTGGAATTTCCAC
TOPBP1_4	TTTGGAATAAATCAGCCCAC
TOPBP1_5	AAATATGTACAAATGATGGG
TOPORS_1	AACAGTACTCCACTATCCGG
TOPORS_2	GGTACGAATCATCTTACAGG
TOPORS_3	GGTGTCGGCAGGATCCGCGA
TOPORS_4	TCTCCGCCTACCCTCCGAAG
TOPORS_5	CAGCTGAAATATCCCTGTAG
TOX_1	ACAGGGAGCGTCGGGCGCAG
TOX_2	GAAGAGGCGGGCTGATCTGG
TOX_3	GATGGCAGCCATGAGACCAA
TOX_4	GCACCAGCGAGTGGTCTGGG
TOX_5	ATCACGAAAGAATAACGCAT
TOX2_1	AGGTTGGGAGGTGTTATCGG
TOX2_2	ATTGCCGGGCGACCCAGCAG
TOX2_3	GATGGTGACAGTGCCTACGT
TOX2_4	GCGCACCTGGACTATTACCA
TOX2_5	CACCTGGACTATTACCACGG
TOX3_1	ACAGACTCAAGTATTATCGC
TOX3_2	GATGGACCGCATGATCAGGG
TOX3_3	GCACTGCGCGAAGTCCAGGC
TOX3_4	GGGAAGGCAATGGATCGCTG
TOX3_5	ACTGTTCCATGTTGAGACAG
TOX4_1	ATGGTGTTGAGGATTTCCGG
TOX4_2	CCAGACATTGGACATGCCTG
TOX4_3	TCCAAGACTATCCCACATGG
TOX4_4	TTTGGTTTCCAGTTTCCCGG
TOX4_5	CCATGGTCAAGCCCCCACTC
TP53_1	ACCAGCAGCTCCTACACCGG
TP53_2	AGTGGAAGGAAATTTGCGTG
TP53_3	GATCCACTCACAGTTTCCAT
TP53_4	GCAGTCACAGCACATGACGG
TP53_5	CCATTGTTCAATATCGTCCG
TP53BP1_1	AGTTCGGCTTACCTTGCAGG
TP53BP1_2	CAAGGCTACCTTCAGCACCA
TP53BP1_3	TCCAAGTTAGAAGAATCCAC
TP53BP1_4	TGTGCGTCTGGAGATTAGGA
TP53BP1_5	AGACCCATGATCCCATACTT
TP63_1	AAGGTACTGCATGAGTTCCA
TP63_2	GCGCGGATAACAGCTCCCTG
TP63_3	TATGTAGAAGATCCCATCAC
TP63_4	TGGAGAGAGAGCATCGAAGG
TP63_5	CAATGATTAAAATTGGACGG
TP73_1	ACACATGAAGTTGTACAGGA
TP73_2	AGAGATTATTGCCTTCCACG

TP73_3	ATGTAGTCATGCCCTCCAGG
TP73_4	GGACCAGATGAGCAGCCGCG
TP73_5	CATGCCTGTTTACAAGAAAG
TPRX1_1	CCGAACCCAGGCCTGATCCC
TPRX1_2	CTAGGCCTGGAAGTGAGCCA
TPRX1_3	GAGACTGAGGATCCCTCCAA
TPRX1_4	TTGGGCCTGGAATTGAGCCA
TPRX1_5	CCAATCTCAGGCCCGAACCC
TRAF7_1	AGATGGCGGAGTCGGAGCGG
TRAF7_2	GCTACAACCGCTTCTCCGGG
TRAF7_3	GTAGGCAAGGGTGCTGGAGG
TRAF7_4	TGACAGCACAGCTTCACCGA
TRAF7_5	AGCTGTCCCACATCAACGCG
TRDMT1_1	AATCAGTCATATCACCCTGC
TRDMT1_2	AGGTCCGTGTGCTTTACCAA
TRDMT1_3	GCTGGAGCTATACAGCGGCG
TRDMT1_4	TATAGCTCCAGCACCCGCAG
TRDMT1_5	ACTGTGATACCTACAGAGGT
TRERF1_1	GAGCGACACAGCAGCACCCG
TRERF1_2	TCGGGCAGCACCCTACCCAA
TRERF1_3	TGGGCGGGAGAAGCTTGACA
TRERF1_4	TTGAGCAGTGGCCAACCCAA
TRERF1_5	ATGGATACCTCGAGGCAGGG
TRIM13_1	AGAGCTTTGAGACCTGGCGT
TRIM13_2	GATGGCCTTTAACATTGCTG
TRIM13_3	TATTGAAGATGCCTATGCTC
TRIM13_4	TGAGAAGTTACAACACACAC
TRIM13_5	CAAGTGTCCTACATGCCGTA
TRIM16_1	CATCCAGGGAGACTATGGTG
TRIM16_2	CCAGTGGAAGAAGAGGACGT
TRIM16_3	TCTCCCTGGATGCAGCCCGC
TRIM16_4	TTCTGGGTCAGCCAGCCCAG
TRIM16_5	GAAGTCCTGTCTAACCTGCA
TRIM24_1	ATGCATCACACCTTGCACGA
TRIM24_2	CAGGACAATGGAGGTGGCGG
TRIM24_3	GAGGACAACGCAGAAGCCAA
TRIM24_4	TCTAAATGGGCAGTTTCCAG
TRIM24_5	CTGCTGCACTAGTAATCGTG
TRIM27_1	GAATCAGGATTCCTGAACCT
TRIM27_2	GCACTCGGCCACACTCCCGG
TRIM27_3	GTTGCAAGAGAACTGGGTGA
TRIM27_4	TATCAGAGAGGATCAGGCTG
TRIM27_5	CCAAGTCTAGCTCCTCAAGG
TRIM28_1	ATTGAGCTGGCAGTCTCGGC
TRIM28_2	CCAGCGGGTGAAGTACACCA
TRIM28_3	GAAGCACTGTTGCTTGCACA
TRIM28_4	GTGAATGGCGGCCTCCGCGG
TRIM28_5	CTTCCCAGGCAGTACCACTG

TRIM33_1	ACTATGGCAAATGCAAACCG
TRIM33_2	CAAAGAGGCAACATGAACTG
TRIM33_3	GCTCAGCGTGCCCATCCCGG
TRIM33_4	TGCTAAGTTAATCTGTCCAG
TRIM33_5	CGGGGGCGCAGTATCGACGC
TRIM37_1	AATGGATGAACAGAGCGTGG
TRIM37_2	AATGTACCAATGCTGGTCCC
TRIM37_3	GATGAAGTAAATCAGCTCGA
TRIM37_4	TCTGACATGCTTCTCGAAGG
TRIM37_5	CTCTAATTTAAATAGCATGG
TRIM5_1	CATAGTGGAGAAGCTCAGGG
TRIM5_2	GTTGATCATTGTGCACGCCA
TRIM5_3	TCACCACACGTTCCTCACAG
TRIM5_4	TCGGCATGTAGCCAACATAG
TRIM5_5	AAGAAGTCCATGCTAGACAA
TRIM66_1	GAGCTGAACAAACAGGCCAA
TRIM66_2	GGAAGGTGTGACTACACAGG
TRIM66_3	TCTCTGGCAGGAGTGAATGG
TRIM66_4	TGAGCAACCCAAGATGGCCA
TRIM66_5	AGAGGACAAGTTGCCAGACT
TRPC3_1	AAAGCAAGTACCCTGCTGCA
TRPC3_2	CGAGTACGGCAACATCCCAG
TRPC3_3	GTACTCACAGGTCCGGCCCG
TRPC3_4	TCTCTGCCGAGACTCAGAAG
TRPC3_5	CGACTTCTACGCTTACGACG
TRPC4_1	AATGAGAGAGGGACTGGCCA
TRPC4_2	AGGAGAGCAGTTCTTCCGAG
TRPC4_3	CTTCCCAGAGATATTTGCAG
TRPC4_4	TCGAGTGGATGATATTACCG
TRPC4_5	CACATGTCCCATGATTCTCG
TRPC5_1	GAAGATTGTGGCCTATGTCA
TRPC5_2	GAAGCCATCATACCACAGGG
TRPC5_3	GAGCTCCCAGCCCAGACGGA
TRPC5_4	TGAGACAGAGCTCTCTGCAG
TRPC5_5	AAAAACGGGTCACTATCCCA
TRPS1_1	AAACCAAGGGATTCCTGCAG
TRPS1_2	AACGTTGCAAGTGAAGGCGA
TRPS1_3	AAGAAAGCGCCTTAACCCAG
TRPS1_4	GCAGAAGCAAGAGTGCACAG
TRPS1_5	CACTACAGCCAACGGCGAAG
TRPV2_1	CATCAGGCACGTCTTACCTG
TRPV2_2	GAAGTCCAGCCAGATCCTCG
TRPV2_3	GTTGTCCGAGATCATCACTA
TRPV2_4	TGGGAGCGGGCTGCCTCCCA
TRPV2_5	ACATGTAGATCAGATTACAC
TRPV6_1	TATGGAGCAAGTTCTGCAGA
TRPV6_2	TGACAACCTGGAGGCCGCCA
TRPV6_3	TGCGAGCCCTGCTTGCCCGC

TRPV6_4	TGTGAGGTCATAGAGAGTCG
TRPV6_5	AGAGGAAGGAAACTCACATG
TRRAP_1	AGAGACTGCACACCTCAGAA
TRRAP_2	AGTGGTCTGGTCAACCACCG
TRRAP_3	GGAGAAATGTAAGGAATCGA
TRRAP_4	GTGGTGTCAAGACAATCACG
TRRAP_5	CAGCATTCCATCATTCCGAG
TSC22D1_1	ATCCCAATGGTGTAGACCAG
TSC22D1_2	ATGGTGTAGACCAGTGGCGA
TSC22D1_3	CTGCGATGCTGGCTGGGCGG
TSC22D1_4	GCAATTGCATGAAATCCCAA
TSC22D1_5	ACTCCTAGATCCATCGCCAC
TSC22D2_1	ACGGCGGCCACCCTTCCCGT
TSC22D2_2	AGTATTCATACCTATCCCAG
TSC22D2_3	CAGGTACTCACTCTGGCCGG
TSC22D2_4	GAGGAGACATTCGGCTGCTG
TSC22D2_5	AGCGGAGAGCCCTATAGACG
TSC22D3_1	GCTTGCCAGGGTCTTCAACA
TSC22D3_2	GTAGACCGCCACCTCCATGG
TSC22D3_3	GTTGTCTATGGCCACCACGC
TSC22D3_4	TTACACCGCAGAACCACCAG
TSC22D3_5	AATGTATCAGACCCCCATGG
TSC22D4_1	CTGGGAGAGCCTTATCGCCG
TSC22D4_2	GAGCTGCTACTGCTCCGAAG
TSC22D4_3	GATAAGGCTCTCCCAGGCCG
TSC22D4_4	GGGAAGGGAGGCTCAGACGG
TSC22D4_5	ACTCCTGGAGGGAATTCGAG
TSHZ1_1	AAAGCAGCCCGACTCTCCCG
TSHZ1_2	ACTCCGAGAAGACCAAGAGG
TSHZ1_3	GACCCACGGCAAGTCTCCCG
TSHZ1_4	GGAGCCCAAGTCTTCCTCGG
TSHZ1_5	CAGTGCCGCGTACGACACGC
TSHZ2_1	AAAGTGGAAAGTGATGCCAC
TSHZ2_2	ACAGCAGGCACCCAAGCGGG
TSHZ2_3	GAGCGAAGTAGAGGATGCCG
TSHZ2_4	TGTGTGGGCATAGAAACCAG
TSHZ2_5	AAAAACGCGTTTCTTAGCCG
TSHZ3_1	AAGATGGAGGCATCCAGCGA
TSHZ3_2	GAACGAAGAGGAGACCAAGG
TSHZ3_3	GCCGGAGAAGCATCATGCCG
TSHZ3_4	GGAGAAGCATCATGCCGAGG
TSHZ3_5	AGTGACATCCGCAATCAACA
TSPYL2_1	AGAGGCTGACAGGATTGCTG
TSPYL2_2	GAAGATAATGATGGCAACGA
TSPYL2_3	GAGGCTCCAGGAGGAAACGG
TSPYL2_4	GGATCTCAGACATATCTCCA
TSPYL2_5	GGAAAATGTCTTCATCACGT
TSSK6_1	AGCCGCCTACGCGTCACCCG

TSSK6_2	GGTCGCGCGCCTGAACTCCG
TSSK6_3	GTTGCGCGCTACCTGGCCCG
TSSK6_4	TGGAGAGGGCAGCTACTCCA
TSSK6_5	AACTTGTTGACGAAGTCCGG
TTF1_1	AAAGCCAGGACTCCTCCTGG
TTF1_2	GTAGGAACATTGCCCGGCCC
TTF1_3	TAAGGCTGACCTTGGCCCGC
TTF1_4	TCGTCTTCCAGTCATTCCCA
TTF1_5	AACAGTCTCAAATAACTAGG
TTF2_1	AGCTTCACGGCCCTCACCCA
TTF2_2	AGTCCTCATGCAATAAGCAA
TTF2_3	CTTAAGACCGGCGTCCGCGA
TTF2_4	GCTGGTGTGGAAGTATTCCA
TTF2_5	ACAGGTTGGGTACCACGACG
TTK_1	AAATGCTGGAAATTGCCCTG
TTK_2	GAGGACAGACTACTAAAGCC
TTK_3	GATGATGGCAAACAACCCAG
TTK_4	TTAACTGGGACTCTTCCAAA
TTK_5	AAAGACAGGTTGCTCAAAAG
TUB_1	CAAGGCGGCAGCTACAGCAG
TUB_2	GACAAGAAAGGGATGGACCG
TUB_3	GCTGAGGTAGGACTCCACCA
TUB_4	GGCACGGAGATCCTGCCCGC
TUB_5	AGTGGCAGCACCAGCTACCA
TULP1_1	AGAAGAAAGCCTGAGCCCGG
TULP1_2	AGAGGCCCACACCTCTCGGA
TULP1_3	GAAGAGCTCCGCAGACCTGA
TULP1_4	GCTACCAGAAAGGTTTCCCG
TULP1_5	CAGGCACTCCCAAAGGCGCG
TULP2_1	AGACAAGAGCGCCAAAGCCA
TULP2_2	TATGGGACCAAACCCTGGAA
TULP2_3	TCCGGAGGAAAGGGTTCCCA
TULP2_4	TCTCCGAGAATCCGACGCAA
TULP2_5	CAGGATTGGCCTGAACCATG
TULP3_1	ACAACAGAGGAGCTTACTGA
TULP3_2	CATGTAAGATGACATTGCTG
TULP3_3	GGGAGACATAGACGACCTGG
TULP3_4	TGAAAGTGTGAACTTCGATG
TULP3_5	AAGGTGTCACAGTAAGATGT
TULP4_1	GAACTACCCGATCTTCCTGG
TULP4_2	GGGACAGGAGTACTCCACAG
TULP4_3	GTGAAGCTGACGGTGAGCAG
TULP4_4	GTGGAGCTGGTCAACGACCG
TULP4_5	AACATTGTAGAACTTGACCA
TWIST1_1	AAGTCTGCGGGCTGTGGCGG
TWIST1_2	CAAGCGCGGCAAGAAGTCTG
TWIST1_3	CGACAGCCTGAGCAACAGCG
TWIST1_4	GATTCAGACCCTCAAGCTGG

TWIST1_5	AGCGGGTCATGGCCAACGTG
TWIST2_1	ACACGGAGAAGGCGTAGCTG
TWIST2_2	CGAGCTGGAGCCCTCCTCCA
TWIST2_3	CTCCTCGAAGGACTGCGCGC
TWIST2_4	GATCTTGCGCAGCGCCGCGA
TWIST2_5	GCGCACCCAGTCGCTCAACG
TXK_1	AAGCCCATCTTACTCACAGG
TXK_2	AGAAATACAATCCTCACTGG
TXK_3	GGTGATACCAGATTAACTCA
TXK_4	TTTGACAATTGGCTGAGCCA
TXK_5	ACCGTAGCTAAACCCAGCTG
TYW5_1	AACACTGGCCGAGTTACCAG
TYW5_2	ACTTGGAGAAGACCCTAGAA
TYW5_3	ATGTACAAGCAAATGGACAG
TYW5_4	CATGCAGCACCTCTACCCAC
TYW5_5	AAATACTACTTACGGTCACT
UBA1_1	ACTAAGGAAGTCCTCAACGA
UBA1_2	AGTGAAGCAGACATAGACGA
UBA1_3	ATGACCAGGGCACTGCCCAG
UBA1_4	GGACAACACGGACTGGGCAG
UBA1_5	CACTAAGGAAGTCCTCAACG
UBA2_1	CCGCCATGGCACTGTCGCGG
UBA2_2	GCAGGATGACCCATCTGCAA
UBA2_3	TAAGAGGAACATCAGCTGCC
UBA2_4	TGGGCTGAAGTACAAAGTCA
UBA2_5	ACAGCCAGGAAAGGTTCTCT
UBA7_1	AAGTCACCAAGTCTCCATCA
UBA7_2	AGCCAGGTCGGACCAGCAGG
UBA7_3	CTTGGAAAGGAGCAGAGCCG
UBA7_4	TTTCTGGCGGCTGACACCCG
UBA7_5	CCCCGCACGTGGATAGACCG
UBE2A_1	AGTGATACTTGCCATTTGGA
UBE2A_2	CGCTCGCGACATGTCCACCC
UBE2A_3	TCCGTCCGAGAACAACATAA
UBE2A_4	TCTCTTACCATCCTCAAACG
UBE2A_5	CAACATAATGGTGTGGAACG
UBE2B_1	CATGAGCCTCCTCCGGGCCG
UBE2B_2	GCTGCGGAGCATGTCGACCC
UBE2B_3	GTTACAAGAGGACCCACCTG
UBE2B_4	TAACAGTCCAGCCAATAGCC
UBE2B_5	CTGAAAACAACATCATGCAG
UBE2C_1	CATATGCCTGGACATCCTGA
UBE2C_2	CTAGCGAGAGCTTATACCTC
UBE2C_3	GGGCGTGAGGAACTTCACTG
UBE2C_4	TCCATCCAGAGCCTTCTAGG
UBE2C_5	CGGCTCAGCTCCTTTACGGG
UBE2D1_1	CCATAATAGTGGCTTGCCAG
UBE2D1_2	CCTGATAGCGCATATCAAGG

UBE2D1_3	CTGAATCCTCTTCAGCGCCA
UBE2D1_4	GGTCCAGCTGAACAGTGAGC
UBE2D1_5	CAGCCTGATAGCGCATATCA
UBE2D3_1	AAGAATACACCGCCTTGATA
UBE2D3_2	ACAATAGGTTAGCAAGAGAG
UBE2D3_3	AATGACAGCCCATATCAAGG
UBE2D3_4	ACTTACAGGTCCCATAATTG
UBE2D3_5	GGGAAAATACTTGCCTTAGG
UBE2E1_1	AGCACAGTACCTCTTGGCGC
UBE2E1_2	GAAGTGAGCAGATAGAAAGG
UBE2E1_3	GTGGCAATACTTCCCACCAA
UBE2E1_4	TATGTCGGATGACGATTCGA
UBE2E1_5	ACAGTACCTCTTGGCGCTGG
UBE2E3_1	ACTAGTGGATAACTTAGCAG
UBE2E3_2	AGATGTCCAGTGATAGGCAA
UBE2E3_3	CCTTCATATACAGAACCCGG
UBE2E3_4	GTGGCTATGCTTCCAACCAG
UBE2E3_5	ATTTCTTACCTGCAATTAGG
UBE2H_1	ATTTCAGCACCATATGAAGG
UBE2H_2	CATCCAGAAATACGCCACGG
UBE2H_3	GAGGCGGATGGACACGGACG
UBE2H_4	GTACATCCAGAAATACGCCA
UBE2H_5	ACGCTTCATCAATGTTGGGA
UBE2I_1	CCAAGACCCAGCTCAAGCAG
UBE2I_2	GTGCCTGTCCATCTTAGAGG
UBE2I_3	TAGAGGAGGACAAGGACTGG
UBE2I_4	TCTCCTGGGCGAGTCTGCTG
UBE2I_5	AAGCCCTCCTTACAAATGGG
UBE2M_1	AGGTCCTGCAGAACAACCGG
UBE2M_2	CATTGTCTCACACTTCACCT
UBE2M_3	GAGTCGGCGGGCGGCACCAA
UBE2M_4	TCACCAAGAAGAGATACTGC
UBE2M_5	GCGCAGCTGCGGATCCAGAA
UBE2N_1	AGATACCTGTTTCTATGGCT
UBE2N_2	TTCTGTCTACAGCTAGAGCA
UBE2N_3	CCTCAAAGGGGGAATCCTGA
UBE2N_4	CCTTCCAGAAGAATACCCAA
UBE2N_5	CTGTTGCCTTCATAGATAAG
UBE2Q2_1	AAGGAAGAAGAGCCTATTAG
UBE2Q2_2	AGGAAGAAGAGCCTATTAGT
UBE2Q2_3	GGACGCCAGGAACTTCAGCT
UBE2Q2_4	TTAGCCATCTTCCTTTGGAG
UBE2Q2_5	CCAGCTTCCAACTGACGATG
UBE2T_1	ACAGTTGCGATGTTGAGGGA
UBE2T_2	GATGCTTGATAATCTACCAG
UBE2T_3	GGAGTGAGAAATCGGATCTG
UBE2T_4	TTGCCAACATGTGATGCCTG
UBE2T_5	CAGCCATGAGCGGGTCATCA

UBE3A_1	CTACTACCACCAGTTAACTG
UBE3A_2	CTAGCTAGAGATGATCGCTA
UBE3A_3	TAGCTTCCTGTAGACAACCA
UBE3A_4	TATCCACAGACACATCATCA
UBE3A_5	AAACAAGAAAGGTCCTCGAG
UBE4A_1	AAACTGTTTGGCATCAGCCA
UBE4A_2	GAAGTAATAAGCGAGCGAAG
UBE4A_3	GGATGAATTCGATTACTCTG
UBE4A_4	TTGGTCAACATAGATCTCTG
UBE4A_5	GAGCTGTAGCCATCTGGCAA
UBN1_1	AGCTGGTGAAGAATATCCGA
UBN1_2	CAGACAGAAGTTCTTCACCC
UBN1_3	GAGCAGTCAGGTCCGCTCTG
UBN1_4	GATGATGGAAGTGATTCCCT
UBN1_5	AAAAGATGACACTTATGACA
UBP1_1	AAATCAAGAAGTCTGTCTCC
UBP1_2	CCAAACCTGAATGAAAGCAG
UBP1_3	GCGAGGCCGGAAGATGGCCT
UBP1_4	TCAGCACAGAATTTACTCCA
UBP1_5	ACGCTTACTTATTTGAACCA
UBR1_1	CCAAATAAGTACCATTCCAG
UBR1_2	GGTTGGGCAACACATTGAAG
UBR1_3	TCTTGACTGTGAGCTCGCAG
UBR1_4	TGAGAGGATGGAAATCAGCG
UBR1_5	AAGACAGTAGTACAATCGTG
UBR2_1	ATGCAGGATCTTCACCACAA
UBR2_2	TCCAAGATGACAACATCAGG
UBR2_3	TTCGGCCGAGGAGATTGCGG
UBR2_4	TTGAGACATCGAGATGCCCA
UBR2_5	AGAGGGTGTTCTATCAACAT
UBR3_1	AAACTAAGCAGTGGCACCAA
UBR3_2	ATGAGACTGGACATACGTCA
UBR3_3	CCTGTGCACAGCCAATGTGG
UBR3_4	CTACGACGAGTTCTGCGCGG
UBR3_5	AAAATGGCTGTAGTTAACTG
UBR4_1	CATACAGACAACTTAGGCTG
UBR4_2	GAGTAGGACGCGGACAGCAG
UBR4_3	GGAACCGATTGATATAGCGT
UBR4_4	GTGAAGACACTTTCAGACGG
UBR4_5	GAAGATCTCGATATATGACG
UBR5_1	AAAGTTGAACAGCAACTCGG
UBR5_2	GAATAACTCGAGTCTGCCGG
UBR5_3	GCAGCGGGTGAACCACGAAA
UBR5_4	GTTCCAATACATTCAAAGGG
UBR5_5	CAGACTCGAGTTATTCGGAC
UBR7_1	AGTCTTCACAGACTACGCAC
UBR7_2	CCAGAGGGAGAAGAACCAGC
UBR7_3	GGGAAAGGATGATGTCCGGG

UBR7_4	GTTGAGGATGGCCGGAGCCG
UBR7_5	AAAAGAACAACGTTTCATGC
UBTF_1	AAGGAGGTGAAGGACTCCCT
UBTF_2	CATGTGCAAGCTCAAATGGG
UBTF_3	GAGATACACCTTCTTCTCGT
UBTF_4	GCTTCCGGAGAAGAAGAAGG
UBTF_5	CCAGAAAAAGAAAGATTACG
UCHL5_1	AAGTACACAACAGTTTCGCC
UCHL5_2	AATGAGCTCGGTGAAGACCC
UCHL5_3	ACAGAACGCAAAGAAAGCTC
UCHL5_4	GTTACAAAGACAACTTGCAG
UCHL5_5	ATGATTGGATCAGTGCAGTA
UGP2_1	AAACTTACCGAATCTTCAGG
UGP2_2	AAAGGACGTGTCTTACTCAG
UGP2_3	GTGGTGGTGAAACTCAATGG
UGP2_4	TGAAGGCAAACTGAGACTGG
UGP2_5	AAGAATATACAGATCCACTG
UHRF1_1	ACACCCGACTCGCTGACCTG
UHRF1_2	ACGAAGTCTTCAAGATTGAG
UHRF1_3	CTACAACCCCGACAACCCCA
UHRF1_4	TGCTCGGGACACGAACATGG
UHRF1_5	GCGGGAACTCTACGCCAACG
UHRF2_1	GATGCCAGAGATGTCGGCCT
UHRF2_2	TGATTGGAAGGTCCTACCCT
UHRF2_3	TGTATGTGAACTCATCACCT
UHRF2_4	TTTGCGAGACACGTCCTCAA
UHRF2_5	AATCTTCTCTGACAGGACCG
UIMC1_1	AGACCCTAACCAGTATACCA
UIMC1_2	GAAGAAATCACTGTTTGTCC
UIMC1_3	GAAGACGGTCCAGTGGCCAG
UIMC1_4	TAGGGAGGTGAACAGCCAGG
UIMC1_5	AAAGGCTCACATATCAGTCA
UNCX_1	AGAGCCCGGGCTGCAAGCGG
UNCX_2	GAAGCGGCTTTCCGGCGCGG
UNCX_3	GATGGAGAAGGGCACCGAGG
UNCX_4	GTCGATGGAGAAGGGCACCG
UNCX_5	ACACGAAAAAGGGCCCGGGG
UNG_1	AAGCAACTGTACCTGGGCGG
UNG_2	GAAGGGCAGTGCCATTGATA
UNG_3	GATCCAGAGGAACAAGGCCG
UNG_4	TGAGGAAAGCGGAGATGCGG
UNG_5	ATGGACCTAATCAAGCTCAC
USF1_1	AAACAGCTGAAACGGAAGAG
USF1_2	GAGGGAACTGGCGCCATCAG
USF1_3	GATGATGCAGTTGACACGGA
USF1_4	TCGGAAGACGTACTTGACGT
USF1_5	ATGATGCAGTTGACACGGAG
USF2_1	AGGCAGCGGGATCCAGACCC

USF2_2	CGACAAGGGACCCGAGGCGG
USF2_3	GAACGCCGCCTGCTGGACGC
USF2_4	GTCCACCGCTGCCTTCGCGG
USF2_5	AGCCGTAGTATCTCCCACAC
USP11_1	AGAACGGACGGCGATGGCGA
USP11_2	AGAGCTGCCCAACATCCAGA
USP11_3	GATAAACTGGCGCCTCAAGG
USP11_4	TGGGAGGCATACGTGCAGGG
USP11_5	GGTCTCCATGATGATCAACT
USP12_1	AGTGAACCACAACAGCAACA
USP12_2	GCCACCTACCATGGTACAGA
USP12_3	TTGCGTATAAGAGTCAACCT
USP12_4	TTTATAGGGCGCCAATGCTT
USP12_5	CAGCACACCAGACCCAACGT
USP13_1	GAAAGCAGGACCCAGACACG
USP13_2	TACGGGAGAGTCGTAGGAGA
USP13_3	TATTGAGGAGTTACCAGCCC
USP13_4	TCTGGCACACTTCCAACCAC
USP13_5	CCTGAAAAGACATGTGCGAG
USP15_1	GAAGATGGAACATGGCCAAG
USP15_2	GATAACTCTGGACTTCTCAA
USP15_3	GATGGCGGAAGGCGGAGCGG
USP15_4	GTTGGAATAAACTTGTCAGC
USP15_5	AAGGTGTTCCTTAAGTGACT
USP16_1	CATGGGAAAGAAACGGACAA
USP16_2	GCTAAAGTAAGAGGGCCTGG
USP16_3	TGGCAAGGAGAATTCATGGG
USP16_4	TTAAGACACAGCCAAACTGA
USP16_5	AAGGAAAGATTCATGAACCA
USP17L2_1	ACACGGGCCTCAGATGAGAG
USP17L2_2	ATTGCGGTCTTTGTCTCCAG
USP17L2_3	CAGGGAAGCGTTCTCGTAGC
USP17L2_4	TGACATGACCAGGACTGTGG
USP17L2_5	GAAGTCACCACTCTCATCTG
USP21_1	GAAAGGCTCAGAGGACCGGG
USP21_2	GCTCCACCGATACTTGCCAA
USP21_3	TAAGGAAGAAGAGCTAGAGT
USP21_4	TTCCGGCAAGAGGTGCCTGG
USP21_5	AAGACAGCTCGGAATCGAGT
USP22_1	AAGGCGAAGCGGCACAACCT
USP22_2	AGACATGGAAATAATCGCCA
USP22_3	GATGTCCTCACTGTTTCAGG
USP22_4	GTGTCCCGGCCAGAGCCCGA
USP22_5	ACCTGGTGTGGACCCACGCG
USP3_1	CTACACTTGGAACTTCAGGG
USP3_2	GAACAAGTCAAACAGACCCA
USP3_3	GCTGCAGCACCAGGACGACG
USP3_4	TTTCAAAGAACTGCCCGCCG

USP3_5	CCCATTCCTTAACTCCACGG
USP34_1	AGCGATACACATCAAAGTGG
USP34_2	GAACTGCGCAGACCTGGTGG
USP34_3	TATAATGTGCTGCATGACAG
USP34_4	TCAGTATCAGATGTAGAAGG
USP34_5	GTCTTGTATCACACGAACTG
USP35_1	CATGGACAAGATCTTGGAGG
USP35_2	GAAGTTCAGCATCTTGATCG
USP35_3	GCACAGACAAGGCAGCTCCC
USP35_4	GGAGCCACCATCTAGCGCCC
USP35_5	AGAACTCGGCGAAGACGTCG
USP36_1	ATTCAGGCAGGCTTTCTGCA
USP36_2	GGCGTTGCCGCTGTTGGCGA
USP36_3	GTGGAGATGACCCACCGGCC
USP36_4	GTGGGAGCGGGTCTTCCGCG
USP36_5	CAGGTTGGATCGTCAAACGC
USP44_1	CCAGAAAGGTATCAATGCAG
USP44_2	GATGGAACTTATTCAGCCAA
USP44_3	TATCTATGACTTGTCCGCGG
USP44_4	TATGATATGCCACCTACCTC
USP44_5	ATCACTGCACAACTCGTAGT
USP46_1	GAAGTACAATGCCTGAAGCA
USP46_2	GTTGCGGTGGTCGTTCACTG
USP46_3	TATTGCGGACATCCTTCAGG
USP46_4	TTTCCAGTGGTCCTAATCGT
USP46_5	CTTTGATAACTACATGCAGC
USP49_1	AGAAAGCTGAGTCTTCCCGT
USP49_2	ATAGCGTTCAGGGAATTCCA
USP49_3	TCTAGCTTCACTCAGAACAA
USP49_4	TGCGGAGAGATCATAGGCAA
USP49_5	CACCAAACAGGTCTTAAAGG
USP51_1	ACAAGAGCGTGAGCCCGCGC
USP51_2	AGGAGCTGCTGTGACAGCGA
USP51_3	TAAGTCCTCAATGGTAGCCT
USP51_4	TTGACGTGGAGCAGCAGCGG
USP51_5	CACAGACCAGACACAAGCTG
USP7_1	AAGAAGCACACAGGCTACGT
USP7_2	AATCAGATTCAGCATTGCAC
USP7_3	GAAGTGACCGATCCTGAGAA
USP7_4	TGATGGACACAACACCGCGG
USP7_5	AGATGTATGATCCCAAAACG
UTP3_1	AGGATTGCCACCCTCACCAG
UTP3_2	AGGCCGGCAGAGTCAACAGG
UTP3_3	GAGTCAACAGGAGGCAGAGG
UTP3_4	TAGGATTGCCACCCTCACCA
UTP3_5	AGGAGGCACAGATCATTCAG
UTY_1	ACAGCTATCTAACTCCGCTC
UTY_2	AGTAGAAGTAGACCAAACCA

UTY_3	AGTAGGAGACAAAGCCACAA
UTY_4	GTGATACTGGGCTGACCCAG
UTY_5	AGGATTCATAGAGAGTACCT
VAX1_1	AAGTAAATCCAATTCCGCAG
VAX1_2	GACGCGCACGTCCTTCACCG
VAX1_3	GGAGTAAGGCTCGAAGGCCG
VAX1_4	TCGGGAGAGCAAGGGCGCGG
VAX1_5	ACGTGCGCGTCCTCTTAGGC
VAX2_1	ATGGCCTCTGCAGATGCCAA
VAX2_2	GAGAGTGGAGCCGACAGCGA
VAX2_3	GGAGGTGGCAAAGGCCTCGG
VAX2_4	TACCTCGCACCAGTATGCGG
VAX2_5	CGGTGGCCACAGCCCAACGG
VCPKMT_1	ACTCGCACAAAGCTCCGCAG
VCPKMT_2	CGCCCGCCAGCTCTTACCCG
VCPKMT_3	GATGATTAAAGGCTTCACGA
VCPKMT_4	GCCCGCCAGCTCTTACCCGA
VCPKMT_5	CTCGCACAAAGCTCCGCAGT
VDR_1	AACTGACCAGGTCAGCCAGG
VDR_2	CAGGCGAAGCATGAAGCGGA
VDR_3	GAACTGGCAGAAGTCGGAGT
VDR_4	GATACAGTGATCTGAGCAGG
VDR_5	ACAGCTCTAGGGTCACAGAA
VENTX_1	AGGCAGATTTGAGGACCCGG
VENTX_2	GAAGGCTGTGCGGACACGGG
VENTX_3	GACAGGGCTGGTCCCAGCGA
VENTX_4	TGTGTGCTATGCCACAGACG
VENTX_5	AGACGTTGAGTAGAAAGCTG
VEZF1_1	GAACAGGAACGCGGTCCAGT
VEZF1_2	GAGATGGTACACATCTCGGA
VEZF1_3	TATGTCAAACCCAGTCACAG
VEZF1_4	TCAGGGACAGCTATCACCTG
VEZF1_5	AACAAGGCATCAGTAAAAGT
VPS72_1	GAAGCGCCGAGTAGTCACCA
VPS72_2	GCCTCGAAAGGTCAACACCC
VPS72_3	TAGGCGGTATGAGTTTGGCT
VPS72_4	TGAGCGGCCACTAACCCAGG
VPS72_5	CCCAGAAAGCCGGTTCCCAG
VRK1_1	ATGGTCAAACACACAGACAG
VRK1_2	GTAGGATTACCCATTGGCCA
VRK1_3	TGGAAAGTAGGATTACCCAT
VRK1_4	TTTAAGGAACCCAGTGACAA
VRK1_5	CCCAATACTTAGGAACACCC
VRK2_1	AAGGTTCTGGATGATATGGA
VRK2_2	ACCGCAGCATGCAGTAGCCG
VRK2_3	TCGGCTACTGCATGCTGCGG
VRK2_4	TTACAGCAACTGCTTCCAGA
VRK2_5	ACCAGCTTGGATGCCCACAA

VSX1_1	GAGACCAAGAAAGTGCACCC
VSX1_2	GCGTGCCGAAGCCACAGAGG
VSX1_3	TCCGTGCTCAACTCCGCCGA
VSX1_4	TGGGCAAGAGGAAGAAGCGG
VSX1_5	GCGGGCCTGATTACCGGACG
VSX2_1	AGTGGCCAAGAGTACCTCGG
VSX2_2	CATGGATGAAGACAGGCCGG
VSX2_3	GAACCCAGTGCACCTGGCCG
VSX2_4	TTGAGGATGGACTCGGGCAG
VSX2_5	AGCCATTGGGCAGAGCATCG
WAC_1	AGAATTATTAAGCTGCAGCG
WAC_2	GTAGCAGTTGGATGAACCAC
WAC_3	GTCGAACAGAAGTTTCACAA
WAC_4	TTGAACTATGAAGAGCACTG
WAC_5	ACTAGAAGGAACAGTGCTTG
WBP11_1	AAAGAGGAATCCCATTCTGA
WBP11_2	CCGGAAGAGAGAATTAAAGA
WBP11_3	TCTTCATCTCGCCTACGAGG
WBP11_4	TGAAGATGATGGCTATCCTG
WBP11_5	ACCTGCCATACGAAGCATCA
WBP5_1	AAGGCTGATTCAATCTCTCC
WBP5_2	TGAGAGTGAACCAAAGCATG
WBP5_3	TGAGGAAGAGCCAAAGCCTG
WBP5_4	AAATCCTGTCAAAAAATGGA
WBP5_5	AGAGGAGGAGAAGCTAGAGG
WBSCR22_1	ACAAAGGCAGGCTTCTCCGG
WBSCR22_2	AGATGAAGGGCACTATTGGG
WBSCR22_3	TTAGCATTTCTGCTGTGCAG
WBSCR22_4	TTTATGACGAGACAGAAGCC
WBSCR22_5	AATCTCACCCAATATCCAGC
WBSCR27_1	ACACACAGGGACCTTCGACG
WBSCR27_2	GAGGCGCTGATAGAGGCCGG
WBSCR27_3	GGTATCCGGCATCTCTGCCA
WBSCR27_4	TGAGGCAGTCCACTGCGAGG
WBSCR27_5	AGCTCAGGTATCGCATTGCA
WDHD1_1	ACTGCTTGATGTCTTTCCAC
WDHD1_2	ATAGTTACTGGCAATTCCTG
WDHD1_3	GCATACAGAGGGACACACGG
WDHD1_4	TTTCCTGAAGGAGTTCCAGA
WDHD1_5	AAAGCACTGATGAACTAGCA
WDR11_1	AATATAGTGCTTACCAGCGA
WDR11_2	CAACGCCCACAACAAGGCGG
WDR11_3	GAGCATGCCAAGCCTATCCA
WDR11_4	GGATAGCAAGCAGTAAATCG
WDR11_5	CAATATAGTGCTTACCAGCG
WDR33_1	ATGTTGACAGCAGACCACGG
WDR33_2	CATGAGGAAAGAATTCTCCG
WDR33_3	GTAGACTGGCATCCAACCAA

WDR33_4	TGAAAGTGAGTCCATTCCAC
WDR33_5	AAAGAGACCAGAGAGATATG
WDR4_1	CTGACACTCAGACATTGCCA
WDR4_2	GAACAGAAGCAGCTACCTGG
WDR4_3	TCTAGACGGCCACACCCGTG
WDR4_4	TGAGGCGGGCGGGTACATGG
WDR4_5	GGTGTGGCCGTCTAGAGCTG
WDR48_1	AAAGGCCTTAGCATATGCCA
WDR48_2	ACCGGCAGAACACAGCAGGG
WDR48_3	GCGCCCAAACACCTTCATCA
WDR48_4	GTTGCAACCCAAATTGCAGG
WDR48_5	AATGAAGCTTAAAGGGCACA
WDR5_1	ACGCACCTTGCTCTGAGTGG
WDR5_2	ATACATCCTGGCCGCCACGC
WDR5_3	ATTCGGGCTGAATTTCACGG
WDR5_4	GAGACAATAAGGTTGGACTG
WDR5_5	ATCTGACGACCAGGCTACAT
WDR61_1	AGTCTGGAGGGACATCAGCT
WDR61_2	GATGGGAAATACCTAGCCAG
WDR61_3	GGTTAAGGAAATGACCAACC
WDR61_4	TGCAACGTTCAGCACCCAGG
WDR61_5	AAGTCCATAGATGCAGGACC
WDR77_1	ACAAAGACTTCACTTTGCTG
WDR77_2	GCAGCGCCCACTCAGGCTGG
WDR77_3	GTGGTACTGAGTTCATACCG
WDR77_4	TCTGGCACACAAGCTGTCAG
WDR77_5	CTCCGCCGGAGTCCAAACGG
WDR82_1	ACAGGGTCAGTCATCAATGG
WDR82_2	AGGGCCTCATGCATCTGCAG
WDR82_3	CAGACGAATGAAGCTGCCGT
WDR82_4	CTGGCAGTCATAGAGCACGA
WDR82_5	GTACAGTAAGAAATATGGTG
WDR92_1	GATGATCTGAGGCTTCTCGA
WDR92_2	GGCAGACTTACTACTTCCAG
WDR92_3	GGTGGACTAGGAATTGGAGA
WDR92_4	TCAGTGAATACCCTATTCAG
WDR92_5	AAGGCCAAACCTATTAAATG
WHSC1_1	AAAGAGTCTTGTCCAAACAC
WHSC1_2	GCAGCTCGGAGTCTTCCCGT
WHSC1_3	GGACAAGCACAGTCTTCGGA
WHSC1_4	TTCCCATGAAGAGAAGGCGG
WHSC1_5	ACAACGTTGGTGATTTGGTG
WHSC1L1_1	AAATTCGGAGACATAGCCAG
WHSC1L1_2	GGAGATGATACACTCTGCGT
WHSC1L1_3	TATGAAGCTACTTTGCAGCA
WHSC1L1_4	TCACCTAGAGACCAAATGAG
WHSC1L1_5	ACACTTTACACGAAAAACAT
WIZ_1	GAGGAGGCGGTCCTCCTGCA

WIZ_2	GATCTCCCGCAGCGTGTCGA
WIZ_3	GGGCTGCGGAGGCATCTGGA
WIZ_4	GTGACAAGCGGCCGTCCCTG
WIZ_5	CGTGACTCGACAGGCCCTTG
WNT5A_1	CAACTGGCAGGACTTTCTCA
WNT5A_2	GATGTGGCCTGCAAGTGCCA
WNT5A_3	GCGGGAGCGCATCCACGCCA
WNT5A_4	TTCAATTACAACCTGGGCGA
WNT5A_5	AGTATCAATTCCGACATCGA
WRN_1	CTAACATTGAGACTGAACTG
WRN_2	GAAATGGCTGCAGATCCTGA
WRN_3	TAGGAATTGAAGGAGATCAG
WRN_4	TGGAAACAACTGCACAGCAG
WRN_5	ATCCTGTGGAACATACCATG
WSB2_1	CTTGCTACGGGACTCAACGA
WSB2_2	GATCTCTCACGACATCTTGG
WSB2_3	GCGTCCTCCATGGAGGCCGG
WSB2_4	TGGACTTCCAATCAAACTGG
WSB2_5	ATCTGGGACCTGAATAAACA
WT1_1	ACTTCAAGGACTGTGAACGA
WT1_2	ATTCAAGCATGAGGATCCCA
WT1_3	GGTGTGGCAGCCATAGACCG
WT1_4	GTAGCTGGGCGTCCCGTCGA
WT1_5	ACGTCGGAGCCCATTTGCTG
WTAP_1	AACAGCAGGAGTCTGCACGC
WTAP_2	CTTGGAAGGCAGCTGTCCCA
WTAP_3	GAAAGTGTAGACTCTCCCAC
WTAP_4	TGAAAGTGTAGACTCTCCCA
WTAP_5	GAAGGAGACACGCCAGCAGT
XBP1_1	CAGGAGTTAAGACAGCGCTT
XBP1_2	GACTCAGCAGACCCGGCCAC
XBP1_3	GGCTGGCACCATGAGCGGCA
XBP1_4	TCAATACCGCCAGAATCCAT
XBP1_5	AGGAGTTAAGACAGCGCTTG
XPA_1	AAGATAATTGACACAGGAGG
XPA_2	ACCGAGGCAGGCAGCTCCGC
XPA_3	GGCGGCTTTAGAGCAACCCG
XPA_4	TGCCCGGCCCTACTCGGCGA
XPA_5	CAAGCTTATAACCAAAACAG
XPC_1	AAGGATGAAGCCCTCAGCGA
XPC_2	AAGGCACACCATCTGAAGAG
XPC_3	AGGCGAAGACAAGAGAAAGA
XPC_4	AGTTTGAGACATATCTTCGG
XPC_5	GCATAGCTGGTATAGACCAG
XRCC1_1	GGTACAGCTTACCTGGGACG
XRCC1_2	TGATGGCTCAGCTTTCGTGG
XRCC1_3	TGCAGGACACGACATGGCGG
XRCC1_4	TTGCAGCCAGCCCTACAGCA

XRCC1_5	ATGAGAGCGCCAACTCTCTG
XRCC2_1	ACTTACAGGAGTCTACTCTG
XRCC2_2	GATGAAGATTCACCTGTGCA
XRCC2_3	GATGTGTAGTGCCTTCCATA
XRCC2_4	TAGGGCTGAGTCTGGGACCG
XRCC2_5	AGGAACTTCTACCTTCAAGT
XRCC3_1	CCTGGCTGTCAAATTCACAG
XRCC3_2	GAGGAGCAGGGCGCAGCACA
XRCC3_3	GGAACGTCAGTGCGCAGCCG
XRCC3_4	GTGCTGGACGCGCTGCTCCG
XRCC3_5	AGGAGCCGTCTACATCTGCA
XRN2_1	CATGAGGTCAAAGATTGTGA
XRN2_2	GAGTCCCGGCGTTCTTCCGC
XRN2_3	GATCAGCTATGTAATAGCGA
XRN2_4	TGAACCAGCAGCGTTCAAGG
XRN2_5	CAGCTGATGAGAAATTCCGT
YAF2_1	CACCTTCCGGAACAGCGCCG
YAF2_2	GCCCGGGCGCTGTTACCTGG
YAF2_3	GTCCCAGTAACCCTCATCCG
YAF2_4	TCCAGGTCATCTTCACCCAG
YAF2_5	AACCTGCTGTGCAACCAACT
YBX1_1	GCTTGGTGTCGGCGGCGCTG
YBX1_2	GTGAGGCAGAATATGTATCG
YBX1_3	TGGCCCGGGCGGCCTCACAT
YBX1_4	TTTCAGGGTGCTGACAACCA
YBX1_5	AGACGCTATCCACGTCGTAG
YBX2_1	CTGGGCCGGAATCTGGGCGG
YBX2_2	GAGTTGGAATCACTCCAGGA
YBX2_3	GGAGCCGCAGAAAGGCGGCG
YBX2_4	TAGGCTGCTGGCGTGGCCTG
YBX2_5	AACCGCCGTCGGTAGAAGAA
YBX3_1	ACACTGCGCAGATATTTCCG
YBX3_2	GAACTGCCTATCAGTGGCAG
YBX3_3	GATCGGCGCCGTTACAGACG
YBX3_4	GTTTCCTGCGACGTGGGCGG
YBX3_5	ACGTCGCAGGAAACCCCGGT
YEATS2_1	AGTGGATGGTATATGTCCGA
YEATS2_2	GCAGGAGATGATGACCTAGA
YEATS2_3	GCTGGAAAGCTGAATTGCCA
YEATS2_4	TGGTGAAGAAGCTTTCACTG
YEATS2_5	AGCCCCAAACCTATAACAGG
YEATS4_1	AAGATGGGCACACTCATCAG
YEATS4_2	ACTGACCTTTACTCTCCCGC
YEATS4_3	CAGACACCAATGCAATGCTG
YEATS4_4	GAATTTGGGCCTGACTCCGG
YEATS4_5	AGCTATGGCAATCCTTTAAG
YLPM1_1	AAAGGGTCCTGTGGTAGCAA
YLPM1_2	GATGGACCAGCTGATTGCAA

YLPM1_3	GCGGAAGCTCATGAAGCCCG
YLPM1_4	GGGAGAGAGGACCACCTCGA
YLPM1_5	AAGTGGGGAATGATTCCCCG
YTHDC1_1	CATGGCGGCTGACAGTCGGG
YTHDC1_2	GAACAACACTGAGAATGAGG
YTHDC1_3	TGTGTCTCTTGCCAAAGCGA
YTHDC1_4	TTGCTAACAGATGAGCTCAG
YTHDC1_5	ATGAGTGCTAAAATGCTGGG
YTHDC2_1	ACAAGTGGGCGACTCAACAA
YTHDC2_2	ACGATTGGCAGCTATCGCTG
YTHDC2_3	ATGTGAATTGCCTTGAACCA
YTHDC2_4	GATCGCAGTCAATATCGCGC
YTHDC2_5	GACTGTGACACATGTTATCG
YTHDF1_1	AAGCATGTCGGCCACCAGCG
YTHDF1_2	ACTGGTGCCGTAGTCCACGG
YTHDF1_3	GCGGCGTTTGGGCAGAGCGG
YTHDF1_4	TAAGGAAATCCAATGGACGG
YTHDF1_5	AGTTTCAAAGCCGACCTCGT
YTHDF2_1	AATATAGGTCAGCCAACCCA
YTHDF2_2	ATATAGGTCAGCCAACCCAG
YTHDF2_3	CTACTCACGGGCCTTGCCTG
YTHDF2_4	TATGACCGAACCCACTGCCA
YTHDF2_5	ATGGAGGGACTGTAGTAACT
YTHDF3_1	CAACCGAAACTTAAACCCAA
YTHDF3_2	TGCTGGATTATAGTTTGAGG
YTHDF3_3	TGGGTAGCTCCTCGTAACAG
YTHDF3_4	TGGTGTATTTAGTCAACCTG
YTHDF3_5	ATGCTGATTTCTCTACATGG
YWHAB_1	CTGCTGGGAGTTCGACACAG
YWHAB_2	GAAAGCCAAACTCGCTGAGC
YWHAB_3	TGAAGGCAGTCACAGAACAG
YWHAB_4	TGACACGCCAGGAAGAGCGG
YWHAB_5	AGAGTACCGTGAGAAGATAG
YWHAE_1	AAGCGAATAGGATGCGTTGG
YWHAE_2	ACTTCAGACATGCAGGGTGA
YWHAE_3	CCTAAGCGAATAGGATGCGT
YWHAE_4	GCAGAATAAAGAAGCGCTGC
YWHAE_5	AATTTGCCACAGGAAACGAC
YWHAQ_1	CCGAGGACTCACCAGCACCG
YWHAQ_2	GAAGACTGAGCTGATCCAGA
YWHAQ_3	GCTAGAGATGACCCTCCAGG
YWHAQ_4	GGGCGCCGAGCTGTCCAACG
YWHAQ_5	GCCTACAAGAACGTGGTCGG
YWHAZ_1	AAAATGTTGTAGGAGCCCGT
YWHAZ_2	GCCGCTGGTGATGACAAGAA
YWHAZ_3	GGCTGAGCGATATGATGACA
YWHAZ_4	TCTCTGCTTGTGAAGCATTG
YWHAZ_5	AGATATCTGCAATGATGTAC

YY1_1	AAGAAGAGTTACCTCAGCGG
YY1_2	CGAGGTGGAGACCATCCCGG
YY1_3	GAAGAGTTACCTCAGCGGCG
YY1_4	GATGTAGAGGGTGTCGCCCG
YY1_5	AGATATTGACCATGAGACAG
YY1AP1_1	ATCGCTCTCGGCCTCCGACA
YY1AP1_2	TGAACAACATCAGATAGCGA
YY1AP1_3	TGATGAACTTCCTTACACTG
YY1AP1_4	TTTGATACAGCGAAAGGCCC
YY1AP1_5	AGGGGAAACGATCCCAGTGT
YY2_1	AAGGGAAGAAACTTCCTCCT
YY2_2	GATGGCAATTGGATCTACGG
YY2_3	GCTGGTGGCACTCTTGCCGC
YY2_4	TCCTATGGAGGACATTCCGA
YY2_5	GGAGCTCCACGACATCAATG
ZAR1_1	ACACATAAGCACTCTCCCAG
ZAR1_2	GCAGTCCGACGACGACGGCG
ZAR1_3	GTAGCTGTCGAAGTACTCGG
ZAR1_4	TACCGCACAAATCTTGACGG
ZAR1_5	AGTACTTCGACAGCTACCAG
ZBED1_1	CATCACGCGAGTGCTCTATG
ZBED1_2	CGAACTCGCAGAATTCCTCG
ZBED1_3	GCATCTCCACCGACATGTGG
ZBED1_4	GCTGTACCCAGCCTCCATCG
ZBED1_5	GCAGCAGTTCGTCATCGCCG
ZBED2_1	ACGTGGGCACCACTGCACTG
ZBED2_2	GAAGAGGTGACATGATGAGG
ZBED2_3	GTGGGCATAGCACTCACAAA
ZBED2_4	TTGGCTGGCCCACAAAGCCA
ZBED2_5	ACAGGCATTGAGGGTAACTG
ZBED3_1	AGTGGCCCGACGGATGCCCG
ZBED3_2	CAGGTGCCTCCACAACGCCG
ZBED3_3	GGACGGCTGCGTCATCACAA
ZBED3_4	TGGCGAGCCGGCCTGCACCA
ZBED3_5	ACAACGCCGAGGTCCCCGCG
ZBED4_1	ATGAAGCAGACAGACAGCGG
ZBED4_2	GGAAGAGGAACTTTCTGCAG
ZBED4_3	GTAGCGAGGATCCAGCAGCG
ZBED4_4	TGTGGCCGGACCATCAGCCG
ZBED4_5	AGCTGCAACCCGTCCCACCG
ZBTB1_1	ATGGCAATGAAGCCAACAGG
ZBTB1_2	GAATGGGTTAGGAAATACTG
ZBTB1_3	GCACAGACGGACAAATACAG
ZBTB1_4	GTGTGGACTTACAATAACCG
ZBTB1_5	AAATACACCACATAATAGAG
ZBTB10_1	ACTCCACGAAGCCAACGCCC
ZBTB10_2	GATGGTTGTAATGTCGACGA
ZBTB10_3	GCAGATAAATCACAGCCAGG

ZBTB10_4	GCTGGCACTAGTCAAGATGG
ZBTB10_5	GCCACACCGAGGTCTCGGGG
ZBTB11_1	ACAGGTTGACACCAAAGGAG
ZBTB11_2	ACGCCGGGACCTCATCGAGG
ZBTB11_3	ACTTAACACACAGTTCACAC
ZBTB11_4	TCAGCCTCTGAAGAAACCTG
ZBTB11_5	AATAAGATGTGCTCGCAAAG
ZBTB12_1	AGACTTGGAGCTGAAAGCCG
ZBTB12_2	AGTGAAGCTGGAGTTCCCAC
ZBTB12_3	GGAGCAACACGGCAAGACCA
ZBTB12_4	TCAGCAGGAACTGGTCCCGC
ZBTB12_5	AGGCGGCCAAGATGACCTTG
ZBTB14_1	ACAGTTGCAGGCGGCAGCGA
ZBTB14_2	GAAGCATGAGAAACTCCACA
ZBTB14_3	GGCAGGCAATCTGCTCCCGA
ZBTB14_4	TGTGTGTGGCTCCTGCACCA
ZBTB14_5	CTAAATGAACAACGCCTGGA
ZBTB16_1	CCTTACAGGAAGCTGCACAG
ZBTB16_2	GAGCCACAAACGCATCCACA
ZBTB16_3	GCAGCACATGGAGGTCCACG
ZBTB16_4	TATAGTGTTGACTATTGCGG
ZBTB16_5	CATCTCGAAGCATTCCAGCG
ZBTB17_1	GACCTCAAGGAGGAGCGCGG
ZBTB17_2	GTGAACACCGTCCACCACAA
ZBTB17_3	TCTGGCTTGAGCTCCACAGG
ZBTB17_4	TGAGCCGGCTACCAGCCCTG
ZBTB17_5	CTCCGAGCTCTCGGACAAAG
ZBTB18_1	AAAGTCGAGAGTCTCTCCGA
ZBTB18_2	AAGCAGGAGAGCGAAAGCGG
ZBTB18_3	GAAAGAGAAAGCCACCACGG
ZBTB18_4	GCACATGAAGATCTGGCCCG
ZBTB18_5	AACATCTGCCGAATCCCTCA
ZBTB2_1	AATGTCAGCAATGTCTGAGG
ZBTB2_2	GAAGCCAGCCTCGCCAGCCA
ZBTB2_3	GAGCATCGACACTTACACCA
ZBTB2_4	GTTGCAGTAGAATAAGTCCA
ZBTB2_5	ATGTGAAAGGTACTCCTGTG
ZBTB20_1	AGGCGTGGTGGCCTGCACGG
ZBTB20_2	GAAGACACAGACCAGGCCGA
ZBTB20_3	GGAAGACACAGACCAGGCCG
ZBTB20_4	GTGCACATGCGCCTCCACCG
ZBTB20_5	CAGCGACATCGAGATCCCGT
ZBTB21_1	AAGAGAGCTTAGCATTACAG
ZBTB21_2	AGTGACGCGGATGATGTCCG
ZBTB21_3	GTGACGCGGATGATGTCCGA
ZBTB21_4	TATGCGCACCTCAGTCACAG
ZBTB21_5	CAAAGTAGAAACGAAGCGCA
ZBTB22_1	AAGGGAAGGAGCGGTCGAGG

ZBTB22_2	GAGGCGGCCAGTGTAAGCGG
ZBTB22_3	GAGGTGGAGGAGCAAACTGA
ZBTB22_4	GGTACATGTGTCCTTCCCTG
ZBTB22_5	ACGGACATCACTTATGCTGA
ZBTB24_1	ACACATCCATGAATTTGCGA
ZBTB24_2	AGAGTCGATACAGCAAGCGG
ZBTB24_3	GAGCACATGAGCCTGCACTC
ZBTB24_4	TGCCCATCAAGAGCAAACAG
ZBTB24_5	AGGACCATGGTTCAGCCAAG
ZBTB25_1	ACAGATATAACCGATGCCAA
ZBTB25_2	CAAGAGACAACTGCAACTGG
ZBTB25_3	GAAGCTCCTTCCAGTAACAG
ZBTB25_4	GGAAACAGAGCTGCTGTCCA
ZBTB25_5	AGCAGGGACACCGAATGGCA
ZBTB26_1	AACGGTGCACACAGGCCCTG
ZBTB26_2	CCAGTGCCCAAAGTGTACCA
ZBTB26_3	GATCATCTTAACCTTCACAG
ZBTB26_4	GTGTAGACAAAGGCCTACAG
ZBTB26_5	ATATTACAGAGTTCCGAAGT
ZBTB3_1	AAAGTCCAGAAGCAGCCCAA
ZBTB3_2	AGCCGTTGGACGCTACCGGG
ZBTB3_3	AGGAGGCCAGCACAGCCCGA
ZBTB3_4	GTGGAACCAAAGGATCCTGG
ZBTB3_5	AACTCCATAGTACCCCACGA
ZBTB32_1	CAGGGAGCAGCGGTACGGAG
ZBTB32_2	GATGGCAGGAGCAACGCAGG
ZBTB32_3	GCAGGAACAGACCAGGTCAA
ZBTB32_4	TAGGGCCGAGACCGAGCAGG
ZBTB32_5	CACAAGCACTCGCCACCAAG
ZBTB33_1	AATACTAATGATCCAGGCGT
ZBTB33_2	AGGAAACAAGGCCAATGAAG
ZBTB33_3	GTTGATGAAAGATTTGCCAC
ZBTB33_4	TTTGCCGAGTAATAACACAG
ZBTB33_5	AAGCATCTCAGGTACAGCGC
ZBTB34_1	ATGGAGGAGAGATCTCCACT
ZBTB34_2	CCTCTCGCACTCCTCTCCCG
ZBTB34_3	GGAATCTAGGCCCATCTCTG
ZBTB34_4	TCCTGACTTCAGCAACGCCT
ZBTB34_5	GATGTTGACTCTGTTACCGT
ZBTB37_1	AAGGGAACAACACTGCCGGA
ZBTB37_2	AGCAGGAAGTATCTGAGCGG
ZBTB37_3	GAGACAGGAGTGGCGGACCG
ZBTB37_4	TATGTTCCCACCTTTCTCCA
ZBTB37_5	ACCCCAAAGGGAAACCGGCG
ZBTB38_1	AAAGACATTCTCCATACCAC
ZBTB38_2	AAGCTGCCAGAACATTGCTA
ZBTB38_3	AGCAGAACTGGATTGCGCCG
ZBTB38_4	GAGGACGGACGTCTGCCACG

ZBTB38_5	AAGCCCGAGCCAGATAAAGT
ZBTB39_1	AATGCAGTAGACATTACCAC
ZBTB39_2	AGTGCCCACAGACTGTGCAG
ZBTB39_3	GAAGCGGAAGCTGCCAGCAG
ZBTB39_4	GTACATGAAGGTCTGCTCGA
ZBTB39_5	AATGTCTACTGCATTGTCAG
ZBTB4_1	ATTGAGCTGGCGCAGGACGG
ZBTB4_2	GACGTGCCCGCACAGAGCGG
ZBTB4_3	GCAATGTACACTCGTGGCGG
ZBTB4_4	GGAGGCCCGGGCTAATGCCG
ZBTB4_5	ACAGCAATGTACACTCGTGG
ZBTB40_1	AGAGAAGACGCTGACTGCTG
ZBTB40_2	GCTGTAGTAGGAGTCTCCGC
ZBTB40_3	GGAGACAAAGACCTGTCCAT
ZBTB40_4	GTAGACACAGTGCAGCCAAA
ZBTB40_5	AAACGTGACTCTGGTTCAGG
ZBTB41_1	TAATCAGTTGACTGTGGCTG
ZBTB41_2	TGAAGAAGATGAGCTAGAGG
ZBTB41_3	TTCAGAAGGAAATGTTGCAG
ZBTB41_4	TTTGCCACACTTTCTCTCCA
ZBTB41_5	GGTCGCCAGTTTAACGACAC
ZBTB42_1	AAGGATCGAAGTCTGGACCC
ZBTB42_2	ACCGTGTCGCGACTGCCCGC
ZBTB42_3	ATGGAGTTCCCTGAGCACGG
ZBTB42_4	GGAGGCCCAGATGCTCGAGG
ZBTB42_5	AGGATCGAAGTCTGGACCCG
ZBTB43_1	AAGTCCTCCTCCACTCCCGA
ZBTB43_2	GGCTGAGCAGAATACAACTG
ZBTB43_3	TACAAGAAGTCACATGCCGG
ZBTB43_4	TGTGGAGGGACAGTTTCCAC
ZBTB43_5	AGTACCTGGTCACAAAAGTA
ZBTB44_1	CAAACTTGTAGGCCAAGCCG
ZBTB44_2	GACCGATCATCATCATCCGG
ZBTB44_3	GCTGAAGAATTCAATACCTG
ZBTB44_4	TGTGCCGGGAAACCCTCATG
ZBTB44_5	CTAAGGAAGAGTCAACTGGT
ZBTB45_1	GAAAGTGACGATGAGACCGA
ZBTB45_2	GCACTGGAAGGCGCGCACGC
ZBTB45_3	GTGCGAGAAGACCTTGCCGC
ZBTB45_4	TATGCAGTCGGGCTGGACAG
ZBTB45_5	CAGCTGTCGCACTGTCTGCG
ZBTB46_1	AGGCGGCGAGGGCTCTCCAG
ZBTB46_2	CCTGCATCAGGCCACCGCGG
ZBTB46_3	GAAGACGTCGGAGCAGGCCA
ZBTB46_4	GCACATGAAGCGCCACACGC
ZBTB46_5	CAGCACGGAAGCTCTCATCT
ZBTB47_1	AACCTGAAGAAGCTGGGCGG
ZBTB47_2	CCACCTCGTAGCCCACACCA

ZBTB47_3	GCAATCCCAGATCATCGTGG
ZBTB47_4	GTGCTTACGCACGTGGGCCA
ZBTB47_5	ACTGGTCGCAGATCTGCATG
ZBTB48_1	GCCAGGAGACATTCCGCCGA
ZBTB48_2	GCTGAGCCAGCCCTGAGCGC
ZBTB48_3	TCTCAGCGAAGCCAGCAGGG
ZBTB48_4	TGGGAAGTGTTACTTTCGGA
ZBTB48_5	ACACAAACAGCTTCTCACCA
ZBTB49_1	ACAAGGCTGGCTCTCTACCG
ZBTB49_2	ACTTGCAGACTCACTTACGA
ZBTB49_3	GCACAGAATTCGGCACACGG
ZBTB49_4	GTGACGCTGGACGTCGCCAG
ZBTB49_5	CACCTCTACAGACCTTACCA
ZBTB5_1	ATGCTGCCAGCACGGAGCGG
ZBTB5_2	TAAGCGCAAGCAGTCTGCAG
ZBTB5_3	TAGCGAGGTGGTGACAGCAG
ZBTB5_4	TGAGAAGGAGCACATGAGAG
ZBTB5_5	CAGACTCATCTATGGAGGGT
ZBTB6_1	AATCTCAACCGACACATCCG
ZBTB6_2	GAAAGCCTCGAGGACACCTG
ZBTB6_3	GACCACTTGAACATACACAG
ZBTB6_4	TCAACATGAAGAAAGCCTCG
ZBTB6_5	CAGGATCAGAAGACTGACAC
ZBTB7A_1	ATTCAACCGGCCCAAGCCGT
ZBTB7A_2	GATCGACTTCGTCAGCGCCG
ZBTB7A_3	TAAGCCGTTGCAGTCGCCCG
ZBTB7A_4	TCCGGCTGTGAAGTTACCGT
ZBTB7A_5	ACCGTCAGCACAGCCAACGT
ZBTB7B_1	ACGGCCTCTGGAGTTCCCAA
ZBTB7B_2	GGAGAGGTCGAGGCCAGCGG
ZBTB7B_3	GTGCCACAAGGCTTTCGCCA
ZBTB7B_4	TGGCAGACAGGGCACTCCTG
ZBTB7B_5	AGCAAACCACCTAGTCCCTG
ZBTB7C_1	AGAGCGCAAGCTGAAGCCCA
ZBTB7C_2	CAAGCGCCAGAGCTGCCGCA
ZBTB7C_3	GATGTGCTTGACATTGCCAG
ZBTB7C_4	GGAGGCTGAGAGGAACGCGG
ZBTB7C_5	AAAGTCGATCTCATAGACGT
ZBTB8A_1	AACAGGGCAAGTGGTACAGG
ZBTB8A_2	CCTTGGCCAAGCATGAACCA
ZBTB8A_3	GGGCACTTGAATCGCATCCG
ZBTB8A_4	TTGTTGAGTATTCTTCTGGG
ZBTB8A_5	AATGTATTATTCGCTAGTAG
ZBTB9_1	AGAAGCAGAGGACTGTACTG
ZBTB9_2	GGAAGCGGAACTCAGCCTGG
ZBTB9_3	GGAGTAGCAGTCATGGGCAG
ZBTB9_4	GGGAATGAAATCCTGTCAGG
ZBTB9_5	AAAACCCCTGATACTCTCTG

ZC3H8_1	AAGGATCAAATGCTTTGCTG
ZC3H8_2	ATAGTTTGACTCTTACCCTG
ZC3H8_3	CAACATACAGTGGAACGCAA
ZC3H8_4	GTACATACAAGCCAGAGAAA
ZC3H8_5	AGTTTGACTCTTACCCTGTG
ZC3HAV1_1	GAGGAATCACAACTTACCTC
ZC3HAV1_2	GTCAGGCCGGGACAAGCCAG
ZC3HAV1_3	TCTGTGACCACTTCACCCGA
ZC3HAV1_4	TTTGTGGTGTTGGAGACCGG
ZC3HAV1_5	ACTTCCATCTGCCTTACCGG
ZCCHC4_1	AAGGGAAAGAAGAAACTCGG
ZCCHC4_2	CAGGAATGGGTTTGAAGCCG
ZCCHC4_3	TCAACCAAGCCACCAAACGG
ZCCHC4_4	TTACAGGAAGATAAAGGCGA
ZCCHC4_5	ACAGTACTCTTCTGAATCCG
ZCCHC8_1	AAGAATGAAAGCAACTCAGC
ZCCHC8_2	CCAAATGAAAGATTGCCCAA
ZCCHC8_3	GGGAGTGGAGGAGTGTCAGG
ZCCHC8_4	TTTCTTCTACTTCTTCTGCG
ZCCHC8_5	ACCTGAATCGAGCTCCATGT
ZCWPW1_1	ATTGGGTAGATACTACGGGC
ZCWPW1_2	GAACCAGGAAACTCACCGGC
ZCWPW1_3	GGCCAGGATAAGCCTGCCAA
ZCWPW1_4	GTGTCATAATGCCCTACCAG
ZCWPW1_5	AGTATCTACCCAATCAGACA
ZCWPW2_1	ATCAAGTGAGGATTCAGCCA
ZCWPW2_2	CAAGAAGCATGTCTACTCTA
ZCWPW2_3	GATGAATCAGAAGAAGGACA
ZCWPW2_4	TATGTAACTTATGACCCGGA
ZCWPW2_5	ATCTTGAATGGGGATCGCCC
ZDBF2_1	AGGAAGAATGATGAACCCAG
ZDBF2_2	GAAGTGAGCTGTGAACCGGA
ZDBF2_3	GATTCAGAAGATAAGAGCTG
ZDBF2_4	GGAGCATTACAAATCACTGG
ZDBF2_5	AAGTAATTGTCAAAGAAACA
ZEB1_1	AAGTTGGGTTCTGTATGCAA
ZEB1_2	CAGGGCACACCAGAAGCCAG
ZEB1_3	GCAGAAAGCAGGCGAACCCG
ZEB1_4	TGACTGTGAAGGTGTACCAG
ZEB1_5	AATGCTTCACCCATACAACA
ZEB2_1	AAGGCGAGACAGCTCCTCAG
ZEB2_2	AATGAAGCAGCCGATCATGG
ZEB2_3	AGACAAGCTTCATATTGCTG
ZEB2_4	GAAGGAAGACTATGACACTA
ZEB2_5	AAAATGGAGTGGATATGTTG
ZFAND3_1	AAGCCGCAGGGACAGCGAGG
ZFAND3_2	ACATTCGACCACATGGGCCG
ZFAND3_3	CTAAACAGAAGAGTCGACGT

ZFAND3_4	GCTGGGACTAAGAGTTGGCG
ZFAND3_5	ACTTACCCCCAGAAGCCGCA
ZFAT_1	GAAGAAACCCATCATAAGTG
ZFAT_2	GACGGCACAGACGTACACGA
ZFAT_3	TCTCCAACACGCGCCAGCTG
ZFAT_4	TTTGTAGCTCCTGTTTCAGG
ZFAT_5	AAACATCGTGAGTCCGACTG
ZFHX2_1	AAGATGCAGCTGCATGCCAG
ZFHX2_2	AATGTCCTAATGCTGCACCA
ZFHX2_3	GTTCTGGGCATCTTTCTCAG
ZFHX2_4	TCTGGGCCTGGGCATCGCGG
ZFHX2_5	ATCCCAAAGATGGCCCCATG
ZFHX3_1	AAACGAACGGACGTACCCAA
ZFHX3_2	CGTCTGCAACAAGTTCACGA
ZFHX3_3	GCTAAGGACCAAGAGGGCGG
ZFHX3_4	GCTGCAGAAGGGCCTTCCAG
ZFHX3_5	AGTAGTTACACACCTCGCAG
ZFHX4_1	ATAAAGCTCTGCTTCCGCCG
ZFHX4_2	TAAGCCTACAGCAGTGGCCG
ZFHX4_3	TACCAGATTCAACTTGACCT
ZFHX4_4	TAGGTGAACAGGGTTGCCAA
ZFHX4_5	ACAAACTTCACACCGCCAGG
ZFP1_1	ATGTATAGGATCCTCTGAGA
ZFP1_2	CAGAATATTCTACTCCACTG
ZFP1_3	GAAACCCAGACGAGCAGGCG
ZFP1_4	GTACAATGAGGTTTGACTGG
ZFP1_5	ATCGCCTCTTTCCTCAATTG
ZFP14_1	AAGGTCTGGACCACATACGA
ZFP14_2	ACATTAACTTACATGGGCCA
ZFP14_3	CAAGTGTAAGGAGTGTAAGA
ZFP14_4	GGGACAAGAAGATACTGCCC
ZFP14_5	AAGGTGTGTACACTGTCTAA
ZFP2_1	AAGATGCATGCTTTGACTGA
ZFP2_2	ACATACTCTCATCTCAGTGA
ZFP2_3	ACTGAGATGAGAGTATGTGG
ZFP2_4	AGTGTGAATGACCTGATGCA
ZFP2_5	AATCCTCTGGTGCTTAAGAA
ZFP28_1	AAACAAGAAGCTGGAGGCTG
ZFP28_2	ATGGGAGAACAACAGTGACC
ZFP28_3	CAAGGAGTTACCTCTCAAGA
ZFP28_4	TCTGCGATGACAAATAAGGG
ZFP28_5	ACATAGGGCTTAATCAACAC
ZFP3_1	AAGGTGTGAACTCTGAATGA
ZFP3_2	ACATCAGGGAGATACAACAG
ZFP3_3	GAACAAGGAGGTGATTCCCA
ZFP3_4	GATGGAAAGTCTCTCTCCTG
ZFP3_5	AGGAGAGAGACTTTCCATCA
ZFP30_1	AATGCCTGAACTCATATCAG

ZFP30_2	GAAGTTTGTGATGTAGTCGA
ZFP30_3	GAGGTGGGCACACTGTCTAA
ZFP30_4	TGTGGAAAGATCTTCACATG
ZFP30_5	AATGAACTTATCTCAGTGGA
ZFP36_1	AGGGTGACAGCTCAGGGCCC
ZFP36_2	CATGGCCAACCGTTACACCA
ZFP36_3	GGGCCGCCAGGTCTTCGCTA
ZFP36_4	GTAGATGGCAGTCAGATCCA
ZFP36_5	AGAGTTCCGTCTTGTATTTG
ZFP36L1_1	GAAGATGGTGGCAGACACGA
ZFP36L1_2	GATGGTGGCAGACACGAGGG
ZFP36L1_3	GGCTCACCTACCCTGCCCGA
ZFP36L1_4	GTCTCGCGAGCTCAGAGCGG
ZFP36L1_5	AAACGGTGCCTGTAAGTACG
ZFP36L2_1	GAAGTCGACATCGTAGAAGG
ZFP36L2_2	GACATGCTGCGCCTCCGCGG
ZFP36L2_3	GTAGAAGGCGGACAGAAGTG
ZFP36L2_4	GTTGCACCTGGGCTTCCCGC
ZFP36L2_5	CGCCGTTCTCGCTAAACGAG
ZFP37_1	AAGGAGAAGCACCATGGTTG
ZFP37_2	AAGGTTCTCAGCCATAAACA
ZFP37_3	GATGGCTGTGTCCGAGCCCG
ZFP37_4	TCGGACACAGCCATCTCCAG
ZFP37_5	AAGGGGAAAAGACCCAGTCA
ZFP41_1	GACGTCAGAGCTGTGCCGGA
ZFP41_2	GAGACAGGAGCTGTAGGCAA
ZFP41_3	GGCACAGTGGGCCTCTCAGG
ZFP41_4	TGCGGGAAAGCCTTTAACTG
ZFP41_5	CACGTCTGCCTCCTCCCTTG
ZFP42_1	AATGAGGAGATGCTTTCTCA
ZFP42_2	ACAGGCAAGAAGCTTCCGCC
ZFP42_3	GCAAAGACAAGACACCAGAA
ZFP42_4	GGAAATAGAACCTGTCAGCG
ZFP42_5	GTATTCCAAAGAACATTCAA
ZFP57_1	AGTTGGGCGGCTCTGTGAGG
ZFP57_2	GAAGAGAGATTGCTGGAGGG
ZFP57_3	GGTAACCCAGATGGACGCGG
ZFP57_4	TGTACAGAAGACGCTCCCAT
ZFP57_5	CAGGTGTAGCAAAAAAAGGG
ZFP62_1	AAGGCCAGAGCTGTTACTGA
ZFP62_2	AATCACAAAGGAATCCACCT
ZFP62_3	CCACAAACGGATCCACACTG
ZFP62_4	GAGCCCAGAGCTGTTCCTGA
ZFP62_5	ACTATGCCTATAGGACAGAG
ZFP64_1	AGAATGGATACCTACCCGTG
ZFP64_2	ATACAAACTGGACCGTGCTG
ZFP64_3	GAGGCTGCTGCTGTCGGCAG
ZFP64_4	GTTGAACGTCCTCTCTCCAA

ZFP64_5	ACAAGCTGAAAACTCACATG
ZFP69_1	GAAGCTGCAACATCCAAAGA
ZFP69_2	GACATCATGAAATACACAGG
ZFP69_3	GCAACATCCAAAGAAGGCCG
ZFP69_4	TCACCAGAATCTATACCGAG
ZFP69_5	AATATGATACACCTACAAAG
ZFP82_1	AAATGAGACCCTTAAGGCCA
ZFP82_2	AAGAGCCTTGGAAAGTTGTG
ZFP82_3	GAGTTGTGAAAGTAGTCTGA
ZFP82_4	GGACAGGAGAGACCTCAAGA
ZFP82_5	AGAAGCTTAATAGTGCTGAC
ZFP90_1	ACCATGTCGACCCTGCTCAG
ZFP90_2	AGAAGAGCCATGGATATCAG
ZFP90_3	ATATCAAGTTTCCAAGCCAG
ZFP90_4	GTAAGAGATCATGTGTGCAG
ZFP90_5	CACATCCCTCACTCAACACG
ZFP91_1	CCTCCGGCGGGACACAGCTG
ZFP91_2	GATACTCGGCCTTCCTCCGG
ZFP91_3	GCTGCATCTAGACCTAGCCG
ZFP91_4	TAACAGTAGGCACTCTCCAG
ZFP91_5	AGGCCGAGTATCCCCGCCGG
ZFP92_1	AAGCACCGCATCATCCACAG
ZFP92_2	ACCTATGTGCAATCCTGCGG
ZFP92_3	GAGGCCCGAACGGCCACGGA
ZFP92_4	TCTCCTGACCACGAGACCCA
ZFP92_5	ACAAAAGGTCCTCTACAAGC
ZFPM1_1	GCGCCGGAGACATGTCCAGG
ZFPM1_2	GGAACCAGAACCCAGGCCCA
ZFPM1_3	GGCCCGACTCAGCCTCGCCA
ZFPM1_4	GTTGGTGGGTGCCAGCCACA
ZFPM1_5	AAGGACAGGAACCAGAACCC
ZFPM2_1	GAAACAGACATCATCTCCAA
ZFPM2_2	GAGTCGACAGCAACTTCCAG
ZFPM2_3	GGCGAAAGCAAAGCAAACCC
ZFPM2_4	GTTGAGACAGACGACTGGGA
ZFPM2_5	AAGGGCTGTAAATGACGGGA
ZFX_1	AGATGCCAATGGGATCCCTG
ZFX_2	AGTGGATGGCACTTGCCCTG
ZFX_3	CATTTGCTGCTCGTGCACGG
ZFX_4	TGTAGAAGTATTGTTAGGGC
ZFX_5	GCTGGCAAAATAGAACACGA
ZFY_1	GAAACTATGTCACTCGTCAG
ZFY_2	TAAAGGTTTCCGACACCCGT
ZFY_3	TGAAGATGTTGTCATAGAGG
ZFY_4	TGCATCGATGGTCACTCCAG
ZFY_5	CAGTCAAAGGATGACCATCA
ZGLP1_1	AAATGACTGAACCTCAGGTG
ZGLP1_2	AGTGGACCCTGGGTTCGAGG

ZGLP1_3	GAAATGACTGAACCTCAGGT
ZGLP1_4	TCAGCAGGCAGCGAGGCCCT
ZGLP1_5	ATTCCAAGGACACACAGACC
ZGPAT_1	GATGGGCTATGAGTTTGGCA
ZGPAT_2	GGTGACTCCAGCTATGCCAG
ZGPAT_3	TCTGGAGTATCACAACGCCA
ZGPAT_4	TGTCTGGCCAAGCACCAGGA
ZGPAT_5	AGTGGTGGGGTCAGATGCTG
ZHX1_1	AATCTGGTGCACCTAAGTCA
ZHX1_2	AGAGGGAGACCCAAAGGACG
ZHX1_3	GAAGAGGAAGACCGCGTGGG
ZHX1_4	GAGAGGGAGACCCAAAGGAC
ZHX1_5	ACCACGGAATCCCCAACTGT
ZHX2_1	AGTAGAACAAGATGTGCCCG
ZHX2_2	GAAGGTGCCCAAGAAGCCCG
ZHX2_3	GCAGTACCAGCACCAGCCCA
ZHX2_4	TCACTGGCTGGTGTGAGCGG
ZHX2_5	CACCGATATCGGTGTCAAAG
ZHX3_1	GAGCTCACAGCAGTTCACAA
ZHX3_2	GATGGCTCTACACTGGCCAA
ZHX3_3	GATGGGTGACACCTATTCAG
ZHX3_4	TTTGGGAAGGGCCTTGGCCG
ZHX3_5	CAATGCCACATGTCACTCCG
ZIC1_1	AATTGGAAGAGAGCGCACTG
ZIC1_2	ACACGGCCACACGGACGCCG
ZIC1_3	GATGAGGCTCGGCTTCTCGG
ZIC1_4	GGTCCACGAATCCTCCTCGC
ZIC1_5	CCCCGGCCGATGCAGCAAAG
ZIC2_1	ATAGCCGGAGCTGGCGGCCG
ZIC2_2	GAAGGCTCCCATGTGCGCGG
ZIC2_3	GCAGAGATAGGGCTTATCGG
ZIC2_4	TATGAACATGGCAGCAGCCG
ZIC2_5	AGCACATTCTGCGAGCCGTG
ZIC3_1	ACTAGCACCAGCCACAACCC
ZIC3_2	GTAGTTAGGAAACTGAGCGC
ZIC3_3	TGAAAGACTTGCCCTCCCGG
ZIC3_4	TTTGCAGAAGCTATAGCGGG
ZIC3_5	CATGAACATGGGAGTGAACG
ZIC4_1	GGAAGAAAGCGCCAGGACCG
ZIC4_2	GTGGAGGCCCGGGAACACCG
ZIC4_3	TAATCGTTTCCTCATCACCA
ZIC4_4	TCGTAGCCAGAGCTGGGCGG
ZIC4_5	CACACCCCGGGAAAGGACAA
ZIC5_1	AAATCCGCTAATCTCAGCGC
ZIC5_2	ACACTCACCCAAGCTCCCTG
ZIC5_3	ATTGAGGTTGGTCACCTGAG
ZIC5_4	TGGCGGAAAGGTCCCTCCGG
ZIC5_5	ATGAACATGCCGGCCGAGTG

ZIK1_1	AACACATATGGACCTCACAA
ZIK1_2	AAGTGAGTACTTGCCACGGA
ZIK1_3	GAAGCCGCAACATGGCTGGA
ZIK1_4	GTGCAAAGAAATCCTTGAAG
ZIK1_5	ACAGGATTGAGTCTTCTGTG
ZIM2_1	AGAGGAGGAATCATATGCAA
ZIM2_2	GATCCATTGGAACCACACCA
ZIM2_3	GGAGAAGGCATACTTACCCA
ZIM2_4	GTCCACAAGCACATCCTCGA
ZIM2_5	CTCACCTGGGACCCAGCAGA
ZIM3_1	AAGGTATGACCTGTCAGCGA
ZIM3_2	ACAGGAAATGAACAATTCCC
ZIM3_3	CACGCCTTCAAAGTCACCTG
ZIM3_4	GATACATCATCTATGCCCAG
ZIM3_5	CCGAACAGAGAAACTTGTAC
ZKSCAN1_1	GATGTCAGAATCTGGCTCGG
ZKSCAN1_2	GCGGCCAGAAATAAACACCA
ZKSCAN1_3	TAGTCCCAGAGACCAGGCGA
ZKSCAN1_4	TGGACCTGAGATGCTCGCAA
ZKSCAN1_5	ATACCGCCCCGATAGTGGAG
ZKSCAN2_1	AGGGAAGGAATCCTTACCCA
ZKSCAN2_2	GAAGCCAGAAATGCGTTCCA
ZKSCAN2_3	TCAGCAAGGGCAGGAACCCA
ZKSCAN2_4	TGAACCGAAAGCGAGAACGT
ZKSCAN2_5	AGATGCCCGAGAGTTAATCA
ZKSCAN3_1	ATTAAGTGAAAGCACAGCCC
ZKSCAN3_2	CAGGGACTTGCCTCCAGCTG
ZKSCAN3_3	GGAGCTTCTGGTCATAAAGG
ZKSCAN3_4	TAGGTTTCAGGTGTTGACCA
ZKSCAN3_5	CACAGCAGGATTCATCTCAG
ZKSCAN4_1	AGTGTGAGAGAAGTTTCACA
ZKSCAN4_2	GCACTATTGACACAAACTCA
ZKSCAN4_3	TAGCTTCCAGGCTCACTCCA
ZKSCAN4_4	TAGTGTCTCAGTCTTACCAA
ZKSCAN4_5	AGGTTCCCGTTGGTGACCAG
ZKSCAN5_1	AGGGCTGGAACTCTTCAGGC
ZKSCAN5_2	GAGGTCTGAGCTATAACCAA
ZKSCAN5_3	GATGACAGGAAGGAGAACTA
ZKSCAN5_4	TATGAGCTCCATATTGTCCC
ZKSCAN5_5	CAGGCACTTCCAGTACCATG
ZKSCAN7_1	AACTGACCAAGCCAGACCTG
ZKSCAN7_2	CATGCCAGAGGTGCACACCA
ZKSCAN7_3	GAGTCGGGAACTCTGAGTAA
ZKSCAN7_4	GATTTCCAGAGACACCTCAG
ZKSCAN7_5	AACAGAATCATTACCCAAGG
ZKSCAN8_1	ACAGCTACACTTCTCACAGC
ZKSCAN8_2	ACATAGGGTACTCTGGGAGG
ZKSCAN8_3	TCAGCTAGAGAACGGAGAGG

ZKSCAN8_4	TTTCAGAAACATGTTCTCCC
ZKSCAN8_5	AGGTAGAGGAGGATCATGGT
ZMAT3_1	ACAGCGGTACAGGAATGAGA
ZMAT3_2	AGGCCGAGTGATCCTGGCCA
ZMAT3_3	GAAGCACGGCGTGTTGCAAG
ZMAT3_4	TGACACACATGTCTCACCTG
ZMAT3_5	ACATTGCTCATTCTAGCAGG
ZMIZ1_1	ATGAACCAGCCCGGGCCGCG
ZMIZ1_2	CAGCCAGCAGTCTGTAGCCG
ZMIZ1_3	CATGAACCAGCCCGGGCCGC
ZMIZ1_4	GCACCAGTCCAGCAGCTCCG
ZMIZ1_5	ACACCTACCGGCAGACTTCG
ZMIZ2_1	CAGGTATGCAGGCGGCCCGG
ZMIZ2_2	GAGGGAACTTGCTTACCCGG
ZMIZ2_3	GCCAGTAGGGTTCATGCCAG
ZMIZ2_4	TGAGTGGCGCTTTGTTGCCA
ZMIZ2_5	CAGGTAGAGTGGCTTGTGCG
ZMYM1_1	AAGAGGTAGTGTCTTCCAGC
ZMYM1_2	ACAGGAAACCTGAATAGCTG
ZMYM1_3	AGGCAGTGGCATCACAGCTG
ZMYM1_4	TAGGCAACAGTCCAGAACTC
ZMYM1_5	TTGAGACTACGAGTGATTTG
ZMYM2_1	GCTGCTGTTGGACAGACGGC
ZMYM2_2	TAAGTTTCAGAACTCGTCAG
ZMYM2_3	TAGCCATCCAAGCTTCCTGA
ZMYM2_4	TGTACTACAGAATTCCTGGA
ZMYM2_5	AACTTGGGAGATGTCTCTAA
ZMYM3_1	AGGACCGTGGTCCACCTCCG
ZMYM3_2	AGGAGATGGCCTGACTGCGA
ZMYM3_3	GACATGCGAGGAGAGCGCCG
ZMYM3_4	TAGGGAGATCTGGAACACCA
ZMYM3_5	CTTGAGCGTAGGGACATGCG
ZMYM4_1	AAAGCAATGGCACCACAGCA
ZMYM4_2	GGTTCCACAGTGTCAGCCGG
ZMYM4_3	GTGAGTCCTTGAAATGGCGA
ZMYM4_4	TGAAAGCCAGTAGTCAACTG
ZMYM4_5	ACTTGCCAATGATACTACAT
ZMYM5_1	GATGCCAAATCTCTTGAAGA
ZMYM5_2	GTCCATGAGACTAGTTGCCA
ZMYM5_3	TAGATCTAGGAACTCACCAG
ZMYM5_4	TGCTTACGAAGTAAGGCCAC
ZMYM5_5	AATTTGTAGTAAATTAGCAG
ZMYND11_1	AAATCTAAGAATGAGGACCG
ZMYND11_2	AGCTAAGCTCAGCTGACGGG
ZMYND11_3	GAGGCTGGTGCACTCAGCTG
ZMYND11_4	TTAGAGACAGCAGTAGTCCC
ZMYND11_5	ATAAACACCCGATGTACAGG
ZMYND8_1	CAGGTAGGATAACTGTTCAA

ZMYND8_2	CCTGCGGTACAACATCAACA
ZMYND8_3	GGAATACATCTTCCATCCAA
ZMYND8_4	TGTTAACAATGCATCCACAG
ZMYND8_5	AGATGTATTCCGCATAGTCA
ZNF10_1	AAGGTGAGAGAACCAGCTGA
ZNF10_2	ATAGGGCCTCACTCTCACAT
ZNF10_3	ATTTGTGGACTTCACCAGGG
ZNF10_4	TGTATTTGTGGACTTCACCA
ZNF100_1	TAATGTCCTGCTCTGCCCAA
ZNF100_2	TTCTCCCGTAGGATGACCCG
ZNF100_3	CCTCACACCTTACTACACAC
ZNF100_4	GAATATAAAGAGACATGAGA
ZNF101_1	AATGTCCTTCTTACCGACCG
ZNF101_2	GAGGGAACTGCACCACCCAA
ZNF101_3	GGTGTGGTAAAGCCTTTGGG
ZNF101_4	TGTCCAGGAATGAATGACGG
ZNF107_1	AACATGTGGATGAGTGTACG
ZNF107_2	ACACAAAAGATACTTCACAC
ZNF107_3	ACTCAAACCTAATTAACCAT
ZNF107_4	CTTATGCCTAGTAAGTTGTG
ZNF112_1	TCAGGAGTCAATTCAAACAG
ZNF112_2	TCCTGCCCAAACCTGGCCGA
ZNF112_3	TTCCTGCCCAAACCTGGCCG
ZNF112_4	TTGTTGCCAGGTCTGACGGG
ZNF112_5	AAGTTCATAAGTGTTAAGAG
ZNF114_1	ACGTGATGAAATTGATACGG
ZNF114_2	GGAATTTGGAGCGTTGGCAG
ZNF114_3	GTTCACAGCCACGTCTGCGA
ZNF114_4	TTAGGAAGAATATCCGGCTG
ZNF117_1	CAGCACAAAGGAGATTATAG
ZNF117_2	GTTGAGTGTAAACAGCACAA
ZNF117_3	ACATAATATAATTCATACTG
ZNF117_4	ACATTCATAACGTTTCTCTC
ZNF12_1	AAGGGCTGAATTCTGGCAGA
ZNF12_2	AAGTGCTGACTTCTGCGAGA
ZNF12_3	ATGCCAGAAAGCCTTCCAAA
ZNF12_4	GAGGTCCGACATCTGGAGAA
ZNF12_5	AATAGCCTTTCTCCAGATGT
ZNF121_1	AAGGCATGAAGAGCTTCTGA
ZNF121_2	AAGGCCTGAGCGCCCAGCGA
ZNF121_3	AATGTGGAAGAGCCTTCGCT
ZNF121_4	GAGTTGTGAAGATGTAGCAA
ZNF121_5	AAGATGTGACTGATCAACAA
ZNF124_1	AAGGCAATTGGAGTAACGGA
ZNF124_2	AAGGGAACTGGCGTATCTGA
ZNF124_3	GAGTCCTTTCATGGTCACGA
ZNF124_4	TTAGTCGTGCTAGTACCCTT
ZNF124_5	AAACAACCCATATGGGTGTG

ZNF131_1	ACGGGTCAATCAAATGCCAG
ZNF131_2	ATTGAGGTAGAGATTGCCGA
ZNF131_3	GAGCAGCCCGACGGCCATGG
ZNF131_4	GATGTTCAGGGAACTCCTGA
ZNF132_1	ACAGGAGTTAGCTTTCTTGG
ZNF132_2	AGAAAGTACATACTGGGCAA
ZNF132_3	GAGGGTGGAGCTATTACTGA
ZNF132_4	TTAGTCACTCATCTAAGCTG
ZNF133_1	ACACCAGAAGGCACACTCGG
ZNF133_2	AGAAGGTAACATCCAGCCTG
ZNF133_3	AGAGAGTATAAGGAATCCGA
ZNF133_4	GCACAAGCGGAAGCACTCGA
ZNF134_1	ACAGTAAGAGGAAGACACAC
ZNF134_2	GAAGCCCTTTACGTGTAAGG
ZNF134_3	GAGGGTGTATTTGTGGCCAA
ZNF134_4	GTATAGAGCAACCCTTAAGA
ZNF134_5	ACTGAAACTTCACACATGTG
ZNF135_1	AAATGGAGTTAGGCTCGCGC
ZNF135_2	AGTACCACAGACCATCCCAC
ZNF135_3	GAGGTGTGTGCTCTGCCGGA
ZNF135_4	TGACTGCGAGACAAAGGCCG
ZNF136_1	AAAGATCACTACAAACACCG
ZNF136_3	AAGGATGGTAGTCAGCGTGG
ZNF136_4	AGACACATCAAAGATCACAG
ZNF136_5	AAGGAGTGGTGAGAACTGAA
ZNF138_1	ATGGTGGTAGCCAAACATTC
ZNF138_2	AAAGTGACACTGAGCAGATA
ZNF138_3	AGTGCCTGGACACTGCACAG
ZNF138_4	GATGAGTGTAAGGGACACCA
ZNF14_1	AAGATGACCACAGAAACCAG
ZNF14_2	AATTTGACTGGAACGAAGGA
ZNF14_3	AGTAGAAGAGGTAGCAAATG
ZNF14_4	ATCGAATTTGACTGGAACGA
ZNF140_1	AAGGGAAAGAACCCTGGCTG
ZNF140_2	AGTCCTCTGGTGTAACACCA
ZNF140_3	TATGCAACAAATCCTTCAGC
ZNF140_4	TGAATATCAGTACTGTGGAG
ZNF141_1	CAGGACCCTGGACCGTCCAA
ZNF141_2	CTTATGTTGACTCCGATCTG
ZNF141_3	TTTATGTTCATTCAGGACCC
ZNF141_4	AAGATCGTAGCCAGACCCCC
ZNF142_1	ACAGCCAGCCAGTAGCACCG
ZNF142_2	AGAGAAGAGTGACACCCAGA
ZNF142_3	AGGAAACTCCTTGCCCACAG
ZNF142_4	CAGGAGGGACTGGGTCAAGG
ZNF142_5	AATGTGGCTATGTCACCAAG
ZNF143_1	AGATGGCCAGGTCATTCAGT
ZNF143_2	CAAGCAGTAACACTTGCAGA

ZNF143_3	GCGGGACTTGCACTGCATGG
ZNF143_4	GGTGTGGTGAATAAATGCTG
ZNF143_5	ACTGCTAAATCTCAACAGAG
ZNF146_1	AAGGTTGGATTTGCCACTGA
ZNF146_2	AAGTGATGTTCGCTGAGAGA
ZNF146_3	GAGGTACTTCTTCTGGCCAA
ZNF146_4	GTATTCTCAAATGCAGAGCA
ZNF146_5	CACATACCTTACAGGCAAAG
ZNF148_1	AATGCAGTCTTCCAGGACAA
ZNF148_2	AATGGACAAAGAGAGTGCTT
ZNF148_3	GACTTCTCAGAAGAAAGCTG
ZNF148_4	GTAACACTTCATCTGCAGCA
ZNF148_5	AGATCGAAGTATGCCTCACC
ZNF154_1	ATTCCGGCCTCATTAAGCAC
ZNF154_2	GAGACTAGAGCTTTGCCTAA
ZNF154_3	GCACAGGAGGGTTCACACTG
ZNF154_4	TCAACAGGCTGCTCACACTG
ZNF154_5	CTGCACATTCCATGTGTCAG
ZNF155_1	ACTGTTTACACTTCCCACCC
ZNF155_2	GGACAGAAACCTTCCCAGGG
ZNF155_3	TATGATAGAGTCCTGAGACC
ZNF155_4	TTACACTTCCCACCCTGGGA
ZNF157_1	AAGGTCATTATCATGCCTGA
ZNF157_2	ACAGACTGGACCCTGCTCAG
ZNF157_3	AGAGCTGTGGATATTAGAGG
ZNF157_4	GAGGAGGAATCCTCAGGCCA
ZNF16_1	GCGTCAAATAGTCCACACTG
ZNF16_2	TATGAGTGGACTTTCAGCTG
ZNF16_3	TCAGCCAGAGTGCACACCTG
ZNF16_4	TCAGCCCTCATTCAGCACCA
ZNF160_1	AGAAGCAGTATTCCACACAG
ZNF160_2	GAACTCTCCGATGCCTTCCG
ZNF160_3	GGTACAATTCATACCTCGGA
ZNF160_4	GTGCTTATGGCCCAGAAAGA
ZNF160_5	AGAACGCCGGAATGTGTCAA
ZNF165_1	GAGAAGTGAACTCCTTAAGC
ZNF165_2	GGTGAAATAATAAGCCACGA
ZNF165_3	GTAAGCAAGTGTCCTGCCCA
ZNF165_4	TAATGTCAAGTTACAGCCAG
ZNF169_1	ACTCCTGACAACCAGGAAGG
ZNF169_2	AGGAGGAACAGACCTTCGCC
ZNF169_3	GCACCAGAGCTCACACACAG
ZNF169_4	TCACAAGAGGATTCACTCCG
ZNF169_5	AGCTATTGAGTTCTGCTCAG
ZNF17_1	ACACTGTCATTCACAAACGA
ZNF17_2	AGAGGATTCACACCAGGCCA
ZNF17_3	CAACAACAGGCTCTTCACAG
ZNF17_4	GGTGTGGAGGCCTTTCAAAG

ZNF174_1	AGGTGGCCGTTTGTATGCAG
ZNF174_2	CATCCAAGAAACCAAAGCAG
ZNF174_3	GCAACCCGAGCTGCACACCA
ZNF174_4	GTGAACCTCGGTTGTCACGG
ZNF174_5	CCCAGGCAGGAGCTTATGAC
ZNF175_1	CTATAGCCATCTCTTCGCAG
ZNF175_2	GAAGAAAGCACTGTGACTCA
ZNF175_3	GTATCACATTCCCAACCCAG
ZNF175_4	TCTGAAGATGACCTCTGGGT
ZNF177_1	ACACAGTCTGATCTCCAAGG
ZNF177_2	ATGCACAAGCGAATCCACAA
ZNF177_3	CAGGCAAGAGGAATTCCTAA
ZNF177_4	TTAGGAATTCCTCTTGCCTG
ZNF18_1	AGATTACTCACCTATGCAGG
ZNF18_2	CCTGAGGCACCACTTCAAGA
ZNF18_3	GCCATCGCTGGCGAAGGCCG
ZNF18_4	TTTGTACGTCAGCTCACCTG
ZNF18_5	AATCTCCAAGCTGCCAAGTG
ZNF180_1	AAAGTCGAGGGAGAAGACAC
ZNF180_2	AAGGACATAACTCTGGCTAA
ZNF180_3	ACGCGGGAAGCTGGAAGCGG
ZNF180_4	TGTCCGATGCGCGCATGCGC
ZNF180_5	ACTTTGATGATAATCTCTTG
ZNF181_1	GATGGCTCAGTTCTGCTCAG
ZNF181_2	TGTAAACACTGTCTGCAGTG
ZNF181_4	ATATGTGATCACGTTATTGG
ZNF181_5	GGTTAGTAAGTGATGAGACA
ZNF182_1	AATCCTGGAATGAAGCCCTA
ZNF182_2	AGTACCTGAACCCACCACAG
ZNF182_3	CATGCAGGAAAGAAAGCCCA
ZNF182_4	TCCGAGTGTGGCCTCTTCCA
ZNF182_5	AAGTTGATGAACAGATTGAG
ZNF184_1	AAACCAACAGACACTGCCAA
ZNF184_2	GAATGGAAACAGCTGGACCC
ZNF184_3	GAGAGCAGAGCGATATCTGA
ZNF184_4	TGAAGAAGAGCTATCTCCAG
ZNF189_1	ACAGCAGGAAGTCTACCCTA
ZNF189_2	ACTTACCTTCGACTCCGGCG
ZNF189_3	GTATGGATAAGTCTTCTCCC
ZNF189_4	TTACTTACCTTCGACTCCGG
ZNF189_5	AAGGTATGTGCTTAATCGAA
ZNF19_1	TAGCTGGGAAGAAGTCCCAA
ZNF19_2	TCTGGGAAGTCAGTTCTCTG
ZNF19_3	TGAAAGGTTTCCTTGCACAG
ZNF19_4	TGAGTGTAGCAAGTATGAGA
ZNF195_1	AATGTGACAGCATCTTCAAG
ZNF195_2	GAATCCTCCTGTGTTCACTG
ZNF195_3	GCAGCAGACGGACATCCAGG

ZNF195_4	GTTTGGAAGGATTTGGGCTG
ZNF197_1	AAGATAAGTGGTCTGAGCAA
ZNF197_2	ACTGAGTAAATACCATGCTG
ZNF197_3	GAGACCAGAAGCACGCACCA
ZNF197_4	GGAGTTGGTGATGTTCGAGG
ZNF2_1	ACAGAGGGACCTCTACAAGG
ZNF2_2	CCCGGGACAAAGGCTTGCGG
ZNF2_3	GACATTCGAAGACGTTGCCG
ZNF2_4	TAGGGACGAACGGTCAAAGA
ZNF20_1	AAGGGTAGTGGAACGAGTAA
ZNF20_2	AAGGTGTGAGCCACATCTAA
ZNF20_3	CACACTGAGGATAAACCCTA
ZNF20_4	TAAAGCCTTCAGATGTACCT
ZNF20_5	AAAGGAGTCAAGATAACTGA
ZNF200_1	AGTGCTCCAGCACTAGCTGG
ZNF200_2	GAAATCGGAGGACAAGGAGA
ZNF200_3	TTGGTGAAGCTTCTGCCCAA
ZNF200_4	TTTGGGCAGAAGCTTCACCA
ZNF200_5	AGAAGCTTCACCAAGGGACG
ZNF202_1	AGTGGCTGAGACCAGAGAGG
ZNF202_2	ATTGCGGAAAGCACTTCCGC
ZNF202_3	TTCCTCAGGAGACTGCTCGG
ZNF202_4	TTGGGTCCAGATCACTCCAC
ZNF202_5	AGGGTTTGCAGAAACAACCC
ZNF205_1	CACGCAGCAGAACTTCTACA
ZNF205_2	CGAAGAGCCACACTCCGAGG
ZNF205_3	GAGGACCAGAGACCGGCAGA
ZNF205_4	GAGTGGAGAGGCGCGTGCAC
ZNF205_5	GAGGGTGCAGACCTCATCCG
ZNF207_1	GACAAAGGATACCAGCACCA
ZNF207_2	GGAACACCTGGCATTAATGG
ZNF207_3	GGAGGAATATAACCTTGCTG
ZNF207_4	TGGAAGAACAGACATAGAGT
ZNF207_5	ATGCAATGAATAGCTAAGCC
ZNF208_1	AAAGACTGACAACCAGCTGA
ZNF208_3	GAGTTGCCTTATGTTCAGTA
ZNF208_4	TGTGATAAGGCAACTCATGC
ZNF208_5	AAGGGTTGAGACCTTACTGA
ZNF211_1	GAACACCAAGGAACAAACTG
ZNF211_2	TAACAGTAACAAGTGTGCGG
ZNF211_3	TCAGCAAGATGGGATCAGAG
ZNF211_4	TCTTTGGAAGTCCTGAACTG
ZNF212_1	AGCACCAGAAGATCCACCAG
ZNF212_2	GAATCTGGAGGATTGGCAGA
ZNF212_3	GAGGTCCTTGGCCAGACAGA
ZNF212_4	TGACTGCGAGAAGATGGCCG
ZNF212_5	ACAGGAGCTACAGTATACAC
ZNF213_1	ACAGGATGTGCCCTCGGAGG

ZNF213_2	GAAAGTGGAAGATTCCTCCT
ZNF213_3	GAACTCCCGGAACACCACCC
ZNF213_4	GGAGAGCGAGAACCGGCCGA
ZNF213_5	ATGGACCCCAGAGACAAAGC
ZNF214_1	AGACATCCAAGAAACCACAT
ZNF214_2	GAATTCGAAGATTTGAGCTC
ZNF214_4	TACTCTCCACACAAGTACCA
ZNF214_5	CATATCACCCCAACATACTG
ZNF215_1	AAGTCCTGACTTCTTCAGGC
ZNF215_2	AATTCAGCAAGGAAGAGTGG
ZNF215_3	ATAGCCATCAACTTGCTCAG
ZNF215_4	TCTGGAGAAGAATCATCCCA
ZNF217_1	CAGAAATAGTCTCTTCCCGG
ZNF217_2	GAGAAGTGCAAACACTCCCA
ZNF217_3	TCTGAGGATGGGCTTCCCGA
ZNF217_4	TGGGTGGTACTGCCATCCGG
ZNF217_5	CAAAATCTCACCCTGAAACG
ZNF219_1	AACGGGCCAGCCGTGAGCGC
ZNF219_2	ACGGGCAGAAGAGGCAGCGG
ZNF219_3	GAAAGGACAATCCTTGCCGG
ZNF219_4	TCTGGCCGCACACTTGGCAG
ZNF219_5	AGGCAAGTTTCGCACCTCGG
ZNF22_1	ATTTCTCGGAGCTCAAGCCA
ZNF22_2	GCATCGACGGATCCATACGG
ZNF22_3	TCAGTCAGAGCTCTCATCTG
ZNF22_4	TCGGAGCTCAAGCCAAGGAA
ZNF22_5	AGAACAAGCGCAAAACAGGC
ZNF221_1	AGCCATTGAAATCTGGAGTG
ZNF221_2	GAACTGAGAGCTGTTCCTTA
ZNF221_3	GAATGAAGCAGTTCAAGTGA
ZNF221_4	TACTTTCCACTTCTTAGGGA
ZNF222_1	AGTCTTGCTTTAATCTGGGA
ZNF222_2	CTTCCACCATAGAACCCACA
ZNF222_3	TCAATGGCTTTCTTTGGCAG
ZNF222_4	ACAAGAGAAAAACCTTTCCA
ZNF223_1	AAAGAAACCAAACAGCACTG
ZNF223_2	ACAGGGTGATGCCCACTCCC
ZNF223_3	AGTCCTACTTACCTTGGACA
ZNF223_4	TCTGCTGAGCGTATTTGCTG
ZNF224_1	AAGACAGCAATCCAAAGGGA
ZNF224_2	ATGGTTTATATCCATTGCCC
ZNF224_3	TCACACGTATCACATCGGAA
ZNF224_5	ACAAGACAGAAATCTTCCCA
ZNF225_1	AAGGACGGAGACATCACTAA
ZNF225_2	AGACATGTGCGAGTCCACAG
ZNF225_3	GTAAGACAGAAACCTTCTGA
ZNF225_4	TCTCACCAGAGAGCGCACAG
ZNF226_1	AAAGCTGGAGAGATCACTGA

ZNF226_2	AAGAAGGGTGTTGATCCCAT
ZNF226_3	ACGACAGCAACCCGAAGACA
ZNF226_4	ATGATGTGAAATCCAACCGA
ZNF227_1	AATTAACCAGGTGTCTTCAG
ZNF227_2	ATGTGGTAAGGGCTTCACTC
ZNF227_3	CAAGTGTGAAACGTGTGGGA
ZNF227_4	TAGATGCGACAGTTGCGGCA
ZNF229_1	AAACACCAGCACATTCACCC
ZNF229_2	GGAGAAGATCATGTCTCAGG
ZNF229_3	GGAGGAGAAGATCATGTCTC
ZNF229_4	GTTCAGTTCCAATTACCCAG
ZNF23_1	ACATCAAGAGAACAACACTG
ZNF23_2	GGGTAGAGAGGTCTTACCCA
ZNF23_3	GTGATCTCACAACTGGAGGG
ZNF23_4	TGGCATCAGAGACTTCACAG
ZNF23_5	CCTTATCAGTGTTCGGAGTG
ZNF230_1	CAGGTCTTAACTTGCACCAG
ZNF230_2	CCCTTAGGAAGTGGAAAGGG
ZNF230_3	GGACAGAAACCTTCACAGAA
ZNF230_4	TATGATGGAGTCTTGAGACT
ZNF232_1	ACAGGACCAGGAGGTTCCAT
ZNF232_2	GAGGCCAGAGAAACACACGA
ZNF232_3	GCAGCAGAGAAATCCCAAAG
ZNF232_4	TACCAGCTGGAATCTTGCAA
ZNF233_1	AACTCAACTCACCTGAAACT
ZNF233_2	ATGATGGAGACAGAAATCCA
ZNF233_3	GGGTGTTCAGGTGAGAACCA
ZNF233_4	AGGTCCAAGTTGCTAAAACA
ZNF233_5	CAACTTCAAGCCCATCAGAG
ZNF234_1	AAGATGGGAAGCATGTATGA
ZNF234_2	GAACATCAGAGAGTTGTCAG
ZNF234_3	GACATGAAGAGCTTTACTGG
ZNF234_4	GCATCTGGTAGTCCACACAG
ZNF235_1	AAGGAGCTTCAAACCCAAAG
ZNF235_2	TACGTGTCAGCAGTGTGGGA
ZNF235_3	TAGATGTGACAGTTGTGGGA
ZNF235_4	TATGATATCCCAGTTGGAGA
ZNF236_1	CGTGCAGCGAGTCCACTCAG
ZNF236_2	GTCCACATGCGCCTGCACAC
ZNF236_3	GTGCACATTCGCCTGCACAC
ZNF236_4	TCATAAATGCACCCACAGCG
ZNF236_5	ATCACGGAAGTCTCGTCCTG
ZNF239_1	ACTGCAAAGACATTCATGGA
ZNF239_2	AGTGTGATGACTCTTCAAAG
ZNF239_3	GGATGCGAAGATCCGAGCTC
ZNF239_4	GTTGCCTCACCATTTAACAA
ZNF239_5	TCATGGATGGAAATCACAGG
ZNF24_1	AAGTCTGTAGCTCTTTGGGT

ZNF24_2	AAGTTGGAGGAGGATCCTGA
ZNF24_3	GAGAGTGAACTTGATGACCC
ZNF24_4	TCTGAGAGTGAAGTTGGAGG
ZNF24_5	CCTGGGAGCTCCATTCCCTA
ZNF248_1	AAATCACTAACTCACCTGGG
ZNF248_2	CATCAAATAGTACACATGGG
ZNF248_3	GCACAGTGAGGGCTGATCTG
ZNF248_4	TCAGGAAGAGTGGTATCTGC
ZNF248_5	CATTTGGAAATGGAGCCGTA
ZNF25_1	ATACAGAGTCCTCTGAGCAG
ZNF25_2	GAAGTAGAATTTCCACATCG
ZNF25_3	TGGGAAGTTCTTCTATGTGA
ZNF25_4	TTATACAGAGTCCTCTGAGC
ZNF25_5	ACATCAGAGAATGCACACAG
ZNF250_1	CAGGTGTGAGTGCTGAACGA
ZNF250_2	TCAACCATAGCACTGTTCTG
ZNF250_3	TGAGTGCAATAGCTGCGGGA
ZNF250_4	TTAAGACCTGAACTTCACGG
ZNF250_5	CAATGAGACTACATTCCCAT
ZNF251_1	GAATTCTCCGATGAAGACGG
ZNF251_2	GCAGAATGAGATTTGCACCA
ZNF251_3	GCCGGAGTTGATCTCCCAGC
ZNF251_4	TCTGCCGTGCTTCTCTCCAG
ZNF251_5	AAGATGAGAGTTAAAACCAA
ZNF253_1	GAGGATTGAGGGCCAGGTAA
ZNF253_2	TATGTGTGGTAACGTGTGAG
ZNF253_3	TTTGTATGTTCTCTGGCCAA
ZNF253_4	CTTATGTGTAGTAAGGTCTG
ZNF254_1	GAGGTGCGAGGACCGATTAA
ZNF254_2	GTAAGGGTTGAGAGTTGCTT
ZNF254_3	CGCTACAAATGCTTAGAATG
ZNF254_4	CTTATGTCTAGTTAGTGTTG
ZNF256_1	AAACAGAACCAAGAGTGCAG
ZNF256_2	AGCAGAGCACTTCTCCACAG
ZNF256_3	CGCGGCACATACCTGGGCCG
ZNF256_4	GAACTCTCAAATGATCACTG
ZNF256_5	CTAACCTGTGACACCCGCTG
ZNF257_1	GAGTGTAAGGTGTGCAAAGG
ZNF257_2	GATGAGTGTAAGGTGTGCAA
ZNF257_3	GTTGAGAAAGGTGTGAAGAC
ZNF257_5	CTTATGTCGAGTAAGAGCTG
ZNF26_1	AAGCCCTGAATTCCAACAGA
ZNF26_2	GAGCTGTTCCTTCCTACCAA
ZNF26_3	GCCTGACTTAATCTTCAAGT
ZNF26_4	GTTTCCGGACAGCTTCGTGC
ZNF260_1	AAGTGATGTGATTCGAGAGA
ZNF260_2	GAGGTATTGCTTCTGGCTGA
ZNF260_3	GATTCTCATGTGCAGAGCAA

ZNF260_4	TAGAAGTCACACAGGAAAGA
ZNF260_5	GAGATTTGACTTGCCACTGA
ZNF263_1	CAGGAAGCAGCACCTGAGGG
ZNF263_2	CAGGTCACAAACCATGGGCG
ZNF263_3	TAGTAAGAGGGCCCTCTCCA
ZNF263_4	TCGGCCTGAGATGCGCACGA
ZNF263_5	ATGTTCTCAGAGCATACAGA
ZNF264_1	GAAAGCCTTTAGCAATCGGA
ZNF264_2	GCAGAGGAAGATGTTTCCTC
ZNF264_3	TTCCCAGGAGACTTCTCCTG
ZNF264_4	TTGTGAGCCAGCCTTGTCAG
ZNF266_1	AAGGCGAGAGGATCTGGCAA
ZNF266_2	CAGATAGGAAGCCACAACGG
ZNF266_3	GAATTCGAAAGTGATCACTG
ZNF266_4	GATAGGAAGCCACAACGGAG
ZNF266_5	AAGGTGTGAATGATTAATGA
ZNF267_1	CCGATGCACAGTAAGACCTG
ZNF267_2	GAGGAGTGTGAAGGGCACAA
ZNF267_3	GAGGTATGACCTATAGCTGA
ZNF267_4	GTTGAGTAAGGCAAGAACTA
ZNF268_1	CAGGAATCCATCTTGGGCCA
ZNF268_2	CATGCCGAAGAGAAACCCTA
ZNF268_3	GAATAGAGAAAGTCCTAGAG
ZNF268_4	GACCGTAGGGATGGCCACCA
ZNF273_1	GAGCATAAGGTGCACAAAAG
ZNF273_2	GCATGTCTCTTCATATTGCA
ZNF273_3	GCCACATCCCTAAATGTCAG
ZNF273_4	TTCTACAAATGTAAGACATG
ZNF274_1	ACAACCTCAATGTTCATTAG
ZNF274_2	GGACAAGGCACAATCCCTTG
ZNF274_3	GGACATGACAGGTCCCCGGG
ZNF274_4	TGTGGTATCTCAGTTAGAGG
ZNF274_5	GCTTCACAGGCAGTGCACCT
ZNF275_1	ACTGCGGCAAGCTCTTCCGA
ZNF275_2	GGAATGTGGCGACTGCGGGA
ZNF275_3	GGCGTCCACAAGCTTCCCAA
ZNF275_4	TGAGTGCGACAAATGCGGCA
ZNF276_1	CATGGAGAGGCCATCCGCAG
ZNF276_2	CCAGGGCCACGACTACACCA
ZNF276_3	GAGTTCAGTGACCTTTCTGA
ZNF276_4	GGCCGCCCTTCCCTTAGCGG
ZNF276_5	ACACTGTCCTCCGAGTACTG
ZNF277_1	ACAACTCTTACTCTTCCTGA
ZNF277_2	ACACTTACCAAATGGAGCAG
ZNF277_3	ATGGCTGCTTCCAAGACCCA
ZNF277_4	TCCCTGCCAGAAAGTCCAGG
ZNF277_5	AAAGGAACTTGGAAAATCGT
ZNF28_1	CAGGAGGTTATCCTCACCCA

ZNF28_2	CATCAATGTGAGATCTACGA
ZNF28_3	TACATCCCTCTTCCTTCACA
ZNF28_4	AAACATAGTATAATTCACAC
ZNF280C_1	AAGAAGAAGAGCTAGAGCCA
ZNF280C_2	AAGAGTGAGCCACACAGTCC
ZNF280C_3	ACTTGGGAGGATGTCACTGG
ZNF280C_4	AGACCCACCATCAAATCCAG
ZNF280C_5	GAAGTGGAGGGATTTATCTG
ZNF280D_1	AAGAATGGTGCACTCAGTCG
ZNF280D_2	GCTCCAGAAACACTTTCGCT
ZNF280D_3	GGATTATCACATTACCAAGG
ZNF280D_4	TTTGGACAAGCTCTTGGAAA
ZNF280D_5	ACAGGATTATCACATTACCA
ZNF281_1	CATCAAACAGGAGAAACCCG
ZNF281_2	GAATGAGGACATGTCTCCGC
ZNF281_3	GAGGATAACACGCATTGCGG
ZNF281_4	GTTGAACATAACCATACCCT
ZNF281_5	CCTCCACTGGAAGACACGGT
ZNF282_1	AATGGGACATGGACGCCCGG
ZNF282_2	CGAAGAGAGAGAAATCCCAA
ZNF282_3	CTGGTGGCAGACCTCCTCCG
ZNF282_4	GGAGGAAAGAGCCATCCCTA
ZNF282_5	CAGGGAAGAACCTTGTGTGT
ZNF283_1	AGATGGATGCCTCAAGGCCA
ZNF283_3	TCACCAATAGAGGAATCCCA
ZNF283_4	TGAGTAAGTTGTCCACAGAA
ZNF283_5	AAGTGCAAAAGCATATTCGA
ZNF284_1	AGACATCAGCGGGTCCACAA
ZNF284_2	AGACTTACTCACCTTGAACA
ZNF284_3	GAAACAACATTCAAGTGCGA
ZNF284_4	GAGAGACACCTTCTGAGCAT
ZNF285_1	AATGAAGCACTTCTTGCGAA
ZNF285_2	AGCATGGCAAAGCCTGACAC
ZNF285_3	GAATGTAAAGGAGAGGACCC
ZNF285_4	GATCACATGAGGTCCAACTG
ZNF286A_1	AAGGGATGAGCTTTCTGTGA
ZNF286A_2	ACTGAAGACAGAGTTCCCAA
ZNF286A_3	GGAAGCTGGATCCTGCACAA
ZNF286A_4	GGCCATGGACTTTACACCAG
ZNF286A_5	AATTGGTTAGAGAATCAGCA
ZNF286B_1	AATTGGTTAGAGAAGCAGCA
ZNF286B_2	GGTGTTGAATATGCACTGAA
ZNF286B_3	TCACCTGGGATCTGGCCGTG
ZNF286B_4	TTGTTGAGCACAGAAAACGA
ZNF286B_5	CGCGCTCTCCTCTCGCGCCG
ZNF287_1	GAGACCTGAGATTCACTCAA
ZNF287_2	GATGATAGACTAGAGAGGCG
ZNF287_3	GTATAGTAAGGTGTGCACGC

ZNF287_4	TCTAAGGTGGAAGTCAGACA
ZNF287_5	ACTACGATGATAGACTAGAG
ZNF292_1	CAGCTGCTGACAGTAGTCGG
ZNF292_2	GAGAGGTTGAGTTGCGGCGA
ZNF292_3	TGCCAAGCGTTCCAGAACCA
ZNF292_4	TTGCCCTTAGTCCAATCCCG
ZNF292_5	AAAAAGTTAAAAAAGACCGT
ZNF296_1	ACAGGGTCCACGGGTTCCGC
ZNF296_2	CCCGAAGGAGGTGTCCTCGG
ZNF296_3	CTGCCTGCAGACCTTCCCGT
ZNF296_4	GCTGCTCTGGGCGCAGGCGT
ZNF296_5	CTGTGGCAAACAGTTCACAG
ZNF3_1	AAGAGCCGAGCTGTACCTGA
ZNF3_2	AGCTGTGTACTTCATCCGGA
ZNF3_3	TTCCGACAAGGACAGCCTGG
ZNF3_5	GAGGAGAAGCTAACCCCCAG
ZNF30_1	AAGCTGTCCATACACAGTGA
ZNF30_2	CAGGCTTTGAGTCTTGACAA
ZNF30_3	GAATTCGCTGATGTGCATGA
ZNF30_4	GAGTCTGGACTCTTCCCAGA
ZNF300_1	GAATTCGCTGGTGTCCCGGA
ZNF300_2	GAGGTGGGACTTCTCACAGA
ZNF300_3	GATACTGAAAGGAGTCACAA
ZNF300_4	TCAAGACAAACTCTTCAGGC
ZNF302_1	AAAAGTGAGAGAATAAATGG
ZNF302_2	ATATGTGATCATGTTATTGG
ZNF302_3	GTCGTGTAAGGGATGAACCA
ZNF302_4	TTCACCCCAACCAGTAACAA
ZNF304_1	ACAGTAAAGGAGACCTACAG
ZNF304_2	ATAGGGTGAGTCCATGCAGA
ZNF304_3	CAGTAAAGGAGACCTACAGA
ZNF304_4	CATGAGATATTTCTCCACTG
ZNF304_5	GTGGGTCACACATCTCACAT
ZNF311_1	ATAGAGATGCCTTTGAGCGT
ZNF311_2	GCATCAGCTTATCCACACCG
ZNF311_3	TGTATCTCACCGCTTTCCTG
ZNF311_4	TTCACTAAAGATGGAAGCCA
ZNF317_1	TCGGTGACTATTTATCCCGG
ZNF317_2	TCTCTGAGAAGAATCCAGCA
ZNF317_3	TTGGACCTGTTCGTGTGCAG
ZNF317_4	TTTGGGAAGGAAACGCCCAG
ZNF317_5	ACGAACAGGTCCAAATTCTG
ZNF318_1	AAAGAGTCAGAACACAGACC
ZNF318_2	AGTGGTCAACTGAGGAACAG
ZNF318_3	GGGCCGACGACTCTTCCCGC
ZNF318_4	GGTGGTCAACTGAGGAACAG
ZNF318_5	CAGGGCAACCTCTACCAATG
ZNF319_1	AAAGACCTACAAGCCAGCTG

ZNF319_2	AGCGCACACACAGCGCCGAG
ZNF319_3	AGCGCACGCACAGCTCCGAG
ZNF319_4	CCGGCGCACACACAAGACCG
ZNF319_5	ACTGTGTGCACTGGAATGAG
ZNF32_1	ACCAGCGAAGCTGCTCACAG
ZNF32_2	AGGAAGAGTTTCCACACCAG
ZNF32_3	CAGGAAGAGTTTCCACACCA
ZNF32_4	GTTCACAGGAGAGTTCACAG
ZNF32_5	CCACAAATATGACCACTCTG
ZNF320_1	AAGCAACGAGCAACTCTTGC
ZNF320_2	AAGGTGTGATGTCTGACTAA
ZNF320_3	GAGGACTGAATTTCGAGCGA
ZNF320_4	TGATGCCTAATAAGGTGTGA
ZNF322_1	CAGATCTTACTTCACACCAG
ZNF322_2	GTGTGAGAGAACCCATACTG
ZNF322_3	GTTGAAGAAGGGCTGAACTC
ZNF322_4	TCTGTGAGGCTTCTGACGCA
ZNF322_5	GTAGAAGTACAAACCTCATA
ZNF324_1	AGTTGTCTAGCATCACGCGG
ZNF324_2	CCGGCAACAGACCTGGCCTG
ZNF324_3	GAACGGACACAACCCTGTCC
ZNF324_4	TGTCCAGGACCACCTACAGG
ZNF324_5	AAAAGCCTGCAGAGACAACG
ZNF324B_1	ACCAGAGTTGAGCCTCCCGT
ZNF324B_2	CATCATCGGAAGGTGCGCCG
ZNF324B_3	CATTCCCGAAGGCTCTCCCA
ZNF324B_4	TGGCCAGGAACACCTACGGG
ZNF324B_5	ACCTACGGGAGGCTCAACTC
ZNF326_1	AATAGCCTTGACTCTTTCGG
ZNF326_2	ATGGATCGTGACTATGGCCA
ZNF326_3	ATGGATCGTGATTATGGCCC
ZNF326_4	CAGCCATTTAATAAGCCCAG
ZNF326_5	AAAAGCGGCGAATTGAGGCT
ZNF329_1	ACACCTGGGAGAGCAACCCA
ZNF329_2	GTTGCCAGAACTCTCTGAGA
ZNF329_3	TCAGAGACTCCATAGCAGGG
ZNF329_4	TTGGGACTGTGAGAACCAGG
ZNF329_5	AAAAGGCTTCAACCATTCCA
ZNF331_1	AATGTAAGAAGGCCTTCCGT
ZNF331_2	CCTGTCTGAACTCTGCTCAG
ZNF331_3	GAAAGACCACAGCGCTCCAG
ZNF331_4	TAAGCACGAGAGGATACATA
ZNF333_1	AGCCAAAGGCAACAGAACGA
ZNF333_2	CATTGCTGGACAGCGCACGG
ZNF333_3	GAACAGGCGGTTGGCAGCCC
ZNF333_4	TGGCCACCCATGGGTTGCGG
ZNF334_1	AAAGGAGAACTTCTGACAGA
ZNF334_2	AATGTGGGAGAACCTACTGC

ZNF334_3	ACTGCAGTTTGACTTCACAA
ZNF334_4	GAGGGTAGATCTCTTAGAAA
ZNF334_5	AATACAGATACAATCCAATG
ZNF335_1	ACTGTGACCAGTGCTGAGGA
ZNF335_2	AGAGGCCGATGACTCTGGCG
ZNF335_3	CCAGTACCGGAGCAGCACCA
ZNF335_4	GATGGCCGAGCCCAGGTCCG
ZNF335_5	ACACAGACGATGAATCAGGG
ZNF337_1	AATGCCAGGAGTGTGGGCGA
ZNF337_2	GAAGAGAGAAGACGCCGGCC
ZNF337_3	GATGTCGGGAGTGTGGGCGA
ZNF337_4	TCATCAGGCGGCTAGAGCAA
ZNF337_5	GCTTACCTAGTGAGACCAGG
ZNF33A_1	AAGGTGCGAAGTCACACGGA
ZNF33A_2	AATACTCTGAGGCATTATGG
ZNF33A_3	GTATTGTGTTCACAAACCAG
ZNF33A_4	TCAGGTAGAACAGAAGTCCC
ZNF33B_1	CCAAGCCAGAGCTTTCCAGG
ZNF33B_2	GTATTGTGCTCACAAACCAG
ZNF34_1	ACATGGAGAAGTCCTCCCGG
ZNF34_2	GGAGGACAGACACTCACCTG
ZNF34_3	GTGGGAAGACATTCACCCGG
ZNF34_4	TCAGCGGATTCACCTCCGGG
ZNF34_5	AAATACAGTTCGTAACTCTG
ZNF341_1	AAACAGGGATTCAAACCCAA
ZNF341_2	ACCGTTCCACTCCCAGCCAG
ZNF341_3	GAAACACATGCAGACCCACA
ZNF341_4	TGAGACTGTGGCCAGGGCGG
ZNF341_5	CAACAAATACTCCACCCCTG
ZNF343_1	ATCCAGGGAATTCTAGCCCA
ZNF343_2	CAGATCTCATCAAACACCAG
ZNF343_3	GTGATTTCCGACTAAAGCCT
ZNF343_4	TGAAGGATAAGGCAACATCA
ZNF345_1	AAGAGCCGAACCACTGCTAA
ZNF345_2	AAGGTCTGAACCACTACTAA
ZNF345_3	AAGGTTTGCACCACTACCAA
ZNF345_4	ACTGTGGGAAGGCTTATGGG
ZNF345_5	AAGGGCTGATCCAAAACTAA
ZNF347_1	AGGGAAGGAGCCTTTCACTT
ZNF347_2	CAAGAAGGCAATCTCACTCA
ZNF347_3	GTCAGGCATGGCTCTCACCC
ZNF347_4	TAGCAGGAAACCCAGATGGA
ZNF35_1	AAGGCAAGAGAGCTGACTGA
ZNF35_2	CTAGGGTCAGAACATATCCT
ZNF35_3	TGTCTGAAGACCCTGCACTG
ZNF35_4	TTGGCAGCTACTCATTACCA
ZNF35_5	CAGAAATGTAAGAAATCTGG
ZNF350_1	AATGAGCTGTGCCTCTCTGC

ZNF350_2	GAAACAAGAGGCAGCCAAGG
ZNF350_3	GGAGAAACAAGAGGCAGCCA
ZNF350_4	TAGCCAAGACTTCTTGATGA
ZNF354A_1	AAGGCCTGACCTTCGGCTGA
ZNF354A_2	AAGGGATGTACTGAGAGTAA
ZNF354A_3	ATCCCAAGGTACATTCCTGT
ZNF354A_4	GATCCAACAGGAATGTACCT
ZNF354B_1	AAGGGATGCACTATGGCTGA
ZNF354B_2	GCATTTAAGGACCCATACTG
ZNF354B_3	GCTTACAAAGGGATGCACTA
ZNF354B_4	GGGTCCTTAAATGCTTACAA
ZNF354B_5	AAGGGATGAATTGTGAATGA
ZNF354C_1	GAGAAAGAAGGGATGATCTG
ZNF354C_2	GAGGGTTGAACACTGGCTAA
ZNF354C_3	TAAATGCGGCGAATGTGAGA
ZNF354C_4	TTACCTAGACGGGTATCTGA
ZNF354C_5	ACCATACAAGTCTTGAATTG
ZNF358_1	CAAGTTGGAGCTCTGCCCGA
ZNF358_2	CAGCGCCGAGCTCTGCCCGA
ZNF358_3	CAGGAGCGAGCCGTGCCCGA
ZNF358_4	GATGCCACGGTGCTGAGCCA
ZNF358_5	CAACACTGTCCCGGAAGACG
ZNF362_1	GATCAAGGCGGAGAACCCGG
ZNF362_2	GGTAGGGCTTGACGCCCAGG
ZNF362_3	GGTGCCGGCGGCACTAGCAA
ZNF362_4	GTGTGGGCGGGCCTACACCT
ZNF365_1	CAACCCTCTGTAGAAGCCGA
ZNF365_2	CAGACAGGTGGACGTGGCCG
ZNF365_3	GCGGGCCTTAAACAGACAGG
ZNF365_4	TGTGCAGACCTACACTGCCA
ZNF365_5	AGAGTTGGCCCAGAAAACTG
ZNF366_1	CAACCGGATGCACAACCTGA
ZNF366_2	GAAGCCTTTCAAATGCAAGG
ZNF366_3	GCTGGAGGAAGCCTGCAAGG
ZNF366_4	GGAGGTGTAGGACTTCTCGC
ZNF366_5	AGACCTAGATGGGTTCCCCG
ZNF367_1	GAAGCGAGCAGCCCAGACAG
ZNF367_2	GAGCTGTCCACTTTGAACAA
ZNF367_3	GCCTGGAGCGATTTCTCCCG
ZNF367_4	GGCTGCCGACAACAAGGCCG
ZNF367_5	CAACAGGATGGAATCCGACG
ZNF37A_1	GTAAACGAGGGAAATTACTC
ZNF37A_2	GTGGAAGATGATCACATCCC
ZNF37A_3	GTGGGCTGACTTCTGGGTGA
ZNF37A_4	TCTCAAGTTGGAGAAAGGCG
ZNF382_1	ACACATACTAGAGCTCACAG
ZNF382_2	CTAAAGGCACTTCCACACTG
ZNF382_3	GAGGATTGCCTTCTGGCGGA

ZNF382_4	GAGGGTGGCCTTCTGGCGGA
ZNF382_5	ATGAGAAAACACATATAGAG
ZNF383_1	AATACTAGAAGCCATGGCTG
ZNF383_2	GTTGGCAGAGAGCTTACAAG
ZNF383_3	TAAGTCCCATTATCTCCCTC
ZNF383_4	TCGGATCTCATTCGTCATCA
ZNF383_5	AAGTTGTGAGCTCTTAGTAA
ZNF384_1	AAGGAGTCTCCTGTTCTCAG
ZNF384_2	CGTTACCCAGAATATCACGG
ZNF384_3	GCACCTCCGTATCCACTCGG
ZNF384_4	GGTAGCATCGACCCTAACCG
ZNF384_5	GCACATCCGTATACACTCAG
ZNF385A_1	ACTGGTCAGGGTGTAACCAA
ZNF385A_2	ATTGAGGCTGCCAAGACCAG
ZNF385A_3	GGGCCGCTTGGTCTTGAGCA
ZNF385A_4	GTAGTTGCTGAAGAGGCCAA
ZNF385A_5	AGGCAGCACCCCAACAAATG
ZNF385B_1	ACAGCAGAACTACTGTCCAC
ZNF385B_2	GATAAAGAACGACAGGCCTG
ZNF385B_3	TCCTTCCAAGGACAGCGCAA
ZNF385B_4	TTCAGCACATTTCTAGCCGA
ZNF385B_5	AATGGTTTGATATCCAGTGA
ZNF391_1	CAGAGTTGAACTCTGACAGA
ZNF391_2	GTACACAGTAGAATTCATGG
ZNF391_3	TATGGTTGAACGGTCACCGA
ZNF391_4	TCAGCAATGGAAAGCCTCAG
ZNF394_1	AGCGCCTGGACCCAGCACGG
ZNF394_2	GTGCAAGCAAAGTTTCCACA
ZNF394_3	TCGCTGCAGAGCCCGCACCA
ZNF394_4	TTGGGAGCCCAACTATCCCG
ZNF394_5	AAAGTTTCCACATGGTGACG
ZNF395_1	GATCAACCTACCTTCTGCCC
ZNF395_2	GGACAGGGACGTCAGCACCA
ZNF395_3	GGGCCTGTAGGCCAGGGCCT
ZNF395_4	TTTCCTGTAGAACTCTGTGA
ZNF395_5	AAGCATCCCCCAAATACTTG
ZNF396_1	AGAGCCGGCTCAGAGCCTCA
ZNF396_2	AGAGGAACCTATGAACAAGA
ZNF396_3	CCCGTGAAGAAGCAGCTCCA
ZNF396_4	GCTGAATCAGAATTGCGCTT
ZNF397_1	AAACTAAGATCTCCTTCCCA
ZNF397_2	AACTAAGATCTCCTTCCCAA
ZNF397_3	AATGCCAGAGTTGCACACAA
ZNF397_4	AGAGGGAGTTTGATGACCCA
ZNF397_5	ACATAATCCAGAAAGCGGCG
ZNF398_1	AAGGTCTAGAGACCAACCAG
ZNF398_2	CAAGAAGAAGAGCCTCAGGT
ZNF398_3	CCTCTCTATAGCCTGCACGG

ZNF398_4	TCTCCCACTTACCTTCACTG
ZNF398_5	AACACCTGCTCGCCAACTGG
ZNF407_1	AAGCATCGCAGACAGCACAC
ZNF407_2	AGAGGACTTCGCCCAGCCGG
ZNF407_3	ATTACCTGTAGTCACACCAG
ZNF407_4	GTAGTGGTCTTTCATTGCGG
ZNF407_5	ACGGTAACAGTGTAACCTCG
ZNF408_1	ACCCGGAATGGAGGAGGCGG
ZNF408_2	AGAACCTGTCATTAGGCCCA
ZNF408_3	AGGGCGAGGGAGTAAAGCCA
ZNF408_4	GATGCAGCAGAACCTTGCAT
ZNF408_5	AACCTGTCATTAGGCCCATG
ZNF41_1	CAGCAACAGGGAATTCCTGG
ZNF41_2	GAGGCGCGACTTCCATATGA
ZNF41_3	GATGCATCAGAGAATCCACA
ZNF41_4	GTTGATCAAATCTCTCACAG
ZNF410_1	CTGGACCCACATTCACCCGA
ZNF410_2	GGGCCAACTCCTGAACCCGG
ZNF410_3	TCAGAATACGTCCATCCCAT
ZNF410_4	TGAGTTAGAATCCAAACCAG
ZNF410_5	GCTGGGAATTCATAGACACC
ZNF414_1	CATGCAGTGCGCCTTCTCCA
ZNF414_2	GGAGGTGGGCGTCGTCTGGG
ZNF414_3	GGCTGCAGCTGGCACAGCCG
ZNF414_4	GTAGGGCAGGAACGGTCCGG
ZNF414_5	AGGCAGCCACACCGCCAGTG
ZNF415_1	AGAGACCAACGCGATAGAAG
ZNF415_2	GAACTAGCACCACAACAGGA
ZNF415_3	GAACTCTCCAATGACGCGCA
ZNF415_4	GTTGAGAAGTCTGTCAACCA
ZNF416_1	CAGCTGCAAAGAGTCCACCC
ZNF416_2	GCAGGAACTATACTTGACTG
ZNF416_3	TCTACAGAAACACTTTGCTC
ZNF416_4	TTGTCCATGTCACTTTCCAA
ZNF417_1	CGTGAGCAACTTACCCAGCG
ZNF417_2	CTTATGAGTGTAGAGAATAT
ZNF417_3	GATAAGCTGTCTTTCCAGTG
ZNF417_4	TGAGGCCACACATTTCACAG
ZNF418_1	GCAGAGACTTCCCTCTAGAG
ZNF418_2	GGAGATACTCATTACAGCTG
ZNF418_3	TAGATGACTCCTCTGACACA
ZNF418_4	TGGGTACTTATATCCTCCCT
ZNF418_5	CACCTTAGGAACCATCAGCG
ZNF419_1	AACACCTGAGCTGATCAGGA
ZNF419_2	CAGCACCAGAGACTACATGC
ZNF419_3	CTTGCTTACAGACCATGAGG
ZNF419_4	GAACACCTGAGCTGATCAGG
ZNF419_5	ACACAGGAGAGAAGTCACAT

ZNF420_1	AAGTAGTAAGCCACGCGCAA
ZNF420_2	ACTCCAATTTACCCGAGCCA
ZNF420_3	TCTTGAAGAGTCCAATTCCA
ZNF420_4	TGTGGGATTGACTTTAGTCA
ZNF423_1	AGGTGGACTGCACTTACCCG
ZNF423_2	CATTACCTCCAGGACAGCGG
ZNF423_3	GATCTCTCTCTCGTTCTCGA
ZNF423_4	GGAGGCCTCAGACTTCTCGC
ZNF423_5	AAGCATTATTACCGTCAGAG
ZNF425_1	AAGCACCTGTGTCTACACCG
ZNF425_2	TACCGAAGCCGGCTCGGCCA
ZNF425_3	TACGAGACCCTTGATTCCCT
ZNF425_4	TTAGAAGCTCATCTTACCCA
ZNF426_1	AAATGCGACTTGAAACCCAG
ZNF426_2	AAGGCGTGAGGATGTAAGGA
ZNF426_3	AGAGCAACAGAGCTTACCAT
ZNF426_4	TCAGCCACTATTCTTCCTGC
ZNF426_5	CTTGTAGAGTCAATAAAACC
ZNF429_1	GACTACTAAGGTTTGAGGAG
ZNF429_2	GTCGAGTAAGTCTTGAAGAC
ZNF429_3	GTTGAGTAAGTCTTGAAGAC
ZNF429_4	AACCATAAGAGAATTTATGT
ZNF429_5	CTGAGGAGATATGATAAACG
ZNF43_1	TGTCTCCTCATAGGCTCCCA
ZNF43_2	GAACATAAGAAAATTCATCC
ZNF43_3	GATGAGTGTAAGGTGCACAG
ZNF43_4	TTTATGTGTAGTAAGGCGTG
ZNF430_1	CCATAGGAGAACCTGAAGTC
ZNF430_2	AAGAACAGGCTGTAAAAGTG
ZNF430_3	AATTCTTCTGTGTCTAGTAA
ZNF430_4	GAATAATCTTATGTGTAGTA
ZNF431_1	GCAAGACCCAGTCACCTGTC
ZNF431_2	AATGAACTTATGTGTACTAA
ZNF431_3	ACATAAGATAATTCATACTG
ZNF431_4	CTTATACTCATCTACACTTG
ZNF432_1	AGGAGAAGAACCATGGACAA
ZNF432_2	ATGTGGAAGAGGCTTTGCCA
ZNF432_3	GCACACAAAGTACATCCATA
ZNF432_4	TACTGTGGAGTTCACCTGGG
ZNF433_1	AAAGGAATGGGAAGAGCTGA
ZNF433_2	ACAGAGGAGAGACAGTTGAA
ZNF433_3	CCTTTCATGTGTCTGAACAG
ZNF433_4	GATGTCCCTCAAACCTTCGA
ZNF436_1	AATGTGAGAAGAGTTTCAGC
ZNF436_2	ACACTATCGAGTCCACACAG
ZNF436_3	GACCTCTGGACGCTGCACAG
ZNF436_4	TATGGCCATGTATCTCACCC
ZNF436_5	AATGAGAACATATATGATAG

ZNF438_1	AGTCAGGAACCAACAAGCCA
ZNF438_2	CCACCAGTGACCAACACCCA
ZNF438_3	GAAGGTTCATTCCTCCTCCG
ZNF438_4	GCATGGAGGGATCATTGGAG
ZNF438_5	CTTTAATGCAGCCACCAAGG
ZNF439_1	GATCCTGTCACAGACATGAA
ZNF439_2	TGTGACAGGATCTGGGACTA
ZNF439_3	TTTGTGTGTGAAGTTGGCCT
ZNF439_4	AGACACATGGTAGTGCACAG
ZNF439_5	ATACATGAAAGAACTCACAT
ZNF44_1	GAAGCTGCCAATCTCATGGA
ZNF44_2	GCTGGAAGCTGCCAATCTCA
ZNF44_3	GGTTCAGCACGAATTCATGA
ZNF44_4	TGATACTGGACACAAACACC
ZNF440_1	AAGGATTGACACAGCTCTGA
ZNF440_2	ATACGTGGTAGGACACACAA
ZNF440_3	CTACCACGTATTCAAAGGTG
ZNF440_4	TATCGAAGGGAACTGGAATG
ZNF440_5	ACACACGTAAGATTGCACTC
ZNF441_1	AAGGCACTGGGACAATGGAA
ZNF441_2	ATATACGAGGTCCATCTCCA
ZNF441_3	GTACACTCACTGCTTTCCCA
ZNF441_4	TAAGAGATGAACGACCCATG
ZNF442_1	AGTGTGTGTGGAGAAATCAT
ZNF442_2	CACATAATAGTACAACGTGG
ZNF442_3	AGACACATAATAGTACAACG
ZNF442_4	AGATGGACCTCACAAATGCA
ZNF443_1	CCTCTCATACTGCTTTCACA
ZNF443_2	ACAAGAACTGGAATCAGGGA
ZNF443_3	AGTGGTTTCATGTCTTTGAA
ZNF443_4	CATGTGTTTGAAATGAGTTG
ZNF444_1	AAGCGGCGGAAGCGGTGCCA
ZNF444_2	CGTCGCCCAGGTGGAAGCGG
ZNF444_3	GCCGGGAGGCGGCTAACCCA
ZNF444_4	GGTGCCTCAGGATGTGACGC
ZNF444_5	CCCAGCAGGCACCGCCCCTG
ZNF445_1	ACCTTGATGGGACATCCTGG
ZNF445_2	GGATATCGATTACTTACCCA
ZNF445_3	TCAGGAGCAGCTCTGCTCTG
ZNF445_4	TTGGAAGCAAGGGAGCCATG
ZNF445_5	AGCCGGCTCAGAGTTTCTAG
ZNF446_1	AAGGAGGAGCCCAATGTCGA
ZNF446_2	CAGCTGGTCATCCACCGCAA
ZNF446_3	GAAGGACTGCAGCATGACCC
ZNF446_4	GCAGCTGGTCATCCACCGCA
ZNF446_5	AGAAGACAGAGGAACCACTT
ZNF449_1	ACACCAACGACTTCATACAG
ZNF449_2	AGAGATAGAGACCTGGGTGA

ZNF449_3	GAGGCATGGATCCCACAGGC
ZNF449_4	TAGAGAAGAGCCATGGGTGA
ZNF449_5	AGTTTGTTAAATGCTTCATG
ZNF45_1	AAAGCTGTGGATGATGAAGA
ZNF45_2	AAGGCTGGAGAACTGACTGA
ZNF45_3	AATCTGCTGGACCATCAAAG
ZNF45_4	AATGTGATAATGCCTTCCGT
ZNF45_5	AAGACTTGAAAACCGACGGA
ZNF451_1	CCGAAGAGGAGGCCACACGT
ZNF451_2	CCTCGGAGAACTATTTGCCG
ZNF451_3	CTACCAGGGCATCTAAACCA
ZNF451_4	TAAATCTTACAGTTGAGCGG
ZNF451_5	ACATCAGTATAAGAGGTGCT
ZNF454_1	AAACACCAGAATATCCACAG
ZNF454_2	AAGGGAAGTGTGGATGCCAG
ZNF454_3	ACCAAGACAGAAGCCTCCAG
ZNF454_4	TGTGGCTATACTGTTCACCC
ZNF454_5	AAGGTGTGCCCTGCACACAA
ZNF460_1	AATCATAGGGCTTCACACGA
ZNF460_2	ATAGGTGGAATAGTGAGTGA
ZNF460_3	GAGGTGTTTGCTCTTGCCGA
ZNF460_4	GCAGTTGGACGTGACCCAGA
ZNF460_5	AAAATGAGCCTTGAACACGA
ZNF461_1	GAAACCTTATCAATGCGGGA
ZNF461_2	GAGGCTACAGACATAAATGC
ZNF461_3	GCAGAGGAATTTGTACAAGG
ZNF461_4	GCCAGCCGTAATCTCCTCAT
ZNF461_5	AAGTTGTGAGCAATGAACAA
ZNF462_1	AATGTGAATGAGCTACGATG
ZNF462_2	ATTACATGAAACGCCACCCA
ZNF462_3	CTCGCGGGTAAACATGGCCA
ZNF462_4	GATCAAGGAGAGGAAAGTGG
ZNF462_5	ATTGGTGGCATTGTAATCGT
ZNF467_1	AGAAGAGGACATCTGCTCCT
ZNF467_2	GGAAGGATCCCATAATGCCC
ZNF467_3	TAGGCCGCGAGCCGAAGCGG
ZNF467_4	TGCCCACACAGAACAAGCCG
ZNF467_5	CAAAGGGGCGACCCTCGCTG
ZNF468_1	TGAAGACGTTGTCGTCAACA
ZNF468_2	AAGCGCTTTGTGAATGAAGA
ZNF468_3	CATGACAGAAATCAAAGAGT
ZNF468_4	CCTTCAGACTGAAATATGTG
ZNF468_5	GTCTGTGCAATGTCCCTGTG
ZNF469_1	AAGAAAGTGAAGACTCCCTG
ZNF469_2	AAGGAGCAAAGACACACGTG
ZNF469_3	GAGAGACCAAATCACTCACG
ZNF469_4	GTGGAGGTAAAGCTGGAGGG
ZNF469_5	AAAATGGCTAGGCAGCATCG

ZNF470_1	AAGATGGGCAACCTGGCTGA
ZNF470_2	GAAGAGTAAGAGACCCACGA
ZNF470_3	GCAGAGTAAGGGATTTACGA
ZNF470_4	GTACTGTCAGAGATGAGCCA
ZNF470_5	AAAAAACACAAGCAAGACCG
ZNF471_1	ATCTCACTCGTCATCTCCCA
ZNF471_2	ATGACAAGAAGCCCATTCTC
ZNF471_3	ATGGAGGGAATTACTAGCTA
ZNF471_4	TTTCCCAGGAAGAATGGCAA
ZNF471_5	AAAAGACGAGCCATCACTGA
ZNF473_1	ACAGGCAACACTCACCCAGC
ZNF473_2	GATCTGGAGCCTCTGGCAGG
ZNF473_3	TGTCGGCATGGACTTCACCT
ZNF473_4	TTAGTCAGAGTACATACCTG
ZNF473_5	AGTATGAGTGATCCAGTGCT
ZNF474_1	AGTTGCCCAAGCATTTGAGG
ZNF474_2	GAAACAGAGAGTGTTAATCC
ZNF474_3	GACGCATTAGGTGAACCTGG
ZNF474_4	TCACAGAAGCTGCAAGCCAA
ZNF474_5	ATTAACACTCTCTGTTTCTG
ZNF479_1	AATTCCGCTGAGCACAATCC
ZNF479_2	AATGCCTGGATTGTGCTCAG
ZNF479_3	ATACTGGAGAGAAACCCTGC
ZNF479_4	CTTGTGGTTAGTAAGTGCTG
ZNF479_5	GGTGAATATGAGGTGCACAA
ZNF48_1	AAACACCTCCGCACCCACAG
ZNF48_2	AAGCACCTCCGCACCCACCG
ZNF48_3	GCCCGCATCAAGCACCAGCG
ZNF48_4	GCGGAGGTGTTTGACTCGGG
ZNF48_5	ACACCAGCGGACTCATAGTG
ZNF480_1	AAGGAGCGCAATCCTATAGA
ZNF480_2	ACTTCACTCTCACCAGACCA
ZNF480_3	CAAATGTAATTCATGCGGCA
ZNF480_4	CCTAGGGAGCAGCTATGCAT
ZNF480_5	AATGCCTGGACCCTGCACAG
ZNF483_1	AAGGCATGAATTCTGCCTAA
ZNF483_2	ACTTGAGGAAGAAATCTCGG
ZNF483_3	AGCCATGATCATCATCCCTG
ZNF483_4	TAAAGTGGGTTGAATTGCCA
ZNF483_5	AGACCTTGTTCTGAACCGCA
ZNF484_1	AGATAGAAGAAAGTTGGCCT
ZNF484_2	GAAGAGCCATGTATGTTGGA
ZNF484_3	TAAGGTTTGAATTCTGAGGG
ZNF484_4	TCACCTGGAAGTCTCGGCTC
ZNF484_5	AGAGCTTGCTTATGATGCAG
ZNF485_1	AAGGCCTGAGCTGTGCCGAA
ZNF485_2	GACACCTGGATGCTGCTCAG
ZNF485_3	GCACTTATAGTTCTTCTCTG

ZNF485_4	TCAGTGTCGTGACTGTGGGA
ZNF485_5	AAGAGCTGCATTCTGAGCGA
ZNF486_1	AAAGCCTTTACATGGTCTGC
ZNF486_2	GAGGCCTGCAGACCATGTAA
ZNF486_3	CAGAGCAAAATATTTCAATG
ZNF486_4	TAAAAAAGGCTGTGAAAGTG
ZNF486_5	TATGTGTAGTAAGGGTATAG
ZNF490_1	ATGCAGAACAGTGAACACCA
ZNF490_2	CAAGGCCGCACAGGAACAGG
ZNF490_3	TATAAGGTTGAACTCCGGTG
ZNF490_4	TTAGGACTCCATCTCCCTTG
ZNF490_5	AGGGGAAAAATGGAAAGACC
ZNF491_1	AAAGCAGTGGCTATGGCTCA
ZNF491_2	ACTCTCCCAGTTCATTTCAA
ZNF491_3	AGATATATGGTAACGCACAG
ZNF491_4	AGTGCAGAAGGTAGTCAGTG
ZNF491_5	AAAGGTTTCTCTCTAGTGTG
ZNF492_1	AAAGAAACCCTACAAATGTG
ZNF492_2	AAGGTGGGATAACTGGCTAA
ZNF492_3	GAGATGGTAGCTGAACCCCC
ZNF492_4	GTGCACAAAGAATGTTACAA
ZNF492_5	CTGAGAAGATATAAAAAATG
ZNF493_1	AAGCGGTCCTCACACCTCGC
ZNF493_2	GAATCTTTAATGCCTGGCCC
ZNF493_3	GAATTTGCTTGTGCCCAGCG
ZNF493_4	AAATCCTACAAATATGAATG
ZNF493_5	AAGAATTGAGAATACACTAA
ZNF496_1	AGACCACCAAGCCAGCTCAG
ZNF496_2	AGAGCAGGCTGTGGCCGCGG
ZNF496_3	CCAGGCCTGCCCACAGACGG
ZNF496_4	CTGTAGAAACCACTCACCGG
ZNF496_5	ACCACCAAGCCAGCTCAGCG
ZNF497_1	AAGCCACCGGCGCACGCACA
ZNF497_2	GAGGTTGGAGCTCCAGGCGA
ZNF497_3	GCAATGTGAAGACTGCCACG
ZNF497_4	GCGGCAGCAAGCCACACTGG
ZNF497_5	CTTCGGCCGAAGCACCACGC
ZNF500_1	AAGTTACCTTCATTCTCCAG
ZNF500_2	CCAGGCCGAAAGGAAGGGCG
ZNF500_3	CTGGCGGAAAGTCTCAGGGC
ZNF500_4	GAATCGAGCCTCTTCCTCCA
ZNF500_5	AGAACTGTCCCCCTATCCCG
ZNF501_1	GAGACATGCACTCTGATTAA
ZNF501_2	GAGTCCTTTCATGTTCAACA
ZNF501_3	AAGGCATATGCTCTGACTGA
ZNF501_4	CAGCACCAGAGGATTCACAG
ZNF501_5	CTTCAAAGTGTAGTGAATGT
ZNF502_1	AGTAGTAAAGAGGTTCCAAG

ZNF502_2	ATTAGAAGAGAGACTTGTCC
ZNF502_3	CAGAAGAGAGTCTGCATCAG
ZNF502_4	GAGATGTGAGTGATTGCGGA
ZNF502_5	CTGTGGATGTTGAATATGCA
ZNF503_1	AAAGACACCGACGTGGGCGG
ZNF503_2	AGAAGCAGTAAGCACAGCGG
ZNF503_3	AGTAAGCACAGCGGCGGCGG
ZNF503_4	GCCTGGGTAGGTCATACCCG
ZNF503_5	AGAGGGTCCTTACCTCGATG
ZNF506_1	AAGGTTACAGGACTGCTTAT
ZNF506_2	GAGCTCTCTTATGTTTAGAG
ZNF506_3	GAGGCTTGAGTACCAGGTAA
ZNF506_4	AAAAACCTTTAACTATGAAG
ZNF506_5	ATTGCCTGGACGCTGCACAG
ZNF507_1	AGTTGACAAGAGCCAACCTG
ZNF507_2	GATCATATGAAAGAGCACGA
ZNF507_3	TAACCAAAGCAACTCCACCT
ZNF507_4	TTTGTCCGGAAACAATGTGG
ZNF507_5	CTTGCACTGATGGCCCATTG
ZNF510_1	GACTATAGAATTCACCCAGG
ZNF510_2	GTACAGATTCTTCTGAACAG
ZNF510_3	TAAGAAATCCTACCAGACGT
ZNF510_4	TTGTCCAGAAGTCAACCCTC
ZNF510_5	CAGCTGTTTGAGGATCAGTG
ZNF511_1	AGAGCGGGATCCCGCGGCTG
ZNF511_2	CCAGGACGTGATCATGCAGG
ZNF511_3	GCCCGCGTTTGCCTGCCAGG
ZNF511_4	TATCAAACCGGAAGTCCGCG
ZNF511_5	CAGAGACCGGAAGGATCACA
ZNF512_1	AAATAGATGAGCCAAGTGAG
ZNF512_2	GCAGAGGAGCACGAGGATCG
ZNF512_3	GCTGCTACTTCTCATGTCGA
ZNF512_4	TCAGCCCAATCCCAAATCCC
ZNF512_5	GTAGAGATATCCCATGATGC
ZNF512B_1	CAGGCCCATGCCCGTCACCA
ZNF512B_2	GCAAGCCGGTGACAGTCAGC
ZNF512B_3	GTGTGCTGCAGGGATGCCGG
ZNF512B_4	TTGGAGGCCGTCGGCTCCCG
ZNF512B_5	AACACCCAAAAAGTTTACAG
ZNF513_1	ACAGAGGGTGGGCTATGAGG
ZNF513_2	ACTGAAGACTCCCTCGACGA
ZNF513_3	AGTGGGACACGAAGGTGCAG
ZNF513_4	GCAGCCCGTGAAATGCGAGG
ZNF513_5	CTGGTCGTGGAGTACAGCAG
ZNF514_1	AAGGGATGAAGTGTGACCAA
ZNF514_2	GAAGAGAAGGTCTAAATCCA
ZNF514_3	TAACTGGCCATCACAACCGC
ZNF514_4	TCGAAGTTGAAAGCAGCCTG

ZNF514_5	AAGGAATCAATGCCAAGTTG
ZNF516_1	AATAAGGAGAGCCATTCCGG
ZNF516_2	ACAGTGGTGTTTGAGTCCGA
ZNF516_3	GACTTTGCGACCCTCTACCA
ZNF516_4	GAGGGACCACATCACCGCGC
ZNF516_5	CAAGATGGGAGATAACGCCT
ZNF517_1	GAGGGACCCTGGAATGGCGA
ZNF517_2	GAGGTTGGACTTGCGACCGA
ZNF517_3	GTAGAGTGCCTGCTGGTCGG
ZNF517_4	TGAAGCGCTGCCCACACCGC
ZNF517_5	AGCAAAGCTCCATCCTGCTG
ZNF519_1	GTGGCAAAGCCTTTAACAGG
ZNF519_2	CCTTTCAAGTGTAAGGAATG
ZNF519_3	GAAAGTATAGGTGAAGGTGA
ZNF519_4	GGAGCAGCTGACAGAAACTG
ZNF519_5	TCAGTGAAATATAAAGAACG
ZNF521_1	AGAGCACAGCTTCGAAGGGA
ZNF521_2	GGGTCTGCATATGTTCCCGG
ZNF521_3	TAAGAAGAGGCCGGAAGACG
ZNF521_4	TCAGCGATTCAAACACCTGG
ZNF521_5	AACTAGAGGACAGAAACCCA
ZNF524_1	AAGGCAACGGGTCTGGGCTG
ZNF524_2	GCGCAAGCACCCGGAGGCCA
ZNF524_3	GTTGCCTTCGCCTTTGCCCG
ZNF524_4	TGCGAAGCGCAAGCACCCGG
ZNF524_5	CGGCACTGCAACATCCATGC
ZNF526_1	ACTGCGGGACTTGTGGCCGC
ZNF526_2	AGTGACTGAGATGACACCTG
ZNF526_3	ATGCTCCAAGAACTCCTCAG
ZNF526_4	TGAGTTCTGTGCCAAAGCCG
ZNF526_5	CAGCCTCTATAACACACTGG
ZNF527_1	AGATATAGGAATTCCTCCTG
ZNF527_2	AGGGAAGGAACCGTGGATGG
ZNF527_3	ATGGCTGAAGCCATCTCAGA
ZNF527_4	GAGCATGCTTACCTGGGACA
ZNF527_5	AAGGTGAGCACTCTGTCTGA
ZNF528_1	ACCCTTGAAATTCATGGATG
ZNF528_2	GACTGAAGACCTTACTGCAC
ZNF528_3	TAACTGATGGGTTATGAGGC
ZNF528_4	TAGCAAACGATCCAGACGGC
ZNF528_5	AAAATAGCAAACGATCCAGA
ZNF529_1	AATATCTGGATTCTGCTCAG
ZNF529_2	ACTGGAGTTATCTATCCCAA
ZNF529_3	TCACTGTCATGAGTTCTCCG
ZNF529_4	TGTGAGAAAGCATTTGGAGT
ZNF529_5	AAAAGCAAGATTGACTTACA
ZNF530_1	AAAGAGCTGACTCTGGAGAA
ZNF530_2	GAGGACAGATACCTGGCGAA

ZNF530_3	GGAGTGCCCACACTAGCACA
ZNF530_4	GTCGAAAGAGGCCAGAGCTT
ZNF530_5	ATAAGGCCTTGTGCTAGTGT
ZNF532_1	AGAAACCAGGACTTACGAGC
ZNF532_2	CGTGAGGAAGGAGCACGGGA
ZNF532_3	GATAAGCTGAAGGCACTCGG
ZNF532_4	GGTGTGATGCATAACTGCAC
ZNF532_5	CAGCAGGCTGCAGATACGAG
ZNF534_1	AGCAGATGCAGGCTTCTGGA
ZNF534_2	GCAATTGTCATTCAGCGATG
ZNF534_3	GCACAAGGTGTGAATTCCGA
ZNF534_4	TATGCTGTGCAAGGTAGGCA
ZNF534_5	AAGTCACTTAAAAATCAACA
ZNF536_1	AGAGGACATGGACTCCTCCA
ZNF536_2	ATGCAGCTGGATTTCAGCGG
ZNF536_3	GAAGAAGCGAGCCTGTGCCT
ZNF536_4	GACCTCCGAAGACATGCCCG
ZNF536_5	ACTCTCGAGTATAACCTGCA
ZNF540_1	ACTGTGACTTACATGGGCCA
ZNF540_2	AGAGCCCTGCGTGGTGGCGA
ZNF540_3	GAACACAGAAGAACTCATGC
ZNF540_4	GTGACAGGAAGACAGTGCCC
ZNF540_5	AAGTTGTGGGTAAAGAGTAA
ZNF541_1	AAGGAGGAAGGGCCACCTCG
ZNF541_2	GAAGGGAGGTGCATTTCTGA
ZNF541_3	TCGAGGGAAGCAGGAACCGC
ZNF541_4	TTCGGGCCAGCACTCCGCGG
ZNF541_5	ACGGCAGACCCAGTTAGGTG
ZNF543_1	CAGGATGGCAGCCTCGGCGC
ZNF543_2	GCCACGTGAGGTATGACCGG
ZNF543_3	TCACAGACAGGAGCTATGGA
ZNF543_4	TGGCACCAACAGATTCACAC
ZNF543_5	AAAATGAGTTCTGAACGTGA
ZNF544_1	AAGGTCTGCTCTTACCTCGG
ZNF544_2	CAGGGAGACAATATGCTCCC
ZNF544_3	GAGGTTAGAGTTCCACCTGA
ZNF544_4	TAACTGATAGCTTTGGCTGA
ZNF544_5	CAGAAAAAGATGAGGCCATG
ZNF546_1	ACACAAACTTACATTGGCCA
ZNF546_2	ACATCACACAATCCATGCTG
ZNF546_3	GATTCTGTGCTTCTCCATGG
ZNF546_4	TGAACAATGGACCATGCAGG
ZNF546_5	AAGTAAAAACACCATCCATG
ZNF547_1	GAGGAGGCACCTTTAGAGCC
ZNF547_2	GAGGTGGGACTTTGTAAGGA
ZNF547_3	TGGGTAGATAGACAGGGCTT
ZNF547_4	TTAGAGCCAGGTGTTTCTGT
ZNF547_5	ACGTGTACAGTCCCTGCTCG

ZNF548_1	ACACAAGCCTCTCTTCACTG
ZNF548_2	ACTAGGGTGGACCTTCTGGC
ZNF548_3	GGAGATCTTCACATGCATGG
ZNF548_4	GGTGTCTAATGAGCCTGCAG
ZNF548_5	CTCGCTTATGTCTCATGAGT
ZNF549_1	ACAAGAATGAGCATTTGGGA
ZNF549_2	GACGTATAAGGTTTCTGCCA
ZNF549_3	TGGAGAGAAACACATCAGAA
ZNF549_4	TTTGCAGCTATTCAGAAGCA
ZNF549_5	AGAGTATGCACATAGAAGCA
ZNF550_1	AATGTGACTCACAGCAACCA
ZNF550_2	ACCGGAGACAGACCTCCACA
ZNF550_3	GCACTACCTCATCCACACCG
ZNF550_4	TCTGAGCGGGCCTTTCTCCG
ZNF550_5	AAGACGCCTTGATTCATGCA
ZNF551_1	ACTGCCGCCATTGAGCTCCG
ZNF551_2	ATTACAATGAAGAAGAGCCC
ZNF551_3	CAACACGAAGCCACTCCCAG
ZNF551_4	TAGTCCACGGAGCTCAATGG
ZNF551_5	CACACATCTTAATCTCACTG
ZNF552_1	ACACAGAAGGTTCTTCTGTA
ZNF552_2	CCCACGTGGTCCCATGGCGG
ZNF552_3	GAAGCTGTCATATTTGCTAG
ZNF552_4	GCGCCACAATTACCTGAACG
ZNF552_5	AGGAGGCGTTGTTTGCGAAG
ZNF554_1	AAGTCACTAGTCTTTCCTCA
ZNF554_2	AATGGGCAGTCATTGAACCA
ZNF554_3	AGTCACTTTATTCAACACGG
ZNF554_4	TTGCAGGAGGAACCAGCCAG
ZNF554_5	AGAACCAATGTACTGATGTG
ZNF555_1	AAGATCAAACCACAAACCAG
ZNF555_2	AGTCCCATCACAGTTCACAC
ZNF555_3	GAAACTCAATTTAAGGCCAG
ZNF555_4	GAAGATCAAACCACAAACCA
ZNF555_5	CGATTGAGGGAGGAAGTACG
ZNF556_1	ATGTGATGATGCACGCCGGA
ZNF556_2	CATATGATCATGCACGCCGG
ZNF556_3	CATGTGATGATGCACGCCGG
ZNF556_4	TAAAGACAAGCACAACACCA
ZNF556_5	ATATGATCATGCACGCCGGA
ZNF557_1	CAGCGAGAAGGACACACAGA
ZNF557_2	CGAGAAGGACACACAGAGGG
ZNF557_3	GAAGAGAGAGGAATTCTCTC
ZNF557_4	GCACAAGAGAGTTCATACGG
ZNF557_5	GAATTCTCAAGTGCGGTCTG
ZNF558_1	AAGCCACAAGAGGATTCACA
ZNF558_2	CACGACTCACCAGCAGTCGA
ZNF558_3	CAGATAGGAGCTCTTCCTGA

ZNF558_4	GTTGGAACAAGACAAGAAGG
ZNF558_5	AAATGCAGTCTAAGGGATGA
ZNF559_1	AAGGCCTGATGAGATACTGA
ZNF559_2	AAGGTGCGAGGAGTATCTAA
ZNF559_3	ATTCACATAAGGGTTCACAC
ZNF559_4	TCTTAACAGCGCGTTCCCGT
ZNF559_5	AAGATGTAGATGTTGGCTGA
ZNF560_1	AAACATTTACGAACACACGC
ZNF560_2	AAGGCCTGCAGATGTACCAA
ZNF560_3	TACAGCTGTGGACTTCACCC
ZNF560_4	TTGCAGCAAGGAGTTCTCCA
ZNF560_5	ACATGTTCAAGAAAGCCTGA
ZNF561_1	GCAGCCATTTATTTGTCCCG
ZNF561_2	GTGGAGGACTACCTGGCAAG
ZNF561_3	TGACTGTAAGAATTGTGGAG
ZNF561_4	TTACCTGATAACCACTTGCC
ZNF561_5	AAAGTCTTTACTCTAACTCC
ZNF562_1	ACTCTGTGAGAATTGTGGAG
ZNF562_2	CTTACCATGGGACATATCAA
ZNF562_3	TAAGTGTGAGGAAGTTGTGA
ZNF562_4	TCAGCCTTTGATATGTCCCA
ZNF562_5	AGACAGCATCAGTGTGCACA
ZNF563_1	AAAGGAGTGATGGTAACTGA
ZNF563_2	AGATGGGCCGAATAAATGCA
ZNF563_3	TCACACTCAGCGCTTTGACA
ZNF563_4	TCGCGTGGAAGGCCTCACAC
ZNF563_5	ATAGGAACTGGAATCAGGCA
ZNF564_1	AGGCACATCAGATCTCACCT
ZNF564_2	GACAGAACTAGGACAGTCAA
ZNF564_3	TACACAGGCCAGGTTTCTAA
ZNF564_4	TCATATGATTAGGCACACTG
ZNF564_5	AAAGGATTGACAAGAACTGA
ZNF565_1	AAGCAAGTGAACGTCACCTA
ZNF565_2	ATTTCAACATACCTGGGCCA
ZNF565_3	CAAATGATGTGACAGGACCA
ZNF565_4	GCTGAACAAGGTGTGAGGCA
ZNF565_5	CCTTAAATGCAGTGACCTGG
ZNF566_1	AAAGAACTCGGCTCTCAGGG
ZNF566_2	ACAGTATCTCACCTGAGCCA
ZNF566_3	GAAAGAACTCGGCTCTCAGG
ZNF566_4	GCTGACAGAGAGCTAACAAG
ZNF566_5	GAGAGCTAACAAGAGGCCAG
ZNF567_1	AAGGGTTGTCTTCTGGCGGA
ZNF567_2	CCATCGCCTTACCTGAGCCA
ZNF567_3	CTATTGCCACCTCATCTCTG
ZNF567_4	GAGGTGTGACTTCAATCTGA
ZNF567_5	ACACATAGTAGACCTTACAA
ZNF568_1	GAGGGATGAGATTCGAGAGA

ZNF568_2	GGAGCGTTTGAGCCACATGA
ZNF568_3	TAGGGATGAACTCTGAGAGA
ZNF568_4	TCAAGAGTCTGCCCTTTCCG
ZNF568_5	AGGAAGAAGAGGATACAACC
ZNF569_1	AAGCAGTGAGCACTGAGAGA
ZNF569_2	AAGGGATGCAATTCGAGGGA
ZNF569_3	GGAGCAAGAAGAAGAACCAT
ZNF569_4	GTATTAAGGAGACACTGGCA
ZNF569_5	GAGCAAGGGATGCAATTCGA
ZNF57_1	AGGCACGTCAAGTCTCACTG
ZNF57_2	CAAATGCGAGCAGTGTCGGA
ZNF57_3	CAGACAATACCAAACTTCAC
ZNF57_4	GGGACTCAATTTAAAGCCAA
ZNF57_5	AAACCTTGCGAGTCCACATG
ZNF570_1	AAGGCAACCAGGTAATCAGA
ZNF570_2	AAGGTAGGCAAACTGGCTGA
ZNF570_3	GTACAGTAAGGTATGCACGA
ZNF570_4	GTGAAGAGAGAGCTGACAAA
ZNF570_5	ACAAAAGATAATCCCCAAAG
ZNF571_1	AAGAAACAGCAACTCACGTG
ZNF571_2	GAGATCAGAAGTACGACCAA
ZNF571_3	GGAGACAATATGGAGTGCAA
ZNF571_4	TCATCGGATAATTCCTACCA
ZNF571_5	GTCATGCCTTATTCAACATG
ZNF572_1	CCTTTGGTGAATCACCAGGG
ZNF572_2	GCACCGGAGGACCCACATAG
ZNF572_3	TTAACCTTATCAGGCACCGG
ZNF572_4	TTTCAGTCAGAGTTCCACCC
ZNF572_5	GATGGCAAGTCAGAGAATTG
ZNF573_1	AATACCTGGACCCTAATCAG
ZNF573_2	AGGGAAGAAACACAGTTCAC
ZNF573_3	TAGAAGAAAGTATGAGGCAG
ZNF573_4	TGTGGGAAGACCTATACTAC
ZNF573_5	GGGAGGGCCTTTAGTCAAGG
ZNF574_1	GACGCAGCAAGAAGGAGCGG
ZNF574_2	GAGGAGGGCCTCTGTGCCGA
ZNF574_3	GGGAAGACACGACTGCACAG
ZNF574_4	TAGGGAAGACACGACTGCAC
ZNF574_5	ACATGAATGCAGAGGATTCG
ZNF575_1	GCGATGGGCGGCCAGCTTGG
ZNF575_2	GCGGTGGGCTGCCAGCTTGG
ZNF575_3	GGTCACTGGTTCCTTGCCAG
ZNF575_4	GGTGAGCCGCCAGCTTGGAG
ZNF575_5	CGAGAAGGCCTTGTCACAGT
ZNF576_1	ACTACATTCGGCATGCCCGG
ZNF576_2	CATGAAGCGGGAGCACCCAG
ZNF576_3	GTGCAGGCGAAGGTGCCAGG
ZNF576_4	GTGCGGTCAGGACTTTGCTC

ZNF576_5	AGTGGTTGCAACCGGCAGGG
ZNF577_1	AGAGCAAGGCAGTTCTTCAG
ZNF577_2	CGGTGGAGAAACCTTCCTCA
ZNF577_3	GAGGACTGACATGTGGGCAA
ZNF577_4	GAGGTATGACTCCTTGAGGA
ZNF577_5	AATGTGGGAAAACATTCATG
ZNF579_1	AGACCACTCAGCCTTCGAGA
ZNF579_2	AGGAAGAGCCGCCCTCTCCG
ZNF579_3	CCACGCACAGGTGCACGCGG
ZNF579_4	GGGTGCACGGAACTCAGCCA
ZNF579_5	AGTCTGGGCACACGAAAGGG
ZNF580_1	ACGCCGCTTCCAGGACGCCG
ZNF580_2	GGAGAGCCCGGCCCTCGCAA
ZNF580_3	GGCAGCTGTAGCCCTTGCGA
ZNF580_4	GGTCCGAGTGCGACACGCGG
ZNF580_5	AGGAGGGGCCTTCCGCCTTG
ZNF581_1	ACACAGCATCACCCACTCGG
ZNF581_2	CACTGCAGAAACACACGCGG
ZNF581_3	GAAGGGCTTTACCTCCGAGT
ZNF581_4	GGTGGACGAGGAGTCACAGA
ZNF581_5	AGCCTGGGGAGATCCGATGG
ZNF582_1	AAGATGTGAGACCCGTTTGA
ZNF582_2	GGAGTCCAGATATGATACCA
ZNF582_3	TATGTGGTAGGGCCTTCAAA
ZNF582_4	TCTCCCAAGAAGAATGGCAG
ZNF582_5	ATGAAGTCGAATCACCCCAA
ZNF583_1	AATCCTTAAAGCCATGTCCA
ZNF583_2	GGAACAAGAGGCCCATGCCC
ZNF583_3	GTACAATTAGGTATCCACGA
ZNF583_4	GTTGCGCCAAGTTTGCACTC
ZNF583_5	ATCTTGGCAAACATTCCACC
ZNF584_1	GACGGTATATTTCTCCAGGG
ZNF584_2	GTCGTGGGTGCCCAGCTGGG
ZNF584_3	TCAGTTACCCGTCTAAGCTG
ZNF584_4	TGTGACGGTATATTTCTCCA
ZNF584_5	AATGCGTTATCAGATGGTCA
ZNF585A_1	AAGAGAAGCCTTTCCCACAC
ZNF585A_2	AATTCTCTGATGCCTGAAGA
ZNF585A_3	ATATCAAGTTCCTGAAGCAG
ZNF585A_4	GTACACCTGAAAGTTCTTGC
ZNF585A_5	ACCTCAAAAAATGTATCCTG
ZNF585B_1	GTACATCTGAAAGTTCCTAC
ZNF585B_2	GTATCAAGTTCCTAAACCAG
ZNF585B_3	ATGTATGTATTGAATGTGGG
ZNF585B_4	CCATATGAATGCAATAACTG
ZNF585B_5	TGGACTGGCCTTCATCCGGA
ZNF586_1	AAACACAGGAATGTTCGCAC
ZNF586_2	AACCACAGGAAAGTTCACTC

ZNF586_3	CATATATACAAAGACCAGGG
ZNF586_4	GGCGACGGAACGAAGAACTG
ZNF586_5	AAACACTTGAGAGTTCACAC
ZNF587_1	ACAGACAGCTTATCCCTGTG
ZNF587_2	ATGAGGCCACACATTTCACA
ZNF587_3	GAACGTCCTTCCCAAACTCG
ZNF587_4	GGAGAGAGAGCTTATCACTG
ZNF587_5	AGCTTGTTCACACTGGAGAA
ZNF587B_1	ACGTGGTTCGATGGCGGTGG
ZNF587B_2	GCATGCGCTGATGGATCCTA
ZNF587B_3	GCGCTGATGGTAACTAAGGC
ZNF587B_4	ACACATAACATTCTTCTCGA
ZNF587B_5	GGTTCTCCAGAGTCACATCA
ZNF589_1	AGAGACTGAGCCTTGAGCAG
ZNF589_2	AGCCCAGTAGCTGCTCCCGC
ZNF589_3	CAGCCCAGTAGCTGCTCCCG
ZNF589_4	GCAGGAAATCAACTCCACCC
ZNF589_5	CCTTCTGATAAAAATCACAG
ZNF592_1	GAATGTCAGCAAGACGCAGG
ZNF592_2	GGTGGGCACAGAAACTGCAC
ZNF592_3	TCAGGTGGACGACACCACAG
ZNF592_4	TGAGCCAGCACTATGGCCGG
ZNF592_5	AGCCCCGGAATCCATTGTGT
ZNF593_1	GCCCACGGAAGTGTCCACTG
ZNF593_2	GGCAGATGAAGGCGAAGCGG
ZNF593_3	TCGGAAGTGGGTCTTCAGGT
ZNF593_4	TGTCCGGCGGGAGCGACCCA
ZNF593_5	ACCGAGCGCACTCTCTAGCC
ZNF594_1	AAGGAGGGAACGCCGCCTGA
ZNF594_2	AAGGAGTGAACGCCGCCTGA
ZNF594_3	ATTGTGGGAGAGATTGGCCA
ZNF594_4	GGAAGAGCAGAGAATTCACC
ZNF594_5	CACCAGAGAATCCACAGTAG
ZNF596_1	AAGGACAGAAGAGTGATTGA
ZNF596_2	AAGGTCAGAGCAATGAGTGA
ZNF596_3	CCACATAGATGACATCCGTG
ZNF596_4	GAGGTCAGAAGATTCAGTGA
ZNF596_5	CACAGATATGCCATCTATGT
ZNF597_1	GTCGGGATAGATGAGAGTGC
ZNF597_2	TCAATCATAGAGCCAACCTG
ZNF597_3	TTCCGAGAAGAGCCACGACG
ZNF597_4	TTGCTGAGGGACCTCTGGGC
ZNF597_5	AGAGTTCTACTAATGGGGTG
ZNF599_1	AACCCTCCACTCACCAACGC
ZNF599_2	GAAAGAGGATTCCTCAGAGA
ZNF599_3	GGAAAGAAACTCTATGAGTG
ZNF599_4	TAAGGAAGAGCTGTCACAAA
ZNF599_5	AAGACCCCATGACTGATGCA

ZNF600_1	AAGGTATGACCTCAGACGGA
ZNF600_2	CTACGATGGTGAACAAGGGA
ZNF600_3	GCAGCAAGACCTTCCGTCTG
ZNF600_4	TTGCAAGGTATGACCTCAGA
ZNF600_5	AAGGGATGATACCTGACTGA
ZNF605_1	AACTGGAACAACAAGATCCA
ZNF605_2	AAGCTGCGACTTCCTACTGA
ZNF605_3	AATCCAGCAACGAAGAGCCA
ZNF605_4	ATGTGATTAGCAGTGAGCCA
ZNF605_5	CTATCGATGCAGTGATTGTG
ZNF606_1	AGGAGAAGAGCCGTGGTCAG
ZNF606_2	GATGGCAGCCATCAACCCGT
ZNF606_3	GGATGACAGCTGTTGACCCA
ZNF606_4	TTTGAGGAAGAACAATCCCA
ZNF606_5	AAGTGCTTGTATGTTGAGTA
ZNF607_1	ATTGTGAGGGAAGAAACAAG
ZNF607_2	GGACGACTTACTCGACATCA
ZNF607_3	GGAGTGGGAATATCTCAGCC
ZNF607_4	TCAGAAGACCTTGTACCAGG
ZNF607_5	AAGGTGGGAAAACTGACGAA
ZNF608_1	AAACAGCCCGGTATGCAGGA
ZNF608_2	AGACTGCTGGAAATCGGCGG
ZNF608_3	GAAACTAGAGGACTCCAAGG
ZNF608_4	TAGGCCATCAGCTAAGGCTG
ZNF608_5	CCAAGCGGGATAAGGATGCG
ZNF609_1	AAGGCGAGACTCCTCTGACG
ZNF609_2	ACACTAGCAAACCCACTCCA
ZNF609_3	TATGGCAGCAAGGTCTCAGG
ZNF609_4	TGCTCGACACACCTTCCCAG
ZNF609_5	AAAGACTCACCATATTACCA
ZNF610_1	AGAAGATGGGCCATAAGGCC
ZNF610_2	AGAGCATTTCACAAGCGTCC
ZNF610_3	GAATTGCAGCTGTTTCAAGC
ZNF610_4	TTCACTGCGTACCTTACAGA
ZNF610_5	ACGCTTGACATTCATGGACG
ZNF611_1	AATGCCTGAACCCTTCACAG
ZNF611_2	CAAGTGTAATGAATGTGACA
ZNF611_3	CATTCCAAAGAATTTCCTGT
ZNF611_4	TCTTTCCAATGTAATAAGAG
ZNF613_1	GAGCCCGGACTTTCTGGAGA
ZNF613_2	GAGGTCTGAGCTGCCACAGA
ZNF613_3	GAGTACGTCGATGAATAAGG
ZNF613_4	TGGATGCAGCCTGTGTGGGA
ZNF613_5	CATTGAGCTGTGATTTCCAG
ZNF614_1	AAGAGCGTGCAGCAATGCAA
ZNF614_2	AATGTGAGCAAACCTTCCTT
ZNF614_3	ATACAAGAGAATCCTGGAAG
ZNF614_4	TGGCACATGGACAAGAACCA

ZNF614_5	AAATGAGCTGAGACTTCCTA
ZNF615_1	AAACTGAGACAACTTGAGGA
ZNF615_2	GAGGCATGTCTTCTTCCTGA
ZNF615_3	GGAGACAATGATGCAGGCCC
ZNF615_4	TTCGCTGATGCACAATAAGG
ZNF615_5	AATATTGTTAATCAGAACAA
ZNF616_1	AAGGTTCCAAACAGTGGCAC
ZNF616_2	AGGAGAAAGGTTCCAAACAG
ZNF616_3	GCATTTATAAGCTTTCTCGC
ZNF616_4	TGTCACCGGAGAATCCACAG
ZNF616_5	AAGAGATCAACATAGTCAAG
ZNF618_1	ACGGAGCCAGTGCAGCCGGA
ZNF618_2	CGAGCAAGGAACGATGACGG
ZNF618_3	GGACCCATGAACCAGCCGGG
ZNF618_4	TTCGCACAGATCCACCGTGG
ZNF618_5	GGTGCGGACATTCGCGACAG
ZNF619_1	CCAGCCTGCACCCTACGCAG
ZNF619_2	GAAGAAATACCGAACTGCGG
ZNF619_3	GATCCCTGGACACTTGCTGG
ZNF619_4	TAATGGAGACCCAACTGCAG
ZNF619_5	AGTCACAGCTACATGATACA
ZNF620_1	GAAGACACCTGTCCAAGCAT
ZNF620_2	GAAGACAGAGCTGCTACTGA
ZNF620_3	GCGCCAAGCGGTCTGGAACA
ZNF620_4	TGTGACATTTCTCATGTCGG
ZNF620_5	ACAAATCAGACAAATCCTAG
ZNF621_1	CCAAATCTGATACTTCGAGG
ZNF621_2	CCAGCCTCGACCCTGCGCAG
ZNF621_3	GCAGCATCAGAGATTACACA
ZNF621_4	GGTCAGTGAAGGCACAGCTA
ZNF621_5	AAAGGCTTTCCGTAGGAGTG
ZNF622_1	CATCTGTGAAGAGCTTACAG
ZNF622_2	GAAGCCCTCGGCGGTCACTG
ZNF622_3	TAAGCAGTCCGTGATAGGGA
ZNF622_4	TGACAAATGCACTTACCAGA
ZNF622_5	GGGCCCACTATAAGACGGAC
ZNF623_1	AATCAAAGGGTTCACCAAGA
ZNF623_2	AATCCAGAAGGTCAGAGCCT
ZNF623_3	GACGACGTCAGACAGACTCA
ZNF623_4	TCAGAGAGAGCTCATAGAGG
ZNF623_5	CTTACACGTGCGATCAGTGT
ZNF624_1	AAGGCTCGAACTACTCCTGA
ZNF624_2	GCAGCCATAATCTCTCCCTC
ZNF624_3	GGTTGATGGACCCTACACAG
ZNF624_4	TAAATGTACTGACTGCGAGA
ZNF624_5	AAGACTCCCACTAGTCAAAG
ZNF625_1	AACGCTCGAGGCACATCCAA
ZNF625_2	AGGCACCACAGAGCTGACAC

ZNF625_3	TGTAGCTCCAACACTTCGAA
ZNF625_4	ATACAAACTGAGACACATCA
ZNF625_5	ATTCATGTGTTCGAAAGTAG
ZNF626_1	AAGGCTACAAGAGTGGTTAA
ZNF626_2	AAGGTCTGAAGACCGCTTGA
ZNF626_3	GTCTAGTAAGGCTACAAGAG
ZNF626_4	ACCATTGCAATTTAGAGATG
ZNF626_5	CTGATCACCTGTCTGGAGCA
ZNF627_1	AACTCAAGGCTCGTCCACAC
ZNF627_2	CGTGAAAGGACTCATCCTGG
ZNF627_3	GAAGACACATGTTAACGCAT
ZNF627_4	GTTCTGTTCGAAGTCACGAG
ZNF627_5	AAAAGAGCGATGATAACTCA
ZNF628_1	AGTGCGGCCTCACCTTCAAG
ZNF628_2	CACGGTCCAGCTCCAGCCCG
ZNF628_3	GGCCTGCGGGCCTTCACCTG
ZNF628_4	TGCTGGCTGGAGCTGGACCG
ZNF628_5	GGCCGGTGTGCACGCTACGG
ZNF629_1	ACTTGAACGGGTCCTCGCGC
ZNF629_2	AGCTGCGGTAGAAAGCCCGG
ZNF629_3	GAGGGCTTCAGCCAGAGGCG
ZNF629_4	TGGAGCCCGAGACTGCGCTG
ZNF629_5	AGCACCGGGTGATCCATGAG
ZNF630_1	AATGTGGGAAGACCTTCCCT
ZNF630_2	AGGGAATCTGCCCTTACCCA
ZNF630_3	GAGGTGTGATTTCTCACTGA
ZNF630_4	TATAATCACCTGGTCTCCGT
ZNF630_5	AAATTTCTTATACTGAACAT
ZNF638_1	GCTCAAGTCACAGATTCCGT
ZNF638_2	TATGAGGCTCATCATCCCAC
ZNF638_3	TCCAAAGATAGACTTACCAG
ZNF638_4	TCTTCAGTACAACCATCCTC
ZNF638_5	ACTGAAGCGGATGCTACAAG
ZNF639_1	ACTGACCTGAATAACGAGAA
ZNF639_2	AGTCAACCAGCAAACCCAAG
ZNF639_3	GGAATAAGCAGAATTGCAGA
ZNF639_4	TTACTGAGAGCTAATAGCAG
ZNF639_5	AAATGTGTGTCGAGTATGCA
ZNF641_1	GAAGCCAGGAAGAGCGGCCA
ZNF641_2	GGTCCTGGTACTGTTCTCCA
ZNF641_3	GTGGGAAGAGCTTTGGCCGA
ZNF641_4	TATCAGCTCAGTCTGCTCCC
ZNF641_5	AACAGTATCTTCAGACACGC
ZNF644_1	AAAGTGGAAGGTCAGGAGCC
ZNF644_2	ACCATGCCAGCTCCAGTCCG
ZNF644_3	GAACACACTTGTCAGCTCTG
ZNF644_4	GGGTTATCAAATCATGTCAG
ZNF644_5	CAAGATAACTCGTTATACCG

ZNF646_1	GATGGTTAACCAGGCTCCCG
ZNF646_2	GGAGCACGGGTACAACCCTG
ZNF646_3	TCAGTGCTCCCTCTGCCCGA
ZNF646_4	TCTGGGATGGGCCCAACCTG
ZNF646_5	CTTCTGCGATAGCCTCACTG
ZNF648_1	ACAGAGGAATCCCTTCTCCC
ZNF648_2	GCACCAACAGATCCACTCCG
ZNF648_3	TATGTGCTGGGCCAACTCAG
ZNF648_4	TGTGTGCGAGACCACTGGGA
ZNF648_5	AAAGTGTAGACACGTCCGCA
ZNF649_1	ACACTCAAGGGAGAAACGGG
ZNF649_2	GAACACGAGAGAGCTCACAA
ZNF649_3	GAGCCTGTACCTCTTGTAGA
ZNF649_4	TCCCACACAAGCTACACCCG
ZNF649_5	CAGTAAGCTCAGATTTCCTG
ZNF652_1	AAGATGGCACTTGACCACGA
ZNF652_2	AGAAAGTCAGCGTTACACAA
ZNF652_3	AGAGTGAGGACAACTTTCTG
ZNF652_4	GTGTTTCCGCACATGAGCCA
ZNF652_5	CCAGGGTATTTAACACTCGC
ZNF653_1	AGCAGCCGAGGAGGGCGCAG
ZNF653_2	GAAGGAGGAGCCAGTAGCCC
ZNF653_3	TACACCCAGACAGAGCCCGA
ZNF653_4	TTCGCACTGCCACACGGCCG
ZNF653_5	CCGGCCGCGACTCACGGAGT
ZNF654_1	ACAGTAATGAAGTGGAGCAA
ZNF654_2	ATCCGGACACAACCAAGAGC
ZNF654_3	GGAGGCAATGGTCTGACGTA
ZNF654_4	TGATCAGGAAGTCACTGCTT
ZNF654_5	CAAGCCTTTAACTGAATGTG
ZNF655_1	ATGGAACAGGGACTCACTGG
ZNF655_2	GATACCTGGACCCTGTTCAG
ZNF655_3	GATGGAGGAAATACCAGCCC
ZNF655_4	GCAGGGACGAGCAATATCGA
ZNF655_5	CCCAGAGCCTCAGTTTGTGC
ZNF658_1	ACATGAGAACTGACACAAGG
ZNF658_2	CACATGAGAACTGACACAAG
ZNF658_3	GGGAAGACATCACATCTCAA
ZNF658_4	TCCCAGAAATCATACCTCAG
ZNF658_5	ATATTGCATTACTAAACCTA
ZNF660_1	AACTTGAGAAGTCTGCCTGA
ZNF660_2	AAGGCCCGAACTCCGACTAA
ZNF660_3	ACACTTGAGAACCCACACTA
ZNF660_4	TCACATCTTATTCGGCACCA
ZNF660_5	AAGGAGTGTGGGAAAACATG
ZNF662_1	ATTCCTGGGATCCACACCAC
ZNF662_2	GCAGCTCGAGGACAAGGCCG
ZNF662_3	TGAGAACGAATGGATCGGCC

ZNF662_4	TGTCCCGCTCGCCAGCGCCA
ZNF662_5	ATGGCCTGGTTACCTCAATG
ZNF664_1	GAGGCTCGTACTCTGACTGA
ZNF664_2	GGACTCTCTGATGCATGCAA
ZNF664_3	GGACTCTTTGATGCATGCAG
ZNF664_4	TTGATGCATGCAGAGGCTGG
ZNF664_5	ACATAAGAAAATCCACACAG
ZNF665_1	AAACACGGATTTGCCACCAA
ZNF665_2	ACACGGATTTGCCACCAAAG
ZNF665_3	GAAGAGCTCAACGTGATAGA
ZNF665_4	TATGGGAGAAGCGTTCTACA
ZNF665_5	CATACGACTTTGAGTGTCAG
ZNF667_1	AAAGGATGCCTTCTGCACGG
ZNF667_2	AGGAAAGGATGCCTTCTGCA
ZNF667_3	GAAAGGGAAAGCACCCTGGA
ZNF667_4	GTCGTGTGAGAGATATTCGG
ZNF667_5	AAAGCTCCTACACGAAAGAG
ZNF668_1	ACACGGGCTGCATGAAGCGG
ZNF668_2	ATAGGGCCTAGGCTTGGCCG
ZNF668_3	ATCACACCACCTGACTCCGG
ZNF668_4	GAGGCTGGCACGCTCGGCGA
ZNF668_5	CAGGCTCGACGACACCACAA
ZNF669_1	AGGTTCCGGAGCCGATGGCG
ZNF669_2	GAAGTCATGATATAGAGGCT
ZNF669_3	GAGTCGCCGTGGCGTAGCCG
ZNF669_4	TGGAATGTTCTAGGACTCGG
ZNF669_5	AAGATCACTTCGAAAAACCT
ZNF670_1	AAGCTCTTTCATGCTTTCGA
ZNF670_2	ATATGGAGAAACCTTCAGCC
ZNF670_3	ATGCCTCAGAGCACTTACAC
ZNF670_4	CAAATTTGAATCCTGGCTGA
ZNF670_5	AGACAGGATGTGCCTATGAA
ZNF671_1	CAGCACCAGAACCAGCCCAA
ZNF671_2	GCAGTCATGAAACTAGAGCG
ZNF671_3	GGAGCTAACGGACTCCGCGC
ZNF671_4	TTAGCTCCGCCATAGGACCG
ZNF671_5	AAGAGCACTAAGCTTGTGAG
ZNF672_1	ACTCCGCTGGCCGTTCGCCC
ZNF672_2	CGACATGAACGAGCTCACGG
ZNF672_3	GAGCAGCGCGCTCTGCCGGA
ZNF672_4	GGTGTAGCAGCAGCGCCGGG
ZNF672_5	CCACAGTCGCCACATGCGTA
ZNF674_1	AGCCCAGTCCCACTAAACAT
ZNF674_2	ATTAGAACTCACCTGGGACA
ZNF674_3	GAGAGTTGATTTCACACTGA
ZNF674_4	GGGACTGGGCTTCTCATCAG
ZNF674_5	ACTCCCTAAATATTATTCAT
ZNF675_1	ACATGAAAGAAATTATACCA

ZNF675_2	AGAGCAACCCTACAAATGTG
ZNF675_3	GAGATGGTGAATGAACCCCC
ZNF675_4	GGATGAATGTAAGTTGCACA
ZNF675_5	TAGACATAAGATAAAACATA
ZNF676_1	AAGGCTTGAGGATCTGCTGA
ZNF676_2	ATAAGGCATACTGGAGAGAA
ZNF676_3	CAACTCGTCCTCAAAGCTCA
ZNF676_4	CACGTTACACTCATCCACAT
ZNF676_5	GAGAAACCCTACAAATGTGA
ZNF677_1	AAGCAAGGGATTATCACCAA
ZNF677_2	GGAGGGAATGGCTCTTTCTC
ZNF677_3	GTTCCACAAGACTTGAACTT
ZNF677_4	TATCCTCATCGAGAGAAAGC
ZNF677_5	ACTACAGAGATTTCAAACTG
ZNF678_1	GAGAACTGTGAATGAAGGTA
ZNF678_2	TCTCAAGCCTTACTCAACAT
ZNF678_3	CTGAATGCCAGTAGTCCCTG
ZNF678_4	GCTCACACCTAACTAGACAT
ZNF678_5	GCTTTGCCACATTCTTCACA
ZNF679_1	TTATGCCTAGCTCTGGCGGA
ZNF679_2	GAGATGGTAACCAAACACCC
ZNF679_3	GCCGCATTCTTCACATTGGT
ZNF679_4	GTAAAAACATGTAAAAGCAT
ZNF679_5	TGTAGTCATAGAATTCTCTC
ZNF680_1	GAGTCTAAGGTGTTCAAAGA
ZNF680_2	TCCAAACAGGTTATCAGGTG
ZNF680_3	AAGGATTGAGAATAAACTAA
ZNF680_4	AATGCCTGGACACTGCACAA
ZNF680_5	TTTCAAAAAGTGATACTGAG
ZNF681_1	CTTGGACTAGAAAGAGACAT
ZNF681_2	GACTAGAAAGAGACATAGGA
ZNF681_3	TAGAAAGAGACATAGGATGG
ZNF681_4	AAGGTGTGAGGACTGCCTAA
ZNF681_5	GAGTGCAAAGTGCAAAAAGG
ZNF682_1	AAAGGGTGAACACCAGTGAA
ZNF682_2	CATTCATCCAACCTACCTGG
ZNF682_3	GTATAGTAAGTAGTGAGCAG
ZNF682_4	TCATTCATCCAACCTACCTG
ZNF682_5	AAAGTCTTTAAATCTCACTC
ZNF683_1	AAGTGTCCTCGACATCCCAG
ZNF683_2	ATGGCCCTGGGAGGTACAGG
ZNF683_3	CAGCAGGCTAGGCATAGCCA
ZNF683_4	TCTAATGTGCTAGATACCGT
ZNF683_5	GCTTAGAGCACGAGCCACCA
ZNF684_1	GGCTGTGGATTTCACTGCAG
ZNF684_2	TATGCATAAAGCCCAACTCG
ZNF684_3	TCACCTGGAGAGCTCTCGTG
ZNF684_4	TGGATTTCACTGCAGAGGAG

ZNF684_5	ACCCACTTGTTGATGAACCA
ZNF687_1	AAACCATTCTGCATTCGATG
ZNF687_2	AAGGTGGTGAGCGTACAGTT
ZNF687_3	CAAACACCACTGAACAGCTG
ZNF687_4	TGGCACGGCACTATGACCGT
ZNF687_5	CACGTGAACACAAGGACAAG
ZNF688_1	AGTGCCGGAAGAGCACAGGC
ZNF688_2	CCACAGGCGCATGCACTCGG
ZNF688_3	CTTCAAGAGGAAGTTCGCAG
ZNF688_4	TCGCAGTGGAAGCGCACCAG
ZNF688_5	AAGAGCCAAAGGGCCTAGAA
ZNF689_1	ACTGTGGGCGTGCCTTCCGG
ZNF689_2	ACTGTGGTCGCTGCTTCCGG
ZNF689_3	GATCCGCAGGAGTACCCGAG
ZNF689_4	TGCGCAGGGAGCGGAGCCGA
ZNF689_5	ATCCGCAGGAGTACCCGAGA
ZNF69_1	ATTAAAGATGACAGTCATTG
ZNF69_2	CTTTGCTGGATATTTCCCAG
ZNF69_3	TAGTCACAGGAGGTGTAGAG
ZNF69_4	TATGTCATCCTTACCTACAG
ZNF69_5	ACACGTGGTAATGCACAGTG
ZNF691_1	AAAGTTGGAGATATCGCTGA
ZNF691_2	AGTGCCAGGAGAGCTTTCGG
ZNF691_3	CCAACCTGAGAACACACCAG
ZNF691_4	TGTAGTGTTTCTCTTCCAGG
ZNF691_5	ACGCACCAGCAAGATCACCT
ZNF692_1	AGAAGATCCTTCCACAGCCA
ZNF692_2	GCAGGACACGTCCACCGCCG
ZNF692_3	GCAGGCCATACCTTCTCCTG
ZNF692_4	GGTGGGAAGGTCTCTGGTGG
ZNF692_5	AGTCCTACCTAATTCTCTTG
ZNF695_1	GGCTCTAGAATTCTCTCCAG
ZNF695_2	GTACAGCAAGTTGTGCAGAG
ZNF695_3	GTTTAGAAAGGTGTGAGCTC
ZNF695_4	GTTTAGTAAGAACTGAGCAC
ZNF695_5	AGAACTGATCATCTGTCTGG
ZNF696_1	CGAACGTGGTCCGGCACCGG
ZNF696_2	GAGCCAGGAGGAGAGCCCAC
ZNF696_3	GCCGGACCACGTGCGAGCTG
ZNF696_4	TGTGCACGCGCTGGTGCCGG
ZNF696_5	AAGGGGCGGCCTTTCCCGTG
ZNF697_1	AATGCGCTGGTGCTGCAGGA
ZNF697_2	GCAGTGCCCGACATCTGCAC
ZNF697_3	GGAGATGGGCTCCAACCCAC
ZNF697_4	TCTCGTCGTCGTCCTCCCGG
ZNF697_5	AGAAGGCGTTTCTGTCCGTG
ZNF699_1	AAAGTAGGCAGGACAAACAA
ZNF699_2	AAGGGATGAATGATCCACGA

ZNF699_3	ATAGGGCTTCTCTTCAGTGG
ZNF699_4	TTTATGGGAGAGCACCGGGA
ZNF699_5	CAAAACTCACTGTACATGGG
ZNF7_1	AAGCCGTGAACTACAACCAA
ZNF7_2	GGCTGTGCACTTCTCTCGGG
ZNF7_3	GGTTCCCAAGACCTTCACCA
ZNF7_4	TGTAGAGGGCCCTCTGGCCA
ZNF7_5	AGTGGTCAGAATCTCCCCAC
ZNF70_1	AATGCGGGAAAGATTTCAGC
ZNF70_2	ACAGGTTAGAGTCACAACAA
ZNF70_3	ATGGACTGGGCTCTTCACTG
ZNF70_4	GCTGGATCAGCGCAGAGCTG
ZNF70_5	CCTATGCGTGTCGAGAGTGT
ZNF700_1	AAGGGAATTGCAACATCTGA
ZNF700_2	AATGGATGGGCGATACCTGA
ZNF700_3	TATACACGCAAGGACTCATA
ZNF700_4	TCACAGCTTCGAGTGCACGG
ZNF700_5	AGACATGAAAGAAGTCACAT
ZNF701_1	GCATGCCATAGATGTCACAC
ZNF701_2	TTGGCCTACAGGAAATTCGT
ZNF701_3	ATGTTCTCATCAACAGGACA
ZNF701_4	CATCCCAACGAATTTCCTGT
ZNF701_5	GTGCACATATTTCACCCCGA
ZNF703_1	CCAAGGACACCCGAAAGCAG
ZNF703_2	GAGCCACTTATCCACAGCGG
ZNF703_3	GGTGTCCCTCTTGCCACCGG
ZNF703_4	TTACTGGTATCCCAGCGCCG
ZNF703_5	AAGATGCTGAGCGCTCACAC
ZNF704_1	AAGCTGTCAGCCGAGAGCGG
ZNF704_2	AAGGTGCTGAGCACTGCAGC
ZNF704_3	ACGGGAGGACTTCGAACCAA
ZNF704_4	GGTGAAGCCAGGGACTGGGA
ZNF704_5	ACATCCGAACCATCCATCTG
ZNF705A_1	AAGGCGAAAGCAATTAGTAT
ZNF705A_2	AATTTGTTTATGTGGTTTAG
ZNF705A_3	CAGCGTTTGTTAACTCACAG
ZNF705A_4	GGAAAAGAGCTGTGGCGGGA
ZNF705A_5	TAACAAACGCTGAGTTATTG
ZNF705B_1	ATCTCTGTACAGCTTTCTTT
ZNF705B_2	CCCGAATCATTATATTCAAA
ZNF705B_3	GAATTTGTTTACGTGGTTTA
ZNF705B_4	GCAAGGAAAAGAGCTGTGGT
ZNF705B_5	CAGTGTTTGTTAACTCACAG
ZNF705D_1	AAGGTAAAAGCAATTAGTAT
ZNF705D_2	CCAGGAAGAGTGGGACATGA
ZNF705D_3	CCCTGTGTCAGCAAACAGTG
ZNF705D_4	TAACAAACACTGAGTTATTG
ZNF705G_1	AATGTTCAGGGACTCACCGA

ZNF705G_2	CAGTGGACAAAAGATTACGA
ZNF705G_3	GCAAGGAAAAGAGCTGTGGA
ZNF705G_4	TCAGTGTAATCTATGTGAAA
ZNF706_1	GAAACAAGGACATGACCAAA
ZNF706_2	GCTCTCAAAGTGCTGCTTGA
ZNF706_3	TCTGGAGGAAGTGGAGTCTT
ZNF706_4	TGAACATCAGCTAATTCTGG
ZNF706_5	CCTTACCCTACAGACAGTGC
ZNF707_1	AAGGACAGACGCCAAGCCCA
ZNF707_2	GGAACAGTGGGAGGAGCCGT
ZNF707_3	GTAGATGGCCACGTCCCTGA
ZNF707_4	TGTTCCCATGGACATGGCCC
ZNF707_5	CACCTGACACGGGAAAGCCG
ZNF708_1	AAGAGTTGAGGACTTGGTAA
ZNF708_2	AAGGTGTGAAGATAGGGTAA
ZNF708_3	CTTATGATTAGTAAGGTGTG
ZNF708_4	CTTATGTCTAGTAAGAGTTG
ZNF708_5	GATGAGCATAAGTTGCACAA
ZNF709_1	AAAGGAACGTGAGCAACTGA
ZNF709_2	AGGATTTGGAGTCTGACTGA
ZNF709_3	ACACATGAGAATGCACACAG
ZNF709_4	TATAGAGGCCAAGTTAACAA
ZNF709_5	TCATGACTTCGAAAGGATGT
ZNF71_1	CAGCTGGCCAGAGAGGCCGC
ZNF71_2	GAGGTGCATGTTCTGGCTGA
ZNF71_3	GCACAGTGAGGTTCATGCGC
ZNF71_4	TCTGCAAGAAGCACTTCACG
ZNF71_5	AGCCATTGGGAATTCCCCAG
ZNF710_1	AGGGACACAGACGGACGCCG
ZNF710_2	CCTGTGAGAAGCACACCCGG
ZNF710_3	GAGAGTCTGCTTCTGTACAA
ZNF710_4	GAGCGTGGACAGCAGCGCCG
ZNF710_5	TGACACGGCGGGTTCGACCG
ZNF711_1	ACAGAGAGTGAGTACACCAG
ZNF711_2	CTAAGTATGGATTCAGGCGG
ZNF711_3	GATGGGATAACTCTTGATCA
ZNF711_4	TATGCCGCAGCCCAGACAAC
ZNF711_5	ATGGCAAGGGTACACTGTGA
ZNF713_1	AATGAATGATGGCTCACAGA
ZNF713_2	GATAGAAAGAGACAGCCTGC
ZNF713_3	GGCCGTGGACTTCACCAGAG
ZNF713_4	GTTGAATGAGATGTATCCTC
ZNF713_5	CCCCTGAATTCTGACACACA
ZNF714_1	AAGAAAAGGCTCCGCAAATG
ZNF714_2	CTTTGTCACATTCTTCACAT
ZNF714_3	GAATATGAAGATATGTGAGA
ZNF714_4	GTGTAGTAAGGGCTGAAGGG
ZNF714_5	CAAGTGATACTGAGAAGACA

ZNF716_1	CAAGACCTTCAGTCAGAGCA
ZNF716_2	CATTTCAAATGTAAAAACGA
ZNF716_3	GGTTAGTAAGTGTTGAAGAG
ZNF716_4	TGTAGTTAGTAAGTGTTGAG
ZNF716_5	CCCTACACTTGTGAAGAATG
ZNF717_1	AGGACCTGGATGATGCTCAG
ZNF717_2	GGAGTTGGTGTCCTTTGAGG
ZNF717_3	GGTAGCTGTGCACTTCACCT
ZNF717_4	GGTTTGGTAATGTAATGCCC
ZNF717_5	AGGGAAAACCTTCAACACGG
ZNF732_1	CAGAACTGAGGACCTACTAA
ZNF732_2	CAGGTATGCAGACCATCCAA
ZNF732_3	AAGAAAAAGCTGTAAAAGGA
ZNF732_4	CATCTATCTGGAGCAAAGAA
ZNF732_5	TCAGACCTAACTCAACATCA
ZNF736_1	AATGTGGCAAAGCATCGAGC
ZNF736_2	ATTTCTCTTGTGTTTAACAA
ZNF736_3	GTTAGAGAACTATGGAAACC
ZNF736_4	TAGCTGACAAACATTGATGA
ZNF736_5	TTGGAAAGTGAAGAGACAGG
ZNF737_1	ACATAAGAAAATTCATACTG
ZNF737_2	CTTATGCGCAGTAAGGATAG
ZNF737_3	GACCATGAAGAAACATGAGA
ZNF737_4	TATGTGTAGTAAGGACAGAG
ZNF737_5	AAGGTATGAAAACCGGCTAA
ZNF74_1	ACAGCCACTCACCTTGGACC
ZNF74_2	CAAGTGCAGCGCCTGCGAGA
ZNF74_3	TACCAGAACCTTCTTGCCCT
ZNF74_4	TCTTCCCGAAGCCAGGTCCA
ZNF74_5	GCGGTGCCCTCTCAACAGCA
ZNF740_1	AGACACAAACGGATGTGCCA
ZNF740_2	GCCAGTCCAAGACTTCCGAC
ZNF740_3	TAGGTGGTAACTGCTCCGAA
ZNF740_4	TGCCAGTCCAAGACTTCCGA
ZNF740_5	GAAAAGACAGTGATAAGTCC
ZNF746_1	CGGAGCAGGAGACATCTCCA
ZNF746_2	CTACGCCATCTCCAAGCCCG
ZNF746_3	GTTTCTGAAAGATTTCTCCG
ZNF746_4	TACCGGAGCCGCGACCGCCT
ZNF746_5	TGAGTGTCAGGCCTTACCCA
ZNF749_1	AACACAAGAGGATTGACCTC
ZNF749_2	AATGATTTCAACTCCAGCCA
ZNF749_3	AGAGGATTGACATTAGGCCA
ZNF749_4	AGAGGATTGACCTCAGGCCA
ZNF749_5	GCTGGAGAAAGGCCTTATGA
ZNF750_1	GAAAGACACGGCCTTGGCAG
ZNF750_2	GCAGAAGCAGACGGCAGCCG
ZNF750_3	GGAAGAGGCTGGCTTCGCCG

ZNF750_4	GGCTCCAGACGCATGCGCGG
ZNF750_5	CAAGGGGCCAGACAACGCCG
ZNF75A_1	AAGCACTTAACAACACACCA
ZNF75A_2	ATTGTAGAGGGCCTTCTGAG
ZNF75A_3	GCAGGAGAAACTTCAGCAGG
ZNF75A_4	TATGCAGGAGAAACTTCAGC
ZNF75A_5	AAAGAAAGAAACTTTCAACC
ZNF75D_1	AATGTCAACAATGTGACAGG
ZNF75D_2	CCTGGTGGAATTCTTGCAGA
ZNF75D_3	TAGGTCACAGCCCATGAGCT
ZNF75D_4	TTGTAGAGAGTCTTCTCAAG
ZNF75D_5	AGTTGGCTGATAGTCTCAAG
ZNF76_1	CACACGGTGACCCTTAGTGA
ZNF76_2	CACCTGGTGAATGTATGCGG
ZNF76_3	GCCAGGATGGTGCTCTCCGA
ZNF76_4	TACATACACCGCACACCCAG
ZNF76_5	GAGCTTGTATAAGCACCACG
ZNF763_1	ACACAAGAAAGGAATCACAC
ZNF763_2	AGACGCATGGTAATGCACAG
ZNF763_3	CTTTGCTGGATATTTCGCAG
ZNF763_4	TGAACATCAGAGGTGACATT
ZNF763_5	AATATGAGTACCAAAACCCC
ZNF764_1	CAGACACAAACGGACCCAGG
ZNF764_2	CTTTGGCCAGAAGTCAGCCG
ZNF764_3	GACCCGAGTGGAGGGAGCCG
ZNF764_4	GGCCACACTCCAGACAGCGG
ZNF764_5	CTGTCCAAGGCACCGCACCG
ZNF765_1	AATAGAGAAGTGTTCCATGC
ZNF765_2	ATAGAGAAGTGTTCCATGCA
ZNF765_3	AAGGTACGACTTCTGACTAA
ZNF765_4	GAGTTCTCGTCAACAGCACA
ZNF765_5	TAGGTAATATGTCTGACTGA
ZNF766_1	GAGGCCTGACTTGTCCCTGA
ZNF766_2	GTGGAGATATGGCGCAACTG
ZNF766_3	GTGGCGAAACTGAAGAACTG
ZNF766_4	TAAAGACCTTACCACACACA
ZNF766_5	ACTTCAAAGAATTTACTCTG
ZNF768_1	GAGGTAGGAGCTGTCGCCGA
ZNF768_2	GGAGCGGGAGGCGTTGCCGT
ZNF768_3	TCTGCGGGAAGAGCTTCGGG
ZNF768_4	TGGAGCGGGAGGCGTTGCCG
ZNF768_5	ACTCCTACGGAGATATTCAA
ZNF77_1	AAGCATGTGAGATCACACAG
ZNF77_2	AAGGGACGAGGAACAACTGT
ZNF77_3	CCAGTGGATCAAGTTCTCAG
ZNF77_4	GAACATGGGAGAACGCACAG
ZNF77_5	ACATGTTCTCGAAAGTATGA
ZNF770_1	AACTGTGAGGTACTTCAGTG

ZNF770_2	ACAGGTCAGAAGATTGCTGG
ZNF770_3	ATGGGATCAGACTTAGAGCA
ZNF770_4	TGGGATCAGACTTAGAGCAC
ZNF770_5	ACAAACCCAGAGAACATTTG
ZNF771_1	AGCCGGCGCAAATATACAGG
ZNF771_2	GCCTGGCGAACAGCAGGCAG
ZNF771_3	GCTCCGCCAGGTTGGAGCGG
ZNF771_4	TGGAGCGGTGGCCGAAGCGA
ZNF771_5	AAGTCGGCGCTGACCAAACA
ZNF772_1	AGCACCAGGAAACACACCCA
ZNF772_2	GTCCACAGGCCTGATGGCGG
ZNF772_3	GTTGCCACAGAGACTCCAGG
ZNF772_4	TCACAGATCAGGATTCCCAA
ZNF772_5	ACCATGCCCCTCACAATGAG
ZNF773_1	ACACGGGAGAGAAGTCACAT
ZNF773_2	GCAAGGACTTCTCTGATAGG
ZNF773_3	GCACCAGAGACTGCACACTG
ZNF773_4	GTGACTTCAGCCATACTGAG
ZNF773_5	CAACACCAGAAGCAGCACTG
ZNF774_1	CGGAGCAGAAAGAAGAGCCA
ZNF774_2	GAAATGGGAACGCTGATTGA
ZNF774_3	TAAGAACCCACACTGGCGAG
ZNF774_4	TAAGCACCAACGAACCCACA
ZNF774_5	AGAAGCCCTACCAATGCAAG
ZNF775_1	GCGGCAAGAGCTTCTCGTGG
ZNF775_2	GCTGCTTCTGGCGGAAGCCG
ZNF775_3	GGCGGGCTCAGCGACAGCGT
ZNF775_4	TACAAAGTGACCCTCGCCGG
ZNF775_5	AACATATTTCAGCAGCACCG
ZNF776_1	ATCGTACAGAAGCAATGCCA
ZNF776_2	CTGGGAGATATCTTACACCA
ZNF776_3	TAGGAGCAACTCCCACCTAA
ZNF776_4	TCGTACAGAAGCAATGCCAA
ZNF776_5	TACAACAGGAGTCCCCACTC
ZNF777_1	CAAGCGGGTGATCTCTGCCG
ZNF777_2	CGAAGGCTTCTCCTTACCCA
ZNF777_3	GGAGCAGGAAGACTCTGAGG
ZNF777_4	GGAGCCAGAGAACACGCTGG
ZNF777_5	AGCGGGTGATCTCTGCCGTG
ZNF778_1	ACACATGCGGACACACACCG
ZNF778_2	CAGGCAAGAAGCCACAATGG
ZNF778_3	GACTGTGGGAAAGCCTGCGG
ZNF778_4	TCGCGTGCAAGTCCACCCTG
ZNF778_5	ATGTGTGATGATTTAGGACG
ZNF780A_1	AAGCAGGGTAAGAAGACTAA
ZNF780A_2	GAAGCATGAGGAGTATGAGT
ZNF780A_3	AAATTGTATGTGAAGTCGAA
ZNF780A_4	AGACAATTTGAGGGACTACA

ZNF780A_5	GTTCTTATGGCGATTAAGCA
ZNF780B_1	AAGCTGGGTGTGAAGACTGA
ZNF780B_2	AAGCTGTGTCAGAAGACCAA
ZNF780B_3	AAGTTGCTACTGATGTCTGA
ZNF780B_4	GGAGAAGCATGAGTATAAGC
ZNF780B_5	AAGCTGATTGAGGAGACTAA
ZNF781_1	AAGGTGGGCACGCTTTCTAA
ZNF781_2	AGGTGGGCACGCTTTCTAAA
ZNF781_3	AATGTAGAAAGACGTTTAGG
ZNF781_4	ATAATCGAATTCACACTGGT
ZNF781_5	CAGCATCAGAGAATTCACAC
ZNF782_1	AACATCAGAAAGCCCACCCA
ZNF782_2	ACATCAGAAAGCCCACCCAG
ZNF782_3	ACCAGAACTGATCTTCACAT
ZNF782_4	TGAATTCAGTAACTCACCTG
ZNF782_5	ACTCACAAAACTCTCACAGG
ZNF783_1	AGGAGCCTTGTCTTGACCGG
ZNF783_2	CACATGCAGACCCACGCCCG
ZNF783_3	GAACTCGTACCTCTACTCCA
ZNF783_4	GGAAATCACACTGTGGACGG
ZNF783_5	CACAGCTGTCTTCTCGCAGT
ZNF784_1	CAGCTCGGAAACCAGCCGGA
ZNF784_2	GAAGGTGACGGACACCACGC
ZNF784_3	GCAACGGCCGCTGTGTCCGG
ZNF784_4	TGGAGCCGGGCACCTCTCGG
ZNF784_5	AAGGCACAGTGGAAAGAACC
ZNF785_1	AAAGTGCCGGAAGTGAACTG
ZNF785_2	AATGCCTGCGGCCAGCGCAG
ZNF785_3	AGCTGCAGAGCTCTCACCGT
ZNF785_4	AGTGGAGGCGTGGAGCCCGG
ZNF785_5	ATGAAGTGGCTGTCAAGGAG
ZNF786_1	AATGGCAGGATCTAGAGGCA
ZNF786_2	ATATCCTGGATTGAACACGG
ZNF786_3	GGAAGTGCCTCTGTACCCGA
ZNF786_4	TACCCGAGGCGGCTCCGCCA
ZNF786_5	CATTTAGTAATGCACCAGAG
ZNF787_1	ACACAAGAAGATCCACGCGG
ZNF787_2	ATGGCCAGTCACGAGAACCC
ZNF787_3	GAGACACAAGAAGATCCACG
ZNF787_4	GGGAAGAAGCCTGGTCTCCG
ZNF787_5	AGAGCCTCGCGCGTCACCTG
ZNF789_1	AAGCACCAGGGCACTCACAA
ZNF789_2	AAGTCATTAGGCGTAAGGCA
ZNF789_3	AGTGGTGATGAATACAGCAG
ZNF789_4	GAGATCCACATTCCTACCAA
ZNF789_5	AGAGTCATATAAGATATCAG
ZNF79_1	CAAACCTCACAAACCATCAG
ZNF79_2	CAAACCTCACGAACCATCAG

ZNF79_3	TCCACGGGACAGGTTCAAGG
ZNF79_4	TCCCAGTTGGAACAAAGGGA
ZNF79_5	CAGGGGGCATCTCTACACAT
ZNF790_1	AACATAAACAGTCAAGGCTG
ZNF790_2	AGTGCCTGGACCTGGAACAG
ZNF790_3	GAGACAAGAGGACCTTGCCC
ZNF790_4	TGAGAGAGAAATAGCCCAAT
ZNF790_5	ATCAGAATGTTCATGTTGGT
ZNF791_1	AAAGGGCTGGTGATATATGA
ZNF791_2	AATGGAGGTGGAACATCTGA
ZNF791_3	ACGGGAGAGAGACTCTGTGA
ZNF791_4	TAAGGATGTGGACACACTGA
ZNF791_5	CATCCCTTACTAGACACATG
ZNF792_1	ACACAACCCTATCAGAACGG
ZNF792_2	ATGACATCAGCCATGGCCAG
ZNF792_3	TGTGAGGTCACTCCCAGCGA
ZNF792_4	TGTGGATATGACATCAGCCA
ZNF792_5	CAACCTCATTCAGCATAAGA
ZNF793_1	AAGAGAGAATCACTTACTGC
ZNF793_2	ACCGGCTGAGTCCTGCTCAG
ZNF793_3	GGTTGTGGGCTTCACCCAAG
ZNF793_4	TCTGGCGAGTTAATATCCAG
ZNF793_5	CCTAGTAACTGGAACCCTTG
ZNF799_1	AGACACATGGCAGTGCAGCG
ZNF799_2	ATGAGTGGAGAAGTCATCAT
ZNF799_3	CATGTGTTTGAAGTGAGTTG
ZNF799_4	CTCACAAATGCAAGATATGT
ZNF799_5	AGTTGTTTCGTGTCTTCGAA
ZNF8_1	GCACCAGCGGAAGCACGCGG
ZNF8_2	GCACCTGAGAACTCACACCG
ZNF8_3	GCTGAGAGAGGAACCACCCA
ZNF8_4	TGGAGCAAGGGACCGAGCTA
ZNF8_5	ATTCCCAGAGATCTCTAGAG
ZNF80_1	AAGATGAGCCCTAAACGCGA
ZNF80_2	AGCACAGGAGGTGCGACCTG
ZNF80_3	GAAGACAGAGTGATAGCTGA
ZNF80_4	TTCATGGAGATTGTCTCCTG
ZNF80_5	AAGACACTTTGGTTCGTGAG
ZNF800_1	AAGTCAGCCTGCGACTCCTG
ZNF800_2	TCCAGGAACAAAGGTTGCCC
ZNF800_3	TCTGTAACAATCTCAACAGG
ZNF800_4	TTATGCCGCAGTCTCTTCAG
ZNF800_5	AAAGAATTCAATTCTAGACG
ZNF804A_1	AAAGGGATGACAGTAGGGTG
ZNF804A_2	AAGGAAGGAGATTGTTAGGG
ZNF804A_3	AGCTCCACGCATCTCAGCAA
ZNF804A_4	TAACGAGGCATCCACAACTG
ZNF804A_5	AAGCAGGAAGACATTTGCAT

ZNF805_1	ACAGGGTCCCGGTATGGCGA
ZNF805_2	ACCAGGGTTAGATTCCCAAA
ZNF805_3	AGACAAGGCTAGCTCACAGG
ZNF805_4	GAGGTATGTACTCTTGCTAA
ZNF805_5	AAAAGAGATTCCTTCAGACA
ZNF808_1	AAGGGATGCCCGGTGACGGA
ZNF808_2	ATACAAGGGATGCCCGGTGA
ZNF808_3	GGCATAGAAGGGATGACCTG
ZNF808_4	GTCGCAAGACCTTCAGCCGC
ZNF808_5	CATCCCAAAGAATTTCCTGT
ZNF81_1	GAGCAAAGAAATGTGACCTC
ZNF81_2	GTTCGAAGTTCCTAAACCAG
ZNF81_3	TTGGAATTAAGCCTTCCCAG
ZNF81_4	TTGGGAAGATCTTCACCCAG
ZNF81_5	ACACATTTGCTAAGCTCATG
ZNF813_1	AGACATGAAAGTCATCACAC
ZNF813_2	CCATCGTAGATGTCACACTG
ZNF813_3	GTTACTCACACAAAAACAGG
ZNF813_4	TCCACATGTTTCAGACCCAA
ZNF813_5	AATCAAACCTTAAAAGACAT
ZNF814_1	AGCCATCAACGAGTTCACGC
ZNF814_2	GGAGAGCCTCAGCGTAGCCG
ZNF814_3	TGGCTGCCGCGGCTACGCTG
ZNF814_4	TTGTACACCAGAGTTCACAC
ZNF814_5	ACTGAAAAAAAACATGAATG
ZNF816_1	AATGTGGCAGGAGTTTCAGT
ZNF816_2	GTAAAGTTTCCCTACACCCA
ZNF816_3	GTACAGACCGAAGTGATCAC
ZNF816_4	TCTTTGGGATTCTGAAGCTG
ZNF816_5	TAGGAAGATTCATACTGGAG
ZNF821_1	ACTGGCAGAGTTCAAGCAAG
ZNF821_2	ATCAGGGAAACATGTCCCGT
ZNF821_3	TAGCTTGTCGCTGACTGCCA
ZNF821_4	TTTACAGCACACTGCAGCAG
ZNF821_5	ACACAGCACAGTTAGATCGC
ZNF823_1	AAATGGGAGGACCAGAACAT
ZNF823_2	ATCGAAGGGTTTCTTTCCGG
ZNF823_3	GAAGGAGTGCTGATGACTGA
ZNF823_4	GTGCCGCCATGTGTCTTCGG
ZNF823_5	AAACCTCCGAAGACACATGG
ZNF827_1	ACACAAAGAAGACACTCCCG
ZNF827_2	GAGAAGGGATGACTTACGTG
ZNF827_3	GAGGACAGAAGCCAAGGTGA
ZNF827_4	TAGGTGGCTTACCTTCATGT
ZNF827_5	ACTGTTGGCCACATACTCGG
ZNF83_1	AATCATCATGCGATCCACAC
ZNF83_2	ATTGGTAGAAGAGACATGCA
ZNF83_3	GGATCGCATGATGATTAGTG

ZNF83_4	GTTCTGGTAGATGTGACTGC
ZNF83_5	ACAGTAGAAGAAATACGTTG
ZNF830_1	AGCAGATAGAGTGTTACCGA
ZNF830_2	GATGTGGAGGGCTGTACCTG
ZNF830_3	GGCGCGAAGGAAGCCAGCCA
ZNF830_4	TAAATCAGGAAGAATTGCGG
ZNF830_5	CCAAGATGTCAAGAGAGCGA
ZNF831_1	AATGGAAGAAAGGCCTGCCT
ZNF831_2	ACAAGGAGGTGATTCTGAGG
ZNF831_3	TGAGGCGGACAGCATCCTGG
ZNF831_4	TTCTGAAGAGGTCTGCCCAG
ZNF831_5	AGCACAGCGCCGAGTCCGAG
ZNF836_1	AGAGAGAAACGTTACCAATG
ZNF836_2	GAGGCATGAGCCTTGTTTGA
ZNF836_3	GCCTGACGTACCATCAGAGA
ZNF836_4	TCGAAGTGGAATTAAAGACC
ZNF836_5	ATCAGATAGTCCATACAAGG
ZNF837_1	CAGGCCGGAGCAGCGCACGA
ZNF837_2	GAGGTGCGAGAACAGCCCGA
ZNF837_3	GGAGAGGCTGCAGGCGACGG
ZNF837_4	TAGGAGCCCAGCGCGTGGCG
ZNF837_5	CTGAGCTGGTTATCCCCGAG
ZNF839_1	AGAGCTGCTCAGCTACCTGG
ZNF839_2	CAACAGGCCGCCCTGCAGCG
ZNF839_3	CCAGTGGAAGCACCCTCCGG
ZNF839_4	GAAATACACCCAGACAACCT
ZNF839_5	CAGCGAGAAGAGGGAACGTG
ZNF84_1	AAGACTTGACTTCTCACCAA
ZNF84_2	AGAGACATAGCCTTTGGCTG
ZNF84_3	ATTTCTCCATCTCCTACCCA
ZNF84_4	GAAGAGCCGTGGGTAGGAGA
ZNF84_5	AAGTAGATGGTAACATGATG
ZNF841_1	ATAGCGAGGAACCCAAACTG
ZNF841_2	CCTCACCAAATTCATTACAG
ZNF841_3	GTGGCAAGGCGTTTGGGCGG
ZNF841_4	TAGCGAGGAACCCAAACTGT
ZNF841_5	AAGAGTTCGACATAGTCAAG
ZNF844_1	AAGGTTTCTGACAGATCCAA
ZNF844_2	ATACATGCAACAATGCACAG
ZNF844_3	ATATATGCTGAGACAAGGGC
ZNF844_4	GTACATGCAAGAACACACCC
ZNF844_5	ACCTTATAAATGTAAGACTG
ZNF845_1	AATGCAGCAGGACCTTTAGT
ZNF845_2	GAAGTCTACGATGGTACACA
ZNF845_3	TCCACATGTTTCAGACCGAA
ZNF845_4	TTGTAGCATTACTAAAGACT
ZNF845_5	CAATATAAATGTGATGTGTG
ZNF846_1	GATCATACAAGAATCCACAG

ZNF846_2	GCAGAAATATCTTGCAGCAG
ZNF846_3	GGAGTGACAGGAGTTCTGCA
ZNF846_4	TCTGCAGAAAGATCACCAAA
ZNF846_5	ACAAGAAAAGTCATGCTGAG
ZNF85_1	ATCATGGTGGCCAAACCCAC
ZNF85_2	GAAGAGACATGAGATCATGG
ZNF85_3	AGTATGAATTACCTCATGTG
ZNF85_4	CCTTTCAAATGTACAAAATG
ZNF85_5	GATGAGTGTAAGATGCACAA
ZNF850_1	GAGTCCTGAGAAACGGACAA
ZNF850_2	GGACACCAGGCAATTCACAC
ZNF850_3	GTGCTGGGAGGATGGTGCCG
ZNF850_4	TGAGTGTCAGGAATCCGTGA
ZNF850_5	CAGCACTACTTCAACATCAG
ZNF852_1	GATGACCGAGCCAGACCTGA
ZNF852_2	GGCGTATGAGGACCTGTCTG
ZNF852_3	TCGGTCATCTTCTTAAGGCA
ZNF852_4	TCTGAGTATAGTCCTCAGAC
ZNF852_5	CCTGCACTGGAACATGATGC
ZNF860_1	GCAATACAGAAGTGGACACA
ZNF860_2	GGCAATACAGAAGTGGACAC
ZNF860_3	ATGCCTTCGATCATATCTGT
ZNF860_4	GAATTCTAGTGTGTTGTGTG
ZNF860_5	TCTTTGGGACGTTAGAACTG
ZNF865_1	AGACAGCGAGAAGGCGGCGG
ZNF865_2	GAAGCCGAAGGTCTTGCCGC
ZNF865_3	GGAGATGGAGGCGAACCCAG
ZNF865_4	GTAGCGGAAGGTCTTGCCAC
ZNF865_5	AGGTCCACGATGCCCCACTG
ZNF878_1	CAGGTACTTGACAGATCTGA
ZNF878_2	TCATGATATTGAAAGGAACT
ZNF878_3	TGGAATAACTAAAGGCTCTG
ZNF878_4	TGTTCACAGAAGACTTATAG
ZNF878_5	TTCCCAGAAAAATCTCTACA
ZNF879_1	AAACCGAGAATAGTTTCCAG
ZNF879_2	AAGAGATGTGGTGAAACTGA
ZNF879_3	AAGCCGTGAAATCCAAGAGA
ZNF879_4	ACTCACCGAGATGTGCGCCT
ZNF879_5	AAGCCTTCACTTCAATATCG
ZNF880_1	GAATTAGTAGATGGGCAGTA
ZNF880_2	GCCCATCTACTAATTCACAC
ZNF880_3	TAATCTATGCCTGAAGACCT
ZNF880_4	TAGCAAACAATCCAGGTGGC
ZNF880_5	CCAACATGGAGATAACACTC
ZNF891_1	AAACAGCATAAGATTCCAGG
ZNF891_2	AAGGTAAGAGCTTGTGCCAA
ZNF891_3	CTATAGAAATCTCACCTCCG
ZNF891_4	GTACAACTGATATTCCACGG

ZNF891_5	AAAACAGCATAAGATTCCAG
ZNF90_1	AAGGACTAAGGAGCGCCTGA
ZNF90_2	AAGGACTGAGGAGCGCTTGA
ZNF90_3	AAGGATTGAGGAGAGCTTGA
ZNF90_4	AAGTGTTGAGGAGCGCTTGA
ZNF90_5	CTTATGTACATAAAGGATCG
ZNF91_1	GTTTAGCAAGGGCTGAAGAA
ZNF91_2	AAACATAAAATAATACATGC
ZNF91_3	AGAGCAAAGTATTTCAATGT
ZNF91_4	GAGATGGTGGATGAACCCAC
ZNF91_5	GTTTAGCAAGGGTTGAAGAA
ZNF92_1	AAACCCTGCAAACATGAATG
ZNF92_2	ACATAAGATAATCCATACTG
ZNF92_3	CCTTTCAAATGTAAAAACCG
ZNF92_4	GAGATGGTAGACAAAACCCC
ZNF92_5	GATGCATGTAAGGTGTACAA
ZNF93_1	AAGGATACGGGAGTACTTAA
ZNF93_2	TAGGACTGAGGACCAAATGA
ZNF93_3	CTTATGTCTAGTTAGGACTG
ZNF93_4	GCCACATTCTTCACAAACGT
ZNF93_5	TCTGGGTAGTTGTACTACAC
ZNF98_1	ATGTGGGAAAGCCTATAATG
ZNF98_2	CCTATATAAATTCTGCTGTG
ZNF98_3	CTGAGAACATATAAAAAATG
ZNF98_4	GAGATGGTAACTGAACCCCC
ZNF98_5	TGGAGCAAGGAAAAGAACCT
ZNF99_1	ATCTACAAATGTGAAGAACG
ZNF99_2	CAATGTTGGACAACTACCCA
ZNF99_3	CCCTACAAATATGAAGAATG
ZNF99_4	GAGATGGTAACTAAACCCCC
ZNF99_5	CCCTACAAATATAAGAAATG
ZNHIT1_1	CAGAACTTGAGTGTGGCCGA
ZNHIT1_2	GAGCTGAGGGAGTCCCGCGT
ZNHIT1_3	GCAGAACTTGAGTGTGGCCG
ZNHIT1_4	GCTGAGGGAGTCCCGCGTGG
ZNHIT1_5	GAAGTTTAAAATGATCACCT
ZNRD1_1	ACGGTCACCTGTATTCGCTG
ZNRD1_2	GACAGCCATGCCTATGTCGG
ZNRD1_3	GCTCGGTCCTGCCTCTGCCC
ZNRD1_4	GGTGACCGTATCCTGAGCCC
ZNRD1_5	AGCTGGAGCAAGTATTGGCG
ZRANB3_1	AAAGGATCCTGACACTCGCG
ZRANB3_2	CAGGAACACCAGCTTTAGGA
ZRANB3_3	GAAGGGACCACTATTAACAG
ZRANB3_4	TGGGATGGTGTCTCTTCCGA
ZRANB3_5	ACCCTAGACACCCTTATGTG
ZSCAN1_1	ACACAGATGTGCCAGCAGGA
ZSCAN1_2	ACTGCCGGAAGCGCAGACGC

ZSCAN1_3	GAAAGGGCCCTTCCTCGCGA
ZSCAN1_4	GAGTCTGCACACCAGGGCGG
ZSCAN1_5	CCAGGACTGGAGTTTCGGTG
ZSCAN10_1	AGGGAAGGGTTGCTTACCCA
ZSCAN10_2	GAAGGGACCCATCTTCACCA
ZSCAN10_3	GAGGTGGTGCTGCTGCTCGA
ZSCAN10_4	TCAAAGCAGCCGCTGAGCCC
ZSCAN10_5	AGCCGGCTCAGGGACGCCCG
ZSCAN12_1	CAGGTCCCAATCCTGTCTGG
ZSCAN12_2	GAATAAGCACAGTTCTCCCA
ZSCAN12_3	GACGGAAGACCTCTCTGCTA
ZSCAN12_4	GGAACAGGAAATGTTCTTGC
ZSCAN12_5	AAACAACACCCATAGCAGAG
ZSCAN16_1	AAGGCAGAGGACCATTACTG
ZSCAN16_2	AGCTGGGTAAGAGCTTCACG
ZSCAN16_3	GAATGAAGAGTTGTTCCAGA
ZSCAN16_4	GATTCATATGGCCTTCCCAA
ZSCAN16_5	AGAAAGACGGGACATACTCA
ZSCAN18_1	ATCAGCGAGGCCCTCCACCA
ZSCAN18_2	GAAGCAAAGACCGAAGAGGA
ZSCAN18_3	TCATCCTCAATTCTTAGCGA
ZSCAN18_4	TGACGAGGACGCTACGGCGG
ZSCAN18_5	AGACAAATTCCCGGAAACGC
ZSCAN2_1	ACACCGGAGAACCCACATGG
ZSCAN2_2	AGCGCCGGCAGAGCTCTCGG
ZSCAN2_3	AGGCGGCCGAGTGCACCCTG
ZSCAN2_4	GAAAGTGCTCAGCACTCCGA
ZSCAN2_5	AAGTGCTCAGCACTCCGATG
ZSCAN20_1	GACATTGCTCCAGTGTCCGC
ZSCAN20_2	TCGCAGGAAGCCCTGGGCCC
ZSCAN20_3	TCGGTGCCAATCCTCCACCA
ZSCAN20_4	TGAGGGACAGTCCCAGAAGA
ZSCAN20_5	CCTGGGAGGAACAATACCAG
ZSCAN21_1	ATTGTGGCAAGGCTTTCAGC
ZSCAN21_2	CAAGATCCAAGAAAGGTCCG
ZSCAN21_3	CGGGCAATGCTCCTGCACCC
ZSCAN21_4	GATGACCAAGGTACTAGGCA
ZSCAN21_5	CAAACGAACAGAAACCGGTG
ZSCAN22_1	ACTCCCTGAGCCCAGTGCCG
ZSCAN22_2	ACTGACAGCACAGCGCTCGG
ZSCAN22_3	CAAGGGAAACACCTCCCATG
ZSCAN22_4	TCAGTGTAAGGTTTGTCCGA
ZSCAN22_5	AAGGGAAACACCTCCCATGA
ZSCAN23_1	AAGAGGAGGAACATTCCTGT
ZSCAN23_2	ACTTGGGTATAATTTGCGAG
ZSCAN23_3	GAAGAGGAGGAACATTCCTG
ZSCAN23_4	GCTGGAATAGGCCTGACCTC
ZSCAN23_5	ATGTGGAGTTAGCCCCAAAG

ZSCAN25_1	AAAGCAGTCTATCTGGGCTG
ZSCAN25_2	ACTGGAGAAAGAGTTGCCAT
ZSCAN25_3	GCGGGCGAAGCTTCAACCAG
ZSCAN25_4	TGGAGGTCAAGCCTGAATGG
ZSCAN25_5	ACTGACATCAGAGAGGTTTG
ZSCAN29_1	AAGGCCGGAGGTGCGCACCA
ZSCAN29_2	ACAGGAGCAGATGAACCCAA
ZSCAN29_3	CTTGGTCCGACACTGTTCCA
ZSCAN29_4	GTTGCGGTGACAGTTCCGGA
ZSCAN29_5	AGCTATCGGAAAGTCAAGAG
ZSCAN30_1	AGCATGGTAGGCCAAGACTG
ZSCAN30_2	CTGGAGAAACACATTCCCAA
ZSCAN30_3	GCCGGCTCAGAGCCTCCCGA
ZSCAN30_4	TGGCCAAGAAATGTTCTGGC
ZSCAN30_5	CTGTCAGTGGTTGAGGCCGG
ZSCAN4_1	CATGAGTCCTCATATGGCGG
ZSCAN4_2	GATCCAAGATTATTCTCGGA
ZSCAN4_3	GTACATCAGAAAGGATCCCA
ZSCAN4_4	TCAGCGTTTCAACAAAGCCA
ZSCAN4_5	AACCCTGTACTCACTAAGGC
ZSCAN5A_1	AGCCCAGTCAGTCACCCGGA
ZSCAN5A_2	GCAGGACTCAGATATCGAGA
ZSCAN5A_3	GGTGAACCAGATGCGTCCAG
ZSCAN5A_4	TCTGAGGATGCCATAGACCG
ZSCAN5A_5	CCCCCCAAAAATAGCCTCTG
ZSCAN5B_1	ACCGTGGAGTGTCTGACCCA
ZSCAN5B_2	ACGACAGGAACCCTGAGACT
ZSCAN5B_3	GCTGAACTCAGATGTCGAGA
ZSCAN5B_4	GGACTCGCTGGTGAACTCGG
ZSCAN5B_5	AATTCATGTGCCAAGTCTCA
ZSCAN5C_1	ATGGTGAAGTCACTGTGAAG
ZSCAN5C_2	TAAGGTCGCCGGCCGACCGA
ZSCAN5C_3	TGTCATGAAAGGTGACCCAA
ZSCAN5C_4	TGTGAACCAGATGTGTCCGG
ZW10_1	AAGAGGACAGAACTGATGGG
ZW10_2	GAAGTATATCCTTAGGCCGC
ZW10_3	GCAGGGCCTGATTACCCAGG
ZW10_4	TTGATACGTGAAGATCCCGG
ZW10_5	AAAGTGCCAGGATGTGATTG
ZWILCH_1	AACGTGGTGATATACAGCCA
ZWILCH_2	AACTGTCACATCTTACCCAA
ZWILCH_3	GATTGTATCATCCAAGGCAG
ZWILCH_4	GCGGCTGAACTGCGCAGCAG
ZWILCH_5	AAATGACATAGTATTCATAG
ZXDA_1	AGGAGGTCGGACCGCAGCTC
ZXDA_2	CTGCGGGCTCGGCTAGCAGG
ZXDA_3	CCGCCGCCGCTAGGTTGATG
ZXDA_4	CCTGCGCAGAGCAGCCCGCG

ZXDA_5	GCAGCCACTTCGAACCCGAG
ZXDB_1	AGGACCGATCAACCTAGCGG
ZXDB_2	CGCCTTGAGGTTGTACACTG
ZXDB_3	GCAGCCACTTCGAACCGGAG
ZXDB_4	TGCGGGGCGATCAGACACCC
ZXDB_5	GGTGCTGTACTTGTGCCCCG
ZXDC_1	CGCGGAGGGCAACATGGCGG
ZXDC_2	GAAAGTGCAGACCTTGGCGC
ZXDC_3	GCTGCCCTGCTGCAGAACCG
ZXDC_4	GGGCAGGACACCTTCGCCGG
ZXDC_5	CACTACGGTCTATAACCTCA
ZZZ3_1	AAGAAGTAGAATCTCGACGC
ZZZ3_2	AAGACCTTGAAAGTTTAGGC
ZZZ3_3	AGATGGTTCAGGTTCTCCGG
ZZZ3_4	GACCAATATACCCATAGCCT
ZZZ3_5	ACAGACGATTGCTGTACTCG
